# Exosomal transfer leads to chemoresistance through oxidative phosphorylation-mediated stemness phenotype in colorectal cancer

**DOI:** 10.7150/thno.84937

**Published:** 2023-09-11

**Authors:** Jinhai Deng, Teng Pan, Chunxin Lv, Lulu Cao, Lifeng Li, Xingang Zhou, Gang Li, Huanxin Li, Jose M Vicencio, Yihan Xu, Fengxiang Wei, Yazhou Wang, Zaoqu Liu, Guanglin Zhou, Mingzhu Yin

**Affiliations:** 1 Clinical Research Center (CRC), Medical Pathology Center (MPC), Cancer Early Detection and Treatment Center (CEDTC), Translational Medicine Research Center (TMRC), Chongqing University Three Gorges Hospital, Chongqing University, Wanzhou, Chongqing, China.; 2 Longgang District Maternity & Child Healthcare Hospital of Shenzhen City (Longgang Maternity and Child Institute of Shantou University Medical College), Shenzhen 518172, China.; 3 Hunan Zixing Intelligent Medical Technology Co., Ltd., Changsha 410221, China.; 4 Richard Dimbleby Laboratory of Cancer Research, School of Cancer & Pharmaceutical Sciences, King's College London, London SE1 1UL, UK.; 5 Oncology Department, Punan Hospital of Pudong New District, Shanghai 200125, China.; 6 Department of Rheumatology and Immunology, Peking University People's Hospital, Beijing 100191, PR China.; 7 Internet Medical and System Applications of National Engineering Laboratory, Zhengzhou, China.; 8 Department of Pathology, Beijing Ditan Hospital, Capital Medical University, Beijing, China.; 9 Department of General Surgery, Peking University Third Hospital, Beijing, China; 10 Department of Chemistry, Physical & Theoretical Chemistry Laboratory, University of Oxford, South Parks Road, Oxford OX1 3QZ, United Kingdom; 11 Cancer Institute, Paul O'Gorman Building, University College London, London, UK.; 12 Chongqing University Medical School, Chongqing 400044, China.; 13 Department of Interventional Radiology, The First Affiliated Hospital of Zhengzhou University, Zhengzhou, China.

**Keywords:** Oxidative Phosphorylation, chemoresistance, PGC-1a, stemness, exosome

## Abstract

**Background:** Recently years have seen the increasing evidence identifying that OXPHOS is involved in different processes of tumor progression and metastasis and has been proposed to be a potential therapeutical target for cancer treatment. However, the exploration in oxidative phosphorylation-mediated chemoresistance is still scarce. In our study, we identify exosomal transfer leads to chemoresistance by reprogramming metabolic phenotype in recipient cells.

**Methods:** RNA sequencing analysis was used to screen altered targets mediating exosome transfer-induced chemoresistance. Seahorse assay allowed us to measure mitochondrial respiration. Stemness was measured by spheroids formation assay. Serum exosomes were isolated for circ_0001610 quantification.

**Results:** The induced oxidative phosphorylation leads to more stem-like properties, which is dependent on the transfer of exosomal circ_0001610. Exosome transfer results in the removal of miR-30e-5p-mediated suppression of PGC-1a, a master of mitochondrial biogenesis and function. Consequently, increased PGC-1a reshapes cellular metabolism towards oxidative phosphorylation, leading to chemoresistance. Inhibition of OXPHOS or exosomal si-circ_0001610 increases the sensitivity of chemotherapy by decreasing cell stemness *in vitro* and *in vivo*.

**Conclusion:** Our data suggests that exosomal circ_0001610-induced OXPHOS plays an important role in chemoresistance and supports a therapeutical potential of circ_0001610 inhibitors in the treatment of oxaliplatin-resistant colorectal cancer by manipulating cell stemness.

## Background

Colorectal cancer is one of the leading causes of cancer-related death, which is highly influenced by metabolic reprogramming 1. Glycolysis is a hallmark of cancer, rewiring energy metabolism to fuel cell growth and division and even modifying tumor microenvironment for immune escape 2,3, with oxidative phosphorylation (OXPHOS) previously being observed to be dysfunctional and regarded as a protective factor in several cancer types 4-6. Indeed, enhancement of OXPHOS Inhibition, due to mutations or decrease in number of mitochondrial DNA (mtDNA), has been observed in a range of cancers and associated with poor prognosis 4,7,8. However, OXPHOS is recently identified as an emerging contributor of tumorigenesis and tumor progression 7,9-11. Especially, the bioenergetic demand of colorectal cancer is more dependent on OXPHOS than that of other cancer types, emphasizing the important roles of OXPHOS in colorectal cancer 12. Notably, cancer stem cells are characterized by increased OXPHOS function 13. For instance, OXPHOS promotes the growth and metastatic potential of colorectal cancer stem cells 14,15. Consequently, OXPHOS inhibitors targeting complex I or complex III of the mitochondrial electron transport chain have also been proved to restrain tumor progression 16,17. Thus, OXPHOS becomes a promising treatment target of colorectal cancer 18.

PPARγ coactivator 1α (PGC-1a) is a master of OXPHOS, stimulating mitochondrial biogenesis that is metabolically more oxidative and less glycolytic in nature 19,20. Cumulative evidence has identified the strong correlation between PGC-1a expression and tumor growth, metastasis and drug resistance 21,22. For instance, loss of PGC-1a reduces the risk of azoxymethane-induced colon carcinogenesis in mice model 19. Compared to primary tumor cells, circulating tumor cells exhibited high levels of PGC-1a-mediated OXPHOS 22. Moreover, PGC-1a-mediated OXPHOS increase is a key player contributing to the resistance to the first-line chemo-therapeutic drugs (oxaliplatin and 5-FU) in colorectal cancer 21. Most importantly, PGC-1a has also shown its close involvement in regulating stem cells 23,24. It is reported to maintain intestinal stem cells by increasing OXPHOS activity and decreasing cellular levels of reactive oxygen species 24. Furthermore, cancer stem cells demonstrate their dependency on PGC-1a-induced mitochondrial OXPHOS 23,25, which leads to tumor relapse 26. Collectively, these data highlight the potential connection between PGC-1a-induced stemness and drug resistance in cancer.

In this study, we show that increased OXPHOS activity induced by the transfer of circular RNAs in exosomes results in chemoresistance of recipient cells in colorectal cancer. Specifically, exosomal circ_0001610 collected from oxaliplatin-resistant cell model leads to the decreased drug sensitivity in recipient cells by upregulating PGC-1a-dependent OXPHOS activity. Consequently, inhibitor of circ_0001610 or loss of PGC-1a reverses the resistant phenotype in recipient cells after exsosomal transfer. Further analysis reveals that the upregulated PGC-1a due to the decrease of miR-30e-5p contributes to drug resistance by inducing stem-like phenotypes, including the overexpression of stemness markers and the increased potential of sphere formation. Finally, we show increased exosomal circ_0001610 and decreased exosomal miR-30e-5p in circulation and overexpressing PGC-1a expression in tumor samples of chemotherapy non-responsive CRC patients compared to that of chemotherapy responsive CRC patients. Taken together, our study suggests a novel mechanism by which increased OXPHOS induced by exosomal transfer can result in chemotherapy-resistance by promoting stem-like properties. Hopefully, this finding can provide a new target for patients unresponsible to chemotherapy.

## Methods

### Clinical specimens

25 colorectal tissue resection and matched blood samples were collected from colorectal cancer patients before neoadjuvant chemotherapy in Peking University Third Hospital. Patient's informed consent forms were signed by all patients. Experiments were approved by Peking university Third Hospital Medical Science Research Ethics committee and performed in accordance with the principle of the Helsinki Declaration II. Information of the human cohorts is provided in Supplementary Table 1.

### Cells and drugs

Human colorectal cancer cell lines DLD1 and HCT15 were acquired from ATCC. Resistant cell lines DLD1/oxaliplatin (DLD1R) and HCT15/oxaliplatin (HCT15R) were established by culturing with constant high oxaliplatin concentration to parental cells for over 12 months. These resistant cell lines were kept in the lab with oxaliplatin and experiments on resistant cell lines were performed after culturing in the medium without oxaliplatin for at least 2-3 weeks. All colorectal cells were maintained at 37°C with 5% CO_2_ in RPMI (Gibco, USA) supplemented with 10% FBS (Gibco, USA) and 1% Penicillin/streptomycin (Gibco, USA) and free of *mycoplasma* throughout the study. Oxaliplatin, GW4869 and Gboxin were purchased from Sigma, USA.

### Conditioned medium and Cell viability assay

Deep Blue Cell Viability^TM^ Kit (BioLegend, USA) was used for cell viability assay. DLD1, DLD1R, HCT15 and HCT15R cells were cultured in complete medium for 48 h and the supernatant (conditioned medium, CM) was collected. DLD1 or HCT15 were cultured with CM or exosomes isolated from CM for 48 h and then tested for cell viability. Briefly, 5,000 cells were reseeded into 96-well plates and incubated overnight. Then, different dose of oxaliplatin was added and incubated for 48 h. Deep Blue Cell Viability^TM^ Kit was added to each well at 1:10 volume ratio and the plates were incubated at 37 °C for 3 h before detection. The optical density (OD) was measured at 450 nm, and the 50% maximum inhibitory concentration (IC50) of the oxaliplatin was calculated from the OD value.

### Cell proliferation assay

DLD1 or HCT15 were cultured with CM for 48 h and then seeded in 96-well plates with the density of 2500 cells per well. After cultured for 1, 2 or 3 days, Deep Blue Cell Viability^TM^ Kit was added to each well at 1:10 volume ratio and the plates were incubated at 37 °C for 3 h before detection.

### EdU Staining

DLD1 or HCT15 were cultured with CM for 48 h and then seeded in 24-well plate with the density of 1×10^4^-2×10^4^ cells per well. The EDU assay was performed by Cell-Lighetm EDU Apollo488 In Vitro Kit (RiboBio, China) according to the manufacturer's protocol.

### Exosome purification and validation

Exosomes from serum were isolated using an exosome isolation kit (Thermo Fisher, USA). Exosomes from CM were purified by ultracentrifugation as previous describe 27. Briefly, cells were incubated in exosome-free medium for 48 h and the supernatant was centrifuged at 2,000 g for 70 min and then 10,000 g for 20 min. Exosomes were pelleted via 110,000 g, 120 min ultracentrifugation. The exosome pellet was washed in PBS and then resuspended in PBS after centrifugation at 110,000 g for additional 60 min. Exosomes were characterized by electron microscopy, nanoparticle tracking analysis (NTA) and western blot. For NTA, exosomes were suspended in PBS and then injected into the sample loading chamber at room temperature (RT). The number and size of exosomes were tracked using a Nanosight NS 300 system (Malvern Technology, UK). Data was analysed using the NTA analytical software (version 2.3).

### PKH26 Staining

PKH26 Red Fluorescent Cell Linker Kits (Sigma, US) was used for lipid bilayer labelling. Exosomes were first resuspended in diluent C and then mixed with the dye solution. After incubating the cell/dye suspension for 5 min with periodic mixing, serum was added to stop the staining. After washed twice with PBS, stained exosomes were added into cell medium and co-incubated for 4 h before standard confocal imaging.

### 3D multicellular tumor sphere formation assay

1,000 vital cells were added into a low-attached 96 well plate (Corning, USA) and then centrifuged at 500 g for 10 min to form spheroids. DLD1 spheroids were formed after 24 h of seeding and HCT15 spheroids were formed after 48 h of seeding.

### Mammosphere formation assay

Cells (1 × 10^3^) were plated in each well of an ultralow 6-well attachment plate (Corning, USA) with DMEM-F12 (Gibco, USA) in serum-free medium, supplemented with EGF (Peprotech, UK) at 10ng/ml final concentration.

### Western blotting

Proteins from cells and tissues were extracted using RIPA buffer (Solarbio, China) and then separated by SDS-PAGE. After proteins were transferred to polyvinyldifluoride membranes (Millipore, USA), 5% non-fat milk was used for blocking. Membranes were incubated overnight at 4 °C with anti-Oct-4 (1:1000, 2750S), anti-PGC-1α (1:1000, 2178S) (both from Cell Signaling Technology, USA), anti-Nanog (1:1000, ab109250) anti-TSG101 (1:1000, ab83) (both from Abcam, UK), anti-CD81 (1:1000, YT5394, Immunoway, USA), and anti-GAPDH (1:5000, 60004-1-Ig, Proteintech, USA). After incubated with peroxidase-conjugated secondary antibody (1:3000; Agilent Technologies, USA) for 2 h at RT, the blots were imaged using a chemiluminescence kit (Millipore, USA).

### RNA sequencing

The RNA-Seq experiments were performed by Novogene (Beijing, China). Briefly, total RNA from CRC cells was isolated using TRIzol reagent (Invitrogen, USA), and cDNA was synthesized using Superscript II reverse transcriptase (Invitrogen, USA) and random hexamer primers. For the data analysis provided by Novogene, base calls were performed using CASAVA. Reads were aligned to the genome using the split read aligner TopHat (v2.0.7) and Bowtie2, using default parameters. HTSeq was used to estimate abundance (Table S3 and S4).

### Metabolism assays

The Oxygen Consumption Rate (OCR) were measured using a Seahorse XF Cell Mito stress test Kit (Agilent, USA) on a Seahorse Extracellular Flux (XF-96) analyser (Seahorse Bioscience, USA) according to the manufacturer's protocol. Briefly, 3 × 10^4^ cells were seeded in 96-well plates and cultured overnight. After washed with XF media, cells were incubated in a CO_2_-free incubator at 37°C for 2 h prior to loading. Afterwards, classical modulators of ETC (electron transport chain) were used to analyze mitochondrial function, including 5 µM oligomycin, 3 µM FCCP, 1 µM antimycin A and rotenone.

### Intracellular ATP measurement

An ATP Detection Kit (Beyotime Biotechnology, China) was used for the measurement of intracellular ATP level. Briefly, 2 × 10^4^ cells were seeded in six-well plates. After adding 200ul of lysis buffer to the cells, the plate was placed on ice for 30 min. The supernatant was then collected after the centrifugation (12,000 g at 4°C for 5 min). 100 µl of ATP detection working solution was then added to the supernatant and incubated for 5 min at RT. 20 µl of samples or standard were added to the detection tubes and relative light unit (RLU) value was detected using a Chemiluminometer.

### RNA extraction and quantitative RT-PCR

RNA isolation, reverse transcription, and real-time qPCR were performed as previously described 27. miRNA was extracted using the miRNeasy Mini Kit (Qiagen, USA) for cell line-derived samples and the miRNeasy Serum/Plasma Kit for patients-derived samples (Qiagen, USA). U6 snRNA was used as an internal control for miRNAs and GAPDH for the circ_0001610 and PPARGC1A mRNA. To standardize exosome-miRNAs, syngeneic cel-miR-39-3p (20 fmol/µl, Qiagen, USA) was added during RNA extraction. The primer sequences used in RT-qPCR are as follows:

5′-TCTGAGTCTGTATGGAGTGACAT-3′ (*PPARGC1A*, sense),

5′-CCAAGTCGTTCACATCTAGTTCA-3′ (*PPARGC1A*, anti-sense),

5′-TGTTCGTCATGGGTGTGAAC-3′ (GAPDH, sense),

5′-ATGGCATGGACTGTGGTCAT-3′ (GAPDH, anti-sense),

5′-CTCGCTTCGGCAGCACA-3′ (U6, sense),

5′-AACGCTTCACGAATTTGCGT-3′ (U6, anti-sense),

5′-GTCTCCCAGAUAAUGCG-3′ (miR-30e-5p, sense),

5′-CGTTCAGGGTCC-3′ (miR-30e-5p, anti-sense),

5′-GGGCTGAAGAAATCCATGAC-3′ (circ_0001610, sense),

5′-TGAGATTCGAGGCCTTCTGT-3′ (circ_0001610, anti-sense).

### Plasmids, oligonucleotides and cell transfection

Lentiviruses to overexpress circ_0001610 were purchased from GenePharma (Shanghai, China). si-circ_0001610, si-NC, miR-30e-5p inhibitors and negative control (NC) were purchased from RiboBio, China. Transient transfection of RNA was performed using Lipofectamine 3000 (Thermo Fisher, USA) according to the manufacturer's protocol. Briefly, cells (2×10^5^) were seeded on 6-well plates one day before transfection with 25 pmol RNAs using 7.5 µl Lipofectamine.

### CRISPR-CAS9 genome engineering

*PPARGC1A* knockouts in CRC cells were accomplished using the Edit-R CRISPR/Cas9 gene engineering protocol (Horizon, UK). All reagents were from Horizon, UK. Cells were transfected in a 6-well plate with crRNA (CM-005111-01-0002 and CM-005111-02-0002): tracrRNA (U-002005-05) transfection complex and Cas9 mRNA (CAS11860), using DharmaFECT Duo Transfection Reagent (T-2010-02). After 48 h, single cells were sorted and cultured in 96-well plates. Western blot and quantitative RT-qPCR assays were used for identifying successful clones after 3-6 weeks.

### Bioinformatics analysis

The miRNA target was predicted using TargetScan (http://www.targetscan.org/vert_72/), DIANA TOOLS (http://diana.imis.athena-innovation.gr/DianaTools/index.php?r=site/page&view=software), miRWalk (Home - miRWalk (uni-heidelberg.de)) and miRDB (https://mirdb.org/). Interactions between miRNA and circRNA were analyzed via STARBASE v3.0 (http://starbase.sysu.edu.cn/).

### Luciferase Reporter Assay

The PCR-amplified fragment containing the 3'-UTR of human *PPARGC1A* or the sequence of hsa_circ_0001610 was inserted into pmirGLO reporter vector (Promega, USA). 100 ng firefly plasmid, 1 ng Renilla plasmid and 100 ng mimics, inhibitors, or NC RNAs were transfected into 5×10^4^ 293T cells using Lipofectamine 3000. Luciferase activity was measured after 48 h using the Luciferase Reporter Assay Kit (Promega, USA).

### RNA pull-down

Dynabeads™ Streptavidin Trial Kit (Invitrogen, USA) was used for RNA pull down assay. Biotinylated miR-30e-5p and its negative controls (GenePharma, China) were transfected into 293T cells respectively. Cells were then lysed, and the supernatant was collected after centrifugation. DynaMag™ Magnet was prepared according to the manufacturer's protocol and co-incubated with the supernatant at RT for 30 min. After three times washes, the beads were resuspended in TRIzol reagent for RNA identification. Quantitative RT-PCR was used to analyse the abundance of circ_0001610 and PPARGC1A mRNA.

### Immunohistochemistry (IHC)

Tumor tissues were fixed with 4% paraformaldehyde, embedded in paraffin as previous describe 28 and stained with anti-PGC-1α (1:200, 66369-1-IG, Proteintech), anti-Oct-4 (1:800, 2750S, cell signaling technology), and anti-Nanog (1:800, ab109250, Abcam). The images were calculated based on H-score. The H-score is calculated as the sum of the percentage of staining multiplied by an ordinal value corresponding to the intensity level (0 = none, 1 = weak, 2 = moderate, 3 = strong). Thus, the H-score ranged from 0 to 300, indicating no staining to diffuse intense staining.

### Tumor xenograft

Five-week-old male BALB/c nude mice (~19-20g) were purchased from SPF (Beijing) Biotechnology Co., Ltd. and kept under specific pathogen-free conditions.

In order to study whether exosomal circ_0001610 is associated with oxaliplatin resistance *in vivo*, tumor xenografts were established in nude mice by injecting 2×10^6^ DLD1 or DLD1R cells into several mice subcutaneously. After 14 days, serum was collected, and exosomes were isolated to measure expression levels of exosomal circ_0001610.

DLD1 cells were treated with circ_0001610 lentivirus or circ_0001610 control lentivirus and then used to implant subcutaneous tumors in nude mice to construct the circ_0001610-positive group, and control group of the mouse xenograft model (n = 5 mice/group). When the transplanted tumor grew to approximately 50 mm^3^, nude mice in each group were treated with 8 mg/kg oxaliplatin every 4 days for a total of 12 days (4 cycles).

si-NC and si-circ_0001610 were transfected into 293T cells to investigate the combined effects of oxaliplatin and exosomal si-circ_0001610. 293T cells were then incubated for 48 h in exosome-free medium. Exosomes were purified from CM. Tumor xenografts were established in nude mice by injecting 2×10^6^ DLD1R cells as mentioned above, using 10 mice in total. Tumor volumes were calculated using the formula: tumor volume (mm3) = 0.5×ab^2^ (a and b are the longest and shortest tumor diameters, respectively). When tumor sizes reached 50 mm^3^, mice were randomly divided into two groups for treatment. The groups exo-si-NC and exo-si-circ_0001610 received s.c. injections around the tumors every 2 days for a total of 12 days. From the same starting time, oxaliplatin was injected intraperitoneally every 4 days for a total of 12 days at the same time. Tumors were collected 2 days after the last treatment. The process of drug administration was performed in a double-blinded manner. All animal experiments were approved by Ethics Review Committee of Shenzhen Longgang ENT Hospital on Laboratory Animal Care (No.2023-0001).

### Statistical analysis

The Cancer Genome Atlas (TCGA) (https://www.genome.gov/Funded-Programs-Projects/Cancer-Genome-Atlas) was used to analyse the correlation between oxidative phosphorylation and stemness in colorectal cancer. All quantitative data are presented as mean ± standard deviation (SD) and were analysed using GraphPad Prism 7.0. Two-way ANOVA was used to evaluate multiple independent groups. Spearman's rank-order correlation was used to determine the correlation. The means of the two datasets were compared using paired t-tests. P-value < 0.05 was considered as statistically significant.

## Results

### Exosomes derived from oxaliplatin-resistant cells cause oxaliplatin resistance and stem-like phenotype in recipient cells

**Figure 1:**
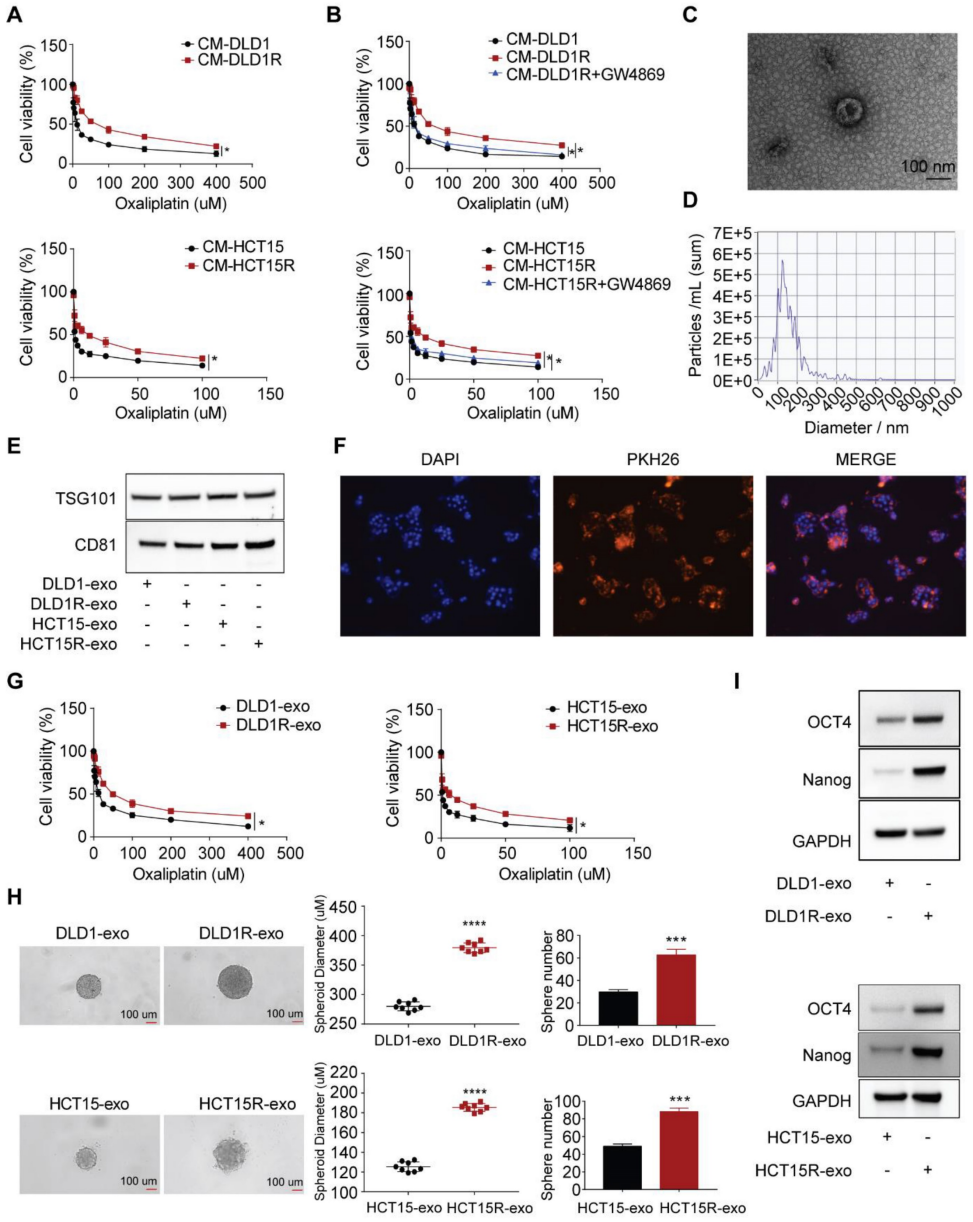
Chemo-resistant cell-derived exosomes (Res-exos) transfer drug resistance by promoting stem-like phenotype in colorectal cancer. (A) Assessment of cell viability of recipient cells after treated with conditioned medium (CM) from either sensitive (DLD1 and HCT15) or resistant cells (DLD1R and HCT15R); (B) Assessment of cell viability of recipient cells after treated with CM from sensitive CRC cells, resistant CRC cells or resistant CRC cells pre-treated with GW4869 (10 µM); (C) Exosomes visualized by transmission electron microscope (scale bar, 100 nm); (D) Nanoparticle tracking analysis (NTA) size profile of exosomes isolated from CM; (E) Western blot of exosome-specific markers (CD81 and TSG101) of collected exosomes; (F) Visualization of internalization of fluorescently labelled exosomes (PKH26) in CRC cells after 48h of incubation by Confocal microscopy; (G) Assessment of cell viability of recipient cells after treated with either Res-exos or Sen-exos; (H) Analysis of Sphere-formation of recipient cells after treated with either Res-exos or Sen-exos; (I) Western blot of OCT4, Nanog and GAPDH in recipient cells after treated with either Res-exos or Sen-exos. *p < 0.05; ****p < 0.0001. Numerical data represent mean± S.D. based on three independent experiments, and the immunoblots are representative of three replicates.

**Figure 2:**
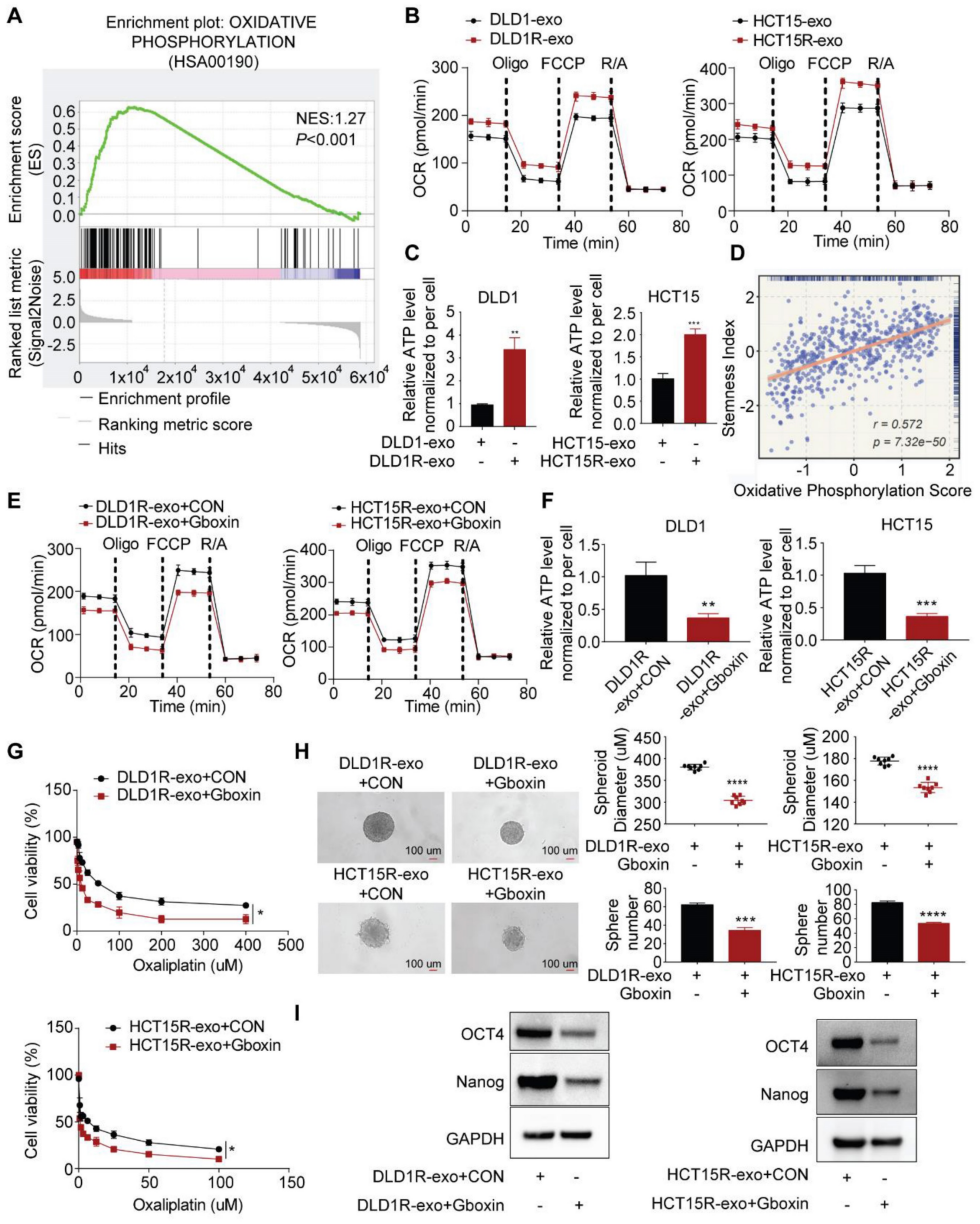
Res-exos transfer the stemness-mediated chemo-resistance by inducing oxidative phosphorylation. (A) Enrichment of oxidative phosphorylation signaling pathway by Gene set enrichment analysis (GSEA) analysis; (B) Assessment of oxygen consumption rate (OCR) of recipient cells after treated with either Res-exos or Sen-exos; (C) Assessment of cellular ATP levels of recipient cells after treated with either Res-exos or Sen-exos; (D) Analysis of the correlation between oxidative phosphorylation score and stemness index in TCGA-COAD dataset; (E-I) Assessment of OCR (E), intracellular ATP measurement (F), cell viability (G), sphere-formation (H) and stemness marker (OCT4 and Nanog) expression in recipient cells after treated with Res-exos with/ without Gboxin (200 nM). *p < 0.05; **p < 0.01; ***p < 0.001. Numerical data represent mean± S.D. based on three independent experiments, and the immunoblots are representative of three replicates.

**Figure 3:**
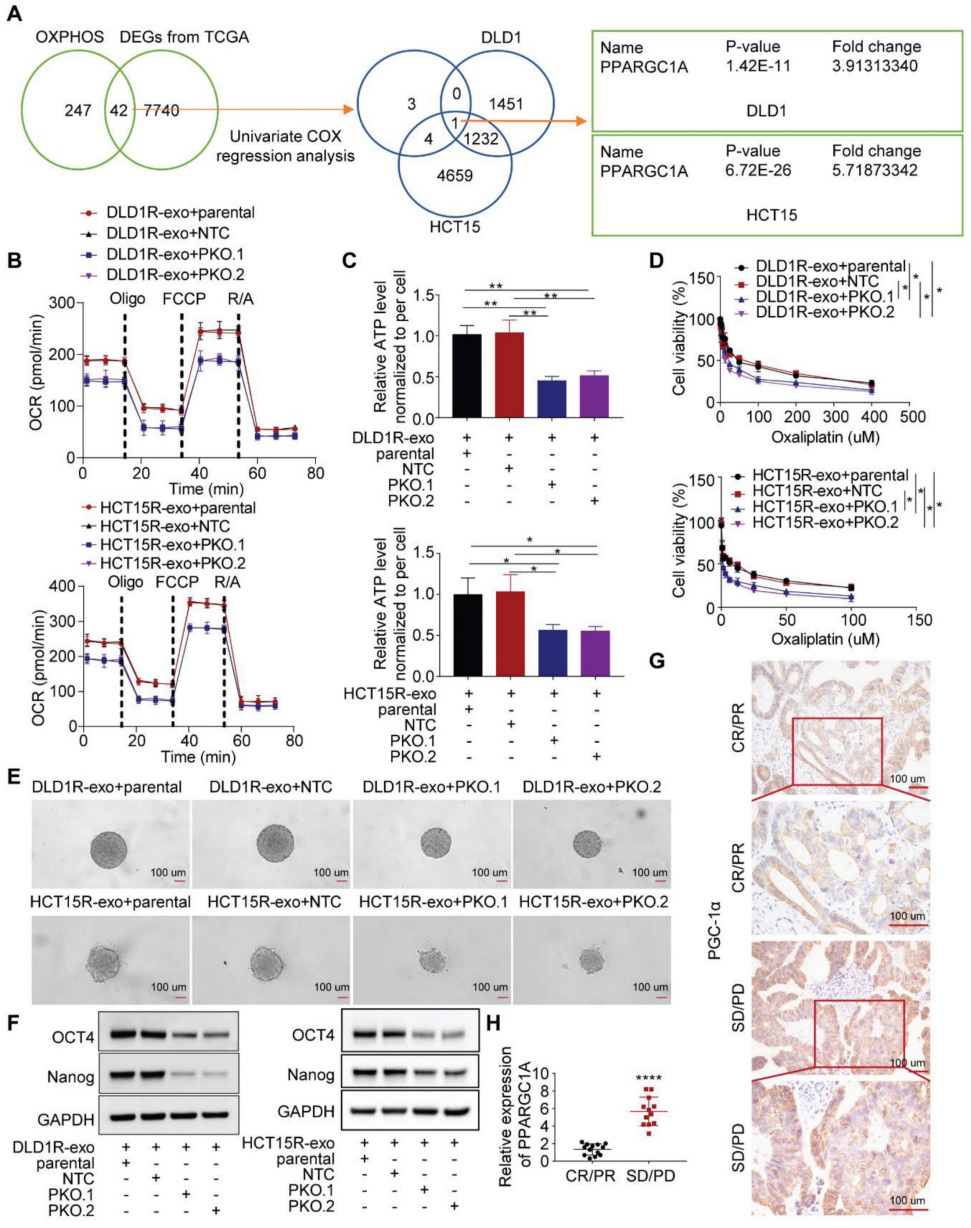
Res-exos transfer promotes oxidative phosphorylation by upregulating PGC-1a expression. (A) Investigation of OXPHOS-related genes after Res-exos transfer. Left venn diagram: analysis of overlapped genes between OXPHOS-related genes and DEGs from TCGA between colorectal cancer samples and normal samples. Eight genes were further selected after Univariate Cox regression analysis39. Right venn diagram: analysis of overlapped genes shared by the above selected eight genes and DEGs of RNA Sequencing data (DLD1 and HCT15); (B-F) Assessment of OCR (B), cellular ATP levels (C), cell viability (D) sphere-formation (E) and the expression of OCT4, Nanog and GAPDH (F) in Parental, non-target control (NTC) and PGC-1α-KO (PKO) cells after 48h of treatment with Res-exos; (G-H) Assessment of PGC-1α expression levels in CRC tumor tissues by IHC (G) and qRT-PCR (H). *p < 0.05; **p < 0.01; ***p < 0.001; ****p < 0.0001. Numerical data represent mean± S.D. based on three independent experiments, and the immunoblots are representative of three replicates.

To investigate the underlying mechanism of oxaliplatin resistance, we initially established oxaliplatin-resistant cell models after culturing parental cells with constant high oxaliplatin concentration over 12 months (Figure S1A). As a result, two resistant cell models were built, including DLD1-OXAR (DLD1R) and HCT15-OXAR (HCT15R) cell models. As shown in Figure S1B, DLD1R and HCT15R exhibited decreased drug sensitivity (using IC50 as readout) compared to their corresponding parental cell models. Similar to our previous work 27, the recipient cells (sensitive cells) exhibited decreased drug sensitivity to oxaliplatin when co-cultured with conditioned medium from resistant cells (DLD1R or HCT15R) than conditioned medium from corresponding sensitive cells (DLD1 or HCT15) (Figure 1A). Further, to investigate whether these effects were dependent on the cell types, we collected the CM from resistant cells, which were different from their parental ones, to treat the recipient cells (for example, CM derived from DLD1R/HCT15R to HCT15/DLD1, respectively). Correspondingly, the results showed the similar effects, suggesting that the transfer of drug resistance was not cell type-dependent. This result highlights the existence of the transfer of chemo-resistance within different subtypes of tumor cells independent of specific cell types, potentially resulting in the wide-spreading drug resistance effects (Figure S1C). It is reported that exosomes are one of the most important message-transferring pathways to mediate therapy resistance 29. To determine the role of exosomes in this observation, we applied GW4869, an inhibitor used for blocking exosome generation 30, to pre-treat resistant cells before collecting conditioned medium. Strikingly, the induced drug resistant phenotype in recipient cells was remarkably compromised under exosomes secretion-inhibited condition (Figure 1B). On this basis, we hypothesized that exosomes from conditioned medium were responsible for transferring drug resistance in recipient cells. To further verify this hypothesis, we isolated conditioned medium-derived exosomes by ultracentrifuge method. Exosomes identity was confirmed by 1) transmission electron microscope analysis demonstrating the classic exosome morphology (cup-shaped form), ranging from 30 to 150 nm in size (Figure 1C); 2) nanoparticle particle tracking analysis (NTA), combined with a Nanosight system, showed that the modal size of the vesicles was around 100 nm without much contamination of larger vesicles (Figure 1D); 3) immune-blotting analysis successfully stained the samples with exosome-specific markers ( both TSG101 and CD81) (Figure 1E).

Next, we tested whether exosomes can be successfully transferred to recipient cells. Exosomes from conditioned medium of resistant cells were labelled with the fluorescent tracer PKH26 and then co-cultured with recipient cells. Strong red fluorescence (PKH26) was observed in the cytoplasm of recipient cells visualized by confocal microscopy, indicating that exosomes released from donor cells are capable of being up taken by recipient cells (Figure 1F). To confirm the requirement of exosomes in mediating drug resistance, more assays were then performed to provide direct evidence. We treated sensitive cells with exosomes collected from resistant (Res-exos, including DLD1R-exo or HCT15R-exo) or sensitive cells (Sen-exos, including DLD1-exo or HCT15-exo) and observed significant higher viability of recipient cells in the presence of varies doses of oxaliplatin when co-cultured with Res-exos than with Sen-exos (Figure 1G). In light of the key role of cancer stem cells in chemotherapy resistance 31-33, we speculated stemness may account for exosome-mediated oxaliplatin resistance in our system. By using 3D spheroids as an *in-vitro* model 34, we observed Res-exos treatment enhanced the efficiency of sphere formation of recipient cells (Figure 1H). Consistently, the results also showed the recipient cells treated with Res-exos presented higher expression of stemness-associated markers, including Oct4 and Nanog (Figure 1I). Notably, cell proliferation and EdU assays showed that treatment of sensitive cells with Res-exos led to increased proliferative capacity compared to treatment with Sen-exos (Figure S1D-E). Collectively, these data illustrated that the transfer of exosomes mediated chemoresistance in recipient cells by promoting stem-like properties.

### Exosomes transfer chemoresistance by enhancing oxidative phosphorylation (OXPHOS) activity

To understand the underlying mechanism, we collected recipient cells after incubation with exosomes for next-generation sequencing. Gene set enrichment analysis (GSEA) demonstrated a strong enrichment of oxidative phosphorylation genes in recipient cells in the presence of Res-exos (Figure 2A, Figure S2). It is interesting as oxidative phosphorylation has recently been identified to be critical for cancer stemness and drug resistance 35,36. We next validated sequencing data by assessing the alteration of OXPHOS activity and cellular ATP levels 37. As expected, our results suggested that Res-exos - treated cells exhibited a higher respiration rate and increased ATP levels (Figure 2B-C). Thus, these results demonstrated Res-exos induced the activation of OXPHOS in recipient cells. Notably, we observed the significantly positive correlation between stemness score and oxidative phosphorylation score from TCGA-CRC analysis (Figure 2D).

To establish whether the induced OXPHOS is responsible for drug resistance and increased stemness in our system, Gboxin, reported as a small molecule specifically inhibiting OXPHOS, was exploited for subsequent analyses 37,38. Indeed, Gboxin treatment decreased respiration rate and cellular ATP levels of sensitive cells co-cultured with Res-exos in both cell lines (Figure 2E-F), confirming its capacity of OXPHOS blockade. Further analysis revealed Gboxin treatment led to compromised drug resistance and spheres-forming ability induced by Res-exos (Figure 2G-H). Accordingly, the expression of stemness-associated markers was downregulated by Gboxin in the presence of Res-exos treatment (Figure 2I). All together, these results provided the evidence that increased oxidative phosphorylation mediated Res-exos-induced oxaliplatin resistance and cell stemness.

### PGC-1a masters OXPHOS increase and associated drug resistance

Next, we sought to investigate the molecular mechanism. In colorectal cancer, forty-two OXPHOS-related genes were differentially expressed between tumor and normal samples, with 8 genes showing prognostic value 39. Combined with our sequencing data, we identified that one target, *PPARGC1A*, was significantly upregulated in recipient cells treated with Res-exos (Figure 3A). PGC-1a (encoded by *PPARGC1A*) is a master of OXPHOS and has been proved to promote chemoresistance and stemness in colorectal cancer 21,23. Therefore, we questioned whether PGC-1a mediated the induced OXPHOS activity and associated drug resistance. Firstly, we tested the expression level of PGC-1a in different cell models and found increased level of PGC-1a in resistant cells than sensitive cells (Figure S3A). Then, we confirmed the increased expression of PGC-1a in recipient cells incubated with Res-exos than with Sen-exos (Figure S3B-C). Moreover, knocking-out of PGC-1a in recipient cells reduced the increased respiration rate and cellular ATP levels by Res-exos transfer (Figure 3B-C, Figure S3D). In accordance with OXPHOS alteration, loss of PGC-1a removed the effects of Res-exos transfer in terms of drug sensitivity (Figure 3D) and stemness properties (Figure 3E-F, Figure S3E-F). To further extrapolate the utilization of PGC-1a as a biomarker for the prediction of clinical treatment, tumor samples (pre - chemotherapy) were collected from 25 patients with available clinical outcome data (Table S1). Remarkably, we observed higher expression of PGC-1a in tumor samples from non-responders (Progressive Disease (PD)/ Stable Disease (SD)) than responders ((Complete Response (CR)/ Partial Response (PR)) (Figure 3G-H, Figure S3G).

### The regulation of PGC-1a is dependent on circ_0001610/ miR-30e-5p axis

**Figure 4:**
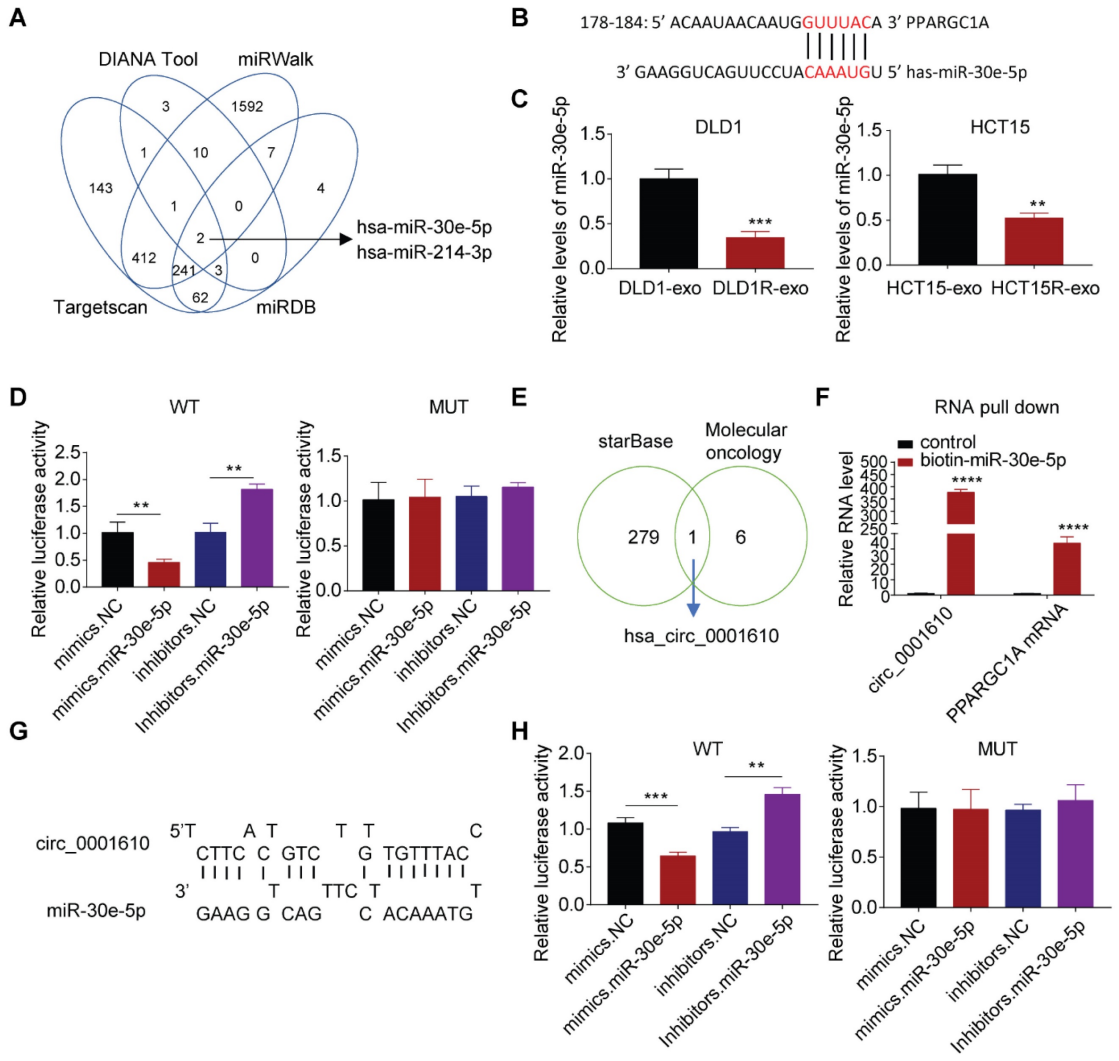
PGC-1a expression is regulated by circ-0001610/ miR-30e-5p axis. (A) Prediction of miRNAs targeted at PPARGC1A by bioinformatic analysis; (B) Predicted binding sites of miR-30e-5p within the 3'UTR of PPARGC1A mRNA; (C) Measurement of miR-30e-5p expression in recipient cells after treatment with either Res-exos or Sen-exos; (D) Investigation of direct binding between miR-30e-5p and the PPARGC1A 3′-UTR using luciferase reporter assay; (E) Analysis of overlapped targets between circRNAs which can directly adsorb miR-30e-5p (starBase) and circRNAs mediating treatment-resistant stemness phenotype45; (F) Detection of the relative level of circ_0001610 and PPARGC1A mRNA after transfection with either biotin-NC or biotin-miR-30e-5p in 293T cells by RNA oull-down assay; (G) Prediction of binding sites between miR-30e-5p and circ_0001610; (H) Assessment of direct recognition of circ_0001610 by miR-30e-5p using luciferase reporter assay. **p < 0.01; ***p < 0.001; ****p < 0.0001. Numerical data represent mean± S.D. based on three independent experiments, and the immunoblots are representative of three replicates.

We further explored whether the alteration of miRNAs resulted in the dysregulation of PGC-1a since miRNAs can repress the expression of target mRNAs by directly inducing instability and has been widely studied in drug resistance 40,41. Bioinformatics analysis predicted two candidates can target PPARGC1A mRNA, with miR-30e-5p exhibiting higher Context++score percentile than miR-214-3p (76 *vs.* 61) (Figure 4A, Figure S4A). More analyses exhibited the binding between miR-30e-5p and PPARGC1A 3′-UTR mRNA (Figure 4B), which is highly conserved across different species (Figure S4B). Especially, miR-30e-5p potential has been reported to be a tumor suppressive regulator, inhibiting tumor cell growth, invasion and cancer stemness 42 and observed to be downregulated after Res-exos treatment (Figure 4C). Accordingly, we tested the abundance of miR-30e-5p and observed lower levels of miR-30e-5p in resistant cells than that in sensitive cells (Figure S4C). Thus, we focused on miR-30e-5p in the subsequent analyses. To confirm the capacity of miR-30e-5p to bind PPARGC1A 3′-UTR mRNA, luciferase reporter assay was performed. The results showed that the relative luciferase activity was massively inhibited with co-transfection of miR-30e-5p mimics, but significantly increased after co-transfected with miR-30e-5p inhibitors (Figure 4D). However, the interactive effects were abolished when treated with a plasmid carrying a mutated sequence (Figure 4D). Furthermore, we transfected tumor cells with miR-30e-5p inhibitors, which showed satisfied transfection efficiency (Figure S4D). As expected, compared to control group, we observed increased PGC-1a expression at both protein and mRNA levels when transfected with miR-30e-5p inhibitors, respectively (Figure S4E and F), suggesting that miR-30e-5p is capable of regulating PGC-1a expression. Further analysis showed that the compromised effects on Res-exos-induced OXPHOS and drug resistance were largely recapitulated by miR-30e-5p inhibitors treatment. However, miR-30e-5p inhibitors-mediated effects were abolished in PGC-1a-KO cell models (Figure S4G-J). Moreover, the expression of miR-30e-5p was analysed in the same set of clinical tumour samples and was observed to be highly expressed in responders than non-responders (Figure S4K). Consistently, miR-30e-5p levels have been shown to be negatively correlated with *PPARGC1A* expression (Figure S4L), highlighting the close association between miR-30e-5p and PGC-1a. To be noted, the effects of miR-214-3p were also tested. Cell viability assay showed that miR-214-3p inhibitors treatment rescued the effects on Res-exos-induced drug resistance in DLD1 cells, but to a lesser extent. Moreover, the loss of miR-214-3p showed no obvious effect in HCT15 cells, which suggested that the role of miR-214-3p was inconsistent across different colorectal cancer cell lines in regulating PGC-1a expression (Figure S4M-N). Collectively, even though miR-214-3p may play a modest role in drug resistance, miR-30e-5p is the key one in our system, confirming that oxaliplatin resistance is mainly mediated via miR-30e-5p.

Circular RNAs (circRNA) are a class of non-coding RNAs, with strong potency to bind and sequestrate miRNAs 43. Since circRNAs can be packaged into exosomes and mediate intercellular communication within tumor microenvironment 44. We then determined whether the increased drug resistance is due to the transfer of exosomal circRNAs. Bioinformatic analysis identified circ_0001610 as a potential player in our system for two reasons. Firstly, it is one of circRNAs that can absorb miR-30e-5p by starBase analysis; secondly, it is part of cancer stemness signature 45 (Figure 4E, Table S2). Notably, circ_0001610 has been identified to promote tumor progression and drug resistance in various cancer types 46. Expectedly, the binding between circ_0001610 and miR-30e-5p was confirmed by RNA pull-down assay, showing increased capture of circ_0001610 by biotin miR-30e-5p compared to biotin-NC (Figure 4F). Moreover, the binding between miR-30e-5p and PPARGC1A mRNA was also confirmed (Figure 4F). Further analysis by luciferase assay exhibited compromised binding effects when mutations were introduced in the sequences of binding sites (Figure 4G-H).

### Exosomal transfer of circ_0001610 resulted in increased PGC-1a and stemness phenotype by supressing miR-30e-5p

**Figure 5:**
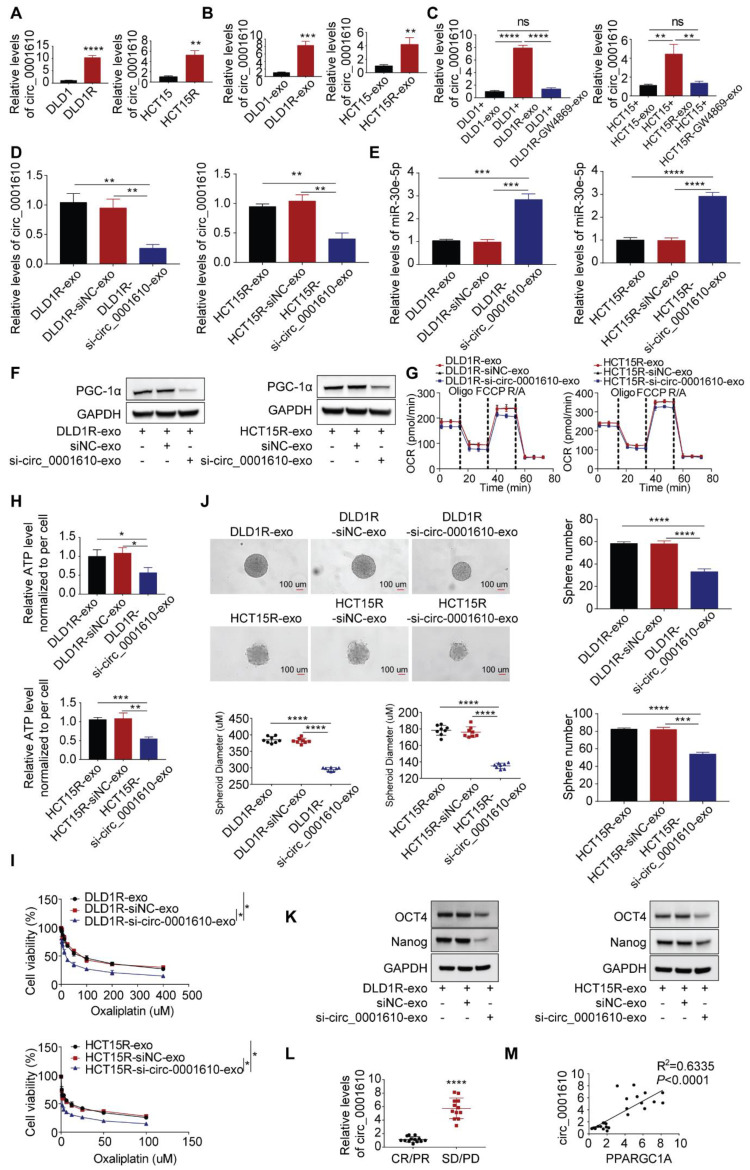
Exosomal circ_0001610 is responsible for Res-exos-mediated chemo-resistant phonotype. (A-C) Assessment of circ_0001610 expression by qRT-PCR analysis in sensitive and resistant cells (A), in exosomes collected from sensitive and resistant cells (B), in recipient cells after treatment with Sen-exos or Res-exos or Res-exos pre-treated with GW4869 (10 µM) (C). (D) Verification of the efficiency of si-circ_0001610 in recipient cells after Res-exos treatment with pre-transfection of si-NC or si-circ_0001610; (E) Measurement of miR-30e-5p expression in recipient cells after Res-exos treatment with pre-transfection of si-NC or si-circ_0001610; (F-K) Assessment of PGC-1a expression (F), OCR (G), cellular ATP levels of sensitive cells (H), cell viability (I), sphere-formation (J) and the expression of stemness markers (Oct4 and Nanog) (K) in recipient cells after Res-exos treatment with pre-transfection of si-NC or si-circ_0001610; (L) Measurement of exosomal circ_0001610 in serum samples of patients with different responses to chemotherapy (n=25); (M) Analysis of the correlation between exosomal circ_0001610 and PPARGC1A expression in 25 CRC clinical samples. *p < 0.05; **p < 0.01; ***p < 0.001; ****p < 0.0001. Numerical data represent mean± S.D. based on three independent experiments, and the immunoblots are representative of three replicates.

We subsequently tested the abundance of circ_0001610 in different cell models and found out that the levels of circ_0001610 were higher in resistant cells than in sensitive cells (Figure 5A). The similar trend was observed in cell-derived exosomes, namely circ_0001610 was more enriched in Res-exos than in Sen-exos (Figure 5B). Moreover, recipient cells demonstrated significantly upregulated levels of circ_0001610 after incubation with Res-exos, suggesting high rate of exosomal circ_0001610 uptake by recipient cells (Figure 5C). However, the increased circ_0001610 in recipient cells was remarkably compromised after pre-treating resistant cells with GW4869 before collecting exosomes, indicating the upregulation of circ_0001610 in recipient cells was induced by exosomes (Figure 5C). Furthermore, the treatment of cell-secreted Res-exos collected after circ_0001610 knockdown by siRNA, with knocking-down efficiency verified by qPCR (Figure 5D), eliminated the suppressive expression of miR-30e-5p in recipient cells (Figure 5E), which further led to decreased expression of PGC-1a (Figure 5F), confirming exosomal transfer of circ_0001610 exerting the impacts on miR-30e-5p/PGC-1a axis. In accordance, we also observed that knock-down of exosomal circ_0001610 can compromise the induced oxidative phosphorylation, stemness properties and even chemoresistance compared to control group (Figure 5G-K), emphasizing the essential role of exosomal circ_0001610 in mediating stemness and oxaliplatin resistance. The detectability of circRNAs in liquid biopsy (including serum or plasma) endows themselves with the strong potential as powerful diagnostic and prognostic biomarkers 47. On this basis, we collected matched serum samples from these 25 patients for exosome extraction. Obviously, qPCR analysis demonstrated higher expression of circ_0001610 in serum exosomes from chemo-resistant patients (PD/SD) than from chemo-sensitive counterparts (CR/PR) (Figure 5L), revealing the prognostic potential of circulating exosomal circ_0001610 in colorectal cancer. Moreover, the expression level of exosomal circ_0001610 is positively correlated with *PPARGC1A* expression (Figure 5M). Taken together, our data confirmed exosomal circ_0001610 confers chemoresistance in recipient cells.

### Treatment of exosomal si-circ_0001610 sensitizes the response to Oxaliplatin *in vivo*

**Figure 6:**
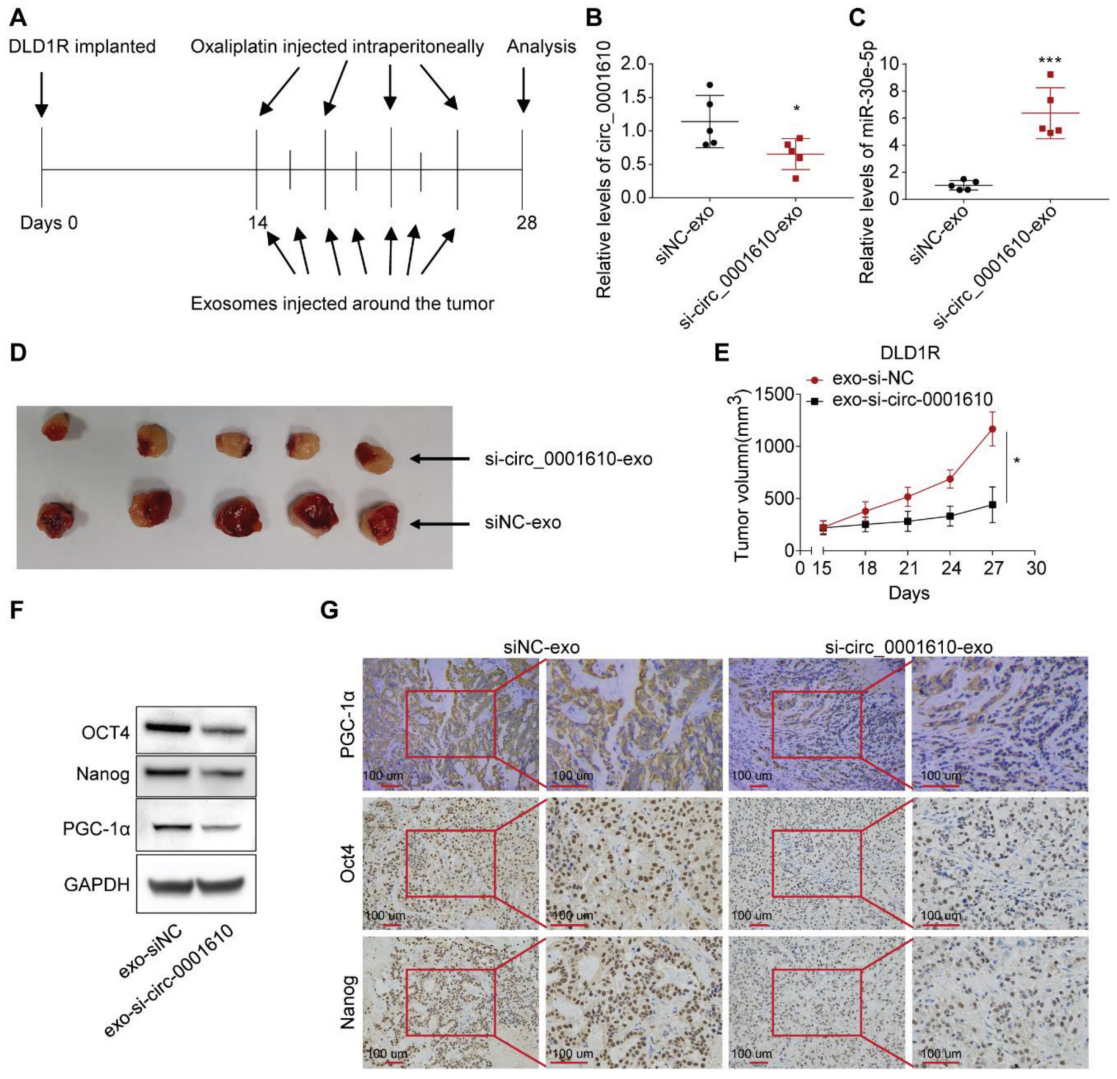
Systemically injected exosomes pre-loaded with si-circ_0001610 sensitizes the response to oxaliplatin in vivo. (A) Diagram depicting the in vivo experimental design; (B) Measurement of the expression levels of circ_0001610 in serum exosomes from mice by qRT-PCR; (C) Measurement of the expression levels of miR-30e-5p in tumor tissues from mice by qRT-PCR; (D) Images of tumors in mice (n = 10) after treatment with siNC-exo or si-circ_0001610-exo; (E) Quantification of tumor volumes in mice after treatment with siNC-exo or si-circ_0001610-exo; (F) Assessment of the expression levels of PGC-1α and stemness markers in tumors; (G) IHC analysis of PGC-1α and stemness markers in tumors. *p < 0.05; ***p < 0.001.

To investigate the effects of circulating exosomal circ_0001610 on chemoresistance *in vivo*, we established DLD1 and DLD1R xenografts by injecting tumor cells in nude mice (see materials and methods for details). After two weeks, serum exosomal circ_0001610 was measured and observed to be markedly increased in DLD1R xenograft mice than DLD1 xenograft mice (Figure S5A). Further, to verify the effects of circ_0001610 on oxaliplatin resistance *in vivo*, xenograft models were established (see materials and methods for details). Circ_0001610 overexpression (circ_0001610 OE) group showed a lower sensitivity to oxaliplatin and an increase in tumor growth rate and volume (Figure S5B-C). qRT-PCR confirmed the high expression levels of circ_0001610 in circ_0001610 OE tumor tissues (Figure S5D). These data confirmed that circ_0001610 could induce chemoresistance *in vivo*. It is clear that exosomes belong to a class of nanoparticles, which are useful tools as cancer therapeutics 48. Thus, we sought to understand the therapeutic effects of exosomal si-circ_0001610 by using tumor-implanted mice. 14 days after DLD1R cells were implanted in nude mice when tumor is just visible, we isolated exosomal si-circ_0001610 or negative control from 293T cells and treated tumor-bearing mice with systemic injections of oxaliplatin (*i.p.*) combined with isolated exosomes (exosomal si-circ_0001610 or negative control) around the tumor (Figure 6A). Afterwards, 7 cycles of exosome treatment and 4 cycles of oxaliplatin treatment were conducted in additional two weeks (Figure 6A). Serum exosomes and tumor tissues were then analysed, and the data confirmed decreased circ_0001610 levels in serum exosomes and increased miR-30e-5p levels in tumor tissues after injection of exosomal si-circ_0001610 (Figure 6B, C). Moreover, we measured the size of tumors every 3 days and observed that the suppression of circ_0001610 reduced the tumor burden clearly, indicating that exosomal si-circ_0001610 can sensitize the chemotherapy response of resistant models *in vivo* (Figure 6D, E). Additionally, we performed assays to test the molecular relevance. By both immune-blotting (Figure 6F) and immunohistochemistry staining (Figure 6G), decreased expression of PGC-1a and stemness markers was observed in tumors with exosomal si-circ_0001610, confirming the regulation of stemness by exosomal circ_0001610. In summary, these *in vivo* findings confirmed the contribution of circ_0001610/miR-30e-5p/PGC-1a axis to chemotherapy resistance in colorectal cancer.

## Discussion

**Figure 7:**
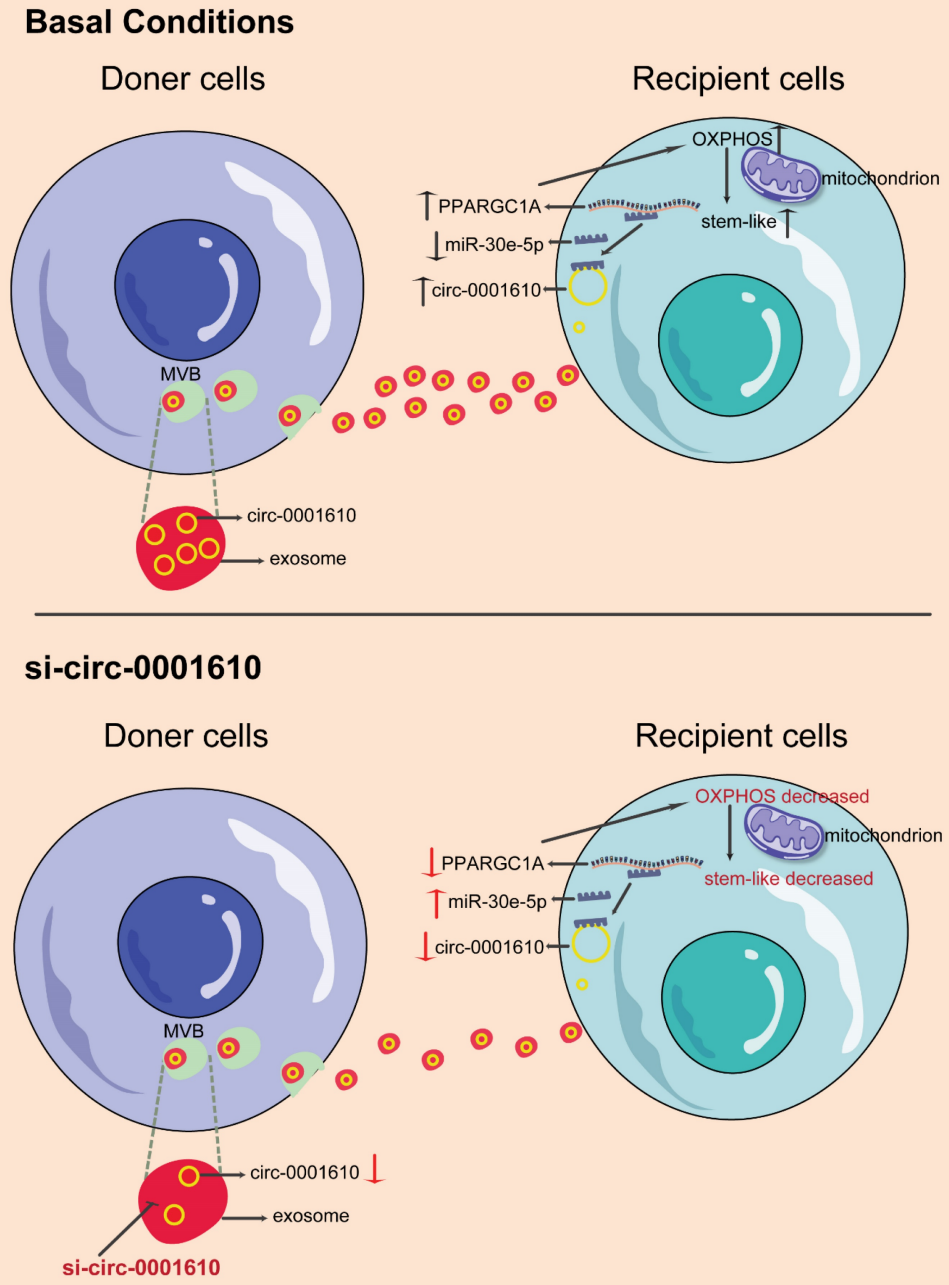
Schematic diagram of the role of exosomal circ_0001610 in transferring an oxaliplatin-resistant phenotype in colorectal cancer.

Cellular metabolism has been well-known to regulate cancer progression and drug sensitivity, thus making it new therapeutic strategies 22,49,50. Even though cancer cells preferentially use glycolysis, oxidative phosphorylation has recently attracted more attention in the community as a therapeutic target due to its important roles in tumorigenesis and drug resistance 11,36,51. Of note, OXPHOS has been confirmed to maintain and promote the function of cancer stem cells by providing required metabolic and energy needs 36. Indeed, convincing evidence demonstrate that cancer stem cells promote the tumorigenesis, metastasis and drug resistance even though they just account for a small subpopulation in colorectal cancer 52-54. However, the understanding of the role of OXPHOS-induced stemness in chemoresistance is still elusive. In this study, our data, for the first time, revealed increased mitochondrial OXPHOS activity endowed recipient cells with decreased drug sensitivity to chemotherapy after exosomal circRNAs transfer from drug resistant cells, highlighting a novel mechanism regarding the crosstalk among tumorous subpopulations in the heterogenous tumor microenvironment (Figure 7). Thus, this study, consistently, emphasizes the contribution of the interplay between different subgroups in the cancer prognosis by modulating cellular metabolic patterns 55,56.

Exosomes are one type of extracellular vesicles with ~100 nm in size, signalling among different cells 27,56. Several components within exosomes can show significant effects by transferring messages between tumor cells/ immune cells/ fibroblasts and tumor cells/ immune cells/ fibroblasts 57, including exosomal genomic DNA 58, mRNA 59, miRNA 60, circRNA 61 and even proteins 62. Transfer of these biologically functional molecules can change the properties of the recipient cells by manipulating the downstream signalling 63. For example, exosomal miRNAs have been established to mediate tumorigenesis and drug resistance 27,64,65. Our previous work identified horizontal transfer of exosomal miR-181b-5p leads to doxorubicin resistance by inhibiting the expression of p53 and cellular senescence in breast cancer 27. CircRNAs are another class of non-coding RNAs and exosomal circRNA (exo-circRNA) released from oxaliplatin-resistant colorectal cancer cells has previously been shown to confer sensitive cells more resistant to oxaliplatin by regulating glycolytic state, suggesting circRNAs play essential roles in the promotion of resistance to oxaliplatin in colorectal cancer 61. Consistently, our present study also shows exosomes, instead of soluble factors, are the main messengers effectively exerting effects on the recipient cells for changes in terms of cellular phenotypes and drug sensitivity in colorectal cancer.

Here, we now show that oxaliplatin-resistant (OXAR) - secreting exosomal circRNA can be up-taken by recipient cells, which leads to increased OXPHOS activity and more stem-like phenotype. Mechanistically, we found exo- circ_0001610 helps to stabilize *PPARGC1A* mRNA by competing with miR-30e-5p and thus increase the protein level of PGC-1a, which further promotes the increased level of oxidative phosphorylation. PGC-1a is a main regulator of OXPHOS and has been well studied in cancer with controversial roles 66. It has been clearer that PGC-1a is indispensable in cancer stemness and drug resistance 23,67-69. Accordingly, PGC-1a overexpression in recipient cells switches them to a state with higher oxidative phosphorylation activity, which facilitates the production of stem-like cells and increased oxaliplatin resistance. Notably, due to the dual roles of PGC-1a in cancer, the modulation of PGC-1a in treating drug resistance should be more specific. Several tools could be potentially applied, including nanoparticles, which has shown great efficacy in specifically targeting tumor cells 70-72. Moreover, loss of PGC-1a reversed miR-30e-5p-mediated chemo-sensitizing effects. Further *In vivo* experiments have also been performed to verify the effects of exo- circ_0001610/miR-30e-5p/PGC-1a axis. Inhibitors of exo- circ_0001610 decreased PGC-1a expression, resulting in the restriction of tumor growth and prolonged survival. Additionally, detection of both cellular and exosomal circRNA showed more abundance in resistant cells than corresponding sensitive cells.

Importantly, previous studies have proved that the transfer of exosomal circ_0001610 is capable of decreasing the sensitivity to treatment. For instance, the expression of circ_0001610 was increased in the grade 3 endometrial cancer (EC) 73. The loss of circ_0001610 repressed the progression of EC 74, suggesting that circ_0001610 acts as a tumor-promoting factor. Moreover, TAM-derived exosomal circ_0001610 diminished the efficacy of radiotherapy in endometrial cancer cells 46. These studies suggest the role of circ_0001610 in reducing therapeutic sensitivity. Our clinical analysis confirmed higher concentration of exo- circ_0001610 in circulation was associated with significant poor response to oxaliplatin-based chemotherapy by analysing 25 patient circulating samples. This observation is consistent with previous studies, suggesting the reasonability of proposing circulating exosomal circ_0001610 as a potential prognostic biomarker in colorectal cancer 75. Due to the development of powerful technologies, including nanotechnology and the combination of exosomes and RNA interference 76,77, it is foreseeable to use circ_0001610 as a target for the establishment of treatment regimen.

Taken as a whole, our data demonstrate the exosomal circRNA transfer leads to stem-like phenotype by modulating cellular metabolism in the recipient cells. This study provides insight into metabolic shifts in sensitive cells to promote chemotherapy resistance, and also suggests circ_0001610 as a potential target for treating patients who fail to benefit from chemotherapy.

## Supplementary Material

Supplementary figures and tables.
