# Supplementary material for: Exosomal transfer leads to chemoresistance through oxidative phosphorylation-mediated stemness phenotype in colorectal cancer
Source: Theranostics. 2023 Sep 11;13(14):5057–74. doi: 10.7150/thno.84937 (PMC10526671; doi:10.7150/thno.84937)
Supplement: Supplementary file 1 — Supplementary figures and tables. [file thnov13p5057s1.pdf]

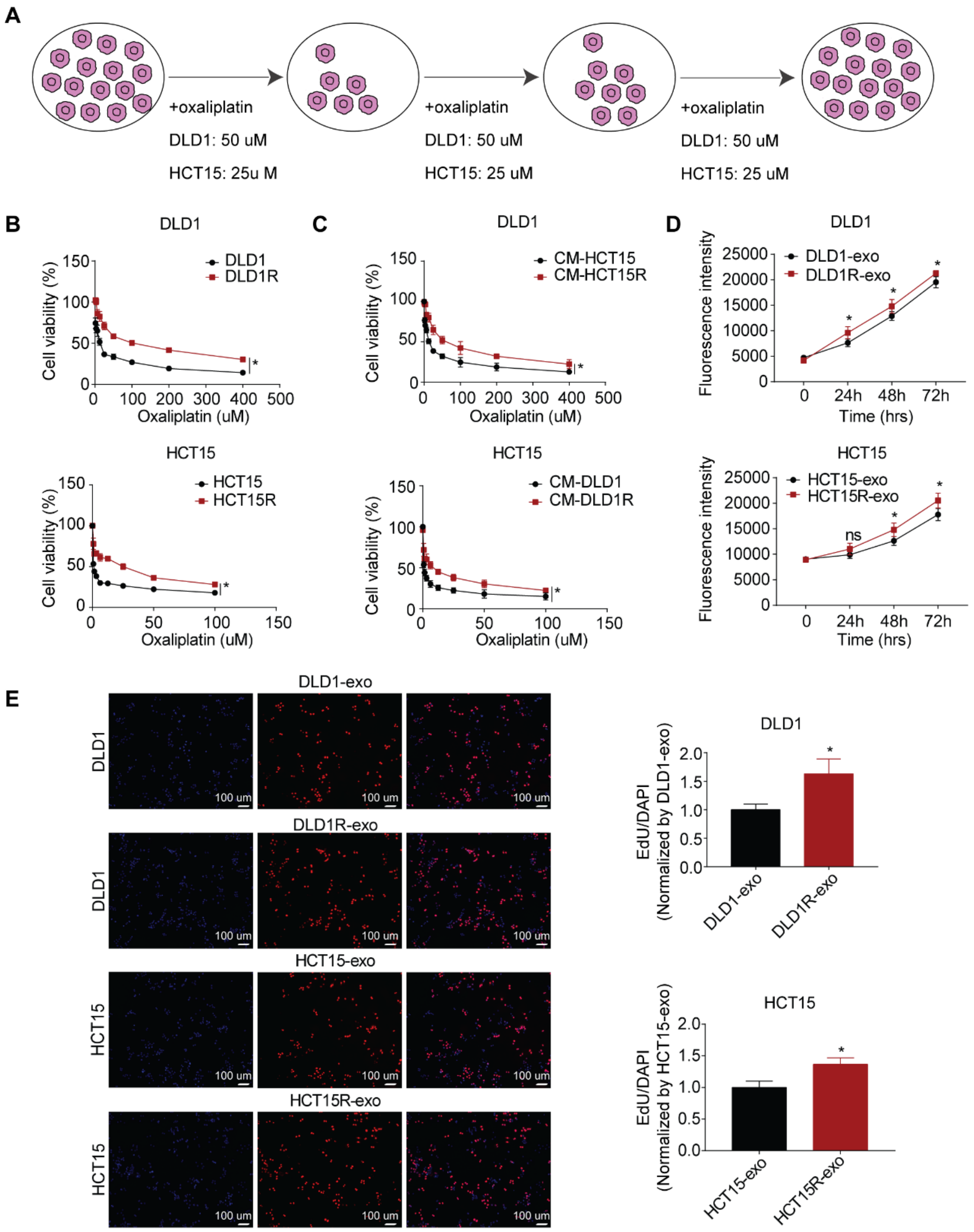

Figure S1: Establishment of oxaliplatin-resistant cell models

(A) A Schematic model depicting the process of acquiring oxaliplatin-resistant CRC cells. (B) Assessment of IC50 values of oxaliplatin of oxaliplatin-resistant cells and their parental cells. (C) Assessment of cell viability of recipient cells after treated with conditioned medium (CM) from either sensitive (DLD1 and HCT15) or resistant cells (DLD1R and HCT15R). (D) The growth curve of recipient cells after CM treatment. (E) EdU incorporation assays were performed to visualize (left panel) and quantify (right panel) cell proliferation. *$p < 0.05$. Numerical data represent mean± S.D. based on three independent experiments

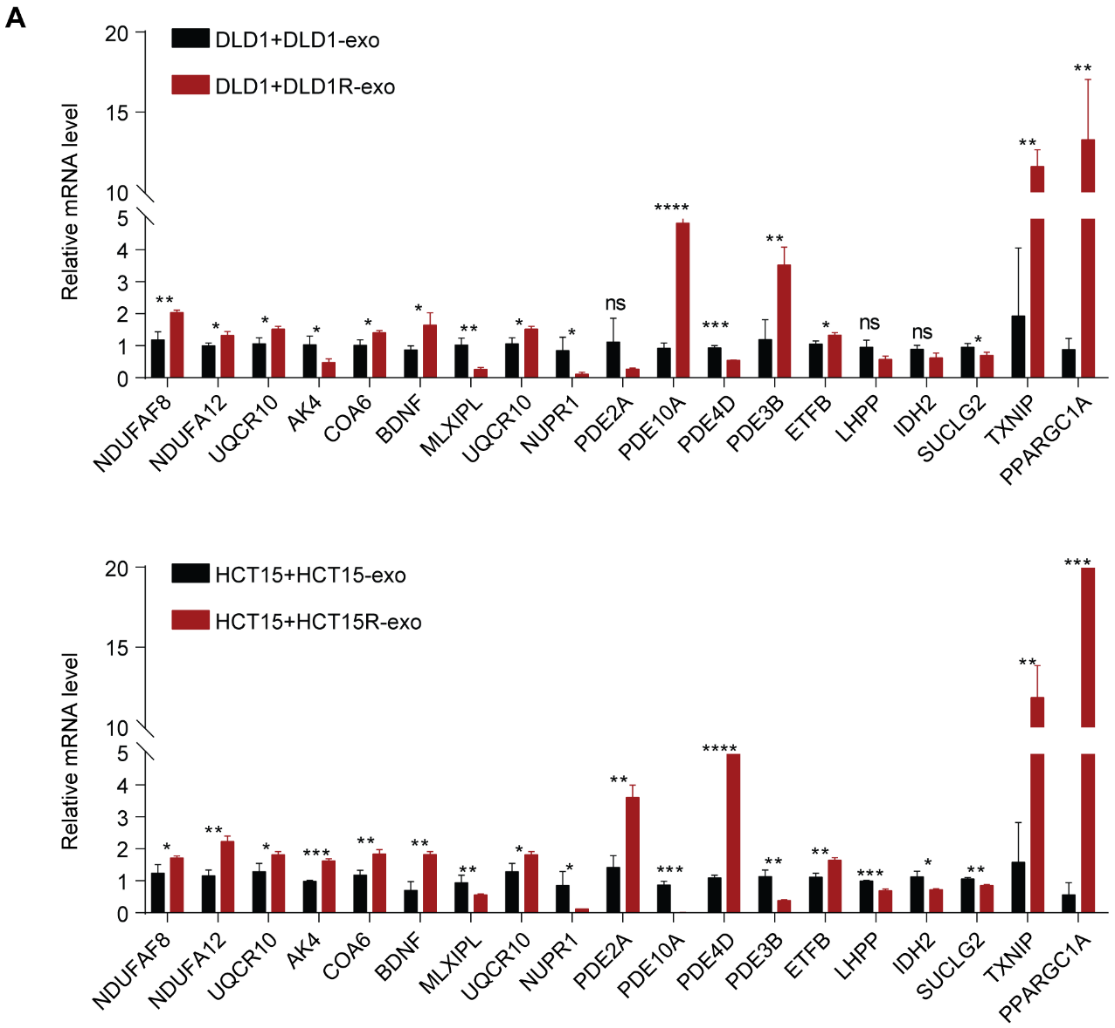

Figure S2. Transfer of Res-exos induces the expression of OXPHOS-related genes in recipient cells. Analysis of the expression of OXPHOS-related genes from RNA sequencing data. *p < 0.05; **p < 0.01; ***p < 0.001; ****p < 0.0001.

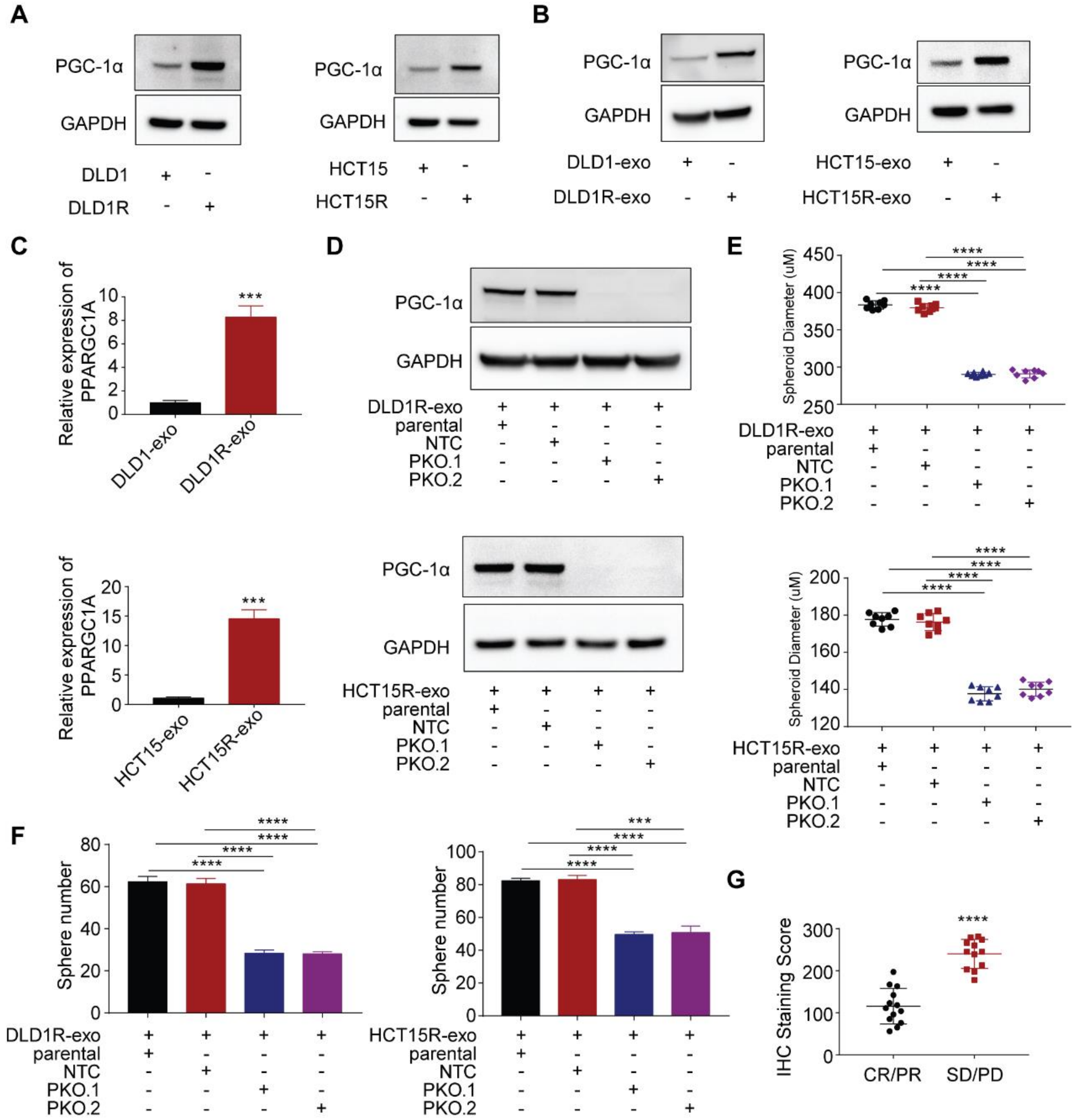

Figure S3. Res-exos induces PGC-1a expression

(A) Western blot showing protein levels of PGC-1α in indicated cells; (B-C) Assessment of PGC-1a expression in recipient cells after treatment with either Sen-exos and Res-exos by western blot (B) and qRT-PCR (C); (D) Western blot showing protein levels of PGC-1α in indicated cells; (E) Quantification of the spheroids diameter; (F) Quantification of the sphere number; (G) Quantification of the IHC staining score of PGC-1a in patient samples. ***$p < 0.001$; ****$p < 0.0001$. Numerical data represent mean± S.D. based on three independent experiments, and the immunoblots are representative of three replicates.

A

miRNA	Position in the UTR	seed match	context++ score	context++ score percentile	weighted context++ score	conserved branch length	Pct
hsa-miR-30e-5p	178-184	7mer -1A	-0.15	76	-0.15	4.875	0.78
hsa-miR-214-3p	194-200	7mer -1A	-0.04	61	-0.04	1.116	N/A

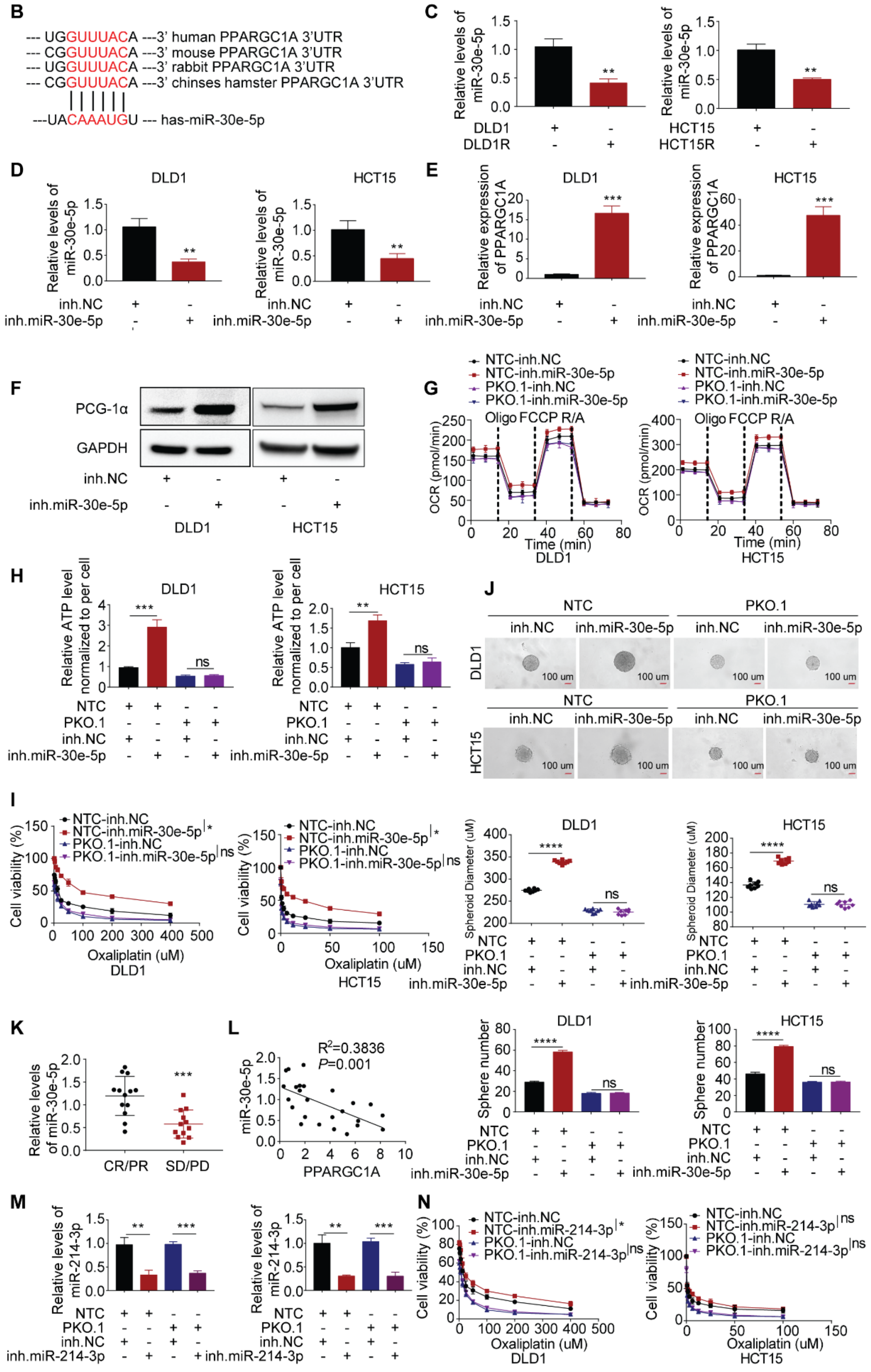

Figure S4. Identification of miR-30e-5p as a regulator of PGC-1a after Res-exos transfer.

(A) Detailed scores of two selected miRNAs by bioinformatic analysis; (B) Predicted conserved binding sites of miR-30e-5p within the 3'UTR of PPARGC1A mRNA across different species; (C) Assessment of miR-30e-5p expression by qRT-PCR analysis in sensitive and resistant cells; (D) Verification of the efficiency of Inhibitors of miR-30e-5p (Inhibitors.miR-30e-5p) in DLD1 and HCT15 cells; (E-F) Assessment of PGC-1a expression in recipient cells after transiently transfected with negative controls (inhibitors.NC) or miR-30e-5p (Inhibitors.miR-30e-5p) by qRT-PCR (E) and western blot (F) analyses; (G-J) Assessment of OCR (G), cellular ATP levels (H), cell viability (I) and sphere-formation (J) in control and PKO cells transiently transfected with inhibitors.miR-30e-5p or inhibitors.NC; (K) Measurement of miR-30e-5p expression levels in CRC tumor tissues by qRT-PCR assay; (L) Analysis of the correlation between miR-30e-5p and PPARGC1A expression in 25 CRC clinical samples; (M) Verification of the efficiency of Inhibitors of miR-214-3p (Inhibitors.miR-214-3p) in DLD1 and HCT15 cells; (N) Cell viability in control and PKO cells transiently transfected with inhibitors.miR-214-3p or inhibitors.NC **p < 0.01; ***p < 0.001; ****p < 0.0001. Numerical data represent mean± S.D. based on three independent experiments, and the immunoblots are representative of three replicates.

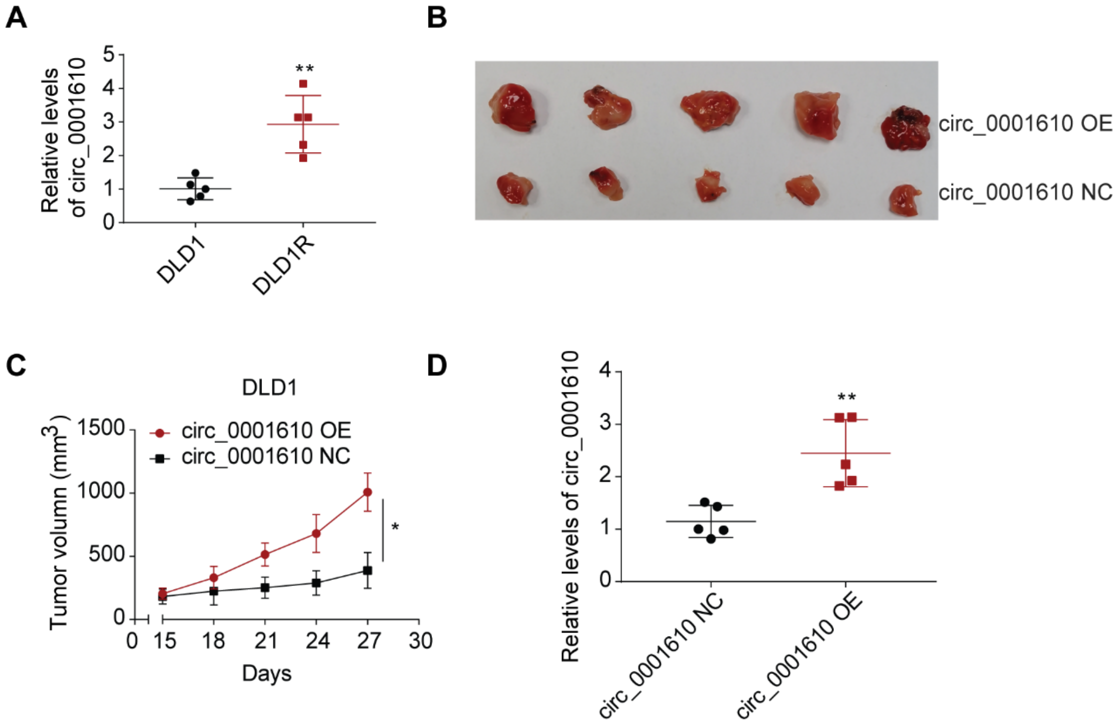

Figure S5. Exosomal circ_0001610 is related to oxaliplatin-resistance in vivo.

(A) Detection of the expression levels of circ_0001610 in serum exosomes by qRT-PCR. (B) Representative images of tumors in nude mice bearing DLD1 cells in different groups (n = 5 mice/group). (C) Quantitative analysis of xenografted tumor volume. (D) Measurement of the expression levels of circ_0001610 in tumor tissues from mice by qRT-PCR; *p < 0.05; **p < 0.01.

Table S1: Information of the human cohorts.

Clinical information of oxaliplatin-sensitive CRC patients

Patients	Gender	Age(years)	TNM stage	Cancer subtype	Chemotherapy response
1	Female	57	1	sigmoid colon	CR
2	Female	39	1	right hemicolon	CR
3	Male	33	1	left hemicolon	PR
4	Male	55	1	right hemicolon	PR
5	Male	45	4	ascending colon	PR
6	Male	72	2	descending colon	PR
7	Male	59	2	right hemicolon	CR
8	Female	54	2	ileocecum	CR
9	Male	56	2	left hemicolon	PR
10	Male	53	4	right hemicolon	PR
11	Female	55	2	left hemicolon	CR
12	Female	60	3	colon	PR
13	Male	55	3	colon	PR
Clinical information of oxaliplatin-resistant CRC patients					
Patients	Gender	Age(years)	TNM stage	Cancer subtype	Chemotherapy response
1	Female	66	3	right hemicolon	SD
2	Female	57	4	colon	SD
3	Female	79	4	transverse colon	SD
4	Female	65	3	sigmoid colon	SD
5	Male	49	1	right hemicolon	PD
6	Male	44	4	right hemicolon	PD
7	Male	58	4	sigmoid colon	PD
8	Male	57	3	left hemicolon	PD
9	Female	64	3	ileocecum	SD
10	Male	47	4	transverse colon	SD
11	Female	65	4	ileocecum	PD
12	Male	54	2	transverse colon	PD
Clinical chemotherapy response: Complete Response (CR); Partial Response (PR); Progressive Disease (PD); Stable Disease (SD).					

Table S2: Prediction of circRNAs which can directly adsorb miR-30e-5p by bioinformatic analysis

name	mirAccession	geneName	targetSites	bioComplex	clipReadNum
hsa-miR-30e-5p	MIMAT0000692	hsa-circRNA14081	2	6	552
hsa-miR-30e-5p	MIMAT0000692	hsa-circRNA5936	1	6	64
hsa-miR-30e-5p	MIMAT0000692	hsa-circRNA11224	1	7	1196
hsa-miR-30e-5p	MIMAT0000692	hsa-circRNA11421	1	3	38
hsa-miR-30e-5p	MIMAT0000692	hsa-circRNA14902	1	3	68
hsa-miR-30e-5p	MIMAT0000692	hsa-circRNA16289	1	3	58
hsa-miR-30e-5p	MIMAT0000692	hsa-circRNA1855	1	7	79
hsa-miR-30e-5p	MIMAT0000692	hsa-circRNA11602	1	6	78

hsa-miR-30e-5p	MIMAT0000692	hsa-circRNA11458	2	3	750
hsa-miR-30e-5p	MIMAT0000692	hsa-circRNA11423	1	6	1186
hsa-miR-30e-5p	MIMAT0000692	hsa-circRNA596	1	4	70
hsa-miR-30e-5p	MIMAT0000692	hsa-circRNA10855	1	4	71
hsa-miR-30e-5p	MIMAT0000692	hsa-circRNA5886	2	3	24
hsa-miR-30e-5p	MIMAT0000692	hsa-circRNA746	2	4	51
hsa-miR-30e-5p	MIMAT0000692	hsa-circRNA6874	4	8	940
hsa-miR-30e-5p	MIMAT0000692	hsa-circRNA1047	1	4	28
hsa-miR-30e-5p	MIMAT0000692	hsa-circRNA1232	1	4	48
hsa-miR-30e-5p	MIMAT0000692	hsa-circRNA10115	1	4	51
hsa-miR-30e-5p	MIMAT0000692	hsa-circRNA12474	2	3	108
hsa-miR-30e-5p	MIMAT0000692	hsa-circRNA11780	2	3	197
hsa-miR-30e-5p	MIMAT0000692	hsa-circRNA5272	1	3	5
hsa-miR-30e-5p	MIMAT0000692	hsa-circRNA6441	1	6	661
hsa-miR-30e-5p	MIMAT0000692	hsa-circRNA4580	1	3	30
hsa-miR-30e-5p	MIMAT0000692	hsa-circRNA13791	1	13	1133
hsa-miR-30e-5p	MIMAT0000692	hsa-circRNA15778	1	3	33
hsa-miR-30e-5p	MIMAT0000692	hsa-circRNA15229	1	8	199
hsa-miR-30e-5p	MIMAT0000692	hsa-circRNA12561	1	17	1823
hsa-miR-30e-5p	MIMAT0000692	hsa-circRNA7358	1	6	421
hsa-miR-30e-5p	MIMAT0000692	hsa-circRNA7984	2	3	151
hsa-miR-30e-5p	MIMAT0000692	hsa-circRNA9308	1	4	60
hsa-miR-30e-5p	MIMAT0000692	hsa-circRNA10433	4	11	1164
hsa-miR-30e-5p	MIMAT0000692	hsa-circRNA14816	2	4	0
hsa-miR-30e-5p	MIMAT0000692	hsa-circRNA10294	2	3	28
hsa-miR-30e-5p	MIMAT0000692	hsa-circRNA11379	1	5	58
hsa-miR-30e-5p	MIMAT0000692	hsa-circRNA8498	1	3	46
hsa-miR-30e-5p	MIMAT0000692	hsa-circRNA1482	1	6	71
hsa-miR-30e-5p	MIMAT0000692	hsa-circRNA2281	1	3	2
hsa-miR-30e-5p	MIMAT0000692	hsa-circRNA10310	1	3	36
hsa-miR-30e-5p	MIMAT0000692	hsa-circRNA8981	1	3	30
hsa-miR-30e-5p	MIMAT0000692	hsa-circRNA1108	1	5	66
hsa-miR-30e-5p	MIMAT0000692	hsa-circRNA15564	1	15	512
hsa-miR-30e-5p	MIMAT0000692	hsa-circRNA952	1	10	75
hsa-miR-30e-5p	MIMAT0000692	hsa-circRNA15985	1	4	34
hsa-miR-30e-5p	MIMAT0000692	hsa-circRNA9265	1	4	39
hsa-miR-30e-5p	MIMAT0000692	hsa-circRNA15162	2	5	153
hsa-miR-30e-5p	MIMAT0000692	hsa-circRNA6999	1	4	222
hsa-miR-30e-5p	MIMAT0000692	hsa-circRNA837	1	10	282
hsa-miR-30e-5p	MIMAT0000692	hsa-circRNA599	1	4	83
hsa-miR-30e-5p	MIMAT0000692	hsa-circRNA5377	1	3	49
hsa-miR-30e-5p	MIMAT0000692	hsa-circRNA11050	1	4	69
hsa-miR-30e-5p	MIMAT0000692	hsa-circRNA6780	1	3	68

hsa-miR-30e-5p	MIMAT0000692	hsa-circRNA851	2	3	19
hsa-miR-30e-5p	MIMAT0000692	hsa-circRNA7721	2	3	287
hsa-miR-30e-5p	MIMAT0000692	hsa-circRNA5300	1	7	83
hsa-miR-30e-5p	MIMAT0000692	hsa-circRNA4216	1	11	396
hsa-miR-30e-5p	MIMAT0000692	hsa-circRNA9624	1	8	86
hsa-miR-30e-5p	MIMAT0000692	hsa-circRNA15446	1	8	83
hsa-miR-30e-5p	MIMAT0000692	hsa-circRNA1186	1	9	3284
hsa-miR-30e-5p	MIMAT0000692	hsa-circRNA8553	1	4	234
hsa-miR-30e-5p	MIMAT0000692	hsa-circRNA15623	2	15	1175
hsa-miR-30e-5p	MIMAT0000692	hsa-circRNA1046	2	14	692
hsa-miR-30e-5p	MIMAT0000692	hsa-circRNA6086	2	4	302
hsa-miR-30e-5p	MIMAT0000692	hsa-circRNA6244	2	5	298
hsa-miR-30e-5p	MIMAT0000692	hsa-circRNA10657	1	7	244
hsa-miR-30e-5p	MIMAT0000692	hsa-circRNA15348	2	9	365
hsa-miR-30e-5p	MIMAT0000692	hsa-circRNA4592	2	22	3749
hsa-miR-30e-5p	MIMAT0000692	hsa-circRNA5547	1	4	34
hsa-miR-30e-5p	MIMAT0000692	hsa-circRNA9983	1	3	2
hsa-miR-30e-5p	MIMAT0000692	hsa-circRNA4326	1	12	1454
hsa-miR-30e-5p	MIMAT0000692	hsa-circRNA9093	1	4	0
hsa-miR-30e-5p	MIMAT0000692	hsa-circRNA15720	1	3	37
hsa-miR-30e-5p	MIMAT0000692	hsa-circRNA1280	1	3	9
hsa-miR-30e-5p	MIMAT0000692	hsa-circRNA12952	2	6	229
hsa-miR-30e-5p	MIMAT0000692	hsa-circRNA10122	2	3	26
hsa-miR-30e-5p	MIMAT0000692	hsa-circRNA5451	1	4	68
hsa-miR-30e-5p	MIMAT0000692	hsa-circRNA11165	1	3	13
hsa-miR-30e-5p	MIMAT0000692	hsa-circRNA11604	1	11	38
hsa-miR-30e-5p	MIMAT0000692	hsa-circRNA9010	1	4	0
hsa-miR-30e-5p	MIMAT0000692	hsa-circRNA6150	1	5	107
hsa-miR-30e-5p	MIMAT0000692	hsa-circRNA12646	1	4	121
hsa-miR-30e-5p	MIMAT0000692	hsa-circRNA14365	1	5	60
hsa-miR-30e-5p	MIMAT0000692	hsa-circRNA13831	1	7	1125
hsa-miR-30e-5p	MIMAT0000692	hsa-circRNA6913	1	3	48
hsa-miR-30e-5p	MIMAT0000692	hsa-circRNA9920	1	6	239
hsa-miR-30e-5p	MIMAT0000692	hsa-circRNA4317	1	4	24
hsa-miR-30e-5p	MIMAT0000692	hsa-circRNA2335	1	3	30
hsa-miR-30e-5p	MIMAT0000692	hsa-circRNA14498	1	6	142
hsa-miR-30e-5p	MIMAT0000692	hsa-circRNA5140	1	16	271
hsa-miR-30e-5p	MIMAT0000692	hsa-circRNA9272	1	3	14
hsa-miR-30e-5p	MIMAT0000692	hsa-circRNA14337	2	3	37
hsa-miR-30e-5p	MIMAT0000692	hsa-circRNA6238	1	3	128
hsa-miR-30e-5p	MIMAT0000692	hsa-circRNA5706	1	7	572
hsa-miR-30e-5p	MIMAT0000692	hsa-circRNA9371	1	3	88
hsa-miR-30e-5p	MIMAT0000692	hsa-circRNA9764	1	4	0

hsa-miR-30e-5p	MIMAT0000692	hsa-circRNA10444	1	9	171
hsa-miR-30e-5p	MIMAT0000692	hsa-circRNA12764	1	6	299
hsa-miR-30e-5p	MIMAT0000692	hsa-circRNA15355	1	7	123
hsa-miR-30e-5p	MIMAT0000692	hsa-circRNA14604	1	5	118
hsa-miR-30e-5p	MIMAT0000692	hsa-circRNA12169	1	3	46
hsa-miR-30e-5p	MIMAT0000692	hsa-circRNA15483	1	3	107
hsa-miR-30e-5p	MIMAT0000692	hsa-circRNA12804	1	3	119
hsa-miR-30e-5p	MIMAT0000692	hsa-circRNA6177	3	5	401
hsa-miR-30e-5p	MIMAT0000692	hsa-circRNA4580	1	6	0
hsa-miR-30e-5p	MIMAT0000692	hsa-circRNA1847	1	7	2997
hsa-miR-30e-5p	MIMAT0000692	hsa-circRNA12063	1	12	398
hsa-miR-30e-5p	MIMAT0000692	hsa-circRNA8475	1	4	473
hsa-miR-30e-5p	MIMAT0000692	hsa-circRNA11766	1	5	40
hsa-miR-30e-5p	MIMAT0000692	hsa-circRNA231	1	6	249
hsa-miR-30e-5p	MIMAT0000692	hsa-circRNA13041	1	6	211
hsa-miR-30e-5p	MIMAT0000692	hsa-circRNA442	1	5	75
hsa-miR-30e-5p	MIMAT0000692	hsa-circRNA10182	1	4	32
hsa-miR-30e-5p	MIMAT0000692	hsa-circRNA5415	3	12	490
hsa-miR-30e-5p	MIMAT0000692	hsa-circRNA673	1	3	26
hsa-miR-30e-5p	MIMAT0000692	hsa-circRNA414	2	5	1
hsa-miR-30e-5p	MIMAT0000692	hsa-circRNA1157	1	3	3
hsa-miR-30e-5p	MIMAT0000692	hsa-circRNA7710	1	4	109
hsa-miR-30e-5p	MIMAT0000692	hsa-circRNA1032	2	3	2
hsa-miR-30e-5p	MIMAT0000692	hsa-circRNA6680	1	5	84
hsa-miR-30e-5p	MIMAT0000692	hsa-circRNA1980	1	12	236
hsa-miR-30e-5p	MIMAT0000692	hsa-circRNA1616	1	3	107
hsa-miR-30e-5p	MIMAT0000692	hsa-circRNA13016	1	3	161
hsa-miR-30e-5p	MIMAT0000692	hsa-circRNA12759	1	3	62
hsa-miR-30e-5p	MIMAT0000692	hsa-circRNA15552	3	3	72
hsa-miR-30e-5p	MIMAT0000692	hsa-circRNA13048	2	6	273
hsa-miR-30e-5p	MIMAT0000692	hsa-circRNA4357	1	10	395
hsa-miR-30e-5p	MIMAT0000692	hsa-circRNA14404	2	9	1112
hsa-miR-30e-5p	MIMAT0000692	hsa-circRNA7394	1	5	297
hsa-miR-30e-5p	MIMAT0000692	hsa-circRNA10571	3	5	297
hsa-miR-30e-5p	MIMAT0000692	hsa-circRNA3099	1	20	1933
hsa-miR-30e-5p	MIMAT0000692	hsa-circRNA15202	1	3	762
hsa-miR-30e-5p	MIMAT0000692	hsa-circRNA4725	1	4	106
hsa-miR-30e-5p	MIMAT0000692	hsa-circRNA2727	1	3	292
hsa-miR-30e-5p	MIMAT0000692	hsa-circRNA13450	1	3	66
hsa-miR-30e-5p	MIMAT0000692	hsa-circRNA11262	2	4	10
hsa-miR-30e-5p	MIMAT0000692	hsa-circRNA7291	1	3	60
hsa-miR-30e-5p	MIMAT0000692	hsa-circRNA4493	1	4	44
hsa-miR-30e-5p	MIMAT0000692	hsa-circRNA11819	1	4	0

hsa-miR-30e-5p	MIMAT0000692	hsa-circRNA10052	1	4	229
hsa-miR-30e-5p	MIMAT0000692	hsa-circRNA14213	3	7	155
hsa-miR-30e-5p	MIMAT0000692	hsa-circRNA1848	1	3	1
hsa-miR-30e-5p	MIMAT0000692	hsa-circRNA617	2	3	10
hsa-miR-30e-5p	MIMAT0000692	hsa-circRNA10842	1	12	326
hsa-miR-30e-5p	MIMAT0000692	hsa-circRNA7773	1	3	39
hsa-miR-30e-5p	MIMAT0000692	hsa-circRNA5824	1	5	171
hsa-miR-30e-5p	MIMAT0000692	hsa-circRNA5667	2	22	2054
hsa-miR-30e-5p	MIMAT0000692	hsa-circRNA6572	1	6	528
hsa-miR-30e-5p	MIMAT0000692	hsa-circRNA1071	2	6	24
hsa-miR-30e-5p	MIMAT0000692	hsa-circRNA1030	1	13	612
hsa-miR-30e-5p	MIMAT0000692	hsa-circRNA1312	1	4	1
hsa-miR-30e-5p	MIMAT0000692	hsa-circRNA12489	1	8	550
hsa-miR-30e-5p	MIMAT0000692	hsa-circRNA9168	2	5	658
hsa-miR-30e-5p	MIMAT0000692	hsa-circRNA16075	1	3	98
hsa-miR-30e-5p	MIMAT0000692	hsa-circRNA5059	1	8	1701
hsa-miR-30e-5p	MIMAT0000692	hsa-circRNA9897	3	12	1155
hsa-miR-30e-5p	MIMAT0000692	hsa-circRNA897	1	7	77
hsa-miR-30e-5p	MIMAT0000692	hsa-circRNA13785	1	3	175
hsa-miR-30e-5p	MIMAT0000692	hsa-circRNA7851	1	4	271
hsa-miR-30e-5p	MIMAT0000692	hsa-circRNA7213	1	7	117
hsa-miR-30e-5p	MIMAT0000692	hsa-circRNA15731	1	10	647
hsa-miR-30e-5p	MIMAT0000692	hsa-circRNA14354	1	3	22
hsa-miR-30e-5p	MIMAT0000692	hsa-circRNA7044	1	4	6148
hsa-miR-30e-5p	MIMAT0000692	hsa-circRNA4134	1	7	39
hsa-miR-30e-5p	MIMAT0000692	hsa-circRNA4384	1	5	93
hsa-miR-30e-5p	MIMAT0000692	hsa-circRNA8544	3	8	135
hsa-miR-30e-5p	MIMAT0000692	hsa-circRNA14784	1	5	852
hsa-miR-30e-5p	MIMAT0000692	hsa-circRNA13283	1	5	1464
hsa-miR-30e-5p	MIMAT0000692	hsa-circRNA10424	1	5	14
hsa-miR-30e-5p	MIMAT0000692	hsa-circRNA302	1	8	89
hsa-miR-30e-5p	MIMAT0000692	hsa-circRNA2172	2	4	126
hsa-miR-30e-5p	MIMAT0000692	hsa-circRNA10577	1	7	51
hsa-miR-30e-5p	MIMAT0000692	hsa-circRNA7853	1	7	514
hsa-miR-30e-5p	MIMAT0000692	hsa-circRNA10624	1	3	39
hsa-miR-30e-5p	MIMAT0000692	hsa-circRNA13743	1	6	105
hsa-miR-30e-5p	MIMAT0000692	hsa-circRNA4386	1	11	498
hsa-miR-30e-5p	MIMAT0000692	hsa-circRNA738	2	5	87
hsa-miR-30e-5p	MIMAT0000692	hsa-circRNA11920	1	8	859
hsa-miR-30e-5p	MIMAT0000692	hsa-circRNA14866	2	9	726
hsa-miR-30e-5p	MIMAT0000692	hsa-circRNA4442	1	4	29
hsa-miR-30e-5p	MIMAT0000692	hsa-circRNA15246	1	12	138
hsa-miR-30e-5p	MIMAT0000692	hsa-circRNA6178	1	4	61

hsa-miR-30e-5p	MIMAT0000692	hsa-circRNA455	1	19	2301
hsa-miR-30e-5p	MIMAT0000692	hsa-circRNA8632	1	3	8
hsa-miR-30e-5p	MIMAT0000692	hsa-circRNA8371	1	4	97
hsa-miR-30e-5p	MIMAT0000692	hsa-circRNA16263	2	4	68
hsa-miR-30e-5p	MIMAT0000692	hsa-circRNA6594	1	5	22
hsa-miR-30e-5p	MIMAT0000692	hsa-circRNA2915	1	4	410
hsa-miR-30e-5p	MIMAT0000692	hsa-circRNA13798	2	4	92
hsa-miR-30e-5p	MIMAT0000692	hsa-circRNA9098	1	4	444
hsa-miR-30e-5p	MIMAT0000692	hsa-circRNA5145	2	3	494
hsa-miR-30e-5p	MIMAT0000692	hsa-circRNA5778	2	8	2457
hsa-miR-30e-5p	MIMAT0000692	hsa-circRNA9103	2	4	28
hsa-miR-30e-5p	MIMAT0000692	hsa-circRNA5840	1	5	87
hsa-miR-30e-5p	MIMAT0000692	hsa-circRNA8962	1	6	705
hsa-miR-30e-5p	MIMAT0000692	hsa-circRNA8255	2	4	2187
hsa-miR-30e-5p	MIMAT0000692	hsa-circRNA10490	1	5	157
hsa-miR-30e-5p	MIMAT0000692	hsa-circRNA13882	1	4	94
hsa-miR-30e-5p	MIMAT0000692	hsa-circRNA13347	1	13	395
hsa-miR-30e-5p	MIMAT0000692	hsa-circRNA2682	1	5	306
hsa-miR-30e-5p	MIMAT0000692	hsa-circRNA4895	1	3	550
hsa-miR-30e-5p	MIMAT0000692	hsa-circRNA4434	1	3	230
hsa-miR-30e-5p	MIMAT0000692	hsa-circRNA6001	1	3	99
hsa-miR-30e-5p	MIMAT0000692	hsa-circRNA7993	1	9	73
hsa-miR-30e-5p	MIMAT0000692	hsa-circRNA8331	1	3	43
hsa-miR-30e-5p	MIMAT0000692	hsa-circRNA5854	1	6	54
hsa-miR-30e-5p	MIMAT0000692	hsa-circRNA5730	1	4	1513
hsa-miR-30e-5p	MIMAT0000692	hsa-circRNA595	1	3	12
hsa-miR-30e-5p	MIMAT0000692	hsa-circRNA1485	2	3	10
hsa-miR-30e-5p	MIMAT0000692	hsa-circRNA6370	1	3	89
hsa-miR-30e-5p	MIMAT0000692	hsa-circRNA10155	1	4	8
hsa-miR-30e-5p	MIMAT0000692	hsa-circRNA6877	1	3	59
hsa-miR-30e-5p	MIMAT0000692	hsa-circRNA4578	1	5	22
hsa-miR-30e-5p	MIMAT0000692	hsa-circRNA9236	1	3	229
hsa-miR-30e-5p	MIMAT0000692	hsa-circRNA3951	1	7	130
hsa-miR-30e-5p	MIMAT0000692	hsa-circRNA7194	1	3	2
hsa-miR-30e-5p	MIMAT0000692	hsa-circRNA11733	1	4	0
hsa-miR-30e-5p	MIMAT0000692	hsa-circRNA2565	1	11	2370
hsa-miR-30e-5p	MIMAT0000692	hsa-circRNA6550	1	8	341
hsa-miR-30e-5p	MIMAT0000692	hsa-circRNA329	1	4	128
hsa-miR-30e-5p	MIMAT0000692	hsa-circRNA7333	1	13	173
hsa-miR-30e-5p	MIMAT0000692	hsa-circRNA14086	1	3	27
hsa-miR-30e-5p	MIMAT0000692	hsa-circRNA16202	1	5	111
hsa-miR-30e-5p	MIMAT0000692	hsa-circRNA8581	1	14	997
hsa-miR-30e-5p	MIMAT0000692	hsa-circRNA4531	1	6	236

hsa-miR-30e-5p	MIMAT0000692	hsa-circRNA8401	1	5	43
hsa-miR-30e-5p	MIMAT0000692	hsa-circRNA7460	2	5	1876
hsa-miR-30e-5p	MIMAT0000692	hsa-circRNA7562	1	3	12
hsa-miR-30e-5p	MIMAT0000692	hsa-circRNA12854	2	3	57
hsa-miR-30e-5p	MIMAT0000692	hsa-circRNA5343	1	3	31
hsa-miR-30e-5p	MIMAT0000692	hsa-circRNA13803	1	3	24
hsa-miR-30e-5p	MIMAT0000692	hsa-circRNA703	1	5	127
hsa-miR-30e-5p	MIMAT0000692	hsa-circRNA14415	1	5	232
hsa-miR-30e-5p	MIMAT0000692	hsa-circRNA296	1	4	19
hsa-miR-30e-5p	MIMAT0000692	hsa-circRNA15980	1	13	868
hsa-miR-30e-5p	MIMAT0000692	hsa-circRNA14803	1	4	69
hsa-miR-30e-5p	MIMAT0000692	hsa-circRNA4359	1	3	90
hsa-miR-30e-5p	MIMAT0000692	hsa-circRNA6424	1	5	49
hsa-miR-30e-5p	MIMAT0000692	hsa-circRNA12099	1	5	79
hsa-miR-30e-5p	MIMAT0000692	hsa-circRNA12521	2	13	671
hsa-miR-30e-5p	MIMAT0000692	hsa-circRNA11596	1	3	12
hsa-miR-30e-5p	MIMAT0000692	hsa-circRNA12472	1	19	736
hsa-miR-30e-5p	MIMAT0000692	hsa-circRNA12918	1	3	130
hsa-miR-30e-5p	MIMAT0000692	hsa-circRNA1610	1	3	921
hsa-miR-30e-5p	MIMAT0000692	hsa-circRNA4974	1	4	68
hsa-miR-30e-5p	MIMAT0000692	hsa-circRNA326	2	4	80
hsa-miR-30e-5p	MIMAT0000692	hsa-circRNA3624	1	6	77
hsa-miR-30e-5p	MIMAT0000692	hsa-circRNA2987	1	3	73
hsa-miR-30e-5p	MIMAT0000692	hsa-circRNA14048	1	4	30
hsa-miR-30e-5p	MIMAT0000692	hsa-circRNA9853	1	3	20
hsa-miR-30e-5p	MIMAT0000692	hsa-circRNA16241	2	10	94
hsa-miR-30e-5p	MIMAT0000692	hsa-circRNA2505	1	4	131
hsa-miR-30e-5p	MIMAT0000692	hsa-circRNA6822	1	4	250
hsa-miR-30e-5p	MIMAT0000692	hsa-circRNA14997	1	29	4180
hsa-miR-30e-5p	MIMAT0000692	hsa-circRNA10041	1	9	910
hsa-miR-30e-5p	MIMAT0000692	hsa-circRNA15986	1	8	190
hsa-miR-30e-5p	MIMAT0000692	hsa-circRNA4319	1	4	28
hsa-miR-30e-5p	MIMAT0000692	hsa-circRNA14836	1	9	53
hsa-miR-30e-5p	MIMAT0000692	hsa-circRNA11817	1	4	126
hsa-miR-30e-5p	MIMAT0000692	hsa-circRNA2576	1	3	40
hsa-miR-30e-5p	MIMAT0000692	hsa-circRNA14411	2	10	745
hsa-miR-30e-5p	MIMAT0000692	hsa-circRNA14967	1	5	6
hsa-miR-30e-5p	MIMAT0000692	hsa-circRNA6410	1	10	67
hsa-miR-30e-5p	MIMAT0000692	hsa-circRNA36	1	3	1
hsa-miR-30e-5p	MIMAT0000692	hsa-circRNA12883	2	5	1996
hsa-miR-30e-5p	MIMAT0000692	hsa-circRNA11382	2	10	2608
hsa-miR-30e-5p	MIMAT0000692	hsa-circRNA10499	1	3	489
hsa-miR-30e-5p	MIMAT0000692	hsa-circRNA11130	1	3	9

hsa-miR-30e-5p	MIMAT0000692	hsa-circRNA277	1	5	23
hsa-miR-30e-5p	MIMAT0000692	hsa-circRNA11577	1	3	40
hsa-miR-30e-5p	MIMAT0000692	hsa-circRNA13378	1	9	176
hsa-miR-30e-5p	MIMAT0000692	hsa-circRNA14381	1	3	89
hsa-miR-30e-5p	MIMAT0000692	hsa-circRNA8391	1	23	2406
hsa-miR-30e-5p	MIMAT0000692	hsa-circRNA815	1	15	1121
hsa-miR-30e-5p	MIMAT0000692	hsa-circRNA3463	3	8	507
hsa-miR-30e-5p	MIMAT0000692	hsa-circRNA219	1	3	36
hsa-miR-30e-5p	MIMAT0000692	hsa-circRNA7424	2	4	15
hsa-miR-30e-5p	MIMAT0000692	hsa-circRNA6281	2	8	106
hsa-miR-30e-5p	MIMAT0000692	hsa-circRNA7283	2	9	265
hsa-miR-30e-5p	MIMAT0000692	hsa-circRNA435	1	5	90
hsa-miR-30e-5p	MIMAT0000692	hsa-circRNA14203	2	6	63
hsa-miR-30e-5p	MIMAT0000692	hsa-circRNA6576	2	5	464

Table S3: DEGs of RNA Sequencing data (DLD1)

gene_id	gene_name	OXAR_mean	NTC_mean	log2FoldChange	pvalue	padj
ENSG00000114251	WNT5A	648.2353578	59.66865038	3.439132829	1.90E-77	3.11E-73
ENSG00000169174	PCSK9	39.47975826	522.3296295	-3.72650958	4.96E-69	4.06E-65
ENSG00000044115	CTNNA1	3306.664069	10536.69368	-1.672080416	3.54E-65	1.93E-61
ENSG00000149150	SLC43A1	47.52784956	460.1699876	-3.276762869	1.42E-47	5.82E-44
ENSG00000067141	NEO1	1527.147273	456.8941576	1.740150166	1.38E-45	4.51E-42
ENSG00000172824	CES4A	20.72329178	269.5439458	-3.698242186	8.97E-37	2.44E-33
ENSG00000130707	ASS1	870.5842796	4075.938286	-2.226900667	1.34E-34	3.13E-31
ENSG00000166347	CYB5A	504.4775526	1356.20996	-1.426863314	2.58E-34	5.27E-31
ENSG00000130600	H19	73.64312867	412.6352797	-2.482831364	1.25E-33	2.27E-30
ENSG00000147862	NFIB	791.4846013	2016.570454	-1.348754144	2.46E-32	4.02E-29
ENSG00000215182	MUC5AC	1957.007586	6938.421811	-1.825858118	4.24E-32	6.30E-29
ENSG00000197043	ANXA6	271.850409	789.3776059	-1.53720341	1.31E-28	1.79E-25
ENSG00000189369	GSPT2	70.90775579	345.1419291	-2.284031749	1.89E-28	2.38E-25
ENSG00000089127	OAS1	294.2230739	1018.327574	-1.792159732	3.11E-28	3.63E-25
ENSG00000172379	ARNT2	166.3780269	585.7026355	-1.817800611	4.76E-28	5.19E-25
ENSG00000146192	FGD2	104.7303633	431.0112034	-2.041200505	6.30E-27	6.44E-24
ENSG00000104723	TUSC3	143.6354661	2.997501866	5.589764802	2.68E-26	2.58E-23
ENSG00000126561	STAT5A	17.103927	201.2271675	-3.554965261	1.00E-25	9.13E-23
ENSG00000009950	MLXIPL	201.2796876	796.2341534	-1.983124232	3.32E-25	2.86E-22
ENSG00000138028	CGREF1	17.60899766	161.4261096	-3.20141408	4.73E-25	3.87E-22
ENSG00000233041	PHGR1	182.358203	21.16363498	3.10816798	5.42E-25	4.22E-22
ENSG00000128340	RAC2	148.2853829	544.4191142	-1.878506298	5.94E-25	4.41E-22
ENSG00000068078	FGFR3	107.204856	525.2276223	-2.292813796	8.72E-25	6.20E-22
ENSG00000135549	PKIB	1287.589893	573.2080232	1.167134591	1.94E-24	1.32E-21
ENSG00000196743	GM2A	2650.201657	1321.165031	1.004137107	3.78E-23	2.47E-20
ENSG00000167363	FN3K	62.29291747	320.2393388	-2.361142324	8.65E-23	5.44E-20

ENSG00000132205	EMILIN2	65.75042306	286.365461	-2.125624853	1.22E-22	7.40E-20
ENSG00000115221	ITGB6	79.59686199	361.6486988	-2.185274795	2.39E-22	1.39E-19
ENSG00000169035	KLK7	8.958198015	170.4617734	-4.234501678	2.75E-22	1.55E-19
ENSG00000131127	ZNF141	5.914302429	126.147157	-4.428025383	3.32E-22	1.81E-19
ENSG00000189431	RASSF10	216.7695441	609.8885139	-1.492734887	3.74E-21	1.97E-18
ENSG00000182568	SATB1	180.3264833	1.201251827	7.241460065	5.14E-21	2.62E-18
ENSG00000074416	MGLL	270.8561805	773.8895846	-1.515765463	1.66E-20	8.25E-18
ENSG00000145703	IQGAP2	427.9473865	139.7231995	1.616893155	4.43E-20	2.13E-17
ENSG00000167755	KLK6	907.1847747	2045.143857	-1.173074297	4.80E-20	2.24E-17
ENSG00000105329	TGFB1	283.0399228	831.3264276	-1.553963271	1.06E-19	4.84E-17
ENSG00000112902	SEMA5A	1607.286078	589.4036162	1.445786442	1.29E-19	5.72E-17
ENSG00000112541	PDE10A	156.5617519	29.98911407	2.382421256	2.15E-19	9.24E-17
ENSG00000106070	GRB10	1180.221159	2718.368758	-1.203599152	2.52E-19	1.06E-16
ENSG00000128283	CDC42EP1	283.9058492	1066.454508	-1.90919894	2.64E-19	1.08E-16
ENSG00000197903	HIST1H2BK	985.4408809	259.9243355	1.924321144	3.45E-19	1.38E-16
ENSG00000138640	FAM13A	138.0434096	389.5248792	-1.497451968	4.77E-19	1.86E-16
ENSG00000137801	THBS1	8373.339625	3810.332721	1.135948154	6.53E-19	2.49E-16
ENSG00000175416	CLTB	2947.606641	1464.227622	1.009430319	8.45E-19	3.14E-16
ENSG00000105976	MET	6170.110799	3237.736868	0.930170486	1.34E-18	4.85E-16
ENSG00000117322	CR2	162.4466698	426.3966661	-1.392221234	2.39E-18	8.51E-16
ENSG00000204385	SLC44A4	22.09087827	210.8049006	-3.250566274	2.80E-18	9.73E-16
ENSG00000101974	ATP11C	85.5325888	3.348650193	4.699484384	3.08E-18	1.05E-15
ENSG00000185432	METTL7A	155.5446124	659.0591648	-2.083077154	5.57E-18	1.86E-15
ENSG00000149600	COMMD7	70.13915653	312.9479158	-2.156034344	8.34E-18	2.73E-15
ENSG00000043355	ZIC2	963.180037	1801.918563	-0.903463993	1.18E-17	3.79E-15
ENSG00000179222	MAGED1	1.461244139	142.68037	-6.612402519	1.78E-17	5.59E-15
ENSG00000213949	ITGA1	285.1475077	88.03063854	1.6948445	1.92E-17	5.92E-15
ENSG00000181577	C6orf223	72.14510211	366.8701293	-2.346508952	2.31E-17	6.99E-15
ENSG00000166123	GPT2	1368.595312	3118.275801	-1.188031249	2.36E-17	7.03E-15
ENSG00000026508	CD44	219.3721316	757.5335667	-1.788921049	4.16E-17	1.22E-14
ENSG00000169504	CLIC4	3545.207774	7855.7548	-1.147975739	4.84E-17	1.39E-14
ENSG00000214425	LRRC37A4P	1951.851295	3822.493558	-0.969607812	7.90E-17	2.23E-14
ENSG00000145632	PLK2	1425.314881	678.4090564	1.070151558	1.17E-16	3.24E-14
ENSG00000197119	SLC25A29	476.2079189	1010.010422	-1.083963205	1.31E-16	3.57E-14
ENSG00000134954	ETS1	155.5672719	31.32391743	2.306915483	1.39E-16	3.74E-14
ENSG00000213445	SIPA1	131.4195284	347.7377124	-1.404212175	1.50E-16	3.97E-14
ENSG00000134240	HMGCS2	16.33557931	128.6092003	-2.973426342	1.58E-16	4.10E-14
ENSG00000072071	ADGRL1	863.8920111	1717.269115	-0.990785247	1.63E-16	4.17E-14
ENSG00000157399	ARSE	15.8974893	121.7088782	-2.947370428	2.27E-16	5.71E-14
ENSG00000120457	KCNJ5	123.749234	392.5142551	-1.664868443	2.66E-16	6.59E-14
ENSG00000177732	SOX12	575.3408248	1124.361811	-0.966183401	3.32E-16	8.10E-14
ENSG00000112343	TRIM38	610.0145701	1280.130444	-1.069960765	3.37E-16	8.11E-14
ENSG00000187837	HIST1H1C	1109.083526	451.2284899	1.297907418	3.76E-16	8.90E-14

ENSG00000184160	ADRA2C	110.4967813	358.4399882	-1.6977346	4.37E-16	1.02E-13
ENSG00000134193	REG4	101.063097	11.88254912	3.067449109	6.28E-16	1.45E-13
ENSG00000086619	ERO1B	286.5869061	726.281691	-1.342152318	9.09E-16	2.07E-13
ENSG00000140961	OSGIN1	978.8035743	362.6990239	1.432504652	1.07E-15	2.40E-13
ENSG00000128917	DLL4	113.8221603	322.2460798	-1.499604372	1.50E-15	3.31E-13
ENSG00000178401	DNAJC22	537.2776788	1082.423402	-1.010747642	1.59E-15	3.47E-13
ENSG00000089472	HEPH	81.98836467	8.053712281	3.348366105	1.82E-15	3.92E-13
ENSG00000166394	CYB5R2	41.45637839	174.1637266	-2.068787019	1.90E-15	4.04E-13
ENSG00000137462	TLR2	44.83022106	173.9780915	-1.956106639	2.03E-15	4.25E-13
ENSG00000117480	FAAH	145.5258265	361.960357	-1.314371754	3.68E-15	7.55E-13
ENSG00000163817	SLC6A20	1412.734759	3222.469359	-1.189943142	3.69E-15	7.55E-13
ENSG00000189060	H1F0	2584.033527	5312.720147	-1.039911063	5.55E-15	1.12E-12
ENSG00000231711	LINC00899	697.0184427	1682.185948	-1.270697348	6.38E-15	1.27E-12
ENSG00000076716	GPC4	41.97180604	180.6630743	-2.111528216	6.85E-15	1.35E-12
ENSG00000158352	SHROOM4	68.82263047	4.989580269	3.77056858	7.88E-15	1.54E-12
ENSG00000180340	FZD2	136.4070419	347.6611802	-1.351017239	1.01E-14	1.95E-12
ENSG00000137261	KIAA0319	283.4599688	103.7459552	1.450834732	1.08E-14	2.05E-12
ENSG00000165626	BEND7	73.45535455	1.8996463	5.345766482	1.28E-14	2.38E-12
ENSG00000182199	SHMT2	3946.582193	6914.385549	-0.809065956	1.28E-14	2.38E-12
ENSG00000147852	VLDLR	144.384424	490.9006828	-1.766408259	1.63E-14	3.00E-12
ENSG00000155158	TTC39B	505.9556199	1081.720191	-1.096726835	2.21E-14	4.02E-12
ENSG00000159640	ACE	52.36528159	209.2582661	-1.997131564	2.24E-14	4.03E-12
ENSG00000115825	PRKD3	595.2687813	1157.529802	-0.959670481	2.43E-14	4.32E-12
ENSG00000236039	AC019117.1	103.2513642	341.6763108	-1.72727454	3.02E-14	5.27E-12
ENSG00000106780	MEGF9	1203.875313	653.5018062	0.881781716	3.05E-14	5.27E-12
ENSG00000167748	KLK1	298.0910164	870.5589442	-1.546256043	3.06E-14	5.27E-12
ENSG00000064270	ATP2C2	533.7145164	1331.092483	-1.318882422	3.12E-14	5.32E-12
ENSG00000276180	HIST1H4I	270.9571469	85.4249533	1.664696072	3.37E-14	5.68E-12
ENSG00000223764	LINC02593	47.71062263	199.5595902	-2.063293371	3.95E-14	6.59E-12
ENSG00000185630	PBX1	152.6166716	434.8313223	-1.512253382	4.27E-14	7.06E-12
ENSG00000102870	ZNF629	350.9235351	751.7357522	-1.099481198	4.39E-14	7.18E-12
ENSG00000100558	PLEK2	88.97045306	281.0440721	-1.660900258	5.58E-14	9.04E-12
ENSG00000221963	APOL6	623.7185181	1608.655696	-1.367184186	6.35E-14	1.02E-11
ENSG00000175471	MCTP1	177.0787009	53.40309253	1.730461037	7.41E-14	1.18E-11
ENSG00000144668	ITGA9	135.6323172	398.3703888	-1.552525298	9.27E-14	1.46E-11
ENSG00000112782	CLIC5	431.7157674	141.3659799	1.606147329	9.68E-14	1.51E-11
ENSG00000124635	HIST1H2BJ	116.0065582	22.84839315	2.349650533	9.79E-14	1.51E-11
ENSG00000143412	ANXA9	466.6817013	942.4832068	-1.014276967	9.93E-14	1.52E-11
ENSG00000048740	CELF2	87.56241469	0	9.052917389	1.24E-13	1.88E-11
ENSG00000188042	ARL4C	2078.270378	3537.122491	-0.767320193	1.32E-13	1.98E-11
ENSG00000142235	LMTK3	151.9593743	449.369337	-1.563771323	1.38E-13	2.05E-11
ENSG00000082512	TRAF5	145.5270991	404.34063	-1.474325896	1.49E-13	2.20E-11
ENSG00000092969	TGFB2	904.0925188	2244.393293	-1.312245668	1.92E-13	2.80E-11

ENSG00000283283	AC013268.4	20.48174973	115.0759505	-2.492975646	2.08E-13	3.01E-11
ENSG00000187634	SAMD11	47.02354096	185.818006	-1.982992518	2.37E-13	3.40E-11
ENSG00000135069	PSAT1	7341.702406	15712.1193	-1.097695815	3.43E-13	4.88E-11
ENSG00000116852	KIF21B	360.8572232	1039.008932	-1.525414188	3.60E-13	5.08E-11
ENSG00000204983	PRSS1	13.28877633	131.2516784	-3.311171371	4.42E-13	6.18E-11
ENSG00000153707	PTPRD	13.1657342	99.07173372	-2.916331236	4.47E-13	6.19E-11
ENSG00000106066	CPVL	117.6164488	297.2644294	-1.338683432	4.83E-13	6.64E-11
ENSG00000130766	SESN2	678.5154511	1517.744175	-1.160953369	5.10E-13	6.95E-11
ENSG00000003436	TFPI	27.35987514	124.4118	-2.185831505	5.79E-13	7.83E-11
ENSG00000135842	FAM129A	16.05700326	122.3323567	-2.930853584	6.13E-13	8.22E-11
ENSG00000145730	PAM	1192.229378	2242.309727	-0.911504644	6.84E-13	9.07E-11
ENSG00000109205	ODAM	58.6626852	3.666483447	4.026799665	6.88E-13	9.07E-11
ENSG00000092621	PHGDH	1941.775451	10311.70737	-2.408833165	7.92E-13	1.04E-10
ENSG00000100504	PYGL	207.6002095	461.9374661	-1.153383726	1.18E-12	1.53E-10
ENSG00000156171	DRAM2	654.6904708	1254.755399	-0.93898689	1.19E-12	1.53E-10
ENSG00000156587	UBE2L6	51.47950664	242.667585	-2.239063433	1.21E-12	1.55E-10
ENSG00000122378	PRXL2A	431.7230546	830.6172574	-0.94456558	1.32E-12	1.67E-10
ENSG00000168032	ENTPD3	202.2144367	592.6970154	-1.550528965	1.42E-12	1.78E-10
ENSG00000172986	GXYLT2	276.8971428	572.5905713	-1.047628679	1.57E-12	1.96E-10
ENSG00000118513	MYB	536.0699987	1316.4689	-1.29629453	1.64E-12	2.04E-10
ENSG00000165376	CLDN2	368.3673965	1044.058615	-1.503324923	1.75E-12	2.15E-10
ENSG00000112655	PTK7	2061.53122	3654.612271	-0.82623153	2.13E-12	2.60E-10
ENSG00000247095	MIR210HG	143.4113167	420.1911429	-1.551745426	2.23E-12	2.70E-10
ENSG00000154358	OBSCN	1066.112456	2150.571399	-1.011872635	2.32E-12	2.78E-10
ENSG00000146733	PSPH	1451.15478	2452.992268	-0.757469033	2.44E-12	2.92E-10
ENSG00000108602	ALDH3A1	6004.606452	3071.256017	0.967125067	2.71E-12	3.22E-10
ENSG00000088543	C3orf18	34.14143552	142.9437879	-2.063622	2.92E-12	3.44E-10
ENSG00000149212	SESN3	445.525986	200.2520895	1.151346922	3.00E-12	3.51E-10
ENSG00000124143	ARHGAP40	6.290516172	73.20744706	-3.554012668	3.56E-12	4.13E-10
ENSG00000141985	SH3GL1	2994.232965	1690.043226	0.825340798	3.74E-12	4.31E-10
ENSG00000157404	KIT	1390.357876	2741.154495	-0.979816554	4.10E-12	4.69E-10
ENSG00000113448	PDE4D	1176.383984	2003.491557	-0.76843013	4.59E-12	5.21E-10
ENSG00000230356	NCAPD2P1	5.147027398	67.76151765	-3.726367163	5.02E-12	5.66E-10
ENSG00000153029	MR1	135.4106164	450.3588758	-1.735474364	5.49E-12	6.15E-10
ENSG00000132436	FIGNL1	1235.336527	636.283803	0.956607972	8.42E-12	9.37E-10
ENSG00000053918	KCNQ1	377.0271631	1001.129843	-1.408649082	9.17E-12	1.01E-09
ENSG00000074219	TEAD2	62.25771821	198.3811137	-1.671360401	9.35E-12	1.02E-09
ENSG00000160183	TMPRSS3	211.7688179	658.3152293	-1.636894036	9.36E-12	1.02E-09
ENSG00000064195	DLX3	22.21575513	115.8371757	-2.38396616	9.51E-12	1.03E-09
ENSG00000154265	ABCA5	156.8866463	49.76676436	1.65324809	1.09E-11	1.17E-09
ENSG00000116675	DNAJC6	106.48401	23.47309549	2.178884302	1.13E-11	1.20E-09
ENSG00000028137	TNFRSF1B	35.77912746	157.0769718	-2.1354101	1.16E-11	1.22E-09
ENSG00000178814	OPLAH	105.3332715	339.5408931	-1.687991016	1.16E-11	1.22E-09

ENSG00000142515	KLK3	14.29361337	106.2470489	-2.896615106	1.16E-11	1.22E-09
ENSG00000124126	PREX1	19.60520744	97.30450252	-2.307629827	1.19E-11	1.24E-09
ENSG00000172985	SH3RF3	165.473321	464.8767514	-1.489485718	1.28E-11	1.33E-09
ENSG00000075223	SEMA3C	964.4534553	509.715051	0.920150932	1.38E-11	1.42E-09
ENSG00000109819	PPARGC1A	53.67466127	3.537125335	3.913133409	1.42E-11	1.46E-09
ENSG00000171124	FUT3	10.25013336	99.99356308	-3.29040289	1.71E-11	1.74E-09
ENSG00000154380	ENAH	1497.770371	2510.489773	-0.744936129	2.00E-11	2.01E-09
ENSG00000100968	NFATC4	50.72600649	240.6219192	-2.246476852	2.33E-11	2.34E-09
ENSG00000145819	ARHGAP26	515.9061151	962.4663247	-0.900577132	2.73E-11	2.73E-09
ENSG00000167291	TBC1D16	898.1443647	2017.097329	-1.167329768	3.19E-11	3.17E-09
ENSG00000183486	MX2	22.27237879	109.1768013	-2.296675591	4.14E-11	4.08E-09
ENSG00000123843	C4BPB	1836.251347	1080.48351	0.764943885	4.24E-11	4.15E-09
ENSG00000139793	MBNL2	1023.762963	2170.238885	-1.084403882	4.80E-11	4.67E-09
ENSG00000283167	AC140479.7	23.58226897	115.818727	-2.294894739	5.01E-11	4.85E-09
ENSG00000165521	EML5	230.4334098	63.60181431	1.859767807	5.29E-11	5.09E-09
ENSG00000251493	FOXD1	754.9941711	399.4047912	0.919219134	5.65E-11	5.41E-09
ENSG00000105538	RASIP1	107.83777	309.2105327	-1.518787675	5.76E-11	5.47E-09
ENSG00000183722	LHFPL6	132.120385	38.11277619	1.801931765	6.02E-11	5.69E-09
ENSG00000158321	AUTS2	107.3959515	23.00055331	2.223897278	6.31E-11	5.93E-09
ENSG00000114805	PLCH1	31.53221206	120.7297078	-1.941333014	7.26E-11	6.79E-09
ENSG00000196954	CASP4	547.9654809	1203.486938	-1.135727645	1.06E-10	9.85E-09
ENSG00000187867	PALM3	29.21636052	137.9766899	-2.234585946	1.08E-10	9.93E-09
ENSG00000141753	IGFBP4	145.187009	399.4231525	-1.461623016	1.11E-10	1.02E-08
ENSG00000140044	JDP2	333.2161162	863.2761345	-1.373274015	1.26E-10	1.15E-08
ENSG00000106003	LFNG	329.0016033	657.1037992	-0.998180236	1.36E-10	1.24E-08
ENSG00000150540	HNMT	63.87505842	183.6508055	-1.525099614	1.41E-10	1.28E-08
ENSG00000245849	RAD51-AS1	242.3999043	507.0492857	-1.06468172	1.62E-10	1.45E-08
ENSG00000170390	DCLK2	238.4570211	91.9996663	1.376136747	1.64E-10	1.46E-08
ENSG00000124496	TRERF1	345.9846335	645.9176801	-0.90068387	1.99E-10	1.77E-08
ENSG00000244586	WNT5A-AS1	59.63686519	6.649278866	3.176011617	2.13E-10	1.89E-08
ENSG00000165275	TRMT10B	209.424144	430.1929588	-1.038310034	2.22E-10	1.93E-08
ENSG00000075461	CACNG4	57.46333484	245.8363467	-2.095979062	2.22E-10	1.93E-08
ENSG00000173391	OLR1	4378.008196	2384.853372	0.875977191	2.22E-10	1.93E-08
ENSG00000162545	CAMK2N1	1541.705074	2866.630927	-0.894912006	2.23E-10	1.93E-08
ENSG00000136098	NEK3	143.525585	328.4561487	-1.193179773	2.42E-10	2.08E-08
ENSG00000183036	PCP4	182.5492394	459.2226951	-1.33248463	2.55E-10	2.18E-08
ENSG00000163283	ALPP	558.1455693	199.7548932	1.483653674	2.62E-10	2.23E-08
ENSG00000228624	HDAC2-AS2	930.9542415	1772.415918	-0.928891949	2.79E-10	2.36E-08
ENSG00000179532	DNHD1	1125.766987	1959.415586	-0.798968863	2.80E-10	2.36E-08
ENSG00000101230	ISM1	1280.092783	735.3404191	0.79985046	2.92E-10	2.45E-08
ENSG00000177707	NECTIN3	432.8096608	206.539707	1.064923408	3.41E-10	2.85E-08
ENSG00000057252	SOAT1	916.1161549	528.9577795	0.792935256	3.49E-10	2.90E-08
ENSG00000116285	ERRFI1	8068.344467	14851.18941	-0.880261412	3.55E-10	2.93E-08

ENSG00000135083	CCNJL	8.681456695	72.24282325	-3.051502839	3.66E-10	3.01E-08
ENSG00000090861	AARS	4944.687737	8855.438954	-0.840696513	3.70E-10	3.02E-08
ENSG00000080819	CPOX	7149.463716	4558.686143	0.649148111	3.79E-10	3.08E-08
ENSG00000142002	DPP9	3318.845243	1960.10031	0.759918554	3.86E-10	3.12E-08
ENSG00000165215	CLDN3	365.0610007	736.6984892	-1.013117039	3.93E-10	3.16E-08
ENSG00000070669	ASNS	180.7349373	484.0936341	-1.419504715	4.28E-10	3.43E-08
ENSG00000144026	ZNF514	143.4039778	330.372306	-1.204353137	5.23E-10	4.17E-08
ENSG00000058668	ATP2B4	373.3221149	783.8786453	-1.071432777	5.40E-10	4.29E-08
ENSG00000172164	SNTB1	2975.021692	5019.221954	-0.754634624	6.27E-10	4.95E-08
ENSG00000075275	CELSR1	880.3685874	1500.228199	-0.768839522	6.91E-10	5.43E-08
ENSG00000023909	GCLM	2439.3213	1542.072711	0.661753697	6.97E-10	5.45E-08
ENSG00000111859	NEDD9	262.0913198	505.3024125	-0.946500051	7.38E-10	5.75E-08
ENSG00000167207	NOD2	31.68881863	122.9807652	-1.957813772	9.03E-10	7.00E-08
ENSG00000236104	ZBTB22	387.4886545	699.8355249	-0.853133326	9.89E-10	7.63E-08
ENSG00000143507	DUSP10	358.7668077	695.5419389	-0.95633708	1.01E-09	7.77E-08
ENSG00000115993	TRAK2	1545.452393	976.336262	0.662387241	1.03E-09	7.84E-08
ENSG00000273079	GRIN2B	187.3619399	382.5199959	-1.031430867	1.15E-09	8.72E-08
ENSG00000154783	FGD5	22.26549135	101.1973922	-2.186637686	1.19E-09	8.99E-08
ENSG00000111981	ULBP1	165.4920379	454.9411449	-1.458343117	1.26E-09	9.46E-08
ENSG00000140450	ARRDC4	421.614791	153.6468097	1.459664495	1.28E-09	9.58E-08
ENSG00000272588	AC139887.4	54.3972528	7.987082134	2.762407027	1.33E-09	9.90E-08
ENSG00000171130	ATP6V0E2	1052.35823	541.6574386	0.958561592	1.42E-09	1.06E-07
ENSG00000125968	ID1	4055.909507	7040.513814	-0.795753406	1.58E-09	1.17E-07
ENSG00000117519	CNN3	1940.675288	3536.261814	-0.865688442	1.62E-09	1.20E-07
ENSG00000186469	GNG2	19.56893551	95.49612334	-2.281167325	1.67E-09	1.23E-07
ENSG00000186472	PCLO	67.54232468	12.63676948	2.408813	1.78E-09	1.30E-07
ENSG00000138002	IFT172	192.6620526	382.5207097	-0.989205491	1.84E-09	1.34E-07
ENSG00000127863	TNFRSF19	836.7993038	1386.202338	-0.72845624	1.95E-09	1.41E-07
ENSG00000143502	SUSD4	128.6142675	274.5652251	-1.0950603	1.97E-09	1.42E-07
ENSG00000163877	SNIP1	902.703153	534.3572768	0.756454356	2.22E-09	1.59E-07
ENSG00000118620	ZNF430	293.3148609	143.0196062	1.037181495	2.24E-09	1.60E-07
ENSG00000198682	PAPSS2	3033.543401	4937.951931	-0.703092578	2.44E-09	1.74E-07
ENSG00000152689	RASGRP3	75.36662885	15.6227247	2.275575109	2.54E-09	1.79E-07
ENSG00000184956	MUC6	46.02212182	156.771005	-1.765598591	2.54E-09	1.79E-07
ENSG00000182389	CACNB4	72.2165733	188.069659	-1.3781679	2.57E-09	1.81E-07
ENSG00000159082	SYNJ1	907.5379507	518.4534392	0.808643075	2.94E-09	2.05E-07
ENSG00000106853	PTGR1	1643.965604	1029.592713	0.674579249	3.06E-09	2.13E-07
ENSG00000173156	RHOD	1359.257326	835.7942924	0.701745314	3.11E-09	2.15E-07
ENSG00000108813	DLX4	108.4764396	285.0074941	-1.395925644	3.26E-09	2.25E-07
ENSG00000264070	DND1P1	149.5578769	333.8538967	-1.15807215	3.36E-09	2.31E-07
ENSG00000187815	ZFP69	97.02145174	236.0502791	-1.283398754	3.57E-09	2.44E-07
ENSG00000111275	ALDH2	2134.469994	3788.340827	-0.827794489	3.91E-09	2.66E-07
ENSG00000078814	MYH7B	235.2728389	430.586262	-0.872273848	4.14E-09	2.81E-07

ENSG00000184575	XPOT	4401.761037	7861.746615	-0.836794831	4.25E-09	2.87E-07
ENSG00000188177	ZC3H6	451.4056372	931.0849884	-1.044387493	4.48E-09	3.01E-07
ENSG00000106144	CASP2	3515.89819	2292.394978	0.617054856	4.84E-09	3.25E-07
ENSG00000169258	GPRIN1	1151.537581	569.6116359	1.016320292	5.13E-09	3.42E-07
ENSG00000244405	ETV5	1301.561017	2272.865265	-0.804342763	5.38E-09	3.58E-07
ENSG00000005187	ACSM3	43.56425231	140.5229383	-1.693512954	5.43E-09	3.59E-07
ENSG00000203985	LDLRAD1	33.2130117	151.6161742	-2.187147026	5.59E-09	3.69E-07
ENSG00000065911	MTHFD2	5788.062394	9931.031111	-0.778876052	5.62E-09	3.69E-07
ENSG00000109689	STIM2	1906.690925	1192.057344	0.677165732	5.99E-09	3.92E-07
ENSG00000244187	TMEM141	959.0478789	1588.624103	-0.727772339	6.28E-09	4.09E-07
ENSG00000110619	CARS	2994.811317	5190.072907	-0.793251241	6.37E-09	4.13E-07
ENSG00000105559	PLEKHA4	76.87964383	211.2975666	-1.45946467	6.77E-09	4.38E-07
ENSG00000074935	TUBE1	557.1000234	1163.73631	-1.062686826	6.86E-09	4.42E-07
ENSG00000241186	TDGF1	36.08127322	121.6832076	-1.754291308	7.42E-09	4.76E-07
ENSG00000137877	SPTBN5	512.3535974	246.2399306	1.058456135	7.52E-09	4.80E-07
ENSG00000138613	APH1B	134.2004576	297.4152914	-1.147646632	7.73E-09	4.92E-07
ENSG00000140479	PCSK6	2099.137233	1329.855299	0.657978291	7.85E-09	4.97E-07
ENSG00000137834	SMAD6	54.36970304	166.2140959	-1.614270226	8.64E-09	5.46E-07
ENSG00000115271	GCA	400.2206785	192.098432	1.057168717	8.74E-09	5.49E-07
ENSG00000137440	FGFBP1	488.8684268	916.9420547	-0.908359179	9.03E-09	5.66E-07
ENSG00000167657	DAPK3	2216.968331	1252.315124	0.824156671	9.07E-09	5.66E-07
ENSG00000129521	EGLN3	140.7735815	320.0646393	-1.187132756	9.16E-09	5.67E-07
ENSG00000144824	PHLDB2	630.7998489	1013.185218	-0.683649644	9.16E-09	5.67E-07
ENSG00000127445	PIN1	2390.905129	1353.121398	0.821475413	9.31E-09	5.74E-07
ENSG00000145284	SCD5	416.042938	722.131265	-0.795753701	1.02E-08	6.28E-07
ENSG00000161267	BDH1	271.0204366	507.8408565	-0.906625596	1.04E-08	6.34E-07
ENSG00000204264	PSMB8	381.8424228	674.1454486	-0.821319445	1.09E-08	6.66E-07
ENSG00000280334	AC009084.2	4.440356071	49.49756366	-3.490697377	1.09E-08	6.66E-07
ENSG00000266714	MYO15B	1464.744816	2557.196508	-0.804009104	1.12E-08	6.77E-07
ENSG00000141756	FKBP10	34.55468326	115.2050173	-1.736102818	1.13E-08	6.84E-07
ENSG00000148468	FAM171A1	244.7885513	443.1156017	-0.85687453	1.15E-08	6.89E-07
ENSG00000227619	AL391056.1	2.152305859	41.74259633	-4.261116941	1.19E-08	7.15E-07
ENSG00000177469	CAVIN1	3281.894713	1838.06985	0.836668951	1.21E-08	7.20E-07
ENSG00000283646	LINC02009	47.17015281	140.0937731	-1.573371236	1.23E-08	7.30E-07
ENSG00000188385	JAKMIP3	78.78383079	17.5304666	2.166773727	1.35E-08	8.02E-07
ENSG00000168758	SEMA4C	577.0837523	974.1757257	-0.755185691	1.43E-08	8.47E-07
ENSG00000180694	TMEM64	1052.549415	649.7681101	0.696208419	1.46E-08	8.57E-07
ENSG00000162772	ATF3	290.4102168	543.6143938	-0.903578767	1.51E-08	8.85E-07
ENSG00000147202	DIAPH2	573.921187	274.4134304	1.062342839	1.52E-08	8.88E-07
ENSG00000008513	ST3GAL1	119.2073119	270.2420491	-1.182324174	1.53E-08	8.91E-07
ENSG00000166446	CDYL2	217.7923876	87.74360014	1.314422695	1.64E-08	9.53E-07
ENSG00000164342	TLR3	219.0573944	449.9017386	-1.039997575	1.68E-08	9.71E-07
ENSG00000212901	KRTAP3-1	37.6203655	1.54114475	4.645503092	1.69E-08	9.74E-07

ENSG00000153879	CEBPG	1492.884106	2473.521021	-0.728462654	1.73E-08	9.92E-07
ENSG00000119185	ITGB1BP1	1834.574593	1118.026099	0.714889485	1.75E-08	1.00E-06
ENSG00000136051	WASHC4	1757.974363	2686.312068	-0.612039407	2.03E-08	1.16E-06
ENSG00000167703	SLC43A2	48.13106841	129.7773255	-1.429622659	2.11E-08	1.19E-06
ENSG00000067798	NAV3	769.1133639	477.0310804	0.688631472	2.11E-08	1.19E-06
ENSG00000060140	STYK1	543.7577448	297.1042307	0.872632582	2.15E-08	1.21E-06
ENSG00000105810	CDK6	2127.73742	1335.649839	0.67218426	2.19E-08	1.23E-06
ENSG00000275896	PRSS2	124.4098903	797.4114671	-2.680480071	2.21E-08	1.24E-06
ENSG00000153714	LURAP1L	134.6425276	378.6947092	-1.491564241	2.21E-08	1.24E-06
ENSG00000135968	GCC2	5262.244287	8258.252667	-0.650167668	2.40E-08	1.33E-06
ENSG00000137819	PAQR5	241.7109289	451.397262	-0.901121296	2.44E-08	1.35E-06
ENSG00000159228	CBR1	177.9324355	353.9485654	-0.991893866	2.44E-08	1.35E-06
ENSG00000160991	ORAI2	200.8275712	385.311636	-0.938633236	2.51E-08	1.38E-06
ENSG00000197982	C1orf122	876.2542198	506.4776585	0.791341574	2.54E-08	1.39E-06
ENSG00000172482	AGXT	39.21408434	134.6576696	-1.776811688	2.56E-08	1.40E-06
ENSG00000138696	BMPR1B	527.3434587	291.3178983	0.854446413	2.67E-08	1.45E-06
ENSG00000112576	CCND3	1503.558922	707.41284	1.088254433	2.68E-08	1.45E-06
ENSG00000225190	PLEKHM1	4006.051933	6455.296832	-0.688307283	2.69E-08	1.46E-06
ENSG00000140968	IRF8	12.59082717	67.93663976	-2.44335129	2.81E-08	1.52E-06
ENSG00000106105	GARS	6844.380671	11255.87777	-0.717717223	2.85E-08	1.53E-06
ENSG00000079313	REXO1	1842.265484	1227.508723	0.585595872	3.07E-08	1.64E-06
ENSG00000168209	DDIT4	727.7264615	4579.493041	-2.653620196	3.11E-08	1.66E-06
ENSG00000113319	RASGRF2	301.4957302	604.2929274	-1.003459615	3.17E-08	1.69E-06
ENSG00000129757	CDKN1C	205.569052	382.7307477	-0.895921561	3.36E-08	1.78E-06
ENSG00000101849	TBL1X	1242.491624	2050.051907	-0.722587496	3.47E-08	1.83E-06
ENSG00000103888	CEMIP	9221.471617	16325.59719	-0.824128084	3.80E-08	2.00E-06
ENSG00000188483	IER5L	330.143568	560.169084	-0.76269543	4.01E-08	2.11E-06
ENSG00000170921	TANC2	742.0156931	1149.861672	-0.631976984	4.12E-08	2.16E-06
ENSG00000180573	HIST1H2AC	260.5528038	89.93989377	1.533893939	4.19E-08	2.19E-06
ENSG00000172159	FRMD3	200.6851909	497.7573082	-1.310728713	4.31E-08	2.25E-06
ENSG00000132773	TOE1	1059.952311	637.6702217	0.733453311	4.33E-08	2.25E-06
ENSG00000150938	CRIM1	7852.362018	5213.095846	0.590841205	4.60E-08	2.38E-06
ENSG00000001617	SEMA3F	1221.987725	2054.745273	-0.74994048	4.65E-08	2.40E-06
ENSG00000132170	PPARG	665.9987952	1295.132917	-0.959809785	4.91E-08	2.53E-06
ENSG00000198026	ZNF335	1927.268767	1295.174226	0.573410806	5.21E-08	2.67E-06
ENSG00000173848	NET1	3846.109628	6183.297401	-0.685110766	5.27E-08	2.70E-06
ENSG00000169683	LRRC45	1394.679229	800.937502	0.800492328	5.40E-08	2.75E-06
ENSG00000276043	UHRF1	4711.922386	2571.390357	0.873964046	5.50E-08	2.79E-06
ENSG00000063046	EIF4B	12147.72347	18489.70269	-0.606080701	5.75E-08	2.91E-06
ENSG00000180596	HIST1H2BC	70.91540529	17.28587433	2.047316831	5.77E-08	2.91E-06
ENSG00000103064	SLC7A6	1750.383662	1122.309075	0.641410454	5.82E-08	2.93E-06
ENSG00000228300	C19orf24	1605.248667	982.6941319	0.708222555	6.08E-08	3.05E-06
ENSG00000136436	CALCOCO2	1659.242343	2878.446831	-0.795043893	6.38E-08	3.19E-06

ENSG00000157150	TIMP4	84.5273895	241.399366	-1.513231047	6.54E-08	3.26E-06
ENSG00000005379	TSPOAP1	241.250142	543.370369	-1.172479969	6.56E-08	3.26E-06
ENSG00000071242	RPS6KA2	226.0817103	454.0145682	-1.006691538	6.58E-08	3.26E-06
ENSG00000138449	SLC40A1	2.152305859	36.19510375	-4.052569242	6.63E-08	3.28E-06
ENSG00000121690	DEPDC7	58.96141307	152.6912866	-1.373901762	6.81E-08	3.36E-06
ENSG00000158373	HIST1H2BD	202.5924476	82.90153547	1.292563036	6.94E-08	3.41E-06
ENSG00000087266	SH3BP2	1540.628862	2638.075791	-0.775923586	7.02E-08	3.44E-06
ENSG00000122861	PLAU	47.98843661	141.1440583	-1.556447592	7.25E-08	3.54E-06
ENSG00000187091	PLCD1	189.172869	364.0793475	-0.945605892	7.31E-08	3.56E-06
ENSG00000184232	OAF	425.7118175	812.439416	-0.932798981	7.69E-08	3.73E-06
ENSG00000021300	PLEKHB1	99.28776697	221.7087894	-1.160219047	7.83E-08	3.79E-06
ENSG00000127954	STEAP4	203.6882348	403.5537341	-0.988023205	7.93E-08	3.82E-06
ENSG00000166444	ST5	408.5704641	718.2850742	-0.814861658	7.95E-08	3.83E-06
ENSG00000203727	SAMD5	2165.992565	1396.436663	0.633178185	8.35E-08	4.00E-06
ENSG00000128165	ADM2	9.383385907	72.89051609	-2.950674201	8.44E-08	4.04E-06
ENSG00000168453	HR	76.69977814	242.4216488	-1.660800665	8.75E-08	4.17E-06
ENSG00000175745	NR2F1	497.3939055	286.6364092	0.794175709	8.82E-08	4.19E-06
ENSG00000125775	SDCBP2	244.69806	96.96580313	1.333587104	9.03E-08	4.28E-06
ENSG00000184292	TACSTD2	1327.822036	5196.421871	-1.968474062	9.10E-08	4.30E-06
ENSG00000243766	HOTTIP	2382.152121	3629.073331	-0.607545373	9.34E-08	4.40E-06
ENSG00000109321	AREG	5079.137251	7886.696655	-0.634889861	9.71E-08	4.56E-06
ENSG00000122591	FAM126A	290.6559013	134.787612	1.108860085	9.74E-08	4.56E-06
ENSG00000154040	CABYR	381.4032085	210.1330066	0.859708052	1.02E-07	4.78E-06
ENSG00000106025	TSPAN12	181.379022	75.56402682	1.261541853	1.10E-07	5.11E-06
ENSG00000127564	PKMYT1	2640.974196	1395.044577	0.921128115	1.10E-07	5.13E-06
ENSG00000237721	AF064858.3	7.723448485	53.23048882	-2.793697999	1.13E-07	5.21E-06
ENSG00000004399	PLXND1	102.8089835	238.5366967	-1.215387249	1.13E-07	5.21E-06
ENSG00000110195	FOLR1	119.4956828	281.8291901	-1.240108261	1.13E-07	5.21E-06
ENSG00000158406	HIST1H4H	74.7557605	16.6835229	2.17298486	1.17E-07	5.35E-06
ENSG00000073605	GSDMB	323.2518676	550.7786306	-0.768333583	1.18E-07	5.41E-06
ENSG00000112531	QKI	406.965005	219.5001325	0.893365161	1.21E-07	5.52E-06
ENSG00000187840	EIF4EBP1	1704.35589	2622.800204	-0.621940219	1.21E-07	5.52E-06
ENSG00000110680	CALCA	0.364584188	41.36609718	-6.721519374	1.22E-07	5.52E-06
ENSG00000100219	XBP1	2713.711239	4701.593574	-0.792965754	1.24E-07	5.60E-06
ENSG00000163629	PTPN13	2252.019114	3795.385802	-0.753217872	1.26E-07	5.67E-06
ENSG00000006210	CX3CL1	18.72815466	72.76238186	-1.960561251	1.26E-07	5.68E-06
ENSG00000132635	PCED1A	322.2820674	657.6675496	-1.028590909	1.27E-07	5.68E-06
ENSG00000132749	TESMIN	337.5153861	553.8523448	-0.714954435	1.27E-07	5.68E-06
ENSG00000021645	NRXN3	255.0650511	497.8730999	-0.966527422	1.27E-07	5.68E-06
ENSG00000196220	SRGAP3	58.21025815	151.2701234	-1.378619984	1.28E-07	5.70E-06
ENSG00000101255	TRIB3	5099.185745	8761.422439	-0.780880075	1.28E-07	5.70E-06
ENSG00000233198	RNF224	202.8929586	376.7565797	-0.89301869	1.37E-07	6.06E-06
ENSG00000132359	RAP1GAP2	649.4506069	1057.727531	-0.703298537	1.38E-07	6.11E-06

ENSG00000135473	PAN2	1166.337583	2022.486267	-0.79428435	1.41E-07	6.20E-06
ENSG00000164088	PPM1M	166.5779927	332.0876706	-0.99620206	1.42E-07	6.23E-06
ENSG00000170949	ZNF160	408.3253934	709.6567597	-0.797764854	1.44E-07	6.32E-06
ENSG00000006047	YBX2	91.21136385	212.8337755	-1.22356705	1.50E-07	6.58E-06
ENSG00000179023	KLHDC7A	20.73201395	87.27832555	-2.074701712	1.51E-07	6.59E-06
ENSG00000106541	AGR2	7662.599022	12601.44868	-0.717756887	1.52E-07	6.59E-06
ENSG00000108511	HOXB6	2658.766686	4189.881232	-0.656125165	1.55E-07	6.74E-06
ENSG00000167741	GGT6	3.638954435	38.23878606	-3.393866293	1.58E-07	6.85E-06
ENSG00000096872	IFT74	297.1743328	523.9263528	-0.817989232	1.60E-07	6.92E-06
ENSG00000182985	CADM1	1941.639673	1021.577036	0.926718793	1.62E-07	6.98E-06
ENSG00000164252	AGGF1	317.783467	534.0195539	-0.749835112	1.64E-07	7.04E-06
ENSG00000237506	RPSAP15	575.7577346	315.3481923	0.867720441	1.68E-07	7.21E-06
ENSG00000169047	IRS1	7130.115119	10850.03245	-0.60574896	1.73E-07	7.38E-06
ENSG00000162600	OMA1	256.8888448	504.7408018	-0.975760429	1.76E-07	7.51E-06
ENSG00000120708	TGFBI	1918.367651	3469.669764	-0.855243325	1.77E-07	7.53E-06
ENSG00000116729	WLS	1610.727833	2361.08683	-0.551880336	1.78E-07	7.56E-06
ENSG00000167775	CD320	2094.900192	1201.648618	0.802050485	1.84E-07	7.78E-06
ENSG00000198431	TXNRD1	24715.5913	17157.52616	0.526586277	1.99E-07	8.40E-06
ENSG00000196420	S100A5	106.1718179	34.55717357	1.619004155	2.05E-07	8.62E-06
ENSG00000123104	ITPR2	2938.652003	4783.185843	-0.702805796	2.09E-07	8.76E-06
ENSG00000144468	RHBDD1	1000.625879	1522.315158	-0.605554718	2.20E-07	9.19E-06
ENSG00000027075	PRKCH	69.71820025	162.8939848	-1.226470798	2.23E-07	9.32E-06
ENSG00000243742	RPLP0P2	251.7485941	570.9988526	-1.181621542	2.26E-07	9.41E-06
ENSG00000143631	FLG	233.5174608	111.3595381	1.068313501	2.28E-07	9.48E-06
ENSG00000167914	GSDMA	119.7383491	271.5247974	-1.183736126	2.32E-07	9.59E-06
ENSG00000149328	GLB1L2	2328.368326	1509.590618	0.62516843	2.37E-07	9.79E-06
ENSG00000196705	ZNF431	599.262784	344.5563075	0.799552766	2.43E-07	1.00E-05
ENSG00000140807	NKD1	795.4590586	1214.269834	-0.61053349	2.58E-07	1.06E-05
ENSG00000168268	NT5DC2	457.0276619	895.4315233	-0.970248934	2.67E-07	1.09E-05
ENSG00000003137	CYP26B1	967.3404338	593.6088435	0.705089352	2.74E-07	1.12E-05
ENSG00000188613	NANOS1	379.5460718	195.7212015	0.957413861	2.80E-07	1.14E-05
ENSG00000071794	HLTF	8.760577396	52.29453551	-2.581826275	2.90E-07	1.18E-05
ENSG00000140323	DISP2	94.07830114	32.23284672	1.541972911	2.96E-07	1.20E-05
ENSG00000166922	SCG5	66.35782186	175.1701551	-1.401533845	3.05E-07	1.24E-05
ENSG00000183844	FAM3B	53.63818944	138.3998857	-1.37194597	3.14E-07	1.27E-05
ENSG00000164949	GEM	609.178783	360.8533151	0.754412696	3.17E-07	1.28E-05
ENSG00000175130	MARCKSL1	3391.589526	4894.377093	-0.529177051	3.24E-07	1.29E-05
ENSG00000130513	GDF15	4751.943792	8172.804575	-0.782310115	3.24E-07	1.29E-05
ENSG00000253846	PCDHGA10	32.92918304	100.4200786	-1.611972487	3.24E-07	1.29E-05
ENSG00000168710	AHCYL1	5599.468039	8255.858742	-0.560210212	3.27E-07	1.30E-05
ENSG00000116771	AGMAT	31.73090534	94.78747855	-1.580720354	3.30E-07	1.31E-05
ENSG00000174938	SEZ6L2	624.2665556	335.9018753	0.892292121	3.36E-07	1.33E-05
ENSG00000171522	PTGER4	91.33073653	32.49156294	1.491398353	3.46E-07	1.37E-05

ENSG00000179388	EGR3	8.085125284	46.41753168	-2.5253159	3.47E-07	1.37E-05
ENSG00000206075	SERPINB5	1881.226879	555.8554181	1.758432977	3.60E-07	1.42E-05
ENSG00000166562	SEC11C	797.1052427	494.3046371	0.688837028	3.82E-07	1.50E-05
ENSG00000196776	CD47	1292.62627	1938.633921	-0.584842392	3.89E-07	1.53E-05
ENSG00000090674	MCOLN1	544.112504	310.1180818	0.810882872	4.01E-07	1.57E-05
ENSG00000121310	ECHDC2	355.6444501	631.0475578	-0.827108476	4.15E-07	1.62E-05
ENSG00000253361	AC069120.1	211.0020534	453.7300767	-1.104587011	4.46E-07	1.74E-05
ENSG00000146376	ARHGAP18	1319.455138	2187.479003	-0.729745903	4.67E-07	1.81E-05
ENSG00000116977	LGALS8	2399.774495	3527.942639	-0.556084567	4.78E-07	1.85E-05
ENSG00000131069	ACSS2	2110.991822	3542.835396	-0.747055727	4.78E-07	1.85E-05
ENSG00000185133	INPP5J	241.013128	494.0333085	-1.034908026	4.91E-07	1.89E-05
ENSG00000103485	QPRT	52.56586126	153.1302949	-1.547269594	4.98E-07	1.91E-05
ENSG00000164292	RHOBTB3	14884.71333	22198.28386	-0.576649511	5.00E-07	1.91E-05
ENSG00000105467	SYNGR4	85.88732649	201.1224749	-1.225194366	5.00E-07	1.91E-05
ENSG00000118777	ABCG2	200.9025636	96.82734432	1.056166946	5.00E-07	1.91E-05
ENSG00000143443	C1orf56	40.25866285	106.2269269	-1.402041187	5.34E-07	2.04E-05
ENSG00000242193	CRYZL2P	27.98451838	95.7970669	-1.776698486	5.39E-07	2.05E-05
ENSG00000100258	LMF2	2402.498103	1549.469631	0.632945485	5.41E-07	2.05E-05
ENSG00000178026	LRRC75B	234.1524469	469.2904979	-1.002650389	5.44E-07	2.06E-05
ENSG00000182264	IZUMO1	107.0080565	259.4919497	-1.276604563	5.53E-07	2.09E-05
ENSG00000183248	PRR36	69.06759041	158.5846701	-1.197928191	5.57E-07	2.10E-05
ENSG00000232480	TGFB2-AS1	103.5535616	232.469063	-1.169147266	5.67E-07	2.13E-05
ENSG00000224877	NDUFAF8	754.944938	439.2059954	0.782952594	5.93E-07	2.23E-05
ENSG00000126391	FRMD8	1373.11458	919.2440552	0.579499662	6.04E-07	2.26E-05
ENSG00000099337	KCNK6	101.1491052	212.4277036	-1.072263095	6.08E-07	2.27E-05
ENSG00000167767	KRT80	3816.105649	6314.631351	-0.726653451	6.17E-07	2.30E-05
ENSG00000101210	EEF1A2	78.21601111	194.3487737	-1.312928722	6.20E-07	2.30E-05
ENSG00000181031	RPH3AL	429.1296923	797.6622512	-0.895088164	6.38E-07	2.37E-05
ENSG00000136280	CCM2	988.8997267	651.9639526	0.601195488	6.40E-07	2.37E-05
ENSG00000169635	HIC2	579.8154698	336.1633419	0.787754199	6.45E-07	2.38E-05
ENSG00000145569	OTULINL	51.4199756	12.04883325	2.101599197	6.53E-07	2.41E-05
ENSG00000185697	MYBL1	421.792622	212.6795766	0.98823475	6.72E-07	2.47E-05
ENSG00000007968	E2F2	575.9919378	329.2587407	0.806923769	6.75E-07	2.47E-05
ENSG00000129968	ABHD17A	3285.77408	2227.614771	0.56086486	6.84E-07	2.50E-05
ENSG00000170412	GPRC5C	131.449675	324.0739852	-1.302564894	6.88E-07	2.51E-05
ENSG00000145911	N4BP3	41.53151904	129.1587239	-1.637271222	7.11E-07	2.59E-05
ENSG00000095637	SORBS1	2453.279995	1528.264078	0.68291033	7.34E-07	2.67E-05
ENSG00000134202	GSTM3	1918.749881	2954.726929	-0.622903881	7.51E-07	2.72E-05
ENSG00000159184	HOXB13	153.4561799	287.3391856	-0.905196618	7.60E-07	2.74E-05
ENSG00000107331	ABCA2	1932.249536	3064.253884	-0.665257981	7.60E-07	2.74E-05
ENSG00000099250	NRP1	1061.126516	600.5305323	0.821169021	7.62E-07	2.75E-05
ENSG00000116096	SPR	2114.526507	1297.385883	0.704984007	7.64E-07	2.75E-05
ENSG00000155304	HSPA13	1264.808545	2082.823562	-0.719893417	7.71E-07	2.76E-05

ENSG00000157992	KRTCAP3	62.8752741	143.0389572	-1.186216831	7.99E-07	2.86E-05
ENSG00000214049	UCA1	80.27680465	197.8450708	-1.300784097	8.03E-07	2.87E-05
ENSG00000145506	NKD2	222.3089053	411.4922236	-0.887073199	8.14E-07	2.90E-05
ENSG00000131242	RAB11FIP4	2030.635036	2945.337561	-0.536688274	8.20E-07	2.92E-05
ENSG00000130522	JUND	1430.876614	2091.938	-0.547922873	8.31E-07	2.95E-05
ENSG00000205060	SLC35B4	863.7674965	517.8966652	0.738401427	8.53E-07	3.02E-05
ENSG00000237886	NALT1	50.58230204	131.2261695	-1.373835778	8.89E-07	3.14E-05
ENSG00000117410	ATP6V0B	2735.913844	1780.003386	0.620411631	9.23E-07	3.25E-05
ENSG00000230699	AL645608.2	21.5461178	76.21230215	-1.823569327	9.27E-07	3.26E-05
ENSG00000185633	NDUFA4L2	20.42803346	77.63563544	-1.927157179	9.85E-07	3.45E-05
ENSG00000262877	AC110285.2	361.6994463	635.002492	-0.810817787	9.86E-07	3.45E-05
ENSG00000196924	FLNA	12493.90811	8842.585961	0.498755414	1.01E-06	3.55E-05
ENSG00000165449	SLC16A9	152.7703708	276.5085681	-0.85517805	1.02E-06	3.57E-05
ENSG00000204282	TNRC6C-AS1	257.7888534	476.195653	-0.885154762	1.04E-06	3.61E-05
ENSG00000101412	E2F1	1566.269712	964.2209277	0.700616605	1.08E-06	3.75E-05
ENSG00000176641	RNF152	27.08022643	2.376541804	3.507160949	1.10E-06	3.81E-05
ENSG00000167670	CHAF1A	2918.885305	1609.932852	0.858729877	1.11E-06	3.85E-05
ENSG00000104825	NFKBIB	693.6702326	430.6778489	0.688013383	1.14E-06	3.94E-05
ENSG00000164741	DLC1	315.3451355	171.1748304	0.883575463	1.14E-06	3.94E-05
ENSG00000186642	PDE2A	26.32565362	111.4002046	-2.081785737	1.16E-06	3.98E-05
ENSG00000128268	MGAT3	46.97620164	9.77597895	2.25955895	1.20E-06	4.12E-05
ENSG00000128564	VGF	1390.595824	857.2111656	0.69782531	1.22E-06	4.19E-05
ENSG00000271303	SRXN1	403.8728081	217.0721467	0.898516329	1.24E-06	4.23E-05
ENSG00000144224	UBXN4	4125.309395	5949.842397	-0.528484663	1.25E-06	4.25E-05
ENSG00000196449	YRDC	848.8388755	522.2962687	0.701530391	1.25E-06	4.25E-05
ENSG00000115902	SLC1A4	4338.150024	6369.985708	-0.554229504	1.26E-06	4.26E-05
ENSG00000001626	CFTR	500.6356069	980.9442115	-0.971482835	1.28E-06	4.34E-05
ENSG00000065361	ERBB3	3257.604706	5384.587623	-0.725081421	1.33E-06	4.49E-05
ENSG00000069812	HES2	78.448831	209.9803935	-1.418971722	1.33E-06	4.49E-05
ENSG00000185090	MANEAL	1195.278362	673.8680187	0.827621163	1.34E-06	4.52E-05
ENSG00000273199	AP000692.2	46.24570904	10.16389339	2.189414953	1.35E-06	4.53E-05
ENSG00000235501	AC105942.1	17.82330055	62.83917856	-1.815047491	1.36E-06	4.55E-05
ENSG00000127955	GNAI1	682.4763174	383.348624	0.83167141	1.36E-06	4.56E-05
ENSG00000166436	TRIM66	2960.048632	4447.146011	-0.587157258	1.37E-06	4.56E-05
ENSG00000166816	LDHD	9.828925521	51.36025541	-2.387162427	1.39E-06	4.61E-05
ENSG00000025770	NCAPH2	1858.261373	1167.938839	0.670304813	1.40E-06	4.66E-05
ENSG00000170439	METTL7B	29.45264996	94.76884138	-1.687448285	1.43E-06	4.75E-05
ENSG00000103187	COTL1	2290.442964	1403.915794	0.706150465	1.45E-06	4.81E-05
ENSG00000151748	SAV1	1376.144198	932.8176689	0.560888813	1.46E-06	4.84E-05
ENSG00000123989	CHPF	5818.193447	4133.317177	0.493207565	1.49E-06	4.92E-05
ENSG00000198088	NUP62CL	41.23044594	106.684894	-1.373521601	1.51E-06	4.97E-05
ENSG00000140105	WARS	2418.929242	3484.829147	-0.526838183	1.52E-06	4.99E-05
ENSG00000167165	UGT1A6	42.74433368	8.08686746	2.380216787	1.54E-06	5.05E-05

ENSG00000134030	CTIF	729.6659652	1288.10032	-0.819692428	1.56E-06	5.10E-05
ENSG00000115649	CNPPD1	1105.061908	759.7224421	0.540856306	1.56E-06	5.10E-05
ENSG00000183853	KIRREL1	282.3528036	490.8095639	-0.79795362	1.64E-06	5.34E-05
ENSG00000168672	FAM84B	878.7221518	1438.626649	-0.711231258	1.70E-06	5.52E-05
ENSG00000065060	UHRF1BP1	2360.117348	3336.13439	-0.499294096	1.72E-06	5.59E-05
ENSG00000099904	ZDHHC8	634.5480706	957.9885957	-0.593667238	1.74E-06	5.63E-05
ENSG00000110492	MDK	61.28012034	136.3973228	-1.154277004	1.77E-06	5.71E-05
ENSG00000115112	TFCP2L1	283.813175	160.6534405	0.819926367	1.78E-06	5.74E-05
ENSG00000139800	ZIC5	321.0724259	524.4083572	-0.707824716	1.78E-06	5.74E-05
ENSG00000113504	SLC12A7	5778.325434	4067.325092	0.506665787	1.80E-06	5.80E-05
ENSG00000008294	SPAG9	4432.032501	3083.639775	0.523122086	1.81E-06	5.80E-05
ENSG00000188732	FAM221A	129.8920657	262.3060169	-1.013101806	1.83E-06	5.87E-05
ENSG00000123612	ACVR1C	56.88001625	13.91890676	2.028144062	1.84E-06	5.87E-05
ENSG00000135838	NPL	25.59357786	77.18666806	-1.593157705	1.85E-06	5.89E-05
ENSG00000170775	GPR37	235.2810506	126.3104412	0.898702997	1.96E-06	6.22E-05
ENSG00000104969	SGTA	3833.485514	2567.786633	0.578120743	1.99E-06	6.32E-05
ENSG00000139289	PHLDA1	7045.280997	10001.1407	-0.505472713	2.01E-06	6.37E-05
ENSG00000175294	CATSPER1	60.3654714	144.8362409	-1.26139053	2.03E-06	6.41E-05
ENSG00000182287	AP1S2	589.8044198	366.9148176	0.684161198	2.05E-06	6.49E-05
ENSG00000198860	TSEN15	1364.67204	1988.31697	-0.543362031	2.06E-06	6.50E-05
ENSG00000175793	SFN	6803.304988	4495.590161	0.597774457	2.07E-06	6.50E-05
ENSG00000170899	GSTA4	38.97254228	132.7591597	-1.76886069	2.07E-06	6.50E-05
ENSG00000104805	NUCB1	1522.077162	2432.24589	-0.67653925	2.08E-06	6.50E-05
ENSG00000121644	DESI2	2437.256533	3392.870958	-0.477396357	2.10E-06	6.55E-05
ENSG00000180964	TCEAL8	291.8660601	493.6522408	-0.759962476	2.10E-06	6.56E-05
ENSG00000197355	UAP1L1	31.08667245	88.70832677	-1.513803984	2.11E-06	6.59E-05
ENSG00000006704	GTF2IRD1	1448.656549	2305.967336	-0.670764991	2.14E-06	6.64E-05
ENSG00000197016	ZNF470	2.903971282	33.94807995	-3.539951862	2.14E-06	6.65E-05
ENSG00000064601	CTSA	1955.732441	1362.022667	0.521686895	2.23E-06	6.90E-05
ENSG00000197956	S100A6	10384.85754	6733.249136	0.625018219	2.23E-06	6.90E-05
ENSG00000100473	COCH	146.3023342	268.7984625	-0.876946398	2.24E-06	6.91E-05
ENSG00000029153	ARNTL2	1448.036025	948.7489395	0.609886685	2.26E-06	6.96E-05
ENSG00000174951	FUT1	644.0198615	1162.148725	-0.85163546	2.29E-06	7.05E-05
ENSG00000175155	YPEL2	134.5941157	282.2361929	-1.06962504	2.42E-06	7.41E-05
ENSG00000139926	FRMD6	997.6110711	672.3923242	0.569563709	2.43E-06	7.43E-05
ENSG00000123136	DDX39A	3042.36441	1952.501669	0.639920944	2.43E-06	7.44E-05
ENSG00000091490	SEL1L3	3645.867884	5529.814188	-0.601133667	2.46E-06	7.52E-05
ENSG00000167680	SEMA6B	77.39976193	205.0080113	-1.404865743	2.50E-06	7.61E-05
ENSG00000104899	AMH	1100.671524	653.7829037	0.75201546	2.58E-06	7.84E-05
ENSG00000158457	TSPAN33	128.1100106	54.21268763	1.241725328	2.59E-06	7.85E-05
ENSG00000141873	SLC39A3	844.0750674	535.5797145	0.656374246	2.62E-06	7.94E-05
ENSG00000196440	ARMCX4	88.02912712	175.3049591	-0.994483786	2.72E-06	8.22E-05
ENSG00000153294	ADGRF4	386.1575398	674.6975934	-0.805937362	2.76E-06	8.33E-05

ENSG00000125826	RBCK1	6825.326189	11797.0818	-0.789430614	2.79E-06	8.40E-05
ENSG00000105401	CDC37	8738.70966	5698.892382	0.616771629	2.81E-06	8.43E-05
ENSG00000197535	MYO5A	2626.042764	1874.113805	0.486392825	2.81E-06	8.43E-05
ENSG00000138316	ADAMTS14	28.76051558	101.7010205	-1.82221765	2.82E-06	8.43E-05
ENSG00000172572	PDE3A	49.37928222	133.1641695	-1.431215461	2.85E-06	8.52E-05
ENSG00000113721	PDGFRB	13.8255767	56.12056089	-2.020503819	2.88E-06	8.59E-05
ENSG00000066322	ELOVL1	3338.496688	2392.130206	0.480647063	2.89E-06	8.60E-05
ENSG00000214814	FER1L6	84.35099337	223.5824721	-1.407119752	2.93E-06	8.70E-05
ENSG00000104964	AES	4491.968219	3318.037851	0.436964012	2.96E-06	8.79E-05
ENSG00000167513	CDT1	1769.143222	1200.271355	0.559828819	2.99E-06	8.86E-05
ENSG00000005801	ZNF195	1906.022842	2663.597462	-0.482629777	3.10E-06	9.16E-05
ENSG00000142065	ZFP14	157.6742214	283.0503886	-0.843124496	3.13E-06	9.23E-05
ENSG00000115255	REEP6	669.9625031	429.2401747	0.64191245	3.15E-06	9.27E-05
ENSG00000172766	NAA16	1013.345163	664.9734617	0.608902602	3.16E-06	9.28E-05
ENSG00000205420	KRT6A	28.54147057	3.259960377	3.125019563	3.18E-06	9.33E-05
ENSG00000121350	PYROXD1	770.080908	1179.268091	-0.615390538	3.29E-06	9.64E-05
ENSG00000169684	CHRNA5	274.3488337	142.360715	0.94939972	3.34E-06	9.78E-05
ENSG00000141522	ARHGDIA	4844.474154	3153.353691	0.619626136	3.40E-06	9.93E-05
ENSG00000116584	ARHGEF2	1354.329722	2136.692278	-0.657908849	3.41E-06	9.95E-05
ENSG00000088002	SULT2B1	1103.262705	759.3317333	0.538698943	3.42E-06	9.95E-05
ENSG00000138380	CARF	221.9895668	429.5569136	-0.953020302	3.43E-06	9.95E-05
ENSG00000049449	RCN1	2292.169618	3255.021951	-0.506206158	3.43E-06	9.95E-05
ENSG00000079999	KEAP1	3314.873441	2325.214358	0.511612533	3.44E-06	9.96E-05
ENSG00000240038	AMY2B	54.21651436	123.1691661	-1.181519167	3.45E-06	9.97E-05
ENSG00000126351	THRA	480.1953374	758.7861861	-0.66032982	3.46E-06	9.98E-05
ENSG00000101306	MYLK2	61.45320928	15.6744885	1.967118672	3.47E-06	9.98E-05
ENSG00000102804	TSC22D1	3570.61317	5649.317002	-0.662067327	3.50E-06	0.0001
ENSG00000054793	ATP9A	1748.758319	2734.95442	-0.64523073	3.53E-06	0.000101
ENSG00000186088	GSAP	91.15290547	202.9161761	-1.154605449	3.53E-06	0.000101
ENSG00000095906	NUBP2	1818.560905	1217.720862	0.578806868	3.57E-06	0.000102
ENSG00000142871	CYR61	2011.680363	1233.978911	0.704551342	3.57E-06	0.000102
ENSG00000065054	SLC9A3R2	718.0845978	1129.162695	-0.652787337	3.64E-06	0.000104
ENSG00000222033	LINC01124	78.48749981	167.4557818	-1.09376727	3.68E-06	0.000105
ENSG00000168874	ATOH8	205.5531834	468.3795053	-1.190076252	3.69E-06	0.000105
ENSG00000260285	AL133367.1	97.78020451	250.7742945	-1.358085502	3.92E-06	0.000111
ENSG00000167861	HID1	1282.083402	2140.602672	-0.739687734	3.93E-06	0.000111
ENSG00000012223	LTF	9.194487454	49.46812223	-2.43546782	3.97E-06	0.000112
ENSG00000125912	NCLN	5381.901624	3749.63127	0.521454268	4.03E-06	0.000114
ENSG00000150347	ARID5B	1222.335773	807.1702551	0.597687743	4.04E-06	0.000114
ENSG00000110108	TMEM109	2185.318811	1395.277054	0.647598555	4.16E-06	0.000117
ENSG00000143126	CELSR2	1007.853503	1438.497363	-0.513206713	4.26E-06	0.00012
ENSG00000198758	EPS8L3	1599.145128	2477.190078	-0.631570743	4.29E-06	0.00012
ENSG00000197747	S100A10	15450.84464	11039.81139	0.484948033	4.32E-06	0.000121

ENSG00000177406	AC021054.1	295.1564988	485.9223141	-0.719127556	4.35E-06	0.000122
ENSG00000101871	MID1	867.9800854	508.9329329	0.770244584	4.38E-06	0.000122
ENSG00000115738	ID2	5024.715912	7936.41316	-0.659553215	4.38E-06	0.000122
ENSG00000186417	GLDN	51.64774277	148.9879781	-1.52863348	4.52E-06	0.000126
ENSG00000178685	PARP10	655.150976	1010.673785	-0.625734653	4.55E-06	0.000126
ENSG00000246982	Z84485.1	53.6849666	152.3590363	-1.502928523	4.63E-06	0.000128
ENSG00000164764	SBSPON	931.6807749	1406.173677	-0.594637488	4.75E-06	0.000131
ENSG00000166908	PIP4K2C	2169.040085	1551.455651	0.483548797	4.94E-06	0.000136
ENSG00000130254	SAFB2	3307.72228	2041.212946	0.69668277	5.07E-06	0.000139
ENSG00000122884	P4HA1	1008.671994	1676.628325	-0.733668098	5.12E-06	0.000141
ENSG00000235703	LINC00894	97.47515136	210.8691265	-1.110592108	5.17E-06	0.000142
ENSG00000170689	HOXB9	5265.402474	8968.486602	-0.768348928	5.24E-06	0.000144
ENSG00000187189	TSPYL4	602.7613036	888.6062889	-0.559669906	5.28E-06	0.000144
ENSG00000110104	CCDC86	1298.466532	728.0613839	0.835305249	5.37E-06	0.000147
ENSG00000153292	ADGRF1	80.2617572	27.06243578	1.562542605	5.38E-06	0.000147
ENSG00000176046	NUPR1	62.05631743	478.1901623	-2.945556989	5.42E-06	0.000148
ENSG00000112312	GMNN	1177.475857	663.9944834	0.82710038	5.50E-06	0.000149
ENSG00000135919	SERPINE2	656.9678233	398.8632186	0.718299784	5.54E-06	0.00015
ENSG00000140443	IGF1R	671.7739428	1047.885059	-0.641641666	5.62E-06	0.000152
ENSG00000102445	RUBCNL	21.48260671	73.16210566	-1.768857341	5.71E-06	0.000154
ENSG00000183778	B3GALT5	401.7795818	231.5795436	0.795642367	5.72E-06	0.000154
ENSG00000088256	GNA11	2124.433902	1444.27406	0.556904898	5.76E-06	0.000155
ENSG00000077713	SLC25A43	153.3447674	284.6412738	-0.893941344	5.79E-06	0.000156
ENSG00000165490	DDIAS	956.4043572	510.6309127	0.905443268	5.82E-06	0.000156
ENSG00000236404	VLDLR-AS1	8.707933795	46.12653401	-2.402956794	5.85E-06	0.000157
ENSG00000119138	KLF9	745.42242	1173.258118	-0.654722491	5.85E-06	0.000157
ENSG00000198203	SULT1C2	369.1884327	1255.712238	-1.766020565	5.89E-06	0.000157
ENSG00000184368	MAP7D2	138.8633282	315.1075271	-1.182446959	5.92E-06	0.000158
ENSG00000184925	LCN12	151.1489989	321.6364476	-1.08779216	6.03E-06	0.00016
ENSG00000106789	CORO2A	112.3633131	237.6292236	-1.082521614	6.03E-06	0.00016
ENSG00000152133	GPATCH11	1144.407054	741.1464852	0.626282661	6.15E-06	0.000163
ENSG00000033050	ABCF2	798.5215444	485.3943265	0.718725914	6.19E-06	0.000164
ENSG00000101040	ZMYND8	1997.277151	3003.89839	-0.589179418	6.30E-06	0.000167
ENSG00000134243	SORT1	2913.213996	3960.180677	-0.443065271	6.32E-06	0.000167
ENSG00000138821	SLC39A8	792.3552322	481.6242298	0.718236366	6.36E-06	0.000168
ENSG00000101160	CTSZ	598.5800963	907.0817792	-0.600104806	6.53E-06	0.000172
ENSG00000184371	CSF1	85.02461075	180.551158	-1.08641104	6.58E-06	0.000173
ENSG00000118985	ELL2	1042.572132	715.0650071	0.544308529	6.59E-06	0.000173
ENSG00000162496	DHRS3	26.27775214	85.86656746	-1.709593648	6.74E-06	0.000177
ENSG00000136856	SLC2A8	1142.086453	772.2364471	0.565512147	6.76E-06	0.000177
ENSG00000180758	GPR157	1470.247741	2152.549782	-0.550263101	6.78E-06	0.000177
ENSG00000085185	BCORL1	254.0899091	430.4690919	-0.760611852	6.82E-06	0.000178
ENSG00000114735	HEMK1	829.358449	1248.609004	-0.590588904	7.00E-06	0.000182

ENSG00000143515	ATP8B2	55.1388128	150.6142303	-1.450802247	7.02E-06	0.000183
ENSG00000187210	GCNT1	772.1433739	511.2323012	0.594648107	7.04E-06	0.000183
ENSG00000257337	AC068888.1	240.803478	389.2779266	-0.692760665	7.05E-06	0.000183
ENSG00000082438	COBLL1	919.6581746	634.9763417	0.533863166	7.16E-06	0.000185
ENSG00000128000	ZNF780B	143.6653611	251.9230134	-0.811825169	7.20E-06	0.000186
ENSG00000169302	STK32A	128.2584055	231.7137805	-0.854718128	7.23E-06	0.000186
ENSG00000275216	AL161431.1	1074.008186	1545.894922	-0.525392866	7.23E-06	0.000186
ENSG00000072422	RHOBTB1	99.61771405	196.0466222	-0.978204096	7.24E-06	0.000186
ENSG00000090238	YPEL3	237.4975263	467.9780436	-0.978339629	7.28E-06	0.000187
ENSG00000111801	BTN3A3	130.9006829	280.4738119	-1.100991159	7.34E-06	0.000188
ENSG00000239697	TNFSF12	109.5765175	207.6860638	-0.921948722	7.36E-06	0.000188
ENSG00000163110	PDLIM5	6108.73065	4528.970614	0.431607994	7.45E-06	0.00019
ENSG00000167081	PBX3	71.46490788	25.73888781	1.470117379	7.70E-06	0.000196
ENSG00000118515	SGK1	549.5522231	358.954731	0.613504072	8.10E-06	0.000206
ENSG00000135318	NT5E	2694.373204	4476.802385	-0.732714682	8.13E-06	0.000207
ENSG00000123080	CDKN2C	406.9818496	227.5678546	0.837592672	8.64E-06	0.000219
ENSG00000099804	CDC34	2405.654729	1682.716925	0.515995846	8.64E-06	0.000219
ENSG00000112294	ALDH5A1	1027.466198	1514.647957	-0.560009146	8.68E-06	0.000219
ENSG00000237609	AF064858.2	19.23955059	64.76175827	-1.749938167	8.68E-06	0.000219
ENSG00000105298	CACTIN	1151.084251	755.1641264	0.60829685	8.68E-06	0.000219
ENSG00000266208	AC080112.1	60.62318522	130.9900954	-1.113291381	8.76E-06	0.000221
ENSG00000138356	AOX1	26.36376028	3.370709862	2.991350789	8.82E-06	0.000222
ENSG00000145990	GFOD1	340.1558287	543.7624194	-0.675673729	8.85E-06	0.000222
ENSG00000077463	SIRT6	1242.57286	884.3095259	0.490726412	8.88E-06	0.000223
ENSG00000114812	VIPR1	750.6114898	1096.276948	-0.546318826	8.95E-06	0.000224
ENSG00000055118	KCNH2	208.1203792	109.3837823	0.925449506	8.99E-06	0.000225
ENSG00000111371	SLC38A1	7993.533137	11084.63887	-0.471683325	9.18E-06	0.000229
ENSG00000110400	NECTIN1	281.5368651	153.160669	0.877704238	9.26E-06	0.000231
ENSG00000187800	PEAR1	40.63120714	8.430662564	2.252234355	9.27E-06	0.000231
ENSG00000171940	ZNF217	1569.76207	2348.971162	-0.581752411	9.32E-06	0.000232
ENSG00000163032	VSNL1	252.9096379	413.0078767	-0.70903055	9.38E-06	0.000233
ENSG00000160867	FGFR4	77.16143786	156.599785	-1.020940713	9.45E-06	0.000234
ENSG00000081760	AACS	1860.560225	2545.414734	-0.452004284	9.46E-06	0.000234
ENSG00000105173	CCNE1	760.72789	468.9218466	0.699075762	9.87E-06	0.000244
ENSG00000162433	AK4	82.92045795	181.0537241	-1.128456102	9.93E-06	0.000245
ENSG00000161091	MFSD12	2103.369181	1403.656299	0.583942133	1.01E-05	0.000249
ENSG00000089060	SLC8B1	2006.619686	1444.372094	0.474756418	1.01E-05	0.000249
ENSG00000145362	ANK2	69.85868672	162.0048577	-1.216155692	1.02E-05	0.000251
ENSG00000163697	APBB2	3433.3847	4784.071442	-0.478751053	1.03E-05	0.000254
ENSG00000175787	ZNF169	398.1402061	659.0715604	-0.727850588	1.04E-05	0.000256
ENSG00000132256	TRIM5	895.5104829	1324.800489	-0.56574924	1.08E-05	0.000264
ENSG00000140750	ARHGAP17	1866.953232	1343.109227	0.475241391	1.09E-05	0.000266
ENSG00000184897	H1FX	1709.262537	2469.223728	-0.530692974	1.09E-05	0.000266

ENSG00000158470	B4GALT5	2625.113343	3555.420478	-0.437781127	1.10E-05	0.000267
ENSG00000031698	SARS	4421.017647	6884.919855	-0.639115431	1.10E-05	0.000268
ENSG00000168754	FAM178B	16.97690482	62.11540473	-1.86710046	1.11E-05	0.000269
ENSG00000153071	DAB2	194.2089051	91.1644006	1.090976404	1.15E-05	0.000278
ENSG00000133048	CHI3L1	262.3810666	469.1184767	-0.839451457	1.15E-05	0.000278
ENSG00000062716	VMP1	4271.39128	6418.764164	-0.587693587	1.15E-05	0.000279
ENSG00000052841	TTC17	2502.741542	3492.569256	-0.481010098	1.17E-05	0.000282
ENSG00000128272	ATF4	14158.50906	21969.25073	-0.633818356	1.19E-05	0.000286
ENSG00000116017	ARID3A	1858.459505	1339.24237	0.472568743	1.19E-05	0.000287
ENSG00000100605	ITPK1	3060.193276	2245.594291	0.446698654	1.19E-05	0.000287
ENSG00000156299	TIAM1	342.0015499	610.3873396	-0.836451967	1.20E-05	0.000287
ENSG00000141582	CBX4	2136.757001	3100.246752	-0.536829393	1.23E-05	0.000293
ENSG00000162458	FBLIM1	347.9089133	585.2991391	-0.751047457	1.23E-05	0.000295
ENSG00000133612	AGAP3	2824.274524	2089.025533	0.435173936	1.25E-05	0.000298
ENSG00000198258	UBL5	1674.348579	1077.830781	0.635898369	1.26E-05	0.000299
ENSG00000015413	DPEP1	201.0392699	351.4500187	-0.805726061	1.27E-05	0.000302
ENSG00000150593	PDCD4	1389.670013	2309.448021	-0.733030592	1.28E-05	0.000304
ENSG00000285410	GABPB1-IT1	286.3485821	451.5411667	-0.657252352	1.30E-05	0.000309
ENSG00000147041	SYTL5	100.9426517	224.9225043	-1.15669082	1.32E-05	0.000313
ENSG00000159314	ARHGAP27	4081.589113	5616.141945	-0.460449059	1.33E-05	0.000315
ENSG00000151150	ANK3	677.7415918	1129.011809	-0.736611924	1.36E-05	0.000322
ENSG00000173457	PPP1R14B	5023.287354	3258.368592	0.624619499	1.36E-05	0.000322
ENSG00000161791	FMNL3	463.6723838	795.7273996	-0.779842512	1.40E-05	0.000329
ENSG00000099821	POLRMT	3842.227252	2892.05461	0.40981722	1.41E-05	0.000333
ENSG00000088992	TESC	141.7929038	250.9507737	-0.82348088	1.43E-05	0.000336
ENSG00000159337	PLA2G4D	21.47388454	76.04285824	-1.82480843	1.45E-05	0.000341
ENSG00000130810	PPAN	244.9044544	138.1493222	0.826362989	1.45E-05	0.000341
ENSG00000237975	FLG-AS1	83.48082803	33.07169481	1.330729982	1.46E-05	0.000341
ENSG00000182489	XKRX	41.13755036	103.9276311	-1.338410478	1.46E-05	0.000342
ENSG00000260328	AC104024.2	141.7103136	268.8955106	-0.923877956	1.46E-05	0.000342
ENSG00000103811	CTSH	624.691174	949.6003814	-0.604633117	1.47E-05	0.000342
ENSG00000184454	NCMAP	33.92713263	91.80229427	-1.437387004	1.48E-05	0.000345
ENSG00000162734	PEA15	1779.192766	1188.252369	0.582561181	1.50E-05	0.000348
ENSG00000063438	AHRR	280.0980663	462.4786315	-0.723209706	1.50E-05	0.000348
ENSG00000232762	AL355483.3	100.1239164	38.78550005	1.372031156	1.51E-05	0.000351
ENSG00000280587	LINC01348	60.23717665	131.9776526	-1.133332754	1.52E-05	0.000352
ENSG00000267968	AC011523.1	5.090403745	32.77292133	-2.684900151	1.53E-05	0.000355
ENSG00000279145	AC011912.1	34.85448379	86.61914315	-1.317062312	1.54E-05	0.000355
ENSG00000164889	SLC4A2	6031.808202	4364.966621	0.466768877	1.55E-05	0.000356
ENSG00000146909	NOM1	1818.097316	1252.856609	0.537689877	1.55E-05	0.000357
ENSG00000159720	ATP6V0D1	2358.008038	1601.132431	0.558573751	1.56E-05	0.000359
ENSG00000179111	HES7	219.6393297	108.4992273	1.020555817	1.57E-05	0.00036
ENSG00000189306	RRP7A	1825.861525	1161.423226	0.653081183	1.57E-05	0.00036

ENSG00000025423	HSD17B6	63.64040381	22.42716363	1.506532249	1.60E-05	0.000365
ENSG00000137267	TUBB2A	258.2342448	125.8245193	1.037383383	1.62E-05	0.000369
ENSG00000128512	DOCK4	459.8202206	260.9896926	0.817179829	1.65E-05	0.000375
ENSG00000126254	RBM42	2665.177535	1880.105489	0.503564589	1.70E-05	0.000387
ENSG00000151632	AKR1C2	54.72688929	14.63981204	1.902633193	1.75E-05	0.000398
ENSG00000197249	SERPINA1	61.37724753	161.9681944	-1.404375996	1.75E-05	0.000398
ENSG00000167123	CERCAM	38.0618734	96.59920597	-1.340937783	1.76E-05	0.0004
ENSG00000285933	AP003498.2	2.529592265	26.27128941	-3.372308834	1.77E-05	0.000401
ENSG00000154556	SORBS2	796.0733449	1220.276233	-0.61689109	1.77E-05	0.000401
ENSG00000171798	KNDC1	37.25521915	95.84198571	-1.364744719	1.78E-05	0.000402
ENSG00000196458	ZNF605	132.9863704	246.7459459	-0.890757013	1.80E-05	0.000406
ENSG00000130164	LDLR	7114.094181	5180.870987	0.457615778	1.80E-05	0.000406
ENSG00000140416	TPM1	4690.112957	6320.583826	-0.430427654	1.82E-05	0.000409
ENSG00000269516	CYP4F23P	45.42257242	115.5966741	-1.346776	1.82E-05	0.00041
ENSG00000186188	FFAR4	22.32502239	67.34857218	-1.593250922	1.84E-05	0.000413
ENSG00000134440	NARS	6595.496198	8981.657886	-0.445596298	1.84E-05	0.000413
ENSG00000096696	DSP	9639.067331	13042.5707	-0.436303567	1.85E-05	0.000414
ENSG00000184113	CLDN5	143.7228058	69.04097481	1.059482823	1.88E-05	0.00042
ENSG00000166986	MARS	6733.118116	9625.293481	-0.515575734	1.89E-05	0.000421
ENSG00000165272	AQP3	103.3918507	201.7210269	-0.966504996	1.89E-05	0.000421
ENSG00000134716	CYP2J2	1375.230586	1986.955508	-0.531277619	1.90E-05	0.000423
ENSG00000181938	GINS3	688.4834638	404.4257239	0.767807477	1.90E-05	0.000423
ENSG00000100234	TIMP3	60.82080584	142.5600954	-1.228376992	1.96E-05	0.000434
ENSG00000131067	GGT7	10.86605443	47.03559465	-2.113403339	1.96E-05	0.000435
ENSG00000184349	EFNA5	473.2039271	309.4464201	0.612742261	1.97E-05	0.000435
ENSG00000062038	CDH3	1672.368668	2311.260587	-0.467117187	1.97E-05	0.000436
ENSG00000111885	MAN1A1	5309.998302	3907.524602	0.442269312	1.97E-05	0.000436
ENSG00000100297	MCM5	5325.763597	3369.96779	0.660361565	1.99E-05	0.000438
ENSG00000186868	MAPT	55.02245821	121.3660139	-1.143201971	2.04E-05	0.000449
ENSG00000137876	RSL24D1	3229.264893	4632.695995	-0.520791672	2.05E-05	0.00045
ENSG00000183734	ASCL2	3276.924438	4548.307042	-0.472985283	2.05E-05	0.000451
ENSG00000086548	CEACAM6	66.49122099	21.56248504	1.61213954	2.08E-05	0.000456
ENSG00000111912	NCOA7	732.1721716	1055.13609	-0.527523126	2.14E-05	0.000469
ENSG00000133816	MICAL2	3841.844026	6217.539847	-0.694617591	2.16E-05	0.000473
ENSG00000105677	TMEM147	2211.888455	1553.758137	0.50969218	2.19E-05	0.000479
ENSG00000139405	RITA1	1428.282925	1007.687902	0.50318295	2.22E-05	0.000485
ENSG00000189159	JPT1	3700.028666	2389.769872	0.630750467	2.22E-05	0.000485
ENSG00000107105	ELAVL2	15.85484043	61.2240219	-1.944481656	2.28E-05	0.000496
ENSG00000144821	MYH15	222.1330713	120.6405669	0.877148368	2.29E-05	0.000498
ENSG00000074410	CA12	2.14939847	24.49667644	-3.490449382	2.31E-05	0.0005
ENSG00000173868	PHOSPHO1	46.03956615	111.5568227	-1.275705293	2.31E-05	0.0005
ENSG00000149927	DOC2A	91.27829283	190.2194616	-1.060145949	2.33E-05	0.000504
ENSG00000155629	PIK3AP1	109.2647393	41.54260308	1.397591947	2.35E-05	0.000507

ENSG00000166002	SMCO4	226.3205074	114.0938391	0.98886919	2.35E-05	0.000507
ENSG00000124588	NQO2	1217.402435	845.9313217	0.525288068	2.35E-05	0.000507
ENSG00000100577	GSTZ1	448.4826975	671.8039807	-0.582614923	2.36E-05	0.000507
ENSG00000130382	MLLT1	3834.125435	2761.302233	0.473667449	2.36E-05	0.000507
ENSG00000277363	SRCIN1	54.5136074	156.0185606	-1.516958827	2.38E-05	0.00051
ENSG00000105520	PLPPR2	376.802828	230.3689438	0.708956523	2.39E-05	0.000511
ENSG00000196517	SLC6A9	232.2660665	875.7882457	-1.914792678	2.39E-05	0.000512
ENSG00000261115	TMEM178B	713.8377267	485.1109144	0.556759042	2.43E-05	0.00052
ENSG00000089289	IGBP1	999.8577899	1485.660296	-0.571543893	2.44E-05	0.00052
ENSG00000198467	TPM2	19.53588157	65.0834937	-1.732976079	2.44E-05	0.00052
ENSG00000181885	CLDN7	5154.722216	7584.617349	-0.557143237	2.44E-05	0.00052
ENSG00000179715	PCED1B	170.2819307	319.2072111	-0.906009811	2.44E-05	0.00052
ENSG00000111886	GABRR2	108.3629408	209.8431585	-0.94993315	2.45E-05	0.000521
ENSG00000103966	EHD4	3126.676233	2060.54456	0.601789614	2.50E-05	0.000531
ENSG00000204588	LINC01123	95.85388276	185.8595327	-0.954125909	2.52E-05	0.000534
ENSG00000147162	OGT	7780.533709	11280.29368	-0.535925794	2.53E-05	0.000535
ENSG00000223768	LINC00205	771.1536575	1273.62752	-0.724172218	2.55E-05	0.000538
ENSG00000128311	TST	1266.884438	1870.609584	-0.562533461	2.56E-05	0.000541
ENSG00000103528	SYT17	172.4705085	323.3843563	-0.906349708	2.57E-05	0.000542
ENSG00000184216	IRAK1	4451.171969	3180.509507	0.485104093	2.62E-05	0.000552
ENSG00000136603	SKIL	763.1627305	1131.65245	-0.568581748	2.69E-05	0.000565
ENSG00000139651	ZNF740	1272.74967	1755.537608	-0.463781978	2.70E-05	0.000566
ENSG00000128510	CPA4	62.62134043	13.76794012	2.177871198	2.72E-05	0.000571
ENSG00000122778	KIAA1549	888.1114773	620.9545772	0.516502657	2.76E-05	0.000577
ENSG00000121380	BCL2L14	157.0657499	274.7614475	-0.808756362	2.79E-05	0.000583
ENSG00000125772	GPCPD1	499.4817168	771.3470898	-0.626974058	2.81E-05	0.000586
ENSG00000101104	PABPC1L	2074.532071	3195.56657	-0.623177138	2.81E-05	0.000587
ENSG00000160613	PCSK7	1903.854276	1409.402285	0.43399189	2.83E-05	0.000589
ENSG00000129932	DOHH	587.2366317	356.7055371	0.719771058	2.83E-05	0.000589
ENSG00000120925	RNF170	736.6534901	1103.85267	-0.583765811	2.84E-05	0.00059
ENSG00000083444	PLOD1	3793.311676	2636.59014	0.524673301	2.90E-05	0.000603
ENSG00000138193	PLCE1	728.0181001	486.6102431	0.579916413	2.94E-05	0.000609
ENSG00000111647	UHRF1BP1L	901.4825782	632.8891814	0.51069716	2.95E-05	0.000611
ENSG00000118655	DCLRE1B	771.8327717	473.2559012	0.706074882	2.96E-05	0.000613
ENSG00000227124	ZNF717	241.920438	402.49605	-0.733445274	2.97E-05	0.000614
ENSG00000106344	RBM28	3021.664431	2194.312157	0.461687493	2.98E-05	0.000614
ENSG00000125124	BBS2	682.8892921	1084.934778	-0.668269026	2.99E-05	0.000615
ENSG00000091136	LAMB1	3562.611789	4826.909956	-0.438307208	2.99E-05	0.000615
ENSG00000109794	FAM149A	20.04493228	60.88099772	-1.603337163	3.00E-05	0.000617
ENSG00000136628	EPRS	10583.65222	14539.99431	-0.458244859	3.02E-05	0.000621
ENSG00000187051	RPS19BP1	1697.55149	1128.435518	0.589493852	3.04E-05	0.000623
ENSG00000187742	SECISBP2	1510.693601	2123.791557	-0.491677056	3.07E-05	0.000629
ENSG00000104774	MAN2B1	1038.313232	1455.471785	-0.487299265	3.10E-05	0.000635

ENSG00000244879	GABPB1-AS1	2463.11788	3556.521374	-0.529924611	3.17E-05	0.000648
ENSG00000225138	SLC9A3-AS1	1193.628139	1729.431768	-0.534344751	3.18E-05	0.000648
ENSG00000162413	KLHL21	1451.369674	1036.956457	0.485245113	3.19E-05	0.000649
ENSG00000175147	TMEM51-AS1	51.2090906	117.444745	-1.193793895	3.19E-05	0.00065
ENSG00000123815	COQ8B	770.0021496	1103.58129	-0.520121878	3.20E-05	0.00065
ENSG00000243147	MRPL33	921.9286323	638.8426431	0.52933427	3.22E-05	0.000653
ENSG00000162194	LBHD1	221.6305975	351.3488782	-0.664822762	3.22E-05	0.000653
ENSG00000084207	GSTP1	14521.60162	10879.47194	0.416576507	3.27E-05	0.000662
ENSG00000261824	LINC00662	238.8966943	380.5448796	-0.671449354	3.28E-05	0.000664
ENSG00000233461	AL445524.1	172.1518428	92.4623065	0.89772812	3.30E-05	0.000665
ENSG00000141524	TMC6	3497.146705	5092.97622	-0.542276117	3.30E-05	0.000665
ENSG00000198182	ZNF607	216.7216426	342.8850319	-0.661306976	3.30E-05	0.000665
ENSG00000177465	ACOT4	9.797706306	39.61367524	-2.012531472	3.34E-05	0.000672
ENSG00000100379	KCTD17	464.8840365	674.3943484	-0.537263345	3.34E-05	0.000672
ENSG00000140682	TGFB1I1	26.91035548	76.17914697	-1.49919475	3.39E-05	0.00068
ENSG00000181634	TNFSF15	1133.917843	1744.239706	-0.621403093	3.50E-05	0.000702
ENSG00000215244	AL137145.2	7.245616993	35.11195463	-2.270847865	3.51E-05	0.000702
ENSG00000125746	EML2	1316.875285	1998.647239	-0.601840334	3.53E-05	0.000706
ENSG00000114446	IFT57	426.3944677	673.8811266	-0.661222686	3.59E-05	0.000717
ENSG00000114779	ABHD14B	813.5656774	1187.121408	-0.545651403	3.66E-05	0.00073
ENSG00000121741	ZMYM2	2819.146135	4187.048398	-0.570631708	3.69E-05	0.000735
ENSG00000077782	FGFR1	970.5161527	1354.853399	-0.480868256	3.74E-05	0.000744
ENSG00000116991	SIPA1L2	465.0406948	695.8556091	-0.582663166	3.75E-05	0.000746
ENSG00000110047	EHD1	2990.716432	2154.347248	0.473260271	3.80E-05	0.000754
ENSG00000105639	JAK3	31.12952122	89.8805883	-1.532805034	3.83E-05	0.000759
ENSG00000158526	TSR2	903.7406067	583.8514768	0.630735021	3.83E-05	0.000759
ENSG00000099256	PRTFDC1	622.4408306	967.0352901	-0.635981916	3.84E-05	0.00076
ENSG00000175309	PHYKPL	1482.990811	2406.281007	-0.698252566	3.85E-05	0.000761
ENSG00000178573	MAF	42.8813506	110.9405643	-1.369200815	3.88E-05	0.000765
ENSG00000223823	LINC01342	35.00925564	93.53958717	-1.417726573	3.94E-05	0.000777
ENSG00000197142	ACSL5	2858.770617	4046.219963	-0.501374847	4.00E-05	0.000786
ENSG00000166173	LARP6	8.361866604	38.89354088	-2.213516241	4.03E-05	0.000792
ENSG00000130005	GAMT	73.31216059	145.1480761	-0.985084454	4.04E-05	0.000794
ENSG00000065000	AP3D1	10317.16424	7955.791009	0.374984008	4.09E-05	0.000802
ENSG00000148346	LCN2	29.23543968	94.66040469	-1.695851116	4.10E-05	0.000802
ENSG00000228437	LINC02474	363.2636251	676.110566	-0.896015719	4.11E-05	0.000805
ENSG00000134684	YARS	6814.794428	9219.385259	-0.436010554	4.14E-05	0.000809
ENSG00000163516	ANKZF1	1136.393978	1704.050648	-0.584608586	4.18E-05	0.000815
ENSG00000075643	MOCOS	1287.88962	1808.375553	-0.489782343	4.18E-05	0.000815
ENSG00000119812	FAM98A	3143.81709	2026.247344	0.633793091	4.18E-05	0.000815
ENSG00000148143	ZNF462	214.1452731	119.3464889	0.845113406	4.23E-05	0.000822
ENSG00000197558	SSPO	17.28195796	71.03875996	-2.032775629	4.23E-05	0.000822
ENSG00000163083	INHBB	614.4633378	1077.205255	-0.809949815	4.24E-05	0.000822

ENSG00000148344	PTGES	239.1626197	382.2933726	-0.677694909	4.24E-05	0.000822
ENSG00000184584	TMEM173	646.6159098	400.2860722	0.690213094	4.26E-05	0.000824
ENSG00000165244	ZNF367	1314.043885	808.9022635	0.700341807	4.30E-05	0.000831
ENSG00000143643	TTC13	2002.355156	1474.60809	0.441176969	4.33E-05	0.000836
ENSG00000139083	ETV6	780.6414954	1154.012915	-0.564379424	4.34E-05	0.000836
ENSG00000188322	SBK1	321.3307984	202.5260345	0.666277881	4.34E-05	0.000837
ENSG00000197780	TAF13	797.1255427	548.0694196	0.540539907	4.36E-05	0.000839
ENSG00000170464	DNAJC18	72.02894741	149.5005033	-1.051957334	4.47E-05	0.000859
ENSG00000162302	RPS6KA4	2653.972892	1784.464469	0.572951406	4.51E-05	0.000865
ENSG00000072818	ACAP1	104.2352874	206.2865947	-0.98328869	4.51E-05	0.000865
ENSG00000204634	TBC1D8	925.773753	1368.621926	-0.56406679	4.56E-05	0.000873
ENSG00000153064	BANK1	23.26160621	72.08373246	-1.633656281	4.58E-05	0.000876
ENSG00000034510	TMSB10	26403.56258	20593.56136	0.358549349	4.61E-05	0.000881
ENSG00000117682	DHDDS	1402.843461	1007.901014	0.477305093	4.62E-05	0.000881
ENSG00000130517	PGPEP1	793.7095919	1135.479436	-0.516340837	4.63E-05	0.000883
ENSG00000079156	OSBPL6	116.1104104	212.5974958	-0.871168521	4.65E-05	0.000885
ENSG00000073910	FRY	29.00552718	5.133644827	2.506972919	4.67E-05	0.000887
ENSG00000135362	PRR5L	505.8175303	762.9932239	-0.593649603	4.67E-05	0.000887
ENSG00000198520	ARMH1	68.60776542	147.1541168	-1.100998014	4.68E-05	0.000887
ENSG00000143303	RRNAD1	650.1794646	953.6520535	-0.552667318	4.68E-05	0.000888
ENSG00000178425	NT5DC1	1295.646212	1844.32192	-0.509514334	4.70E-05	0.000889
ENSG00000134294	SLC38A2	7196.690319	10610.93623	-0.56012773	4.73E-05	0.000895
ENSG00000180773	SLC36A4	382.0826407	237.3809194	0.686380338	4.79E-05	0.000905
ENSG00000134291	TMEM106C	4315.70752	2961.283968	0.543394099	4.86E-05	0.000917
ENSG00000275410	HNF1B	512.0885749	742.1413784	-0.535047484	4.92E-05	0.000927
ENSG00000079257	LXN	60.76234746	123.7048588	-1.02533604	4.94E-05	0.000929
ENSG00000124882	EREG	21870.57285	29400.71017	-0.426862515	4.97E-05	0.000934
ENSG00000002745	WNT16	61.32327971	18.71972064	1.718564722	5.08E-05	0.000953
ENSG00000180336	MEIOC	125.5166557	63.14201412	0.992684516	5.14E-05	0.000964
ENSG00000006468	ETV1	203.7504217	331.1674592	-0.701851975	5.19E-05	0.000972
ENSG00000105662	CRTC1	464.5024669	691.05628	-0.573726022	5.24E-05	0.00098
ENSG00000187017	ESPN	89.82995081	412.0366955	-2.19692054	5.25E-05	0.000982
ENSG00000173221	GLRX	503.4811862	722.9679566	-0.522336715	5.27E-05	0.000984
ENSG00000089195	TRMT6	1812.338717	1288.059252	0.492391826	5.28E-05	0.000984
ENSG00000198668	CALM1	16215.47818	11047.55744	0.553644707	5.31E-05	0.000989
ENSG00000128626	MRPS12	1507.848124	997.0888756	0.596901925	5.32E-05	0.000989
ENSG00000120686	UFM1	2470.534254	1846.098145	0.420221255	5.36E-05	0.000996
ENSG00000100889	PCK2	1652.400877	5607.500407	-1.762777021	5.46E-05	0.001013
ENSG00000196659	TTC30B	130.8538541	230.5127913	-0.818210028	5.51E-05	0.001022
ENSG00000079112	CDH17	5338.666531	3570.861683	0.580019192	5.52E-05	0.001022
ENSG00000122952	ZWINT	4020.910307	2433.774626	0.724375669	5.59E-05	0.001034
ENSG00000167103	PIP5KL1	61.84232514	141.3256313	-1.193052109	5.60E-05	0.001034
ENSG00000089685	BIRC5	4349.05802	2665.835563	0.70619673	5.62E-05	0.001037

ENSG00000167130	DOLPP1	815.9163874	571.1811164	0.513931016	5.66E-05	0.001044
ENSG00000175984	DENND2C	175.3414775	90.58039509	0.957316752	5.69E-05	0.001047
ENSG00000138759	FRAS1	4407.230226	5938.887141	-0.430334598	5.71E-05	0.00105
ENSG00000104231	ZFAND1	897.7031299	1364.758059	-0.604880652	5.75E-05	0.001054
ENSG00000157214	STEAP2	473.351242	751.1832154	-0.667346223	5.75E-05	0.001054
ENSG00000096092	TMEM14A	1199.171147	823.5480604	0.542031899	5.75E-05	0.001054
ENSG00000203783	PRR9	153.9507454	71.72167706	1.099179409	5.86E-05	0.001073
ENSG00000114120	SLC25A36	3900.605919	5789.538765	-0.569740189	5.89E-05	0.001076
ENSG00000116990	MYCL	189.4557357	313.728926	-0.727134741	5.89E-05	0.001076
ENSG00000137275	RIPK1	1307.98661	931.692816	0.48912989	5.90E-05	0.001076
ENSG00000164294	GPX8	628.5048522	879.3105405	-0.484858255	5.91E-05	0.001076
ENSG00000108106	UBE2S	5315.587013	3700.704787	0.522485307	5.91E-05	0.001076
ENSG00000111674	ENO2	185.1254089	355.4636133	-0.940946475	5.99E-05	0.001089
ENSG00000254858	MPV17L2	735.4364579	454.0118288	0.697291131	6.03E-05	0.001095
ENSG00000197451	HNRNPAB	12136.33683	7762.908173	0.644724889	6.07E-05	0.001102
ENSG00000103257	SLC7A5	19687.23006	25667.76071	-0.382688494	6.08E-05	0.001102
ENSG00000146416	AIG1	1675.011027	2451.670296	-0.549877657	6.13E-05	0.001111
ENSG00000175198	PCCA	1334.201689	1843.024943	-0.466299821	6.18E-05	0.001118
ENSG00000007080	CCDC124	2028.985537	1385.779359	0.550497986	6.23E-05	0.001126
ENSG00000164897	TMUB1	1858.611421	1297.157354	0.518631867	6.27E-05	0.001131
ENSG00000115866	DARS	2610.022573	3493.116931	-0.420698567	6.27E-05	0.001131
ENSG00000088280	ASAP3	136.2601194	255.4749765	-0.907962209	6.28E-05	0.001131
ENSG00000184465	WDR27	947.4245657	1315.981177	-0.473418749	6.28E-05	0.001131
ENSG00000184489	PTP4A3	5.894712769	34.54727159	-2.557587076	6.30E-05	0.001132
ENSG00000148677	ANKRD1	83.51021252	174.4004289	-1.066584684	6.32E-05	0.001134
ENSG00000151914	DST	4900.950082	3729.084916	0.394172444	6.35E-05	0.00114
ENSG00000091536	MYO15A	6.303218391	35.14585219	-2.493021049	6.39E-05	0.001145
ENSG00000124391	IL17C	18.6559214	54.07252525	-1.535553654	6.40E-05	0.001146
ENSG00000164442	CITED2	3434.446979	2445.327751	0.489847319	6.48E-05	0.001157
ENSG00000182871	COL18A1	58.67462536	143.1076832	-1.285753407	6.50E-05	0.00116
ENSG00000170915	PAQR8	39.01064894	105.3709264	-1.435430474	6.50E-05	0.00116
ENSG00000115350	POLE4	773.134206	523.1025441	0.564705235	6.52E-05	0.001162
ENSG00000163053	SLC16A14	148.4758055	269.4003057	-0.861472376	6.63E-05	0.00118
ENSG00000185875	THNSL1	543.056999	769.0335405	-0.502728824	6.64E-05	0.001181
ENSG00000135480	KRT7	80.31332814	174.8712218	-1.124299678	6.68E-05	0.001185
ENSG00000242498	ARPIN	828.8070453	1207.668124	-0.54351897	6.70E-05	0.001188
ENSG00000149639	SOGA1	576.9714294	347.3422087	0.732315062	6.73E-05	0.001192
ENSG00000154079	SDHAF4	281.7920713	433.1310981	-0.621187997	6.73E-05	0.001192
ENSG00000144290	SLC4A10	22.96344051	3.178623784	2.828312374	6.75E-05	0.001193
ENSG00000057704	TMCC3	81.62158349	189.0569974	-1.210404202	6.76E-05	0.001194
ENSG00000184678	HIST2H2BE	142.8880913	70.42048001	1.017613685	6.77E-05	0.001194
ENSG00000138764	CCNG2	730.7873266	1201.335253	-0.717276761	6.78E-05	0.001195
ENSG00000066923	STAG3	208.0939021	332.8211294	-0.679018647	6.84E-05	0.001204

ENSG00000108509	CAMTA2	767.8679468	1090.089988	-0.505851561	6.87E-05	0.001206
ENSG00000064666	CNN2	9403.42135	7177.516428	0.389709419	6.87E-05	0.001206
ENSG00000196502	SULT1A1	344.1310039	522.9105608	-0.602335629	6.89E-05	0.00121
ENSG00000165406	8-Mar	478.6140692	311.6618478	0.617029486	6.91E-05	0.00121
ENSG00000104853	CLPTM1	3715.759059	2713.313087	0.453575531	6.92E-05	0.001212
ENSG00000181220	ZNF746	1275.885454	941.6091476	0.438317826	7.10E-05	0.001242
ENSG00000196730	DAPK1	639.727479	936.170585	-0.5495336	7.13E-05	0.001247
ENSG00000146918	NCAPG2	5657.553166	3883.617531	0.542716962	7.15E-05	0.001248
ENSG00000169230	PRELID1	2623.321387	1673.292855	0.648968962	7.52E-05	0.00131
ENSG00000130725	UBE2M	2854.732802	2074.813913	0.460551703	7.53E-05	0.001312
ENSG00000173227	SYT12	338.6230027	535.5324336	-0.660889514	7.59E-05	0.00132
ENSG00000151176	PLBD2	2317.972616	1766.122384	0.39218173	7.60E-05	0.00132
ENSG00000197653	DNAH10	109.309571	51.46622898	1.086986812	7.60E-05	0.00132
ENSG00000116922	C1orf109	1161.805425	818.4784512	0.505439795	7.68E-05	0.001331
ENSG00000149260	CAPN5	367.7964554	1422.300197	-1.951254878	7.70E-05	0.001334
ENSG00000167306	MYO5B	4231.391551	5843.042942	-0.465661044	7.74E-05	0.001339
ENSG00000135114	OASL	57.38982902	19.52238511	1.543954967	7.79E-05	0.001347
ENSG00000160446	ZDHHC12	1275.90679	899.7118156	0.504013801	7.80E-05	0.001348
ENSG00000172922	RNASEH2C	1610.248499	1080.421308	0.576140608	7.87E-05	0.001357
ENSG00000225648	SBDSP1	682.7857129	458.1205546	0.575827097	7.89E-05	0.001359
ENSG00000232354	VIPR1-AS1	94.83154973	176.9353762	-0.898699121	7.91E-05	0.001362
ENSG00000105726	ATP13A1	3156.649178	2374.407255	0.410885591	8.04E-05	0.001382
ENSG00000006756	ARSD	692.7428009	1205.357876	-0.799378682	8.04E-05	0.001382
ENSG00000124177	CHD6	1962.289727	2621.709968	-0.418191343	8.06E-05	0.001383
ENSG00000005007	UPF1	6527.265745	4848.532315	0.429012227	8.07E-05	0.001383
ENSG00000099624	ATP5F1D	4167.33672	2954.509191	0.496439233	8.15E-05	0.001396
ENSG00000102781	KATNAL1	280.6764946	171.5120146	0.711633769	8.16E-05	0.001396
ENSG00000188959	C9orf152	953.2532007	1452.27354	-0.607777109	8.18E-05	0.001397
ENSG00000162714	ZNF496	1007.907619	1385.769398	-0.459490535	8.19E-05	0.001398
ENSG00000109066	TMEM104	620.0147499	418.7793998	0.567023983	8.22E-05	0.001401
ENSG00000104880	ARHGEF18	261.6654215	160.8086577	0.704963426	8.24E-05	0.001401
ENSG00000276256	AC011043.1	289.2946991	174.8015193	0.727549572	8.24E-05	0.001401
ENSG00000119950	MXI1	723.6768918	1174.354336	-0.698175256	8.25E-05	0.001401
ENSG00000231483	AL365356.4	11.02031578	40.46032769	-1.882550717	8.25E-05	0.001401
ENSG00000166619	BLCAP	2355.078182	1755.184261	0.424093325	8.34E-05	0.001414
ENSG00000181852	RNF41	1249.39451	1678.250909	-0.425857133	8.36E-05	0.001417
ENSG00000129355	CDKN2D	298.6746081	164.1550522	0.865035852	8.50E-05	0.001439
ENSG00000105011	ASF1B	2411.17976	1458.0546	0.72592061	8.67E-05	0.001466
ENSG00000183684	ALYREF	3836.346837	2422.95216	0.663049098	8.70E-05	0.00147
ENSG00000090266	NDUFB2	1497.251347	1053.521196	0.507643025	8.72E-05	0.001472
ENSG00000120217	CD274	169.9830546	89.54698642	0.919577274	8.74E-05	0.001474
ENSG00000112739	PRPF4B	2555.325779	1880.224511	0.442617692	8.77E-05	0.001477
ENSG00000237686	AL109615.3	16.54462959	50.78689427	-1.623136439	8.78E-05	0.001477

ENSG00000079215	SLC1A3	282.1134069	64.61897468	2.128681466	8.80E-05	0.001478
ENSG00000162616	DNAJB4	871.8371819	603.063877	0.531315492	8.81E-05	0.001479
ENSG00000111961	SASH1	719.743152	508.8758813	0.500183729	8.85E-05	0.001484
ENSG00000021776	AQR	1252.479944	1732.862307	-0.46877434	8.87E-05	0.001486
ENSG00000137501	SYTL2	1890.34766	2650.30287	-0.487932034	9.04E-05	0.001513
ENSG00000175772	LINC01106	114.128486	204.4148766	-0.838929819	9.06E-05	0.001516
ENSG00000223745	CCDC18-AS1	308.5120417	567.6752506	-0.879632113	9.08E-05	0.001517
ENSG00000146094	DOK3	549.421294	314.1440851	0.807790604	9.11E-05	0.001521
ENSG00000198774	RASSF9	298.1862572	521.8391032	-0.807294413	9.21E-05	0.001536
ENSG00000151778	SERP2	212.099786	330.485313	-0.639722241	9.27E-05	0.001544
ENSG00000124541	RRP36	1621.743901	1109.340248	0.54806387	9.28E-05	0.001544
ENSG00000204147	ASAH2B	109.1339369	192.4803467	-0.821424569	9.34E-05	0.001552
ENSG00000134262	AP4B1	640.1683872	443.7688146	0.528648091	9.38E-05	0.001557
ENSG00000041802	LSG1	2655.896543	1864.586876	0.510395026	9.47E-05	0.00157
ENSG00000251169	LINC01843	54.49218301	119.1558305	-1.129322062	9.48E-05	0.001571
ENSG00000169221	TBC1D10B	2620.464741	1946.051854	0.429407229	9.49E-05	0.001571
ENSG00000143320	CRABP2	30.64296756	73.46180997	-1.261212395	9.53E-05	0.001577
ENSG00000197183	NOL4L	42.44453315	98.84638966	-1.21781273	9.56E-05	0.00158
ENSG00000125505	MBOAT7	4208.611419	3109.278252	0.436702632	9.79E-05	0.001615
ENSG00000198435	NRARP	383.0122854	561.6764168	-0.551101071	9.84E-05	0.001623
ENSG00000162344	FGF19	8.100734891	33.69281477	-2.062378326	9.89E-05	0.001629
ENSG00000170266	GLB1	2093.872136	2870.914434	-0.455550278	9.93E-05	0.001632
ENSG00000136159	NUDT15	2393.654551	1798.801069	0.411979913	9.93E-05	0.001632
ENSG00000172493	AFF1	2641.65832	3513.988963	-0.411799781	0.000100072	0.001643
ENSG00000104522	TSTA3	1868.683693	1311.164174	0.511436501	0.000100489	0.001648
ENSG00000235888	AF064858.1	16.40199779	50.57229739	-1.625008162	0.000101233	0.001659
ENSG00000144043	TEX261	3383.870543	2332.165315	0.53707056	0.000101541	0.001662
ENSG00000008405	CRY1	796.9770371	556.2089359	0.520068646	0.000101636	0.001662
ENSG00000147454	SLC25A37	5377.374538	7431.635333	-0.466878889	0.000101783	0.001663
ENSG00000101220	C20orf27	1540.634986	966.382686	0.673194311	0.0001022	0.001668
ENSG00000119559	C19orf25	1454.366711	976.3413441	0.575102526	0.000102798	0.001676
ENSG00000162512	SDC3	735.932844	529.3327064	0.474940745	0.000103194	0.001681
ENSG00000173210	ABLIM3	62.59873268	23.59900257	1.409888951	0.000103884	0.00169
ENSG00000162104	ADCY9	247.4448035	152.8261916	0.695463517	0.000104259	0.001695
ENSG00000187741	FANCA	2232.035495	1519.8676	0.554927368	0.000105215	0.001709
ENSG00000153233	PTPRR	70.39763242	134.2870103	-0.930641239	0.000106684	0.001731
ENSG00000184012	TMPRSS2	775.8735891	1127.879108	-0.540075451	0.000106954	0.001734
ENSG00000148362	PAXX	1705.288588	1105.972699	0.624941293	0.000107192	0.001736
ENSG00000167964	RAB26	480.3142888	761.26475	-0.664157298	0.000107565	0.00174
ENSG00000111639	MRPL51	2131.683315	1461.127567	0.545273723	0.000108258	0.001749
ENSG00000218186	KRT8P43	27.13338053	4.934205526	2.443287002	0.000108891	0.001758
ENSG00000183484	GPR132	15.64849764	53.23067726	-1.763551622	0.000109231	0.001761
ENSG00000262188	LINC01978	24.12651894	68.93516114	-1.516476237	0.000109321	0.001761

ENSG00000198496	NBR2	123.4333134	229.4752292	-0.892848885	0.000109664	0.001765
ENSG00000049130	KITLG	1824.053564	1255.108907	0.539329516	0.000110252	0.001773
ENSG00000117461	PIK3R3	263.594754	164.4084824	0.680985138	0.000111056	0.001784
ENSG00000164111	ANXA5	7063.632475	5145.324513	0.457097819	0.000111237	0.001785
ENSG00000105397	TYK2	3431.119206	2528.310657	0.44069212	0.00011174	0.001792
ENSG00000106615	RHEB	2845.860073	2154.218442	0.401646247	0.000112507	0.001802
ENSG00000163121	NEURL3	23.99766203	64.97261286	-1.435379089	0.000112612	0.001802
ENSG00000099984	GSTT2	11.28726227	40.78206313	-1.85306307	0.000112906	0.001805
ENSG00000254670	AC084859.1	15.03839135	47.68209396	-1.669077187	0.000113499	0.001812
ENSG00000162777	DENND2D	165.4966908	269.4462867	-0.703264864	0.000113585	0.001812
ENSG00000213621	RPSAP54	71.35533003	26.81784351	1.412911705	0.000114018	0.001816
ENSG00000103404	USP31	763.1007724	496.7487531	0.620779518	0.00011402	0.001816
ENSG00000101945	SUV39H1	585.3585522	360.5217366	0.699179169	0.000114865	0.001827
ENSG00000100280	AP1B1	4118.198951	2989.119242	0.462375682	0.0001174	0.001866
ENSG00000049541	RFC2	1363.258371	910.8286852	0.58205369	0.000117767	0.00187
ENSG00000128567	PODXL	6942.414642	5343.140565	0.377664666	0.000118218	0.001874
ENSG00000108523	RNF167	2650.769594	1982.015752	0.419235608	0.000118243	0.001874
ENSG00000213366	GSTM2	85.16744245	158.5443216	-0.895360517	0.000119236	0.001888
ENSG00000249087	ZNF436-AS1	107.7633914	190.5828836	-0.819926698	0.000119762	0.001894
ENSG00000128309	MPST	1979.661134	2776.666752	-0.488255101	0.000121703	0.001923
ENSG00000156042	CFAP70	744.8450343	1015.134165	-0.446299043	0.000121913	0.001924
ENSG00000055044	NOP58	4080.506567	3019.869403	0.434369186	0.000123193	0.001942
ENSG00000124172	ATP5F1E	3150.946339	2172.918132	0.536226031	0.000123333	0.001942
ENSG00000132716	DCAF8	1024.435153	1385.196142	-0.435233442	0.000123391	0.001942
ENSG00000128524	ATP6V1F	1537.680022	1117.905557	0.459538452	0.000123788	0.001947
ENSG00000070985	TRPM5	88.18011882	204.2446599	-1.21100392	0.000124035	0.001947
ENSG00000131018	SYNE1	232.1574721	132.1104333	0.808977553	0.000124041	0.001947
ENSG00000135127	BICDL1	1076.233236	743.3441454	0.534276307	0.000124205	0.001948
ENSG00000007376	RPUSD1	1356.843702	919.4435534	0.561791444	0.000125275	0.001962
ENSG00000106266	SNX8	1252.823163	895.6032193	0.484822816	0.00012564	0.001966
ENSG00000172331	BPGM	309.1307736	196.060906	0.657801486	0.000128157	0.002004
ENSG00000175063	UBE2C	2661.123063	1808.165554	0.557386391	0.000128389	0.002005
ENSG00000105229	PIAS4	1269.198942	915.3730082	0.47186988	0.000128459	0.002005
ENSG00000184076	UQCR10	1649.842015	1152.154344	0.518331483	0.000129483	0.002019
ENSG00000105219	CNTD2	6.215375523	28.75470706	-2.209348486	0.000130111	0.002027
ENSG00000105993	DNAJB6	3058.081088	2191.258523	0.481032882	0.000130286	0.002027
ENSG00000063245	EPN1	3885.93949	2871.45652	0.436512054	0.000130651	0.002031
ENSG00000121361	KCNJ8	133.9039267	65.78443693	1.02168861	0.000132065	0.002051
ENSG00000168026	TTC21A	71.11302591	144.8366635	-1.027476403	0.000132588	0.002055
ENSG00000166165	CKB	187.3145489	555.5994314	-1.56871221	0.000132593	0.002055
ENSG00000171604	CXXC5	2094.145089	2958.181285	-0.498578383	0.000133758	0.002071
ENSG00000113070	HBEGF	199.4954355	339.6200768	-0.767693762	0.000134724	0.002083
ENSG00000198208	RPS6KL1	110.3210956	193.3140248	-0.807943947	0.00013477	0.002083

ENSG00000177565	TBL1XR1	5826.451532	7622.393696	-0.387675312	0.000135951	0.002099
ENSG00000160953	MUM1	2734.815284	1934.661082	0.499693014	0.000136379	0.002104
ENSG00000175634	RPS6KB2	1102.648019	778.9404058	0.501854067	0.00013691	0.00211
ENSG00000144909	OSBPL11	1016.884807	719.1697126	0.500217534	0.000138354	0.002131
ENSG00000125731	SH2D3A	1709.326091	1284.804018	0.411857691	0.000139157	0.002141
ENSG00000114126	TFDP2	940.8923967	1294.898432	-0.460743423	0.000139858	0.00215
ENSG00000006062	MAP3K14	1138.836073	825.3612554	0.464982774	0.000140096	0.002151
ENSG00000160957	RECQL4	3630.815212	2296.215273	0.66117523	0.000140224	0.002151
ENSG00000182197	EXT1	1115.772229	831.4833769	0.424035936	0.000141064	0.002162
ENSG00000127588	GNG13	55.21135666	18.34651264	1.59412287	0.000142208	0.002178
ENSG00000162836	ACP6	463.9592305	699.0649231	-0.591569303	0.000143944	0.002202
ENSG00000151376	ME3	890.8531754	1206.914968	-0.438387631	0.000144756	0.002212
ENSG00000075131	TIPIN	678.2428663	427.6251911	0.666376285	0.000145924	0.002228
ENSG00000184898	RBM43	146.6660973	256.1332567	-0.80611185	0.000146075	0.002228
ENSG00000136111	TBC1D4	2535.100988	1801.173331	0.493315555	0.000146302	0.00223
ENSG00000215845	TSTD1	1596.146256	2106.343693	-0.400154713	0.000146596	0.002231
ENSG00000139174	PRICKLE1	52.77358731	18.28768686	1.531450279	0.000146627	0.002231
ENSG00000142444	TIMM29	549.7909168	377.5994917	0.542928093	0.000147813	0.002247
ENSG00000000460	C1orf112	830.9719125	492.6462213	0.754537307	0.000148345	0.002253
ENSG00000064763	FAR2	135.7199602	225.8317837	-0.736058449	0.000148677	0.002255
ENSG00000262585	LINC01979	14.0023351	49.00235078	-1.812999085	0.000148794	0.002255
ENSG00000130332	LSM7	2364.947463	1608.451158	0.55644818	0.000149276	0.00226
ENSG00000112812	PRSS16	555.0262385	789.0338262	-0.508207331	0.000150356	0.002272
ENSG00000173295	FAM86B3P	608.9790619	895.6273788	-0.556495205	0.000150364	0.002272
ENSG00000171310	CHST11	57.07819903	19.90626602	1.525527681	0.000150445	0.002272
ENSG00000167608	TMC4	1288.72452	2149.767251	-0.738222063	0.000151873	0.002291
ENSG00000232934	AL157786.1	114.375643	195.6668033	-0.774254813	0.000152485	0.002298
ENSG00000185513	L3MBTL1	348.2926732	523.9743571	-0.587919363	0.000154235	0.002321
ENSG00000181817	LSM10	659.1490548	448.7129507	0.554781938	0.000154298	0.002321
ENSG00000147118	ZNF182	91.21111229	167.950515	-0.880235345	0.000155217	0.002333
ENSG00000111696	NT5DC3	1650.787262	2168.509809	-0.393642929	0.000155411	0.002334
ENSG00000012232	EXTL3	1118.86137	822.1893167	0.444674959	0.000155709	0.002334
ENSG00000120889	TNFRSF10B	3865.149798	5370.085487	-0.474396576	0.000155762	0.002334
ENSG00000115163	CENPA	1048.039844	667.9258753	0.649662637	0.000155939	0.002334
ENSG00000145439	CBR4	763.2874591	1082.706658	-0.505227637	0.000156003	0.002334
ENSG00000247809	NR2F2-AS1	87.47324759	158.181758	-0.854605024	0.000157092	0.002348
ENSG00000204642	HLA-F	96.75179776	174.1809818	-0.847365706	0.000157313	0.002349
ENSG00000182512	GLRX5	1895.197889	1309.69154	0.533175557	0.000158072	0.002359
ENSG00000188747	NOXA1	603.4774451	1015.464853	-0.750666013	0.000158591	0.002364
ENSG00000131584	ACAP3	3069.866068	2227.100597	0.463319562	0.000158747	0.002364
ENSG00000100292	HMOX1	656.7001073	452.7456087	0.536770633	0.000158985	0.002366
ENSG00000152078	TMEM56	801.1828352	574.347101	0.479640679	0.000159759	0.002375
ENSG00000174177	CTU2	817.161294	541.3510492	0.594659149	0.000160278	0.002381

ENSG00000198125	MB	141.0637354	244.0039148	-0.790759497	0.000160422	0.002381
ENSG00000099889	ARVCF	791.9258502	1075.800697	-0.441714465	0.000162831	0.002414
ENSG00000105327	BBC3	575.3500876	935.4167568	-0.701383473	0.00016296	0.002414
ENSG00000139178	C1RL	421.3752835	606.0528303	-0.524401456	0.000163337	0.002417
ENSG00000260563	AC132872.1	103.7636329	197.9298127	-0.930807449	0.000163873	0.002423
ENSG00000196453	ZNF777	921.5120632	679.7236903	0.438807788	0.000164428	0.002429
ENSG00000221926	TRIM16	2061.250845	1566.865932	0.39586975	0.00016469	0.002431
ENSG00000161847	RAVER1	2021.338841	1382.249849	0.548646553	0.000167695	0.002472
ENSG00000148426	PROSER2	742.7644512	1022.404941	-0.460479833	0.00016779	0.002472
ENSG00000236234	AC091132.2	50.39498674	117.2793346	-1.216772163	0.000169449	0.002494
ENSG00000148291	SURF2	478.7719625	318.8483173	0.586794763	0.000170216	0.002503
ENSG00000148357	HMCN2	13.90760479	58.93080968	-2.083764733	0.00017054	0.002506
ENSG00000182481	KPNA2	10159.31192	6650.406289	0.611296731	0.00017069	0.002506
ENSG00000130734	ATG4D	653.2956667	459.5813964	0.507409256	0.000172241	0.002526
ENSG00000145920	CPLX2	77.68268029	32.04466282	1.270834994	0.000173475	0.002539
ENSG00000157426	AASDH	372.5296427	536.5057373	-0.52564297	0.000173504	0.002539
ENSG00000031823	RANBP3	2457.771233	1779.695969	0.465834994	0.00017356	0.002539
ENSG00000091622	PITPNM3	155.7166287	296.5150859	-0.929823532	0.000174923	0.002556
ENSG00000186594	MIR22HG	482.711776	327.9758878	0.558806167	0.000175342	0.00256
ENSG00000118412	CASP8AP2	1425.758431	986.2494012	0.532395554	0.000176422	0.002574
ENSG00000168350	DEGS2	14.97304553	52.99725476	-1.829974676	0.00017784	0.002592
ENSG00000139514	SLC7A1	5957.204035	8202.433177	-0.461434598	0.000179778	0.002618
ENSG00000168907	PLA2G4F	106.7098908	189.6866089	-0.831084	0.000181519	0.002639
ENSG00000128973	CLN6	1836.101805	1247.128853	0.558453058	0.000181529	0.002639
ENSG00000105612	DNASE2	986.1260848	1417.23888	-0.523597175	0.000182072	0.002644
ENSG00000121797	CCRL2	67.15795094	135.133897	-1.013398881	0.000184774	0.002681
ENSG00000169992	NLGN2	237.3356154	369.6505005	-0.637621964	0.00018525	0.002685
ENSG00000073331	ALPK1	194.395037	380.9178004	-0.972329706	0.000185519	0.002685
ENSG00000137310	TCF19	1336.507278	836.3365539	0.67659496	0.000185555	0.002685
ENSG00000175376	EIF1AD	926.3120756	595.0270105	0.639159237	0.000186516	0.002697
ENSG00000170006	TMEM154	158.5843797	303.2605677	-0.936656762	0.000188657	0.002726
ENSG00000075240	GRAMD4	396.9298849	652.6346666	-0.717720119	0.000190946	0.002756
ENSG00000184117	NIPSNAP1	2315.124856	3226.011206	-0.47881627	0.000191202	0.002757
ENSG00000136699	SMPD4	3804.684481	2597.244597	0.55095284	0.000191772	0.002763
ENSG00000075624	ACTB	86942.888	63270.7387	0.458526972	0.000192372	0.002769
ENSG00000171791	BCL2	58.65850525	14.67989785	2.015564501	0.000192856	0.002773
ENSG00000066230	SLC9A3	223.829229	359.728999	-0.683413731	0.000192971	0.002773
ENSG00000120915	EPHX2	307.4843595	467.8929095	-0.607052688	0.000193257	0.002775
ENSG00000171476	HOPX	7.296425867	33.26810567	-2.194030706	0.00019357	0.002777
ENSG00000167566	NCKAP5L	272.5079063	434.9928686	-0.674511301	0.0001954	0.002801
ENSG00000133067	LGR6	59.49423339	132.1627795	-1.154434585	0.000198029	0.002836
ENSG00000165238	WNK2	148.1379574	266.9473192	-0.848608659	0.000199494	0.002854
ENSG00000188566	NDOR1	1654.849015	1193.876582	0.471506348	0.000199645	0.002854

ENSG00000137171	KLC4	644.031218	953.9199447	-0.566945419	0.000199858	0.002855
ENSG00000197785	ATAD3A	2550.090545	1639.570188	0.637579116	0.000200944	0.002868
ENSG00000111424	VDR	2166.975474	1620.870479	0.418561424	0.000201409	0.002872
ENSG00000198719	DLL1	28.72455425	74.11666759	-1.367039601	0.000204076	0.002907
ENSG00000176697	BDNF	147.8898901	77.73767828	0.924701772	0.000207651	0.002956
ENSG00000114648	KLHL18	1425.27124	960.1149259	0.570430195	0.000209035	0.002973
ENSG00000091651	ORC6	1541.624472	991.1311309	0.637861642	0.00020977	0.00298
ENSG00000132746	ALDH3B2	33.79378517	93.28116219	-1.468307238	0.000209921	0.00298
ENSG00000126217	MCF2L	102.8261763	205.1234339	-0.996686347	0.000210347	0.002984
ENSG00000180769	WDFY3-AS2	121.4837325	205.4019256	-0.760464331	0.000213291	0.00302
ENSG00000143409	MINDY1	479.335529	669.4294051	-0.48190493	0.000213467	0.00302
ENSG00000090857	PDPR	636.1767297	908.0701245	-0.513955428	0.000213473	0.00302
ENSG00000138074	SLC5A6	1612.585175	1119.463907	0.527039043	0.000213832	0.003022
ENSG00000012963	UBR7	1739.300353	1112.327983	0.645135451	0.000214336	0.003026
ENSG00000235903	CPB2-AS1	28.36287745	5.503241887	2.348894673	0.000214418	0.003026
ENSG00000119969	HELLS	3065.021548	1997.920605	0.617655874	0.000215112	0.003033
ENSG00000173821	RNF213	7540.280139	10847.187	-0.524670487	0.000215366	0.003034
ENSG00000006747	SCIN	171.5529522	279.4728862	-0.702795765	0.000216832	0.003052
ENSG00000242516	LINC00960	84.0522655	153.0360868	-0.864733467	0.000218193	0.003068
ENSG00000134398	ERN2	2258.621616	3394.810411	-0.587994678	0.000220358	0.003092
ENSG00000113732	ATP6V0E1	1706.300416	1279.509348	0.415132971	0.000220367	0.003092
ENSG00000074071	MRPS34	3430.668323	2411.442602	0.508850263	0.000220558	0.003092
ENSG00000165891	E2F7	1170.75607	784.5255229	0.578151547	0.000220656	0.003092
ENSG00000110090	CPT1A	632.2026789	883.027067	-0.482599264	0.000220977	0.003092
ENSG00000113790	EHHADH	413.9222511	614.0420861	-0.569519096	0.000220994	0.003092
ENSG00000144730	IL17RD	621.9978288	898.0420705	-0.530404155	0.000221835	0.003101
ENSG00000169499	PLEKHA2	821.0107497	1297.510378	-0.66078728	0.000223091	0.003116
ENSG00000151014	NOCT	302.9984096	182.6141189	0.731925826	0.000224098	0.003127
ENSG00000188807	TMEM201	1135.518758	751.5622173	0.596102801	0.000225128	0.003139
ENSG00000149930	TAOK2	3221.062686	2366.351026	0.444983744	0.000225632	0.003143
ENSG00000145808	ADAMTS19	88.93367063	164.5115589	-0.888473268	0.000225843	0.003143
ENSG00000163827	LRRC2	4.050367446	23.27755838	-2.532534751	0.0002266	0.003151
ENSG00000070495	JMJD6	848.8171177	528.2615986	0.685097012	0.00022697	0.003152
ENSG00000197635	DPP4	43.73519593	92.98780602	-1.092092328	0.000227033	0.003152
ENSG00000123636	BAZ2B	764.0405903	1108.292755	-0.536412237	0.000227454	0.003155
ENSG00000090006	LTBP4	638.7314017	995.5944356	-0.640059977	0.00022819	0.003163
ENSG00000259673	IQCH-AS1	139.7449232	223.6077219	-0.679300982	0.000229154	0.003173
ENSG00000183044	ABAT	1334.052132	959.1894439	0.476070977	0.000229823	0.00318
ENSG00000212864	RNF208	218.8609356	352.6031901	-0.688258471	0.000230718	0.003189
ENSG00000177076	ACER2	385.5345314	258.2253659	0.577441935	0.000231001	0.003191
ENSG00000178202	KDELC2	1040.669136	1387.829116	-0.415469041	0.000231814	0.003199
ENSG00000153956	CACNA2D1	27.80832215	4.57541273	2.618485774	0.000232827	0.003211
ENSG00000228526	MIR34AHG	96.42373707	185.7733042	-0.947216069	0.000233888	0.003222

ENSG00000162599	NFIA	1169.191662	1566.549717	-0.421881572	0.000234749	0.003232
ENSG00000175550	DRAP1	2251.374598	1680.62994	0.422108648	0.000235194	0.003233
ENSG00000105325	FZR1	2791.963416	2107.919127	0.405661436	0.000235266	0.003233
ENSG00000166340	TPP1	1227.060906	1706.769734	-0.476466585	0.000236513	0.003248
ENSG00000182307	C8orf33	2026.068945	1539.399973	0.396510341	0.000237138	0.003253
ENSG00000140832	MARVELD3	740.1326415	515.8586896	0.521402247	0.000241394	0.003309
ENSG00000154822	PLCL2	43.57639237	11.19856985	1.95656215	0.00024175	0.003311
ENSG00000169957	ZNF768	1377.658109	1853.605363	-0.428440044	0.000242092	0.003313
ENSG00000078114	NEBL	4170.073945	5968.198747	-0.517289697	0.000247324	0.00338
ENSG00000152766	ANKRD22	246.0271702	395.9112941	-0.687870085	0.000247413	0.00338
ENSG00000113108	APBB3	779.0999029	1169.727893	-0.586128248	0.000248647	0.003394
ENSG00000085433	WDR47	430.6600698	291.7861443	0.56199751	0.000254718	0.003474
ENSG00000170909	OSCAR	13.8890878	43.98290648	-1.664100877	0.000255494	0.003482
ENSG00000074590	NUAK1	492.1945753	769.2695538	-0.644614601	0.000255785	0.003483
ENSG00000136875	PRPF4	2647.845396	1785.648732	0.568556265	0.000256927	0.003496
ENSG00000182168	UNC5C	73.87763505	28.55107433	1.373878068	0.000259147	0.003523
ENSG00000006118	TMEM132A	830.0112042	1113.385953	-0.423688425	0.000260996	0.003545
ENSG00000142687	KIAA0319L	1706.430458	2298.660158	-0.429980215	0.000262746	0.003566
ENSG00000115457	IGFBP2	3079.447645	2310.700303	0.414237265	0.00026355	0.003574
ENSG00000261236	BOP1	3634.775266	2596.503869	0.485576645	0.000265032	0.003591
ENSG00000204103	MAFB	18.64612657	55.74013102	-1.58450228	0.000268109	0.00363
ENSG00000178295	GEN1	1816.489482	1308.609002	0.473359205	0.000268987	0.003639
ENSG00000118276	B4GALT6	542.2088202	381.585613	0.507213949	0.000274474	0.00371
ENSG00000174775	HRAS	2022.308864	1416.263263	0.514324685	0.000275313	0.003718
ENSG00000183960	KCNH8	50.57832198	14.0929667	1.828877604	0.000275715	0.00372
ENSG00000035664	DAPK2	66.00721246	149.9230577	-1.182178061	0.000276524	0.003726
ENSG00000006534	ALDH3B1	814.5783121	1214.129114	-0.575768975	0.000276694	0.003726
ENSG00000129235	TXNDC17	1043.056251	708.2934625	0.558690104	0.000276828	0.003726
ENSG00000165879	FRAT1	118.182323	199.8506907	-0.758428768	0.000278606	0.003747
ENSG00000109654	TRIM2	3408.7206	4869.878707	-0.514680688	0.000279996	0.00376
ENSG00000143753	DEGS1	856.4860879	577.2004772	0.570332222	0.00028003	0.00376
ENSG00000151575	TEX9	68.90991118	28.58871419	1.260090123	0.000281027	0.00377
ENSG00000151725	CENPU	1787.565102	1305.942119	0.45288547	0.000284428	0.00381
ENSG00000159596	TMEM69	732.9974911	1028.760011	-0.489505675	0.000284698	0.00381
ENSG00000105289	TJP3	1836.401623	2582.512598	-0.49196066	0.000284718	0.00381
ENSG00000060491	OGFR	2133.51023	1585.914132	0.42806689	0.00028553	0.003817
ENSG00000141759	TXNL4A	2666.350164	1902.935246	0.486689401	0.00028566	0.003817
ENSG00000084652	TXLNA	4786.82462	3658.361815	0.38791205	0.000285891	0.003817
ENSG00000256029	SNHG28	34.78643048	87.92514466	-1.335012568	0.000286833	0.003826
ENSG00000006459	KDM7A	2554.096499	3725.739852	-0.544764675	0.000287731	0.003835
ENSG00000163378	EOGT	776.7014303	565.0612392	0.45814469	0.000288484	0.003842
ENSG00000132768	DPH2	1182.777257	826.5628288	0.517277468	0.000289324	0.00385
ENSG00000049283	EPN3	667.6920972	968.5857376	-0.537296906	0.000294356	0.003914

ENSG00000142920	AZIN2	38.86725508	84.5574347	-1.119515483	0.000294769	0.003916
ENSG00000182107	TMEM30B	559.6796766	817.037705	-0.546833137	0.000295714	0.003925
ENSG00000229404	LINC00858	137.950514	234.9563257	-0.769186793	0.000298221	0.003955
ENSG00000164241	C5orf63	165.8448443	257.6474916	-0.635662891	0.000299231	0.003965
ENSG00000205464	ATP6AP1L	138.6621864	267.6236504	-0.947486671	0.000299447	0.003965
ENSG00000006576	PHTF2	1434.323829	1023.742182	0.486729378	0.000301273	0.003986
ENSG00000166888	STAT6	4675.339073	6317.082637	-0.434267163	0.000301755	0.003989
ENSG00000011007	ELOA	2775.013083	2034.335533	0.448045512	0.000303038	0.004
ENSG00000170917	NUDT6	151.9361668	246.4460246	-0.69897102	0.000303048	0.004
ENSG00000105376	ICAM5	213.0459647	327.4834121	-0.618581068	0.000303821	0.004007
ENSG00000105447	GRWD1	2727.692522	1831.698952	0.574703794	0.000305023	0.004017
ENSG00000147526	TACC1	7567.349473	9590.894595	-0.341890793	0.00030506	0.004017
ENSG00000143793	C1orf35	959.3428106	634.9500008	0.596358013	0.000305506	0.00402
ENSG00000134755	DSC2	1245.589057	842.527674	0.563265726	0.000308991	0.004057
ENSG00000168685	IL7R	25.45656094	4.583057199	2.444998464	0.00030905	0.004057
ENSG00000117691	NENF	893.3863855	661.6631824	0.432961258	0.000309112	0.004057
ENSG00000172757	CFL1	19460.73418	14123.42871	0.462491518	0.000311626	0.004087
ENSG00000142632	ARHGEF19	110.8020343	183.8279224	-0.729831407	0.000312236	0.004092
ENSG00000117115	PADI2	18.14579803	52.24699367	-1.524175006	0.000316078	0.004138
ENSG00000163577	EIF5A2	1135.402071	855.670053	0.407885918	0.000316265	0.004138
ENSG00000180992	MRPL14	1146.085819	814.6578432	0.492830874	0.000319206	0.004173
ENSG00000185551	NR2F2	7431.353975	9481.340093	-0.35153457	0.000319529	0.004174
ENSG00000151327	FAM177A1	1652.822895	1261.874975	0.388996379	0.000323552	0.004223
ENSG00000134330	IAH1	466.148881	664.1288348	-0.510896829	0.000324385	0.00423
ENSG00000165055	METTL2B	1189.422969	864.2943281	0.461328856	0.000326712	0.004256
ENSG00000117984	CTSD	5071.409606	6522.802928	-0.363193698	0.000326874	0.004256
ENSG00000077514	POLD3	1487.754817	1021.788094	0.54218334	0.000328268	0.004271
ENSG00000272502	AC104958.2	58.33039289	110.7055542	-0.923693758	0.000332487	0.004322
ENSG00000228223	HCG11	116.3385399	197.2962896	-0.764795695	0.000332888	0.004324
ENSG00000159128	IFNGR2	486.577166	738.1140638	-0.6019671	0.000334203	0.004337
ENSG00000127324	TSPAN8	3964.334242	2978.626875	0.412149071	0.000334392	0.004337
ENSG00000130713	EXOSC2	1697.591269	1153.645091	0.557795004	0.000335189	0.004344
ENSG00000148229	POLE3	2389.724236	1592.281298	0.585965158	0.000335722	0.004347
ENSG00000112715	VEGFA	1905.203318	5164.590729	-1.438698534	0.000336348	0.00435
ENSG00000116688	MFN2	5315.1824	3869.302425	0.458133745	0.000336478	0.00435
ENSG00000078403	MLLT10	1356.537578	1821.340979	-0.42535439	0.000336967	0.004353
ENSG00000124749	COL21A1	22.27528618	64.46218531	-1.538800972	0.000338894	0.004374
ENSG00000173264	GPR137	1012.933512	745.0519665	0.442817139	0.000340426	0.004391
ENSG00000143036	SLC44A3	218.8396219	330.9624712	-0.598311113	0.000340852	0.004393
ENSG00000179627	ZBTB42	222.4907681	343.5742668	-0.627433992	0.000343379	0.004422
ENSG00000148225	WDR31	105.5862432	187.8654592	-0.833271889	0.00034439	0.004431
ENSG00000198648	STK39	737.9608727	489.8891622	0.59081896	0.00034674	0.004458
ENSG00000125656	CLPP	2738.191271	2044.154304	0.421865573	0.000350099	0.004498

ENSG00000050820	BCAR1	4751.203675	3714.677869	0.355007257	0.000351784	0.004516
ENSG00000002330	BAD	389.738857	264.9506972	0.555898134	0.000353154	0.00453
ENSG00000155974	GRIP1	46.60396795	17.02370707	1.452928361	0.000354188	0.004539
ENSG00000114654	EFCC1	9.093942368	34.59874415	-1.924801371	0.000355586	0.004554
ENSG00000160818	GPATCH4	1982.033481	1326.898594	0.579301261	0.000356058	0.004556
ENSG00000168101	NUDT16L1	980.681727	696.2778569	0.493847965	0.000364838	0.004663
ENSG00000147130	ZMYM3	1464.795195	1929.50522	-0.397897454	0.000364968	0.004663
ENSG00000101751	POLI	329.3812866	492.0250674	-0.579066851	0.000368237	0.004701
ENSG00000111684	LPCAT3	553.2006101	391.7745088	0.497823	0.000368784	0.004704
ENSG00000130193	THEM6	730.9941565	525.877483	0.475803424	0.000372527	0.004748
ENSG00000125378	BMP4	15695.19111	20619.4558	-0.393731415	0.000374219	0.004766
ENSG00000058085	LAMC2	2579.18052	1886.571853	0.450799033	0.000374558	0.004767
ENSG00000060982	BCAT1	47.32150677	15.76317832	1.584255236	0.000374847	0.004767
ENSG00000122687	MRM2	1420.389924	997.3075346	0.510431708	0.000376495	0.004784
ENSG00000203760	CENPW	459.1620505	301.6014381	0.60662665	0.000379837	0.004823
ENSG00000104823	ECH1	762.9389434	1039.168451	-0.446383847	0.000385567	0.004892
ENSG00000111328	CDK2AP1	1109.633391	759.6686871	0.54652324	0.000386146	0.004895
ENSG00000146278	PNRC1	635.1990802	916.7941432	-0.529811212	0.000387557	0.004908
ENSG00000067334	DNTTIP2	2168.120302	1516.046853	0.516052585	0.00038787	0.004908
ENSG00000013275	PSMC4	2877.455714	2133.701205	0.431393753	0.000388032	0.004908
ENSG00000140675	SLC5A2	5.429583496	25.44701641	-2.223223681	0.000388728	0.004913
ENSG00000120798	NR2C1	824.8675356	1095.908761	-0.409865434	0.000389371	0.004917
ENSG00000124107	SLPI	270.1474745	441.8010099	-0.711578676	0.000390126	0.004923
ENSG00000115568	ZNF142	1710.912996	1191.533123	0.522371255	0.000390911	0.004929
ENSG00000165959	CLMN	98.22972417	176.1304256	-0.842520725	0.000391315	0.00493
ENSG00000156650	KAT6B	1036.73664	1494.40927	-0.527916321	0.000392299	0.004938
ENSG00000251562	MALAT1	9996.800313	14794.88301	-0.565552348	0.000392555	0.004938
ENSG00000172336	POP7	1012.983086	668.2718651	0.600907473	0.000392813	0.004938
ENSG00000100600	LGMN	1230.18794	1858.039703	-0.594996337	0.000395309	0.004965
ENSG00000055070	SZRD1	3563.245885	2681.784777	0.41003122	0.000396719	0.004979
ENSG00000105723	GSK3A	1847.674295	1428.488169	0.371024791	0.000399378	0.005009
ENSG00000169905	TOR1AIP2	5517.471022	4260.907848	0.372884849	0.000401181	0.005028
ENSG00000166845	C18orf54	624.0794027	376.2017851	0.731842448	0.000403172	0.005049
ENSG00000129562	DAD1	3133.980278	2336.330248	0.423598956	0.000405123	0.005069
ENSG00000088305	DNMT3B	88.96596251	154.7624441	-0.800045754	0.000405768	0.005073
ENSG00000180884	ZNF792	66.00410517	123.1177963	-0.901404878	0.000406054	0.005073
ENSG00000267056	AC005336.1	34.68170545	9.225391322	1.891946379	0.000409139	0.005108
ENSG00000155265	GOLGA7B	289.0019269	187.9121558	0.620114307	0.000410242	0.005118
ENSG00000030110	BAK1	598.7593998	402.6974117	0.573940627	0.000410649	0.005119
ENSG00000163812	ZDHHC3	4034.917291	3087.859721	0.38572658	0.000413103	0.005145
ENSG00000168393	DTYMK	2340.099247	1575.551136	0.570972881	0.000421851	0.00525
ENSG00000150867	PIP4K2A	1017.759049	766.9068312	0.408656298	0.000422707	0.005253
ENSG00000223478	AL441992.1	175.6925974	100.289584	0.811486	0.000422745	0.005253

ENSG00000158716	DUSP23	336.2606471	222.1028921	0.597233937	0.000424563	0.005272
ENSG00000137714	FDX1	1406.487313	1040.122069	0.435140511	0.000425747	0.005283
ENSG00000125798	FOXA2	123.2757449	208.1256392	-0.755647579	0.000427795	0.005304
ENSG00000257829	AC121757.1	15.26432379	51.97418204	-1.766715971	0.000429417	0.00532
ENSG00000249115	HAUS5	1514.778014	1071.045447	0.500447349	0.000432027	0.005349
ENSG00000198569	SLC34A3	259.4459351	392.2952021	-0.596874699	0.000438459	0.005424
ENSG00000137814	HAUS2	1672.221219	1160.690003	0.52715605	0.000438769	0.005424
ENSG00000188215	DCUN1D3	1010.462305	757.8939848	0.414548494	0.000440083	0.005436
ENSG00000125148	MT2A	4579.088153	3212.915988	0.510919742	0.000440842	0.005441
ENSG00000175197	DDIT3	274.2749139	497.7032525	-0.85882331	0.000445843	0.005499
ENSG00000160570	DEDD2	1247.269975	846.1705687	0.560478926	0.000447999	0.005521
ENSG00000145860	RNF145	3189.250916	4086.006866	-0.357639435	0.000451946	0.005566
ENSG00000161036	LRWD1	820.9099672	594.3093374	0.466459416	0.000454518	0.005593
ENSG00000018408	WWTR1	107.3652427	180.5269454	-0.751843495	0.000456127	0.005609
ENSG00000112874	NUDT12	609.8570391	868.4603704	-0.510639644	0.000458058	0.005628
ENSG00000132603	NIP7	1588.954397	1132.080147	0.48919243	0.000461434	0.005665
ENSG00000260231	KDM7A-DT	280.6090409	437.5261027	-0.641139031	0.000462873	0.005679
ENSG00000100726	TELO2	2800.038708	1985.473763	0.496266917	0.000463217	0.005679
ENSG00000080839	RBL1	1013.680784	688.1500602	0.559009025	0.00046448	0.00569
ENSG00000234127	TRIM26	2627.003428	1968.491138	0.416253588	0.000465489	0.005698
ENSG00000151718	WWC2	835.3556824	1139.530852	-0.448126183	0.000467065	0.00571
ENSG00000185298	CCDC137	1320.551989	907.8271041	0.541330053	0.000467137	0.00571
ENSG00000106049	HIBADH	1486.829834	2033.412088	-0.452068342	0.000467563	0.005711
ENSG00000171224	FAM241B	316.2722868	209.0665302	0.59774094	0.000467962	0.005711
ENSG00000172432	GTPBP2	2490.696712	3713.08943	-0.576047681	0.000469695	0.005728
ENSG00000103479	RBL2	2179.941348	3009.591877	-0.465375324	0.000471811	0.00575
ENSG00000117318	ID3	1657.73356	2299.001779	-0.472020994	0.000474561	0.005779
ENSG00000156983	BRPF1	1388.136393	1005.510481	0.465292043	0.000475111	0.005781
ENSG00000276672	AL161891.1	114.5675005	59.09451832	0.955370533	0.000475646	0.005783
ENSG00000119632	IFI27L2	71.99064086	128.7208521	-0.840345029	0.000477236	0.005798
ENSG00000130165	ELOF1	1297.854888	908.7009862	0.514607487	0.000479449	0.005821
ENSG00000127616	SMARCA4	10268.95419	8131.764062	0.336631104	0.00048074	0.005832
ENSG00000127946	HIP1	1975.915227	2622.263104	-0.408281428	0.000482366	0.005848
ENSG00000164896	FASTK	2617.364511	2059.973876	0.345478388	0.000483257	0.005851
ENSG00000115850	LCT	86.00113596	41.10637587	1.071886464	0.000483338	0.005851
ENSG00000041353	RAB27B	2401.572247	3209.477304	-0.418561987	0.00048564	0.005874
ENSG00000196372	ASB13	478.7795388	718.7055423	-0.586218088	0.000488124	0.0059
ENSG00000140548	ZNF710	554.9859866	777.7295283	-0.487143004	0.000489466	0.005912
ENSG00000012822	CALCOCO1	642.264107	1000.271867	-0.639459866	0.00049039	0.005919
ENSG00000197557	TTC30A	73.71617566	139.3578701	-0.922537185	0.000495988	0.005982
ENSG00000088356	PDRG1	807.2244845	518.7056891	0.639391358	0.000496623	0.005985
ENSG00000126934	MAP2K2	3602.649771	2817.983326	0.354305681	0.000501753	0.00604
ENSG00000130803	ZNF317	1895.599479	1482.891542	0.354363613	0.000502135	0.00604

ENSG00000182957	SPATA13	619.1997659	844.3093639	-0.447792885	0.000502308	0.00604
ENSG00000100413	POLR3H	1454.586984	1021.997923	0.509651924	0.000505071	0.006069
ENSG00000169891	REPS2	48.43612155	18.22134796	1.404327593	0.000505976	0.006075
ENSG00000138771	SHROOM3	4432.916116	6028.901448	-0.443767555	0.000509572	0.006114
ENSG00000134369	NAV1	105.5883886	179.9515437	-0.768224414	0.000512068	0.00614
ENSG00000099800	TIMM13	1833.541666	1236.825701	0.568335698	0.000515302	0.006174
ENSG00000151715	TMEM45B	391.9546665	558.9015961	-0.512950899	0.000517222	0.006192
ENSG00000160949	TONSL	3506.173508	2355.326765	0.574193156	0.000517695	0.006193
ENSG00000132507	EIF5A	20963.34181	14456.83504	0.536139111	0.000518183	0.006195
ENSG00000171992	SYNPO	120.1375188	229.4805019	-0.93431853	0.000519663	0.006206
ENSG00000171365	CLCN5	1313.770141	987.9295722	0.411151409	0.000519923	0.006206
ENSG00000129451	KLK10	1273.520393	2011.338799	-0.659413968	0.000520337	0.006207
ENSG00000126461	SCAF1	3432.046644	2626.37688	0.385945329	0.000521043	0.006211
ENSG00000106263	EIF3B	9557.721941	6950.062879	0.459702588	0.000523611	0.006237
ENSG00000078018	MAP2	1319.808182	930.9018943	0.502953896	0.000524855	0.006247
ENSG00000108588	CCDC47	4315.805438	3388.921601	0.348645664	0.000526749	0.006265
ENSG00000033100	CHPF2	981.7524505	728.6748883	0.429806885	0.000529345	0.006288
ENSG00000116133	DHCR24	14588.78676	10847.87117	0.427462073	0.000529473	0.006288
ENSG00000088247	KHSRP	19280.4187	14622.09391	0.399002317	0.000530494	0.006295
ENSG00000168952	STXBP6	472.5693784	683.5519922	-0.533226836	0.000530915	0.006295
ENSG00000204054	LINC00963	334.8555536	549.2650335	-0.714386709	0.000531598	0.006295
ENSG00000196872	KIAA1211L	541.8024974	791.5204475	-0.547576767	0.000531872	0.006295
ENSG00000266173	STRADA	289.3780889	448.3078363	-0.630054663	0.000531926	0.006295
ENSG00000277053	GTF2IP1	240.0859392	348.4285937	-0.537752719	0.000532805	0.006298
ENSG00000104133	SPG11	2031.503694	2918.086723	-0.522623197	0.000533017	0.006298
ENSG00000163818	LZTFL1	313.5338441	473.0971894	-0.594972206	0.000536816	0.006339
ENSG00000280022	AC126544.1	7.233987437	31.90811138	-2.13624601	0.000539409	0.006365
ENSG00000166046	TCP11L2	133.9464132	265.8949042	-0.989006415	0.000545501	0.006428
ENSG00000162769	FLVCR1	947.0039858	691.9618856	0.453241478	0.000545796	0.006428
ENSG00000081803	CADPS2	50.43656295	98.08810723	-0.962842389	0.000546402	0.006428
ENSG00000040199	PHLPP2	1472.721589	1068.817202	0.4631688	0.000546588	0.006428
ENSG00000257702	LBX2-AS1	105.4713611	184.1275411	-0.804433048	0.000546744	0.006428
ENSG00000134986	NREP	143.4399391	226.8205345	-0.662184391	0.000550026	0.006462
ENSG00000115267	IFIH1	798.5210714	588.9621733	0.438433008	0.000553994	0.006504
ENSG00000047617	ANO2	24.22415664	5.185568528	2.238183867	0.000554732	0.006508
ENSG00000100983	GSS	2351.86879	3147.451172	-0.420553254	0.000555106	0.006508
ENSG00000141258	SGSM2	1843.208344	2597.983753	-0.49513148	0.000557041	0.006526
ENSG00000163346	PBXIP1	756.1479953	1094.408043	-0.534024208	0.000557666	0.006528
ENSG00000253368	TRNP1	158.782311	256.3077791	-0.68975249	0.000558714	0.006536
ENSG00000258881	AC007040.2	21.9869153	4.760436831	2.205692464	0.000562159	0.006572
ENSG00000179912	R3HDM2	532.1854779	861.7768973	-0.695724508	0.000563219	0.006576
ENSG00000175048	ZDHHC14	449.530235	308.2105569	0.546006857	0.000563351	0.006576
ENSG00000186715	MST1L	22.03899674	57.49125663	-1.376312905	0.000564916	0.00659

ENSG00000152256	PDK1	734.8032193	1044.177175	-0.507005072	0.000566929	0.006608
ENSG00000107815	TWNK	1234.143664	876.8233784	0.493744738	0.000569251	0.006631
ENSG00000196876	SCN8A	560.7588406	402.6622331	0.479035594	0.000570471	0.00664
ENSG00000106397	PLOD3	3520.290029	2649.192776	0.41008935	0.000571317	0.006645
ENSG00000144895	EIF2A	3513.49636	4541.230277	-0.370384067	0.000573908	0.006671
ENSG00000174370	C11orf45	199.1478442	297.1286203	-0.577965376	0.000574988	0.006679
ENSG00000084073	ZMPSTE24	2952.752631	2059.574902	0.519665504	0.000581903	0.006754
ENSG00000169359	SLC33A1	394.6761753	546.2057952	-0.469144718	0.000588197	0.006822
ENSG00000165322	ARHGAP12	878.0851169	1202.517432	-0.45386944	0.000589256	0.00683
ENSG00000100139	MICALL1	1393.272139	1079.297737	0.368337224	0.000593441	0.006873
ENSG00000103005	USB1	2612.96858	1848.037371	0.499904716	0.000594053	0.006876
ENSG00000185442	FAM174B	518.5688032	712.7526934	-0.459721711	0.000594709	0.006878
ENSG00000070182	SPTB	1502.231079	1128.765907	0.412087137	0.000598014	0.006912
ENSG00000159259	CHAF1B	1567.585461	1060.8333	0.56368225	0.00059851	0.006912
ENSG00000178623	GPR35	670.0653418	956.8031147	-0.514052808	0.000599378	0.006917
ENSG00000175756	AURKAIP1	3165.04287	2295.529265	0.463534088	0.000599771	0.006917
ENSG00000147650	LRP12	679.1377632	454.9616569	0.578803009	0.000603625	0.006955
ENSG00000112624	BICRAL	589.4566071	856.829765	-0.540416171	0.000603902	0.006955
ENSG00000094804	CDC6	3328.149413	2306.905652	0.528938778	0.00060521	0.006965
ENSG00000110172	CHORDC1	2275.889203	1494.452227	0.607066382	0.000606024	0.00697
ENSG00000141543	EIF4A3	6434.406983	4666.01836	0.463642404	0.000606708	0.006973
ENSG00000260793	AC003102.1	107.0567201	175.4380595	-0.712147	0.00061192	0.007028
ENSG00000088179	PTPN4	617.8881892	854.5087824	-0.467414376	0.000613443	0.00704
ENSG00000164237	CMBL	1247.476637	1698.261997	-0.445155513	0.000614917	0.007052
ENSG00000204866	IGFL2	426.2863838	618.2509723	-0.537559348	0.000618771	0.007089
ENSG00000142765	SYTL1	197.9021755	640.1265949	-1.693470977	0.000619284	0.007089
ENSG00000048162	NOP16	889.738723	588.7387248	0.596162686	0.000619412	0.007089
ENSG00000166532	RIMKLB	73.18968062	29.20561217	1.31390871	0.000621579	0.007109
ENSG00000117266	CDK18	105.6978557	220.5926432	-1.061591179	0.000622318	0.007112
ENSG00000183161	FANCF	382.8499157	530.8122882	-0.4722936	0.000622812	0.007112
ENSG00000204060	FOXO6	27.20617596	65.37165136	-1.266754917	0.000623146	0.007112
ENSG00000123975	CKS2	2377.581562	1621.025715	0.552655175	0.000625974	0.007137
ENSG00000172301	COPRS	864.9855711	626.1753986	0.466534418	0.000626408	0.007137
ENSG00000196141	SPATS2L	2274.99129	1783.392431	0.351063612	0.000626665	0.007137
ENSG00000084092	NOA1	1330.826987	1781.915269	-0.421584133	0.000627725	0.007144
ENSG00000146205	ANO7	88.18119148	155.1250077	-0.811962082	0.000628139	0.007144
ENSG00000143153	ATP1B1	4875.613739	6914.175682	-0.504089591	0.000629186	0.007151
ENSG00000147044	CASK	2716.227919	3555.959138	-0.388850787	0.000633127	0.00719
ENSG00000242485	MRPL20	2444.754433	1738.715778	0.491814838	0.000635203	0.007207
ENSG00000165175	MID1IP1	3190.197014	4064.80352	-0.349489048	0.000635474	0.007207
ENSG00000149503	INCENP	3396.552596	2402.593841	0.499443426	0.000636451	0.007208
ENSG00000204396	VWA7	294.1490133	442.2317587	-0.587326687	0.000636464	0.007208
ENSG00000124535	WRNIP1	1304.181514	957.2308976	0.446501807	0.000637587	0.007216

ENSG00000245498	AP000866.1	36.90226451	85.2160917	-1.206383622	0.000645146	0.007296
ENSG00000130811	EIF3G	3445.037485	2716.111917	0.342984043	0.000646578	0.007308
ENSG00000166387	PPFIBP2	1703.79543	2221.681291	-0.383180744	0.000651193	0.007355
ENSG00000122783	CYREN	692.6716618	501.9506614	0.464447447	0.000652979	0.00737
ENSG00000204623	ZNRD1ASP	319.2153267	445.6999411	-0.482186978	0.000656516	0.007402
ENSG00000186575	NF2	2571.533203	1963.301094	0.38945784	0.000657075	0.007402
ENSG00000129103	SUMF2	1994.621654	2611.852592	-0.389162618	0.000657216	0.007402
ENSG00000169814	BTD	309.4943958	440.2415324	-0.509879848	0.000657613	0.007402
ENSG00000184990	SIVA1	1901.292975	1318.705696	0.528222516	0.000667003	0.007502
ENSG00000109472	CPE	193.8004887	305.9560068	-0.659703779	0.000672607	0.00756
ENSG00000134138	MEIS2	251.9760205	159.0016776	0.661780964	0.000677427	0.007605
ENSG00000004777	ARHGAP33	613.6669962	417.7215444	0.555198759	0.000677559	0.007605
ENSG00000204611	ZNF616	236.2025353	339.3330117	-0.523080572	0.000679093	0.007617
ENSG00000128849	CGNL1	13.15012459	39.0925005	-1.575370485	0.000681636	0.00764
ENSG00000173786	CNP	2230.654716	1729.495654	0.367114704	0.000685119	0.007674
ENSG00000174233	ADCY6	1566.540928	2055.43811	-0.391849058	0.000692328	0.00775
ENSG00000152253	SPC25	545.4686018	358.9259024	0.603070088	0.000695528	0.00778
ENSG00000253873	PCDHGA11	5.05918453	23.8800697	-2.23012373	0.000695959	0.00778
ENSG00000188290	HES4	1038.729387	755.9550387	0.45921386	0.000701241	0.007833
ENSG00000196305	IARS	10284.74714	13227.17244	-0.36303226	0.000704325	0.007862
ENSG00000101003	GINS1	1873.569211	1207.4511	0.634158179	0.000705455	0.00787
ENSG00000101417	PXMP4	201.0925347	127.3786955	0.656516099	0.000706814	0.00788
ENSG00000172009	THOP1	4089.013839	2891.733825	0.499966798	0.000709081	0.007899
ENSG00000114631	PODXL2	726.6170525	1029.001255	-0.502233696	0.000710028	0.007905
ENSG00000013810	TACC3	5473.159076	3797.779742	0.527289958	0.000711695	0.007918
ENSG00000271201	AC247036.1	27.05374933	70.43937988	-1.382902379	0.000712185	0.007918
ENSG00000124788	ATXN1	1824.282531	2565.262285	-0.491731349	0.000712715	0.007918
ENSG00000167925	GHDC	487.0760221	668.3304571	-0.457129115	0.000725916	0.008059
ENSG00000124523	SIRT5	342.4740255	532.6505581	-0.637629856	0.000727421	0.008071
ENSG00000106261	ZKSCAN1	3164.037066	4167.592177	-0.397610264	0.000727956	0.008071
ENSG00000100401	RANGAP1	7257.608877	5241.840221	0.469485871	0.000728415	0.008071
ENSG00000116062	MSH6	988.1521896	753.364001	0.391840536	0.000733163	0.008118
ENSG00000185340	GAS2L1	994.7092008	736.8151221	0.433566055	0.000734569	0.008128
ENSG00000146263	MMS22L	2216.501117	1597.061535	0.473153657	0.000737329	0.008149
ENSG00000106733	NMRK1	639.0503331	891.0021971	-0.47968417	0.000737456	0.008149
ENSG00000003147	ICA1	907.8655613	1302.833501	-0.521469566	0.000739788	0.008169
ENSG00000177873	ZNF619	183.0583491	275.4042045	-0.590689096	0.000740891	0.008176
ENSG00000196584	XRCC2	1469.949945	957.7250347	0.618506738	0.000742492	0.008188
ENSG00000147100	SLC16A2	87.45442	151.2251303	-0.791062265	0.000744535	0.008205
ENSG00000113407	TARS	7579.258902	9763.886741	-0.365462517	0.000747395	0.008231
ENSG00000132952	USPL1	581.0075188	420.4509077	0.467038923	0.000762112	0.008387
ENSG00000165516	KLHDC2	560.4581082	804.2346342	-0.52129103	0.000762802	0.008389
ENSG00000112659	CUL9	1361.84739	1827.444252	-0.423998779	0.000766748	0.008427

ENSG00000026297	RNASET2	613.1364621	835.4122142	-0.44588696	0.000771305	0.008471
ENSG00000105364	MRPL4	2205.843417	1566.302535	0.494209484	0.000772689	0.008476
ENSG00000128965	CHAC1	704.3083594	1690.721687	-1.263170821	0.000772753	0.008476
ENSG00000225953	SATB2-AS1	211.1172461	309.3172219	-0.550743792	0.000775135	0.008493
ENSG00000105887	MTPN	7873.147617	6226.403788	0.33849717	0.000775341	0.008493
ENSG00000156253	RWDD2B	695.6350535	928.8788707	-0.417954177	0.000777878	0.008515
ENSG00000185896	LAMP1	4794.444171	3661.808612	0.388733478	0.000780777	0.008541
ENSG00000147155	EBP	2171.698689	1500.859838	0.533417435	0.000783447	0.008564
ENSG00000112110	MRPL18	2149.708992	1476.953915	0.541613359	0.000789951	0.00863
ENSG00000089775	ZBTB25	916.6106471	1258.205197	-0.457063254	0.000792637	0.008653
ENSG00000114767	RRP9	1250.583776	892.7126522	0.48709546	0.000794958	0.008667
ENSG00000100320	RBFOX2	4217.059535	5402.164982	-0.357413956	0.000794967	0.008667
ENSG00000161328	LRRC56	100.8614963	175.6872963	-0.802198475	0.000795577	0.008668
ENSG00000155115	GTF3C6	1319.83624	971.4143473	0.441805096	0.000796136	0.008668
ENSG00000133740	E2F5	375.9683207	519.0738892	-0.464901327	0.000798414	0.008687
ENSG00000237940	LINC01238	81.02581425	143.1123866	-0.817925897	0.000801745	0.008717
ENSG00000170608	FOXA3	7.384268735	27.71987071	-1.916231646	0.000802203	0.008717
ENSG00000159023	EPB41	2369.753825	3040.507584	-0.359628291	0.000803988	0.008731
ENSG00000166762	CATSPER2	192.9276749	301.631169	-0.642936529	0.000805192	0.008738
ENSG00000003400	CASP10	397.8881447	582.1355605	-0.549618678	0.000819855	0.008891
ENSG00000090520	DNAJB11	1513.793335	1955.475827	-0.369487426	0.00082178	0.008906
ENSG00000213339	QTRT1	1462.553943	1123.557445	0.380668944	0.00082267	0.00891
ENSG00000116574	RHOU	1003.944001	1478.706082	-0.558902404	0.000828096	0.008963
ENSG00000108175	ZMIZ1	1186.881401	1656.364058	-0.481167991	0.000833362	0.009014
ENSG00000092470	WDR76	1157.330839	810.4236786	0.514240426	0.000835756	0.009034
ENSG00000204791	SMPD5	5.778558076	27.73909037	-2.253610193	0.00084018	0.009066
ENSG00000089351	GRAMD1A	31.56286911	82.17438486	-1.381563429	0.000840395	0.009066
ENSG00000134905	CARS2	2029.412559	1584.963442	0.356726096	0.000840995	0.009066
ENSG00000119922	IFIT2	56.48262968	114.114909	-1.019905868	0.000840997	0.009066
ENSG00000111206	FOXM1	3839.151994	2745.264552	0.483819708	0.000842683	0.009079
ENSG00000236255	AC009404.1	267.2260589	172.6358606	0.632453447	0.000843985	0.009081
ENSG00000118707	TGIF2	69.12895618	124.7245376	-0.850950113	0.000844013	0.009081
ENSG00000108852	MPP2	71.08634891	130.929625	-0.883560307	0.000846558	0.009102
ENSG00000186517	ARHGAP30	49.6941302	108.0873897	-1.119987051	0.000848834	0.009121
ENSG00000174562	KLK15	11.17692236	37.67381178	-1.747820526	0.000849616	0.009123
ENSG00000134248	LAMTOR5	1472.570384	1120.262542	0.394376452	0.000854921	0.009174
ENSG00000163728	TTC14	1463.712599	1941.812608	-0.408009746	0.0008603	0.009226
ENSG00000164379	FOXQ1	5168.478178	7236.742207	-0.485679768	0.000861414	0.009232
ENSG00000205730	ITPRIPL2	1895.671084	2608.892222	-0.460764086	0.00086391	0.009252
ENSG00000068654	POLR1A	6197.5612	4708.597857	0.396490954	0.000865381	0.009261
ENSG00000104447	TRPS1	513.1528839	707.7280964	-0.46473994	0.000865806	0.009261
ENSG00000255050	AC067930.5	153.3671235	78.26565229	0.974238886	0.000867281	0.009268
ENSG00000054967	RELT	574.9927746	384.2979132	0.582547677	0.000868182	0.009268

ENSG00000172819	RARG	2047.690152	2697.747771	-0.398033242	0.00086824	0.009268
ENSG00000140650	PMM2	1121.23983	852.6278778	0.395209826	0.000870751	0.009289
ENSG00000012171	SEMA3B	2445.624615	3669.939356	-0.585600537	0.000873493	0.009312
ENSG00000160058	BSDC1	1971.653634	2528.249346	-0.359051762	0.000876042	0.009331
ENSG00000198356	ASNA1	2064.128613	1556.079407	0.407732898	0.000876861	0.009331
ENSG00000103121	CMC2	840.9375443	579.9546279	0.53643631	0.000876976	0.009331
ENSG00000002016	RAD52	445.4253893	601.3908824	-0.432946076	0.000878749	0.00934
ENSG00000140545	MFGE8	2376.173894	1814.599135	0.388731008	0.000878907	0.00934
ENSG00000105290	APLP1	63.26419006	28.24462784	1.15734334	0.00088112	0.009357
ENSG00000165269	AQP7	4.28900211	25.38832198	-2.555720267	0.000882892	0.00937
ENSG00000182247	UBE2E2	1178.028956	1598.977216	-0.441268568	0.000887686	0.009409
ENSG00000106617	PRKAG2	423.419698	294.525598	0.525061246	0.000887706	0.009409
ENSG00000197905	TEAD4	1135.919541	872.6554349	0.380831271	0.000893379	0.00946
ENSG00000169217	CD2BP2	2349.575027	1793.985066	0.38948658	0.000893755	0.00946
ENSG00000119729	RHOQ	682.8888406	923.3292234	-0.435772194	0.000894887	0.009466
ENSG00000204311	PJVK	44.79716712	98.1525084	-1.1313411	0.000899637	0.00951
ENSG00000146411	SLC2A12	142.7561646	484.4288275	-1.762655201	0.000900412	0.009512
ENSG00000163946	FAM208A	3677.045342	4614.102375	-0.327626059	0.000901729	0.00952
ENSG00000073111	MCM2	4909.359115	3276.939028	0.58328975	0.000904322	0.009541
ENSG00000136155	SCEL	1595.342509	1058.833719	0.590950855	0.000907177	0.009565
ENSG00000185386	MAPK11	281.186145	187.719807	0.58341025	0.000908034	0.009568
ENSG00000104432	IL7	45.2514289	18.10295401	1.320056701	0.000908865	0.009569
ENSG00000182796	TMEM198B	771.7495092	1110.797489	-0.525011053	0.000909285	0.009569
ENSG00000116874	WARS2	471.9505499	635.6921513	-0.43024784	0.000911461	0.009586
ENSG00000159335	PTMS	764.1763487	1024.597281	-0.423798301	0.000912808	0.009594
ENSG00000196787	HIST1H2AG	84.71323233	41.60185146	1.020909829	0.000913947	0.0096
ENSG00000164010	ERMAP	258.1633358	417.1755121	-0.693825068	0.000919888	0.009656
ENSG00000182557	SPNS3	59.09261521	114.3922624	-0.954560664	0.000921127	0.009663
ENSG00000060642	PIGV	333.3491155	503.5274951	-0.596476825	0.000925323	0.009698
ENSG00000177542	SLC25A22	3071.223349	2353.233893	0.384367744	0.000925643	0.009698
ENSG00000143774	GUK1	4551.227827	3470.579575	0.391133808	0.000928111	0.009717
ENSG00000114796	KLHL24	483.7393992	765.4229078	-0.66182206	0.000932417	0.009756
ENSG00000103227	LMF1	105.992352	188.3686934	-0.828468573	0.000936287	0.00979
ENSG00000100263	RHBDD3	807.5315723	592.324875	0.447387086	0.000939252	0.009815
ENSG00000244754	N4BP2L2	2081.540843	2788.397805	-0.421804012	0.000941874	0.009836
ENSG00000041982	TNC	175.0173452	276.6361959	-0.661262414	0.000950018	0.009915
ENSG00000181026	AEN	1896.072279	885.7298893	1.098283252	0.00095366	0.009947
ENSG00000119640	ACYP1	342.0600674	231.7705675	0.562244538	0.000955769	0.009962
ENSG00000177084	POLE	5315.836392	3921.970382	0.438896489	0.000959326	0.009991
ENSG00000167523	SPATA33	265.7243112	161.745675	0.719004121	0.000959801	0.009991
ENSG00000214021	TTLL3	711.9888324	1025.457032	-0.526020088	0.000961236	0.009995
ENSG00000085872	CHERP	2378.597524	1782.73459	0.416121405	0.000961885	0.009995
ENSG00000099953	MMP11	45.27866806	95.40950079	-1.076903304	0.000962894	0.009995

ENSG00000160214	RRP1	1885.462134	1375.581642	0.455248093	0.000963029	0.009995
ENSG00000148834	GSTO1	2273.702913	1756.281497	0.372399222	0.000963179	0.009995
ENSG00000236699	ARHGEF38	97.0933744	171.8206312	-0.824575558	0.000964858	0.010006
ENSG00000006042	TMEM98	225.077946	343.9521669	-0.613258942	0.000966915	0.010021
ENSG00000113272	THG1L	575.9258889	794.2906409	-0.46479274	0.000973522	0.010083
ENSG00000183111	ARHGEF37	81.41871026	142.2746464	-0.807174591	0.000975373	0.010091
ENSG00000108797	CNTNAP1	58.73201107	109.8533531	-0.903021198	0.000975498	0.010091
ENSG00000162576	MXRA8	1365.518999	1855.679792	-0.442409786	0.000979894	0.010124
ENSG00000138172	CALHM2	216.4634182	318.4532838	-0.557502429	0.000979924	0.010124
ENSG00000130544	ZNF557	486.8500004	333.1116588	0.548216977	0.000981454	0.010133
ENSG00000101188	NTSR1	27.77128815	63.60507692	-1.198624155	0.000983262	0.010144
ENSG00000131652	THOC6	628.8276086	828.9273578	-0.398498374	0.000983748	0.010144
ENSG00000136816	TOR1B	801.1968757	595.1433964	0.428069476	0.000984337	0.010144
ENSG00000178209	PLEC	51400.82017	39687.31201	0.373109604	0.000987194	0.010167
ENSG00000280441	FP236383.1	27.0093174	4.923110016	2.470789328	0.000988003	0.010169
ENSG00000160753	RUSC1	1704.863652	1221.883841	0.48087058	0.000991003	0.010193
ENSG00000233532	LINC00460	91.40404246	160.0232923	-0.810432493	0.000993602	0.010213
ENSG00000161395	PGAP3	887.9729738	1205.226494	-0.440869233	0.000995883	0.01023
ENSG00000162604	TM2D1	458.610913	631.7067135	-0.462577097	0.000997771	0.010243
ENSG00000103066	PLA2G15	687.0412471	491.5861637	0.483313415	0.001000623	0.010266
ENSG00000125835	SNRPB	4520.78236	3293.637049	0.456970109	0.001003883	0.010287
ENSG00000078177	N4BP2	743.7146401	547.3192309	0.442174694	0.001003898	0.010287
ENSG00000266010	GATA6-AS1	190.619014	283.800604	-0.572388871	0.001010094	0.010344
ENSG00000070010	UFD1	2755.397401	2034.450974	0.437706545	0.001013242	0.010366
ENSG00000214113	LYRM4	451.5130839	313.9767827	0.523251797	0.00101356	0.010366
ENSG00000088340	FER1L4	1250.872156	2575.940318	-1.042134472	0.001020004	0.010426
ENSG00000108829	LRRC59	6455.138628	4686.945616	0.461841981	0.001022019	0.01043
ENSG00000101928	MOSPD1	868.1119604	640.4816526	0.43789897	0.001022237	0.01043
ENSG00000107902	LHPP	131.087436	218.7938462	-0.738942103	0.001022294	0.01043
ENSG00000172216	CEBPB	1475.989296	2114.044104	-0.518266532	0.00102857	0.010487
ENSG00000124787	RPP40	251.7209551	158.4124451	0.668889696	0.001031118	0.010506
ENSG00000138092	CENPO	1052.063283	695.7062297	0.596996733	0.001034513	0.010525
ENSG00000165140	FBP1	78.32615112	134.5713401	-0.783513565	0.001035622	0.010525
ENSG00000117643	MAN1C1	15.56356216	43.25464797	-1.470641761	0.001035809	0.010525
ENSG00000092841	MYL6	6912.348842	5431.080539	0.347931897	0.001036029	0.010525
ENSG00000172340	SUCLG2	1462.949237	1991.299613	-0.445023208	0.00103617	0.010525
ENSG00000077585	GPR137B	272.3353795	391.6910175	-0.525308972	0.001038159	0.010539
ENSG00000116396	KCNC4	184.0279869	117.8389505	0.644744996	0.001041476	0.010566
ENSG00000164761	TNFRSF11B	27.73027411	62.29217332	-1.170515723	0.00104764	0.010622
ENSG00000131467	PSME3	5610.838828	4126.175228	0.443486911	0.001048425	0.010623
ENSG00000104267	CA2	640.2557196	414.1339077	0.629108578	0.001049884	0.010626
ENSG00000128394	APOBEC3F	588.3506554	846.1916847	-0.525237107	0.001049992	0.010626
ENSG00000165775	FUNDC2	595.6851061	401.141098	0.570149985	0.00105128	0.010632

ENSG00000129347	KRI1	1717.065733	1284.927468	0.418426414	0.001052371	0.010637
ENSG00000132155	RAF1	4482.369176	3598.310815	0.316800583	0.001054257	0.010649
ENSG00000136002	ARHGEF4	62.07968725	112.961416	-0.865989792	0.001057189	0.010667
ENSG00000173917	HOXB2	118.6315911	64.87508505	0.873188488	0.001057709	0.010667
ENSG00000168887	C2orf68	783.6951858	1040.170058	-0.408419578	0.001057999	0.010667
ENSG00000144115	THNSL2	277.8923474	450.1336174	-0.697067952	0.001066554	0.010747
ENSG00000104450	SPAG1	784.3750319	591.1761008	0.407897084	0.001068341	0.010754
ENSG00000117408	IPO13	1178.268196	871.3906271	0.435085474	0.001068574	0.010754
ENSG00000044574	HSPA5	25871.98975	34570.64951	-0.41817509	0.001069899	0.010761
ENSG00000124225	PMEPA1	122.6192612	225.706937	-0.881326768	0.001077412	0.01083
ENSG00000245694	CRNDE	443.2404809	599.3573648	-0.435320417	0.001078943	0.010838
ENSG00000116857	TMEM9	1192.723536	1539.892569	-0.368830256	0.001081722	0.01086
ENSG00000166669	ATF7IP2	66.83565336	116.0579894	-0.796443573	0.001082392	0.01086
ENSG00000184574	LPAR5	20.39390686	52.16188625	-1.358233182	0.001084772	0.010877
ENSG00000115946	PNO1	1555.956488	1088.336882	0.516088039	0.001085945	0.010882
ENSG00000272668	AL590560.1	152.8592864	256.8781575	-0.75057264	0.001088294	0.010899
ENSG00000148204	CRB2	196.166387	312.7988565	-0.674765331	0.00109291	0.010937
ENSG00000071859	FAM50A	1693.471613	1311.287559	0.369420891	0.001093412	0.010937
ENSG00000172115	CYCS	6702.255844	4606.019569	0.541220012	0.001095057	0.010947
ENSG00000173465	SSSCA1	855.8537737	591.416313	0.533065453	0.001095973	0.010949
ENSG00000176619	LMNB2	16659.717	11633.32797	0.518119976	0.001097529	0.010955
ENSG00000115977	AAK1	5095.853392	3983.413096	0.355328571	0.001098161	0.010955
ENSG00000205593	DENND6B	67.7584623	129.3761894	-0.932925242	0.00109854	0.010955
ENSG00000166920	C15orf48	16.6290029	46.81396433	-1.491413176	0.001101752	0.010977
ENSG00000100029	PES1	4245.250296	3067.475303	0.468901724	0.001102151	0.010977
ENSG00000106554	CHCHD3	2143.832109	1568.788546	0.450656925	0.001105292	0.011002
ENSG00000158106	RHPN1	272.878964	410.872082	-0.589047132	0.001109336	0.011035
ENSG00000171241	SHCBP1	1436.461975	953.6568213	0.591119166	0.001116331	0.011098
ENSG00000141696	P3H4	473.8230662	647.0457213	-0.450125357	0.001120653	0.011134
ENSG00000101084	RAB5IF	779.6392907	522.0834782	0.579036315	0.001124181	0.011163
ENSG00000267041	ZNF850	263.2735291	163.3718701	0.689728205	0.001126958	0.011183
ENSG00000155287	SLC25A28	833.1073811	630.4578188	0.402135045	0.001131808	0.011225
ENSG00000105550	FGF21	9.287582936	38.75321861	-2.050733216	0.001136328	0.011263
ENSG00000204175	GPRIN2	301.049939	438.2298384	-0.542180077	0.001137816	0.011271
ENSG00000101152	DNAJC5	3995.310863	3164.647452	0.336297027	0.001143175	0.011317
ENSG00000152292	SH2D6	16.05409587	46.06770823	-1.523411166	0.00114599	0.011338
ENSG00000184752	NDUFA12	1201.967412	902.9090195	0.412530624	0.001148704	0.011358
ENSG00000114554	PLXNA1	5598.759005	4451.436203	0.330910385	0.001153385	0.011397
ENSG00000167674	HDGFL2	2807.774268	2034.232865	0.465137219	0.001154076	0.011397
ENSG00000120254	MTHFD1L	2151.966083	2756.951944	-0.357558542	0.00115921	0.011441
ENSG00000178607	ERN1	806.505127	1169.467833	-0.535988952	0.001166402	0.0115
ENSG00000101347	SAMHD1	869.2441302	562.5265982	0.627830649	0.001166587	0.0115
ENSG00000135632	SMYD5	1558.958653	1168.491974	0.416310492	0.001169108	0.011512

ENSG00000184207	PGP	1839.31248	1322.939044	0.475937047	0.001169198	0.011512
ENSG00000179115	FARSA	2940.652486	2221.143091	0.404901263	0.001172728	0.01154
ENSG00000275481	AC025031.4	18.42253935	48.79449608	-1.402159151	0.001174477	0.01155
ENSG00000126453	BCL2L12	1726.011266	1184.211299	0.54373813	0.001176091	0.011559
ENSG00000250644	AC068580.4	43.15518453	94.4858936	-1.127821144	0.001179359	0.011584
ENSG00000168264	IRF2BP2	2163.406512	2819.406947	-0.382199782	0.001181059	0.011594
ENSG00000177302	TOP3A	1696.724462	1254.089822	0.436373336	0.001183679	0.011612
ENSG00000224597	SVIL-AS1	469.3274107	337.465544	0.476358022	0.001184496	0.011613
ENSG00000146842	TMEM209	1347.142725	1052.231182	0.356153285	0.00118706	0.011632
ENSG00000164099	PRSS12	63.24352774	115.3232799	-0.867688334	0.001191232	0.011666
ENSG00000185361	TNFAIP8L1	718.1458154	527.7296064	0.444363125	0.001195722	0.011702
ENSG00000126215	XRCC3	921.1743184	688.0908386	0.421358412	0.001197067	0.011709
ENSG00000166128	RAB8B	1157.560789	1480.304842	-0.354550455	0.001201561	0.011746
ENSG00000181826	RELL1	223.1574464	145.8839791	0.612946949	0.001205742	0.011779
ENSG00000128951	DUT	2795.297175	2067.4269	0.435237558	0.001212433	0.011838
ENSG00000197915	HRNR	187.8605819	121.1254552	0.633229295	0.001213605	0.011842
ENSG00000138768	USO1	3777.433794	4777.037462	-0.338830897	0.001215209	0.011851
ENSG00000006327	TNFRSF12A	2959.617057	2253.2014	0.393822254	0.001217797	0.011869
ENSG00000281706	LINC01012	89.55989703	149.9840097	-0.742312321	0.0012204	0.011887
ENSG00000196636	SDHAF3	14.25657937	39.69817162	-1.477427671	0.001224238	0.011917
ENSG00000254166	CASC19	1432.95091	2061.711421	-0.524838586	0.001226121	0.01192
ENSG00000182749	PAQR7	24.88874126	62.75513331	-1.33652866	0.00122629	0.01192
ENSG00000033011	ALG1	618.3106019	423.9682595	0.544733119	0.001227259	0.01192
ENSG00000180185	FAHD1	713.0087452	520.8147949	0.453541332	0.001227465	0.01192
ENSG00000068028	RASSF1	703.9493882	487.9587324	0.529304897	0.001229828	0.011936
ENSG00000105063	PPP6R1	3837.461526	3075.887715	0.319124263	0.001230941	0.011937
ENSG00000136982	DSCC1	1237.007087	818.7662927	0.595450766	0.001231391	0.011937
ENSG00000081870	HSPB11	4064.850494	3173.339608	0.357192656	0.001236483	0.01198
ENSG00000272398	CD24	5801.620707	4295.495121	0.433356211	0.001242075	0.012027
ENSG00000104131	EIF3J	4397.955544	3476.575985	0.339196216	0.0012461	0.012058
ENSG00000125779	PANK2	892.3310053	663.2283074	0.428362944	0.001255347	0.012141
ENSG00000275183	LENG9	204.7917306	307.1058431	-0.584771273	0.001262265	0.0122
ENSG00000165905	LARGE2	824.0728517	1197.128037	-0.538140412	0.001264749	0.012211
ENSG00000143217	NECTIN4	460.105411	728.7305678	-0.663683092	0.001264803	0.012211
ENSG00000093183	SEC22C	1479.258679	1151.24744	0.361408379	0.001268228	0.012236
ENSG00000117360	PRPF3	1786.029612	1333.291531	0.422041916	0.001271688	0.012261
ENSG00000095066	HOOK2	2092.723898	2960.700622	-0.500508599	0.001272313	0.012261
ENSG00000181544	FANCB	423.6374188	267.0647366	0.666968867	0.001278315	0.012312
ENSG00000120334	CENPL	704.0102952	513.7890626	0.453760889	0.001282743	0.012347
ENSG00000225177	FLJ46906	72.57554261	136.84945	-0.913792032	0.001285967	0.012371
ENSG00000136279	DBNL	2519.921096	1978.752454	0.348662335	0.001289452	0.012397
ENSG00000189043	NDUFA4	2818.758097	2141.018027	0.396692652	0.001290885	0.012397
ENSG00000267296	CEBPA-DT	22.77270733	54.98941879	-1.272914887	0.001291713	0.012397

ENSG00000205542	TMSB4X	8895.328699	11286.13355	-0.34350182	0.001291723	0.012397
ENSG00000173517	PEAK1	608.3741707	437.0075185	0.47842395	0.001292487	0.012397
ENSG00000185420	SMYD3	292.4457092	424.3996976	-0.538898001	0.001294276	0.012407
ENSG00000157600	TMEM164	940.2653634	717.0022915	0.391011007	0.001296196	0.012418
ENSG00000123836	PFKFB2	1043.904578	1447.60271	-0.471595568	0.001298167	0.01243
ENSG00000103415	HMOX2	1750.134774	1270.194437	0.462719525	0.001307738	0.012514
ENSG00000267169	AC022098.1	45.74303527	86.53048188	-0.919248325	0.001311784	0.012546
ENSG00000076924	XAB2	1992.34322	1578.146644	0.335966432	0.001313104	0.012551
ENSG00000136908	DPM2	1130.111857	845.0208333	0.420041657	0.001316731	0.012578
ENSG00000100994	PYGB	3443.950724	4853.180756	-0.494872216	0.001317463	0.012578
ENSG00000124224	PPP4R1L	337.4058082	481.7970183	-0.513342593	0.001318276	0.012578
ENSG00000169242	EFNA1	868.9439459	1356.597968	-0.642715756	0.001320951	0.012596
ENSG00000142910	TINAGL1	4544.922422	6250.642087	-0.459767501	0.001326314	0.012635
ENSG00000204520	MICA	970.0614911	734.8090797	0.40126363	0.001326515	0.012635
ENSG00000205978	NYNRIN	20.83072431	50.59496809	-1.280890442	0.001329578	0.012655
ENSG00000198624	CCDC69	351.2560789	246.3930673	0.510203258	0.001330151	0.012655
ENSG00000227214	HCG15	43.42269318	83.43751946	-0.942689211	0.001335918	0.012699
ENSG00000100156	SLC16A8	264.5582738	391.6043207	-0.565730513	0.001336713	0.012699
ENSG00000152223	EPG5	1718.546827	2209.932293	-0.363161911	0.00133719	0.012699
ENSG00000089280	FUS	12159.91988	9316.762827	0.384316659	0.001341816	0.012733
ENSG00000188486	H2AFX	5414.401074	3630.398501	0.576715704	0.001342248	0.012733
ENSG00000104613	INTS10	1962.592346	2459.552556	-0.325845518	0.0013452	0.012751
ENSG00000180447	GAS1	25.13533602	6.172383363	2.047380216	0.001345707	0.012751
ENSG00000197601	FAR1	8806.755077	7041.652106	0.322750698	0.001350697	0.012791
ENSG00000100211	CBY1	233.4125734	334.6834557	-0.520783135	0.001352523	0.0128
ENSG00000104689	TNFRSF10A	1469.656314	1129.31566	0.380071972	0.001354688	0.012814
ENSG00000100764	PSMC1	2458.407964	1825.803662	0.42927296	0.001357977	0.012837
ENSG00000169435	RASSF6	193.9516427	122.0574778	0.666357336	0.001362155	0.012869
ENSG00000172270	BSG	19330.14121	15302.4523	0.337094526	0.00137472	0.01298
ENSG00000281912	LINC01144	11.81881003	36.25783171	-1.624521368	0.00137806	0.013004
ENSG00000120162	MOB3B	392.5294327	246.5599185	0.673052495	0.00138057	0.013021
ENSG00000196205	EEF1A1P5	3143.249006	4142.101619	-0.398312171	0.001382591	0.013032
ENSG00000189339	SLC35E2B	2460.924796	3290.70213	-0.419269695	0.001386094	0.013058
ENSG00000135925	WNT10A	107.0376926	188.6979134	-0.818224255	0.001392275	0.013108
ENSG00000151276	MAGI1	1252.107178	1594.607604	-0.349151848	0.001396004	0.013129
ENSG00000196693	ZNF33B	826.4654485	1137.402408	-0.460961665	0.001396048	0.013129
ENSG00000157557	ETS2	5230.163398	7031.634878	-0.42719172	0.001398577	0.013145
ENSG00000104897	SF3A2	2422.1063	1839.063196	0.397508894	0.001403973	0.013173
ENSG00000064419	TNPO3	3768.688041	2956.120688	0.350311208	0.001404299	0.013173
ENSG00000155438	NIFK	1459.935694	1066.652407	0.452997573	0.001404314	0.013173
ENSG00000171160	MORN4	241.9422763	345.7833309	-0.515961322	0.001404785	0.013173
ENSG00000106348	IMPDH1	2473.844993	1835.086086	0.431153078	0.001430434	0.013406
ENSG00000186395	KRT10	496.9474556	327.4768602	0.603277374	0.001434255	0.013434

ENSG00000135469	COQ10A	68.6665344	118.611591	-0.789736665	0.001436741	0.01345
ENSG00000182117	NOP10	1290.092039	941.8313926	0.453967615	0.001438261	0.013456
ENSG00000072858	SIDT1	117.2745615	188.0310009	-0.681328801	0.001444604	0.013508
ENSG00000101049	SGK2	38.74262979	81.79265866	-1.080993719	0.001448358	0.013529
ENSG00000136100	VPS36	1807.639856	2365.435904	-0.388081403	0.001448604	0.013529
ENSG00000122034	GTF3A	3107.75571	2413.234649	0.364969014	0.001449361	0.013529
ENSG00000171448	ZBTB26	490.634221	649.7929794	-0.405375959	0.001451905	0.013545
ENSG00000183873	SCN5A	57.6411659	127.9213456	-1.15001403	0.001453631	0.013553
ENSG00000198691	ABCA4	12.66938571	38.37610843	-1.595696191	0.001457642	0.013579
ENSG00000182732	RGS6	103.0161474	183.5198294	-0.832829125	0.001459712	0.013579
ENSG00000104408	EIF3E	9292.287503	11944.31516	-0.362300404	0.001459862	0.013579
ENSG00000173681	BCLAF3	652.3680424	893.463816	-0.454013927	0.001460082	0.013579
ENSG00000196247	ZNF107	995.6354276	683.3551719	0.543516729	0.00146052	0.013579
ENSG00000234741	GAS5	5227.66152	6657.072063	-0.348723348	0.001479941	0.013752
ENSG00000235027	AC068580.3	103.5043359	162.8970418	-0.654968974	0.001482387	0.013767
ENSG00000116741	RGS2	54.4522933	22.44548101	1.284609245	0.001486442	0.013796
ENSG00000186432	KPNA4	4425.776257	3483.799758	0.345376033	0.00149183	0.013839
ENSG00000132199	ENOSF1	1325.146274	1745.68278	-0.397703314	0.001497196	0.01388
ENSG00000169629	RGPD8	12.45217543	36.92320236	-1.571921031	0.001499847	0.013891
ENSG00000180279	LINC01869	25.88883617	59.62076022	-1.205994946	0.001500161	0.013891
ENSG00000185813	PCYT2	3127.874807	2471.752439	0.339833044	0.001500846	0.013891
ENSG00000115504	EHBP1	1855.610804	2340.37439	-0.335073473	0.001503337	0.013905
ENSG00000136143	SUCLA2	3206.967139	2566.114493	0.321534466	0.001504053	0.013905
ENSG00000205885	C1RL-AS1	455.6849035	671.9528193	-0.560089894	0.001505074	0.013906
ENSG00000228474	OST4	1697.784333	1265.842374	0.423843693	0.001508621	0.013931
ENSG00000173275	ZNF449	202.2258664	302.164153	-0.579133638	0.00151181	0.013949
ENSG00000180425	C11orf71	38.25607613	79.51245114	-1.057759619	0.001512235	0.013949
ENSG00000100298	APOBEC3H	5.539923411	23.6288666	-2.099900566	0.001515372	0.01397
ENSG00000158715	SLC45A3	341.1189413	463.5404937	-0.442600611	0.001517207	0.013979
ENSG00000129255	MPDU1	1455.514469	1088.045945	0.419807072	0.001523953	0.014033
ENSG00000129173	E2F8	930.1418383	650.8263214	0.514657931	0.00153011	0.014082
ENSG00000110237	ARHGEF17	1834.826812	2397.333719	-0.385827067	0.001532397	0.014095
ENSG00000115556	PLCD4	164.3683011	99.86132105	0.723090272	0.001539135	0.014149
ENSG00000129566	TEP1	1777.481415	2281.266431	-0.360135901	0.001544771	0.014193
ENSG00000151233	GXYLT1	1831.81065	1414.466867	0.373360605	0.001546955	0.014203
ENSG00000148400	NOTCH1	347.604319	487.3964935	-0.48595838	0.001547619	0.014203
ENSG00000011304	PTBP1	20469.82233	15949.00175	0.360062485	0.001550216	0.014219
ENSG00000168701	TMEM208	764.9626137	578.7514822	0.401876293	0.001554044	0.014246
ENSG00000160014	CALM3	7970.968581	5809.663851	0.456291722	0.001561702	0.014308
ENSG00000259319	AF111167.2	5.794167683	24.73030456	-2.092800902	0.00156603	0.01434
ENSG00000260855	AL591848.4	48.84120929	95.40201622	-0.968842477	0.001567976	0.01435
ENSG00000179271	GADD45GIP1	2076.308589	1579.810768	0.394369687	0.00156968	0.014357
ENSG00000284976	BX255925.3	915.3047068	696.0127468	0.395465756	0.001575062	0.014398

ENSG00000106636	YKT6	3743.584833	2890.828928	0.372946659	0.001583323	0.014466
ENSG00000260022	AL031716.1	13.08187138	39.04509002	-1.576180165	0.001588854	0.014508
ENSG00000171608	PIK3CD	80.0238846	134.4499462	-0.750280359	0.001593781	0.014545
ENSG00000247077	PGAM5	3556.642236	2500.240743	0.508659177	0.001594706	0.014545
ENSG00000141570	CBX8	526.1324862	701.5787434	-0.414943782	0.001615325	0.014725
ENSG00000152926	ZNF117	742.3631811	555.8602946	0.4170324	0.001620563	0.01476
ENSG00000257556	LINC02298	108.7825655	191.6697447	-0.817431796	0.001620956	0.01476
ENSG00000184743	ATL3	2131.110811	1676.737806	0.345851168	0.001631606	0.014849
ENSG00000149679	CABLES2	520.7673241	367.8961712	0.502684988	0.001636039	0.014881
ENSG00000150051	MKX	21.02436487	53.41150793	-1.339880171	0.001637445	0.014885
ENSG00000116237	ICMT	4156.311506	3101.922365	0.422252753	0.001639486	0.014894
ENSG00000268324	LRRC2-AS1	12.06035208	35.2932079	-1.554731387	0.001641416	0.014894
ENSG00000198816	ZNF358	117.3798487	212.6964513	-0.858650225	0.001641812	0.014894
ENSG00000010310	GIPR	84.40812752	143.9248217	-0.765909702	0.001642075	0.014894
ENSG00000170745	KCNS3	352.7383852	227.5973988	0.631004268	0.001643024	0.014895
ENSG00000120696	KBTBD7	280.2890511	390.9610972	-0.478865368	0.001648072	0.014932
ENSG00000114738	MAPKAPK3	2980.273309	2355.796994	0.339080745	0.00165246	0.014964
ENSG00000168890	TMEM150A	156.7749821	249.5892928	-0.670221248	0.001659251	0.015017
ENSG00000155792	DEPTOR	57.42630085	112.5811461	-0.971785529	0.001662021	0.015034
ENSG00000182054	IDH2	2040.573313	2915.190685	-0.514703271	0.001673461	0.015127
ENSG00000117543	DPH5	509.4002824	678.7815202	-0.415189301	0.001674192	0.015127
ENSG00000008710	PKD1	5069.539726	6892.253067	-0.443108181	0.001680818	0.015178
ENSG00000133216	EPHB2	1314.76076	1857.709137	-0.498680061	0.001682712	0.015187
ENSG00000157014	TATDN2	969.7914816	731.3046584	0.407361178	0.001686766	0.015212
ENSG00000154839	SKA1	1149.170159	849.0086925	0.436503389	0.001687383	0.015212
ENSG00000227500	SCAMP4	2199.654408	1754.07952	0.32640124	0.001690714	0.015234
ENSG00000198933	TBKBP1	138.8696535	210.8387543	-0.601677577	0.001700491	0.01531
ENSG00000136271	DDX56	3245.898095	2379.805079	0.447885372	0.001700974	0.01531
ENSG00000144136	SLC20A1	4318.515165	5504.622006	-0.350124704	0.001705229	0.01534
ENSG00000183763	TRAIP	782.0909691	538.9182761	0.537385223	0.001707004	0.015347
ENSG00000157617	C2CD2	895.575836	1153.082681	-0.364922835	0.00171046	0.01537
ENSG00000165023	DIRAS2	76.67202848	39.5839711	0.952012037	0.001714839	0.0154
ENSG00000224051	CPTP	1076.165359	826.5770252	0.380934303	0.001715707	0.0154
ENSG00000101181	MTG2	1396.897178	1081.362881	0.369821297	0.001720662	0.015428
ENSG00000010030	ETV7	23.24890399	54.92605125	-1.240771502	0.001720755	0.015428
ENSG00000162607	USP1	3780.072623	2889.751763	0.38756583	0.001724642	0.015455
ENSG00000004866	ST7	462.4527032	324.4896412	0.509053459	0.001726478	0.015463
ENSG00000079462	PAFAH1B3	905.4525148	680.646831	0.412217034	0.001747987	0.015647
ENSG00000166546	BEAN1	21.98002786	55.13045491	-1.329258592	0.001750012	0.015656
ENSG00000114933	INO80D	1548.394819	1173.229078	0.400931538	0.001752283	0.015668
ENSG00000183011	NAA38	1277.895387	907.5784669	0.494001844	0.001755618	0.015689
ENSG00000232850	PTGES2-AS1	10.94119508	33.26410068	-1.599680618	0.001758068	0.015703
ENSG00000103044	HAS3	195.9905014	108.8670483	0.851854538	0.001781386	0.015897

ENSG00000164543	STK17A	1383.224782	1093.116436	0.339544197	0.001781745	0.015897
ENSG00000172531	PPP1CA	2832.495305	2228.169175	0.34617777	0.001785789	0.015924
ENSG00000165804	ZNF219	709.9573483	538.4801094	0.398752639	0.001787282	0.015929
ENSG00000104140	RHOV	438.9920931	608.8518694	-0.47306384	0.001788452	0.01593
ENSG00000124733	MEA1	1450.053718	1038.33834	0.482236436	0.001793409	0.015966
ENSG00000101577	LPIN2	784.5081204	1014.051964	-0.370663044	0.001795134	0.015973
ENSG00000183186	C2CD4C	20.06926405	47.42353763	-1.241511722	0.001797265	0.015983
ENSG00000163376	KBTBD8	96.54183719	38.69938754	1.325572014	0.001798481	0.015985
ENSG00000158109	TPRG1L	1263.671706	985.2702158	0.359080125	0.001810128	0.016072
ENSG00000106462	EZH2	2375.363614	1738.308466	0.450783108	0.001810278	0.016072
ENSG00000062822	POLD1	2682.181917	2016.90223	0.411482733	0.001813783	0.016095
ENSG00000108352	RAPGEFL1	1351.306971	1941.004523	-0.522399194	0.001826653	0.016197
ENSG00000011426	ANLN	6200.874307	4675.513253	0.407314723	0.001827317	0.016197
ENSG00000165475	CRYL1	380.7662629	549.5455657	-0.53017851	0.001836201	0.016267
ENSG00000104886	PLEKHJ1	2171.863227	1652.684287	0.394609448	0.001837816	0.016273
ENSG00000188554	NBR1	2867.470296	3940.896729	-0.458925048	0.001846386	0.01634
ENSG00000204271	SPIN3	579.4948446	806.6817256	-0.477968561	0.001847662	0.01634
ENSG00000272068	AL365181.2	168.9580657	268.5167825	-0.669071355	0.001848361	0.01634
ENSG00000128487	SPECC1	1358.07178	1745.188334	-0.362129561	0.001855566	0.016388
ENSG00000112893	MAN2A1	3338.120062	2677.175056	0.318341162	0.001855829	0.016388
ENSG00000171320	ESCO2	1074.89908	733.9299895	0.550660248	0.001856924	0.016389
ENSG00000242950	ERVW-1	85.39102967	142.4148659	-0.736596911	0.001859226	0.0164
ENSG00000276603	AL109614.1	80.48932448	39.89429123	1.009174864	0.001867918	0.016468
ENSG00000101361	NOP56	8427.869699	6166.814443	0.450713033	0.001871048	0.016487
ENSG00000187109	NAP1L1	14551.83159	18301.68481	-0.330826263	0.001877333	0.016533
ENSG00000108515	ENO3	208.0619209	308.8690694	-0.570851618	0.001882107	0.016566
ENSG00000100867	DHRS2	23.04654126	5.082012372	2.148518868	0.001896734	0.016686
ENSG00000114383	TUSC2	982.8654003	709.3584541	0.470809727	0.001912638	0.016817
ENSG00000158545	ZC3H18	2894.765349	2310.39056	0.325446775	0.001914678	0.016823
ENSG00000198917	SPOUT1	1323.664286	974.5482367	0.442446733	0.00191537	0.016823
ENSG00000090273	NUDC	3792.33305	2605.246546	0.541867341	0.001920302	0.016857
ENSG00000109618	SEPSECS	538.091672	707.5583155	-0.395550577	0.001925212	0.016891
ENSG00000146233	CYP39A1	122.5682008	72.88812904	0.751424299	0.00193121	0.016935
ENSG00000167977	KCTD5	1710.258436	1362.181523	0.328171624	0.001945243	0.017048
ENSG00000185963	BICD2	2007.709428	1548.225482	0.375082518	0.001947486	0.017048
ENSG00000105974	CAV1	961.5818866	737.0155094	0.383649272	0.001948143	0.017048
ENSG00000133812	SBF2	2224.997946	3094.921006	-0.476244402	0.001948267	0.017048
ENSG00000092820	EZR	22867.75912	18779.48892	0.28413252	0.00195149	0.017067
ENSG00000170442	KRT86	37.19859549	153.1217749	-2.041450639	0.001955396	0.017092
ENSG00000113356	POLR3G	853.2803633	593.1150466	0.525382447	0.001959647	0.01712
ENSG00000185278	ZBTB37	1113.978019	1446.753876	-0.376523096	0.00196822	0.017185
ENSG00000117877	CD3EAP	262.9762361	161.9157014	0.702137886	0.00198692	0.017339
ENSG00000164951	PDP1	2886.182048	3675.971441	-0.349051686	0.002000648	0.01745

ENSG00000185745	IFIT1	108.9675355	176.3220606	-0.697452361	0.002004188	0.017471
ENSG00000170275	CRTAP	4343.093547	5461.964338	-0.330850114	0.002009057	0.017505
ENSG00000160392	C19orf47	686.1834734	485.1534765	0.500867837	0.002010985	0.017512
ENSG00000185989	RASA3	5.910322377	23.75071159	-2.015606809	0.002013721	0.017527
ENSG00000148019	CEP78	2385.97856	1735.247757	0.459646095	0.002028376	0.017645
ENSG00000182534	MXRA7	24.95913979	55.98010727	-1.169517896	0.002034366	0.017687
ENSG00000226752	CUTALP	537.6872571	753.2385871	-0.486792784	0.002039098	0.017719
ENSG00000133706	LARS	2805.869038	3622.163885	-0.36861309	0.002043601	0.017749
ENSG00000138036	DYNC2LI1	384.2459623	539.2857434	-0.488902457	0.002045906	0.017759
ENSG00000167508	MVD	2238.406559	1756.328329	0.35040997	0.002052526	0.017807
ENSG00000170445	HARS	2618.145672	2045.256428	0.356358629	0.002060783	0.01787
ENSG00000182685	BRICD5	484.6752941	341.2078457	0.507907395	0.002063971	0.017888
ENSG00000260912	AL158206.1	246.3688059	165.8919949	0.570694395	0.002066281	0.017898
ENSG00000149636	DSN1	1058.297264	749.5955526	0.497496944	0.002070064	0.017922
ENSG00000160087	UBE2J2	2064.161194	1619.902635	0.349705427	0.002079175	0.017982
ENSG00000205861	PCOTH	22.30176328	5.787919962	1.960004121	0.002079224	0.017982
ENSG00000167461	RAB8A	1490.945066	1103.817626	0.433917572	0.002087108	0.01804
ENSG00000178449	COX14	383.269851	276.3113863	0.471960615	0.002090852	0.018063
ENSG00000173418	NAA20	2182.406089	1744.043588	0.323455058	0.002104855	0.018175
ENSG00000104765	BNIP3L	2570.250207	3622.312009	-0.495136671	0.002124027	0.018319
ENSG00000279716	AC006128.1	70.38038798	122.0710192	-0.790523619	0.002124089	0.018319
ENSG00000143416	SELENBP1	2252.950394	2886.660703	-0.357567698	0.002124974	0.018319
ENSG00000165916	PSMC3	4221.426819	3140.535166	0.426779597	0.002129776	0.018351
ENSG00000211584	SLC48A1	1166.360072	895.9888345	0.380341508	0.002138569	0.018417
ENSG00000204673	AKT1S1	2811.544089	2142.672203	0.391883679	0.002141402	0.018431
ENSG00000123064	DDX54	5030.782206	3908.481549	0.364193399	0.002142457	0.018431
ENSG00000176087	SLC35A4	2852.579223	2116.781623	0.430454702	0.002164452	0.018611
ENSG00000132846	ZBED3	560.0786248	812.242318	-0.536232635	0.002169871	0.018647
ENSG00000099326	MZF1	912.6021738	1213.00227	-0.410519083	0.002174686	0.018679
ENSG00000246067	RAB30-AS1	197.6866	282.7695099	-0.515120933	0.002176527	0.018685
ENSG00000057657	PRDM1	136.1223404	80.01533704	0.764278661	0.002177648	0.018685
ENSG00000168679	SLC16A4	367.6976042	1094.706705	-1.573982588	0.002188037	0.018764
ENSG00000163950	SLBP	4514.373766	3409.76333	0.404893587	0.002190555	0.018776
ENSG00000135253	KCP	300.6389848	201.8285138	0.574411878	0.002196109	0.018814
ENSG00000175575	PAAF1	469.1997673	634.1257014	-0.435782024	0.002202171	0.018856
ENSG00000171045	TSNARE1	165.6673238	250.3572317	-0.597144897	0.002203749	0.018859
ENSG00000140365	COMMD4	1596.064715	1178.808555	0.43733609	0.00220684	0.018876
ENSG00000285184	AC244033.2	32.86459929	66.42838749	-1.014010981	0.002208248	0.018878
ENSG00000189067	LITAF	1647.347054	2247.249954	-0.448349971	0.00221664	0.01894
ENSG00000135164	DMTF1	1386.464087	1770.8188	-0.353213227	0.002219525	0.018948
ENSG00000233927	RPS28	9173.10289	7444.213616	0.301260937	0.002219957	0.018948
ENSG00000196083	IL1RAP	365.6387561	260.2456351	0.489515752	0.002222199	0.018958
ENSG00000166224	SGPL1	4246.310123	5376.600917	-0.340652356	0.002233789	0.019044

ENSG00000132382	MYBBP1A	4211.785792	3236.553612	0.380205743	0.002234628	0.019044
ENSG00000121210	TMEM131L	1492.173298	1141.977885	0.385938731	0.002236966	0.019054
ENSG00000081026	MAGI3	1665.83584	1266.062637	0.395755182	0.00224365	0.019101
ENSG00000179041	RRS1	1198.725665	822.054088	0.544865739	0.002248428	0.019132
ENSG00000070371	CLTCL1	474.804312	663.7763159	-0.482856819	0.002254831	0.019176
ENSG00000155744	FAM126B	779.8320444	596.8236528	0.386810729	0.002259953	0.01921
ENSG00000074696	HACD3	4505.574144	3577.4094	0.332912556	0.002263235	0.019228
ENSG00000225377	NRSN2-AS1	54.09750394	100.0141951	-0.890100284	0.002282614	0.019382
ENSG00000128641	MYO1B	6026.812374	7350.227569	-0.286432378	0.002286213	0.019403
ENSG00000131389	SLC6A6	6209.195118	5054.436489	0.296883033	0.002288161	0.019409
ENSG00000176915	ANKLE2	3468.463036	2792.68975	0.312758891	0.002300796	0.019498
ENSG00000164362	TERT	108.6987026	174.7305929	-0.68189347	0.002301085	0.019498
ENSG00000163069	SGCB	849.0694166	646.5696995	0.392431677	0.002305402	0.019521
ENSG00000075568	TMEM131	2392.753256	1830.203911	0.386743316	0.002306136	0.019521
ENSG00000250508	AP000808.1	12.47467248	34.1101421	-1.452979096	0.002316582	0.019589
ENSG00000163406	SLC15A2	87.11504035	142.6356967	-0.714313044	0.002316879	0.019589
ENSG00000172638	EFEMP2	88.20210537	166.3003968	-0.916102792	0.002317806	0.019589
ENSG00000171680	PLEKHG5	729.0801089	986.8202996	-0.436340131	0.002326895	0.019656
ENSG00000164181	ELOVL7	774.5952287	599.5971526	0.369459775	0.002333451	0.019701
ENSG00000176974	SHMT1	2398.439125	3049.628446	-0.346627762	0.002338656	0.019732
ENSG00000055163	CYFIP2	1941.07585	1505.855378	0.36621953	0.002340442	0.019732
ENSG00000116138	DNAJC16	1069.291103	1403.261441	-0.392620606	0.002340682	0.019732
ENSG00000164172	MOCS2	1079.800985	836.6526686	0.367503451	0.002345331	0.019761
ENSG00000154582	ELOC	1619.25795	1254.343458	0.36845734	0.002353563	0.01982
ENSG00000176438	SYNE3	114.9315225	177.6967898	-0.629665149	0.002355882	0.019829
ENSG00000205808	PLPP6	320.4334456	434.357044	-0.439456768	0.002362234	0.019872
ENSG00000156675	RAB11FIP1	1688.239769	2323.595969	-0.461046897	0.00236756	0.019907
ENSG00000147459	DOCK5	5561.225972	4481.249089	0.311546757	0.002376982	0.019976
ENSG00000135823	STX6	980.1473395	1254.20159	-0.355718335	0.00238659	0.020046
ENSG00000182154	MRPL41	1678.125572	1286.006218	0.384353121	0.002400165	0.020134
ENSG00000181104	F2R	162.3947883	241.8560939	-0.574670269	0.002401309	0.020134
ENSG00000168765	GSTM4	711.5526288	937.1762576	-0.396979807	0.002401745	0.020134
ENSG00000128563	PRKRIP1	966.7174844	742.930294	0.38017316	0.002401905	0.020134
ENSG00000265778	AC018413.1	33.39033226	70.6202391	-1.081906694	0.002403545	0.020137
ENSG00000169689	CENPX	1566.160266	738.7416494	1.084312781	0.002405941	0.020147
ENSG00000143319	ISG20L2	1765.482671	1264.507949	0.481698706	0.00241057	0.020175
ENSG00000277449	CEBPB-AS1	27.3188611	57.12540186	-1.061561859	0.002418181	0.020229
ENSG00000214078	CPNE1	2069.031576	2553.339105	-0.303448248	0.002422671	0.020256
ENSG00000125841	NRSN2	18.55537631	43.96039567	-1.242854595	0.002426904	0.020281
ENSG00000160401	CFAP157	114.0121831	66.32047801	0.780017445	0.002428608	0.020285
ENSG00000214530	STARD10	2214.360383	2870.679466	-0.374581541	0.002432483	0.020301
ENSG00000112578	BYSL	1175.763446	855.2242586	0.459705498	0.002433046	0.020301
ENSG00000145014	TMEM44	340.1919008	474.7889467	-0.480410028	0.00243973	0.020346

ENSG00000092964	DPYSL2	5965.618299	7468.196542	-0.324162852	0.002447632	0.020395
ENSG00000197191	CYSRT1	170.6114263	271.6145494	-0.670887212	0.002448004	0.020395
ENSG00000090889	KIF4A	2083.769878	1545.270347	0.431094433	0.002450073	0.020401
ENSG00000125454	SLC25A19	332.4377952	201.5203787	0.723732487	0.002451479	0.020403
ENSG00000175274	TP53I11	228.9049261	346.0590854	-0.5965286	0.002456928	0.020438
ENSG00000114054	PCCB	1617.215866	2043.328007	-0.337493485	0.002459412	0.020448
ENSG00000265817	FSBP	17.31317718	47.59398664	-1.45659232	0.00246401	0.020467
ENSG00000134153	EMC7	1100.4193	867.7640963	0.342863769	0.002464251	0.020467
ENSG00000273066	AL355987.4	146.5925398	235.4706269	-0.68293527	0.002472729	0.020527
ENSG00000166262	FAM227B	108.9547816	168.1678206	-0.626301837	0.002481944	0.020593
ENSG00000167996	FTH1	46831.92306	36087.57324	0.376007345	0.002485743	0.020615
ENSG00000117245	KIF17	78.2978393	39.77679956	0.975117398	0.002502462	0.020743
ENSG00000124201	ZNFX1	2026.837787	1594.065386	0.346315984	0.002505726	0.020759
ENSG00000049323	LTBP1	1404.284998	1053.900366	0.413210852	0.002516057	0.020815
ENSG00000263345	AC006435.2	52.86270274	95.80102617	-0.858061274	0.002516075	0.020815
ENSG00000109084	TMEM97	1527.131048	1140.370065	0.421449928	0.002516249	0.020815
ENSG00000127152	BCL11B	35.88763265	13.28324025	1.428470414	0.002532576	0.020939
ENSG00000065357	DGKA	501.6741639	691.6876503	-0.463489919	0.002544997	0.021031
ENSG00000161558	TMEM143	213.3554494	303.3604405	-0.508048139	0.002549511	0.021058
ENSG00000241553	ARPC4	1802.494196	1333.377502	0.43494843	0.002554019	0.021085
ENSG00000148053	NTRK2	125.6843296	73.52363565	0.774098044	0.002555421	0.021085
ENSG00000148926	ADM	451.2835786	308.891957	0.547393157	0.002558212	0.021098
ENSG00000139505	MTMR6	1117.30692	877.1704954	0.348588274	0.002562151	0.021113
ENSG00000099817	POLR2E	6002.415045	4514.571715	0.411023972	0.002562678	0.021113
ENSG00000105227	PRX	149.6386324	227.1427496	-0.602297294	0.002570522	0.021165
ENSG00000145022	TCTA	1132.513176	864.2976346	0.389940435	0.002571542	0.021165
ENSG00000197958	RPL12	31966.84246	38857.49961	-0.281627409	0.002581876	0.021239
ENSG00000050438	SLC4A8	568.0586843	748.2715195	-0.397675596	0.002584018	0.021246
ENSG00000186812	ZNF397	962.9050714	1265.961971	-0.394869783	0.002586255	0.021254
ENSG00000169972	PUSL1	706.8481069	486.4422532	0.539984667	0.002587971	0.021257
ENSG00000099999	RNF215	383.5028191	534.8029227	-0.479863965	0.002589387	0.021258
ENSG00000183010	PYCR1	3397.246486	4259.665209	-0.326315396	0.002600014	0.021327
ENSG00000104936	DMPK	1107.922609	1417.830648	-0.356085124	0.002600299	0.021327
ENSG00000163344	PMVK	382.8992005	271.3549327	0.495997985	0.002625995	0.021527
ENSG00000184220	CMSS1	971.7650837	686.5228601	0.501714942	0.002634091	0.021575
ENSG00000170860	LSM3	2257.239201	1633.53432	0.46662006	0.002635487	0.021575
ENSG00000237719	Z95152.1	42.5536005	16.9753943	1.333087438	0.002635826	0.021575
ENSG00000167074	TEF	828.7053242	1262.644007	-0.607488132	0.002639479	0.021594
ENSG00000116273	PHF13	1042.39783	819.8873693	0.346738358	0.002640809	0.021594
ENSG00000119711	ALDH6A1	661.0815911	847.0486007	-0.357828246	0.002660022	0.02174
ENSG00000215424	MCM3AP-AS1	236.5983387	333.1391054	-0.492625482	0.002664798	0.021768
ENSG00000156976	EIF4A2	12499.85071	15363.34736	-0.297646848	0.00267682	0.021848
ENSG00000187735	TCEA1	2385.587256	2986.060781	-0.324053382	0.002677182	0.021848

ENSG00000107020	PLGRKT	340.3918076	462.754226	-0.44402802	0.00268023	0.021855
ENSG00000164105	SAP30	372.0725252	249.5726808	0.577746283	0.002680715	0.021855
ENSG00000079335	CDC14A	592.2159636	429.1901144	0.464739928	0.00268241	0.021855
ENSG00000237441	RGL2	1494.477832	2100.057979	-0.490920123	0.002683396	0.021855
ENSG00000104228	TRIM35	758.2225262	579.2045706	0.388715378	0.002689082	0.02189
ENSG00000163466	ARPC2	9384.02339	7152.556576	0.391724168	0.002691897	0.021896
ENSG00000086289	EPDR1	3081.037265	2520.498303	0.289624946	0.002692561	0.021896
ENSG00000198561	CTNND1	11998.22748	15269.44008	-0.347873792	0.002693851	0.021896
ENSG00000165816	VWA2	159.6567079	242.5248585	-0.605069196	0.002696826	0.02191
ENSG00000187325	TAF9B	1273.951542	969.3711618	0.393628935	0.002699298	0.021919
ENSG00000148356	LRSAM1	1440.73778	1829.201468	-0.344652782	0.002702679	0.021935
ENSG00000129911	KLF16	1247.217124	909.1219625	0.457055889	0.002706929	0.021959
ENSG00000111319	SCNN1A	7816.22652	10649.96444	-0.446324279	0.002716733	0.022023
ENSG00000145912	NHP2	1975.825851	1459.271937	0.437550044	0.002718334	0.022023
ENSG00000075234	TTC38	706.0346544	522.8239972	0.43417253	0.002718925	0.022023
ENSG00000277161	PIGW	706.8630578	480.8874074	0.55650491	0.002724421	0.022057
ENSG00000228065	LINC01515	16.56314658	42.90995059	-1.378967739	0.002733894	0.02212
ENSG00000189221	MAOA	63.21154646	30.9210053	1.030616449	0.002734865	0.02212
ENSG00000123395	ATG101	694.8064643	510.9940113	0.444273194	0.002736282	0.02212
ENSG00000176209	SMIM19	340.1689308	492.9013098	-0.535494746	0.002750175	0.022222
ENSG00000059769	DNAJC25	277.2939297	189.5261189	0.549223946	0.002752313	0.022228
ENSG00000162144	CYB561A3	938.1496991	701.1363601	0.420440078	0.002753665	0.022228
ENSG00000130768	SMPDL3B	667.7655655	869.3579986	-0.381438435	0.002756826	0.022242
ENSG00000232112	TMA7	408.0191784	266.8135335	0.614050052	0.002761558	0.022269
ENSG00000196338	NLGN3	31.05254584	68.54337306	-1.14157116	0.002765606	0.022288
ENSG00000128595	CALU	6605.085607	5251.870427	0.330610363	0.0027666	0.022288
ENSG00000144711	IQSEC1	1215.975355	1568.711097	-0.367958725	0.002770056	0.022305
ENSG00000109323	MANBA	1097.48485	1450.206935	-0.402488826	0.002790551	0.022459
ENSG00000152582	SPEF2	42.17814882	79.58240098	-0.91985509	0.002793236	0.02247
ENSG00000128228	SDF2L1	1226.649411	918.2327954	0.418338297	0.0027962	0.022482
ENSG00000161996	WDR90	3216.438109	2358.350562	0.447956643	0.002798902	0.022493
ENSG00000010404	IDS	2029.551973	1618.361125	0.326240153	0.002801568	0.022494
ENSG00000082153	BZW1	12885.41225	10003.76317	0.365169812	0.002801791	0.022494
ENSG00000243927	MRPS6	753.6731545	556.4498334	0.437928715	0.002806782	0.022521
ENSG00000255112	CHMP1B	868.3995336	1151.397838	-0.407145008	0.0028084	0.022521
ENSG00000165171	METTL27	128.0607849	197.4561228	-0.62702836	0.002809216	0.022521
ENSG00000177570	SAMD12	575.5917994	767.0477513	-0.414512276	0.00281477	0.022554
ENSG00000186854	TRABD2A	48.75173159	95.46222578	-0.965838646	0.002817851	0.022568
ENSG00000065183	WDR3	2722.699483	2039.09884	0.41720135	0.00282886	0.022645
ENSG00000165282	PIGO	1823.343475	2314.854871	-0.344499127	0.002852653	0.022824
ENSG00000025434	NR1H3	260.219787	370.8989155	-0.512538708	0.00287113	0.022961
ENSG00000137331	IER3	2649.870114	3437.257043	-0.375429404	0.00287528	0.022974
ENSG00000099901	RANBP1	5693.526	3946.183106	0.528952895	0.002875644	0.022974

ENSG00000170270	GON7	276.0149481	174.3633561	0.663493436	0.002890659	0.023083
ENSG00000173163	COMMD1	574.3796503	433.8161984	0.40434216	0.002892474	0.023086
ENSG00000214413	BBIP1	373.9850131	499.1952316	-0.416132986	0.002900159	0.023136
ENSG00000159388	BTG2	94.50078154	162.2340886	-0.777317707	0.002905355	0.023163
ENSG00000164308	ERAP2	2006.396846	2624.794873	-0.388001302	0.00290632	0.023163
ENSG00000104524	PYCR3	1120.751744	740.7460453	0.59804185	0.002910903	0.023188
ENSG00000168275	COA6	441.9463111	317.7570441	0.476179551	0.002918915	0.02324
ENSG00000137364	TPMT	1169.797137	902.6797904	0.373658505	0.002922701	0.023259
ENSG00000102317	RBM3	5146.667093	4162.59025	0.306253889	0.002926383	0.023277
ENSG00000149476	TKFC	1818.153126	2268.111235	-0.319026589	0.002928472	0.023283
ENSG00000163803	PLB1	33.19505687	67.16025693	-1.013318231	0.00294148	0.023375
ENSG00000144647	POMGNT2	629.0375833	467.910018	0.427038224	0.002953511	0.023459
ENSG00000164885	CDK5	663.7299939	511.2213656	0.376657273	0.002965863	0.023545
ENSG00000011083	SLC6A7	16.12923652	39.13693963	-1.280366577	0.002975828	0.023613
ENSG00000147174	GCNA	78.95370175	130.3844814	-0.723686109	0.002977235	0.023613
ENSG00000176485	PLA2G16	513.177164	674.6849978	-0.394900012	0.002990167	0.023704
ENSG00000157800	SLC37A3	1391.574377	1079.404573	0.365954778	0.003002179	0.023788
ENSG00000097021	ACOT7	2276.164355	1671.595184	0.445589395	0.003023003	0.023938
ENSG00000149115	TNKS1BP1	3089.0182	3961.317086	-0.358718669	0.003025052	0.023938
ENSG00000173511	VEGFB	244.2384349	336.9747872	-0.464010832	0.003025494	0.023938
ENSG00000100243	CYB5R3	3834.124023	3103.785133	0.304730624	0.00302847	0.023941
ENSG00000180902	D2HGDH	1218.441688	1689.615083	-0.471334749	0.003028876	0.023941
ENSG00000109255	NMU	1268.893896	855.9619584	0.567960472	0.003035589	0.023983
ENSG00000070778	PTPN21	409.8251065	301.5976654	0.441513831	0.003042083	0.024022
ENSG00000168300	PCMTD1	473.1357115	662.0200528	-0.48517966	0.003052648	0.024094
ENSG00000146410	MTFR2	570.4376256	396.1584483	0.526120128	0.003061166	0.02415
ENSG00000109452	INPP4B	569.799015	781.9393661	-0.457352745	0.003065028	0.024168
ENSG00000102384	CENPI	1014.650015	689.9334845	0.556287108	0.003067086	0.024173
ENSG00000232600	TONSL-AS1	65.41573386	31.48584823	1.057632317	0.003071073	0.024193
ENSG00000167183	PRR15L	1782.503713	2481.222338	-0.477448077	0.003075528	0.024216
ENSG00000188186	LAMTOR4	1105.703817	826.4553705	0.42033836	0.003085692	0.024285
ENSG00000223396	RPS10P7	165.5217846	247.6223768	-0.581433208	0.003093622	0.024326
ENSG00000174243	DDX23	4816.089575	3559.865897	0.436067133	0.003096832	0.024326
ENSG00000104142	VPS18	2110.278704	1666.312452	0.340415728	0.003097351	0.024326
ENSG00000119508	NR4A3	198.1677012	118.6024903	0.742538865	0.003097359	0.024326
ENSG00000204650	LINC02210	1188.962524	1510.57868	-0.34506089	0.003098337	0.024326
ENSG00000160767	FAM189B	971.8786865	1220.389536	-0.328142039	0.00310392	0.024358
ENSG00000166130	IKBIP	322.1125212	202.5933803	0.66822319	0.003108992	0.024386
ENSG00000213699	SLC35F6	2228.060651	1763.507537	0.337140873	0.003111844	0.024397
ENSG00000187531	SIRT7	2062.471872	1618.434957	0.35031323	0.003114091	0.024402
ENSG00000188897	AC099489.1	30.32919224	71.06079109	-1.230222426	0.003118769	0.024427
ENSG00000050327	ARHGEF5	1847.784428	1490.749819	0.309861951	0.003122291	0.024443
ENSG00000258947	TUBB3	195.8614446	118.6521851	0.726436821	0.003139642	0.024567

ENSG00000279233	AC122688.3	418.290174	551.7569558	-0.399088515	0.003146226	0.024597
ENSG00000164749	HNF4G	82.93948544	134.0209124	-0.694270596	0.003146471	0.024597
ENSG00000196407	THEM5	6.466712406	23.81327966	-1.872418757	0.003148069	0.024598
ENSG00000171105	INSR	486.7064817	350.5557085	0.472698591	0.003153239	0.024627
ENSG00000274386	TMEM269	52.69339395	95.40836435	-0.854162632	0.003155641	0.024634
ENSG00000017797	RALBP1	4816.20301	3877.870837	0.312565348	0.003165915	0.024702
ENSG00000215039	CD27-AS1	81.65153015	147.0473723	-0.850123408	0.003180201	0.024802
ENSG00000278828	HIST1H3H	42.42076354	17.77792742	1.259852445	0.003183629	0.024815
ENSG00000188191	PRKAR1B	552.2005884	382.6868471	0.530205957	0.003185572	0.024815
ENSG00000185495	AC138393.1	233.2832134	337.7783825	-0.53316473	0.003186645	0.024815
ENSG00000244625	MIATNB	55.15947512	95.57817378	-0.794982903	0.003188458	0.024815
ENSG00000188976	NOC2L	6717.873707	4937.781898	0.444217794	0.003189548	0.024815
ENSG00000268903	AL627309.6	39.13369107	72.96285479	-0.899608739	0.0031976	0.024859
ENSG00000189223	PAX8-AS1	1120.265436	1411.316883	-0.332732815	0.003198169	0.024859
ENSG00000105185	PDCD5	1684.938751	1266.196053	0.412676865	0.003206075	0.024908
ENSG00000167553	TUBA1C	9586.435333	7168.179308	0.419349885	0.003238449	0.025132
ENSG00000047597	XK	855.3373767	668.5397983	0.35490195	0.003238678	0.025132
ENSG00000273002	AL355388.2	14.88122261	38.06517726	-1.353749682	0.003239415	0.025132
ENSG00000157916	RER1	3366.29415	2739.988463	0.297031552	0.003247605	0.025183
ENSG00000117748	RPA2	1626.972025	1168.763245	0.477358268	0.003249104	0.025183
ENSG00000261156	LINC01989	92.95710952	50.48782954	0.881099178	0.003264301	0.025289
ENSG00000198843	SELENOT	1998.24357	1590.494336	0.329115	0.003272577	0.025341
ENSG00000003987	MTMR7	76.58816567	40.95150705	0.906767162	0.003277913	0.02537
ENSG00000168924	LETM1	4760.871066	3607.581253	0.400320793	0.003282748	0.025395
ENSG00000056558	TRAF1	372.6964956	538.0937064	-0.529477027	0.003284992	0.025401
ENSG00000180979	LRRC57	743.2439175	568.1456092	0.38819166	0.003287598	0.025409
ENSG00000103489	XYLT1	23.05128337	60.87096439	-1.401513977	0.003311913	0.025585
ENSG00000129951	PLPPR3	61.47223678	28.49989302	1.108805537	0.003313762	0.025586
ENSG00000100299	ARSA	458.351616	611.9814951	-0.417250273	0.003315151	0.025586
ENSG00000130255	RPL36	10928.56867	8792.914654	0.313771271	0.0033237	0.02564
ENSG00000132646	PCNA	7276.611653	5204.635601	0.483506325	0.003326626	0.02565
ENSG00000174669	SLC29A2	1241.23005	1614.736149	-0.378859759	0.003329724	0.025656
ENSG00000260877	AP005233.2	30.86201256	66.58731839	-1.104508786	0.003330544	0.025656
ENSG00000174442	ZWILCH	1882.49963	1267.755	0.570404683	0.003337409	0.025697
ENSG00000197150	ABCB8	2047.564054	1641.666449	0.319083796	0.003340984	0.025712
ENSG00000197019	SERTAD1	475.311039	340.2063245	0.483510725	0.003350875	0.025776
ENSG00000186638	KIF24	702.1465601	497.0405275	0.497709954	0.003364251	0.025857
ENSG00000102531	FNDC3A	1410.162969	1799.713668	-0.352341106	0.003364571	0.025857
ENSG00000130726	TRIM28	16294.25332	12679.5339	0.361885612	0.003368744	0.02587
ENSG00000106031	HOXA13	3271.463482	4022.295166	-0.298254473	0.003369417	0.02587
ENSG00000196072	BLOC1S2	1077.320729	847.9438133	0.345431555	0.003375487	0.025904
ENSG00000074621	SLC24A1	406.0712699	606.5864408	-0.579806645	0.003376933	0.025904
ENSG00000182087	TMEM259	5516.150888	4517.962402	0.288098919	0.003382627	0.025926

ENSG00000197776	KLHDC1	6.117737826	25.05939321	-2.029283943	0.003383351	0.025926
ENSG00000139579	NABP2	1486.51403	1074.359617	0.468724203	0.003384634	0.025926
ENSG00000066044	ELAVL1	4159.897292	3157.736961	0.39777506	0.003392234	0.025972
ENSG00000125037	EMC3	892.6076949	687.7364448	0.37594164	0.003402149	0.026036
ENSG00000100056	ESS2	656.263149	495.1064564	0.406308593	0.003404364	0.026041
ENSG00000178234	GALNT11	1207.968322	940.1394261	0.360917194	0.003425594	0.026191
ENSG00000267355	RPL9P29	15.69532646	40.80441404	-1.381457148	0.003428653	0.026202
ENSG00000030419	IKZF2	191.3529245	283.9461705	-0.570700918	0.003447015	0.02633
ENSG00000165271	NOL6	1944.776065	1398.227404	0.476490161	0.003454992	0.026369
ENSG00000168411	RFWD3	3579.056064	2647.264811	0.435157308	0.003455377	0.026369
ENSG00000197989	SNHG12	1296.977259	900.933743	0.526334994	0.003461696	0.026405
ENSG00000075975	MKRN2	1206.577432	1504.888727	-0.319065732	0.003468288	0.026435
ENSG00000131495	NDUFA2	699.5515703	518.5586133	0.432619277	0.003469369	0.026435
ENSG00000080298	RFX3	213.8552674	304.8358859	-0.511846976	0.003474089	0.026435
ENSG00000108379	WNT3	244.1936924	345.0264304	-0.498340324	0.003476811	0.026435
ENSG00000127837	AAMP	3405.325527	2683.347919	0.343921373	0.003478101	0.026435
ENSG00000185808	PIGP	273.3312944	378.1463809	-0.469414932	0.003479698	0.026435
ENSG00000143878	RHOB	8758.567428	6798.979641	0.365320816	0.003480005	0.026435
ENSG00000164970	FAM219A	897.0525274	696.508034	0.36507128	0.003480624	0.026435
ENSG00000215375	MYL5	557.1001931	767.8557285	-0.462768738	0.003481012	0.026435
ENSG00000278771	RN7SL3	317.5801208	212.6770849	0.580767782	0.003482687	0.026435
ENSG00000005700	IBTK	3247.35037	4182.390436	-0.365083652	0.003483419	0.026435
ENSG00000184178	SCFD2	508.4481406	350.420731	0.536976301	0.003489649	0.02647
ENSG00000002586	CD99	4963.480581	4064.146527	0.28833086	0.003492994	0.026483
ENSG00000152503	TRIM36	363.2556274	258.9253326	0.489606196	0.003503941	0.026554
ENSG00000131504	DIAPH1	9308.140135	7150.517222	0.380473237	0.003512208	0.026604
ENSG00000217801	AL390719.1	451.2301729	623.7553906	-0.467321879	0.003517875	0.026635
ENSG00000163507	CIP2A	1882.660868	1295.020017	0.539967408	0.003519917	0.026638
ENSG00000116161	CACYBP	4837.040989	3370.143472	0.521379314	0.003529109	0.026695
ENSG00000232434	AJM1	639.3243958	876.71088	-0.455499181	0.003534556	0.02671
ENSG00000168246	UBTD2	771.7897605	1007.129072	-0.384502506	0.003534773	0.02671
ENSG00000231181	AL954705.1	78.51183159	126.654499	-0.691000635	0.003535983	0.02671
ENSG00000065717	TLE2	21.38604167	51.63006715	-1.27220379	0.003538807	0.026719
ENSG00000260279	AC137932.1	314.9582542	436.3034879	-0.47005953	0.003542981	0.026738
ENSG00000175334	BANF1	3598.751637	2619.774885	0.45815735	0.003546146	0.02675
ENSG00000157212	PAXIP1	1588.876373	1209.5933	0.393633917	0.003551519	0.026769
ENSG00000254635	WAC-AS1	468.8116208	641.2187188	-0.452380191	0.003551947	0.026769
ENSG00000280734	LINC01232	237.2883278	328.5880556	-0.470411675	0.003563768	0.026846
ENSG00000113916	BCL6	458.8918343	619.6067275	-0.433468005	0.003566717	0.026855
ENSG00000172375	C2CD2L	832.4593305	643.1302922	0.371979293	0.00357019	0.026857
ENSG00000258088	AC078820.1	123.1280604	187.4644847	-0.608963231	0.003571226	0.026857
ENSG00000158055	GRHL3	1828.381561	2444.308394	-0.419315901	0.00357187	0.026857
ENSG00000126106	TMEM53	240.036965	333.8047388	-0.475272112	0.003579132	0.026894

ENSG00000283154	IQCJ-SCHIP1	121.6888028	74.99531025	0.698395679	0.003580037	0.026894
ENSG00000127328	RAB3IP	989.140396	1230.646547	-0.315257326	0.003586851	0.026933
ENSG00000104067	TJP1	6016.519926	7354.044001	-0.289705499	0.003593911	0.026973
ENSG00000050344	NFE2L3	2521.639051	3296.482601	-0.386702488	0.003614482	0.027115
ENSG00000172428	COPS9	590.2416586	392.7348668	0.588528303	0.00362508	0.027182
ENSG00000164796	CSMD3	42.9869484	17.32696523	1.297307631	0.003646279	0.027329
ENSG00000152270	PDE3B	28.54728535	9.646329592	1.571453664	0.003650955	0.027351
ENSG00000140398	NEIL1	234.2430563	349.1045041	-0.574949599	0.003664574	0.027435
ENSG00000118894	EEF2KMT	530.2169802	374.7180538	0.502092504	0.003665673	0.027435
ENSG00000141084	RANBP10	1480.782183	1157.859094	0.354269042	0.003669516	0.027435
ENSG00000261150	EPPK1	500.6113932	380.5945476	0.394923254	0.003670171	0.027435
ENSG00000121316	PLBD1	662.8650597	929.9332084	-0.488755448	0.003670587	0.027435
ENSG00000130816	DNMT1	9824.565962	7116.313839	0.465304125	0.00367217	0.027435
ENSG00000147955	SIGMAR1	3762.929316	2800.545073	0.4263669	0.003675533	0.027447
ENSG00000187554	TLR5	28.32477079	57.95702809	-1.031773529	0.003684039	0.027498
ENSG00000213801	ZNF321P	123.6841398	186.4333164	-0.591654557	0.00369568	0.027566
ENSG00000085982	USP40	1862.48546	2400.484038	-0.366278701	0.003696556	0.027566
ENSG00000241258	CRCP	778.960569	576.3390612	0.435123086	0.003698189	0.027566
ENSG00000049246	PER3	242.9554733	386.8137839	-0.67069575	0.003705203	0.027606
ENSG00000176624	MEX3C	1832.877676	2279.038349	-0.31440689	0.003709712	0.027615
ENSG00000166321	NUDT13	103.0190548	159.5492939	-0.629900612	0.003709835	0.027615
ENSG00000148090	AUH	482.035865	642.3930608	-0.414682921	0.003716561	0.027653
ENSG00000135722	FBXL8	382.1907319	513.6288621	-0.425343401	0.003719495	0.027659
ENSG00000198720	ANKRD13B	752.052234	576.4611689	0.384343203	0.003720786	0.027659
ENSG00000166557	TMED3	1734.491291	1359.201218	0.351338081	0.003728221	0.027702
ENSG00000139618	BRCA2	1362.898211	937.0458826	0.540872462	0.003740287	0.027779
ENSG00000095203	EPB41L4B	1453.479451	1884.286418	-0.374790024	0.003752964	0.02786
ENSG00000176101	SSNA1	1045.588603	803.4094454	0.380123551	0.003757226	0.027879
ENSG00000138160	KIF11	4742.166682	3568.791765	0.410078525	0.00376489	0.027923
ENSG00000168539	CHRM1	31.69861346	65.79672597	-1.056644074	0.003775724	0.027985
ENSG00000125995	ROMO1	798.4096589	598.395914	0.417086387	0.003776604	0.027985
ENSG00000042286	AIFM2	2987.139554	2452.669367	0.284408512	0.003778979	0.02799
ENSG00000130363	RSPH3	354.6258382	526.9442945	-0.571400126	0.003793571	0.028085
ENSG00000129514	FOXA1	5343.086224	6921.112848	-0.373457177	0.003796902	0.028085
ENSG00000196878	LAMB3	6157.142997	4501.210763	0.451911113	0.003798075	0.028085
ENSG00000150995	ITPR1	48.09352392	21.00428148	1.203322863	0.003798711	0.028085
ENSG00000233369	GTF2IP4	511.9886812	690.2125989	-0.431676266	0.00380776	0.028139
ENSG00000250328	MGC32805	301.624846	433.7955932	-0.524994353	0.003818963	0.028209
ENSG00000121542	SEC22A	264.5754592	185.6236928	0.509358056	0.003820609	0.028209
ENSG00000196937	FAM3C	2521.633226	2036.729385	0.307883802	0.003825433	0.028232
ENSG00000100578	KIAA0586	514.9636722	718.9302921	-0.481044655	0.003846613	0.028375
ENSG00000074582	BCS1L	952.2474823	701.3832384	0.441701342	0.003857737	0.028444
ENSG00000156273	BACH1	1231.751926	1651.533955	-0.423232623	0.003866562	0.028497

ENSG00000162913	OBSCN-AS1	27.89851024	62.72152699	-1.165793053	0.003868532	0.028498
ENSG00000083457	ITGAE	372.7463942	255.1280348	0.549206905	0.003879218	0.028564
ENSG00000149922	TBX6	16.03159883	39.93570192	-1.319007964	0.003886609	0.028606
ENSG00000259863	SH3RF3-AS1	42.10010079	77.44609812	-0.883649815	0.003888922	0.02861
ENSG00000204843	DCTN1	3588.167614	2948.646721	0.283259989	0.003894995	0.028636
ENSG00000165983	PTER	1423.605963	1791.757569	-0.332088812	0.003895949	0.028636
ENSG00000166451	CENPN	1057.708582	777.3597596	0.444462277	0.003916236	0.028754
ENSG00000160352	ZNF714	782.1322488	553.6795962	0.498803928	0.003918287	0.028754
ENSG00000152056	AP1S3	761.2571301	567.8331647	0.423331732	0.003919557	0.028754
ENSG00000151881	TMEM267	444.6102128	602.794269	-0.439987641	0.003920698	0.028754
ENSG00000083750	RRAGB	256.1079431	382.7241807	-0.579803465	0.003922797	0.028754
ENSG00000135404	CD63	6793.47932	5654.998934	0.264639027	0.003922894	0.028754
ENSG00000197457	STMN3	155.1057604	231.441319	-0.575809215	0.003924395	0.028754
ENSG00000205763	RP9P	277.2438829	196.2417082	0.49811115	0.003927556	0.028765
ENSG00000106546	AHR	2089.53657	2726.648398	-0.384336944	0.003932511	0.028788
ENSG00000179361	ARID3B	175.4975736	116.6526963	0.58948108	0.003938014	0.028815
ENSG00000152104	PTPN14	3485.110652	2841.353718	0.294757936	0.003942613	0.028834
ENSG00000133466	C1QTNF6	620.0320835	931.8394012	-0.588258072	0.003944087	0.028834
ENSG00000153551	CMTM7	607.322046	775.3477898	-0.35273033	0.003960037	0.028938
ENSG00000125319	C17orf53	413.7683004	299.8023195	0.464737938	0.00396541	0.028964
ENSG00000099783	HNRNPM	11651.81353	8495.997326	0.455708485	0.003970067	0.028985
ENSG00000165156	ZHX1	796.3819783	621.2794725	0.358694222	0.003980853	0.029051
ENSG00000065970	FOXJ2	1454.068975	1174.92919	0.307411227	0.004010996	0.029258
ENSG00000262468	LINC01569	109.0812417	173.8905952	-0.67265912	0.004015563	0.029278
ENSG00000173960	UBXN2A	976.9628683	760.5765066	0.361711886	0.004018448	0.029286
ENSG00000090615	GOLGA3	4611.707372	3715.411245	0.311831253	0.004022158	0.029289
ENSG00000131470	PSMC3IP	429.1729624	284.4874536	0.594084741	0.004022413	0.029289
ENSG00000128534	LSM8	1411.52133	1126.083994	0.326006586	0.004037303	0.029384
ENSG00000167114	SLC27A4	1684.608138	1343.425895	0.326526633	0.004043031	0.029413
ENSG00000229589	ACVR2B-AS1	21.1317974	47.32404355	-1.163714068	0.004046999	0.02942
ENSG00000104979	C19orf53	1993.041591	1562.447185	0.351119541	0.004047584	0.02942
ENSG00000143353	LYPLAL1	802.0610941	1092.453936	-0.446271623	0.004049848	0.029423
ENSG00000148841	ITPRIP	429.7583746	321.0288709	0.421562161	0.004068717	0.029547
ENSG00000087053	MTMR2	2331.805731	1880.74394	0.30984868	0.004073797	0.029571
ENSG00000100097	LGALS1	187.0960144	309.6925696	-0.728157934	0.004088564	0.029665
ENSG00000165724	ZMYND19	1626.434618	1164.604195	0.482408653	0.00410157	0.029746
ENSG00000112118	MCM3	7287.106087	5281.378026	0.4644953	0.004103344	0.029746
ENSG00000103534	TMC5	698.8726413	892.027613	-0.352647994	0.004108658	0.029752
ENSG00000141527	CARD14	1006.741493	1273.758105	-0.339665226	0.004109252	0.029752
ENSG00000175220	ARHGAP1	2326.448492	1879.388284	0.307772215	0.004109718	0.029752
ENSG00000130529	TRPM4	1725.716431	2210.193429	-0.356705682	0.004116409	0.029788
ENSG00000196154	S100A4	325.6833633	456.6904089	-0.48949934	0.004131487	0.029883
ENSG00000146731	CCT6A	15261.89429	11545.58172	0.402606363	0.004141368	0.029942

ENSG00000212237	RNA5SP18	14.26239415	36.38373879	-1.351685079	0.004146259	0.029964
ENSG00000078399	HOXA9	1674.605281	2194.542674	-0.390547857	0.004182969	0.030216
ENSG00000141295	SCRN2	628.3686939	818.3381443	-0.380972776	0.004186049	0.030225
ENSG00000236287	ZBED5	2088.69323	2575.263155	-0.302368813	0.004191263	0.030249
ENSG00000284691	AC073111.5	213.2464337	306.469143	-0.52372997	0.004199975	0.030298
ENSG00000179277	MEIS3P1	18.12437364	43.68352196	-1.267326005	0.00420926	0.030352
ENSG00000157778	PSMG3	1582.341977	1219.421928	0.376309513	0.004220794	0.030422
ENSG00000104047	DTWD1	616.3591132	786.016899	-0.351254195	0.004232812	0.030495
ENSG00000165410	CFL2	1507.361222	1042.517613	0.532233872	0.004238542	0.030511
ENSG00000214944	ARHGEF28	140.032473	221.5356032	-0.664329492	0.004240004	0.030511
ENSG00000137857	DUOX1	48.86207151	88.81188292	-0.860469585	0.004240607	0.030511
ENSG00000166912	MTMR10	658.5558307	864.0687978	-0.392549009	0.004247054	0.030537
ENSG00000107175	CREB3	1586.384798	1238.156067	0.357299994	0.004247947	0.030537
ENSG00000081138	CDH7	32.00132139	10.86603015	1.549743674	0.004253622	0.030564
ENSG00000119574	ZBTB45	1151.145447	1425.528187	-0.30829779	0.00425767	0.030575
ENSG00000102401	ARMCX3	719.3296078	920.7375878	-0.356743939	0.004258887	0.030575
ENSG00000168301	KCTD6	166.8753963	109.401837	0.611289055	0.004282645	0.030732
ENSG00000172534	HCFC1	5951.845157	4848.905386	0.29567793	0.004310414	0.030918
ENSG00000102977	ACD	1151.267735	896.9207579	0.360232214	0.004315606	0.030941
ENSG00000110442	COMMD9	831.274931	618.971253	0.425028193	0.004328256	0.031019
ENSG00000170779	CDCA4	1330.659172	966.3400972	0.46165507	0.004346877	0.031116
ENSG00000169241	SLC50A1	1186.570799	1572.508695	-0.406600683	0.004347493	0.031116
ENSG00000077063	CTTNBP2	200.4773757	287.2989417	-0.520098572	0.004347585	0.031116
ENSG00000170122	FOXD4	54.14698858	22.43048332	1.282312044	0.004355824	0.031161
ENSG00000137198	GMPR	28.85631855	57.86114494	-1.004842277	0.004360268	0.03118
ENSG00000137393	RNF144B	33.55673367	63.46749184	-0.918559849	0.004380424	0.03131
ENSG00000215912	TTC34	49.91190264	92.73731675	-0.895878848	0.004388052	0.031351
ENSG00000138688	KIAA1109	3330.77629	4161.903136	-0.321429812	0.004394703	0.031382
ENSG00000133315	MACROD1	534.0192147	717.4324161	-0.425116349	0.004396176	0.031382
ENSG00000111832	RWDD1	1255.42578	995.4641807	0.334627143	0.004403509	0.03142
ENSG00000011258	MBTD1	732.0160239	958.1439007	-0.388380346	0.004415876	0.031486
ENSG00000153363	LINC00467	217.183354	304.4284035	-0.488940617	0.004417961	0.031486
ENSG00000137710	RDX	4480.628633	3620.613484	0.30727679	0.004418502	0.031486
ENSG00000105058	FAM32A	1881.164662	1468.213819	0.357469075	0.004424847	0.031517
ENSG00000075914	EXOSC7	1043.344808	827.4097879	0.334609886	0.004431014	0.031537
ENSG00000159714	ZDHHC1	92.15881517	154.7790579	-0.748992316	0.004431442	0.031537
ENSG00000102030	NAA10	788.4978915	579.8297373	0.444722243	0.004446315	0.031616
ENSG00000134690	CDCA8	2545.628722	1814.787171	0.488127843	0.004446437	0.031616
ENSG00000167895	TMC8	270.4566043	392.18119	-0.53523921	0.004484845	0.031875
ENSG00000134697	GNL2	2671.512713	2047.74062	0.383751678	0.00448891	0.03189
ENSG00000154122	ANKH	792.0365524	616.4013537	0.361835611	0.004491421	0.031894
ENSG00000248508	SRP14-AS1	105.0835695	161.2235105	-0.620251829	0.004505331	0.031973
ENSG00000102763	VWA8	1408.384901	1734.269486	-0.300491624	0.004506443	0.031973

ENSG00000003989	SLC7A2	1238.999695	921.5413712	0.427732965	0.004528417	0.032115
ENSG00000085999	RAD54L	1335.048402	939.02094	0.507992103	0.004530857	0.032119
ENSG00000056972	TRAF3IP2	773.6234973	1040.23688	-0.427967661	0.004534301	0.032129
ENSG00000162927	PUS10	559.830779	716.8042817	-0.357133406	0.004540761	0.032151
ENSG00000136830	FAM129B	9480.682382	7880.794385	0.266620505	0.004541372	0.032151
ENSG00000225489	AL354707.1	194.475541	302.2769127	-0.637901745	0.00458325	0.032434
ENSG00000171159	C9orf16	1114.638276	881.8405692	0.337335636	0.004596395	0.032513
ENSG00000113719	ERGIC1	1726.99806	2135.570257	-0.306452916	0.004603552	0.032549
ENSG00000139182	CLSTN3	1148.953807	1585.907419	-0.465262636	0.004622448	0.032669
ENSG00000105048	TNNT1	40.52213979	85.74127142	-1.079226952	0.004625294	0.032675
ENSG00000159173	TNNI1	35.94660153	69.65503279	-0.9506983	0.004630527	0.032698
ENSG00000186185	KIF18B	2931.670084	2130.068552	0.460936682	0.004633257	0.032703
ENSG00000138162	TACC2	2545.588671	3396.111199	-0.416022998	0.004647797	0.032791
ENSG00000213024	NUP62	2470.411803	1937.982174	0.350053956	0.004654114	0.032822
ENSG00000113494	PRLR	17.18722766	45.80840951	-1.406608364	0.004661336	0.032856
ENSG00000181381	DDX60L	329.4514336	464.2320585	-0.49518087	0.004663048	0.032856
ENSG00000166471	TMEM41B	3015.382749	3910.985432	-0.375207809	0.004666152	0.032863
ENSG00000095752	IL11	52.56744443	26.10490248	1.013559646	0.004668014	0.032863
ENSG00000160633	SAFB	4288.403653	3394.731616	0.337313211	0.004670749	0.032868
ENSG00000105926	MPP6	922.9963075	702.2826426	0.395127987	0.004678344	0.032907
ENSG00000063244	U2AF2	7956.425805	6174.608252	0.365825287	0.004697197	0.033026
ENSG00000118503	TNFAIP3	168.8520165	109.3942668	0.622569181	0.004717408	0.033142
ENSG00000164920	OSR2	161.5574253	237.7626171	-0.558872428	0.004719027	0.033142
ENSG00000132481	TRIM47	803.7327933	614.7266135	0.387608379	0.0047198	0.033142
ENSG00000138413	IDH1	7150.086532	8951.979738	-0.32426208	0.004724876	0.033163
ENSG00000173918	C1QTNF1	133.9329489	199.2294108	-0.573337118	0.004738786	0.033247
ENSG00000105676	ARMC6	1358.530051	1024.875725	0.406978639	0.004742809	0.033249
ENSG00000241288	AC092902.2	20.45925268	46.31864863	-1.176710239	0.004743148	0.033249
ENSG00000161980	POLR3K	558.9934677	373.5370571	0.58242481	0.004754525	0.033305
ENSG00000100399	CHADL	35.36006495	65.67790942	-0.892942437	0.004755217	0.033305
ENSG00000279878	AP003108.5	40.4319517	73.86111117	-0.870223724	0.004765887	0.033365
ENSG00000178802	MPI	896.6128986	1146.741204	-0.355426068	0.004778787	0.033441
ENSG00000197063	MAFG	2789.650901	2268.515192	0.298677368	0.004785174	0.033472
ENSG00000224888	AC138028.2	105.9441989	58.81767315	0.854487012	0.00478759	0.033474
ENSG00000204314	PRRT1	12.00082104	32.01479879	-1.416558929	0.004800556	0.033551
ENSG00000063660	GPC1	4660.486795	3821.013993	0.286506743	0.004808838	0.033564
ENSG00000140406	TLNRD1	2467.641195	2018.450769	0.290022068	0.004809169	0.033564
ENSG00000132424	PNISR	5058.900195	6212.678934	-0.296439416	0.004809751	0.033564
ENSG00000118482	PHF3	3162.824096	3900.432769	-0.302557483	0.004810634	0.033564
ENSG00000105618	PRPF31	1088.301503	841.7201476	0.37094788	0.004816945	0.03359
ENSG00000106078	COBL	2085.975013	1674.171645	0.317143926	0.004819506	0.03359
ENSG00000102265	TIMP1	2450.596376	1942.624145	0.334800512	0.004820576	0.03359
ENSG00000251474	RPL32P3	641.4312413	815.198527	-0.345783061	0.004845949	0.033752

ENSG00000175324	LSM1	549.0909847	397.3936263	0.466522391	0.004860564	0.03384
ENSG00000120438	TCP1	13913.7587	10078.3465	0.46528649	0.004866259	0.033865
ENSG00000138180	CEP55	2874.45219	2189.764857	0.392354686	0.004873855	0.033903
ENSG00000101004	NINL	70.90637252	120.8671901	-0.770425527	0.004888519	0.033991
ENSG00000011275	RNF216	1574.109559	1279.314392	0.298998763	0.004890638	0.033991
ENSG00000260401	AP002761.4	10.28425997	29.02842098	-1.499545367	0.00489909	0.034036
ENSG00000184428	TOP1MT	869.9549083	1090.892111	-0.326103912	0.004915308	0.034134
ENSG00000170043	TRAPPC1	1246.83881	930.817864	0.421516022	0.004918639	0.034142
ENSG00000116001	TIA1	3135.639662	3867.872817	-0.30301067	0.004931305	0.034216
ENSG00000148459	PDSS1	550.3297659	364.3971387	0.595129559	0.004940327	0.034264
ENSG00000119280	C1orf198	3719.66738	3036.503113	0.292782476	0.004943871	0.034274
ENSG00000237187	NR2F1-AS1	106.9842869	65.4967875	0.707823669	0.004952915	0.034322
ENSG00000132661	NXT1	536.8372362	376.5041392	0.51296947	0.004966874	0.034404
ENSG00000265972	TXNIP	1435.684566	238.879532	2.587664685	0.004994076	0.034578
ENSG00000230989	HSBP1	2664.014915	2058.941868	0.3717322	0.005009901	0.034661
ENSG00000264717	NPY4R2	24.53793195	56.06170656	-1.198106355	0.005010319	0.034661
ENSG00000137841	PLCB2	36.79008987	72.70862324	-0.984235704	0.005025178	0.034749
ENSG00000110871	COQ5	741.1435824	936.4358853	-0.337941915	0.005032224	0.034783
ENSG00000175582	RAB6A	2313.042259	2953.890665	-0.353057537	0.005037612	0.034806
ENSG00000170515	PA2G4	6228.699739	4550.761131	0.452884343	0.005053675	0.034902
ENSG00000157152	SYN2	7.358864298	25.1701427	-1.782659574	0.005061796	0.034943
ENSG00000181513	ACBD4	742.6237433	984.8924796	-0.407516906	0.005084489	0.035085
ENSG00000125885	MCM8	2160.940741	1596.725038	0.436773803	0.005086677	0.035085
ENSG00000010278	CD9	8767.989605	11253.01993	-0.360060358	0.005113609	0.035256
ENSG00000153207	AHCTF1	6318.306215	5057.480076	0.321226	0.005118581	0.035264
ENSG00000168939	SPRY3	137.0329577	216.2104548	-0.659474441	0.005119109	0.035264
ENSG00000163001	CFAP36	729.440927	929.5494512	-0.349888091	0.005142748	0.035408
ENSG00000110455	ACCS	501.5249036	717.990635	-0.517508969	0.005144239	0.035408
ENSG00000014257	ACPP	298.3194565	418.4965131	-0.489334032	0.005152314	0.035448
ENSG00000196396	PTPN1	2905.245098	2388.358796	0.282476115	0.005167403	0.035537
ENSG00000100325	ASCC2	2207.997298	1790.906304	0.301754746	0.005180361	0.035601
ENSG00000176095	IP6K1	1554.182238	1196.233642	0.37797021	0.005180993	0.035601
ENSG00000183023	SLC8A1	29.82595631	59.80175078	-1.003164515	0.005188103	0.035635
ENSG00000172927	MYEOV	1093.952196	1412.552396	-0.368676948	0.005192677	0.035651
ENSG00000155760	FZD7	1095.745562	1446.844885	-0.401332754	0.00520485	0.03572
ENSG00000116141	MARK1	386.0368946	511.9610108	-0.407606749	0.005207379	0.035722
ENSG00000177721	ANXA2R	83.74486713	130.18382	-0.638305226	0.005210441	0.035728
ENSG00000267374	AC016205.1	155.3283266	96.66015791	0.68503108	0.005216146	0.035752
ENSG00000078487	ZCWPW1	72.37955682	118.8901094	-0.717522353	0.005235262	0.035868
ENSG00000180822	PSMG4	508.5687342	390.773787	0.380399444	0.005243632	0.035901
ENSG00000136824	SMC2	5780.684113	4420.046435	0.387129813	0.005245261	0.035901
ENSG00000055609	KMT2C	4715.7084	3884.752145	0.279592231	0.005246666	0.035901
ENSG00000115109	EPB41L5	1083.898529	1337.905151	-0.303727564	0.005256154	0.03593

ENSG00000124383	MPHOSPH10	2307.99474	1831.810026	0.333023097	0.005256415	0.03593
ENSG00000105855	ITGB8	128.6229897	193.08475	-0.584153751	0.005257501	0.03593
ENSG00000162910	MRPL55	767.7855189	590.764088	0.378567074	0.005264783	0.035965
ENSG00000125977	EIF2S2	5921.71379	7251.398052	-0.292302155	0.005274332	0.036015
ENSG00000165912	PACSIN3	108.8341364	169.777853	-0.641858695	0.005278203	0.036027
ENSG00000130313	PGLS	870.4324809	1127.206629	-0.373293853	0.005294004	0.036119
ENSG00000171862	PTEN	2799.673325	3523.85016	-0.332161416	0.005321794	0.036267
ENSG00000144485	HES6	308.9605029	201.044414	0.622398134	0.005322724	0.036267
ENSG00000232233	LINC02043	36.87502535	16.00357716	1.201791093	0.005323261	0.036267
ENSG00000065618	COL17A1	754.9826824	495.9359415	0.605272355	0.005326142	0.036267
ENSG00000143147	GPR161	252.5116375	341.8428423	-0.436579745	0.005330465	0.036267
ENSG00000174885	NLRP6	171.5779568	246.2649598	-0.52073949	0.005330549	0.036267
ENSG00000189180	ZNF33A	1272.073921	1575.715565	-0.308679126	0.005331161	0.036267
ENSG00000137807	KIF23	3166.74497	2381.033806	0.411241794	0.005343557	0.036336
ENSG00000071553	ATP6AP1	2622.250214	2148.64375	0.287174626	0.005353968	0.036392
ENSG00000084774	CAD	6954.356196	5524.935054	0.33205498	0.005379966	0.036553
ENSG00000102901	CENPT	916.8071132	702.0350391	0.385397195	0.005384128	0.036566
ENSG00000163617	CCDC191	131.6226016	210.6247113	-0.677569936	0.005386914	0.03657
ENSG00000160131	VMA21	2241.375735	1745.232462	0.361174127	0.005392449	0.036593
ENSG00000124098	FAM210B	602.3454001	459.9520728	0.387622518	0.005395004	0.036595
ENSG00000215790	SLC35E2A	479.2901351	640.446004	-0.418561318	0.005403441	0.036637
ENSG00000249592	AC139887.2	226.0846768	315.5319468	-0.479670136	0.005418298	0.036722
ENSG00000263327	TAPT1-AS1	31.50788029	12.5516906	1.32853525	0.005424021	0.036746
ENSG00000105373	NOP53	3505.215894	4393.734639	-0.326030372	0.005432834	0.03679
ENSG00000111358	GTF2H3	1611.844904	1991.18186	-0.305071788	0.005440007	0.036824
ENSG00000101000	PROCR	521.0379917	684.7502726	-0.394632877	0.005461148	0.036937
ENSG00000101442	ACTR5	647.4227781	506.8857692	0.352961981	0.005461211	0.036937
ENSG00000213523	SRA1	1040.02622	827.3164078	0.330486393	0.005474101	0.037008
ENSG00000143891	GALM	114.3774777	174.9759733	-0.614786184	0.005478671	0.03701
ENSG00000140307	GTF2A2	1052.670733	751.5896829	0.486240924	0.005478888	0.03701
ENSG00000129351	ILF3	20921.81051	16269.78168	0.362833189	0.00549188	0.037066
ENSG00000177042	TMEM80	1037.764143	1375.928238	-0.406745715	0.005493685	0.037066
ENSG00000160094	ZNF362	320.316877	493.3780721	-0.623359252	0.00549391	0.037066
ENSG00000145832	SLC25A48	7.997282416	24.97731424	-1.64324377	0.005502497	0.037108
ENSG00000123131	PRDX4	1686.602927	1285.097581	0.392464377	0.00553251	0.037295
ENSG00000099139	PCSK5	113.5139999	171.6299575	-0.596517309	0.005553173	0.037419
ENSG00000047634	SCML1	633.5694517	456.7833185	0.473380123	0.005556405	0.037426
ENSG00000100221	JOSD1	2784.103253	2292.750101	0.28006698	0.005579794	0.037568
ENSG00000186951	PPARA	429.6252271	562.2916112	-0.388053705	0.005586592	0.037593
ENSG00000265763	ZNF488	161.9445066	99.42432291	0.706416539	0.005588125	0.037593
ENSG00000243449	C4orf48	591.2284301	423.261244	0.483381223	0.005598944	0.03765
ENSG00000214160	ALG3	1238.112611	990.7275225	0.321217836	0.005637658	0.037884
ENSG00000165474	GJB2	2063.795981	1676.375336	0.299574782	0.005638398	0.037884

ENSG00000100410	PHF5A	1386.739888	1005.756865	0.463641663	0.005655351	0.037983
ENSG00000174744	BRMS1	1162.094676	879.8986231	0.401399706	0.005660596	0.038002
ENSG00000104413	ESRP1	995.514502	1317.96012	-0.404860123	0.005663753	0.038008
ENSG00000139722	VPS37B	1414.588123	1129.501067	0.324351215	0.005667875	0.03802
ENSG00000213413	PVRIG	55.65449938	96.82702453	-0.796297549	0.005676433	0.038062
ENSG00000140553	UNC45A	2581.722718	2083.150977	0.309713017	0.005685065	0.0381
ENSG00000119314	PTBP3	9308.65374	11258.82785	-0.2744347	0.005686812	0.0381
ENSG00000271533	Z83843.1	109.8773907	175.12899	-0.671216644	0.005692397	0.038122
ENSG00000116863	ADPRHL2	892.303204	700.8540405	0.348239662	0.005697451	0.038124
ENSG00000007520	TSR3	1216.695253	972.7791288	0.3228206	0.005698127	0.038124
ENSG00000123358	NR4A1	596.9817104	777.0577656	-0.379980256	0.005699768	0.038124
ENSG00000119688	ABCD4	805.2539166	1003.20128	-0.317029424	0.005729528	0.038308
ENSG00000180098	TRNAU1AP	677.6384714	529.8599666	0.355639296	0.005743397	0.038385
ENSG00000110900	TSPAN11	41.53442643	75.71302719	-0.867942197	0.005754173	0.038441
ENSG00000106538	RARRES2	182.7623288	264.2765171	-0.532024696	0.005783942	0.038615
ENSG00000229950	TFAP2A-AS1	21.08496858	44.8952868	-1.088150561	0.005784975	0.038615
ENSG00000196670	ZFP62	1002.389179	1254.475611	-0.323983946	0.005793558	0.038657
ENSG00000159921	GNE	2610.177443	3291.028911	-0.334501024	0.005805177	0.038719
ENSG00000152127	MGAT5	9647.137561	11739.10087	-0.283189403	0.005810331	0.038737
ENSG00000166503	HDGFL3	1011.502452	815.2767495	0.310818099	0.005821858	0.038798
ENSG00000089248	ERP29	4042.189228	4856.699711	-0.264893886	0.005832781	0.038855
ENSG00000143294	PRCC	2140.368758	1723.954412	0.311883234	0.005836809	0.038866
ENSG00000138621	PPCDC	541.0222384	402.8039546	0.425477583	0.00585324	0.03896
ENSG00000185818	NAT8L	137.2748103	203.1763047	-0.562952911	0.005873714	0.039066
ENSG00000152700	SAR1B	1571.039867	1240.804399	0.340684179	0.005873923	0.039066
ENSG00000140941	MAP1LC3B	1819.203009	1458.535323	0.318331028	0.005885856	0.039129
ENSG00000204389	HSPA1A	86.01394888	38.66291267	1.15872733	0.005892139	0.039155
ENSG00000145779	TNFAIP8	398.0214922	541.8820253	-0.4456921	0.005902574	0.039193
ENSG00000013583	HEBP1	1059.654745	839.6455544	0.335399819	0.005902684	0.039193
ENSG00000179409	GEMIN4	2688.982533	1957.413974	0.458314754	0.005907792	0.039211
ENSG00000010626	LRRC23	175.1319167	251.3422705	-0.522583625	0.00591781	0.039262
ENSG00000128578	STRIP2	427.4835888	298.7286783	0.51782401	0.005938509	0.039383
ENSG00000267246	AC091132.5	39.71890343	78.14465249	-0.972886668	0.005942105	0.039391
ENSG00000276952	AL121772.3	23.12693452	53.73695711	-1.214388107	0.005970467	0.039551
ENSG00000112208	BAG2	1670.792518	1261.516182	0.405663978	0.005971601	0.039551
ENSG00000175567	UCP2	93.36744986	150.4240534	-0.692355806	0.005976359	0.039551
ENSG00000171865	RNASEH1	1253.561646	976.4713133	0.360656242	0.005977029	0.039551
ENSG00000198824	CHAMP1	1706.82669	1359.066212	0.328529684	0.005978315	0.039551
ENSG00000105248	YJU2	790.4842173	612.0462636	0.368232551	0.00598637	0.039588
ENSG00000053438	NNAT	150.9747998	92.48065243	0.711753639	0.005993002	0.039616
ENSG00000184983	NDUFA6	1441.2418	1119.33828	0.364867312	0.005998062	0.039633
ENSG00000163479	SSR2	2170.064653	2664.910537	-0.2966381	0.006022487	0.039779
ENSG00000185418	TARSL2	423.1182768	308.3084788	0.45561533	0.006027952	0.039799

ENSG00000139428	MMAB	637.2038049	810.8547779	-0.347730085	0.00605264	0.039945
ENSG00000124766	SOX4	6283.20442	7730.98005	-0.299214133	0.006067053	0.040024
ENSG00000131374	TBC1D5	2055.74323	2619.965269	-0.34995076	0.006069959	0.040027
ENSG00000189195	BTBD8	332.5108798	457.0597718	-0.459907198	0.006087513	0.040127
ENSG00000059378	PARP12	1473.829495	1200.600223	0.295674062	0.006097002	0.040173
ENSG00000198729	PPP1R14C	238.2649637	165.1284854	0.529593224	0.006122197	0.040323
ENSG00000078070	MCCC1	418.3810725	555.3402867	-0.408945716	0.006138065	0.040411
ENSG00000128709	HOXD9	55.79738274	97.253641	-0.804118451	0.006144911	0.040432
ENSG00000107140	TESK1	908.6093988	718.6420851	0.338643162	0.006148707	0.040432
ENSG00000103365	GGA2	3189.735249	2565.549345	0.314381455	0.006150364	0.040432
ENSG00000285533	AP001362.2	42.3085889	75.67944942	-0.83711796	0.006151051	0.040432
ENSG00000182795	C1orf116	1833.436507	2455.738643	-0.421810064	0.006168513	0.04053
ENSG00000075218	GTSE1	2289.585287	1700.384857	0.429171065	0.006173223	0.040545
ENSG00000258404	LINC02320	8.072423065	24.84856716	-1.626931188	0.006179463	0.04057
ENSG00000103363	ELOB	4273.639393	3538.866569	0.272267356	0.006185308	0.040592
ENSG00000084444	FAM234B	235.1921941	165.2219797	0.510388445	0.006205254	0.040692
ENSG00000204789	ZNF204P	276.9034681	377.9936097	-0.449283875	0.006207278	0.040692
ENSG00000063180	CA11	184.3183924	277.1317268	-0.58861626	0.006208118	0.040692
ENSG00000142669	SH3BGRL3	2625.256041	2167.409876	0.276385284	0.006212877	0.0407
ENSG00000135452	TSPAN31	299.847319	431.1453089	-0.524401591	0.006214219	0.0407
ENSG00000088881	EBF4	30.45059955	62.68476088	-1.038935595	0.006235144	0.040808
ENSG00000113360	DROSHA	2611.507646	2071.55993	0.334052942	0.006235678	0.040808
ENSG00000117395	EBNA1BP2	2743.924287	2060.00392	0.413676102	0.006249864	0.040884
ENSG00000122299	ZC3H7A	1849.784603	2264.71981	-0.29222556	0.006266706	0.040964
ENSG00000196526	AFAP1	1445.323697	1173.674644	0.300678044	0.006267157	0.040964
ENSG00000100523	DDHD1	1036.387672	1320.238186	-0.348495597	0.006272242	0.040981
ENSG00000168538	TRAPPC11	1764.012366	2181.711473	-0.306950137	0.006304175	0.041174
ENSG00000112367	FIG4	682.104462	911.3587268	-0.418391947	0.006309164	0.04119
ENSG00000230454	U73166.1	92.72898008	146.0076287	-0.654126839	0.006334755	0.04134
ENSG00000005075	POLR2J	775.5742615	605.9440774	0.356253065	0.006338964	0.041351
ENSG00000135047	CTSL	1303.938529	1050.370108	0.311564403	0.006345306	0.041376
ENSG00000133313	CNDP2	4530.071845	3659.210213	0.30810468	0.006348493	0.04138
ENSG00000136560	TANK	860.2052444	1119.546689	-0.380685738	0.006371315	0.041504
ENSG00000139112	GABARAPL1	2250.154801	1842.141131	0.288406516	0.006372512	0.041504
ENSG00000089486	CDIP1	73.53498575	39.84629826	0.888773513	0.006378919	0.041529
ENSG00000056097	ZFR	4285.270531	3311.249907	0.371950464	0.006385161	0.041553
ENSG00000111667	USP5	4982.523591	3925.761381	0.343936021	0.006390911	0.041574
ENSG00000170855	TRIAP1	621.1208492	440.5982425	0.495837243	0.006394164	0.041579
ENSG00000258498	DIO3OS	226.6965071	329.5585175	-0.539505983	0.006403221	0.041621
ENSG00000105939	ZC3HAV1	2599.5198	2116.86616	0.296344524	0.006415635	0.041685
ENSG00000133019	CHRM3	72.58248172	39.62073721	0.872341106	0.006422106	0.041711
ENSG00000162062	TEDC2	892.5798936	622.8097709	0.519854216	0.006429974	0.041745
ENSG00000143977	SNRPG	3510.804841	2571.382359	0.449295406	0.00644164	0.041789

ENSG00000116704	SLC35D1	890.2236419	709.1675027	0.327806121	0.006441922	0.041789
ENSG00000123144	TRIR	3282.417577	2620.404036	0.325062594	0.006467282	0.041937
ENSG00000143321	HDGF	14549.63468	11743.05794	0.309180645	0.006471547	0.041948
ENSG00000153814	JAZF1	62.93556721	31.40828247	1.011140357	0.006481245	0.041995
ENSG00000188910	GJB3	1047.311519	818.3748248	0.354877412	0.006489062	0.042029
ENSG00000088298	EDEM2	547.579583	709.6744375	-0.374939111	0.006493679	0.042042
ENSG00000205339	IPO7	18675.44031	22674.90432	-0.279990517	0.006496873	0.042046
ENSG00000136950	ARPC5L	1815.608026	1344.275517	0.433934027	0.006524525	0.042208
ENSG00000122971	ACADS	379.4569348	499.8910487	-0.397763218	0.006527872	0.042213
ENSG00000215271	HOMEZ	410.1239309	547.9374538	-0.417853397	0.006531063	0.042217
ENSG00000137409	MTCH1	4360.097801	3499.111336	0.317469187	0.00655496	0.042344
ENSG00000149573	MPZL2	975.3737808	1285.626816	-0.398987221	0.006555845	0.042344
ENSG00000275549	STPG3-AS1	22.0585864	47.26792642	-1.094634442	0.006590952	0.042554
ENSG00000108592	FTSJ3	2568.563013	1977.42946	0.377441919	0.006608118	0.042648
ENSG00000051108	HERPUD1	2440.273333	3391.9898	-0.475111804	0.006617782	0.042693
ENSG00000128191	DGCR8	2035.432667	1580.19406	0.365787122	0.006635639	0.042792
ENSG00000099785	2-Mar	474.575383	358.8589506	0.401501402	0.006655803	0.042905
ENSG00000180233	ZNRF2	155.0631632	103.9608434	0.57717635	0.006661392	0.042924
ENSG00000170260	ZNF212	440.9387665	337.864554	0.38483293	0.006686541	0.043054
ENSG00000101596	SMCHD1	5590.761901	4699.492766	0.250514883	0.006686837	0.043054
ENSG00000170836	PPM1D	529.9152484	393.3700518	0.430214095	0.006695024	0.043075
ENSG00000260822	AC004656.1	239.6669283	323.5944703	-0.433694753	0.006695352	0.043075
ENSG00000181458	TMEM45A	52.19418974	20.89021231	1.314306586	0.006718491	0.043207
ENSG00000148248	SURF4	6199.517437	5060.640025	0.292762986	0.006726317	0.04324
ENSG00000123374	CDK2	1789.186726	1292.028982	0.469728945	0.006731315	0.043254
ENSG00000260528	FAM157C	28.82693406	59.00373088	-1.032980807	0.006733748	0.043254
ENSG00000178980	SELENOW	1443.864457	1086.075517	0.411228888	0.006755584	0.043377
ENSG00000123219	CENPK	1225.951913	913.5148505	0.424525562	0.006768079	0.04344
ENSG00000111144	LTA4H	1848.254884	2327.825252	-0.333128389	0.006777228	0.043478
ENSG00000186073	C15orf41	421.4671655	315.674281	0.416714038	0.006779324	0.043478
ENSG00000232973	CYP1B1-AS1	391.8172947	557.759608	-0.510318135	0.006787588	0.043514
ENSG00000135821	GLUL	520.1199249	674.0596826	-0.374554668	0.006792192	0.043526
ENSG00000168078	PBK	2533.464265	1921.765735	0.398666181	0.006808711	0.043615
ENSG00000129474	AJUBA	4386.026492	5397.393116	-0.299304998	0.006816289	0.043647
ENSG00000241360	PDXP	84.63625696	44.42861307	0.93379166	0.006840939	0.043774
ENSG00000168646	AXIN2	12306.0269	15010.75289	-0.286701807	0.006843333	0.043774
ENSG00000117036	ETV3	1266.843993	1035.091744	0.291711102	0.00684416	0.043774
ENSG00000176340	COX8A	1145.735275	868.3632173	0.40019978	0.006850006	0.043794
ENSG00000144320	LNPK	1203.568698	898.4067925	0.421703937	0.006856481	0.043807
ENSG00000142623	PADI1	282.7965461	825.3095436	-1.545141474	0.006858701	0.043807
ENSG00000154102	C16orf74	10.22182154	27.84590913	-1.449861706	0.006860055	0.043807
ENSG00000159753	CARMIL2	61.84049042	33.29335368	0.897869165	0.006871083	0.04386
ENSG00000275437	AL121832.3	226.9776497	153.3271415	0.56886911	0.00688036	0.043902

ENSG00000203880	PCMTD2	2016.365703	2469.546625	-0.292669186	0.006887533	0.043931
ENSG00000127528	KLF2	1291.658793	977.6684766	0.401038185	0.006915585	0.044093
ENSG00000076003	MCM6	5413.208697	4066.743862	0.41269429	0.006921715	0.044114
ENSG00000116191	RALGPS2	1347.186063	1698.2334	-0.333920807	0.006925385	0.044121
ENSG00000275371	AC012645.4	47.65741687	91.6728048	-0.940466392	0.006935964	0.044171
ENSG00000244045	TMEM199	210.4216938	148.4270963	0.502503201	0.006943061	0.044184
ENSG00000169727	GPS1	3502.952788	2760.432405	0.343796097	0.006943499	0.044184
ENSG00000167779	IGFBP6	127.2421388	190.9140681	-0.583853734	0.006971745	0.044347
ENSG00000114790	ARHGEF26	717.1223506	556.8724164	0.365261516	0.00698046	0.044385
ENSG00000111653	ING4	273.5531952	383.6229643	-0.48911513	0.00698363	0.044388
ENSG00000113811	SELENOK	808.0797433	624.005912	0.372805118	0.00699178	0.044423
ENSG00000165115	KIF27	167.7278068	234.0341023	-0.479426747	0.007002314	0.044465
ENSG00000169136	ATF5	477.2777751	338.8130875	0.494139513	0.007003904	0.044465
ENSG00000188681	TEKT4P2	44.07090613	21.11142003	1.068876299	0.007009395	0.044483
ENSG00000130520	LSM4	5231.145811	4123.431554	0.343277903	0.00701585	0.044496
ENSG00000113328	CCNG1	3235.201737	4061.194277	-0.328200949	0.007016963	0.044496
ENSG00000204394	VARS	8180.394755	6656.44142	0.297491932	0.007069828	0.044814
ENSG00000171067	C11orf24	1349.526993	1022.458212	0.400346972	0.007078588	0.044852
ENSG00000181016	LSMEM1	252.6683548	170.6429085	0.569828469	0.007086344	0.044884
ENSG00000131165	CHMP1A	3143.786758	2516.303368	0.321230458	0.007110143	0.045017
ENSG00000144802	NFKBIZ	799.4205837	615.8755933	0.376625368	0.00711835	0.045052
ENSG00000183520	UTP11	1743.571726	1401.844181	0.314660878	0.007121972	0.045057
ENSG00000182580	EPHB3	619.9046757	826.9441346	-0.41690957	0.007133279	0.045111
ENSG00000105953	OGDH	4677.81374	3886.266433	0.267352983	0.007139244	0.045132
ENSG00000150990	DHX37	1799.253619	1386.210928	0.376325252	0.00715083	0.045187
ENSG00000164597	COG5	1342.425828	1657.95606	-0.304834503	0.00716044	0.045231
ENSG00000075826	SEC31B	693.8540642	884.4959909	-0.349351792	0.007167533	0.045248
ENSG00000116521	SCAMP3	1738.720756	1380.817303	0.332330271	0.007168675	0.045248
ENSG00000137100	DCTN3	1002.69188	806.6489908	0.314031727	0.007193446	0.045386
ENSG00000108375	RNF43	2094.440258	2560.331603	-0.290018168	0.007199303	0.045402
ENSG00000021762	OSBPL5	1187.377557	1495.064622	-0.332392992	0.00720293	0.045402
ENSG00000183340	JRKL	759.5926505	951.5134378	-0.32561285	0.007205933	0.045402
ENSG00000198839	ZNF277	762.5041089	1061.337597	-0.477567307	0.007207011	0.045402
ENSG00000148843	PDCD11	4583.347921	3621.98872	0.339704662	0.007214511	0.045432
ENSG00000198840	MT-ND3	22033.04001	18426.17976	0.257897924	0.007221934	0.045461
ENSG00000179981	TSHZ1	853.3930933	672.7427169	0.342801385	0.007241394	0.045566
ENSG00000119471	HSDL2	1594.884525	1978.130449	-0.310961303	0.007256355	0.045642
ENSG00000259494	MRPL46	324.9938848	227.2459421	0.51885956	0.00726029	0.04565
ENSG00000204616	TRIM31	118.2654238	178.3700219	-0.59204891	0.007286377	0.045796
ENSG00000141179	PCTP	374.0387293	493.3672715	-0.400403082	0.007291844	0.045812
ENSG00000130023	ERMARD	634.740676	858.9891725	-0.436497933	0.007294513	0.045812
ENSG00000183077	AFMID	514.4657922	651.0384248	-0.340346725	0.007309785	0.04589
ENSG00000213551	DNAJC9	1481.121673	1119.773228	0.403442365	0.007333198	0.04602

ENSG00000102547	CAB39L	479.0469509	329.3356823	0.538049926	0.007359425	0.046144
ENSG00000164535	DAGLB	817.5066883	649.4022553	0.332446781	0.007361853	0.046144
ENSG00000141664	ZCCHC2	1320.87729	1051.773302	0.328579656	0.007363809	0.046144
ENSG00000154065	ANKRD29	669.9707739	872.744184	-0.381910864	0.007364339	0.046144
ENSG00000259291	ZNF710-AS1	85.71372705	148.5646528	-0.793741783	0.007375993	0.0462
ENSG00000106366	SERPINE1	335.224407	954.2101071	-1.509169388	0.007391058	0.046276
ENSG00000105379	ETFB	887.8933942	701.4966662	0.340294505	0.007400632	0.046313
ENSG00000113456	RAD1	1168.333348	899.5826763	0.377445726	0.007402569	0.046313
ENSG00000103351	CLUAP1	305.7932029	404.3985078	-0.403965277	0.00740697	0.046323
ENSG00000111196	MAGOHB	757.4300467	584.7800769	0.374117988	0.00741326	0.046335
ENSG00000213071	LPAL2	55.6407245	93.87886906	-0.754201226	0.00741454	0.046335
ENSG00000048052	HDAC9	57.38217951	29.25034254	0.9640884	0.007449943	0.046538
ENSG00000165186	PTCHD1	20.62274669	44.99146119	-1.123615266	0.007499258	0.046828
ENSG00000123159	GIPC1	5237.242643	4407.821757	0.248740439	0.007512818	0.046895
ENSG00000005243	COPZ2	16.74913765	39.70639858	-1.248283705	0.007524608	0.046951
ENSG00000106211	HSPB1	15381.98121	12217.07534	0.332383599	0.007533141	0.046986
ENSG00000139354	GAS2L3	1529.884399	1190.324174	0.361915796	0.007548923	0.047052
ENSG00000067064	IDI1	3902.899278	3187.630173	0.292173025	0.007549573	0.047052
ENSG00000172818	OVOL1	340.6880279	463.8435348	-0.445606115	0.007553674	0.04706
ENSG00000147164	SNX12	1084.482683	877.7080801	0.304969584	0.007569085	0.047138
ENSG00000278730	AC005332.6	475.8304326	344.6097313	0.464011166	0.007586668	0.04723
ENSG00000126903	SLC10A3	643.397053	492.898165	0.384782216	0.007600927	0.047283
ENSG00000132361	CLUH	7415.587221	5839.925674	0.344723355	0.007600941	0.047283
ENSG00000110628	SLC22A18	2036.08567	4078.601684	-1.002248653	0.007632136	0.047449
ENSG00000026103	FAS	362.3138285	249.661896	0.537136564	0.007633557	0.047449
ENSG00000156508	EEF1A1	230067.4948	279578.3712	-0.281199219	0.007651652	0.047544
ENSG00000197555	SIPA1L1	3253.450869	4103.954009	-0.335119394	0.007666526	0.047618
ENSG00000070731	ST6GALNAC2	24.95515974	52.82009211	-1.085027646	0.007675207	0.047654
ENSG00000130312	MRPL34	972.7177433	751.5206163	0.373272731	0.007683788	0.047689
ENSG00000073150	PANX2	255.8233524	177.7346181	0.52460968	0.007691056	0.047716
ENSG00000164938	TP53INP1	18.31566899	43.90921435	-1.265150487	0.007706285	0.047792
ENSG00000076641	PAG1	841.7485268	665.197205	0.339854135	0.007719431	0.047839
ENSG00000079308	TNS1	47.08230994	83.39714241	-0.826222919	0.007719697	0.047839
ENSG00000060656	PTPRU	2197.364517	2727.536848	-0.311884867	0.007734962	0.047903
ENSG00000153904	DDAH1	5247.918829	4305.471391	0.285535368	0.00773576	0.047903
ENSG00000171793	CTPS1	3299.648564	2514.337376	0.392408866	0.007743247	0.047927
ENSG00000116212	LRRC42	4091.038931	3370.892761	0.279334402	0.007745606	0.047927
ENSG00000134864	GGACT	41.36802502	75.64262621	-0.876374448	0.007748767	0.047929
ENSG00000123473	STIL	1882.390038	1430.003049	0.396645754	0.007757308	0.047963
ENSG00000128342	LIF	1839.362387	2331.221631	-0.342243277	0.007760507	0.047965
ENSG00000105402	NAPA	1316.362607	1061.72637	0.310440117	0.007767988	0.047993
ENSG00000170037	CNTROB	1661.936901	1344.764252	0.305623118	0.007777515	0.048034
ENSG00000196230	TUBB	28527.83314	20716.04839	0.461631558	0.007785061	0.048062

ENSG00000117450	PRDX1	12928.48661	10154.14638	0.348493081	0.007809921	0.048198
ENSG00000100784	RPS6KA5	285.6196726	204.5202106	0.48236932	0.007831481	0.048312
ENSG00000179058	C9orf50	24.70831341	54.49091476	-1.136775783	0.007838679	0.048339
ENSG00000279407	AC007191.1	202.0801273	277.5722634	-0.456083757	0.007841682	0.048339
ENSG00000142949	PTPRF	14992.2097	18986.79057	-0.340803417	0.007854002	0.048397
ENSG00000186522	10-Sep	4469.360517	5600.377836	-0.325591285	0.007866012	0.048452
ENSG00000188786	MTF1	1130.945602	877.1229114	0.366887843	0.007884082	0.048545
ENSG00000166295	ANAPC16	1615.090814	2049.253143	-0.343625499	0.007889578	0.048561
ENSG00000173890	GPR160	596.6056	787.4159341	-0.401207739	0.007911674	0.048679
ENSG00000129195	PIMREG	1286.430084	988.305247	0.380161276	0.007915574	0.048684
ENSG00000131480	AOC2	166.44338	103.4683677	0.688852748	0.007930923	0.048752
ENSG00000171848	RRM2	6228.830608	3476.152029	0.841504902	0.007932563	0.048752
ENSG00000204366	ZBTB12	154.3793511	221.9600496	-0.525172435	0.007950605	0.048845
ENSG00000083520	DIS3	3018.35151	2447.79959	0.301961536	0.007962785	0.048901
ENSG00000133678	TMEM254	97.12148633	146.4947174	-0.59608108	0.00800411	0.049132
ENSG00000266074	BAHCC1	2191.664125	2680.082622	-0.290328146	0.008006437	0.049132
ENSG00000100316	RPL3	38883.44041	47381.94691	-0.285201333	0.008013608	0.049158
ENSG00000138303	ASCC1	673.423589	843.5198622	-0.325520748	0.008025171	0.049201
ENSG00000142197	DOP1B	1409.392883	1750.30961	-0.312655753	0.008026743	0.049201
ENSG00000271009	AC116667.1	23.36613135	47.26463528	-1.014962113	0.008039384	0.04926
ENSG00000105656	ELL	834.2044623	659.7970967	0.338969131	0.008060151	0.049369
ENSG00000165338	HECTD2	131.6997393	86.80806943	0.598762609	0.008079496	0.049469
ENSG00000132600	PRMT7	1701.222752	1341.270666	0.343024366	0.008086258	0.049492
ENSG00000107816	LZTS2	1678.921847	2085.1932	-0.312665078	0.008094848	0.049526
ENSG00000238290	AL034417.2	11.2970571	29.49061003	-1.382949481	0.008134868	0.049752
ENSG00000280924	LINC00628	23.32802469	8.386251981	1.479875763	0.008143951	0.049773
ENSG00000141552	ANAPC11	1361.548735	1092.338467	0.318180422	0.008146078	0.049773
ENSG00000244486	SCARF2	17.4841208	39.11113767	-1.157321479	0.008147395	0.049773
ENSG00000260032	NORAD	12649.3896	16061.10521	-0.344534755	0.008151222	0.049778
ENSG00000110958	PTGES3	11311.20387	8646.26432	0.387616272	0.008155044	0.049783
ENSG00000149100	EIF3M	5566.311465	6755.530443	-0.279459742	0.008163711	0.049801
ENSG00000185803	SLC52A2	1711.750403	1304.485524	0.392421527	0.008164187	0.049801
ENSG00000145494	NDUFS6	1537.009874	1181.982996	0.379411937	0.008169318	0.049814
ENSG00000130309	COLGALT1	5024.594721	3917.066035	0.359285365	0.008172552	0.049815
ENSG00000111361	EIF2B1	1748.087304	1333.49144	0.39058978	0.008193349	0.049923

Table S4: DEGs of RNA Sequencing data (HCT15).

	gene_name	OXAR_mean	NTC_mean	log2FoldChange	pvalue	padj
ENSG00000213949	ITGA1	4622.361104	88.66139145	5.711601228	0	0
ENSG00000071575	TRIB2	2454.45744	147.1610971	4.057538871	8.83E-232	1.08E-227
ENSG00000131711	MAP1B	1811.2038	28.61290819	5.982942158	1.07E-226	8.80E-223
ENSG00000101210	EEF1A2	62.0078061	2637.855576	-5.409358646	1.85E-188	1.14E-184
ENSG00000127831	VIL1	95.58856588	3677.049215	-5.26378577	8.86E-175	4.35E-171

ENSG00000170961	HAS2	1391.452504	10.99567448	6.965034086	3.38E-150	1.38E-146
ENSG00000125384	PTGER2	1399.990762	18.35661804	6.216654749	4.24E-147	1.49E-143
ENSG00000085563	ABCB1	385.1247317	8825.980297	-4.51865197	1.65E-141	5.06E-138
ENSG00000165802	NSMF	33912.09078	3002.307059	3.497514192	1.46E-140	3.97E-137
ENSG00000096696	DSP	2740.893747	13256.8951	-2.274012846	2.75E-140	6.76E-137
ENSG00000127124	HIVEP3	1057.615922	44.5555104	4.556122915	1.99E-138	4.44E-135
ENSG00000128512	DOCK4	1311.381671	114.9762612	3.510409288	2.61E-134	5.34E-131
ENSG00000168528	SERINC2	11910.53539	1882.287762	2.661213904	2.06E-124	3.89E-121
ENSG00000113448	PDE4D	1689.636304	239.9018714	2.814355303	1.30E-120	2.29E-117
ENSG00000104059	FAM189A1	1762.9211	191.3214679	3.202781265	1.33E-117	2.17E-114
ENSG00000142089	IFITM3	2812.842733	13117.76822	-2.221356643	4.29E-117	6.58E-114
ENSG00000204103	MAFB	784.3463367	50.19500674	3.968186087	4.97E-116	7.17E-113
ENSG00000077984	CST7	836.8152598	37.78456542	4.457121515	1.16E-115	1.59E-112
ENSG00000172927	MYEOV	2035.494826	172.9715215	3.552798701	3.74E-112	4.83E-109
ENSG00000134775	FHOD3	2073.332304	365.2247968	2.504640374	2.65E-109	3.26E-106
ENSG00000143320	CRABP2	13599.61288	1980.4271	2.77916154	2.06E-107	2.41E-104
ENSG00000090238	YPEL3	15.18476027	1483.757392	-6.605418275	3.94E-107	4.40E-104
ENSG00000268621	IGFL2-AS1	710.2578087	27.44614904	4.704673504	1.02E-106	1.08E-103
ENSG00000182111	ZNF716	760.6775614	59.6682759	3.671735657	5.39E-106	5.51E-103
ENSG00000092969	TGFB2	2200.890626	108.9031098	4.329038732	8.98E-105	8.64E-102
ENSG00000149212	SESN3	1258.165617	140.6656525	3.163945857	9.15E-105	8.64E-102
ENSG00000104783	KCNN4	845.8927999	25.29250617	5.084521309	6.76E-102	6.15E-99
ENSG00000175471	MCTP1	951.4784546	124.1297813	2.936345767	1.60E-99	1.38E-96
ENSG00000121753	ADGRB2	1380.894107	181.0652306	2.933051041	1.63E-99	1.38E-96
ENSG00000147027	TMEM47	640.8795542	28.37073793	4.498700405	1.05E-98	8.60E-96
ENSG00000026508	CD44	1632.146203	207.623065	2.97616975	4.54E-97	3.59E-94
ENSG00000078018	MAP2	607.8164752	35.649568	4.085529421	9.87E-95	7.58E-92
ENSG00000142910	TINAGL1	6668.153443	1493.009039	2.159186244	4.52E-93	3.36E-90
ENSG00000021826	CPS1	1213.520837	164.9407853	2.882736594	2.11E-90	1.52E-87
ENSG00000124882	EREG	718.0534217	6779.921873	-3.2393035	5.47E-89	3.84E-86
ENSG00000179163	FUCA1	4741.17991	1145.922429	2.048285565	1.36E-87	9.29E-85
ENSG00000110090	CPT1A	341.6952581	1797.555704	-2.394314001	5.93E-87	3.93E-84
ENSG00000179403	VWA1	154.8281439	1202.979524	-2.955885933	6.63E-85	4.28E-82
ENSG00000109452	INPP4B	886.7376063	81.77910755	3.43060951	1.38E-84	8.67E-82
ENSG00000142623	PADI1	654.7045524	22.05810017	4.906098858	1.71E-84	1.05E-81
ENSG00000261150	EPPK1	577.8908994	2602.373792	-2.171471175	2.28E-84	1.36E-81
ENSG00000167755	KLK6	4733.832899	1088.389892	2.120257075	2.94E-83	1.72E-80
ENSG00000141526	SLC16A3	4631.514723	937.3425662	2.305839287	4.03E-83	2.30E-80
ENSG00000116132	PRRX1	771.1512747	46.43692336	4.053201309	3.35E-82	1.87E-79
ENSG00000138821	SLC39A8	1377.373491	250.3474052	2.457217461	3.01E-81	1.64E-78
ENSG00000057704	TMCC3	35.90217809	572.433479	-3.993209488	1.21E-80	6.46E-78
ENSG00000105514	RAB3D	47.46916636	858.0855012	-4.17406909	2.28E-80	1.19E-77
ENSG00000163171	CDC42EP3	2536.654036	644.2277048	1.977663419	2.40E-80	1.23E-77

ENSG00000129521	EGLN3	1356.639771	148.3318827	3.189461159	1.37E-79	6.88E-77
ENSG00000067798	NAV3	535.4833338	8.009354469	6.090275653	3.14E-79	1.51E-76
ENSG00000159733	ZFYVE28	1037.084636	153.7958944	2.751865899	3.14E-79	1.51E-76
ENSG00000128683	GAD1	2991.469505	608.3862348	2.29698969	3.37E-79	1.59E-76
ENSG00000050555	LAMC3	450.2278602	17.66242075	4.682987017	5.27E-79	2.44E-76
ENSG00000161405	IKZF3	483.3424834	19.80721642	4.603960165	7.31E-79	3.33E-76
ENSG00000119655	NPC2	9544.372013	2363.898286	2.013634598	6.35E-77	2.83E-74
ENSG00000188158	NHS	988.6622375	116.474457	3.088106038	8.95E-77	3.92E-74
ENSG00000160183	TMPRSS3	25.80595687	554.5374745	-4.426627089	5.55E-76	2.39E-73
ENSG00000124225	PMEPA1	897.2307249	3.946219393	7.838124707	6.95E-76	2.94E-73
ENSG00000102886	GDPD3	242.9604536	1214.446717	-2.321285036	1.93E-75	8.04E-73
ENSG00000169174	PCSK9	16.28561312	778.3456777	-5.575972883	4.74E-74	1.94E-71
ENSG00000092068	SLC7A8	9192.959101	2011.850622	2.192357093	4.96E-74	2.00E-71
ENSG00000198203	SULT1C2	101.0387714	1635.124624	-4.017636443	8.16E-72	3.21E-69
ENSG00000135925	WNT10A	1383.402448	262.0085729	2.402773169	8.23E-72	3.21E-69
ENSG00000171658	NMRAL2P	540.1417097	16.32333062	5.01650203	8.77E-72	3.36E-69
ENSG00000165215	CLDN3	707.0597427	3795.664102	-2.42391333	2.18E-71	8.23E-69
ENSG00000081803	CADPS2	51.53501812	537.6266197	-3.382184434	3.31E-71	1.23E-68
ENSG00000117394	SLC2A1	23798.92779	6585.193491	1.853450216	3.58E-70	1.31E-67
ENSG00000123095	BHLHE41	473.6860689	45.92604125	3.368358426	5.35E-70	1.93E-67
ENSG00000203727	SAMD5	103.1061895	868.6693599	-3.075864382	1.02E-68	3.64E-66
ENSG00000110492	MDK	48.41383944	593.1605402	-3.610735757	2.53E-68	8.88E-66
ENSG00000159871	LYPD5	1449.849737	256.0566498	2.501230251	7.78E-68	2.69E-65
ENSG00000049618	ARID1B	970.1856697	3540.352271	-1.867420369	2.26E-67	7.70E-65
ENSG00000117984	CTSD	742.3497192	3239.469027	-2.125508509	9.16E-67	3.08E-64
ENSG00000139318	DUSP6	2420.331876	380.2954761	2.668658642	3.76E-66	1.25E-63
ENSG00000132718	SYT11	350.9612764	12.15255707	4.860936151	6.84E-66	2.24E-63
ENSG00000196660	SLC30A10	539.352112	28.89485805	4.212501153	1.10E-64	3.54E-62
ENSG00000235437	LINC01278	5.483188014	641.8951121	-6.879141349	1.11E-64	3.55E-62
ENSG00000224078	SNHG14	408.8638985	20.86092966	4.294798941	1.55E-64	4.86E-62
ENSG00000145287	PLAC8	133.8277046	899.4073889	-2.747220713	3.46E-64	1.08E-61
ENSG00000124813	RUNX2	3835.377644	754.770121	2.344182714	6.58E-64	2.02E-61
ENSG00000138795	LEF1	6137.086318	1819.415946	1.754237013	9.19E-64	2.78E-61
ENSG00000012124	CD22	464.753335	46.86824624	3.317166476	1.91E-63	5.71E-61
ENSG00000162849	KIF26B	878.9777037	126.1968288	2.806183133	5.28E-63	1.55E-60
ENSG00000160712	IL6R	743.7895867	132.4588848	2.48870942	5.31E-63	1.55E-60
ENSG00000279806	AC018629.1	906.8612832	140.8170186	2.68625834	7.86E-63	2.27E-60
ENSG00000077274	CAPN6	343.3250364	12.44386244	4.793520703	1.36E-62	3.89E-60
ENSG00000148053	NTRK2	1057.34241	255.6110596	2.048717132	3.23E-62	9.12E-60
ENSG00000188959	C9orf152	444.9385891	1915.275058	-2.105795685	3.44E-62	9.60E-60
ENSG00000135373	EHF	364.6467138	2156.399289	-2.563655427	5.44E-62	1.50E-59
ENSG00000143867	OSR1	362.3194363	24.10762262	3.908664308	1.55E-61	4.23E-59
ENSG00000188761	BCL2L15	2031.74306	493.222867	2.041337806	7.95E-61	2.14E-58

ENSG00000115828	QPCT	4923.505622	1547.128464	1.670328103	1.13E-60	3.01E-58
ENSG00000115468	EFHD1	11.58918312	405.3527189	-5.124628398	1.16E-60	3.05E-58
ENSG00000197329	PELI1	831.6783586	2656.039278	-1.67483566	7.62E-60	1.99E-57
ENSG00000168172	HOOK3	1880.708523	6606.575309	-1.812709236	1.54E-59	3.98E-57
ENSG00000143819	EPHX1	11825.27921	2889.636485	2.032564558	1.68E-59	4.29E-57
ENSG00000163618	CADPS	3744.616247	378.8638478	3.305389202	1.86E-59	4.70E-57
ENSG00000144366	GULP1	1251.305538	302.962278	2.044316772	2.37E-59	5.94E-57
ENSG00000221963	APOL6	172.4503765	1316.000206	-2.930726469	2.41E-59	5.98E-57
ENSG00000123843	C4BPB	241.8075913	1455.663928	-2.589085171	2.96E-59	7.27E-57
ENSG00000119681	LTBP2	409.3990336	39.08387594	3.398760074	5.93E-59	1.44E-56
ENSG00000120708	TGFBI	1022.940956	68.61192647	3.908241043	6.47E-59	1.56E-56
ENSG00000113657	DPYSL3	5447.607275	650.7801419	3.064052161	1.01E-58	2.41E-56
ENSG00000071073	MGAT4A	693.1518354	2302.785807	-1.731848543	1.55E-58	3.67E-56
ENSG00000213722	DDAH2	438.7403187	1747.735692	-1.993119954	1.83E-58	4.28E-56
ENSG00000188833	ENTPD8	2470.993224	197.9255807	3.64584511	2.74E-58	6.34E-56
ENSG00000143631	FLG	316.6416443	12.69736861	4.617188833	9.70E-58	2.22E-55
ENSG00000196440	ARMCX4	6.145002206	473.4039629	-6.262432812	4.61E-57	1.05E-54
ENSG00000197915	HRNR	300.93079	17.99401404	4.075554802	9.36E-57	2.11E-54
ENSG00000127022	CANX	19863.49565	57178.89433	-1.525356715	7.10E-56	1.58E-53
ENSG00000183873	SCN5A	390.7147672	42.89943332	3.194403478	3.24E-55	7.16E-53
ENSG00000196542	SPTSSB	344.4687175	27.18387398	3.665693398	3.28E-55	7.19E-53
ENSG00000128340	RAC2	13.53799593	355.6943271	-4.723089254	3.33E-55	7.24E-53
ENSG00000127084	FGD3	388.8944054	1417.31847	-1.865280564	4.81E-55	1.04E-52
ENSG00000170190	SLC16A5	23.42698392	550.4133461	-4.559753951	5.87E-55	1.25E-52
ENSG00000162783	IER5	2809.162652	896.8320006	1.647076615	6.46E-55	1.37E-52
ENSG00000111885	MAN1A1	4533.167809	1459.418906	1.634923257	2.40E-54	5.04E-52
ENSG00000232774	FLJ22447	347.842021	12.5092326	4.785326969	4.20E-54	8.74E-52
ENSG00000157601	MX1	160.5247411	809.5476808	-2.333931325	5.48E-54	1.13E-51
ENSG00000079393	DUSP13	355.3183973	21.02311068	4.063655693	8.40E-54	1.72E-51
ENSG00000164379	FOXQ1	13439.05031	4779.421599	1.491575905	4.53E-53	9.18E-51
ENSG00000136111	TBC1D4	4129.351586	11505.79125	-1.478274949	6.59E-53	1.33E-50
ENSG00000145920	CPLX2	527.1867121	28.05771179	4.246321349	7.10E-53	1.42E-50
ENSG00000102678	FGF9	485.2571951	48.61813717	3.307938837	8.33E-53	1.65E-50
ENSG00000130707	ASS1	2507.619416	11540.947	-2.202314023	8.40E-53	1.65E-50
ENSG00000057019	DCBLD2	8180.270649	2205.794921	1.890437215	1.62E-52	3.16E-50
ENSG00000165272	AQP3	369.0504616	1456.622184	-1.979611013	2.12E-52	4.10E-50
ENSG00000006468	ETV1	487.6035108	73.07950386	2.733843153	2.94E-52	5.64E-50
ENSG00000124459	ZNF45	1410.157415	212.7079388	2.732631042	3.15E-52	5.99E-50
ENSG00000101460	MAP1LC3A	68.15803952	592.688148	-3.118373479	4.56E-52	8.60E-50
ENSG00000182107	TMEM30B	418.1021738	1423.177361	-1.76684204	1.26E-51	2.37E-49
ENSG00000188372	ZP3	17.40977941	293.9796168	-4.076012464	6.00E-51	1.12E-48
ENSG00000167637	ZNF283	1706.868262	284.3012989	2.588162509	1.48E-50	2.74E-48
ENSG00000129038	LOXL1	637.6285032	121.7289124	2.393661307	1.80E-50	3.30E-48

ENSG00000276644	DACH1	537.7284844	35.80770992	3.927991581	3.34E-50	6.07E-48
ENSG00000166441	RPL27A	11594.77937	32536.19205	-1.488556168	5.61E-50	1.01E-47
ENSG00000164093	PITX2	838.6733841	3946.442179	-2.234131395	5.92E-50	1.06E-47
ENSG00000172830	SSH3	505.3632214	1564.541197	-1.629915222	6.57E-50	1.17E-47
ENSG00000136010	ALDH1L2	35.29760459	496.4092569	-3.814778775	8.32E-50	1.47E-47
ENSG00000044115	CTNNA1	2825.89096	9333.801999	-1.723886786	9.48E-50	1.66E-47
ENSG00000204335	SP5	6044.999187	2040.928195	1.566125687	1.09E-49	1.90E-47
ENSG00000044524	EPHA3	283.5701662	6.795948919	5.411641176	1.51E-49	2.60E-47
ENSG00000012779	ALOX5	299.41371	24.47460471	3.614901516	1.64E-49	2.82E-47
ENSG00000120318	ARAP3	609.7686072	136.9455249	2.153877249	1.75E-49	2.99E-47
ENSG00000232480	TGFB2-AS1	265.7349672	8.09337007	5.030397838	6.52E-49	1.10E-46
ENSG00000128165	ADM2	182.3553833	1963.539981	-3.427374185	1.51E-48	2.55E-46
ENSG00000198074	AKR1B10	305.2828423	6.363674984	5.535910101	1.63E-48	2.72E-46
ENSG00000108771	DHX58	11.10590613	305.9872445	-4.768019343	1.99E-48	3.30E-46
ENSG00000182578	CSF1R	352.7926026	22.61278826	3.941928685	2.66E-48	4.39E-46
ENSG00000196776	CD47	1249.118861	4238.898446	-1.76267391	4.12E-48	6.75E-46
ENSG00000111275	ALDH2	832.5564359	2403.96344	-1.52971467	1.96E-47	3.18E-45
ENSG00000154930	ACSS1	141.8419327	1102.291272	-2.957250715	3.14E-47	5.07E-45
ENSG00000138759	FRAS1	2435.084391	5959.551	-1.291136886	3.94E-47	6.32E-45
ENSG00000120149	MSX2	2750.337731	764.8022233	1.846928336	5.54E-47	8.84E-45
ENSG00000120594	PLXDC2	673.1201732	146.7710005	2.198410877	6.43E-47	1.02E-44
ENSG00000142235	LMTK3	236.6959014	981.1856489	-2.051292505	6.77E-47	1.07E-44
ENSG00000153721	CNKSR3	149.1125113	702.9138923	-2.236926179	1.28E-46	2.00E-44
ENSG00000147883	CDKN2B	1625.664903	189.0331764	3.100064829	2.73E-46	4.24E-44
ENSG00000101298	SNPH	803.5420966	219.0674051	1.873928404	2.79E-46	4.30E-44
ENSG00000130600	H19	6.193201786	457.2502145	-6.208424554	2.86E-46	4.39E-44
ENSG00000171903	CYP4F11	1882.983726	503.4031672	1.903554627	4.28E-46	6.53E-44
ENSG00000164125	FAM198B	287.360605	13.07070918	4.465739963	4.48E-46	6.79E-44
ENSG00000188385	JAKMIP3	353.7938743	39.7623465	3.158872411	1.73E-45	2.60E-43
ENSG00000213445	SIPA1	69.72663828	514.9923071	-2.882375058	2.73E-45	4.08E-43
ENSG00000177595	PIDD1	369.5372345	1215.060026	-1.716417968	3.10E-45	4.61E-43
ENSG00000111145	ELK3	1746.008227	603.8564409	1.531481821	6.58E-45	9.74E-43
ENSG00000196872	KIAA1211L	33.61183315	402.353356	-3.58468925	7.62E-45	1.12E-42
ENSG00000102804	TSC22D1	12296.57677	3523.659388	1.803112686	1.38E-44	2.02E-42
ENSG00000111962	UST	344.5142145	1178.904749	-1.774154195	1.83E-44	2.66E-42
ENSG00000159882	ZNF230	903.5991335	161.3030446	2.490086205	2.23E-44	3.23E-42
ENSG00000166446	CDYL2	736.026062	153.604253	2.258510775	3.51E-44	5.05E-42
ENSG00000115756	HPCAL1	2635.031273	1026.412621	1.360282604	5.60E-44	7.99E-42
ENSG00000139970	RTN1	234.2417681	13.57179303	4.110416629	2.41E-43	3.43E-41
ENSG00000024422	EHD2	391.327437	1871.695045	-2.257680271	2.85E-43	4.02E-41
ENSG00000168743	NPNT	562.4887686	3164.98987	-2.492184578	3.50E-43	4.91E-41
ENSG00000071242	RPS6KA2	370.8426181	1173.126199	-1.66085018	3.64E-43	5.08E-41
ENSG00000111981	ULBP1	147.034	1129.902493	-2.940092601	3.90E-43	5.41E-41

ENSG00000163082	SGPP2	252.0956548	927.3626488	-1.878586128	4.29E-43	5.91E-41
ENSG00000204219	TCEA3	22.25588801	281.6639149	-3.664853585	5.28E-43	7.24E-41
ENSG00000260604	AL590004.3	464.2546895	85.13035094	2.446434935	6.69E-43	9.12E-41
ENSG00000129451	KLK10	3291.276465	1154.430953	1.512036622	1.42E-42	1.92E-40
ENSG00000184160	ADRA2C	4483.956465	1362.765046	1.718054823	2.06E-42	2.77E-40
ENSG00000139211	AMIGO2	214.125442	10.39536934	4.347764211	2.07E-42	2.77E-40
ENSG00000142156	COL6A1	12.60013799	261.6016674	-4.365521148	2.64E-42	3.53E-40
ENSG00000082781	ITGB5	8502.336931	3171.263124	1.422669564	3.72E-42	4.94E-40
ENSG00000115295	CLIP4	502.385537	70.98867687	2.817204743	7.20E-42	9.51E-40
ENSG00000087842	PIR	788.4160066	208.9708072	1.913658267	7.85E-42	1.03E-39
ENSG00000111344	RASAL1	413.6602319	60.90771473	2.761216436	8.82E-42	1.15E-39
ENSG00000143195	ILDR2	1048.727126	318.3163282	1.719666412	1.09E-41	1.42E-39
ENSG00000168237	GLYCTK	335.6921634	1940.3996	-2.530404058	1.24E-41	1.60E-39
ENSG00000229512	AC068580.1	324.1836745	32.88574344	3.314996456	1.58E-41	2.03E-39
ENSG00000104870	FCGRT	9.370665155	315.1035905	-5.062202775	2.86E-41	3.66E-39
ENSG00000115825	PRKD3	4429.704343	1700.545235	1.380850382	4.27E-41	5.43E-39
ENSG00000134107	BHLHE40	1234.310819	369.1250347	1.740183446	4.82E-41	6.10E-39
ENSG00000186205	1-Mar	424.9119984	1552.381006	-1.870124689	1.36E-40	1.72E-38
ENSG00000145349	CAMK2D	6232.448276	2632.345531	1.243363155	1.37E-40	1.72E-38
ENSG00000275342	PRAG1	12417.84807	3614.842506	1.780137637	1.56E-40	1.95E-38
ENSG00000070081	NUCB2	689.5753077	2343.632718	-1.764760025	1.71E-40	2.12E-38
ENSG00000171124	FUT3	32.32752582	440.0781137	-3.766325151	2.00E-40	2.47E-38
ENSG00000168453	HR	125.0668441	586.6818917	-2.227978463	2.63E-40	3.23E-38
ENSG00000187134	AKR1C1	211.5127404	10.67438775	4.321075415	3.23E-40	3.94E-38
ENSG00000110700	RPS13	5355.087881	13191.26029	-1.300569544	3.27E-40	3.97E-38
ENSG00000186481	ANKRD20A5P	401.8622345	63.75546386	2.659721202	3.97E-40	4.80E-38
ENSG00000137501	SYTL2	1320.955561	463.3457111	1.511426562	5.27E-40	6.35E-38
ENSG00000053747	LAMA3	3291.38082	1373.263313	1.261300566	1.02E-39	1.23E-37
ENSG00000152127	MGAT5	11363.38463	4339.031948	1.388861619	2.17E-39	2.59E-37
ENSG00000082438	COBLL1	534.3388929	1799.190127	-1.750943152	3.64E-39	4.31E-37
ENSG00000212901	KRTAP3-1	284.4097017	24.04670885	3.581293562	4.11E-39	4.85E-37
ENSG00000166924	NYAP1	26.35059838	275.3648411	-3.380914839	4.63E-39	5.44E-37
ENSG00000114541	FRMD4B	375.9506607	1433.855477	-1.93053043	4.95E-39	5.79E-37
ENSG00000175600	SUGCT	370.8097982	46.04141927	3.016206508	7.21E-39	8.39E-37
ENSG00000283167	AC140479.7	5.015604692	259.2773095	-5.674517139	7.58E-39	8.78E-37
ENSG00000073150	PANX2	1394.585869	330.9893833	2.072372035	1.26E-38	1.46E-36
ENSG00000118523	CTGF	1803.461595	347.2960234	2.375420328	1.53E-38	1.75E-36
ENSG00000197635	DPP4	8.043075514	368.0072289	-5.520926527	1.62E-38	1.85E-36
ENSG00000141448	GATA6	635.5013382	2009.543775	-1.660468476	3.52E-38	4.00E-36
ENSG00000138316	ADAMTS14	752.2089374	100.3077049	2.899787511	4.10E-38	4.64E-36
ENSG00000196154	S100A4	516.4852977	111.96538	2.203878196	1.07E-37	1.21E-35
ENSG00000118513	MYB	75.50791352	534.1847814	-2.823705317	2.70E-37	3.03E-35
ENSG00000182704	TSKU	3880.95052	1027.409563	1.91659002	3.13E-37	3.49E-35

ENSG00000104723	TUSC3	272.5603423	2.606099912	6.655149578	3.31E-37	3.68E-35
ENSG00000169247	SH3TC2	539.9486087	97.70676475	2.472179012	4.35E-37	4.81E-35
ENSG00000137502	RAB30	640.6540762	166.5576821	1.940539189	6.13E-37	6.75E-35
ENSG00000148346	LCN2	48.75885628	461.4681027	-3.242396222	7.95E-37	8.72E-35
ENSG00000174607	UGT8	4937.219753	2041.115499	1.274319453	2.05E-36	2.24E-34
ENSG00000186377	CYP4X1	223.5065696	18.25533805	3.636471199	2.19E-36	2.38E-34
ENSG00000141293	SKAP1	205.5159954	19.82637018	3.384379196	2.26E-36	2.44E-34
ENSG00000164690	SHH	527.9091115	131.5239766	2.008448758	2.37E-36	2.56E-34
ENSG00000053918	KCNQ1	324.5778328	1270.822632	-1.968157579	4.50E-36	4.83E-34
ENSG00000163527	STT3B	25870.45174	11626.33527	1.153954244	4.61E-36	4.92E-34
ENSG00000131746	TNS4	10547.40881	1743.167552	2.596611911	5.35E-36	5.68E-34
ENSG00000248323	LUCAT1	578.3586677	60.09238028	3.255606803	7.85E-36	8.30E-34
ENSG00000162366	PDZK1IP1	3.650277911	308.4777843	-6.405979498	9.55E-36	1.01E-33
ENSG00000105048	TNNT1	1202.881695	256.1909339	2.234219879	1.20E-35	1.26E-33
ENSG00000196730	DAPK1	189.0036455	898.1897749	-2.248044132	1.44E-35	1.51E-33
ENSG00000123989	CHPF	18758.21261	6279.432965	1.578812148	1.48E-35	1.54E-33
ENSG00000175322	ZNF519	1159.443078	302.8816894	1.936029379	1.70E-35	1.76E-33
ENSG00000205213	LGR4	573.7094559	1669.954442	-1.541088111	1.98E-35	2.04E-33
ENSG00000167680	SEMA6B	4741.983716	1538.674031	1.623634594	2.22E-35	2.28E-33
ENSG00000159450	TCHH	1094.654646	157.0673436	2.804466701	4.07E-35	4.16E-33
ENSG00000124496	TRERF1	370.3095576	1087.021668	-1.552912621	4.20E-35	4.28E-33
ENSG00000198795	ZNF521	193.809791	13.44113102	3.841208416	4.69E-35	4.76E-33
ENSG00000240694	PNMA2	624.49309	2417.612307	-1.953141818	4.98E-35	5.03E-33
ENSG00000133083	DCLK1	195.0474939	4.398168129	5.437043191	6.72E-35	6.74E-33
ENSG00000235027	AC068580.3	357.100069	44.52004331	3.002057102	6.72E-35	6.74E-33
ENSG00000112419	PHACTR2	711.060936	2084.515289	-1.551901083	7.09E-35	7.08E-33
ENSG00000013275	PSMC4	6365.988275	2237.144017	1.508262664	8.20E-35	8.15E-33
ENSG00000005001	PRSS22	3428.685687	1341.103742	1.354185263	1.25E-34	1.24E-32
ENSG00000175274	TP53I11	301.7118261	1003.575514	-1.733446841	1.36E-34	1.35E-32
ENSG00000109321	AREG	238.2419801	920.1918198	-1.950150323	1.51E-34	1.48E-32
ENSG00000187800	PEAR1	8.263311119	232.9724458	-4.804400615	1.84E-34	1.80E-32
ENSG00000102996	MMP15	2145.834762	5298.295357	-1.303732826	1.86E-34	1.81E-32
ENSG00000110514	MADD	5831.763446	2749.06496	1.085061689	1.95E-34	1.89E-32
ENSG00000237975	FLG-AS1	171.7278511	9.160829629	4.241611773	2.31E-34	2.24E-32
ENSG00000121316	PLBD1	813.4152366	1955.222272	-1.264909925	2.72E-34	2.62E-32
ENSG00000075426	FOSL2	5907.168543	2488.929513	1.247252242	2.97E-34	2.84E-32
ENSG00000283283	AC013268.4	5.470185647	217.9938477	-5.319952161	3.46E-34	3.31E-32
ENSG00000186625	KATNA1	68.6451264	377.4645887	-2.459627667	3.72E-34	3.54E-32
ENSG00000100473	COCH	2.866059824	284.2455319	-6.612958741	4.68E-34	4.43E-32
ENSG00000173559	NABP1	2257.575419	880.1809849	1.359541489	5.23E-34	4.94E-32
ENSG00000110696	C11orf58	2948.876601	6095.055983	-1.047510464	5.89E-34	5.54E-32
ENSG00000213981	AC007277.1	358.9959304	62.90414126	2.516633757	6.34E-34	5.94E-32
ENSG00000179941	BBS10	27.76762068	321.9471894	-3.539878301	6.79E-34	6.34E-32

ENSG00000088726	TMEM40	172.383792	9.415795155	4.202727282	7.74E-34	7.19E-32
ENSG00000135477	KRT87P	16.13038935	228.748303	-3.833292496	8.11E-34	7.51E-32
ENSG00000130396	AFDN	3568.274401	8612.892147	-1.271315826	1.40E-33	1.29E-31
ENSG00000166173	LARP6	10.12491725	188.0528463	-4.211692985	2.03E-33	1.87E-31
ENSG00000127955	GNAI1	506.1136524	114.4988785	2.139253142	2.05E-33	1.88E-31
ENSG00000196139	AKR1C3	213.6835353	16.37487615	3.695847301	2.07E-33	1.89E-31
ENSG00000184465	WDR27	592.2323113	1552.07779	-1.389535284	2.17E-33	1.97E-31
ENSG00000104765	BNIP3L	7028.527103	3121.535155	1.171132522	5.57E-33	5.04E-31
ENSG00000134324	LPIN1	5335.052533	2071.954913	1.36409434	5.95E-33	5.37E-31
ENSG00000102755	FLT1	37.44191823	574.956945	-3.940071659	7.40E-33	6.66E-31
ENSG00000145911	N4BP3	52.9575856	387.3493095	-2.868622369	1.04E-32	9.35E-31
ENSG00000196420	S100A5	191.8489431	13.58897912	3.800485549	1.16E-32	1.04E-30
ENSG00000122035	RASL11A	44.09255486	366.3430861	-3.050105732	1.19E-32	1.06E-30
ENSG00000276170	AC244153.1	28.92871947	313.4269786	-3.433181554	1.29E-32	1.14E-30
ENSG00000204991	SPIRE2	452.7895084	1489.176633	-1.717057332	1.38E-32	1.22E-30
ENSG00000267041	ZNF850	712.39471	200.9342991	1.828672893	1.60E-32	1.41E-30
ENSG00000148357	HMCN2	5.799822779	293.0516266	-5.655938747	1.62E-32	1.42E-30
ENSG00000146112	PPP1R18	1663.613785	671.5079724	1.308455885	1.75E-32	1.53E-30
ENSG00000167183	PRR15L	604.5482726	3691.799248	-2.610099813	3.20E-32	2.78E-30
ENSG00000107249	GLIS3	238.7033762	38.31365023	2.634105212	3.53E-32	3.06E-30
ENSG00000175294	CATSPER1	321.3228459	66.63379368	2.264199733	4.48E-32	3.87E-30
ENSG00000130775	THEMIS2	10.50529389	188.6059967	-4.165306677	5.36E-32	4.61E-30
ENSG00000166444	ST5	291.4124843	1054.368049	-1.855039548	7.83E-32	6.72E-30
ENSG00000166471	TMEM41B	1934.303269	4298.288445	-1.152070642	9.02E-32	7.72E-30
ENSG00000065361	ERBB3	3316.99884	6977.990188	-1.072996083	1.30E-31	1.11E-29
ENSG00000144730	IL17RD	1869.480981	569.8862466	1.712331733	1.59E-31	1.35E-29
ENSG00000099864	PALM	3.949949052	215.5322344	-5.754097605	1.76E-31	1.49E-29
ENSG00000108602	ALDH3A1	3645.931616	1053.622736	1.790000144	2.44E-31	2.06E-29
ENSG00000003989	SLC7A2	884.3159902	1990.019827	-1.170233676	3.00E-31	2.52E-29
ENSG00000152061	RABGAP1L	243.1608605	762.4606921	-1.649827027	3.03E-31	2.54E-29
ENSG00000166394	CYB5R2	4.342058096	196.3146649	-5.493636497	3.22E-31	2.69E-29
ENSG00000100889	PCK2	4702.262184	11028.69648	-1.229846246	4.42E-31	3.68E-29
ENSG00000197956	S100A6	20787.11812	6057.194269	1.779005147	5.12E-31	4.25E-29
ENSG00000196352	CD55	952.6969313	2526.760164	-1.406826419	6.46E-31	5.34E-29
ENSG00000223749	MIR503HG	203.3220721	21.13263853	3.267883192	7.87E-31	6.48E-29
ENSG00000111678	C12orf57	244.7689429	714.5093891	-1.544628279	9.44E-31	7.75E-29
ENSG00000107897	ACBD5	1754.923193	3662.514711	-1.061306289	1.18E-30	9.69E-29
ENSG00000182795	C1orf116	1109.056741	2764.051861	-1.317130833	1.55E-30	1.26E-28
ENSG00000064692	SNCAIP	11.57094953	217.3481989	-4.225461306	1.70E-30	1.38E-28
ENSG00000188706	ZDHHC9	4182.300823	2040.51178	1.035252658	2.27E-30	1.84E-28
ENSG00000157064	NMNAT2	9.05403039	197.0940112	-4.439180509	2.71E-30	2.19E-28
ENSG00000135046	ANXA1	11924.34353	2089.04861	2.512573243	2.94E-30	2.37E-28
ENSG00000167741	GGT6	993.7087388	2683.691637	-1.433010044	3.20E-30	2.57E-28

ENSG00000128564	VGF	971.6395292	255.304574	1.926032865	3.29E-30	2.63E-28
ENSG00000250328	MGC32805	82.51688331	516.1261922	-2.642238473	4.12E-30	3.28E-28
ENSG00000130024	PHF10	1495.384952	3646.185962	-1.285986898	4.70E-30	3.73E-28
ENSG00000124406	ATP8A1	364.1204684	1626.633328	-2.158793228	5.15E-30	4.08E-28
ENSG00000150051	MKX	197.7372313	684.3065029	-1.790981852	5.17E-30	4.08E-28
ENSG00000168350	DEGS2	259.9546592	992.5158233	-1.931949632	6.00E-30	4.72E-28
ENSG00000136999	NOV	225.8371804	38.0763791	2.563855083	7.25E-30	5.69E-28
ENSG00000239467	AC007405.3	1603.45627	668.5098992	1.261331993	8.56E-30	6.69E-28
ENSG00000160191	PDE9A	718.0665837	195.2126765	1.882511731	9.17E-30	7.15E-28
ENSG00000110321	EIF4G2	19826.56476	38256.2578	-0.948269724	1.09E-29	8.47E-28
ENSG00000169241	SLC50A1	1249.530508	2917.295225	-1.223197533	1.26E-29	9.77E-28
ENSG00000197601	FAR1	2109.518134	5061.767038	-1.262796075	1.57E-29	1.22E-27
ENSG00000204869	IGFL4	1881.706265	606.3237735	1.635187901	1.62E-29	1.24E-27
ENSG00000107242	PIP5K1B	130.8926716	575.7813211	-2.135274064	1.63E-29	1.25E-27
ENSG00000130066	SAT1	15819.3577	5838.156349	1.438225416	1.76E-29	1.35E-27
ENSG00000129353	SLC44A2	2790.404209	6154.787458	-1.141246746	2.86E-29	2.18E-27
ENSG00000204334	ERICH2	1038.498014	419.3920562	1.307733838	3.03E-29	2.31E-27
ENSG00000137331	IER3	3994.876847	1015.260537	1.975591958	3.39E-29	2.57E-27
ENSG00000253276	CCDC71L	761.609092	276.4812664	1.463605816	3.99E-29	3.01E-27
ENSG00000175928	LRRN1	746.8237017	240.8313467	1.632471426	4.02E-29	3.03E-27
ENSG00000185339	TCN2	1037.588678	314.8575632	1.722471135	5.65E-29	4.24E-27
ENSG00000154040	CABYR	384.3373429	100.4715404	1.937603446	6.35E-29	4.76E-27
ENSG00000151224	MAT1A	191.759174	17.00808137	3.500741626	6.67E-29	4.98E-27
ENSG00000268879	IGFL1P1	192.8582916	15.45079557	3.661353513	7.18E-29	5.34E-27
ENSG00000138696	BMPR1B	139.3419772	5.733388486	4.604286596	8.04E-29	5.96E-27
ENSG00000154856	APCDD1	7882.155838	2520.554784	1.645072531	8.08E-29	5.98E-27
ENSG00000151468	CCDC3	2031.420964	377.8693697	2.42436523	9.27E-29	6.84E-27
ENSG00000173530	TNFRSF10D	3190.337771	1015.21002	1.65256405	1.06E-28	7.79E-27
ENSG00000078579	FGF20	212.2455911	33.41190953	2.672188283	1.40E-28	1.03E-26
ENSG00000198743	SLC5A3	3276.010052	1489.624048	1.137241929	1.74E-28	1.27E-26
ENSG00000092621	PHGDH	6151.652762	15302.7693	-1.314696556	3.52E-28	2.57E-26
ENSG00000166598	HSP90B1	26902.58265	58714.57657	-1.125974421	4.91E-28	3.57E-26
ENSG00000174197	MGA	1020.500473	2184.901092	-1.098054381	4.97E-28	3.60E-26
ENSG00000186806	VSIG10L	730.5787104	209.9116312	1.797123552	5.32E-28	3.84E-26
ENSG00000132359	RAP1GAP2	1432.486783	3203.511698	-1.160958443	5.92E-28	4.26E-26
ENSG00000159784	FAM131B	114.1548549	590.7743974	-2.37216372	6.02E-28	4.32E-26
ENSG00000130338	TULP4	1073.700954	2339.678084	-1.123400861	6.44E-28	4.61E-26
ENSG00000184014	DENND5A	1654.197084	4226.191386	-1.353382573	6.81E-28	4.86E-26
ENSG00000163545	NUAK2	603.3986372	1658.775307	-1.45820009	7.29E-28	5.19E-26
ENSG00000011405	PIK3C2A	2716.120803	5588.482298	-1.040942576	8.13E-28	5.77E-26
ENSG00000140511	HAPLN3	491.1432657	138.2192449	1.831896675	8.26E-28	5.84E-26
ENSG00000149948	HMGA2	1574.300225	618.4671059	1.3475014	1.15E-27	8.08E-26
ENSG00000079257	LXN	2.542923875	215.9795317	-6.409516722	1.19E-27	8.35E-26

ENSG00000196754	S100A2	256.6551059	49.1698155	2.376829078	1.33E-27	9.36E-26
ENSG00000228536	AL513283.1	133.6804033	4.152049802	4.988418009	1.56E-27	1.09E-25
ENSG00000146433	TMEM181	1332.332347	3395.106007	-1.349322065	1.71E-27	1.20E-25
ENSG00000181885	CLDN7	4761.830668	12513.77065	-1.393906553	1.79E-27	1.25E-25
ENSG00000145431	PDGFC	383.6729888	92.9732246	2.039378573	1.91E-27	1.33E-25
ENSG00000196189	SEMA4A	17.71072052	210.9074662	-3.568339597	2.04E-27	1.41E-25
ENSG00000105357	MYH14	3402.922516	8634.234918	-1.343127983	2.09E-27	1.44E-25
ENSG00000100968	NFATC4	271.5213081	1545.673438	-2.508210985	2.12E-27	1.46E-25
ENSG00000197016	ZNF470	432.3454318	117.0477778	1.888602258	2.28E-27	1.56E-25
ENSG00000164647	STEAP1	428.6382271	68.02210996	2.646124223	2.40E-27	1.64E-25
ENSG00000147044	CASK	1155.304389	2527.710538	-1.129587406	2.82E-27	1.92E-25
ENSG00000149564	ESAM	97.6997457	418.3267993	-2.100771006	2.85E-27	1.94E-25
ENSG00000091136	LAMB1	2330.657912	4992.10903	-1.09868647	2.93E-27	1.99E-25
ENSG00000114812	VIPR1	227.1961686	754.5533341	-1.730485727	3.23E-27	2.18E-25
ENSG00000101236	RNF24	1836.664582	814.5659199	1.173173053	3.47E-27	2.34E-25
ENSG00000163694	RBM47	2504.729087	5749.663879	-1.198892977	3.51E-27	2.36E-25
ENSG00000164120	HPGD	207.2796297	1098.789604	-2.405485664	3.54E-27	2.38E-25
ENSG00000050820	BCAR1	6925.019993	3195.126293	1.115686482	3.97E-27	2.65E-25
ENSG00000105289	TJP3	1765.541224	4627.023104	-1.389805285	4.12E-27	2.75E-25
ENSG00000158258	CLSTN2	140.8793288	3.499169782	5.39833507	4.65E-27	3.10E-25
ENSG00000163659	TIPARP	1351.260266	532.460155	1.342435269	6.79E-27	4.50E-25
ENSG00000090339	ICAM1	1212.715635	3106.315494	-1.357019319	7.69E-27	5.09E-25
ENSG00000177600	RPLP2	7822.626215	19152.90954	-1.291855701	8.04E-27	5.31E-25
ENSG00000117020	AKT3	381.9934046	98.80816648	1.950096587	8.21E-27	5.40E-25
ENSG00000126218	F10	23.96512425	230.7982427	-3.265334811	9.37E-27	6.15E-25
ENSG00000100644	HIF1A	12894.32674	4515.288196	1.513649233	9.46E-27	6.19E-25
ENSG00000260877	AP005233.2	139.8634678	8.65338729	4.005117924	9.95E-27	6.50E-25
ENSG00000163686	ABHD6	1184.634444	386.9550694	1.612226592	1.06E-26	6.91E-25
ENSG00000184828	ZBTB7C	230.0048363	32.88574344	2.818603963	1.31E-26	8.49E-25
ENSG00000133048	CHI3L1	155.922019	2.441508473	6.012401136	1.32E-26	8.57E-25
ENSG00000198758	EPS8L3	1954.253741	759.4474645	1.36371016	1.43E-26	9.25E-25
ENSG00000146416	AIG1	792.6651391	1756.214363	-1.147580851	1.55E-26	1.00E-24
ENSG00000108932	SLC16A6	330.1503021	74.86221657	2.135161227	1.63E-26	1.04E-24
ENSG00000161714	PLCD3	2427.060805	5511.004599	-1.182879658	1.73E-26	1.11E-24
ENSG00000145632	PLK2	1114.924141	279.6287123	1.992865732	1.74E-26	1.12E-24
ENSG00000175745	NR2F1	793.3456887	310.966564	1.352248324	1.75E-26	1.12E-24
ENSG00000183020	AP2A2	1152.411545	2369.708196	-1.040233259	1.84E-26	1.17E-24
ENSG00000138172	CALHM2	106.8168871	490.4982354	-2.196809373	1.85E-26	1.17E-24
ENSG00000182667	NTM	1049.384682	0	12.61924686	2.59E-26	1.64E-24
ENSG00000157399	ARSE	73.01858303	321.0421187	-2.133521041	2.85E-26	1.80E-24
ENSG00000186017	ZNF566	980.0774333	312.807259	1.648073854	2.86E-26	1.80E-24
ENSG00000121310	ECHDC2	13.49502757	179.8697928	-3.738108537	3.80E-26	2.39E-24
ENSG00000128641	MYO1B	4173.820125	10394.23798	-1.316467339	4.59E-26	2.88E-24

ENSG00000131023	LATS1	862.3388597	2031.89743	-1.23670699	5.97E-26	3.73E-24
ENSG00000109819	PPARGC1A	143.4290678	2.813389681	5.718733428	6.72E-26	4.19E-24
ENSG00000085511	MAP3K4	513.2599517	1190.824556	-1.214327166	7.08E-26	4.40E-24
ENSG00000132781	MUTYH	367.6437533	904.2649334	-1.298649147	7.85E-26	4.86E-24
ENSG00000162105	SHANK2	216.5711733	632.9364252	-1.546129312	8.78E-26	5.43E-24
ENSG00000024862	CCDC28A	202.6941739	564.8655445	-1.478179368	9.20E-26	5.68E-24
ENSG00000198108	CHSY3	132.087395	6.135251054	4.395320068	9.55E-26	5.87E-24
ENSG00000163435	ELF3	354.8198837	1639.924679	-2.208226466	1.01E-25	6.20E-24
ENSG00000177666	PNPLA2	1895.343537	4260.117419	-1.168194484	1.05E-25	6.43E-24
ENSG00000058091	CDK14	178.4114589	27.02418166	2.728709014	1.34E-25	8.17E-24
ENSG00000166313	APBB1	43.94921486	248.4304997	-2.498543401	1.55E-25	9.45E-24
ENSG00000180815	MAP3K15	220.7984248	43.34751229	2.342976806	1.59E-25	9.66E-24
ENSG00000253361	AC069120.1	398.6701707	117.3495463	1.764832985	1.81E-25	1.10E-23
ENSG00000135097	MSI1	565.7956136	1352.412925	-1.257717447	2.01E-25	1.22E-23
ENSG00000099139	PCSK5	1121.612546	306.6021302	1.869913791	2.24E-25	1.35E-23
ENSG00000267056	AC005336.1	197.2071873	31.98382637	2.631861886	2.26E-25	1.36E-23
ENSG00000115648	MLPH	1009.55263	375.6681809	1.427626098	2.47E-25	1.48E-23
ENSG00000065923	SLC9A7	501.8006072	1248.355802	-1.315652407	4.32E-25	2.59E-23
ENSG00000165376	CLDN2	1594.961551	4842.569952	-1.601930907	4.38E-25	2.61E-23
ENSG00000158055	GRHL3	196.0181717	710.5677041	-1.857515348	5.68E-25	3.38E-23
ENSG00000250986	LINC02600	748.4295582	157.8720198	2.248349314	5.69E-25	3.38E-23
ENSG00000099822	HCN2	261.3367982	57.65019416	2.184287148	5.97E-25	3.54E-23
ENSG00000165140	FBP1	41.57690568	227.9639009	-2.451194042	6.07E-25	3.59E-23
ENSG00000236287	ZBED5	1076.563835	2358.017607	-1.130869333	6.50E-25	3.83E-23
ENSG00000269069	AC007842.1	297.5132735	55.01411289	2.441430147	7.10E-25	4.18E-23
ENSG00000198223	CSF2RA	19.93192977	171.8364638	-3.104083189	7.62E-25	4.47E-23
ENSG00000187678	SPRY4	2262.138848	727.1348337	1.636200444	7.68E-25	4.50E-23
ENSG00000117226	GBP3	1123.381344	470.869253	1.255439276	8.13E-25	4.75E-23
ENSG00000141480	ARRB2	493.1696162	1160.234192	-1.233726861	9.26E-25	5.40E-23
ENSG00000157193	LRP8	7710.316534	1888.031628	2.029415163	1.08E-24	6.28E-23
ENSG00000125827	TMX4	3326.363035	1369.372561	1.279718878	1.15E-24	6.68E-23
ENSG00000198947	DMD	1.482499455	297.79223	-7.677257055	1.20E-24	6.96E-23
ENSG00000167969	ECI1	2381.863141	5158.677692	-1.11473103	1.25E-24	7.25E-23
ENSG00000219665	ZNF433-AS1	323.0108433	90.93101168	1.825982592	2.31E-24	1.33E-22
ENSG00000187187	ZNF546	393.5698881	94.97585301	2.056191015	2.34E-24	1.34E-22
ENSG00000171346	KRT15	621.6682603	187.544648	1.731169599	2.36E-24	1.35E-22
ENSG00000021645	NRXN3	3.241205243	187.7968132	-5.844940849	2.37E-24	1.36E-22
ENSG00000068078	FGFR3	228.3663197	862.17892	-1.914921878	2.45E-24	1.40E-22
ENSG00000152818	UTRN	2105.119626	4532.586906	-1.106395192	2.61E-24	1.49E-22
ENSG00000153707	PTPRD	7.591034486	143.8938566	-4.240207895	2.76E-24	1.57E-22
ENSG00000178573	MAF	315.371598	80.8887998	1.965095384	2.85E-24	1.61E-22
ENSG00000081320	STK17B	1074.147613	2490.336213	-1.213394285	2.98E-24	1.68E-22
ENSG00000131013	PPIL4	567.166323	1248.308503	-1.138330324	3.54E-24	2.00E-22

ENSG00000185650	ZFP36L1	9724.815425	3705.013134	1.392410992	3.68E-24	2.07E-22
ENSG00000184588	PDE4B	180.6444006	27.34692776	2.729568151	3.79E-24	2.13E-22
ENSG00000043355	ZIC2	747.6253824	1930.177419	-1.368028236	5.24E-24	2.94E-22
ENSG00000164749	HNF4G	169.4222778	497.3422118	-1.554213528	5.36E-24	3.00E-22
ENSG00000164236	ANKRD33B	9.905951608	151.7215246	-3.948615798	6.02E-24	3.36E-22
ENSG00000158292	GPR153	540.0394402	190.3021014	1.50750523	6.14E-24	3.42E-22
ENSG00000066629	EML1	80.13933168	307.1289997	-1.936792257	6.19E-24	3.44E-22
ENSG00000108846	ABCC3	9552.080271	3093.19412	1.626972545	6.35E-24	3.52E-22
ENSG00000109436	TBC1D9	617.3447415	228.8645152	1.430626882	6.41E-24	3.54E-22
ENSG00000126016	AMOT	1603.240531	644.9869734	1.313838352	6.50E-24	3.59E-22
ENSG00000214456	PLIN5	51.2026897	368.6615282	-2.846636968	6.54E-24	3.60E-22
ENSG00000153071	DAB2	237.3418593	54.22283132	2.126214835	6.63E-24	3.64E-22
ENSG00000120265	PCMT1	971.5258188	2446.839344	-1.332810995	6.65E-24	3.65E-22
ENSG00000151151	IPMK	435.589177	1025.067603	-1.235044792	6.68E-24	3.66E-22
ENSG00000177225	GATD1	1819.62964	3495.501626	-0.941976338	7.21E-24	3.93E-22
ENSG00000170011	MYRIP	424.8742696	117.0193341	1.8642336	7.88E-24	4.29E-22
ENSG00000135597	REPS1	996.2142761	2048.11337	-1.040041446	7.89E-24	4.29E-22
ENSG00000081923	ATP8B1	1344.672892	3513.913154	-1.385465772	8.04E-24	4.36E-22
ENSG00000079112	CDH17	493.5867656	2279.218632	-2.207085717	8.30E-24	4.49E-22
ENSG00000105281	SLC1A5	33014.27794	14753.92087	1.162002661	8.86E-24	4.78E-22
ENSG00000175220	ARHGAP1	3838.158107	2026.799175	0.921021646	1.18E-23	6.36E-22
ENSG00000198363	ASPH	20136.91321	9244.022154	1.123205831	1.21E-23	6.48E-22
ENSG00000155465	SLC7A7	1210.798291	3111.69888	-1.36135428	1.31E-23	7.02E-22
ENSG00000028137	TNFRSF1B	437.7393468	90.28441184	2.280753367	1.34E-23	7.18E-22
ENSG00000092964	DPYSL2	8796.181088	4497.056607	0.967792392	1.35E-23	7.22E-22
ENSG00000117707	PROX1	184.5715269	17.50813587	3.423188302	1.52E-23	8.07E-22
ENSG00000142871	CYR61	5249.115534	1241.015747	2.079972098	1.72E-23	9.12E-22
ENSG00000067225	PKM	95127.26836	33289.25215	1.514775442	1.74E-23	9.23E-22
ENSG00000183186	C2CD4C	270.2809364	56.46194915	2.25483316	1.93E-23	1.02E-21
ENSG00000112541	PDE10A	1.800404184	203.4750289	-6.807473605	1.95E-23	1.03E-21
ENSG00000196110	ZNF699	614.1022887	221.8843128	1.466830311	2.28E-23	1.20E-21
ENSG00000253958	CLDN23	70.96432995	336.7573919	-2.242506248	2.45E-23	1.29E-21
ENSG00000156011	PSD3	377.6522883	907.3378361	-1.263890978	2.54E-23	1.33E-21
ENSG00000049130	KITLG	201.2898401	762.5046818	-1.922138386	2.67E-23	1.40E-21
ENSG00000146457	WTAP	1295.219253	2779.802573	-1.102053309	3.15E-23	1.65E-21
ENSG00000149925	ALDOA	1112.226588	430.2230097	1.368999895	3.59E-23	1.87E-21
ENSG00000171759	PAH	128.0242233	9.187371224	3.815865873	4.42E-23	2.30E-21
ENSG00000101230	ISM1	251.7907525	54.78138918	2.193435449	5.20E-23	2.70E-21
ENSG00000019582	CD74	15.33840012	139.4061871	-3.189159057	6.32E-23	3.27E-21
ENSG00000120262	CCDC170	1.414796324	239.7618412	-7.368219512	6.46E-23	3.34E-21
ENSG00000171817	ZNF540	190.2773391	28.2527929	2.763646589	7.88E-23	4.07E-21
ENSG00000185561	TLCD2	245.6126276	748.7903669	-1.607821384	8.42E-23	4.33E-21
ENSG00000166689	PLEKHA7	1202.709802	2476.278608	-1.041622193	8.97E-23	4.61E-21

ENSG00000177311	ZBTB38	3729.43634	1991.100603	0.905303653	9.03E-23	4.63E-21
ENSG00000083857	FAT1	237712.3988	100847.9745	1.237024901	1.01E-22	5.14E-21
ENSG00000114923	SLC4A3	2125.633116	917.7404879	1.212334325	1.01E-22	5.14E-21
ENSG00000170484	KRT74	114.5964335	2.682727676	5.402092623	1.03E-22	5.25E-21
ENSG00000129595	EPB41L4A	134.1073956	16.36748831	3.038188113	1.04E-22	5.29E-21
ENSG00000144815	NXPE3	1262.187247	568.0094982	1.152777164	1.12E-22	5.66E-21
ENSG00000135090	TAOK3	337.9875769	799.855356	-1.241852047	1.21E-22	6.14E-21
ENSG00000178035	IMPDH2	3780.505126	7591.95138	-1.005965693	1.26E-22	6.35E-21
ENSG00000177106	EPS8L2	4927.374156	15379.75595	-1.642054471	1.27E-22	6.41E-21
ENSG00000130023	ERMARD	571.2547089	1294.893953	-1.180722569	1.41E-22	7.07E-21
ENSG00000184731	FAM110C	695.6277095	290.8695143	1.258218949	1.55E-22	7.74E-21
ENSG00000049192	ADAMTS6	112.4273851	2.874811761	5.229997252	1.55E-22	7.74E-21
ENSG00000256294	ZNF225	611.1765884	165.8799805	1.886089728	1.61E-22	8.05E-21
ENSG00000181045	SLC26A11	159.8044275	518.3271846	-1.695683174	1.64E-22	8.16E-21
ENSG00000112096	SOD2	695.657838	1776.99137	-1.353371085	1.75E-22	8.72E-21
ENSG00000132623	ANKEF1	1353.481969	602.6311911	1.166822463	2.07E-22	1.03E-20
ENSG00000168646	AXIN2	21537.0066	11275.30645	0.933723809	2.47E-22	1.23E-20
ENSG00000051620	HEBP2	921.9484808	1819.185452	-0.980679396	2.50E-22	1.24E-20
ENSG00000121380	BCL2L14	22.81876311	190.7416065	-3.059561007	2.55E-22	1.26E-20
ENSG00000112559	MDFI	364.1141186	103.1465285	1.815996613	2.55E-22	1.26E-20
ENSG00000153250	RBMS1	371.4860361	112.6483069	1.719928332	2.63E-22	1.29E-20
ENSG00000151790	TDO2	135.5569238	6.862270124	4.292638096	2.84E-22	1.39E-20
ENSG00000151715	TMEM45B	2852.930966	1473.045257	0.95399515	2.88E-22	1.41E-20
ENSG00000133639	BTG1	1391.202585	665.9768962	1.062503052	3.14E-22	1.54E-20
ENSG00000163884	KLF15	367.5455935	973.6567652	-1.405846601	3.20E-22	1.56E-20
ENSG00000095303	PTGS1	21.84950663	198.0052838	-3.179430709	3.31E-22	1.61E-20
ENSG00000171703	TCEA2	220.0506851	24.94863866	3.131727635	3.48E-22	1.69E-20
ENSG00000120254	MTHFD1L	1187.090444	3197.94062	-1.430065045	3.70E-22	1.79E-20
ENSG00000204531	POU5F1	15.28893057	138.4418969	-3.18075158	3.86E-22	1.87E-20
ENSG00000166347	CYB5A	841.3016027	1738.117455	-1.046978925	3.86E-22	1.87E-20
ENSG00000143772	ITPKB	1687.429157	767.2750029	1.136350227	4.09E-22	1.97E-20
ENSG00000166387	PPFIBP2	477.8836908	1094.147008	-1.194746284	4.31E-22	2.08E-20
ENSG00000283431	AC099654.6	135.1145518	1.958119017	6.212905965	5.13E-22	2.46E-20
ENSG00000128710	HOXD10	872.5212838	313.2365232	1.479873856	5.78E-22	2.77E-20
ENSG00000188766	SPRED3	461.7341036	117.0472568	1.985183836	6.08E-22	2.91E-20
ENSG00000177335	C8orf31	18.76860501	213.6524104	-3.50316743	7.06E-22	3.37E-20
ENSG00000174915	PTDSS2	703.1435836	1467.806214	-1.06177574	7.57E-22	3.61E-20
ENSG00000168398	BDKRB2	213.4510908	606.9331607	-1.506596732	7.65E-22	3.64E-20
ENSG00000055211	GINM1	608.545516	1388.267412	-1.190494733	7.69E-22	3.65E-20
ENSG00000167004	PDIA3	14446.1156	25986.2717	-0.847066928	1.01E-21	4.78E-20
ENSG00000157227	MMP14	497.7514134	1148.038574	-1.205226756	1.16E-21	5.49E-20
ENSG00000122779	TRIM24	1050.710472	2310.652304	-1.137386246	1.20E-21	5.65E-20
ENSG00000140807	NKD1	9559.857246	4459.606644	1.100177433	1.30E-21	6.15E-20

ENSG00000134954	ETS1	242.297532	50.16218496	2.263809843	1.34E-21	6.29E-20
ENSG00000111912	NCOA7	964.0005671	1906.279667	-0.983737756	1.51E-21	7.08E-20
ENSG00000231789	PIK3CD-AS2	4.312092104	112.3627734	-4.690372563	1.62E-21	7.61E-20
ENSG00000031081	ARHGAP31	768.6665895	337.1027315	1.188869319	1.64E-21	7.65E-20
ENSG00000144908	ALDH1L1	1030.942454	2437.829721	-1.241367687	1.72E-21	8.05E-20
ENSG00000254004	ZNF260	2124.137205	899.6037216	1.240159636	1.85E-21	8.61E-20
ENSG00000058866	DGKG	202.7709182	39.48040937	2.350889449	2.45E-21	1.14E-19
ENSG00000161011	SQSTM1	23119.23488	10530.82023	1.134466218	2.62E-21	1.22E-19
ENSG00000033327	GAB2	525.8748161	1131.08304	-1.105239645	2.66E-21	1.23E-19
ENSG00000225530	SP3P	0	500.2182098	-11.2837881	3.20E-21	1.48E-19
ENSG00000130363	RSPH3	423.1277532	963.9006737	-1.187882336	3.71E-21	1.71E-19
ENSG00000205978	NYNRIN	281.8764581	814.8701775	-1.530122087	3.87E-21	1.78E-19
ENSG00000107719	PALD1	9904.708679	3780.050293	1.389463443	3.95E-21	1.81E-19
ENSG00000161642	ZNF385A	1198.211416	437.0618263	1.453129042	4.56E-21	2.09E-19
ENSG00000233429	HOTAIRM1	459.688272	172.3602322	1.415342508	5.30E-21	2.43E-19
ENSG00000177156	TALDO1	2893.844659	6368.494031	-1.138046254	5.60E-21	2.56E-19
ENSG00000244342	LINC00698	109.9715165	3.190170014	5.173318058	6.14E-21	2.80E-19
ENSG00000175920	DOK7	1866.411798	865.8404128	1.108948787	7.15E-21	3.26E-19
ENSG00000272405	AL365181.3	4423.536798	2268.803976	0.963019157	7.35E-21	3.34E-19
ENSG00000155906	RMND1	327.5885055	773.8241558	-1.241109588	7.41E-21	3.36E-19
ENSG00000197050	ZNF420	531.3245788	173.8343624	1.614925078	8.09E-21	3.67E-19
ENSG00000197415	VEPH1	291.6336983	72.93208578	2.003972398	8.38E-21	3.79E-19
ENSG00000237664	LINC00316	313.9669585	75.37170488	2.055729627	8.44E-21	3.81E-19
ENSG00000138709	LARP1B	973.088222	1971.753359	-1.018933521	9.35E-21	4.21E-19
ENSG00000163814	CDCP1	8667.248454	3826.323448	1.179382034	9.44E-21	4.25E-19
ENSG00000075673	ATP12A	105.0656161	6.342540823	4.051025791	1.07E-20	4.80E-19
ENSG00000237172	B3GNT9	281.9955428	654.9713325	-1.215569092	1.09E-20	4.86E-19
ENSG00000260077	AC104794.2	326.5764433	100.0524917	1.710509752	1.11E-20	4.95E-19
ENSG00000204264	PSMB8	344.0637462	852.7182762	-1.309338973	1.29E-20	5.74E-19
ENSG00000157557	ETS2	5788.772074	14670.49875	-1.341525161	1.39E-20	6.21E-19
ENSG00000128510	CPA4	130.0512129	17.59807995	2.877205812	1.42E-20	6.33E-19
ENSG00000102554	KLF5	3609.430309	6702.428303	-0.892844328	1.63E-20	7.23E-19
ENSG00000057294	PKP2	4104.035842	7825.488335	-0.9310622	1.63E-20	7.23E-19
ENSG00000183742	MACC1	803.6388238	2068.074534	-1.364314063	1.64E-20	7.23E-19
ENSG00000157404	KIT	157.1243509	27.3832676	2.526535985	1.73E-20	7.65E-19
ENSG00000157613	CREB3L1	11.85238708	126.2788767	-3.401657991	1.82E-20	8.01E-19
ENSG00000142694	EVA1B	81.09685576	284.7774699	-1.80954739	1.93E-20	8.50E-19
ENSG00000104341	LAPTM4B	6117.003098	2945.477126	1.054092297	2.02E-20	8.85E-19
ENSG00000185386	MAPK11	755.6207461	281.2168124	1.426557544	2.02E-20	8.86E-19
ENSG00000136155	SCEL	15.80233614	195.3077804	-3.617817823	2.11E-20	9.24E-19
ENSG00000139174	PRICKLE1	113.8566051	436.0602328	-1.93688582	2.49E-20	1.09E-18
ENSG00000174672	BRSK2	37.29096968	195.1031487	-2.383660658	2.59E-20	1.13E-18
ENSG00000100453	GZMB	219.5006498	0.855270664	7.924803979	2.61E-20	1.14E-18

ENSG00000146414	SHPRH	330.1970691	821.6152983	-1.315212283	2.67E-20	1.16E-18
ENSG00000108759	KRT32	103.3070729	2.504389913	5.413050461	2.67E-20	1.16E-18
ENSG00000111859	NEDD9	8.998059663	120.4695254	-3.734129785	3.14E-20	1.36E-18
ENSG00000112414	ADGRG6	1135.040153	2263.65217	-0.995991711	3.40E-20	1.47E-18
ENSG00000010404	IDS	2900.056532	1602.274465	0.855740777	3.67E-20	1.58E-18
ENSG00000176136	MC5R	153.6059945	1.240898196	6.991425559	3.71E-20	1.60E-18
ENSG00000140678	ITGAX	426.0073144	102.3435978	2.059865592	4.10E-20	1.76E-18
ENSG00000106665	CLIP2	1794.696196	4086.15445	-1.187052829	4.22E-20	1.81E-18
ENSG00000076356	PLXNA2	34.54731375	186.6419238	-2.433569765	5.24E-20	2.25E-18
ENSG00000143797	MBOAT2	3965.371154	2020.815957	0.972865354	5.30E-20	2.26E-18
ENSG00000100596	SPTLC2	1262.882997	2367.704031	-0.906564866	5.30E-20	2.26E-18
ENSG00000039068	CDH1	13210.20584	26568.83112	-1.008036116	5.44E-20	2.32E-18
ENSG00000163947	ARHGEF3	1036.348317	454.3310751	1.18822994	5.67E-20	2.41E-18
ENSG00000129083	COPB1	4009.229372	7006.121333	-0.805291638	5.97E-20	2.53E-18
ENSG00000123104	ITPR2	1634.349706	3007.239394	-0.879781195	6.30E-20	2.67E-18
ENSG00000152229	PSTPIP2	177.6259709	659.9677865	-1.894039882	6.36E-20	2.69E-18
ENSG00000181019	NQO1	105307.7438	36288.91256	1.536984106	6.38E-20	2.69E-18
ENSG00000124762	CDKN1A	3474.975089	1391.389356	1.320067562	7.16E-20	3.02E-18
ENSG00000187210	GCNT1	279.2143123	728.8235893	-1.383254987	7.28E-20	3.07E-18
ENSG00000164830	OXR1	3858.577786	1716.57038	1.16801158	8.36E-20	3.51E-18
ENSG00000167996	FTH1	56987.9255	31002.39485	0.878269303	8.55E-20	3.59E-18
ENSG00000155158	TTC39B	941.9898009	1801.23226	-0.93514722	8.66E-20	3.63E-18
ENSG00000164574	GALNT10	2130.846125	1120.47797	0.926935181	9.09E-20	3.80E-18
ENSG00000155629	PIK3AP1	1059.433943	397.9390435	1.410782209	9.13E-20	3.81E-18
ENSG00000120278	PLEKHG1	245.9176814	651.338686	-1.404285331	9.17E-20	3.82E-18
ENSG00000134086	VHL	2661.120226	5096.934824	-0.9375177	1.02E-19	4.25E-18
ENSG00000145506	NKD2	2043.331907	875.5674488	1.223556659	1.18E-19	4.91E-18
ENSG00000205809	KLRC2	284.5126245	80.54981867	1.824584035	1.27E-19	5.27E-18
ENSG00000179168	GGN	221.9045089	53.64650076	2.05452633	1.27E-19	5.28E-18
ENSG00000149179	C11orf49	1658.835199	805.1607703	1.043632071	1.32E-19	5.44E-18
ENSG00000185507	IRF7	451.3902431	1025.678655	-1.184273816	1.36E-19	5.60E-18
ENSG00000213066	FGFR1OP	256.1226762	666.2627687	-1.379982246	1.38E-19	5.70E-18
ENSG00000166123	GPT2	2478.45675	5021.142478	-1.018567867	1.72E-19	7.08E-18
ENSG00000172915	NBEA	469.4099899	182.2565637	1.364692587	1.79E-19	7.33E-18
ENSG00000069667	RORA	2866.884334	1422.718573	1.010896561	1.86E-19	7.63E-18
ENSG00000108448	TRIM16L	2191.65925	699.7655473	1.645889757	1.97E-19	8.04E-18
ENSG00000110768	GTF2H1	829.0650403	1644.310549	-0.988080264	2.07E-19	8.46E-18
ENSG00000162599	NFIA	773.0652462	1501.540728	-0.957906577	2.14E-19	8.72E-18
ENSG00000058335	RASGRF1	226.9359811	57.22040894	1.984772794	2.30E-19	9.35E-18
ENSG00000117500	TMED5	1075.224861	2280.662529	-1.084885123	2.36E-19	9.59E-18
ENSG00000001036	FUCA2	1787.472401	3712.343439	-1.054656174	2.43E-19	9.84E-18
ENSG00000176485	PLA2G16	430.9943744	1031.577353	-1.259012732	2.44E-19	9.90E-18
ENSG00000027697	IFNGR1	500.42195	1098.783099	-1.134944232	2.68E-19	1.08E-17

ENSG00000123094	RASSF8	656.4492803	287.4230633	1.19136549	2.85E-19	1.15E-17
ENSG00000171345	KRT19	48048.98785	25077.49263	0.938149518	2.99E-19	1.20E-17
ENSG00000149100	EIF3M	3548.627287	6689.503308	-0.914670747	3.25E-19	1.31E-17
ENSG00000125398	SOX9	21168.13413	11455.51408	0.885870496	3.45E-19	1.38E-17
ENSG00000213903	LTB4R	889.6274561	415.7488298	1.097850513	3.46E-19	1.39E-17
ENSG00000089057	SLC23A2	3242.242132	1684.640341	0.94433343	3.93E-19	1.58E-17
ENSG00000141934	PLPP2	4170.948657	2072.292641	1.008844598	4.26E-19	1.70E-17
ENSG00000112182	BACH2	135.7882498	25.22816537	2.434509948	4.32E-19	1.72E-17
ENSG00000121680	PEX16	973.7785667	469.4676459	1.053251792	4.51E-19	1.80E-17
ENSG00000139289	PHLDA1	10485.95529	4668.134965	1.167409767	4.61E-19	1.83E-17
ENSG00000172771	EFCAB12	185.53618	41.70716194	2.155203147	5.24E-19	2.08E-17
ENSG00000183734	ASCL2	2259.236915	6619.281478	-1.550683514	5.91E-19	2.34E-17
ENSG00000104419	NDRG1	7339.896943	2243.552936	1.710397855	6.53E-19	2.59E-17
ENSG00000172818	OVOL1	99.52265868	327.6481333	-1.720498892	6.87E-19	2.72E-17
ENSG00000165655	ZNF503	1472.432282	663.4331949	1.149415495	7.46E-19	2.95E-17
ENSG00000175390	EIF3F	3662.128904	6342.877576	-0.792432218	8.22E-19	3.24E-17
ENSG00000135002	RFK	408.4973176	912.4620977	-1.160486219	8.25E-19	3.24E-17
ENSG00000110042	DTX4	906.5831394	2082.620167	-1.199209111	9.07E-19	3.56E-17
ENSG00000180891	CUEDC1	2400.48842	1273.318278	0.915202196	9.44E-19	3.70E-17
ENSG00000078814	MYH7B	527.8370966	184.3318845	1.519494282	9.67E-19	3.79E-17
ENSG00000169242	EFNA1	773.7202116	2245.905734	-1.537149345	1.06E-18	4.14E-17
ENSG00000006047	YBX2	1168.235003	499.1620989	1.228263396	1.10E-18	4.28E-17
ENSG00000133812	SBF2	1107.909892	2104.851275	-0.92566523	1.25E-18	4.86E-17
ENSG00000129422	MTUS1	1084.194179	2055.811757	-0.923307448	1.37E-18	5.33E-17
ENSG00000153132	CLGN	196.011194	48.42209228	2.023570693	1.54E-18	6.00E-17
ENSG00000143847	PPFIA4	407.5624762	82.02358445	2.320773006	1.58E-18	6.12E-17
ENSG00000206337	HCP5	23.02218646	185.6520558	-3.005577691	1.58E-18	6.13E-17
ENSG00000100504	PYGL	88.76605582	341.6432235	-1.94488845	1.62E-18	6.26E-17
ENSG00000157833	GAREM2	37.50962137	171.933444	-2.199625281	1.65E-18	6.38E-17
ENSG00000002746	HECW1	126.4475506	20.41687707	2.639106999	1.67E-18	6.42E-17
ENSG00000055208	TAB2	1469.240765	2921.038458	-0.991522224	1.92E-18	7.38E-17
ENSG00000197355	UAP1L1	329.5756946	827.5000517	-1.327138185	2.34E-18	9.00E-17
ENSG00000158158	CNNM4	597.061445	1259.414332	-1.077352321	2.38E-18	9.12E-17
ENSG00000189334	S100A14	3389.512349	7270.983854	-1.100953953	2.39E-18	9.16E-17
ENSG00000125148	MT2A	8302.322887	4532.63567	0.873277234	2.55E-18	9.75E-17
ENSG00000124191	TOX2	959.7700018	338.1763672	1.503276574	2.60E-18	9.92E-17
ENSG00000120549	KIAA1217	2886.13595	1345.653695	1.100327745	2.61E-18	9.94E-17
ENSG00000187123	LYPD6	224.2077048	516.1699979	-1.202546637	2.76E-18	1.05E-16
ENSG00000196923	PDLIM7	2909.321532	1169.411491	1.314118759	2.85E-18	1.08E-16
ENSG00000184860	SDR42E1	551.9644365	198.9014417	1.476022916	2.89E-18	1.10E-16
ENSG00000138193	PLCE1	222.671612	645.5467175	-1.53474736	2.93E-18	1.11E-16
ENSG00000071994	PDCD2	1238.336262	2432.802219	-0.974322395	3.05E-18	1.16E-16
ENSG00000099834	CDHR5	41.55723954	733.8096177	-4.142795532	3.18E-18	1.20E-16

ENSG00000002834	LASP1	21290.04448	12006.46169	0.826318869	3.49E-18	1.32E-16
ENSG00000092929	UNC13D	3914.49566	7620.07355	-0.960842021	3.74E-18	1.41E-16
ENSG00000140323	DISP2	128.8304848	22.60196065	2.510837635	3.95E-18	1.48E-16
ENSG00000119888	EPCAM	14782.1748	24546.87464	-0.731667035	3.98E-18	1.49E-16
ENSG00000173482	PTPRM	110.0187489	7.441441101	3.909564777	4.12E-18	1.54E-16
ENSG00000001617	SEMA3F	385.7337401	1629.897727	-2.078252611	4.13E-18	1.55E-16
ENSG00000101871	MID1	1485.971456	593.9945214	1.321587333	4.19E-18	1.57E-16
ENSG00000205339	IPO7	7975.737448	15356.39417	-0.945206155	4.50E-18	1.68E-16
ENSG00000154153	RETREG1	349.7535395	843.9980597	-1.270362253	4.76E-18	1.77E-16
ENSG00000143776	CDC42BPA	7741.325024	4383.175157	0.820462299	5.00E-18	1.86E-16
ENSG00000169047	IRS1	7157.446857	2889.133877	1.308500722	5.22E-18	1.94E-16
ENSG00000140961	OSGIN1	2948.152528	315.8831584	3.221283816	5.24E-18	1.94E-16
ENSG00000137193	PIM1	822.0933394	1665.886641	-1.018716468	5.88E-18	2.18E-16
ENSG00000077063	CTTNBP2	338.3612786	108.6813834	1.641355775	6.40E-18	2.36E-16
ENSG00000169035	KLK7	2831.152176	915.7470832	1.627500008	6.56E-18	2.42E-16
ENSG00000111424	VDR	1173.541226	588.9258173	0.994212939	6.79E-18	2.50E-16
ENSG00000273443	AL645608.8	149.6898213	33.47333161	2.161557441	7.74E-18	2.85E-16
ENSG00000134686	PHC2	5033.37266	2547.916061	0.981914491	7.78E-18	2.86E-16
ENSG00000198431	TXNRD1	59734.80478	20134.49915	1.568854788	7.91E-18	2.90E-16
ENSG00000206538	VGLL3	17.01116918	118.6282437	-2.798014024	7.96E-18	2.92E-16
ENSG00000258102	MAP1LC3B2	166.4560201	37.58311309	2.156015194	8.31E-18	3.04E-16
ENSG00000167123	CERCAM	312.2681847	778.8817449	-1.317238688	8.37E-18	3.06E-16
ENSG00000049246	PER3	749.5294662	338.6299576	1.146752498	8.48E-18	3.09E-16
ENSG00000144369	FAM171B	9287.512017	4452.377555	1.060626783	8.50E-18	3.10E-16
ENSG00000115221	ITGB6	160.7175507	38.13041334	2.073018515	8.62E-18	3.14E-16
ENSG00000170608	FOXA3	64.59782218	254.5979987	-1.975015795	9.02E-18	3.27E-16
ENSG00000254632	AP003119.1	82.03725362	5.850304168	3.832202167	9.66E-18	3.50E-16
ENSG00000125430	HS3ST3B1	20.73042019	121.7496166	-2.555533391	1.02E-17	3.70E-16
ENSG00000275395	FCGBP	650.2427705	152.8858208	2.092851276	1.06E-17	3.82E-16
ENSG00000164342	TLR3	76.74163271	287.8791424	-1.906213895	1.07E-17	3.85E-16
ENSG00000253741	LNCOC1	375.8174691	137.6744334	1.451970005	1.13E-17	4.09E-16
ENSG00000232762	AL355483.3	107.9757517	5.943166968	4.169534759	1.20E-17	4.31E-16
ENSG00000138670	RASGEF1B	93.15218965	9.801422686	3.262505838	1.27E-17	4.56E-16
ENSG00000198435	NRARP	1554.298542	686.0815286	1.179038597	1.27E-17	4.56E-16
ENSG00000164951	PDP1	6920.537676	2303.676022	1.586538724	1.32E-17	4.74E-16
ENSG00000197822	OCLN	1806.129241	3462.874011	-0.939161627	1.34E-17	4.78E-16
ENSG00000063660	GPC1	10937.03153	4438.538847	1.300868753	1.35E-17	4.81E-16
ENSG00000230316	FEZF1-AS1	213.4707681	584.2308982	-1.450928699	1.55E-17	5.54E-16
ENSG00000110422	HIPK3	2487.954422	4658.219221	-0.90465848	1.63E-17	5.81E-16
ENSG00000204876	AC021218.1	206.684714	59.14115876	1.80906744	1.72E-17	6.13E-16
ENSG00000116260	QSOX1	8382.281209	4691.891566	0.83710163	1.75E-17	6.23E-16
ENSG00000198121	LPAR1	346.4879247	129.6764931	1.418330371	1.77E-17	6.27E-16
ENSG00000084112	SSH1	5014.167259	2374.854623	1.077905953	1.83E-17	6.47E-16

ENSG00000106541	AGR2	6.198433007	778.9670332	-6.975131564	1.85E-17	6.55E-16
ENSG00000124783	SSR1	3900.424979	8035.454866	-1.042702428	1.90E-17	6.71E-16
ENSG00000140450	ARRDC4	698.8394057	226.6226353	1.622512548	1.91E-17	6.75E-16
ENSG00000055163	CYFIP2	1380.695576	2422.503686	-0.810989484	2.00E-17	7.04E-16
ENSG00000212864	RNF208	119.1795007	338.9761573	-1.507028517	2.15E-17	7.57E-16
ENSG00000008311	AASS	690.4762332	1518.575652	-1.136593975	2.40E-17	8.43E-16
ENSG00000135127	BICDL1	1058.591665	454.0034811	1.221404624	2.50E-17	8.76E-16
ENSG00000128268	MGAT3	189.8776102	26.55753555	2.844644613	2.52E-17	8.83E-16
ENSG00000118257	NRP2	499.1670032	156.6315914	1.669826307	2.57E-17	8.97E-16
ENSG00000135318	NT5E	242.7730491	71.39637688	1.767827684	3.03E-17	1.06E-15
ENSG00000160867	FGFR4	22.49181727	139.5900234	-2.634919948	3.25E-17	1.13E-15
ENSG00000129562	DAD1	5082.077785	2772.476611	0.874048346	3.28E-17	1.14E-15
ENSG00000267309	AC092295.2	195.3061087	47.22587281	2.050339769	3.40E-17	1.18E-15
ENSG00000134574	DDB2	1566.201781	712.1913639	1.135851199	3.47E-17	1.20E-15
ENSG00000172965	MIR4435-2HG	1224.956372	391.6473448	1.642994811	3.62E-17	1.25E-15
ENSG00000138642	HERC6	560.1311971	1091.054027	-0.962335372	3.85E-17	1.33E-15
ENSG00000164091	WDR82	5004.314556	8999.961714	-0.846811903	3.98E-17	1.38E-15
ENSG00000135439	AGAP2	49.70860921	186.9061792	-1.909329904	4.03E-17	1.39E-15
ENSG00000164506	STXBP5	385.7759068	807.2942826	-1.065573353	4.34E-17	1.50E-15
ENSG00000267922	AC007785.1	87.43862876	7.18011709	3.599592189	4.34E-17	1.50E-15
ENSG00000174567	GOLT1A	328.1781871	106.6554055	1.626509057	4.52E-17	1.56E-15
ENSG00000133818	RRAS2	971.798518	2079.594667	-1.097775705	4.66E-17	1.60E-15
ENSG00000130164	LDLR	11865.25507	6395.091425	0.891579875	4.83E-17	1.66E-15
ENSG00000068079	IFI35	157.0905862	527.7461063	-1.746979632	4.90E-17	1.68E-15
ENSG00000068024	HDAC4	1972.270034	1091.143423	0.854081272	5.01E-17	1.71E-15
ENSG00000060709	RIMBP2	79.62212745	3.373406901	4.580486505	5.34E-17	1.82E-15
ENSG00000100311	PDGFB	1864.204721	989.5907427	0.913855308	5.48E-17	1.87E-15
ENSG00000139737	SLAIN1	557.7771215	250.1636473	1.155874172	5.53E-17	1.88E-15
ENSG00000155660	PDIA4	11138.59095	24030.39469	-1.10928391	5.82E-17	1.98E-15
ENSG00000106366	SERPINE1	288.4096081	59.09956808	2.27981555	6.02E-17	2.05E-15
ENSG00000144677	CTDSPL	6801.77461	3830.030536	0.82865626	6.10E-17	2.07E-15
ENSG00000165548	TMEM63C	349.4619647	122.5937079	1.508017842	6.60E-17	2.23E-15
ENSG00000221926	TRIM16	2100.132101	1039.964595	1.013223145	7.84E-17	2.65E-15
ENSG00000189042	ZNF567	544.0815245	237.448936	1.197890201	8.41E-17	2.84E-15
ENSG00000177542	SLC25A22	1155.69841	2127.942865	-0.881004501	8.49E-17	2.86E-15
ENSG00000159348	CYB5R1	282.7031792	600.1359218	-1.085379659	9.96E-17	3.35E-15
ENSG00000153291	SLC25A27	576.0938481	209.2534605	1.463016258	1.06E-16	3.56E-15
ENSG00000204536	CCHCR1	1236.063217	2354.726328	-0.929736561	1.14E-16	3.82E-15
ENSG00000185127	C6orf120	627.9264769	1234.97178	-0.976144986	1.14E-16	3.83E-15
ENSG00000120253	NUP43	1037.016179	2394.809986	-1.207800529	1.15E-16	3.86E-15
ENSG00000111266	DUSP16	1424.246634	2860.584982	-1.005837582	1.19E-16	3.98E-15
ENSG00000126561	STAT5A	143.6843492	411.3029347	-1.516520576	1.24E-16	4.13E-15
ENSG00000164684	ZNF704	1919.593883	889.3224546	1.110292801	1.25E-16	4.16E-15

ENSG00000256340	ABCC6P1	2.909028184	85.67413312	-4.881696002	1.30E-16	4.34E-15
ENSG00000171843	MLLT3	324.7755596	745.163182	-1.19729062	1.31E-16	4.37E-15
ENSG00000148400	NOTCH1	2820.872644	1554.261271	0.859657039	1.33E-16	4.41E-15
ENSG00000078246	TULP3	1440.399219	2663.098685	-0.886845341	1.49E-16	4.94E-15
ENSG00000109787	KLF3	1060.835215	1989.216987	-0.907005665	1.74E-16	5.76E-15
ENSG00000149177	PTPRJ	3873.496501	2073.777327	0.901312251	1.74E-16	5.76E-15
ENSG00000179954	SSC5D	172.8691436	37.75896214	2.205121113	1.83E-16	6.05E-15
ENSG00000152270	PDE3B	128.2217987	381.7002368	-1.575261881	1.86E-16	6.15E-15
ENSG00000118496	FBXO30	594.2857851	1262.161271	-1.086902766	1.91E-16	6.30E-15
ENSG00000164932	CTHRC1	422.0127906	159.1544046	1.405254418	1.98E-16	6.52E-15
ENSG00000144355	DLX1	34.76596544	170.2464345	-2.296273581	2.14E-16	7.04E-15
ENSG00000128805	ARHGAP22	197.1938597	43.63339749	2.182253894	2.14E-16	7.04E-15
ENSG00000225975	LINC01534	158.7827074	31.7102154	2.331146143	2.24E-16	7.35E-15
ENSG00000164867	NOS3	142.7136602	24.01621918	2.584144948	2.25E-16	7.35E-15
ENSG00000102081	FMR1	2588.851656	1329.480584	0.961133553	2.33E-16	7.62E-15
ENSG00000176945	MUC20	377.8509599	1139.418893	-1.591775826	2.46E-16	8.04E-15
ENSG00000233369	GTF2IP4	488.3461789	1036.995241	-1.085784248	2.48E-16	8.09E-15
ENSG00000144136	SLC20A1	6765.018041	2564.782876	1.398897467	2.49E-16	8.11E-15
ENSG00000142065	ZFP14	908.9020685	300.3956063	1.59952944	2.66E-16	8.66E-15
ENSG00000149150	SLC43A1	898.0283629	1786.402029	-0.992388944	2.69E-16	8.75E-15
ENSG00000105771	SMG9	3653.6064	2191.18496	0.737604801	2.84E-16	9.21E-15
ENSG00000008256	CYTH3	1583.489457	2795.749736	-0.820004099	3.08E-16	9.99E-15
ENSG00000187244	BCAM	2673.233478	6666.260245	-1.318061502	3.10E-16	1.00E-14
ENSG00000266010	GATA6-AS1	31.23571408	157.6122335	-2.333181767	3.33E-16	1.08E-14
ENSG00000132016	C19orf57	966.9925268	357.2052271	1.438768814	3.43E-16	1.11E-14
ENSG00000234859	AC003958.2	89.08602393	1.541050765	5.880457263	3.53E-16	1.14E-14
ENSG00000143344	RGL1	331.6144166	113.2327287	1.555373284	3.59E-16	1.16E-14
ENSG00000229056	AC020571.1	103.9492322	313.053729	-1.589721971	3.72E-16	1.19E-14
ENSG00000010030	ETV7	123.8549946	341.4826059	-1.46400612	3.74E-16	1.20E-14
ENSG00000103888	CEMIP	2647.440692	889.3881863	1.574362813	4.09E-16	1.31E-14
ENSG00000104763	ASAH1	1571.88458	2866.802842	-0.866715002	4.66E-16	1.49E-14
ENSG00000135452	TSPAN31	355.0417145	877.1387925	-1.30400743	4.84E-16	1.55E-14
ENSG00000064393	HIPK2	3224.295229	1884.938527	0.774339327	4.91E-16	1.57E-14
ENSG00000110619	CARS	1926.660068	3806.649794	-0.982311655	5.01E-16	1.60E-14
ENSG00000082397	EPB41L3	75.16273409	2.050981817	5.160423493	5.05E-16	1.61E-14
ENSG00000114529	C3orf52	785.3742776	361.8206269	1.116158293	5.07E-16	1.61E-14
ENSG00000267680	ZNF224	1642.534695	504.9775483	1.703037636	5.25E-16	1.67E-14
ENSG00000048052	HDAC9	85.69291412	5.362966638	4.041503784	5.43E-16	1.72E-14
ENSG00000105426	PTPRS	377.497401	119.3661687	1.66001714	6.07E-16	1.92E-14
ENSG00000161921	CXCL16	1100.285909	2066.07176	-0.909203911	6.14E-16	1.94E-14
ENSG00000175048	ZDHHC14	85.16112361	247.8565923	-1.541904297	6.31E-16	1.99E-14
ENSG00000100097	LGALS1	1731.951331	778.1270765	1.155070529	6.51E-16	2.05E-14
ENSG00000060140	STYK1	494.0749682	213.5247196	1.207996716	6.87E-16	2.16E-14

ENSG00000164761	TNFRSF11B	207.2889623	0.631745859	8.423473835	7.05E-16	2.22E-14
ENSG00000234805	AC090505.1	76.92794107	7.31472717	3.4057732	7.31E-16	2.30E-14
ENSG00000164659	KIAA1324L	47.67227575	189.5354593	-1.992235278	8.11E-16	2.55E-14
ENSG00000100342	APOL1	35.92960415	163.7574394	-2.187439629	8.34E-16	2.61E-14
ENSG00000228594	FNDC10	1.464265867	107.3500455	-6.206815214	8.55E-16	2.68E-14
ENSG00000128383	APOBEC3A	71.0174468	3.53799834	4.302893885	8.62E-16	2.69E-14
ENSG00000099849	RASSF7	1446.843589	2510.816182	-0.794983487	8.98E-16	2.80E-14
ENSG00000258947	TUBB3	206.7956995	48.72955438	2.093677144	9.51E-16	2.97E-14
ENSG00000197261	C6orf141	289.0484369	110.3531089	1.38747143	9.87E-16	3.08E-14
ENSG00000125378	BMP4	26158.65691	12019.03181	1.121942567	9.97E-16	3.10E-14
ENSG00000228474	OST4	2147.544923	1253.273621	0.776891536	1.25E-15	3.88E-14
ENSG00000119682	AREL1	3507.051497	2000.601585	0.809793408	1.29E-15	4.01E-14
ENSG00000128739	SNRPN	73.22296239	2.159050301	5.11269573	1.38E-15	4.27E-14
ENSG00000126562	WNK4	220.7668749	68.30688751	1.695543576	1.38E-15	4.29E-14
ENSG00000146054	TRIM7	75.71371421	227.805238	-1.590420557	1.40E-15	4.34E-14
ENSG00000105255	FSD1	2.847826236	80.40058952	-4.794986298	1.42E-15	4.37E-14
ENSG00000169016	E2F6	1457.192415	669.5762253	1.120528634	1.43E-15	4.41E-14
ENSG00000166922	SCG5	29.19699207	686.3149035	-4.555821569	1.43E-15	4.42E-14
ENSG00000091986	CCDC80	223.4171369	66.89459665	1.744523109	1.44E-15	4.42E-14
ENSG00000281406	BLACAT1	122.8416624	25.75184275	2.257245419	1.54E-15	4.74E-14
ENSG00000157514	TSC22D3	331.617562	1099.332612	-1.728494944	1.81E-15	5.57E-14
ENSG00000112902	SEMA5A	0	172.1441353	-9.744421272	1.91E-15	5.87E-14
ENSG00000204529	GUCY2EP	74.24310975	1.827457011	5.358268364	1.93E-15	5.91E-14
ENSG00000237854	LINC00674	607.7459153	283.7185058	1.098756627	1.95E-15	5.97E-14
ENSG00000186529	CYP4F3	355.9955912	131.3628249	1.441196681	2.28E-15	6.97E-14
ENSG00000139438	FAM222A	587.2390416	257.5464412	1.189452845	2.31E-15	7.03E-14
ENSG00000001084	GCLC	5313.960039	1659.685603	1.678339415	2.31E-15	7.03E-14
ENSG00000020577	SAMD4A	3175.724033	1536.750949	1.0469392	2.33E-15	7.08E-14
ENSG00000186654	PRR5	502.4988856	1089.696773	-1.115950405	2.54E-15	7.73E-14
ENSG00000087086	FTL	108670.109	50488.97343	1.105899015	2.58E-15	7.84E-14
ENSG00000183161	FANCF	315.1220356	651.0816615	-1.046699504	2.68E-15	8.11E-14
ENSG00000181472	ZBTB2	587.6918985	1161.406163	-0.983420686	2.81E-15	8.50E-14
ENSG00000184574	LPAR5	6.43167098	86.70336347	-3.738734434	2.90E-15	8.77E-14
ENSG00000118194	TNNT2	71.62978022	393.0723278	-2.454972889	2.97E-15	8.98E-14
ENSG00000121742	GJB6	11.25177484	98.6825602	-3.1348424	3.03E-15	9.14E-14
ENSG00000099194	SCD	101233.7076	47011.4227	1.106587389	3.08E-15	9.28E-14
ENSG00000110713	NUP98	2997.353075	5773.227799	-0.94583528	3.10E-15	9.33E-14
ENSG00000152256	PDK1	1913.215954	961.8447944	0.992128599	3.51E-15	1.06E-13
ENSG00000169976	SF3B5	1033.458781	2046.713546	-0.986251989	3.61E-15	1.08E-13
ENSG00000166473	PKD1L2	485.7865942	153.3815755	1.667254423	3.67E-15	1.10E-13
ENSG00000103187	COTL1	5938.924318	2438.059972	1.284074777	3.74E-15	1.12E-13
ENSG00000048740	CELF2	732.704768	309.1933233	1.243005642	3.77E-15	1.13E-13
ENSG00000197006	METTL9	3612.785474	1936.533253	0.899180568	3.85E-15	1.15E-13

ENSG00000151778	SERP2	43.21573628	177.7378052	-2.038006231	4.03E-15	1.20E-13
ENSG00000114554	PLXNA1	8606.528723	5075.313292	0.761956654	4.18E-15	1.25E-13
ENSG00000148120	C9orf3	981.6635646	537.1767952	0.870142432	4.35E-15	1.30E-13
ENSG00000160284	SPATC1L	97.44731813	276.3172088	-1.504920319	4.42E-15	1.31E-13
ENSG00000146233	CYP39A1	79.7993947	7.126082848	3.472000523	4.51E-15	1.34E-13
ENSG00000179933	C14orf119	1869.927115	963.9024205	0.955314995	4.57E-15	1.36E-13
ENSG00000131015	ULBP2	126.3462342	343.7660635	-1.443731376	4.72E-15	1.40E-13
ENSG00000099953	MMP11	49.8663729	213.96317	-2.096633756	4.83E-15	1.43E-13
ENSG00000143353	LYPLAL1	1066.143536	550.599658	0.952886515	4.84E-15	1.43E-13
ENSG00000019186	CYP24A1	174.2595191	0.555118095	8.178079749	5.77E-15	1.70E-13
ENSG00000095397	WHRN	278.5529747	663.6203929	-1.251137713	5.83E-15	1.72E-13
ENSG00000128342	LIF	2718.728966	1075.877259	1.336581721	6.04E-15	1.78E-13
ENSG00000179148	ALOXE3	446.4789713	191.3938614	1.219574059	6.11E-15	1.80E-13
ENSG00000100600	LGMN	3130.910869	1696.177802	0.883985057	6.40E-15	1.88E-13
ENSG00000176834	VSIG10	1343.43774	2761.138347	-1.038957047	7.22E-15	2.12E-13
ENSG00000111961	SASH1	703.6603113	1332.187026	-0.920874685	7.46E-15	2.18E-13
ENSG00000088970	KIZ	1308.083175	646.5589677	1.016537979	7.67E-15	2.24E-13
ENSG00000145087	STXBP5L	96.51865267	15.51075829	2.629138293	7.82E-15	2.28E-13
ENSG00000163793	DNAJC5G	196.3332226	28.14859419	2.818097609	8.38E-15	2.45E-13
ENSG00000056998	GYG2	0	137.3216276	-9.417774331	8.50E-15	2.48E-13
ENSG00000154655	L3MBTL4	66.54013381	3.937372195	4.084830461	8.51E-15	2.48E-13
ENSG00000241322	CDRT1	107.9121836	17.44972355	2.614086537	8.55E-15	2.49E-13
ENSG00000128309	MPST	1887.339132	3281.062767	-0.798027413	8.83E-15	2.56E-13
ENSG00000132254	ARFIP2	1407.013797	2405.5616	-0.773830667	9.03E-15	2.62E-13
ENSG00000163629	PTPN13	1186.128491	2172.044733	-0.872456808	9.17E-15	2.66E-13
ENSG00000033030	ZCCHC8	1422.013867	2412.227317	-0.762352658	9.36E-15	2.71E-13
ENSG00000168918	INPP5D	5.41944614	85.99155008	-3.984244339	9.39E-15	2.72E-13
ENSG00000197093	GAL3ST4	54.89989723	177.3440081	-1.692826949	9.66E-15	2.79E-13
ENSG00000197958	RPL12	17799.60588	29774.40547	-0.742225505	9.90E-15	2.86E-13
ENSG00000111261	MANSC1	362.9503397	711.7073353	-0.971134279	1.05E-14	3.02E-13
ENSG00000187720	THSD4	459.863804	191.1440684	1.268273232	1.09E-14	3.13E-13
ENSG00000168874	ATOH8	53.3119575	247.1331826	-2.209886374	1.09E-14	3.15E-13
ENSG00000095539	SEMA4G	134.0650552	376.4450783	-1.48837839	1.11E-14	3.18E-13
ENSG00000073910	FRY	111.7907822	21.88473983	2.349722739	1.13E-14	3.25E-13
ENSG00000101104	PABPC1L	1363.795838	3650.897587	-1.420556093	1.14E-14	3.28E-13
ENSG00000164970	FAM219A	814.7712195	384.3892447	1.082346692	1.18E-14	3.38E-13
ENSG00000169169	CPT1C	39.57592081	191.3070312	-2.271076264	1.19E-14	3.39E-13
ENSG00000250899	AC125807.2	188.1544156	440.3436605	-1.227442893	1.20E-14	3.43E-13
ENSG00000179104	TMTC2	796.1094649	374.8157761	1.087169319	1.20E-14	3.43E-13
ENSG00000158470	B4GALT5	6055.156476	3417.444366	0.825012513	1.23E-14	3.49E-13
ENSG00000129219	PLD2	687.2897146	1391.828173	-1.017180523	1.23E-14	3.49E-13
ENSG00000111276	CDKN1B	2402.197926	4101.247688	-0.771521945	1.25E-14	3.56E-13
ENSG00000177459	ERICH5	185.0836737	448.6580794	-1.278157833	1.27E-14	3.61E-13

ENSG00000105612	DNASE2	1447.575604	2700.499083	-0.899713771	1.28E-14	3.64E-13
ENSG00000198626	RYR2	72.31759915	1.541050765	5.580523202	1.29E-14	3.65E-13
ENSG00000145050	MANF	1899.370219	4181.609765	-1.138490989	1.31E-14	3.71E-13
ENSG00000204866	IGFL2	1109.117489	532.1206783	1.058344501	1.34E-14	3.79E-13
ENSG00000154065	ANKRD29	833.2298311	390.9946201	1.091893034	1.43E-14	4.03E-13
ENSG00000070047	PHRF1	2009.438286	3476.122645	-0.790871455	1.45E-14	4.09E-13
ENSG00000151332	MBIP	1142.077331	639.9323671	0.835235918	1.46E-14	4.12E-13
ENSG00000065911	MTHFD2	7285.048184	12773.48111	-0.810165713	1.47E-14	4.13E-13
ENSG00000111911	HINT3	875.7258958	1547.504359	-0.821614153	1.52E-14	4.26E-13
ENSG00000132329	RAMP1	525.4583087	1239.789555	-1.237728983	1.52E-14	4.26E-13
ENSG00000165650	PDZD8	2650.922727	4357.760964	-0.71720473	1.52E-14	4.27E-13
ENSG00000271857	AL096865.1	103.7473927	16.33948736	2.655582916	1.54E-14	4.32E-13
ENSG00000052344	PRSS8	2552.86187	4583.750941	-0.844270971	1.55E-14	4.33E-13
ENSG00000026297	RNASET2	435.3985905	868.9486514	-0.996292975	1.62E-14	4.52E-13
ENSG00000160789	LMNA	30921.52043	14838.90536	1.059164085	1.68E-14	4.70E-13
ENSG00000128923	MINDY2	1110.221981	1936.212214	-0.802468948	1.73E-14	4.81E-13
ENSG00000135525	MAP7	1328.402669	2433.695746	-0.873659176	1.82E-14	5.05E-13
ENSG00000137834	SMAD6	395.4598846	877.6192649	-1.14877316	1.83E-14	5.10E-13
ENSG00000075461	CACNG4	193.0742491	561.1782916	-1.537601497	1.87E-14	5.21E-13
ENSG00000129173	E2F8	363.1008117	896.3153737	-1.304395147	1.89E-14	5.25E-13
ENSG00000131471	AOC3	112.036546	23.56384044	2.240950595	1.97E-14	5.45E-13
ENSG00000112406	HECA	364.5021262	768.9503776	-1.077260581	2.21E-14	6.11E-13
ENSG00000132669	RIN2	645.0796907	305.4605701	1.08017766	2.34E-14	6.46E-13
ENSG00000095637	SORBS1	998.8943303	546.9334609	0.869562093	2.36E-14	6.50E-13
ENSG00000240065	PSMB9	54.09775951	228.7889041	-2.077079518	2.40E-14	6.63E-13
ENSG00000150687	PRSS23	7027.546251	3010.607672	1.22271125	2.51E-14	6.90E-13
ENSG00000285904	AC006452.1	27.37043178	130.0020796	-2.249224363	2.54E-14	6.98E-13
ENSG00000197461	PDGFA	496.3326558	183.4996501	1.436638906	2.61E-14	7.18E-13
ENSG00000170242	USP47	2187.449074	3739.939743	-0.773952292	2.74E-14	7.52E-13
ENSG00000147894	C9orf72	444.0701838	836.7529493	-0.914561272	2.75E-14	7.54E-13
ENSG00000171700	RGS19	649.5915618	262.2068459	1.305857206	2.77E-14	7.60E-13
ENSG00000119922	IFIT2	37.28431713	163.7529703	-2.131560445	2.78E-14	7.61E-13
ENSG00000242612	DECR2	389.6487976	749.3464758	-0.943572379	2.85E-14	7.79E-13
ENSG00000262580	AC087741.1	85.89603474	324.5158676	-1.915240088	2.88E-14	7.87E-13
ENSG00000162433	AK4	3426.775254	2079.369968	0.720800726	2.96E-14	8.07E-13
ENSG00000151632	AKR1C2	69.62088406	1.432982281	5.53727672	3.12E-14	8.50E-13
ENSG00000188176	SMTNL2	21.3467261	136.2065579	-2.668361655	3.21E-14	8.73E-13
ENSG00000078804	TP53INP2	1098.274779	608.768787	0.850622269	3.32E-14	9.02E-13
ENSG00000135960	EDAR	1397.44827	502.2432478	1.477185885	3.45E-14	9.37E-13
ENSG00000167323	STIM1	1460.275337	2655.461737	-0.862623046	3.52E-14	9.56E-13
ENSG00000196668	LINC00173	1074.720319	509.3098122	1.078876954	3.54E-14	9.60E-13
ENSG00000175906	ARL4D	310.3862845	101.1668453	1.611493915	3.55E-14	9.61E-13
ENSG00000138600	SPPL2A	1950.716997	3564.52726	-0.869492988	3.61E-14	9.77E-13

ENSG00000171723	GPHN	281.5095633	571.8522875	-1.023385783	3.69E-14	9.96E-13
ENSG00000104413	ESRP1	862.1710652	1678.778308	-0.961082552	3.73E-14	1.01E-12
ENSG00000095383	TBC1D2	1942.315605	843.3222994	1.202793199	3.93E-14	1.06E-12
ENSG00000150201	FXYD4	5.035108243	102.0565922	-4.330324275	4.25E-14	1.14E-12
ENSG00000148985	PGAP2	706.6435521	1396.515873	-0.982144046	4.63E-14	1.24E-12
ENSG00000151012	SLC7A11	9951.484926	5511.529257	0.852368127	4.77E-14	1.28E-12
ENSG00000163735	CXCL5	165.1851946	37.89709028	2.122116828	4.94E-14	1.33E-12
ENSG00000007237	GAS7	460.0616821	182.4813129	1.337259464	5.51E-14	1.48E-12
ENSG00000119711	ALDH6A1	1213.328927	2286.921083	-0.914270149	5.90E-14	1.58E-12
ENSG00000172819	RARG	1482.371552	2545.973162	-0.780050545	6.11E-14	1.63E-12
ENSG00000183722	LHFPL6	132.9431261	27.83967272	2.265293411	6.16E-14	1.65E-12
ENSG00000154188	ANGPT1	95.1398582	0	9.154867778	6.20E-14	1.65E-12
ENSG00000112624	BICRAL	645.9390691	1134.455678	-0.812184589	6.30E-14	1.68E-12
ENSG00000167914	GSDMA	324.4915933	98.22152936	1.725993215	7.04E-14	1.88E-12
ENSG00000185238	PRMT3	621.0487641	1197.013619	-0.94732306	7.18E-14	1.91E-12
ENSG00000106617	PRKAG2	1080.834742	568.2932461	0.926528649	7.26E-14	1.93E-12
ENSG00000073350	LLGL2	6167.657885	10573.96815	-0.777619375	7.28E-14	1.93E-12
ENSG00000075213	SEMA3A	386.9768173	930.1948294	-1.265190034	7.54E-14	2.00E-12
ENSG00000165556	CDX2	3536.60179	8987.036402	-1.345355601	7.93E-14	2.10E-12
ENSG00000123066	MED13L	15802.32288	9833.102811	0.684376985	7.96E-14	2.11E-12
ENSG00000162738	VANGL2	188.5384508	48.48652411	1.949629814	8.61E-14	2.28E-12
ENSG00000283498	MIR1244-2	132.9989342	384.5990486	-1.533873259	8.65E-14	2.28E-12
ENSG00000152409	JMY	1741.415945	2956.056878	-0.763242255	8.74E-14	2.31E-12
ENSG00000279192	PWAR5	88.52996396	0.909304906	6.611694018	9.02E-14	2.38E-12
ENSG00000131773	KHDRBS3	851.7314476	374.3039828	1.184281757	9.24E-14	2.43E-12
ENSG00000214026	MRPL23	453.8591253	895.330026	-0.980762844	9.55E-14	2.51E-12
ENSG00000205362	MT1A	0	112.2273816	-9.131499549	9.60E-14	2.52E-12
ENSG00000128000	ZNF780B	2432.180482	820.7654213	1.568238642	9.77E-14	2.56E-12
ENSG00000138326	RPS24	13215.99987	20368.59082	-0.624067688	9.78E-14	2.56E-12
ENSG00000179218	CALR	19579.94514	32813.14109	-0.744895645	9.80E-14	2.56E-12
ENSG00000173821	RNF213	10738.13751	17240.78664	-0.683082067	9.86E-14	2.58E-12
ENSG00000159618	ADGRG5	19.61402504	116.416462	-2.566578507	1.02E-13	2.67E-12
ENSG00000278730	AC005332.6	953.2262184	478.964824	0.993414012	1.04E-13	2.72E-12
ENSG00000243742	RPLP0P2	407.1610121	139.0228262	1.550616418	1.09E-13	2.83E-12
ENSG00000136542	GALNT5	94.54241379	258.1552418	-1.448861498	1.10E-13	2.87E-12
ENSG00000139044	B4GALNT3	692.1198155	300.3185485	1.207125896	1.13E-13	2.93E-12
ENSG00000152766	ANKRD22	127.0859	29.33605748	2.103883597	1.14E-13	2.96E-12
ENSG00000196586	MYO6	3833.32516	6391.028549	-0.737285499	1.16E-13	3.02E-12
ENSG00000142657	PGD	11998.42958	6496.288716	0.885018464	1.19E-13	3.09E-12
ENSG00000164796	CSMD3	88.51570285	0.886711385	6.614846449	1.20E-13	3.11E-12
ENSG00000128228	SDF2L1	851.6691383	2101.232902	-1.302905074	1.24E-13	3.21E-12
ENSG00000155090	KLF10	2553.12296	1240.216945	1.041285781	1.47E-13	3.81E-12
ENSG00000084628	NKAIN1	146.0287558	40.14995444	1.858263983	1.51E-13	3.91E-12

ENSG00000119042	SATB2	1332.16707	2222.296811	-0.738218001	1.53E-13	3.94E-12
ENSG00000105655	ISYNA1	1509.706843	803.2460801	0.91111803	1.54E-13	3.97E-12
ENSG00000141574	SECTM1	950.886404	1662.328432	-0.805809376	1.58E-13	4.07E-12
ENSG00000196092	PAX5	0	97.69602819	-8.92863837	1.69E-13	4.35E-12
ENSG00000121274	TENT4B	1105.346394	1915.660454	-0.793344329	1.73E-13	4.45E-12
ENSG00000100439	ABHD4	2067.466488	1043.33189	0.987261603	1.75E-13	4.49E-12
ENSG00000250786	SNHG18	0	105.9046852	-9.042207272	1.77E-13	4.54E-12
ENSG00000167011	NAT16	117.4390285	25.42368922	2.20171871	1.77E-13	4.54E-12
ENSG00000185037	ZNF733P	85.45571489	0	9.000504721	1.79E-13	4.59E-12
ENSG00000181690	PLAG1	452.1877888	126.2271618	1.845995641	1.81E-13	4.63E-12
ENSG00000166546	BEAN1	211.8382649	74.77424016	1.50697629	1.82E-13	4.64E-12
ENSG00000127954	STEAP4	85.7812394	233.2507481	-1.442487483	1.84E-13	4.68E-12
ENSG00000111731	C2CD5	1958.246224	3385.588886	-0.789660562	1.84E-13	4.68E-12
ENSG00000254166	CASC19	255.4979571	612.9999408	-1.261543508	1.88E-13	4.79E-12
ENSG00000054598	FOXC1	1111.318404	615.4754695	0.851667136	1.92E-13	4.87E-12
ENSG00000221890	NPTXR	574.5839001	278.6031296	1.042252472	2.11E-13	5.36E-12
ENSG00000136235	GPNMB	61.14256856	5.733388486	3.414978394	2.14E-13	5.43E-12
ENSG00000114268	PFKFB4	1251.69264	534.9421083	1.227050262	2.16E-13	5.48E-12
ENSG00000103647	CORO2B	123.001966	30.31065424	2.017747016	2.17E-13	5.49E-12
ENSG00000236473	KRT43P	66.20224898	2.163949425	4.96190587	2.17E-13	5.50E-12
ENSG00000148677	ANKRD1	929.1035267	55.4899194	4.062323393	2.20E-13	5.55E-12
ENSG00000170522	ELOVL6	1378.815899	2717.796634	-0.979312975	2.21E-13	5.59E-12
ENSG00000074319	TSG101	1195.835132	2050.817257	-0.778413009	2.23E-13	5.61E-12
ENSG00000011422	PLAUR	4034.562798	1972.727554	1.03177448	2.24E-13	5.63E-12
ENSG00000160161	CILP2	96.16952321	294.1165205	-1.611968484	2.26E-13	5.69E-12
ENSG00000189143	CLDN4	9552.141737	16345.34871	-0.774969902	2.36E-13	5.94E-12
ENSG00000183337	BCOR	2141.405691	3722.944313	-0.797695826	2.48E-13	6.23E-12
ENSG00000110013	SIAE	1173.758412	1978.559811	-0.753591071	2.55E-13	6.39E-12
ENSG00000035403	VCL	16121.31935	9068.703128	0.829909101	2.60E-13	6.51E-12
ENSG00000110497	AMBRA1	2557.007875	1583.54465	0.691124409	2.61E-13	6.53E-12
ENSG00000135334	AKIRIN2	2591.522378	1377.07065	0.911577533	2.78E-13	6.94E-12
ENSG00000180739	S1PR5	2.167778456	70.21748737	-5.011970693	2.92E-13	7.30E-12
ENSG00000163683	SMIM14	1661.299414	2929.729198	-0.818528524	3.02E-13	7.54E-12
ENSG00000146425	DYNLT1	678.0761852	1299.943125	-0.93930115	3.07E-13	7.64E-12
ENSG00000267605	AC016590.1	85.47871438	0.994779868	6.550700425	3.43E-13	8.53E-12
ENSG00000172348	RCAN2	73.16080444	3.83961027	4.310664567	3.45E-13	8.58E-12
ENSG00000100558	PLEK2	213.1736369	526.3913035	-1.303900598	3.49E-13	8.67E-12
ENSG00000258405	ZNF578	62.76205819	1.626525727	5.365843201	3.54E-13	8.78E-12
ENSG00000188215	DCUN1D3	960.2329876	444.0287252	1.111142706	3.82E-13	9.47E-12
ENSG00000198087	CD2AP	3210.011291	5503.633805	-0.777913929	3.87E-13	9.58E-12
ENSG00000175832	ETV4	2414.725009	1105.703546	1.126148183	3.93E-13	9.71E-12
ENSG00000197301	AC090673.1	144.6138081	31.16489556	2.220535967	3.93E-13	9.71E-12
ENSG00000137942	FNBP1L	3167.44639	4977.828042	-0.65209071	4.00E-13	9.87E-12

ENSG00000047617	ANO2	60.85225248	1.572491485	5.328567543	4.01E-13	9.88E-12
ENSG00000172059	KLF11	1111.154451	579.3783964	0.939571095	4.11E-13	1.01E-11
ENSG00000153237	CCDC148	174.7075988	46.44035039	1.921811364	4.12E-13	1.01E-11
ENSG00000188157	AGRN	30842.72457	17711.91637	0.800217167	4.17E-13	1.03E-11
ENSG00000108854	SMURF2	2816.946216	1633.437329	0.786111001	4.20E-13	1.03E-11
ENSG00000124766	SOX4	3910.095517	2102.882757	0.89521951	4.33E-13	1.06E-11
ENSG00000101187	SLCO4A1	2274.644953	1229.784063	0.886685872	4.35E-13	1.07E-11
ENSG00000181274	FRAT2	189.945476	408.4637069	-1.104040799	4.53E-13	1.11E-11
ENSG00000180354	MTURN	661.7967115	1621.819549	-1.292598597	4.67E-13	1.14E-11
ENSG00000126603	GLIS2	924.4544468	1649.892246	-0.835205403	4.74E-13	1.16E-11
ENSG00000117425	PTCH2	19.22318596	96.13479431	-2.320495585	4.95E-13	1.21E-11
ENSG00000273143	AL355512.1	83.40637907	13.87684472	2.586959085	5.10E-13	1.24E-11
ENSG00000148730	EIF4EBP2	7270.521244	4367.317354	0.735209748	5.13E-13	1.25E-11
ENSG00000111640	GAPDH	121554.0808	71620.28092	0.763149145	5.31E-13	1.29E-11
ENSG00000131389	SLC6A6	8201.561272	4575.281826	0.842233363	5.32E-13	1.29E-11
ENSG00000186081	KRT5	70.135397	7.021962438	3.293572287	5.64E-13	1.37E-11
ENSG00000146648	EGFR	5847.195699	3413.918318	0.776154484	5.66E-13	1.37E-11
ENSG00000110651	CD81	4331.874898	7889.015275	-0.864817155	5.86E-13	1.42E-11
ENSG00000251493	FOXD1	1519.088613	815.182759	0.898036941	6.32E-13	1.53E-11
ENSG00000120925	RNF170	733.4471392	1245.360048	-0.76411879	6.41E-13	1.55E-11
ENSG00000173801	JUP	9032.24462	14026.27124	-0.634932494	6.42E-13	1.55E-11
ENSG00000164764	SBSPON	310.2106124	1123.333148	-1.855566711	6.57E-13	1.59E-11
ENSG00000174775	HRAS	575.4661013	1159.154576	-1.010964393	6.84E-13	1.65E-11
ENSG00000176222	ZNF404	59.11669755	4.013999958	3.903706886	7.40E-13	1.78E-11
ENSG00000243927	MRPS6	1135.205027	578.664863	0.970954032	7.44E-13	1.79E-11
ENSG00000176046	NUPR1	26.87938365	192.917608	-2.84201701	7.52E-13	1.81E-11
ENSG00000161647	MPP3	1078.776365	535.2858972	1.01023343	7.98E-13	1.92E-11
ENSG00000005801	ZNF195	661.9827313	1168.394601	-0.820276834	8.17E-13	1.96E-11
ENSG00000260231	KDM7A-DT	1129.263448	464.1489282	1.284520619	9.24E-13	2.22E-11
ENSG00000161638	ITGA5	1633.358193	819.7232653	0.993852912	9.39E-13	2.25E-11
ENSG00000177494	ZBED2	59.35008689	1.965506855	4.832538435	1.01E-12	2.43E-11
ENSG00000154310	TNIK	432.2642468	175.7765068	1.294786995	1.03E-12	2.47E-11
ENSG00000185432	METTL7A	294.7691381	648.9953835	-1.139152597	1.07E-12	2.56E-11
ENSG00000159335	PTMS	4844.560952	2580.494186	0.908382722	1.07E-12	2.56E-11
ENSG00000118263	KLF7	458.3914306	224.141034	1.032682452	1.08E-12	2.58E-11
ENSG00000196405	EVL	8.024841926	70.96848103	-3.146549428	1.11E-12	2.65E-11
ENSG00000229953	AL590666.2	629.0576097	325.2857285	0.950024302	1.14E-12	2.70E-11
ENSG00000250303	AP002884.1	103.49069	24.87397856	2.061138703	1.19E-12	2.83E-11
ENSG00000122026	RPL21	10729.33557	16449.49887	-0.616468409	1.22E-12	2.90E-11
ENSG00000172164	SNTB1	5230.420581	2821.806182	0.890420202	1.23E-12	2.93E-11
ENSG00000130294	KIF1A	200.6841592	58.59153913	1.767779526	1.27E-12	3.00E-11
ENSG00000138771	SHROOM3	4427.585563	9388.61136	-1.084251943	1.31E-12	3.09E-11
ENSG00000175538	KCNE3	0.349140684	143.9585619	-8.525963375	1.31E-12	3.09E-11

ENSG00000105767	CADM4	773.7020004	402.0880053	0.942701148	1.49E-12	3.53E-11
ENSG00000149218	ENDOD1	1218.201573	2159.023277	-0.825844967	1.51E-12	3.57E-11
ENSG00000141756	FKBP10	853.4825195	1635.295156	-0.937578563	1.55E-12	3.65E-11
ENSG00000143434	SEMA6C	562.8734205	292.642416	0.944765103	1.58E-12	3.73E-11
ENSG00000187867	PALM3	25.94675694	115.7200236	-2.151588883	1.59E-12	3.75E-11
ENSG00000260719	AC009133.3	0	83.65743244	-8.702322969	1.59E-12	3.75E-11
ENSG00000250479	CHCHD10	1212.430735	2746.096346	-1.179669264	1.61E-12	3.79E-11
ENSG00000136169	SETDB2	1892.607016	3738.423921	-0.981841532	1.65E-12	3.88E-11
ENSG00000212724	KRTAP2-3	68.80145754	0	8.687514077	1.68E-12	3.94E-11
ENSG00000185905	C16orf54	0	82.18321119	-8.676399443	1.74E-12	4.08E-11
ENSG00000175130	MARCKSL1	4371.022703	2457.702075	0.830287979	1.79E-12	4.20E-11
ENSG00000078081	LAMP3	214.863664	658.1062552	-1.615465068	1.80E-12	4.22E-11
ENSG00000196177	ACADSB	821.5377618	1404.408483	-0.773761166	2.02E-12	4.72E-11
ENSG00000166068	SPRED1	1761.089426	1053.657513	0.74051215	2.03E-12	4.73E-11
ENSG00000150093	ITGB1	7101.200234	11253.82317	-0.664285251	2.03E-12	4.74E-11
ENSG00000100784	RPS6KA5	273.1430379	620.1487334	-1.184598337	2.08E-12	4.85E-11
ENSG00000156587	UBE2L6	604.323773	1167.612296	-0.949565853	2.10E-12	4.89E-11
ENSG00000269825	AC022150.4	465.5240854	239.9553846	0.956061371	2.15E-12	5.01E-11
ENSG00000197444	OGDHL	0	77.62690115	-8.59665325	2.16E-12	5.01E-11
ENSG00000104219	ZDHHC2	3462.660432	2104.701904	0.717996283	2.21E-12	5.12E-11
ENSG00000171606	ZNF274	1135.625201	670.1009451	0.76087682	2.21E-12	5.13E-11
ENSG00000262454	MIR193BHG	322.8998467	130.5406237	1.302543239	2.38E-12	5.52E-11
ENSG00000151014	NOCT	544.5167674	170.3989994	1.672177359	2.38E-12	5.52E-11
ENSG00000184106	TREML3P	53.92794945	1.841203334	4.892696539	2.48E-12	5.75E-11
ENSG00000152782	PANK1	538.5234789	997.3567155	-0.888177049	2.53E-12	5.85E-11
ENSG00000167676	PLIN4	169.524082	503.9759144	-1.57015523	2.55E-12	5.90E-11
ENSG00000157349	DDX19B	238.9158407	485.911853	-1.023828523	2.64E-12	6.09E-11
ENSG00000196507	TCEAL3	177.574766	415.3538739	-1.227034375	2.68E-12	6.18E-11
ENSG00000165474	GJB2	2452.056715	1296.113053	0.919095357	2.73E-12	6.30E-11
ENSG00000106683	LIMK1	3140.356415	1473.166329	1.091414403	2.74E-12	6.30E-11
ENSG00000259291	ZNF710-AS1	116.7060265	298.6401383	-1.353212948	2.78E-12	6.40E-11
ENSG00000163251	FZD5	430.158475	871.0622282	-1.018680925	2.80E-12	6.43E-11
ENSG00000102057	KCND1	184.6742759	58.9451266	1.650534975	2.85E-12	6.53E-11
ENSG00000010818	HIVEP2	621.4039153	1277.535698	-1.039017632	2.87E-12	6.57E-11
ENSG00000161905	ALOX15	5.048110611	63.76577041	-3.649075915	2.87E-12	6.57E-11
ENSG00000159263	SIM2	596.098387	1044.877111	-0.810092398	2.90E-12	6.63E-11
ENSG00000169814	BTD	165.1323917	349.2150007	-1.080325375	2.91E-12	6.64E-11
ENSG00000151136	BTBD11	269.4023825	106.6442389	1.333364103	2.91E-12	6.66E-11
ENSG00000214049	UCA1	22.48135483	119.0743678	-2.407140278	2.94E-12	6.70E-11
ENSG00000167703	SLC43A2	228.9548744	645.224439	-1.495767633	3.23E-12	7.36E-11
ENSG00000134242	PTPN22	124.1091687	27.81459049	2.172293099	3.29E-12	7.49E-11
ENSG00000196937	FAM3C	4464.188989	2735.936397	0.706213122	3.35E-12	7.62E-11
ENSG00000105329	TGFB1	3385.106367	1806.48214	0.905563077	3.46E-12	7.86E-11

ENSG00000145730	PAM	6491.568025	3519.248225	0.883606726	3.47E-12	7.88E-11
ENSG00000136205	TNS3	3732.479815	7256.075918	-0.958885464	3.49E-12	7.92E-11
ENSG00000214021	TTLL3	2357.427574	1046.781991	1.172060849	3.50E-12	7.94E-11
ENSG00000166250	CLMP	185.07574	66.8135125	1.470928171	3.55E-12	8.05E-11
ENSG00000182534	MXRA7	2647.144963	1368.523533	0.951163159	3.61E-12	8.17E-11
ENSG00000198467	TPM2	412.298715	1014.151734	-1.297393352	3.82E-12	8.63E-11
ENSG00000041353	RAB27B	591.1075254	275.4409605	1.100645974	3.83E-12	8.66E-11
ENSG00000052802	MSMO1	6313.468576	4012.000496	0.653990557	3.84E-12	8.67E-11
ENSG00000158301	GPRASP2	18.01689284	100.2157021	-2.470262028	3.86E-12	8.71E-11
ENSG00000100461	RBM23	3795.31051	2447.259004	0.633135757	4.10E-12	9.24E-11
ENSG00000142937	RPS8	25216.62576	39744.93225	-0.656388942	4.31E-12	9.71E-11
ENSG00000138166	DUSP5	2385.277644	237.0571719	3.32984784	4.53E-12	1.02E-10
ENSG00000106077	ABHD11	3170.025748	5574.675062	-0.814269246	4.56E-12	1.02E-10
ENSG00000146192	FGD2	5.083307823	62.43260876	-3.620225724	4.58E-12	1.03E-10
ENSG00000124249	KCNK15	0	73.11524436	-8.50784079	4.68E-12	1.05E-10
ENSG00000002745	WNT16	520.320617	196.1714683	1.411116831	4.71E-12	1.06E-10
ENSG00000130402	ACTN4	38273.73307	23514.10934	0.702794118	4.80E-12	1.07E-10
ENSG00000168062	BATF2	24.63613092	123.3873089	-2.319802059	4.82E-12	1.08E-10
ENSG00000125772	GPCPD1	598.5444323	1073.016807	-0.842180627	4.94E-12	1.10E-10
ENSG00000103355	PRSS33	55.48481581	2.163949425	4.708513767	5.02E-12	1.12E-10
ENSG00000172046	USP19	2004.771959	3196.95598	-0.673233553	5.10E-12	1.14E-10
ENSG00000206075	SERPINB5	33.01265346	123.1634324	-1.897133298	5.29E-12	1.18E-10
ENSG00000133706	LARS	3682.401118	5734.02041	-0.63891846	5.30E-12	1.18E-10
ENSG00000163406	SLC15A2	23.95085192	103.2672356	-2.106577082	5.42E-12	1.21E-10
ENSG00000124198	ARFGEF2	1819.865544	2849.906662	-0.647136955	5.44E-12	1.21E-10
ENSG00000167566	NCKAP5L	1668.715592	988.9203634	0.755325528	5.66E-12	1.26E-10
ENSG00000170439	METTL7B	877.1983982	496.1848992	0.821450214	5.82E-12	1.29E-10
ENSG00000112852	PCDHB2	1.806905368	59.05817251	-5.024407668	6.02E-12	1.33E-10
ENSG00000103966	EHD4	3517.506004	1698.73598	1.049588069	6.07E-12	1.34E-10
ENSG00000141013	GAS8	623.5996217	1055.600543	-0.759290929	6.40E-12	1.42E-10
ENSG00000198182	ZNF607	734.0109816	360.8755817	1.026242178	6.61E-12	1.46E-10
ENSG00000134398	ERN2	4.392797602	60.70231433	-3.794650868	6.72E-12	1.48E-10
ENSG00000254615	AC027031.2	135.6788483	40.06103971	1.759859536	6.74E-12	1.49E-10
ENSG00000132256	TRIM5	278.9434998	573.6583099	-1.039774675	6.77E-12	1.49E-10
ENSG00000112378	PERP	6067.127051	12200.49206	-1.007780784	6.80E-12	1.50E-10
ENSG00000198929	NOS1AP	645.347313	332.8445026	0.955789026	6.87E-12	1.51E-10
ENSG00000023191	RNH1	1796.017926	3003.902026	-0.742058621	6.89E-12	1.52E-10
ENSG00000137801	THBS1	4812.404543	2095.117833	1.200179048	7.27E-12	1.60E-10
ENSG00000141576	RNF157	68.27489824	205.6096746	-1.593454149	7.37E-12	1.62E-10
ENSG00000130940	CASZ1	1435.974395	749.0590001	0.938290736	7.56E-12	1.66E-10
ENSG00000135547	HEY2	62.99752214	5.855203293	3.446142902	7.62E-12	1.67E-10
ENSG00000184752	NDUFA12	1735.326117	903.8705495	0.940194195	7.65E-12	1.67E-10
ENSG00000165434	PGM2L1	497.7474297	251.2953694	0.986524442	8.13E-12	1.78E-10

ENSG00000185156	MFSD6L	80.85569527	238.3954408	-1.557147889	8.33E-12	1.82E-10
ENSG00000101624	CEP76	479.8039731	898.1752609	-0.905095437	8.44E-12	1.84E-10
ENSG00000103196	CRISPLD2	355.2251689	165.5781079	1.09940941	9.44E-12	2.06E-10
ENSG00000179119	SPTY2D1	838.4274577	1520.354312	-0.859101781	9.53E-12	2.08E-10
ENSG00000170390	DCLK2	185.4849752	67.09597068	1.468262366	1.02E-11	2.22E-10
ENSG00000269915	AP006621.4	19.92669855	95.65337253	-2.258756648	1.07E-11	2.32E-10
ENSG00000258657	AL136018.1	57.23827916	0	8.421684182	1.07E-11	2.33E-10
ENSG00000128573	FOXP2	1.102122816	82.20536197	-6.232844083	1.08E-11	2.35E-10
ENSG00000142512	SIGLEC10	152.3235992	48.10029724	1.668082278	1.11E-11	2.41E-10
ENSG00000131480	AOC2	155.118146	52.8664768	1.550785	1.16E-11	2.51E-10
ENSG00000115194	SLC30A3	115.2699801	31.57560532	1.872094362	1.16E-11	2.51E-10
ENSG00000119917	IFIT3	38.20013159	140.1603344	-1.876474695	1.23E-11	2.66E-10
ENSG00000092439	TRPM7	2609.536605	4581.062457	-0.811675381	1.25E-11	2.69E-10
ENSG00000146072	TNFRSF21	744.1392292	1793.917313	-1.269867104	1.28E-11	2.77E-10
ENSG00000188846	RPL14	14311.73087	21763.55621	-0.604725283	1.33E-11	2.86E-10
ENSG00000148925	BTBD10	480.0444916	894.1853648	-0.898168813	1.34E-11	2.88E-10
ENSG00000178209	PLEC	44967.49284	29223.54809	0.621740241	1.34E-11	2.90E-10
ENSG00000263002	ZNF234	1281.397279	514.0254231	1.319196663	1.41E-11	3.03E-10
ENSG00000044574	HSPA5	29587.58891	56351.80226	-0.929469772	1.41E-11	3.04E-10
ENSG00000011028	MRC2	439.1711068	946.433673	-1.106575849	1.45E-11	3.11E-10
ENSG00000127863	TNFRSF19	1141.280568	611.3201493	0.899274384	1.46E-11	3.13E-10
ENSG00000177628	GBA	934.1517692	1550.448256	-0.731260575	1.46E-11	3.14E-10
ENSG00000104447	TRPS1	938.7156327	482.4792921	0.960042581	1.46E-11	3.14E-10
ENSG00000184277	TM2D3	418.5447084	733.424565	-0.809096102	1.50E-11	3.22E-10
ENSG00000120457	KCNJ5	1685.828717	683.1200291	1.304060535	1.55E-11	3.31E-10
ENSG00000197808	ZNF461	213.6355125	49.10435431	2.131474103	1.57E-11	3.37E-10
ENSG00000246334	PRR7-AS1	212.611544	84.3266258	1.334697932	1.63E-11	3.48E-10
ENSG00000110092	CCND1	32648.90269	15623.85329	1.063227584	1.65E-11	3.51E-10
ENSG00000100450	GZMH	54.30578616	0	8.345833603	1.65E-11	3.52E-10
ENSG00000197990	ZNF734P	55.62307595	0	8.382469653	1.70E-11	3.61E-10
ENSG00000175806	MSRA	407.0987027	198.7046405	1.032549204	1.70E-11	3.62E-10
ENSG00000181652	ATG9B	98.82041606	17.50272844	2.508088867	1.75E-11	3.71E-10
ENSG00000205189	ZBTB10	1028.97386	1637.760875	-0.670377766	1.76E-11	3.74E-10
ENSG00000167315	ACAA2	1428.855891	2296.055516	-0.684282692	1.78E-11	3.78E-10
ENSG00000204542	C6orf15	67.905298	9.464930272	2.853514293	1.81E-11	3.84E-10
ENSG00000186487	MYT1L	1.470767051	61.30020906	-5.395957728	1.81E-11	3.84E-10
ENSG00000170412	GPRC5C	528.3632018	988.1520779	-0.90319267	1.82E-11	3.85E-10
ENSG00000229117	RPL41	10779.67719	16559.04291	-0.619302455	1.87E-11	3.95E-10
ENSG00000128713	HOXD11	184.0706442	61.16120816	1.594607601	2.12E-11	4.49E-10
ENSG00000196371	FUT4	301.1288308	579.5445382	-0.945286056	2.17E-11	4.59E-10
ENSG00000177181	RIMKLA	546.3615359	251.1229346	1.123864483	2.22E-11	4.67E-10
ENSG00000178531	CTXN1	857.2778868	445.2776618	0.943521079	2.23E-11	4.70E-10
ENSG00000184384	MAML2	310.467304	112.6139347	1.46390117	2.25E-11	4.74E-10

ENSG00000170373	CST1	56.3405357	2.289712306	4.7079852	2.31E-11	4.87E-10
ENSG00000256043	CTSO	492.0308748	148.2061455	1.735301791	2.37E-11	4.98E-10
ENSG00000163050	COQ8A	1160.15461	1896.22711	-0.708517979	2.38E-11	5.01E-10
ENSG00000121691	CAT	1232.681371	2410.328303	-0.966871234	2.40E-11	5.03E-10
ENSG00000162591	MEGF6	498.0733018	1463.947946	-1.554776623	2.40E-11	5.04E-10
ENSG00000102144	PGK1	20711.26359	11122.57431	0.896861045	2.42E-11	5.07E-10
ENSG00000137941	TTLL7	706.5026036	345.2001664	1.035048136	2.45E-11	5.14E-10
ENSG00000130147	SH3BP4	15524.03909	10098.95181	0.620273068	2.46E-11	5.14E-10
ENSG00000067064	IDI1	6208.898359	3614.239857	0.780429406	2.50E-11	5.23E-10
ENSG00000026025	VIM	73.62030266	11.81701571	2.642923095	2.52E-11	5.27E-10
ENSG00000140057	AK7	60.98765874	168.48238	-1.466306487	2.56E-11	5.34E-10
ENSG00000165568	AKR1E2	102.2816943	24.17840021	2.082023207	2.58E-11	5.38E-10
ENSG00000196859	KRT39	89.85090106	0.331593289	8.110942588	2.68E-11	5.57E-10
ENSG00000145990	GFOD1	321.3862738	667.1239676	-1.055032765	2.73E-11	5.67E-10
ENSG00000130589	HELZ2	1574.199037	2815.363864	-0.838689583	2.76E-11	5.74E-10
ENSG00000170775	GPR37	3.994187375	55.46767759	-3.796665114	2.80E-11	5.81E-10
ENSG00000169991	IFFO2	1155.388938	584.840923	0.981158893	2.83E-11	5.88E-10
ENSG00000171940	ZNF217	2289.78846	3654.758813	-0.674519002	2.85E-11	5.91E-10
ENSG00000182208	MOB2	407.2545656	860.7006832	-1.078591609	2.85E-11	5.91E-10
ENSG00000155304	HSPA13	2115.901221	3574.060265	-0.756358688	2.86E-11	5.92E-10
ENSG00000160211	G6PD	15278.98302	2908.78234	2.392941393	2.90E-11	5.99E-10
ENSG00000007171	NOS2	374.8358382	154.8388456	1.277266018	3.09E-11	6.38E-10
ENSG00000198380	GFPT1	5231.416364	10008.50398	-0.935924063	3.23E-11	6.67E-10
ENSG00000181523	SGSH	615.6063297	1205.503926	-0.968761981	3.30E-11	6.81E-10
ENSG00000100346	CACNA1I	139.8905799	44.85119386	1.644719823	3.61E-11	7.43E-10
ENSG00000132692	BCAN	649.7103296	354.8929083	0.871055016	3.62E-11	7.46E-10
ENSG00000205364	MT1M	0	57.11774789	-8.154937116	3.69E-11	7.60E-10
ENSG00000088280	ASAP3	577.6381662	985.1626842	-0.769957363	3.74E-11	7.70E-10
ENSG00000069535	MAOB	21.06005732	102.3701394	-2.279740855	3.87E-11	7.96E-10
ENSG00000196365	LONP1	5921.296548	9626.038575	-0.70107525	4.06E-11	8.33E-10
ENSG00000179715	PCED1B	47.83003945	3.592032582	3.728423656	4.09E-11	8.39E-10
ENSG00000160360	GPSM1	743.1418822	397.2124147	0.904426005	4.26E-11	8.73E-10
ENSG00000165898	ISCA2	756.1804422	436.0839066	0.793305056	4.31E-11	8.82E-10
ENSG00000112592	TBP	323.8730644	657.8932987	-1.023953151	4.34E-11	8.88E-10
ENSG00000100814	CCNB1IP1	1034.390917	1676.716199	-0.69662658	4.36E-11	8.92E-10
ENSG00000273674	AC021752.1	5.863564653	70.76308063	-3.597717773	4.39E-11	8.97E-10
ENSG00000109099	PMP22	877.504422	416.7952606	1.072883862	4.46E-11	9.11E-10
ENSG00000154274	C4orf19	65.40788241	10.66554055	2.625232745	4.67E-11	9.51E-10
ENSG00000147274	RBMX	7449.84328	11324.22526	-0.604111814	4.73E-11	9.64E-10
ENSG00000163071	SPATA18	8.328474324	216.2406208	-4.697005975	4.79E-11	9.76E-10
ENSG00000151233	GXYLT1	1173.887493	1907.043171	-0.700247727	4.85E-11	9.87E-10
ENSG00000268601	AC115522.1	81.96400532	14.84413195	2.46794495	5.00E-11	1.02E-09
ENSG00000183018	SPNS2	717.6625206	1429.97266	-0.994240586	5.02E-11	1.02E-09

ENSG00000173706	HEG1	1409.214278	655.0054329	1.105131641	5.10E-11	1.04E-09
ENSG00000163909	HEYL	2.885563375	54.58388493	-4.232610178	5.15E-11	1.04E-09
ENSG00000085276	MECOM	275.3628258	112.4326	1.292694759	5.16E-11	1.05E-09
ENSG00000133069	TMCC2	304.2674496	132.9182869	1.195121003	5.25E-11	1.06E-09
ENSG00000177788	AL162595.1	160.8293407	58.19654336	1.463830179	5.41E-11	1.09E-09
ENSG00000135404	CD63	11680.33665	7232.758323	0.691397123	5.43E-11	1.10E-09
ENSG00000132688	NES	52.56928644	196.1857099	-1.900793955	5.51E-11	1.11E-09
ENSG00000215817	ZC3H11B	65.64080643	5.148289031	3.725637975	5.59E-11	1.13E-09
ENSG00000009844	VTA1	844.8651646	1550.577746	-0.876500615	5.60E-11	1.13E-09
ENSG00000006747	SCIN	8.380483794	62.26844733	-2.894693991	5.65E-11	1.14E-09
ENSG00000018236	CNTN1	48.0488425	0	8.169548252	5.74E-11	1.15E-09
ENSG00000188051	TMEM221	408.287065	209.4633956	0.963200739	5.82E-11	1.17E-09
ENSG00000159403	C1R	62.41037758	191.5200416	-1.616191813	5.84E-11	1.17E-09
ENSG00000152484	USP12	1745.925162	2841.165559	-0.70240455	5.85E-11	1.17E-09
ENSG00000112297	CRYBG1	1258.795573	2087.480473	-0.72967236	5.91E-11	1.19E-09
ENSG00000078269	SYNJ2	926.491456	1654.970762	-0.837052447	5.93E-11	1.19E-09
ENSG00000142627	EPHA2	8666.914328	4291.850503	1.013717958	6.08E-11	1.22E-09
ENSG00000090861	AARS	7849.664857	12536.78453	-0.6754337	6.21E-11	1.24E-09
ENSG00000110436	SLC1A2	53.58039269	5.850304168	3.215932071	6.26E-11	1.25E-09
ENSG00000176406	RIMS2	75.01147158	9.179983386	3.065337054	6.28E-11	1.25E-09
ENSG00000166979	EVA1C	1360.984683	683.230509	0.993038783	6.34E-11	1.27E-09
ENSG00000218426	AL590867.2	54.25758658	156.2501198	-1.52757926	6.36E-11	1.27E-09
ENSG00000107282	APBA1	207.9870923	84.34577956	1.304930167	6.40E-11	1.28E-09
ENSG00000167779	IGFBP6	570.4619233	249.6166459	1.192409005	6.41E-11	1.28E-09
ENSG00000182197	EXT1	801.9576386	1323.846337	-0.72336905	6.47E-11	1.29E-09
ENSG00000078177	N4BP2	670.7133005	1366.237638	-1.026088937	7.23E-11	1.44E-09
ENSG00000104518	GSDMD	1657.569129	2551.972826	-0.622445982	7.24E-11	1.44E-09
ENSG00000266088	AC004585.1	104.466599	26.28977476	1.999690327	7.42E-11	1.47E-09
ENSG00000162755	KLHDC9	102.6466913	234.9981503	-1.194131192	7.44E-11	1.48E-09
ENSG00000181192	DHTKD1	1231.68373	1977.148329	-0.683188986	7.61E-11	1.51E-09
ENSG00000106123	EPHB6	121.7758554	33.33726217	1.861096812	7.85E-11	1.56E-09
ENSG00000165915	SLC39A13	2500.292015	1452.824254	0.783580809	7.97E-11	1.58E-09
ENSG00000114942	EEF1B2	6031.522205	9125.238895	-0.597387511	8.11E-11	1.60E-09
ENSG00000184545	DUSP8	439.3521756	750.0196045	-0.771599882	8.12E-11	1.60E-09
ENSG00000104432	IL7	5.122314926	60.81973832	-3.573519066	8.18E-11	1.62E-09
ENSG00000011021	CLCN6	1707.185093	1047.343886	0.704938383	8.34E-11	1.64E-09
ENSG00000173068	BNC2	53.02798002	4.021387796	3.673899131	8.35E-11	1.65E-09
ENSG00000204610	TRIM15	1294.078563	774.6771071	0.739574128	8.42E-11	1.66E-09
ENSG00000198189	HSD17B11	1165.498165	1890.544642	-0.69780004	8.76E-11	1.72E-09
ENSG00000110427	KIAA1549L	72.14303442	186.2631885	-1.368780375	8.85E-11	1.74E-09
ENSG00000247271	ZBED5-AS1	73.7076607	200.9995909	-1.445356385	8.95E-11	1.76E-09
ENSG00000136689	IL1RN	30.40202645	110.5107425	-1.857750916	9.04E-11	1.77E-09
ENSG00000179477	ALOX12B	113.5387004	30.80485857	1.888530608	9.17E-11	1.80E-09

ENSG00000116663	FBXO6	75.72544661	193.725751	-1.356317757	9.35E-11	1.83E-09
ENSG00000114854	TNNC1	4372.024163	2512.476379	0.798973245	9.70E-11	1.90E-09
ENSG00000213079	SCAF8	1071.447716	1942.297874	-0.858388344	9.86E-11	1.93E-09
ENSG00000064042	LIMCH1	1028.48852	612.9017359	0.747021837	9.88E-11	1.93E-09
ENSG00000198818	SFT2D1	933.7139864	1599.791783	-0.777127744	9.92E-11	1.94E-09
ENSG00000110328	GALNT18	30.61559828	125.1693965	-2.036189567	1.02E-10	1.99E-09
ENSG00000126217	MCF2L	633.9874178	1313.350015	-1.050079797	1.04E-10	2.03E-09
ENSG00000151276	MAGI1	1536.28255	2451.11459	-0.673971546	1.04E-10	2.03E-09
ENSG00000153936	HS2ST1	1613.439453	2478.246616	-0.619136331	1.04E-10	2.03E-09
ENSG00000160213	CSTB	5375.95756	3522.262645	0.609912946	1.04E-10	2.03E-09
ENSG00000135362	PRR5L	518.2547829	239.7061655	1.115542059	1.09E-10	2.11E-09
ENSG00000196396	PTPN1	4528.430238	2478.27527	0.869291737	1.09E-10	2.12E-09
ENSG00000143878	RHOB	2922.761021	1674.10206	0.80339003	1.11E-10	2.16E-09
ENSG00000135116	HRK	114.366212	279.941021	-1.288757562	1.13E-10	2.20E-09
ENSG00000138678	GPAT3	1316.038789	562.3266781	1.225172855	1.17E-10	2.27E-09
ENSG00000181577	C6orf223	955.1303022	2006.286308	-1.070221123	1.20E-10	2.32E-09
ENSG00000134265	NAPG	2329.974539	1342.490443	0.794708216	1.21E-10	2.33E-09
ENSG00000064763	FAR2	450.2491132	883.4214245	-0.973364283	1.21E-10	2.34E-09
ENSG00000183605	SFXN4	815.0355197	1369.408705	-0.748939948	1.22E-10	2.35E-09
ENSG00000135842	FAM129A	329.871382	773.9868566	-1.230156716	1.24E-10	2.38E-09
ENSG00000101347	SAMHD1	684.2595554	1264.713271	-0.88687005	1.24E-10	2.39E-09
ENSG00000235934	AC007405.2	95.23371743	19.57484442	2.280261636	1.26E-10	2.43E-09
ENSG00000114698	PLSCR4	17.37854346	88.3194133	-2.340333979	1.28E-10	2.46E-09
ENSG00000143416	SELENBP1	716.3560887	1958.183329	-1.450218509	1.32E-10	2.53E-09
ENSG00000197782	ZNF780A	2625.191491	1429.111747	0.877921309	1.32E-10	2.54E-09
ENSG00000135069	PSAT1	7414.966766	12328.83695	-0.733597949	1.33E-10	2.55E-09
ENSG00000186642	PDE2A	348.4397428	136.9627237	1.343631168	1.38E-10	2.64E-09
ENSG00000249992	TMEM158	174.5663222	54.57015134	1.669480973	1.51E-10	2.89E-09
ENSG00000077044	DGKD	1994.705726	3070.65619	-0.622167051	1.55E-10	2.97E-09
ENSG00000131370	SH3BP5	1224.426617	645.3300446	0.922733844	1.62E-10	3.11E-09
ENSG00000198168	SVIP	534.6986391	1022.906663	-0.936905437	1.65E-10	3.16E-09
ENSG00000167325	RRM1	2663.060365	5512.971218	-1.049912703	1.67E-10	3.20E-09
ENSG00000196781	TLE1	2226.010412	1359.446706	0.711143679	1.74E-10	3.33E-09
ENSG00000196511	TPK1	77.66395957	256.2516268	-1.724012852	1.80E-10	3.44E-09
ENSG00000100219	XBP1	3896.199832	7688.343238	-0.980526218	2.06E-10	3.92E-09
ENSG00000162407	PLPP3	241.9575726	103.0108891	1.230137092	2.07E-10	3.94E-09
ENSG00000186834	HEXIM1	4731.125528	7191.420179	-0.60397739	2.10E-10	3.99E-09
ENSG00000172270	BSG	18232.6641	26718.28018	-0.551314309	2.12E-10	4.04E-09
ENSG00000283294	AP005212.4	52.61621606	4.72091422	3.457822211	2.14E-10	4.08E-09
ENSG00000137449	CPEB2	483.5796602	255.0396409	0.922659025	2.16E-10	4.11E-09
ENSG00000164465	DCBLD1	1358.525319	2164.350868	-0.671591166	2.17E-10	4.13E-09
ENSG00000266094	RASSF5	428.5334372	192.5154335	1.152654083	2.21E-10	4.19E-09
ENSG00000237380	HOXD-AS2	202.5484566	76.21453198	1.415042815	2.22E-10	4.20E-09

ENSG00000169435	RASSF6	192.4882372	63.32524862	1.59574307	2.42E-10	4.59E-09
ENSG00000155463	OXA1L	6635.313871	4373.312439	0.601349095	2.55E-10	4.82E-09
ENSG00000128849	CGNL1	7.196385516	64.78081169	-3.16212347	2.62E-10	4.96E-09
ENSG00000153879	CEBPG	2090.892322	3250.335056	-0.63647937	2.63E-10	4.97E-09
ENSG00000185591	SP1	6493.014285	9730.3154	-0.583536355	2.64E-10	4.99E-09
ENSG00000087077	TRIP6	1912.585987	2901.804144	-0.601354652	2.68E-10	5.05E-09
ENSG00000173581	CCDC106	182.088535	70.73567901	1.362426539	2.71E-10	5.11E-09
ENSG00000277476	AC005332.5	217.7552604	92.96033829	1.231849537	2.73E-10	5.14E-09
ENSG00000197079	KRT35	44.63972508	1.841203334	4.619848517	2.80E-10	5.27E-09
ENSG00000112339	HBS1L	1589.291351	2838.026465	-0.836721375	2.89E-10	5.44E-09
ENSG00000130175	PRKCSH	7018.172207	11027.10754	-0.651943714	2.98E-10	5.59E-09
ENSG00000173805	HAP1	6.884981971	59.90648534	-3.115265531	3.04E-10	5.71E-09
ENSG00000169105	CHST14	487.672481	863.6137255	-0.824375452	3.04E-10	5.71E-09
ENSG00000179051	RCC2	6896.716155	11232.51639	-0.703748816	3.10E-10	5.82E-09
ENSG00000102103	PQBP1	1083.446277	1697.085922	-0.647357582	3.35E-10	6.28E-09
ENSG00000185745	IFIT1	88.98231895	241.0814914	-1.438370167	3.35E-10	6.28E-09
ENSG00000114948	ADAM23	209.4147397	73.98966879	1.507943621	3.40E-10	6.36E-09
ENSG00000248690	HAS2-AS1	42.00294197	1.487016523	4.803931864	3.42E-10	6.40E-09
ENSG00000138095	LRPPRC	9493.735301	14851.38749	-0.64560508	3.49E-10	6.51E-09
ENSG00000121060	TRIM25	2382.582339	3664.966679	-0.621159424	3.49E-10	6.52E-09
ENSG00000170876	TMEM43	1459.385334	2382.532669	-0.706968277	3.51E-10	6.55E-09
ENSG00000174136	RGMB	210.0067761	432.659592	-1.04217216	3.65E-10	6.80E-09
ENSG00000197712	FAM114A1	2217.559685	1358.324351	0.706745306	3.66E-10	6.81E-09
ENSG00000178685	PARP10	766.1368052	1567.837664	-1.032411646	3.69E-10	6.86E-09
ENSG00000089220	PEBP1	8300.577086	12389.83269	-0.577911351	3.72E-10	6.92E-09
ENSG00000162194	LBHD1	188.2246587	400.4108453	-1.088662045	3.73E-10	6.92E-09
ENSG00000089127	OAS1	126.9179766	321.3224398	-1.339764886	3.73E-10	6.92E-09
ENSG00000230461	PROX1-AS1	49.36723967	0.994779868	5.757382725	3.86E-10	7.16E-09
ENSG00000023909	GCLM	2499.972754	1015.502552	1.298862435	3.88E-10	7.19E-09
ENSG00000104894	CD37	281.8168542	134.2549011	1.068671174	4.04E-10	7.48E-09
ENSG00000131409	LRRC4B	160.0880798	59.90210726	1.419379851	4.09E-10	7.57E-09
ENSG00000204455	TRIM51BP	43.06828366	2.418914951	4.155725335	4.49E-10	8.31E-09
ENSG00000162104	ADCY9	1170.805947	1832.08916	-0.645988552	4.51E-10	8.35E-09
ENSG00000143167	GPA33	148.4836796	36.68996492	2.026500248	4.53E-10	8.37E-09
ENSG00000227063	RPL41P1	1555.342906	2454.322038	-0.657834703	4.58E-10	8.45E-09
ENSG00000150782	IL18	711.6548733	1145.965141	-0.687188213	4.89E-10	9.01E-09
ENSG00000219481	NBPF1	711.6965717	1336.441774	-0.908590888	4.89E-10	9.01E-09
ENSG00000151116	UEVLD	376.7604182	670.8606691	-0.833213462	4.93E-10	9.08E-09
ENSG00000198842	DUSP27	43.69759193	3.350813379	3.714393447	4.95E-10	9.10E-09
ENSG00000130741	EIF2S3	7561.136112	11196.04689	-0.56628757	5.01E-10	9.21E-09
ENSG00000006459	KDM7A	6025.048732	3119.895337	0.949789009	5.07E-10	9.31E-09
ENSG00000277693	AP003900.1	150.6693777	55.91567825	1.425610667	5.19E-10	9.53E-09
ENSG00000152219	ARL14EP	434.6920614	730.7471665	-0.749111214	5.24E-10	9.61E-09

ENSG00000067057	PFKP	10779.79607	6494.466841	0.730907853	5.46E-10	1.00E-08
ENSG00000120875	DUSP4	369.3147841	177.9944777	1.052169713	5.48E-10	1.00E-08
ENSG00000148700	ADD3	3926.339917	6903.29654	-0.813926412	5.49E-10	1.00E-08
ENSG00000184164	CRELD2	1495.503321	3789.34925	-1.341260834	5.50E-10	1.01E-08
ENSG00000107581	EIF3A	17569.3536	25984.10694	-0.564556038	5.55E-10	1.01E-08
ENSG00000131242	RAB11FIP4	1749.813575	2754.270294	-0.654118922	5.61E-10	1.02E-08
ENSG00000179913	B3GNT3	1089.292886	1836.458334	-0.753242258	5.63E-10	1.03E-08
ENSG00000051009	FAM160A2	725.2835577	1172.000261	-0.69210073	5.64E-10	1.03E-08
ENSG00000142082	SIRT3	471.8950198	819.1680291	-0.795592533	5.75E-10	1.05E-08
ENSG00000144642	RBMS3	1.439531096	71.24842502	-5.614895281	5.75E-10	1.05E-08
ENSG00000169302	STK32A	210.8946992	73.458108	1.522020231	5.76E-10	1.05E-08
ENSG00000106615	RHEB	4217.411783	2494.768859	0.757041199	5.85E-10	1.06E-08
ENSG00000174885	NLRP6	418.1064715	158.5534218	1.401205071	5.95E-10	1.08E-08
ENSG00000271605	MILR1	40.18953542	2.06472814	4.258235834	6.01E-10	1.09E-08
ENSG00000053254	FOXN3	2387.178711	1547.406091	0.625752565	6.25E-10	1.13E-08
ENSG00000197217	ENTPD4	4121.457898	2774.929222	0.570588064	6.50E-10	1.18E-08
ENSG00000181634	TNFSF15	149.6295642	433.1336263	-1.53269465	6.51E-10	1.18E-08
ENSG00000183010	PYCR1	8146.55055	12987.14282	-0.672860537	6.59E-10	1.19E-08
ENSG00000183760	ACP7	311.2030085	147.414095	1.074752505	6.61E-10	1.20E-08
ENSG00000119698	PPP4R4	40.66108	3.005473767	3.754740763	6.67E-10	1.21E-08
ENSG00000180479	ZNF571	222.5468308	99.78729792	1.157768308	6.71E-10	1.21E-08
ENSG00000233922	LINC01694	0	42.41063643	-7.723787111	6.72E-10	1.21E-08
ENSG00000105971	CAV2	1431.133854	729.2517595	0.97150475	6.86E-10	1.24E-08
ENSG00000104687	GSR	10431.94652	5660.540322	0.881840993	6.89E-10	1.24E-08
ENSG00000221869	CEBPD	830.465553	488.2164848	0.765982048	7.08E-10	1.27E-08
ENSG00000173391	OLR1	3209.254538	5757.821286	-0.843300029	7.12E-10	1.28E-08
ENSG00000102879	CORO1A	1491.24743	2988.624334	-1.0031938	7.18E-10	1.29E-08
ENSG00000240891	PLCXD2	322.9026893	125.0298873	1.36428462	7.25E-10	1.30E-08
ENSG00000182718	ANXA2	50524.74524	28886.41449	0.806571355	7.25E-10	1.30E-08
ENSG00000259683	AC243562.2	1.108624	61.79484339	-5.818475604	7.28E-10	1.31E-08
ENSG00000166483	WEE1	1335.720354	2540.024108	-0.927517271	7.61E-10	1.37E-08
ENSG00000186174	BCL9L	5005.833685	3320.397791	0.592030777	7.78E-10	1.39E-08
ENSG00000179630	LACC1	327.5381024	163.2087581	1.005528864	7.84E-10	1.40E-08
ENSG00000057657	PRDM1	64.78761525	9.126978497	2.809805584	7.98E-10	1.43E-08
ENSG00000108828	VAT1	1576.810275	2904.628362	-0.881655501	7.98E-10	1.43E-08
ENSG00000164543	STK17A	1255.816803	729.0253803	0.784310127	8.19E-10	1.46E-08
ENSG00000062282	DGAT2	411.2064155	217.5483485	0.920407107	8.33E-10	1.49E-08
ENSG00000153246	PLA2R1	100.3982073	287.6148742	-1.515679999	8.47E-10	1.51E-08
ENSG00000203778	FAM229B	329.0025084	165.709721	0.987684	8.48E-10	1.51E-08
ENSG00000025423	HSD17B6	96.81309259	231.7221536	-1.259856948	8.59E-10	1.53E-08
ENSG00000267023	LRRC37A16P	522.6274236	275.7718634	0.924171783	8.80E-10	1.57E-08
ENSG00000197142	ACSL5	726.4624106	1490.924972	-1.036627995	8.84E-10	1.57E-08
ENSG00000142541	RPL13A	40463.72377	64717.38233	-0.677515301	8.99E-10	1.60E-08

ENSG00000085662	AKR1B1	48.6572259	4.916946379	3.268752648	9.05E-10	1.61E-08
ENSG00000029363	BCLAF1	3666.245784	6168.844908	-0.750822828	9.18E-10	1.63E-08
ENSG00000185585	OLFML2A	75.7348129	247.996402	-1.709102301	9.50E-10	1.68E-08
ENSG00000084234	APLP2	26287.2067	38911.74541	-0.565819776	9.61E-10	1.70E-08
ENSG00000161860	SYCE2	287.2523192	140.5812069	1.031477194	9.66E-10	1.71E-08
ENSG00000205531	NAP1L4	3130.269751	5186.810941	-0.728709502	1.00E-09	1.77E-08
ENSG00000102158	MAGT1	3204.390591	5271.281421	-0.718061909	1.01E-09	1.79E-08
ENSG00000204856	FAM216A	187.7998923	377.8054847	-1.009901824	1.05E-09	1.86E-08
ENSG00000101439	CST3	5113.03161	3027.421005	0.755869742	1.07E-09	1.88E-08
ENSG00000197019	SERTAD1	980.5128076	506.1010748	0.953136181	1.08E-09	1.91E-08
ENSG00000135387	CAPRIN1	8139.885181	12597.73458	-0.630135951	1.09E-09	1.92E-08
ENSG00000108387	4-Sep	152.1988067	52.9218921	1.53102598	1.12E-09	1.97E-08
ENSG00000035862	TIMP2	6219.370796	4098.718282	0.601619953	1.13E-09	2.00E-08
ENSG00000257151	PWAR6	45.99712935	0.900457708	5.668907209	1.14E-09	2.01E-08
ENSG00000149972	CNTN5	0	41.00917317	-7.677670675	1.15E-09	2.03E-08
ENSG00000183066	WBP2NL	141.2283069	35.10913454	2.020453309	1.16E-09	2.05E-08
ENSG00000170525	PFKFB3	2169.28169	1271.586826	0.770743854	1.18E-09	2.08E-08
ENSG00000131196	NFATC1	95.61853187	27.36316279	1.804136644	1.20E-09	2.11E-08
ENSG00000146950	SHROOM2	1323.93724	768.8521729	0.783526632	1.21E-09	2.13E-08
ENSG00000101670	LIPG	1253.231303	607.0072737	1.044478798	1.22E-09	2.14E-08
ENSG00000157110	RBPMS	495.5443251	830.7630322	-0.744932046	1.22E-09	2.14E-08
ENSG00000105974	CAV1	948.3171838	399.8552202	1.244070736	1.22E-09	2.14E-08
ENSG00000164638	SLC29A4	499.7918819	205.3976423	1.281677462	1.24E-09	2.17E-08
ENSG00000167600	CYP2S1	437.467256	739.9508383	-0.757790257	1.25E-09	2.18E-08
ENSG00000137145	DENND4C	2577.860674	3865.744939	-0.584399283	1.26E-09	2.21E-08
ENSG00000173418	NAA20	2816.168967	1779.391495	0.662018861	1.27E-09	2.22E-08
ENSG00000102317	RBM3	5068.032687	7779.780926	-0.618400965	1.28E-09	2.23E-08
ENSG00000226507	IPMKP1	51.95186194	8.192591356	2.67041867	1.29E-09	2.24E-08
ENSG00000215788	TNFRSF25	551.4489788	1154.723481	-1.065432054	1.30E-09	2.27E-08
ENSG00000250548	LINC01303	34.40794623	0	7.689038896	1.33E-09	2.32E-08
ENSG00000092758	COL9A3	204.8270691	400.8015158	-0.967045542	1.35E-09	2.34E-08
ENSG00000167508	MVD	2307.269114	1512.581225	0.608882774	1.37E-09	2.38E-08
ENSG00000188130	MAPK12	2741.15133	1722.150811	0.670784191	1.41E-09	2.44E-08
ENSG00000125257	ABCC4	1867.354865	3013.365861	-0.690585328	1.41E-09	2.46E-08
ENSG00000170745	KCNS3	318.9479968	126.6220408	1.330379254	1.46E-09	2.53E-08
ENSG00000177830	CHID1	1086.545726	1771.184205	-0.705310885	1.48E-09	2.56E-08
ENSG00000158186	MRAS	475.216454	252.4268053	0.913527005	1.49E-09	2.58E-08
ENSG00000008517	IL32	2060.538086	996.8855866	1.046602813	1.54E-09	2.66E-08
ENSG00000231500	RPS18	27686.20703	42155.22534	-0.60653237	1.54E-09	2.66E-08
ENSG00000099250	NRP1	733.4250845	377.0233495	0.961526047	1.56E-09	2.69E-08
ENSG00000116809	ZBTB17	1282.428665	731.986084	0.80802184	1.58E-09	2.73E-08
ENSG00000146476	ARMT1	849.8509546	1611.192617	-0.923412214	1.59E-09	2.74E-08
ENSG00000143322	ABL2	2198.473473	1240.784077	0.824516079	1.59E-09	2.74E-08

ENSG00000173227	SYT12	1558.127349	845.2736939	0.881338828	1.62E-09	2.79E-08
ENSG00000211455	STK38L	543.589055	928.1548687	-0.771279977	1.66E-09	2.85E-08
ENSG00000171931	FBXW10	61.79011042	10.62379327	2.52303431	1.70E-09	2.92E-08
ENSG00000228440	MTND5P6	33.68620005	0	7.65841798	1.71E-09	2.93E-08
ENSG00000198915	RASGEF1A	142.2977724	53.48937546	1.409148216	1.72E-09	2.96E-08
ENSG00000103942	HOMER2	410.608018	155.2895301	1.406671474	1.74E-09	2.98E-08
ENSG00000198830	HMGN2	14646.99869	21696.81342	-0.566907193	1.74E-09	2.98E-08
ENSG00000172159	FRMD3	38.69244968	146.8440191	-1.923617593	1.74E-09	2.98E-08
ENSG00000088832	FKBP1A	6491.458546	4310.126608	0.590642984	1.78E-09	3.05E-08
ENSG00000175895	PLEKHF2	1599.853258	2394.084948	-0.581504027	1.81E-09	3.09E-08
ENSG00000126903	SLC10A3	835.4982981	515.617733	0.696195233	1.83E-09	3.12E-08
ENSG00000116251	RPL22	1526.4454	2452.104638	-0.683667323	1.83E-09	3.14E-08
ENSG00000116191	RALGPS2	1155.234172	1924.390572	-0.736668814	1.86E-09	3.18E-08
ENSG00000162616	DNAJB4	1025.585073	575.9034616	0.831495971	1.88E-09	3.21E-08
ENSG00000169750	RAC3	134.224414	369.8554936	-1.460428195	1.88E-09	3.21E-08
ENSG00000237732	AC010980.1	52.74512114	8.416116161	2.644910299	1.92E-09	3.27E-08
ENSG00000133789	SWAP70	1077.512472	1869.352447	-0.795329178	1.94E-09	3.31E-08
ENSG00000184575	XPOT	5443.643645	8780.075636	-0.689611288	1.97E-09	3.35E-08
ENSG00000152465	NMT2	1168.11416	587.33816	0.990765768	1.99E-09	3.39E-08
ENSG00000130005	GAMT	1032.68502	1977.796635	-0.937512568	2.03E-09	3.46E-08
ENSG00000177706	FAM20C	137.1770414	52.22829416	1.394068783	2.06E-09	3.50E-08
ENSG00000116761	CTH	278.2747191	542.6624237	-0.96457585	2.06E-09	3.50E-08
ENSG00000137310	TCF19	1131.664187	1889.258063	-0.739778724	2.17E-09	3.68E-08
ENSG00000169994	MYO7B	1.108624	71.0012245	-6.018960127	2.18E-09	3.69E-08
ENSG00000177707	NECTIN3	1.44603228	46.58329935	-5.000673986	2.19E-09	3.70E-08
ENSG00000101966	XIAP	2343.068265	3418.737214	-0.545089647	2.22E-09	3.76E-08
ENSG00000167748	KLK1	1291.377396	678.1234596	0.930538387	2.22E-09	3.76E-08
ENSG00000054392	HHAT	124.315132	35.76889409	1.799976611	2.25E-09	3.80E-08
ENSG00000008018	PSMB1	3158.720542	5499.691598	-0.800168945	2.28E-09	3.86E-08
ENSG00000147100	SLC16A2	723.595863	389.3340099	0.894866472	2.32E-09	3.91E-08
ENSG00000253649	PRSS51	286.6099861	107.8790393	1.414946824	2.33E-09	3.93E-08
ENSG00000177045	SIX5	304.6999871	688.0420058	-1.173983862	2.34E-09	3.94E-08
ENSG00000158092	NCK1	461.0987931	763.945741	-0.728424602	2.39E-09	4.03E-08
ENSG00000144824	PHLDB2	5273.450828	2602.593003	1.019076303	2.40E-09	4.04E-08
ENSG00000130340	SNX9	1792.875021	2928.294381	-0.708006618	2.44E-09	4.11E-08
ENSG00000185177	ZNF479	54.02720249	0.277559048	7.3778969	2.45E-09	4.12E-08
ENSG00000255717	SNHG1	2094.440707	3223.909578	-0.622261996	2.49E-09	4.18E-08
ENSG00000128610	FEZF1	30.10108535	99.45072721	-1.721619567	2.51E-09	4.22E-08
ENSG00000164096	C4orf3	3168.805998	2013.490167	0.654684266	2.66E-09	4.47E-08
ENSG00000271303	SRXN1	706.0656113	261.0589798	1.432894094	2.67E-09	4.48E-08
ENSG00000214753	HNRNPUL2	1455.467925	2375.346195	-0.706981661	2.74E-09	4.59E-08
ENSG00000177685	CRACR2B	477.7808017	1010.588158	-1.079815386	2.76E-09	4.63E-08
ENSG00000188015	S100A3	44.12379081	4.03659348	3.478647145	2.77E-09	4.64E-08

ENSG00000138162	TACC2	2384.438529	3555.906327	-0.576632506	2.82E-09	4.72E-08
ENSG00000165516	KLHDC2	825.6958747	1287.608777	-0.640823935	2.87E-09	4.80E-08
ENSG00000204388	HSPA1B	2669.085542	1631.643293	0.709874041	2.88E-09	4.81E-08
ENSG00000179604	CDC42EP4	1136.223273	1736.566305	-0.612102118	2.93E-09	4.89E-08
ENSG00000174227	PIGG	2595.869017	1667.493088	0.63898112	2.95E-09	4.91E-08
ENSG00000203706	SERTAD4-AS1	80.13791035	21.81447055	1.881030799	3.00E-09	4.99E-08
ENSG00000176928	GCNT4	40.27547214	1.326373158	5.04012004	3.03E-09	5.05E-08
ENSG00000152527	PLEKHH2	24.86936889	112.0834689	-2.169170708	3.06E-09	5.09E-08
ENSG00000241749	RPSAP52	101.6149741	30.24433304	1.75139169	3.11E-09	5.17E-08
ENSG00000112029	FBXO5	587.2834679	1187.630101	-1.016643882	3.12E-09	5.19E-08
ENSG00000135074	ADAM19	67.33450038	15.05486148	2.159185837	3.14E-09	5.21E-08
ENSG00000213619	NDUFS3	3027.866423	1741.808903	0.79715423	3.20E-09	5.31E-08
ENSG00000120162	MOB3B	56.4919608	160.20834	-1.503695418	3.21E-09	5.32E-08
ENSG00000197912	SPG7	6249.997067	4307.376765	0.537149688	3.21E-09	5.32E-08
ENSG00000149182	ARFGAP2	4139.403592	2810.358745	0.558443212	3.27E-09	5.41E-08
ENSG00000159921	GNE	1387.94283	2541.781739	-0.873191634	3.28E-09	5.44E-08
ENSG00000271601	LIX1L	34.99666348	126.1946004	-1.855569731	3.29E-09	5.44E-08
ENSG00000187889	FYB2	39.74764288	1.532203567	4.714302478	3.37E-09	5.57E-08
ENSG00000173918	C1QTNF1	408.0020026	800.3136489	-0.971127259	3.37E-09	5.57E-08
ENSG00000151640	DPYSL4	47.65166483	5.864050491	3.044520856	3.38E-09	5.58E-08
ENSG00000172594	SMPDL3A	237.9088471	462.8444579	-0.958726748	3.39E-09	5.59E-08
ENSG00000185551	NR2F2	14479.51923	9159.406458	0.660591132	3.40E-09	5.60E-08
ENSG00000135838	NPL	205.1887357	93.66433383	1.130273133	3.42E-09	5.63E-08
ENSG00000120675	DNAJC15	292.9324294	511.1749644	-0.802679463	3.45E-09	5.69E-08
ENSG00000233461	AL445524.1	235.5887099	88.10644269	1.414127121	3.54E-09	5.82E-08
ENSG00000108298	RPL19	23533.45069	33929.27862	-0.527821325	3.57E-09	5.87E-08
ENSG00000051108	HERPUD1	2997.028904	6336.852617	-1.08015717	3.58E-09	5.89E-08
ENSG00000165923	AGBL2	5.513154006	63.32354163	-3.528597177	3.59E-09	5.89E-08
ENSG00000174705	SH3PXD2B	2901.701333	1641.211263	0.821691677	3.66E-09	6.01E-08
ENSG00000168488	ATXN2L	5621.208997	8559.348939	-0.606679861	3.81E-09	6.24E-08
ENSG00000166794	PPIB	4320.668733	6737.818162	-0.641025013	3.82E-09	6.26E-08
ENSG00000228223	HCG11	65.09235502	180.3292987	-1.473295028	4.02E-09	6.58E-08
ENSG00000167380	ZNF226	1534.152685	790.956789	0.956981269	4.07E-09	6.66E-08
ENSG00000267150	AC006557.1	51.07378462	7.143777245	2.827882555	4.11E-09	6.73E-08
ENSG00000173557	C2orf70	109.910152	31.36195707	1.802779745	4.14E-09	6.77E-08
ENSG00000143013	LMO4	1028.202958	1724.090636	-0.746032533	4.27E-09	6.97E-08
ENSG00000065154	OAT	3901.142299	5733.212037	-0.555470927	4.27E-09	6.97E-08
ENSG00000187609	EXD3	1252.987199	618.7120768	1.018986564	4.33E-09	7.07E-08
ENSG00000074590	NUAK1	1321.072276	855.8576041	0.62670556	4.43E-09	7.22E-08
ENSG00000184343	SRPK3	298.3230169	134.9101005	1.149057957	4.43E-09	7.22E-08
ENSG00000175213	ZNF408	773.5822878	478.3510858	0.692712206	4.45E-09	7.25E-08
ENSG00000178996	SNX18	1252.044559	748.7552782	0.740859332	4.53E-09	7.37E-08
ENSG00000197951	ZNF71	267.6146667	134.5250723	0.992457297	4.55E-09	7.39E-08

ENSG00000198948	MFAP3L	405.0809056	665.7995751	-0.716882602	4.60E-09	7.47E-08
ENSG00000119699	TGFB3	359.181608	899.3285207	-1.323278803	4.72E-09	7.67E-08
ENSG00000174695	TMEM167A	3606.104821	2325.800705	0.632495696	4.75E-09	7.70E-08
ENSG00000135480	KRT7	77.58975526	200.7357254	-1.370804079	4.83E-09	7.82E-08
ENSG00000114126	TFDP2	2331.352341	3812.844453	-0.709434185	4.87E-09	7.89E-08
ENSG00000174130	TLR6	80.63164978	12.71755172	2.640066934	4.96E-09	8.03E-08
ENSG00000271201	AC247036.1	0	37.16011636	-7.529571664	5.03E-09	8.14E-08
ENSG00000144283	PKP4	7211.239591	4703.914388	0.616285465	5.05E-09	8.17E-08
ENSG00000087253	LPCAT2	2184.10942	1316.095246	0.731458086	5.07E-09	8.20E-08
ENSG00000160229	ZNF66	29.75321463	0	7.478875464	5.12E-09	8.26E-08
ENSG00000275410	HNF1B	301.1369047	568.216385	-0.916434068	5.15E-09	8.31E-08
ENSG00000149428	HYOU1	8517.875122	16808.30672	-0.980587356	5.20E-09	8.39E-08
ENSG00000183955	KMT5A	2312.888689	3480.366372	-0.589798394	5.25E-09	8.46E-08
ENSG00000172493	AFF1	3940.76994	2745.780337	0.521253521	5.34E-09	8.61E-08
ENSG00000132286	TIMM10B	531.3619654	908.1277222	-0.77293255	5.38E-09	8.67E-08
ENSG00000100316	RPL3	49711.25062	75842.15957	-0.609419755	5.44E-09	8.75E-08
ENSG00000184743	ATL3	2763.994986	1852.630065	0.577375091	5.74E-09	9.22E-08
ENSG00000204634	TBC1D8	914.3774264	1462.786661	-0.677795122	5.81E-09	9.34E-08
ENSG00000127152	BCL11B	55.44976996	149.5710083	-1.429512742	5.83E-09	9.36E-08
ENSG00000278921	EPB41L4A-DT	42.08887869	3.427441143	3.649268538	5.86E-09	9.41E-08
ENSG00000168907	PLA2G4F	7.615769258	56.59999899	-2.895076497	5.88E-09	9.44E-08
ENSG00000168374	ARF4	8903.785591	5491.090572	0.697142262	5.90E-09	9.45E-08
ENSG00000256124	LINC01152	1.090390412	45.3320946	-5.376659526	6.02E-09	9.65E-08
ENSG00000142920	AZIN2	319.8525554	156.0089662	1.03807372	6.07E-09	9.72E-08
ENSG00000177731	FLII	10127.1071	6200.822438	0.707541444	6.20E-09	9.91E-08
ENSG00000137699	TRIM29	376.2712455	174.675444	1.110171668	6.21E-09	9.92E-08
ENSG00000090520	DNAJB11	1870.000714	3134.977138	-0.745423506	6.26E-09	1.00E-07
ENSG00000158555	GDPD5	770.9182413	370.3164337	1.060080185	6.33E-09	1.01E-07
ENSG00000147010	SH3KBP1	3148.445001	2138.66205	0.557755373	6.35E-09	1.01E-07
ENSG00000130590	SAMD10	589.9820808	352.6841235	0.741844108	6.36E-09	1.01E-07
ENSG00000106066	CPVL	933.5513054	556.7902333	0.746451969	6.52E-09	1.04E-07
ENSG00000113758	DBN1	2287.759256	1490.853128	0.617449764	6.66E-09	1.06E-07
ENSG00000125895	TMEM74B	349.9558667	160.2547388	1.131168601	6.66E-09	1.06E-07
ENSG00000121940	CLCC1	912.492521	1402.558066	-0.620275222	6.73E-09	1.07E-07
ENSG00000128242	GAL3ST1	0.716514956	56.28017162	-6.277289337	6.79E-09	1.08E-07
ENSG00000206417	H1FX-AS1	34.03026088	128.666781	-1.915085288	6.85E-09	1.09E-07
ENSG00000173852	DPY19L1	2806.604222	1641.633673	0.773215513	6.98E-09	1.11E-07
ENSG00000105550	FGF21	41.39709851	141.4844394	-1.772366024	7.06E-09	1.12E-07
ENSG00000054967	RELT	505.9866818	258.9591108	0.96505667	7.14E-09	1.13E-07
ENSG00000224093	AL109613.1	166.8390881	379.4810675	-1.183352776	7.18E-09	1.14E-07
ENSG00000100065	CARD10	588.1334741	1016.544355	-0.788752226	7.22E-09	1.14E-07
ENSG00000285925	AC007391.2	46.36942088	5.334014631	3.103883324	7.26E-09	1.15E-07
ENSG00000171853	TRAPPC12	2283.612584	1553.060413	0.556116385	7.37E-09	1.16E-07

ENSG00000166106	ADAMTS15	137.7218343	44.12125606	1.637582912	7.67E-09	1.21E-07
ENSG00000224723	GUSBP10	36.01284958	1.263491718	4.889469268	7.91E-09	1.25E-07
ENSG00000170345	FOS	519.0826196	219.4490317	1.24047298	8.12E-09	1.28E-07
ENSG00000142634	EFHD2	3354.142402	2272.569997	0.561450356	8.15E-09	1.28E-07
ENSG00000089006	SNX5	4083.504226	6303.846084	-0.626564201	8.51E-09	1.34E-07
ENSG00000196141	SPATS2L	914.21414	1621.126147	-0.826821321	8.62E-09	1.36E-07
ENSG00000103528	SYT17	105.1858165	308.7815845	-1.550796857	8.64E-09	1.36E-07
ENSG00000176771	NCKAP5	29.2204681	0	7.454122858	8.84E-09	1.39E-07
ENSG00000196526	AFAP1	1046.807952	1724.986277	-0.720137697	8.88E-09	1.40E-07
ENSG00000155366	RHOC	3113.19921	2046.786814	0.604726538	8.98E-09	1.41E-07
ENSG00000184009	ACTG1	72215.53323	45320.51028	0.672121368	9.03E-09	1.42E-07
ENSG00000165916	PSMC3	7098.871314	3857.090731	0.879823571	9.03E-09	1.42E-07
ENSG00000042286	AIFM2	3524.956816	1932.989743	0.866268936	9.05E-09	1.42E-07
ENSG00000183853	KIRREL1	1925.634869	1209.758261	0.670224802	9.07E-09	1.42E-07
ENSG00000106571	GLI3	27.93330689	0	7.3878752	9.13E-09	1.43E-07
ENSG00000122359	ANXA11	7169.939954	10468.50189	-0.546048289	9.19E-09	1.44E-07
ENSG00000151694	ADAM17	2333.256801	1413.097585	0.722853093	9.28E-09	1.45E-07
ENSG00000108679	LGALS3BP	12936.45474	19496.11961	-0.591701684	9.28E-09	1.45E-07
ENSG00000182919	C11orf54	605.3856442	957.5802709	-0.661208492	9.29E-09	1.45E-07
ENSG00000250770	AC005865.2	47.47312762	7.560845497	2.654062633	9.40E-09	1.47E-07
ENSG00000115363	EVA1A	139.8999238	49.04800069	1.505740629	9.63E-09	1.50E-07
ENSG00000263325	AC003965.2	73.72716426	19.86519874	1.890692458	9.79E-09	1.53E-07
ENSG00000090530	P3H2	68.04055289	15.68316757	2.130512514	9.91E-09	1.54E-07
ENSG00000069869	NEDD4	1298.062173	1972.239096	-0.603557927	9.92E-09	1.54E-07
ENSG00000213186	TRIM59	426.295884	736.6717405	-0.790000674	1.01E-08	1.57E-07
ENSG00000144821	MYH15	71.88522426	12.39618669	2.553407586	1.01E-08	1.57E-07
ENSG00000148908	RGS10	673.1214319	1125.780517	-0.741754027	1.01E-08	1.57E-07
ENSG00000140545	MFGE8	3690.380297	2396.861647	0.622993736	1.01E-08	1.57E-07
ENSG00000183044	ABAT	393.5374046	709.4556046	-0.849092554	1.01E-08	1.57E-07
ENSG00000248905	FMN1	1155.622656	708.7596746	0.706132472	1.01E-08	1.57E-07
ENSG00000183379	SYNDIG1L	46.21451107	6.759609074	2.789338148	1.01E-08	1.57E-07
ENSG00000163283	ALPP	17.81885208	83.99115001	-2.235816814	1.04E-08	1.62E-07
ENSG00000139445	FOXN4	54.44927753	231.5662531	-2.086281303	1.06E-08	1.64E-07
ENSG00000163069	SGCB	1627.858406	2730.033988	-0.745600068	1.06E-08	1.64E-07
ENSG00000204628	RACK1	32794.04755	48293.74658	-0.558389027	1.06E-08	1.64E-07
ENSG00000163513	TGFBR2	2199.304301	1400.279728	0.651659209	1.06E-08	1.64E-07
ENSG00000156675	RAB11FIP1	2012.934304	2978.379249	-0.565138514	1.06E-08	1.64E-07
ENSG00000123444	KBTBD4	490.7909572	281.0284686	0.803707868	1.07E-08	1.65E-07
ENSG00000182287	AP1S2	589.9442035	336.0883551	0.811542763	1.07E-08	1.66E-07
ENSG00000167555	ZNF528	38.36819513	1.841203334	4.398752056	1.08E-08	1.66E-07
ENSG00000184060	ADAP2	163.0621198	323.1370504	-0.988023338	1.09E-08	1.67E-07
ENSG00000100994	PYGB	3403.774351	6131.099698	-0.848987228	1.11E-08	1.70E-07
ENSG00000072182	ASIC4	86.23898819	19.22211697	2.173290014	1.12E-08	1.72E-07

ENSG00000276850	AC245041.2	30.48527188	106.3071472	-1.800711606	1.14E-08	1.76E-07
ENSG00000118508	RAB32	0	31.70634563	-7.306720678	1.15E-08	1.77E-07
ENSG00000100804	PSMB5	4970.436755	2669.199293	0.896604479	1.17E-08	1.80E-07
ENSG00000177556	ATOX1	1179.183378	674.1269713	0.805932241	1.18E-08	1.81E-07
ENSG00000110911	SLC11A2	1069.653661	1626.399326	-0.604313817	1.23E-08	1.88E-07
ENSG00000174684	B4GAT1	401.1065357	669.044796	-0.737634736	1.24E-08	1.90E-07
ENSG00000158813	EDA	238.3816616	109.9149848	1.114478604	1.28E-08	1.96E-07
ENSG00000099957	P2RX6	156.129426	52.77697546	1.568014041	1.32E-08	2.02E-07
ENSG00000224189	HAGLR	613.1861349	286.989512	1.092873174	1.32E-08	2.02E-07
ENSG00000246022	ALDH1L1-AS2	17.01116918	74.76729507	-2.13176643	1.35E-08	2.06E-07
ENSG00000166452	AKIP1	415.736192	792.9197808	-0.93247298	1.35E-08	2.06E-07
ENSG00000204520	MICA	1158.171416	701.3400068	0.7230298	1.37E-08	2.09E-07
ENSG00000204936	CD177	1.06565564	55.77644245	-5.680816848	1.39E-08	2.13E-07
ENSG00000092445	TYRO3	1139.184802	1833.523759	-0.686784165	1.40E-08	2.14E-07
ENSG00000099377	HSD3B7	221.957637	426.3499911	-0.943236188	1.41E-08	2.15E-07
ENSG00000204528	PSORS1C3	1.090390412	41.51756656	-5.252461499	1.41E-08	2.15E-07
ENSG00000147231	CXorf57	37.24118618	3.359660578	3.481875391	1.41E-08	2.15E-07
ENSG00000248898	AC022126.1	34.23972009	1.787169093	4.244974925	1.44E-08	2.18E-07
ENSG00000178026	LRRC75B	121.5510165	355.7553449	-1.547240187	1.44E-08	2.19E-07
ENSG00000158201	ABHD3	845.9178291	1335.523206	-0.659217836	1.47E-08	2.24E-07
ENSG00000107984	DKK1	2.137812464	116.6086769	-5.756336117	1.47E-08	2.24E-07
ENSG00000154162	CDH12	214.3003207	89.38891883	1.267321589	1.48E-08	2.24E-07
ENSG00000107864	CPEB3	230.4007553	454.6149656	-0.980136143	1.48E-08	2.24E-07
ENSG00000176697	BDNF	321.4154464	122.5985033	1.39465032	1.48E-08	2.24E-07
ENSG00000170791	CHCHD7	1157.464422	752.716443	0.620669157	1.49E-08	2.26E-07
ENSG00000011347	SYT7	5644.987622	3640.453457	0.632966835	1.50E-08	2.28E-07
ENSG00000180537	RNF182	3.295906007	38.87658617	-3.56734538	1.52E-08	2.30E-07
ENSG00000138604	GLCE	2345.463335	1536.465583	0.610282878	1.53E-08	2.32E-07
ENSG00000061794	MRPS35	1846.947115	2865.0606	-0.633672896	1.54E-08	2.33E-07
ENSG00000239521	CASTOR3	500.8009165	801.2356978	-0.677663075	1.55E-08	2.34E-07
ENSG00000167291	TBC1D16	2060.840538	3218.354554	-0.643139224	1.57E-08	2.37E-07
ENSG00000114480	GBE1	1497.624809	934.6900672	0.679613891	1.57E-08	2.37E-07
ENSG00000172986	GXYLT2	1139.389983	740.7585242	0.621356236	1.57E-08	2.37E-07
ENSG00000168036	CTNNB1	19354.99928	13095.57892	0.563577648	1.59E-08	2.39E-07
ENSG00000244405	ETV5	2903.201688	1543.521968	0.91077626	1.59E-08	2.39E-07
ENSG00000132002	DNAJB1	5686.878646	3833.089582	0.568911382	1.67E-08	2.52E-07
ENSG00000132744	ACY3	86.28291256	202.2109505	-1.226097535	1.71E-08	2.57E-07
ENSG00000272505	AC104964.3	208.7700405	81.8422624	1.355169002	1.72E-08	2.58E-07
ENSG00000166669	ATF7IP2	305.9440174	589.6930463	-0.945246974	1.74E-08	2.61E-07
ENSG00000198242	RPL23A	7499.439407	10670.29124	-0.508781957	1.74E-08	2.62E-07
ENSG00000183150	GPR19	83.67466289	24.95945352	1.750790459	1.77E-08	2.65E-07
ENSG00000174521	TTC9B	78.90275861	19.16962039	2.037269604	1.77E-08	2.65E-07
ENSG00000154874	CCDC144B	46.08956725	120.5401591	-1.3867295	1.77E-08	2.65E-07

ENSG00000104805	NUCB1	2506.193793	3841.950532	-0.616159771	1.80E-08	2.70E-07
ENSG00000115596	WNT6	84.41337269	23.71460726	1.836071846	1.81E-08	2.71E-07
ENSG00000134575	ACP2	1777.78128	1210.958518	0.554133951	1.85E-08	2.76E-07
ENSG00000162426	SLC45A1	51.12736679	139.299578	-1.444586337	1.87E-08	2.80E-07
ENSG00000196967	ZNF585A	489.5791189	280.554708	0.805247172	1.92E-08	2.87E-07
ENSG00000099860	GADD45B	2201.941247	1494.262796	0.559629237	1.99E-08	2.96E-07
ENSG00000137628	DDX60	119.2462478	258.2835462	-1.11372558	2.01E-08	2.99E-07
ENSG00000260220	CCDC187	21.26729056	92.86344912	-2.11929504	2.01E-08	3.00E-07
ENSG00000137571	SLCO5A1	28.14814869	103.9173535	-1.890980329	2.03E-08	3.02E-07
ENSG00000161267	BDH1	1648.67065	2450.833562	-0.572164526	2.04E-08	3.03E-07
ENSG00000139269	INHBE	17.13357308	83.52721318	-2.286079836	2.07E-08	3.08E-07
ENSG00000147601	TERF1	1119.487542	1737.249859	-0.633581526	2.09E-08	3.10E-07
ENSG00000104635	SLC39A14	7070.94159	10627.08728	-0.587725618	2.09E-08	3.10E-07
ENSG00000102580	DNAJC3	2456.24579	4520.963849	-0.880102414	2.14E-08	3.18E-07
ENSG00000135999	EPC2	895.3625698	1375.180545	-0.618770189	2.16E-08	3.20E-07
ENSG00000034053	APBA2	35.78484283	2.342287188	3.901158154	2.19E-08	3.25E-07
ENSG00000142188	TMEM50B	923.650274	1810.256927	-0.970474424	2.22E-08	3.28E-07
ENSG00000196531	NACA	8731.75441	12327.77535	-0.497557223	2.23E-08	3.30E-07
ENSG00000168067	MAP4K2	510.5166209	887.3581846	-0.798002344	2.23E-08	3.31E-07
ENSG00000132938	MTUS2	123.0214696	31.22580933	1.975917613	2.24E-08	3.32E-07
ENSG00000150403	TMCO3	3239.741105	5119.148639	-0.659950189	2.26E-08	3.34E-07
ENSG00000054793	ATP9A	1761.013514	2713.559071	-0.623394423	2.26E-08	3.34E-07
ENSG00000173598	NUDT4	2777.553416	4056.128864	-0.546089418	2.28E-08	3.37E-07
ENSG00000125450	NUP85	1260.210052	1912.699854	-0.602292656	2.31E-08	3.41E-07
ENSG00000109205	ODAM	0	32.1818262	-7.322304326	2.40E-08	3.54E-07
ENSG00000162069	BICDL2	658.9132452	1250.082082	-0.923169691	2.40E-08	3.54E-07
ENSG00000134030	CTIF	2309.644761	1460.705488	0.661485914	2.43E-08	3.58E-07
ENSG00000128590	DNAJB9	706.4206028	1403.312198	-0.989792765	2.45E-08	3.61E-07
ENSG00000265787	CYP4F35P	42.55123079	0.631745859	6.135639612	2.47E-08	3.63E-07
ENSG00000170209	ANKK1	36.80119151	2.942592326	3.621572972	2.48E-08	3.64E-07
ENSG00000105784	RUNDC3B	45.93307352	123.8074652	-1.431718699	2.51E-08	3.69E-07
ENSG00000222041	CYTOR	551.310556	273.0660082	1.010974248	2.52E-08	3.71E-07
ENSG00000128833	MYO5C	1490.597483	2262.312651	-0.602203008	2.53E-08	3.72E-07
ENSG00000174444	RPL4	47763.16229	68727.91461	-0.524987942	2.66E-08	3.90E-07
ENSG00000106367	AP1S1	6169.031508	4016.617809	0.618881801	2.67E-08	3.92E-07
ENSG00000082497	SERTAD4	470.6289685	248.456624	0.920629744	2.71E-08	3.97E-07
ENSG00000197935	ZNF311	587.2647436	350.7553738	0.745070082	2.72E-08	3.99E-07
ENSG00000170442	KRT86	117.3859117	252.2448056	-1.101223888	2.73E-08	4.00E-07
ENSG00000105819	PMPCB	1789.774467	2557.96759	-0.515120467	2.76E-08	4.04E-07
ENSG00000152380	FAM151B	9.77704653	52.33395223	-2.418409353	2.78E-08	4.06E-07
ENSG00000016402	IL20RA	556.8814273	306.457148	0.859572895	2.79E-08	4.07E-07
ENSG00000100399	CHADL	22.80052952	93.12331377	-2.025215642	2.79E-08	4.08E-07
ENSG00000140463	BBS4	329.4674004	560.5302451	-0.765728923	2.80E-08	4.08E-07

ENSG00000047634	SCML1	1330.255251	680.7963117	0.965303227	2.91E-08	4.25E-07
ENSG00000233760	AC004947.1	41.48684512	3.790475153	3.492866375	2.96E-08	4.32E-07
ENSG00000163412	EIF4E3	422.3192993	224.2162934	0.911882181	2.96E-08	4.32E-07
ENSG00000088002	SULT2B1	679.5821607	1154.651335	-0.764110408	3.02E-08	4.40E-07
ENSG00000003400	CASP10	614.4744035	976.5395005	-0.668155834	3.03E-08	4.41E-07
ENSG00000183625	CCR3	40.137212	3.429929856	3.498510105	3.16E-08	4.59E-07
ENSG00000160113	NR2F6	2518.485547	3884.238414	-0.625013962	3.23E-08	4.70E-07
ENSG00000198755	RPL10A	11845.48191	16436.31899	-0.472557682	3.24E-08	4.70E-07
ENSG00000119185	ITGB1BP1	1934.696973	1143.678973	0.757794767	3.29E-08	4.78E-07
ENSG00000109919	MTCH2	4817.209332	2651.518214	0.861018359	3.33E-08	4.83E-07
ENSG00000059145	UNKL	1350.19715	897.8299598	0.589232809	3.38E-08	4.90E-07
ENSG00000134531	EMP1	1314.326847	548.2173847	1.260414359	3.46E-08	5.02E-07
ENSG00000005238	FAM214B	563.0405392	347.5544672	0.696567844	3.49E-08	5.06E-07
ENSG00000013375	PGM3	1449.311493	2260.223072	-0.641135079	3.54E-08	5.12E-07
ENSG00000162009	SSTR5	70.21753505	15.98675991	2.135795345	3.56E-08	5.15E-07
ENSG00000138448	ITGAV	7477.18152	5213.218234	0.520321981	3.57E-08	5.16E-07
ENSG00000108669	CYTH1	1636.622246	2448.747206	-0.581444694	3.74E-08	5.40E-07
ENSG00000110400	NECTIN1	638.4637606	315.8278872	1.014907188	3.75E-08	5.42E-07
ENSG00000143578	CREB3L4	420.9802688	772.1076462	-0.874241753	3.76E-08	5.42E-07
ENSG00000146733	PSPH	1676.58239	2637.275595	-0.653753649	3.81E-08	5.49E-07
ENSG00000275023	MLLT6	7821.752235	11109.18015	-0.506121756	3.81E-08	5.50E-07
ENSG00000103024	NME3	1388.882313	2283.633892	-0.716989666	3.84E-08	5.53E-07
ENSG00000077585	GPR137B	1059.645905	692.9405941	0.613352305	3.84E-08	5.53E-07
ENSG00000170889	RPS9	15749.7595	22951.99928	-0.543295044	3.85E-08	5.53E-07
ENSG00000130487	KLHDC7B	3.638545507	42.29759052	-3.537943981	3.85E-08	5.53E-07
ENSG00000184613	NELL2	35.46170688	2.195390146	4.052621713	3.87E-08	5.56E-07
ENSG00000170485	NPAS2	763.7630888	436.1956498	0.806694337	3.88E-08	5.58E-07
ENSG00000103089	FA2H	221.4161521	422.3411382	-0.931116271	3.89E-08	5.58E-07
ENSG00000113742	CPEB4	982.8849235	621.9085283	0.659454631	3.92E-08	5.63E-07
ENSG00000115902	SLC1A4	1980.969645	3640.614	-0.877863775	3.95E-08	5.67E-07
ENSG00000121236	TRIM6	10.90517408	59.43048373	-2.443443477	3.97E-08	5.68E-07
ENSG00000131981	LGALS3	7001.056299	11221.79904	-0.680590515	3.98E-08	5.70E-07
ENSG00000182985	CADM1	3405.775995	2024.835105	0.75009718	4.02E-08	5.75E-07
ENSG00000049323	LTBP1	357.1587534	618.1621853	-0.79085381	4.02E-08	5.75E-07
ENSG00000117245	KIF17	107.0001678	39.27450066	1.446331787	4.11E-08	5.88E-07
ENSG00000168994	PXDC1	602.4111359	343.2602244	0.810463517	4.39E-08	6.28E-07
ENSG00000169981	ZNF35	460.0616709	264.6403672	0.795961426	4.41E-08	6.30E-07
ENSG00000138669	PRKG2	93.36957137	27.03448822	1.79746563	4.43E-08	6.33E-07
ENSG00000204611	ZNF616	556.6302697	328.2082033	0.76183519	4.46E-08	6.37E-07
ENSG00000133315	MACROD1	816.225791	1344.268802	-0.719459566	4.52E-08	6.45E-07
ENSG00000110318	CEP126	130.3583412	48.83916053	1.422742818	4.58E-08	6.53E-07
ENSG00000101935	AMMECR1	1012.255244	1600.370629	-0.66110039	4.66E-08	6.64E-07
ENSG00000168427	KLHL30	80.10809573	21.43425046	1.912042334	4.77E-08	6.79E-07

ENSG00000140859	KIFC3	3290.54933	2112.782361	0.639646199	4.77E-08	6.79E-07
ENSG00000141101	NOB1	2009.726022	3192.468488	-0.667551352	4.78E-08	6.80E-07
ENSG00000172575	RASGRP1	383.5685045	208.8655664	0.878681262	4.85E-08	6.89E-07
ENSG00000167037	SGSM1	258.9428996	492.6398265	-0.926336985	4.90E-08	6.96E-07
ENSG00000157214	STEAP2	1655.622234	987.1800772	0.745978337	4.92E-08	6.98E-07
ENSG00000168679	SLC16A4	296.9537176	149.1790095	0.996391051	4.93E-08	6.99E-07
ENSG00000225177	FLJ46906	82.05025599	209.0932599	-1.348533649	4.97E-08	7.04E-07
ENSG00000176597	B3GNT5	1118.772449	698.4767642	0.678639698	5.05E-08	7.16E-07
ENSG00000166007	TRIM51HP	23.27730532	0	7.124143162	5.08E-08	7.19E-07
ENSG00000125968	ID1	3136.294779	6377.307312	-1.023693482	5.13E-08	7.27E-07
ENSG00000115841	RMDN2	195.922252	85.94327498	1.193006768	5.24E-08	7.41E-07
ENSG00000132205	EMILIN2	2.879062192	36.78539473	-3.667523349	5.27E-08	7.44E-07
ENSG00000154229	PRKCA	2077.408127	1307.103203	0.667928524	5.27E-08	7.44E-07
ENSG00000063127	SLC6A16	145.8729012	60.7701732	1.26570208	5.32E-08	7.52E-07
ENSG00000173702	MUC13	202.9781514	398.8795673	-0.973106399	5.33E-08	7.52E-07
ENSG00000172175	MALT1	1664.883469	1058.925532	0.65204967	5.42E-08	7.64E-07
ENSG00000276071	AC074138.1	73.13686308	18.5417443	1.994729738	5.45E-08	7.69E-07
ENSG00000126705	AHDC1	2349.140773	1482.366379	0.663903263	5.52E-08	7.78E-07
ENSG00000137968	SLC44A5	357.3369767	157.2894747	1.188365892	5.61E-08	7.90E-07
ENSG00000064270	ATP2C2	1367.369509	2351.399472	-0.781923047	5.65E-08	7.94E-07
ENSG00000186594	MIR22HG	531.3421703	255.5965955	1.0552093	5.69E-08	8.00E-07
ENSG00000167971	CASKIN1	121.9348891	309.2330905	-1.340408671	5.69E-08	8.00E-07
ENSG00000245468	LINC02447	22.32644502	83.17266193	-1.890209828	5.72E-08	8.04E-07
ENSG00000111252	SH2B3	773.8813057	1572.831812	-1.023692077	5.73E-08	8.04E-07
ENSG00000146535	GNA12	2881.860143	1974.343276	0.545407412	5.96E-08	8.36E-07
ENSG00000148154	UGCG	2664.446785	1800.598689	0.565564042	5.97E-08	8.37E-07
ENSG00000140470	ADAMTS17	25.29144393	96.14451426	-1.922052588	6.00E-08	8.41E-07
ENSG00000103269	RHBDL1	343.9897157	770.4726508	-1.162276929	6.06E-08	8.48E-07
ENSG00000196517	SLC6A9	866.0781748	1596.940968	-0.882699111	6.07E-08	8.49E-07
ENSG00000164850	GPER1	118.4412562	247.2769003	-1.059717046	6.26E-08	8.75E-07
ENSG00000198113	TOR4A	3917.02086	2546.629701	0.621029782	6.34E-08	8.87E-07
ENSG00000124374	PAIP2B	555.0489966	879.7215844	-0.664200276	6.36E-08	8.88E-07
ENSG00000072682	P4HA2	2416.469271	1584.353182	0.608638803	6.38E-08	8.91E-07
ENSG00000060656	PTPRU	3346.207116	4784.763511	-0.515879869	6.60E-08	9.21E-07
ENSG00000180423	HARBI1	189.5311608	91.22282536	1.053716918	6.61E-08	9.22E-07
ENSG00000166165	CKB	191.4309807	360.4369493	-0.910923473	6.65E-08	9.27E-07
ENSG00000204682	CASC10	1214.881692	740.1965524	0.715859166	6.67E-08	9.29E-07
ENSG00000165959	CLMN	716.2766195	1076.50782	-0.587496889	6.74E-08	9.39E-07
ENSG00000246695	RASSF8-AS1	144.8849346	56.86321237	1.355188429	6.85E-08	9.54E-07
ENSG00000111728	ST8SIA1	7.902438031	66.02643968	-3.050932595	6.91E-08	9.61E-07
ENSG00000066248	NGEF	166.1346307	347.2392783	-1.062785364	6.94E-08	9.65E-07
ENSG00000197249	SERPINA1	838.0423264	330.073746	1.346405326	7.04E-08	9.78E-07
ENSG00000157014	TATDN2	610.255055	973.3066576	-0.674401693	7.06E-08	9.80E-07

ENSG00000236908	AC005865.1	46.61565	4.0680342	3.545767158	7.07E-08	9.82E-07
ENSG00000170638	TRABD	2038.117931	3048.457444	-0.580729468	7.15E-08	9.92E-07
ENSG00000257138	TAS2R38	91.47689187	20.70225396	2.162890386	7.31E-08	1.01E-06
ENSG00000198821	CD247	35.65324645	4.169744199	3.084148527	7.45E-08	1.03E-06
ENSG00000181007	ZFP82	33.58202975	2.055880942	4.002831984	7.50E-08	1.04E-06
ENSG00000134240	HMGCS2	37.45095934	0.331593289	6.84851852	7.64E-08	1.06E-06
ENSG00000110002	VWA5A	327.206542	173.876709	0.913704402	7.68E-08	1.06E-06
ENSG00000233927	RPS28	7848.845795	11569.82077	-0.559816347	7.76E-08	1.07E-06
ENSG00000247317	LY6E-DT	25.24324435	85.1603323	-1.750983752	7.82E-08	1.08E-06
ENSG00000082014	SMARCD3	1917.577835	1096.593011	0.807001962	7.88E-08	1.09E-06
ENSG00000164305	CASP3	1183.814067	1797.079636	-0.602710586	7.91E-08	1.09E-06
ENSG00000184489	PTP4A3	471.4249077	268.0651503	0.816217938	7.94E-08	1.09E-06
ENSG00000111052	LIN7A	40.13848196	5.647913524	2.815299788	7.99E-08	1.10E-06
ENSG00000245849	RAD51-AS1	278.35857	608.4005282	-1.127074208	8.06E-08	1.11E-06
ENSG00000123159	GIPC1	3230.642057	4787.714467	-0.567625824	8.07E-08	1.11E-06
ENSG00000108352	RAPGEFL1	612.918667	1044.606783	-0.768366809	8.10E-08	1.12E-06
ENSG00000171634	BPTF	3432.947332	4819.55953	-0.489487124	8.13E-08	1.12E-06
ENSG00000076928	ARHGEF1	1874.682087	2812.6357	-0.585321548	8.34E-08	1.15E-06
ENSG00000184979	USP18	321.071223	594.322101	-0.889661444	8.38E-08	1.15E-06
ENSG00000116525	TRIM62	696.3646615	423.9894102	0.714471765	8.41E-08	1.15E-06
ENSG00000147649	MTDH	6256.292665	8962.037314	-0.518591067	8.51E-08	1.17E-06
ENSG00000174652	ZNF266	1271.810836	823.4305323	0.627673353	8.56E-08	1.17E-06
ENSG00000136490	LIMD2	43.93875242	113.7447185	-1.371918425	8.57E-08	1.18E-06
ENSG00000172590	MRPL52	2081.093614	1282.090692	0.698221409	8.75E-08	1.20E-06
ENSG00000028839	TBPL1	227.1500324	400.5344326	-0.819441071	8.90E-08	1.22E-06
ENSG00000169962	TAS1R3	54.69424791	143.1778602	-1.384742402	8.94E-08	1.22E-06
ENSG00000180535	BHLHA15	97.12418218	254.2604368	-1.389932723	8.97E-08	1.23E-06
ENSG00000113638	TTC33	338.1526352	549.8949463	-0.702113731	8.97E-08	1.23E-06
ENSG00000181852	RNF41	1406.412857	2248.176408	-0.676956119	9.03E-08	1.23E-06
ENSG00000064651	SLC12A2	3011.479239	5516.562201	-0.873121581	9.05E-08	1.24E-06
ENSG00000110680	CALCA	5.078076603	39.77163644	-2.968519752	9.09E-08	1.24E-06
ENSG00000148468	FAM171A1	2139.133111	1200.87215	0.83226443	9.28E-08	1.27E-06
ENSG00000163364	LINC01116	53.55930522	11.89759155	2.178160872	9.42E-08	1.28E-06
ENSG00000148180	GSN	4882.981207	3123.67549	0.644712856	9.42E-08	1.28E-06
ENSG00000107738	VSIR	56.90499472	138.5479978	-1.283464254	9.61E-08	1.31E-06
ENSG00000086544	ITPKC	837.2203712	1259.811451	-0.589296914	9.61E-08	1.31E-06
ENSG00000026950	BTN3A1	335.5242287	577.0928527	-0.782529023	9.61E-08	1.31E-06
ENSG00000020181	ADGRA2	149.3368707	281.5563674	-0.912996703	1.02E-07	1.39E-06
ENSG00000162878	PKDCC	69.419196	176.1427202	-1.339436044	1.03E-07	1.40E-06
ENSG00000162490	DRAXIN	78.98932324	18.83553838	2.074025519	1.04E-07	1.41E-06
ENSG00000151292	CSNK1G3	971.6079456	1428.669973	-0.556251865	1.05E-07	1.43E-06
ENSG00000116478	HDAC1	6229.316734	8981.037959	-0.52774974	1.05E-07	1.43E-06
ENSG00000242028	HYPK	146.0628456	64.65497051	1.179184176	1.06E-07	1.44E-06

ENSG00000185989	RASA3	106.5928304	232.6957994	-1.126666197	1.06E-07	1.44E-06
ENSG00000172828	CES3	58.36402937	145.6312129	-1.315704842	1.07E-07	1.45E-06
ENSG00000164074	ABHD18	504.6698349	800.6635375	-0.665777441	1.09E-07	1.47E-06
ENSG00000176845	METRNL	1233.618136	2050.830336	-0.733222444	1.09E-07	1.48E-06
ENSG00000149091	DGKZ	3692.063283	2549.423768	0.534037896	1.10E-07	1.49E-06
ENSG00000259820	AC083843.2	422.8712159	180.578558	1.227249861	1.11E-07	1.50E-06
ENSG00000112983	BRD8	2050.340193	2947.941021	-0.524023205	1.13E-07	1.52E-06
ENSG00000121210	TMEM131L	818.6312482	1249.909766	-0.610372437	1.13E-07	1.52E-06
ENSG00000175727	MLXIP	2991.791881	4202.236887	-0.490198035	1.13E-07	1.53E-06
ENSG00000137154	RPS6	25735.65899	36430.76898	-0.501390474	1.14E-07	1.53E-06
ENSG00000109458	GAB1	309.3361712	588.4662605	-0.926608923	1.15E-07	1.55E-06
ENSG00000108417	KRT37	36.83480481	0.600305139	5.931652485	1.16E-07	1.57E-06
ENSG00000175198	PCCA	561.6384284	933.8012318	-0.732417334	1.17E-07	1.57E-06
ENSG00000196305	IARS	11892.52439	17139.62119	-0.527278446	1.19E-07	1.61E-06
ENSG00000108312	UBTF	4451.210918	6381.129636	-0.519468199	1.20E-07	1.62E-06
ENSG00000084764	MAPRE3	973.3525275	569.4105569	0.773643155	1.21E-07	1.63E-06
ENSG00000232677	LINC00665	71.52022733	10.62276392	2.769627317	1.24E-07	1.66E-06
ENSG00000168539	CHRM1	63.83183768	164.637714	-1.363072965	1.24E-07	1.67E-06
ENSG00000196924	FLNA	77481.02931	42141.3381	0.878585478	1.25E-07	1.68E-06
ENSG00000196544	BORCS6	212.5271912	367.2648727	-0.788938154	1.26E-07	1.69E-06
ENSG00000147642	SYBU	412.949265	667.6774573	-0.69240583	1.28E-07	1.71E-06
ENSG00000103534	TMC5	460.9759127	718.205347	-0.640158854	1.30E-07	1.73E-06
ENSG00000124193	SRSF6	5252.126288	8027.476521	-0.611925755	1.31E-07	1.75E-06
ENSG00000140988	RPS2	61808.80552	89825.70635	-0.539324296	1.32E-07	1.76E-06
ENSG00000135541	AHI1	454.0270262	703.4234189	-0.631754036	1.32E-07	1.77E-06
ENSG00000116584	ARHGEF2	3781.302825	2474.188352	0.611805552	1.33E-07	1.78E-06
ENSG00000170871	KIAA0232	3432.373212	2454.236417	0.484038714	1.35E-07	1.81E-06
ENSG00000141527	CARD14	109.4064154	254.3158012	-1.215057215	1.36E-07	1.81E-06
ENSG00000163644	PPM1K	706.5591798	1072.830808	-0.602351334	1.36E-07	1.81E-06
ENSG00000165424	ZCCHC24	441.8472139	216.2625236	1.027670946	1.36E-07	1.82E-06
ENSG00000196267	ZNF836	1.831640139	35.80720161	-4.294376084	1.37E-07	1.83E-06
ENSG00000165868	HSPA12A	1602.422806	1080.098876	0.569480344	1.38E-07	1.84E-06
ENSG00000182179	UBA7	43.55837578	157.6696037	-1.852676556	1.39E-07	1.85E-06
ENSG00000264012	AC091588.1	7.69916605	53.16361961	-2.791446909	1.40E-07	1.86E-06
ENSG00000113916	BCL6	483.2904486	276.4896836	0.806459939	1.40E-07	1.86E-06
ENSG00000135740	SLC9A5	564.6337102	316.752358	0.836213255	1.40E-07	1.87E-06
ENSG00000166401	SERPINB8	456.783208	250.2140196	0.868130215	1.42E-07	1.88E-06
ENSG00000146386	ABRACL	644.7622542	1070.25194	-0.731985711	1.44E-07	1.91E-06
ENSG00000134285	FKBP11	526.8074653	869.8243783	-0.723884117	1.45E-07	1.92E-06
ENSG00000198853	RUSC2	700.6975467	422.0513832	0.731458352	1.45E-07	1.92E-06
ENSG00000213190	MLLT11	370.7766644	206.6914015	0.840965294	1.50E-07	1.98E-06
ENSG00000117519	CNN3	7247.660936	11333.65685	-0.644986118	1.51E-07	2.00E-06
ENSG00000101255	TRIB3	4862.566594	8579.661822	-0.819205329	1.52E-07	2.01E-06

ENSG00000010361	FUZ	26.59144492	90.16749616	-1.762076743	1.53E-07	2.02E-06
ENSG00000127561	SYNGR3	69.50481876	169.135599	-1.281323965	1.53E-07	2.02E-06
ENSG00000272734	ADIRF-AS1	3151.989774	1994.34374	0.660716935	1.53E-07	2.03E-06
ENSG00000223572	CKMT1A	50.44066646	136.0677518	-1.434389677	1.54E-07	2.04E-06
ENSG00000184454	NCMAP	156.6490076	70.21886843	1.162101425	1.56E-07	2.06E-06
ENSG00000227308	AC009502.1	84.70004147	23.54373563	1.833554122	1.56E-07	2.06E-06
ENSG00000223784	LINP1	41.91827522	5.194505427	2.978168883	1.59E-07	2.10E-06
ENSG00000091527	CDV3	5951.522205	8263.908435	-0.473603578	1.61E-07	2.12E-06
ENSG00000254827	SLC22A18AS	45.27411321	117.6874325	-1.377166254	1.65E-07	2.17E-06
ENSG00000164104	HMGB2	8148.541087	14497.99045	-0.831294461	1.65E-07	2.17E-06
ENSG00000261167	AC107027.3	27.75207839	86.58833715	-1.645059082	1.66E-07	2.19E-06
ENSG00000109956	B3GAT1	58.89535456	15.2346586	1.957918953	1.67E-07	2.19E-06
ENSG00000122863	CHST3	583.5878189	955.5849919	-0.711552443	1.69E-07	2.22E-06
ENSG00000147454	SLC25A37	7190.528927	4921.018147	0.547296093	1.69E-07	2.22E-06
ENSG00000172893	DHCR7	7772.791525	5201.866703	0.579241455	1.72E-07	2.25E-06
ENSG00000214226	C17orf67	253.6403122	127.5263027	0.98818592	1.73E-07	2.27E-06
ENSG00000163710	PCOLCE2	115.9552591	39.88322299	1.529620197	1.75E-07	2.29E-06
ENSG00000142686	C1orf216	1367.107306	896.2740675	0.608372833	1.77E-07	2.32E-06
ENSG00000165929	TC2N	1170.679293	695.7305701	0.749868341	1.79E-07	2.35E-06
ENSG00000029993	HMGB3	2372.647868	3592.166187	-0.598462877	1.80E-07	2.36E-06
ENSG00000207926	MIR135A1	15.89350407	72.43831146	-2.18230456	1.81E-07	2.37E-06
ENSG00000109066	TMEM104	787.8746872	493.0160199	0.674987135	1.82E-07	2.38E-06
ENSG00000225438	KRT41P	30.97124015	0.994779868	5.076382426	1.82E-07	2.38E-06
ENSG00000180644	PRF1	906.4756641	378.2156587	1.259454982	1.82E-07	2.38E-06
ENSG00000205885	C1RL-AS1	988.4944654	608.74394	0.700497552	1.82E-07	2.38E-06
ENSG00000173295	FAM86B3P	647.3362739	400.680652	0.692905231	1.86E-07	2.43E-06
ENSG00000227331	AC005042.1	45.19055383	8.308047677	2.429938899	1.86E-07	2.43E-06
ENSG00000177697	CD151	3816.214197	5661.298854	-0.568986979	1.87E-07	2.44E-06
ENSG00000181163	NPM1	27871.60081	39913.96987	-0.518120161	1.89E-07	2.46E-06
ENSG00000140968	IRF8	0	27.19709926	-7.078653885	1.91E-07	2.49E-06
ENSG00000106404	CLDN15	557.1902909	348.5929747	0.677183616	1.94E-07	2.53E-06
ENSG00000197696	NMB	108.8673303	248.2066487	-1.189559296	1.95E-07	2.54E-06
ENSG00000137103	TMEM8B	1604.226535	1006.118158	0.673158777	2.05E-07	2.67E-06
ENSG00000135094	SDS	81.89661614	230.2900056	-1.493092554	2.05E-07	2.67E-06
ENSG00000116560	SFPQ	13579.52832	19590.55068	-0.528701342	2.08E-07	2.70E-06
ENSG00000237441	RGL2	1667.36712	2902.751897	-0.799500429	2.12E-07	2.75E-06
ENSG00000132199	ENOSF1	1932.184159	3024.719073	-0.646331517	2.12E-07	2.76E-06
ENSG00000182742	HOXB4	3311.846705	1643.636418	1.011252958	2.20E-07	2.85E-06
ENSG00000197123	ZNF679	21.77926357	0	7.028688961	2.20E-07	2.85E-06
ENSG00000040933	INPP4A	1229.565278	1845.718871	-0.586126667	2.20E-07	2.85E-06
ENSG00000159958	TNFRSF13C	100.1128085	200.1547962	-0.999305647	2.24E-07	2.90E-06
ENSG00000105639	JAK3	17.82931452	72.51553857	-2.026505186	2.24E-07	2.90E-06
ENSG00000141198	TOM1L1	1787.570536	2487.590827	-0.476787786	2.24E-07	2.91E-06

ENSG00000224880	MTCYBP29	20.5036834	0	6.939567698	2.25E-07	2.91E-06
ENSG00000006062	MAP3K14	943.1580541	628.5329279	0.585100964	2.26E-07	2.92E-06
ENSG00000206418	RAB12	879.2012726	535.0525214	0.7149982	2.29E-07	2.96E-06
ENSG00000138798	EGF	34.08623161	3.242744896	3.37269603	2.30E-07	2.96E-06
ENSG00000115266	APC2	801.7225252	459.4826603	0.804669886	2.35E-07	3.03E-06
ENSG00000034693	PEX3	255.4261526	513.5555461	-1.008541136	2.35E-07	3.03E-06
ENSG00000189157	FAM47E	131.0683438	307.9780159	-1.231384588	2.36E-07	3.04E-06
ENSG00000124067	SLC12A4	2267.376925	1533.981989	0.564145947	2.36E-07	3.05E-06
ENSG00000141985	SH3GL1	3487.379839	2491.99267	0.48476781	2.39E-07	3.08E-06
ENSG00000132837	DMGDH	44.17578906	156.8256161	-1.828119757	2.42E-07	3.11E-06
ENSG00000196268	ZNF493	744.6762539	437.5867412	0.768439495	2.43E-07	3.13E-06
ENSG00000229036	VDAC1P8	98.76809263	206.9188744	-1.069798791	2.47E-07	3.18E-06
ENSG00000178597	PSAPL1	2.17427964	35.02760766	-4.002203082	2.48E-07	3.19E-06
ENSG00000060762	MPC1	264.1532147	565.1258131	-1.098845046	2.48E-07	3.19E-06
ENSG00000165672	PRDX3	4837.684103	7519.09616	-0.636339072	2.50E-07	3.21E-06
ENSG00000169548	ZNF280A	7.539025016	48.77087165	-2.683324168	2.52E-07	3.23E-06
ENSG00000139597	N4BP2L1	106.247337	219.3763921	-1.045976099	2.58E-07	3.31E-06
ENSG00000148690	FRA10AC1	792.4660275	1193.064064	-0.590666831	2.63E-07	3.37E-06
ENSG00000128536	CDHR3	207.9769439	97.13395226	1.103515369	2.69E-07	3.44E-06
ENSG00000091428	RAPGEF4	5.806323963	43.40833502	-2.897256683	2.69E-07	3.45E-06
ENSG00000197702	PARVA	1829.765143	2710.484774	-0.566572764	2.69E-07	3.45E-06
ENSG00000008710	PKD1	4566.681572	7863.761718	-0.783972362	2.70E-07	3.45E-06
ENSG00000182264	IZUMO1	3077.44006	1958.298416	0.651986308	2.70E-07	3.45E-06
ENSG00000166348	USP54	1047.787006	1541.380001	-0.556503044	2.72E-07	3.47E-06
ENSG00000174171	AC020659.1	12.26399968	65.97877666	-2.419671292	2.72E-07	3.48E-06
ENSG00000124920	MYRF	580.4519026	948.4691232	-0.707434873	2.74E-07	3.50E-06
ENSG00000113369	ARRDC3	1618.344538	1122.439216	0.527846426	2.78E-07	3.54E-06
ENSG00000182674	KCNB2	19.19195001	0	6.846861501	2.80E-07	3.57E-06
ENSG00000005187	ACSM3	76.88750141	172.2187426	-1.164568876	2.81E-07	3.59E-06
ENSG00000159184	HOXB13	348.5279167	559.6806423	-0.683162195	2.82E-07	3.59E-06
ENSG00000141456	PELP1	2534.097137	4092.791259	-0.691820556	2.86E-07	3.64E-06
ENSG00000063176	SPHK2	716.2983378	1072.856892	-0.583294192	2.87E-07	3.65E-06
ENSG00000167384	ZNF180	479.7887448	275.4799715	0.802174345	2.90E-07	3.69E-06
ENSG00000171680	PLEKHG5	1612.717564	2621.315901	-0.700335255	2.94E-07	3.74E-06
ENSG00000260588	AC027702.1	37.91869403	5.106541752	2.88760328	2.95E-07	3.75E-06
ENSG00000064687	ABCA7	4063.139643	2536.970431	0.679729673	2.96E-07	3.76E-06
ENSG00000235169	SMIM1	106.8349581	40.47167117	1.406198861	3.00E-07	3.81E-06
ENSG00000254815	AP006284.1	67.63687403	238.3244028	-1.814152379	3.03E-07	3.84E-06
ENSG00000183111	ARHGEF37	601.6698637	366.3744482	0.715025876	3.04E-07	3.85E-06
ENSG00000196684	HSH2D	804.6863831	1225.820266	-0.607839985	3.04E-07	3.86E-06
ENSG00000118276	B4GALT6	621.5640817	391.3663718	0.666186909	3.05E-07	3.87E-06
ENSG00000137693	YAP1	9134.402767	6638.678239	0.460363438	3.09E-07	3.91E-06
ENSG00000107104	KANK1	1776.300222	2532.966572	-0.51185641	3.14E-07	3.97E-06

ENSG00000205632	LINC01310	18.89751009	0	6.823623589	3.15E-07	3.98E-06
ENSG00000110344	UBE4A	1685.081014	2417.098803	-0.520295458	3.15E-07	3.98E-06
ENSG00000100373	UPK3A	9.450100691	52.37810992	-2.469014379	3.16E-07	3.99E-06
ENSG00000161091	MFSD12	5371.609323	7755.395097	-0.529820273	3.16E-07	3.99E-06
ENSG00000112584	FAM120B	841.6997446	1287.243972	-0.612279903	3.16E-07	3.99E-06
ENSG00000148426	PROSER2	1640.705712	2455.912717	-0.582008784	3.16E-07	3.99E-06
ENSG00000142552	RCN3	1.843372544	32.20589182	-4.144368215	3.18E-07	4.00E-06
ENSG00000203857	HSD3B1	2.966268874	39.16540283	-3.740463435	3.18E-07	4.00E-06
ENSG00000122335	SERAC1	415.5457823	767.7825105	-0.886650867	3.19E-07	4.02E-06
ENSG00000145832	SLC25A48	68.26998098	19.16077319	1.82896429	3.24E-07	4.08E-06
ENSG00000160606	TLCD1	279.1543691	481.8877158	-0.789154629	3.27E-07	4.11E-06
ENSG00000261104	AC093904.4	180.5020166	82.42452143	1.127749234	3.28E-07	4.13E-06
ENSG00000143127	ITGA10	116.9763625	46.46002519	1.334852791	3.36E-07	4.22E-06
ENSG00000163682	RPL9	13288.41971	18646.55915	-0.488731867	3.36E-07	4.23E-06
ENSG00000275880	AL139385.1	95.34486546	32.00941692	1.581260303	3.37E-07	4.23E-06
ENSG00000188910	GJB3	1076.548977	468.5854304	1.198532206	3.38E-07	4.25E-06
ENSG00000164062	APEH	7736.149266	4424.21095	0.805981525	3.39E-07	4.26E-06
ENSG00000108588	CCDC47	3680.817942	5113.174325	-0.474345985	3.42E-07	4.29E-06
ENSG00000120885	CLU	11492.74633	3433.750998	1.742771606	3.44E-07	4.31E-06
ENSG00000109971	HSPA8	38539.67464	27989.28695	0.461450212	3.46E-07	4.33E-06
ENSG00000146250	PRSS35	7.852968488	59.75665685	-2.912238206	3.47E-07	4.35E-06
ENSG00000130813	C19orf66	400.3283423	656.8687583	-0.714179507	3.49E-07	4.37E-06
ENSG00000257337	AC068888.1	488.5917802	777.3263739	-0.669474118	3.53E-07	4.41E-06
ENSG00000225792	AC004540.2	65.35984542	17.64867443	1.896489847	3.59E-07	4.49E-06
ENSG00000133135	RNF128	32.20639189	2.567271354	3.710168406	3.59E-07	4.49E-06
ENSG00000107669	ATE1	1209.27547	1829.410314	-0.597609751	3.60E-07	4.49E-06
ENSG00000089876	DHX32	1751.17145	1188.153868	0.559002627	3.66E-07	4.57E-06
ENSG00000231185	SPRY4-AS1	92.38097393	34.11882379	1.437263378	3.67E-07	4.58E-06
ENSG00000267279	AC090409.1	31.26568007	3.242744896	3.246717143	3.68E-07	4.58E-06
ENSG00000160469	BRSK1	91.22827419	212.3296336	-1.21591466	3.70E-07	4.61E-06
ENSG00000136444	RSAD1	569.9091855	877.09732	-0.621791447	3.71E-07	4.62E-06
ENSG00000256268	LINC02454	33.70047238	3.700101066	3.208441566	3.77E-07	4.69E-06
ENSG00000184378	ACTRT3	48.14255037	118.1389385	-1.29775511	3.79E-07	4.71E-06
ENSG00000231252	AC099792.1	80.15883524	17.49044148	2.213282974	3.83E-07	4.76E-06
ENSG00000237187	NR2F1-AS1	100.1621154	35.95271759	1.474119642	3.88E-07	4.82E-06
ENSG00000153294	ADGRF4	39.56260449	119.9707081	-1.604325214	3.92E-07	4.87E-06
ENSG00000005884	ITGA3	13959.95121	9386.408048	0.572633853	3.92E-07	4.87E-06
ENSG00000165312	OTUD1	529.7438971	843.7652704	-0.670969488	4.01E-07	4.98E-06
ENSG00000152684	PELO	1241.272276	705.3709576	0.814079158	4.07E-07	5.05E-06
ENSG00000142227	EMP3	203.675802	88.69920339	1.200497347	4.08E-07	5.06E-06
ENSG00000130222	GADD45G	97.70402091	38.18195887	1.358444785	4.10E-07	5.08E-06
ENSG00000135114	OASL	1.838141323	29.96041551	-4.042515959	4.11E-07	5.09E-06
ENSG00000280042	AC022336.3	55.33497463	13.51527008	2.045570166	4.13E-07	5.11E-06

ENSG00000213057	C1orf220	24.27002661	76.66947774	-1.655138744	4.18E-07	5.17E-06
ENSG00000109686	SH3D19	2921.975844	4119.619289	-0.495434356	4.20E-07	5.20E-06
ENSG00000172469	MANEA	428.0779032	712.1602495	-0.73451357	4.21E-07	5.20E-06
ENSG00000122643	NT5C3A	597.7857199	921.1279285	-0.624484228	4.22E-07	5.21E-06
ENSG00000210164	MT-TG	12.67957353	54.01159348	-2.091660821	4.25E-07	5.25E-06
ENSG00000123268	ATF1	939.6560335	1378.843122	-0.553612068	4.29E-07	5.29E-06
ENSG00000109475	RPL34	8464.706235	11856.70362	-0.486147193	4.30E-07	5.30E-06
ENSG00000213976	AC010615.1	169.0140098	75.19080011	1.170028389	4.34E-07	5.35E-06
ENSG00000013374	NUB1	1648.612767	2319.177791	-0.492164518	4.34E-07	5.35E-06
ENSG00000245680	ZNF585B	1139.775243	645.3338621	0.821803889	4.39E-07	5.40E-06
ENSG00000142549	IGLON5	53.18019854	11.99681283	2.161006364	4.40E-07	5.42E-06
ENSG00000272899	ATP6V1FNB	159.4484717	285.6273987	-0.839913402	4.40E-07	5.42E-06
ENSG00000124243	BCAS4	379.6740243	623.6711234	-0.71633531	4.42E-07	5.44E-06
ENSG00000171552	BCL2L1	5724.359176	3849.667636	0.572255307	4.48E-07	5.51E-06
ENSG00000196208	GREB1	117.2540013	42.80468116	1.447160338	4.51E-07	5.54E-06
ENSG00000184471	C1QTNF8	35.4878742	4.954745585	2.864258368	4.51E-07	5.55E-06
ENSG00000117408	IPO13	1248.839204	866.3772241	0.527515085	4.53E-07	5.56E-06
ENSG00000234311	AL451069.3	3.901749472	38.54250416	-3.279868194	4.55E-07	5.58E-06
ENSG00000172840	PDP2	1177.595463	1882.24156	-0.676978628	4.62E-07	5.67E-06
ENSG00000198131	ZNF544	1368.33645	908.7841604	0.590973106	4.64E-07	5.68E-06
ENSG00000003096	KLHL13	13.45205921	57.66899621	-2.104192197	4.65E-07	5.69E-06
ENSG00000245164	LINC00861	60.50548913	13.99521977	2.130510358	4.70E-07	5.76E-06
ENSG00000119866	BCL11A	480.564889	297.3904712	0.691596838	4.74E-07	5.80E-06
ENSG00000158486	DNAH3	600.9003974	333.5663749	0.85129178	4.80E-07	5.87E-06
ENSG00000147604	RPL7	25108.95287	35252.63953	-0.489519591	4.82E-07	5.89E-06
ENSG00000155189	AGPAT5	1581.361339	2502.288975	-0.66242785	4.89E-07	5.97E-06
ENSG00000198221	AFDN-DT	24.68163921	82.23871753	-1.734707418	4.89E-07	5.98E-06
ENSG00000091592	NLRP1	39.16684814	6.679033237	2.557556716	4.90E-07	5.98E-06
ENSG00000109501	WFS1	2071.244695	1380.847168	0.584744484	5.08E-07	6.20E-06
ENSG00000102760	RGCC	58.23100045	16.36748831	1.833942817	5.09E-07	6.21E-06
ENSG00000164199	ADGRV1	901.4918813	587.0437923	0.618706568	5.09E-07	6.21E-06
ENSG00000129116	PALLD	1242.915348	706.0995799	0.814550218	5.13E-07	6.25E-06
ENSG00000175592	FOSL1	1582.294484	306.4229194	2.367591902	5.15E-07	6.27E-06
ENSG00000197279	ZNF165	412.6759126	728.0867586	-0.82013368	5.21E-07	6.35E-06
ENSG00000068831	RASGRP2	49.82832181	118.5932977	-1.251717327	5.23E-07	6.36E-06
ENSG00000280780	JAKMIP2-AS1	42.14341687	4.675727177	3.14618829	5.38E-07	6.54E-06
ENSG00000204380	PKP4-AS1	85.52183411	24.83909808	1.791014317	5.53E-07	6.72E-06
ENSG00000205352	PRR13	1053.811354	1645.34386	-0.643058404	5.55E-07	6.74E-06
ENSG00000196387	ZNF140	680.3016697	383.123199	0.830495237	5.57E-07	6.76E-06
ENSG00000078902	TOLLIP	1089.170312	1547.797945	-0.506905512	5.59E-07	6.79E-06
ENSG00000183496	MEX3B	221.7063168	118.2936534	0.907557466	5.60E-07	6.79E-06
ENSG00000120438	TCP1	4595.076993	8948.940676	-0.961730291	5.62E-07	6.81E-06
ENSG00000131323	TRAF3	3146.282808	2171.037042	0.534901152	5.65E-07	6.85E-06

ENSG00000240342	RPS2P5	6108.556694	9844.958234	-0.68861684	5.66E-07	6.85E-06
ENSG00000135424	ITGA7	678.6249393	410.2379113	0.728121351	5.67E-07	6.87E-06
ENSG00000221947	XKR9	78.42185897	24.53602679	1.670698957	5.74E-07	6.94E-06
ENSG00000086598	TMED2	7260.353692	10277.83044	-0.501459783	5.75E-07	6.95E-06
ENSG00000100196	KDELR3	1488.541651	980.3099873	0.601857435	5.83E-07	7.05E-06
ENSG00000134900	TPP2	2814.429956	3969.271463	-0.496146711	5.83E-07	7.05E-06
ENSG00000250033	SLC7A11-AS1	141.4442672	61.50114034	1.202731547	5.91E-07	7.14E-06
ENSG00000086289	EPDR1	2733.675052	1933.132875	0.499653224	5.92E-07	7.15E-06
ENSG00000196867	ZFP28	30.3942553	0.631745859	5.649816199	5.93E-07	7.15E-06
ENSG00000166803	PCLAF	568.0242942	1026.847163	-0.855056581	5.93E-07	7.15E-06
ENSG00000075290	WNT8B	117.4119164	49.74013928	1.236412407	5.95E-07	7.17E-06
ENSG00000129493	HEATR5A	270.7647041	448.2600738	-0.727388845	5.97E-07	7.20E-06
ENSG00000185046	ANKS1B	45.87599542	6.95315252	2.756323963	6.05E-07	7.28E-06
ENSG00000261863	LINC01996	0.373875456	33.33769218	-6.413095772	6.06E-07	7.30E-06
ENSG00000166863	TAC3	2.523420324	33.62015035	-3.725813101	6.07E-07	7.30E-06
ENSG00000239887	C1orf226	1055.153227	722.5845437	0.546812246	6.17E-07	7.41E-06
ENSG00000087510	TFAP2C	498.3246108	885.018621	-0.8289336	6.24E-07	7.50E-06
ENSG00000267385	AC011498.4	3.1917357	41.33571074	-3.67104366	6.27E-07	7.53E-06
ENSG00000129534	MIS18BP1	461.8145064	878.7086687	-0.928698172	6.27E-07	7.53E-06
ENSG00000278540	ACACA	5162.578786	8098.326668	-0.649642139	6.28E-07	7.53E-06
ENSG00000112357	PEX7	171.4770074	339.7748919	-0.987714238	6.29E-07	7.55E-06
ENSG00000159720	ATP6V0D1	2885.673769	2005.624464	0.524469724	6.32E-07	7.58E-06
ENSG00000143842	SOX13	685.6312066	1116.711574	-0.702860262	6.33E-07	7.58E-06
ENSG00000108799	EZH1	1042.203446	1617.017356	-0.633197203	6.35E-07	7.60E-06
ENSG00000161395	PGAP3	852.5668564	1601.000001	-0.908518165	6.36E-07	7.61E-06
ENSG00000145198	VWA5B2	500.713721	316.4317617	0.662435813	6.39E-07	7.64E-06
ENSG00000242282	AC108488.1	153.2719196	71.23581182	1.103199109	6.46E-07	7.73E-06
ENSG00000256894	AC022509.3	37.57367719	4.64823453	2.98632845	6.49E-07	7.75E-06
ENSG00000166887	VPS39	2357.862335	3228.287512	-0.453311745	6.51E-07	7.78E-06
ENSG00000217801	AL390719.1	1070.331963	642.3132737	0.737804608	6.53E-07	7.79E-06
ENSG00000065833	ME1	6453.530458	4621.092082	0.481707	6.60E-07	7.87E-06
ENSG00000231537	MTCO3P10	17.92556231	0	6.745841512	6.65E-07	7.93E-06
ENSG00000164484	TMEM200A	4.398028823	35.51451518	-3.022388054	6.66E-07	7.94E-06
ENSG00000107968	MAP3K8	464.7895107	728.8130876	-0.64801158	6.75E-07	8.05E-06
ENSG00000198951	NAGA	1185.715793	1875.178281	-0.661486263	6.79E-07	8.09E-06
ENSG00000197442	MAP3K5	147.4061289	281.2083567	-0.933505805	6.80E-07	8.10E-06
ENSG00000111371	SLC38A1	11484.25006	15461.67947	-0.429038547	6.80E-07	8.10E-06
ENSG00000106025	TSPAN12	198.0755956	422.9517629	-1.093329441	6.86E-07	8.15E-06
ENSG00000026652	AGPAT4	202.5157993	350.5953953	-0.790742051	6.86E-07	8.15E-06
ENSG00000110906	KCTD10	2213.748756	1572.022979	0.493634464	6.90E-07	8.20E-06
ENSG00000171492	LRRC8D	933.6895572	1406.862628	-0.591969848	6.95E-07	8.26E-06
ENSG00000110799	VWF	54.35177099	9.126978497	2.561575612	7.07E-07	8.39E-06
ENSG00000175787	ZNF169	231.1170964	478.7851838	-1.049862074	7.11E-07	8.44E-06

ENSG00000241878	PISD	1263.99937	1998.859745	-0.661581308	7.14E-07	8.47E-06
ENSG00000122952	ZWINT	2379.971914	3510.34619	-0.560871617	7.18E-07	8.51E-06
ENSG00000225313	AL513327.1	130.1457142	55.16847607	1.234418373	7.20E-07	8.53E-06
ENSG00000215712	TMEM242	249.4377842	406.88685	-0.706486265	7.21E-07	8.53E-06
ENSG00000162302	RPS6KA4	2320.557581	1372.413112	0.757117907	7.24E-07	8.56E-06
ENSG00000185262	UBALD2	1866.79865	2940.120583	-0.655477454	7.27E-07	8.60E-06
ENSG00000157796	WDR19	613.7610481	902.7101618	-0.556923826	7.34E-07	8.68E-06
ENSG00000110880	CORO1C	10526.31438	6047.692197	0.799397022	7.34E-07	8.68E-06
ENSG00000257732	AC089983.1	38.24722379	5.894031851	2.681118158	7.39E-07	8.73E-06
ENSG00000143217	NECTIN4	223.9666957	398.8437612	-0.831680953	7.41E-07	8.76E-06
ENSG00000138744	NAAA	428.0832746	663.4820312	-0.633027444	7.43E-07	8.77E-06
ENSG00000145781	COMMD10	735.4059462	488.1690952	0.591358193	7.48E-07	8.83E-06
ENSG00000146410	MTFR2	125.8819842	323.5320987	-1.363931618	7.51E-07	8.85E-06
ENSG00000278974	AC093909.6	2.536422691	29.86119422	-3.557458217	7.52E-07	8.86E-06
ENSG00000231683	AL033397.1	17.75638017	0	6.734481593	7.60E-07	8.96E-06
ENSG00000165983	PTER	1445.475442	2043.483207	-0.499289926	7.64E-07	9.00E-06
ENSG00000137841	PLCB2	13.38435608	63.85072433	-2.246698556	7.85E-07	9.23E-06
ENSG00000179912	R3HDM2	1118.465169	1706.148846	-0.608743221	7.85E-07	9.24E-06
ENSG00000107021	TBC1D13	1238.975228	803.8783196	0.623892013	7.89E-07	9.28E-06
ENSG00000196705	ZNF431	1087.649144	733.4778704	0.56817047	7.93E-07	9.32E-06
ENSG00000104408	EIF3E	9891.356451	13682.6746	-0.468059546	8.13E-07	9.55E-06
ENSG00000196277	GRM7	49.71479643	6.707034193	2.925955586	8.24E-07	9.68E-06
ENSG00000253368	TRNP1	837.4491714	481.5880187	0.796705298	8.31E-07	9.75E-06
ENSG00000090097	PCBP4	1834.214705	1280.783967	0.518109644	8.34E-07	9.78E-06
ENSG00000100503	NIN	1920.012631	1341.699175	0.516574675	8.37E-07	9.81E-06
ENSG00000213741	RPS29	4108.18516	6106.312678	-0.571798623	8.38E-07	9.81E-06
ENSG00000172992	DCAKD	1513.686551	2124.494669	-0.489025107	8.38E-07	9.81E-06
ENSG00000261221	ZNF865	2086.988728	1464.826045	0.510514088	8.42E-07	9.86E-06
ENSG00000087074	PPP1R15A	2154.726358	3360.105838	-0.641061724	8.46E-07	9.90E-06
ENSG00000244588	RAD21L1	29.50697428	0.663186579	5.597773974	8.47E-07	9.91E-06
ENSG00000100528	CNIH1	3039.72437	4200.032309	-0.466583438	8.53E-07	9.98E-06
ENSG00000197467	COL13A1	760.3732982	97.55538556	2.960345429	8.65E-07	1.01E-05
ENSG00000115107	STEAP3	732.1701348	1100.195767	-0.587443914	8.72E-07	1.02E-05
ENSG00000266145	RHOT1P1	47.95498327	10.76476183	2.169402989	8.75E-07	1.02E-05
ENSG00000182054	IDH2	2679.499298	4151.937775	-0.632004461	9.05E-07	1.06E-05
ENSG00000163191	S100A11	4236.212078	2328.743506	0.862843269	9.11E-07	1.06E-05
ENSG00000131650	KREMEN2	759.3197031	390.7772329	0.956234956	9.11E-07	1.06E-05
ENSG00000133318	RTN3	5180.49577	7119.086679	-0.458499569	9.12E-07	1.06E-05
ENSG00000135749	PCNX2	1684.872149	1187.107867	0.505073016	9.13E-07	1.06E-05
ENSG00000135697	BCO1	5.126276183	37.96831061	-2.897863815	9.16E-07	1.07E-05
ENSG00000084092	NOA1	1766.541523	2617.175758	-0.566928213	9.17E-07	1.07E-05
ENSG00000277363	SRCIN1	1430.20994	813.2663493	0.815579273	9.19E-07	1.07E-05
ENSG00000162244	RPL29	24591.46255	34434.34422	-0.485669775	9.26E-07	1.08E-05

ENSG00000225178	RPSAP58	935.6813272	1492.475404	-0.674321325	9.62E-07	1.12E-05
ENSG00000112972	HMGCS1	8380.84938	4967.533179	0.754411532	9.74E-07	1.13E-05
ENSG00000140105	WARS	3165.356396	5364.35619	-0.761033673	9.85E-07	1.14E-05
ENSG00000148218	ALAD	847.2899456	1309.228505	-0.627173157	9.88E-07	1.15E-05
ENSG00000272502	AC104958.2	133.9648573	53.85095011	1.312894915	9.89E-07	1.15E-05
ENSG00000166532	RIMKLB	22.27935281	90.30501223	-2.01782362	1.01E-06	1.17E-05
ENSG00000103363	ELOB	4139.356068	2827.235681	0.549749291	1.03E-06	1.19E-05
ENSG00000141738	GRB7	891.83552	1395.549813	-0.645845054	1.03E-06	1.19E-05
ENSG00000153012	LGI2	25.44762371	87.5228809	-1.785632625	1.03E-06	1.20E-05
ENSG00000163840	DTX3L	1317.806824	1867.340305	-0.502459663	1.05E-06	1.21E-05
ENSG00000197859	ADAMTSL2	139.3294401	49.10238664	1.511434795	1.05E-06	1.21E-05
ENSG00000134202	GSTM3	5116.475426	3270.456971	0.645466559	1.05E-06	1.21E-05
ENSG00000273079	GRIN2B	13.73349677	54.21690284	-1.975985522	1.05E-06	1.22E-05
ENSG00000214517	PPME1	2174.034609	1501.123464	0.533988187	1.06E-06	1.22E-05
ENSG00000285517	LINC00941	190.1423981	98.00889773	0.959205869	1.06E-06	1.22E-05
ENSG00000115350	POLE4	840.7147833	506.1310561	0.730860515	1.07E-06	1.24E-05
ENSG00000185220	PGBD2	497.3672269	307.6859416	0.69431642	1.07E-06	1.24E-05
ENSG00000140451	PIF1	528.0952432	922.3822484	-0.804958894	1.07E-06	1.24E-05
ENSG00000135406	PRPH	6.543612434	47.12709427	-2.850178655	1.08E-06	1.25E-05
ENSG00000174460	ZCCHC12	86.26197646	25.29456488	1.75738178	1.08E-06	1.25E-05
ENSG00000187699	C2orf88	37.24260751	104.1004211	-1.48436599	1.09E-06	1.25E-05
ENSG00000178814	OPLAH	791.1130469	1301.13813	-0.717299788	1.09E-06	1.25E-05
ENSG00000137309	HMGA1	15860.48912	23046.85155	-0.539157076	1.11E-06	1.28E-05
ENSG00000251728	RF00019	63.03922053	16.63765952	1.933888099	1.12E-06	1.28E-05
ENSG00000087191	PSMC5	1731.595022	2588.753047	-0.580487976	1.12E-06	1.29E-05
ENSG00000146858	ZC3HAV1L	988.7925609	676.7701018	0.547100333	1.12E-06	1.29E-05
ENSG00000161328	LRRC56	88.53408781	213.4240647	-1.267063926	1.13E-06	1.30E-05
ENSG00000128789	PSMG2	1311.789295	1832.084795	-0.48169001	1.14E-06	1.31E-05
ENSG00000198832	SELENOM	175.2626916	311.2421554	-0.827943116	1.15E-06	1.32E-05
ENSG00000204385	SLC44A4	7.247125022	40.94226536	-2.494908485	1.17E-06	1.34E-05
ENSG00000148943	LIN7C	1283.997149	1939.301011	-0.595142138	1.18E-06	1.35E-05
ENSG00000205089	CCNI2	56.41886386	125.9537202	-1.158728396	1.18E-06	1.35E-05
ENSG00000167693	NXN	3666.866107	2409.371607	0.605569677	1.18E-06	1.35E-05
ENSG00000166272	WBP1L	2330.683221	1638.837925	0.508070242	1.19E-06	1.36E-05
ENSG00000112110	MRPL18	674.2059969	1228.548951	-0.86616926	1.19E-06	1.36E-05
ENSG00000130779	CLIP1	3616.509998	5134.033702	-0.505646726	1.20E-06	1.37E-05
ENSG00000261514	LINC01976	26.6369532	2.141355904	3.64988923	1.22E-06	1.39E-05
ENSG00000175197	DDIT3	424.1470959	844.4767034	-0.993080082	1.24E-06	1.42E-05
ENSG00000229320	KRT8P12	195.6021101	96.45505169	1.02201556	1.26E-06	1.43E-05
ENSG00000184497	TMEM255B	181.1882628	92.14733595	0.975832691	1.26E-06	1.43E-05
ENSG00000081026	MAGI3	2156.371264	1321.002135	0.706542417	1.27E-06	1.45E-05
ENSG00000244187	TMEM141	805.4820392	1208.436878	-0.584819159	1.28E-06	1.46E-05
ENSG00000138814	PPP3CA	1679.845288	1089.418419	0.623999504	1.28E-06	1.46E-05

ENSG00000162444	RBP7	0.747750911	32.39556549	-5.478017027	1.29E-06	1.47E-05
ENSG00000255171	LINC01499	15.99498309	0	6.582954417	1.32E-06	1.50E-05
ENSG00000113119	TMCO6	474.8023127	710.5747405	-0.581765731	1.32E-06	1.50E-05
ENSG00000171502	COL24A1	28.50633048	0.631745859	5.553729651	1.34E-06	1.52E-05
ENSG00000232759	AC002480.1	1.102122816	30.5224004	-4.8027788	1.34E-06	1.52E-05
ENSG00000167965	MLST8	1878.736655	2604.592388	-0.471569196	1.39E-06	1.58E-05
ENSG00000260265	LINC02562	138.2205022	48.85282855	1.509450432	1.44E-06	1.63E-05
ENSG00000128694	OSGEPL1	179.428601	336.416248	-0.90848076	1.44E-06	1.63E-05
ENSG00000176842	IRX5	786.0911095	488.4263674	0.687493741	1.45E-06	1.64E-05
ENSG00000147403	RPL10	27336.65177	39008.46674	-0.512934653	1.46E-06	1.65E-05
ENSG00000110665	C11orf21	48.260037	10.29760741	2.232210172	1.46E-06	1.65E-05
ENSG00000184185	KCNJ12	1.819907735	29.00094613	-3.994194222	1.46E-06	1.66E-05
ENSG00000173599	PC	1227.865246	1756.083558	-0.51602528	1.46E-06	1.66E-05
ENSG00000029639	TFB1M	233.345132	450.8970804	-0.951627894	1.46E-06	1.66E-05
ENSG00000119335	SET	21451.80977	31672.92502	-0.562184823	1.47E-06	1.66E-05
ENSG00000158169	FANCC	900.608895	1519.019235	-0.754599111	1.49E-06	1.69E-05
ENSG00000231412	AC005392.2	0	19.24127072	-6.579311066	1.50E-06	1.69E-05
ENSG00000203761	MSTO2P	199.0088502	342.2847151	-0.781365573	1.51E-06	1.71E-05
ENSG00000170425	ADORA2B	958.3098656	634.3396212	0.596018307	1.55E-06	1.75E-05
ENSG00000107779	BMPR1A	1676.838602	2373.260737	-0.501132988	1.56E-06	1.76E-05
ENSG00000163701	IL17RE	730.1475859	1070.014564	-0.551110183	1.56E-06	1.76E-05
ENSG00000133805	AMPD3	666.0203635	973.4465443	-0.54724033	1.57E-06	1.78E-05
ENSG00000167766	ZNF83	1392.661326	925.2778289	0.590846241	1.59E-06	1.79E-05
ENSG00000140548	ZNF710	985.1215432	1438.278791	-0.545740206	1.59E-06	1.80E-05
ENSG00000101384	JAG1	2230.375775	1593.836564	0.485119859	1.60E-06	1.80E-05
ENSG00000108950	FAM20A	140.9104021	48.74965919	1.538560794	1.60E-06	1.80E-05
ENSG00000167723	TRPV3	480.2831233	298.5036133	0.687630847	1.60E-06	1.81E-05
ENSG00000115844	DLX2	134.2510608	56.36908635	1.250440064	1.61E-06	1.81E-05
ENSG00000163697	APBB2	7442.206134	10247.65646	-0.461463228	1.62E-06	1.82E-05
ENSG00000102890	ELMO3	1138.784125	1676.99127	-0.557953253	1.62E-06	1.82E-05
ENSG00000105419	MEIS3	979.5900042	485.5880524	1.01424454	1.63E-06	1.83E-05
ENSG00000186007	LEMD1	43.82681096	8.831725052	2.294622724	1.65E-06	1.85E-05
ENSG00000178719	GRINA	4727.80942	3468.944416	0.446742308	1.66E-06	1.86E-05
ENSG00000184584	TMEM173	1058.753239	1698.990907	-0.681599962	1.66E-06	1.86E-05
ENSG00000090013	BLVRB	2759.173177	1854.932625	0.572557688	1.68E-06	1.89E-05
ENSG00000112576	CCND3	1531.010279	889.0059865	0.783185191	1.71E-06	1.91E-05
ENSG00000065618	COL17A1	124.2030279	52.4768229	1.245600122	1.71E-06	1.92E-05
ENSG00000153317	ASAP1	1654.29318	1069.126563	0.62907152	1.75E-06	1.96E-05
ENSG00000198393	ZNF26	726.8409147	378.6811466	0.942559624	1.76E-06	1.97E-05
ENSG00000120913	PDLIM2	1234.408068	727.3363917	0.762244036	1.76E-06	1.97E-05
ENSG00000109472	CPE	277.7142072	132.0224935	1.076750228	1.77E-06	1.97E-05
ENSG00000011009	LYPLA2	2366.847869	1600.084639	0.56432543	1.77E-06	1.98E-05
ENSG00000162889	MAPKAPK2	3322.801351	2166.061814	0.616935829	1.77E-06	1.98E-05

ENSG00000161016	RPL8	36432.29598	51599.4896	-0.502151357	1.78E-06	1.98E-05
ENSG00000102897	LYRM1	1066.410093	700.4716789	0.606818518	1.78E-06	1.98E-05
ENSG00000113391	FAM172A	1266.871488	839.2843366	0.594833609	1.78E-06	1.99E-05
ENSG00000117560	FASLG	26.44034499	2.364880709	3.463446374	1.79E-06	2.00E-05
ENSG00000105810	CDK6	2163.388843	2960.777988	-0.452758494	1.80E-06	2.00E-05
ENSG00000112773	TENT5A	112.6005285	229.5980494	-1.030220285	1.83E-06	2.03E-05
ENSG00000091972	CD200	27.04602587	0.631745859	5.477860577	1.83E-06	2.04E-05
ENSG00000082684	SEMA5B	7.227621471	41.79460457	-2.523257892	1.84E-06	2.05E-05
ENSG00000167552	TUBA1A	2508.223012	1504.787774	0.736530943	1.85E-06	2.05E-05
ENSG00000139163	ETNK1	2111.096534	3370.028365	-0.674493164	1.85E-06	2.06E-05
ENSG00000163295	ALPI	2.17427964	30.07286207	-3.781915903	1.85E-06	2.06E-05
ENSG00000083544	TDRD3	712.5505646	1033.410531	-0.536173211	1.86E-06	2.06E-05
ENSG00000072954	TMEM38A	746.9840136	486.9022293	0.618129227	1.87E-06	2.08E-05
ENSG00000237036	ZEB1-AS1	319.961329	194.7160745	0.716372502	1.87E-06	2.08E-05
ENSG00000188994	ZNF292	2448.87258	1649.431218	0.570089775	1.87E-06	2.08E-05
ENSG00000203780	FANK1	114.001529	47.21702562	1.274464776	1.89E-06	2.10E-05
ENSG00000177380	PPFIA3	536.5317006	343.7842535	0.641374981	1.90E-06	2.11E-05
ENSG00000159216	RUNX1	1800.80223	1212.715575	0.569850226	1.91E-06	2.12E-05
ENSG00000141540	TTYH2	33.22257799	97.82616915	-1.552763678	1.91E-06	2.12E-05
ENSG00000185567	AHNAK2	590.3037701	1217.335744	-1.043499867	1.92E-06	2.12E-05
ENSG00000169609	C15orf40	675.449203	418.3582657	0.692410083	1.93E-06	2.14E-05
ENSG00000162496	DHRS3	13.02871421	59.70116324	-2.188980659	1.95E-06	2.16E-05
ENSG00000125170	DOK4	1184.242954	1657.135271	-0.484456333	1.95E-06	2.16E-05
ENSG00000107554	DNMBP	3271.501189	2334.357114	0.486804699	1.97E-06	2.17E-05
ENSG00000198959	TGM2	4.422763594	34.22345251	-2.9623206	1.97E-06	2.18E-05
ENSG00000140044	JDP2	893.3476461	1480.198373	-0.728226431	2.01E-06	2.22E-05
ENSG00000198833	UBE2J1	2446.460487	3595.650612	-0.555432985	2.02E-06	2.23E-05
ENSG00000167797	CDK2AP2	1650.995724	2572.831379	-0.639859839	2.03E-06	2.23E-05
ENSG00000224769	MUC20P1	98.08835881	221.5936198	-1.173494522	2.04E-06	2.25E-05
ENSG00000006740	ARHGAP44	601.109551	922.724083	-0.618079667	2.08E-06	2.29E-05
ENSG00000224397	SMIM25	27.55420021	3.01922009	3.191154658	2.09E-06	2.30E-05
ENSG00000128709	HOXD9	510.6566081	318.2774341	0.680951178	2.11E-06	2.32E-05
ENSG00000155380	SLC16A1	4703.777986	6808.069889	-0.533561507	2.12E-06	2.34E-05
ENSG00000005471	ABCB4	4.000688559	30.94144905	-2.953628805	2.14E-06	2.35E-05
ENSG00000119401	TRIM32	1526.721618	1102.977405	0.468933901	2.16E-06	2.37E-05
ENSG00000279191	AC068491.4	100.7235692	32.71290415	1.612003017	2.19E-06	2.41E-05
ENSG00000162734	PEA15	1508.863467	901.3958802	0.742171669	2.21E-06	2.42E-05
ENSG00000134970	TMED7	2659.931241	3732.507419	-0.488745365	2.21E-06	2.43E-05
ENSG00000182199	SHMT2	7449.727797	10985.75126	-0.560389209	2.25E-06	2.47E-05
ENSG00000180884	ZNF792	294.9424441	172.5307394	0.774768676	2.26E-06	2.48E-05
ENSG00000126821	SGPP1	1304.476855	1811.64049	-0.473536671	2.30E-06	2.52E-05
ENSG00000059377	TBXAS1	59.64072814	16.52469192	1.862322646	2.31E-06	2.53E-05
ENSG00000213937	CLDN9	736.2750049	402.7809125	0.868436895	2.32E-06	2.54E-05

ENSG00000168913	ENHO	4.07362291	33.66936377	-3.065372682	2.35E-06	2.58E-05
ENSG00000171425	ZNF581	707.0935215	1159.439717	-0.712928376	2.35E-06	2.58E-05
ENSG00000213020	ZNF611	650.9405641	418.5926309	0.637935815	2.36E-06	2.58E-05
ENSG00000076513	ANKRD13A	1054.475685	1496.437652	-0.505070702	2.37E-06	2.60E-05
ENSG00000136059	VILL	356.0972216	208.4013562	0.772157929	2.39E-06	2.61E-05
ENSG00000185803	SLC52A2	1769.947978	1109.095207	0.673561417	2.39E-06	2.62E-05
ENSG00000153208	MERTK	234.4018979	401.4698491	-0.776628808	2.40E-06	2.62E-05
ENSG00000164284	GRPEL2	1310.980981	1897.316329	-0.533379994	2.43E-06	2.65E-05
ENSG00000214357	NEURL1B	695.6400586	1139.839455	-0.711578617	2.45E-06	2.67E-05
ENSG00000164187	LMBRD2	793.0179383	1135.078199	-0.51706686	2.47E-06	2.69E-05
ENSG00000278952	AP003068.4	18.21746231	77.05348931	-2.079117966	2.50E-06	2.73E-05
ENSG00000226950	DANCR	972.6095342	1378.331886	-0.503268811	2.51E-06	2.74E-05
ENSG00000167775	CD320	1223.204002	1869.81581	-0.612524886	2.51E-06	2.74E-05
ENSG00000235217	TSPY26P	23.66164322	74.23286839	-1.648711837	2.54E-06	2.76E-05
ENSG00000168404	MLKL	272.4285834	434.399672	-0.672501607	2.55E-06	2.77E-05
ENSG00000140948	ZCCHC14	4157.4164	3121.9603	0.413229438	2.56E-06	2.78E-05
ENSG00000083896	YTHDC1	2877.20722	3878.495323	-0.430785469	2.56E-06	2.79E-05
ENSG00000185909	KLHDC8B	16.8351719	62.64668703	-1.897790283	2.58E-06	2.80E-05
ENSG00000065357	DGKA	331.2248363	520.3320809	-0.652305625	2.60E-06	2.82E-05
ENSG00000277053	GTF2IP1	191.8059747	328.1275364	-0.77328248	2.63E-06	2.86E-05
ENSG00000145113	MUC4	17.59100791	91.17739068	-2.366093147	2.64E-06	2.86E-05
ENSG00000169255	B3GALNT1	746.8861961	1079.308037	-0.531116795	2.69E-06	2.92E-05
ENSG00000226695	ANKRD20A10P	0	16.28201335	-6.343658904	2.71E-06	2.93E-05
ENSG00000147862	NFIB	1606.244024	2399.02712	-0.579096563	2.75E-06	2.98E-05
ENSG00000189266	PNRC2	4158.792018	5579.490841	-0.424015172	2.77E-06	3.00E-05
ENSG00000164171	ITGA2	2190.582333	4318.475061	-0.97901599	2.80E-06	3.03E-05
ENSG00000167080	B4GALNT2	0.373875456	91.18621241	-7.866559245	2.82E-06	3.05E-05
ENSG00000230487	PSMG3-AS1	1072.886596	571.2796704	0.910585354	2.84E-06	3.07E-05
ENSG00000187997	C17orf99	1.838141323	26.31975612	-3.852570195	2.84E-06	3.07E-05
ENSG00000067191	CACNB1	634.2417657	416.153325	0.60828884	2.85E-06	3.08E-05
ENSG00000175115	PACS1	2141.155778	1514.266828	0.499583784	2.85E-06	3.08E-05
ENSG00000164946	FREM1	0	19.60086497	-6.60431989	2.86E-06	3.09E-05
ENSG00000125388	GRK4	305.6468862	185.8254161	0.718254796	2.87E-06	3.10E-05
ENSG00000119471	HSDL2	1378.542546	2060.591528	-0.580189559	2.92E-06	3.15E-05
ENSG00000129103	SUMF2	3436.326957	4627.791372	-0.429413467	2.94E-06	3.17E-05
ENSG00000188647	PTAR1	2717.709598	1899.504633	0.516663053	2.98E-06	3.21E-05
ENSG00000137876	RSL24D1	3542.903129	4893.839845	-0.466082857	3.02E-06	3.25E-05
ENSG00000182670	TTC3	10853.94034	8185.933691	0.406999696	3.05E-06	3.28E-05
ENSG00000171105	INSR	2038.221243	2776.549885	-0.446027751	3.05E-06	3.28E-05
ENSG00000174579	MSL2	1286.011731	1791.859671	-0.478694887	3.06E-06	3.29E-05
ENSG00000135604	STX11	20.86059523	80.66405054	-1.958091202	3.12E-06	3.36E-05
ENSG00000179348	GATA2	605.007014	901.3981617	-0.574775331	3.13E-06	3.36E-05
ENSG00000144802	NFKBIZ	130.6970194	274.7425421	-1.069483567	3.13E-06	3.37E-05

ENSG00000153234	NR4A2	134.554368	242.6306589	-0.851641029	3.17E-06	3.41E-05
ENSG00000148948	LRRC4C	28.96502406	0.631745859	5.573761491	3.21E-06	3.45E-05
ENSG00000170786	SDR16C5	201.3878231	106.1312981	0.925618091	3.28E-06	3.52E-05
ENSG00000186666	BCDIN3D	122.4936403	230.3365339	-0.910915917	3.28E-06	3.52E-05
ENSG00000235370	DNM1P51	3.602078331	32.86365823	-3.176477666	3.29E-06	3.53E-05
ENSG00000080819	CPOX	1963.985156	1188.687709	0.723737943	3.30E-06	3.54E-05
ENSG00000172216	CEBPB	1810.352798	3029.436905	-0.742725047	3.31E-06	3.55E-05
ENSG00000070019	GUCY2C	8.686656118	43.16024903	-2.30843765	3.32E-06	3.56E-05
ENSG00000169288	MRPL1	630.252333	962.7434919	-0.611857634	3.33E-06	3.56E-05
ENSG00000170385	SLC30A1	1664.974948	2284.087772	-0.4560013	3.35E-06	3.59E-05
ENSG00000285106	AC016831.6	88.1952582	35.87850024	1.297775304	3.38E-06	3.62E-05
ENSG00000169258	GPRIN1	595.1846218	340.7997187	0.802817543	3.39E-06	3.62E-05
ENSG00000268592	RAET1E-AS1	58.27381744	137.727021	-1.24289323	3.41E-06	3.64E-05
ENSG00000265972	TXNIP	5819.31448	776.8807618	2.904869506	3.42E-06	3.65E-05
ENSG00000254639	AC116021.1	26.69561522	2.235678064	3.631850489	3.42E-06	3.65E-05
ENSG00000131401	NAPSB	93.79593284	28.38491426	1.739159549	3.44E-06	3.67E-05
ENSG00000260953	AC009093.4	0.349140684	26.70384599	-6.09235831	3.45E-06	3.68E-05
ENSG00000130270	ATP8B3	521.3440835	336.2492719	0.634273613	3.53E-06	3.76E-05
ENSG00000246763	RGMB-AS1	37.2191427	109.193581	-1.553565357	3.57E-06	3.81E-05
ENSG00000143947	RPS27A	14427.70244	19507.41435	-0.435203169	3.58E-06	3.81E-05
ENSG00000168394	TAP1	683.9714287	1057.231226	-0.62886204	3.58E-06	3.82E-05
ENSG00000173727	AP000769.1	2.847826236	34.68879587	-3.593608137	3.61E-06	3.84E-05
ENSG00000106605	BLVRA	2053.10908	1357.332686	0.596397805	3.61E-06	3.84E-05
ENSG00000204390	HSPA1L	116.8595037	54.66000438	1.097396205	3.62E-06	3.85E-05
ENSG00000107902	LHPP	686.5576573	998.9378845	-0.540631542	3.62E-06	3.85E-05
ENSG00000204420	MPIG6B	36.46219931	94.32744211	-1.372144214	3.62E-06	3.85E-05
ENSG00000153093	ACOXL	229.7113412	123.0175774	0.897512327	3.62E-06	3.85E-05
ENSG00000279207	AC015813.6	1065.779949	713.6858596	0.578330274	3.63E-06	3.85E-05
ENSG00000166143	PPP1R14D	29.4587747	85.57826056	-1.535359012	3.66E-06	3.89E-05
ENSG00000103160	HSDL1	1512.277802	972.7248938	0.635735664	3.68E-06	3.91E-05
ENSG00000165023	DIRAS2	24.24006062	74.62880248	-1.616455992	3.68E-06	3.91E-05
ENSG00000137073	UBAP2	1768.453617	2623.421804	-0.569254163	3.72E-06	3.94E-05
ENSG00000162458	FBLIM1	207.8840406	370.6199076	-0.83322349	3.75E-06	3.97E-05
ENSG00000257671	KRT7-AS	14.86685554	55.40368849	-1.892966025	3.79E-06	4.01E-05
ENSG00000125510	OPRL1	64.01639953	21.78302983	1.556783424	3.79E-06	4.01E-05
ENSG00000168763	CNNM3	1698.431546	2326.545811	-0.453738817	3.81E-06	4.04E-05
ENSG00000164164	OTUD4	2592.741034	3487.739043	-0.427705632	3.84E-06	4.06E-05
ENSG00000107338	SHB	798.7911367	515.9118402	0.630045558	3.84E-06	4.06E-05
ENSG00000103254	FAM173A	1056.925566	695.5278917	0.602455531	3.92E-06	4.14E-05
ENSG00000220205	VAMP2	1145.081326	1752.084048	-0.613120645	3.94E-06	4.16E-05
ENSG00000274070	CASTOR2	232.0725683	387.1280911	-0.736585515	3.95E-06	4.17E-05
ENSG00000071082	RPL31	13094.1322	18139.22838	-0.470171685	3.96E-06	4.18E-05
ENSG00000174992	ZG16	2.847826236	29.61894567	-3.356350233	3.97E-06	4.19E-05

ENSG00000074695	LMAN1	9608.745427	12946.61163	-0.430136817	3.97E-06	4.19E-05
ENSG00000072134	EPN2	2220.105777	1506.662462	0.55885858	3.98E-06	4.20E-05
ENSG00000250748	AC025419.1	21.84300545	0.300152569	6.070366571	4.04E-06	4.26E-05
ENSG00000074416	MGLL	85.72716655	26.3457894	1.700639476	4.04E-06	4.26E-05
ENSG00000131737	KRT34	21.39096442	0.331593289	6.04126873	4.04E-06	4.26E-05
ENSG00000197747	S100A10	9436.955241	13540.93429	-0.520866475	4.07E-06	4.29E-05
ENSG00000102854	MSLN	49.99020935	152.477157	-1.603145367	4.09E-06	4.30E-05
ENSG00000143801	PSEN2	994.9865674	676.7281196	0.556493438	4.11E-06	4.32E-05
ENSG00000166974	MAPRE2	1026.512118	605.705019	0.759815779	4.12E-06	4.33E-05
ENSG00000113621	TXNDC15	1036.802408	1673.035041	-0.689947314	4.12E-06	4.33E-05
ENSG00000110711	AIP	1690.894279	1218.867791	0.472418496	4.15E-06	4.36E-05
ENSG00000130766	SESN2	1139.654703	2090.53015	-0.875143754	4.16E-06	4.37E-05
ENSG00000065802	ASB1	2005.252556	1364.90316	0.554467844	4.17E-06	4.38E-05
ENSG00000119314	PTBP3	8271.322372	11346.78202	-0.456156468	4.17E-06	4.38E-05
ENSG00000131115	ZNF227	585.507134	324.7586372	0.852093067	4.18E-06	4.38E-05
ENSG00000135093	USP30	582.8567289	870.9881296	-0.578731544	4.21E-06	4.41E-05
ENSG00000204592	HLA-E	3409.831485	4968.227901	-0.542907564	4.24E-06	4.44E-05
ENSG00000137955	RABGGTB	1666.582893	2488.769091	-0.578845409	4.27E-06	4.47E-05
ENSG00000167202	TBC1D2B	1586.59301	1126.776379	0.493780997	4.34E-06	4.54E-05
ENSG00000050344	NFE2L3	2248.275333	1475.495014	0.607111205	4.37E-06	4.58E-05
ENSG00000187098	MITF	370.5778414	211.6329874	0.806842686	4.38E-06	4.58E-05
ENSG00000224272	AC131097.3	140.491181	263.1416888	-0.90353025	4.42E-06	4.62E-05
ENSG00000132677	RHBG	176.2914147	92.44843957	0.931776914	4.48E-06	4.68E-05
ENSG00000261578	AP003119.3	159.4265796	72.79558633	1.12696761	4.51E-06	4.71E-05
ENSG00000237550	RPL9P9	3135.727968	4357.817185	-0.47475891	4.52E-06	4.72E-05
ENSG00000105379	ETFB	1295.454374	875.0364358	0.565286393	4.57E-06	4.77E-05
ENSG00000102786	INTS6	1253.992441	1746.013398	-0.477297387	4.61E-06	4.81E-05
ENSG00000182870	GALNT9	97.77695526	34.7495403	1.500936603	4.62E-06	4.82E-05
ENSG00000184500	PROS1	885.1311274	1345.565626	-0.603455319	4.65E-06	4.84E-05
ENSG00000164128	NPY1R	0	16.01965999	-6.315849458	4.66E-06	4.86E-05
ENSG00000178623	GPR35	656.8212437	1125.78049	-0.776673405	4.68E-06	4.87E-05
ENSG00000179361	ARID3B	442.2269514	257.3316725	0.782952596	4.74E-06	4.93E-05
ENSG00000112562	SMOC2	1.072156824	28.04842185	-4.684890273	4.77E-06	4.96E-05
ENSG00000196337	CGB7	104.2841005	43.69818103	1.261381697	4.77E-06	4.96E-05
ENSG00000139835	GRTP1	609.2339628	883.0840198	-0.53504804	4.78E-06	4.97E-05
ENSG00000163956	LRPAP1	3961.344004	2990.429078	0.405630745	4.79E-06	4.98E-05
ENSG00000161671	EMC10	4645.967275	3484.348265	0.415021813	4.79E-06	4.98E-05
ENSG00000211829	TRDC	14.29874921	0	6.419237082	4.83E-06	5.01E-05
ENSG00000132109	TRIM21	224.1973938	376.6151682	-0.748508229	4.83E-06	5.01E-05
ENSG00000137266	SLC22A23	945.9353204	651.9070037	0.537603267	4.84E-06	5.02E-05
ENSG00000127995	CASD1	487.7476189	751.4939298	-0.622913695	4.88E-06	5.06E-05
ENSG00000149043	SYT8	76.2087236	239.4591346	-1.650780734	4.88E-06	5.06E-05
ENSG00000141458	NPC1	2011.543707	1290.998316	0.639231483	4.90E-06	5.07E-05

ENSG00000181666	HKR1	914.3726746	510.4148234	0.842599815	4.94E-06	5.11E-05
ENSG00000135821	GLUL	2516.690012	3481.624223	-0.468221759	4.94E-06	5.11E-05
ENSG00000059758	CDK17	1446.930807	1010.293454	0.518130492	4.95E-06	5.13E-05
ENSG00000136327	NKX2-8	0	18.34681979	-6.520776169	5.00E-06	5.17E-05
ENSG00000132763	MMACHC	466.6614054	699.9662567	-0.585725173	5.01E-06	5.18E-05
ENSG00000138134	STAMBPL1	1006.231723	565.6768921	0.829589986	5.06E-06	5.23E-05
ENSG00000129084	PSMA1	387.3871599	651.142759	-0.750125465	5.08E-06	5.25E-05
ENSG00000134470	IL15RA	248.2552811	410.1309632	-0.722659716	5.10E-06	5.27E-05
ENSG00000169583	CLIC3	640.5107557	984.404733	-0.618999129	5.11E-06	5.27E-05
ENSG00000165816	VWA2	1366.934919	808.6496338	0.758464338	5.12E-06	5.28E-05
ENSG00000017427	IGF1	21.04959488	0.331593289	6.017756594	5.14E-06	5.30E-05
ENSG00000272068	AL365181.2	1418.978731	905.5081494	0.648522969	5.17E-06	5.33E-05
ENSG00000185187	SIGIRR	1260.918336	2115.311908	-0.745909193	5.17E-06	5.33E-05
ENSG00000198944	SOWAHA	25.81372801	76.1174477	-1.564158196	5.21E-06	5.36E-05
ENSG00000163362	INAVA	2205.634515	3106.053674	-0.493931715	5.22E-06	5.37E-05
ENSG00000196678	ERI2	685.7739187	973.1423013	-0.50463883	5.23E-06	5.38E-05
ENSG00000164294	GPX8	1323.529135	1847.3403	-0.480828841	5.26E-06	5.41E-05
ENSG00000100823	APEX1	5524.236633	7377.763433	-0.417432044	5.28E-06	5.43E-05
ENSG00000169188	APEX2	1490.014445	905.5097537	0.717601185	5.37E-06	5.52E-05
ENSG00000101335	MYL9	2.156046052	25.92038226	-3.576283517	5.41E-06	5.55E-05
ENSG00000235007	AL161785.2	24.46917475	0.663186579	5.323154537	5.41E-06	5.55E-05
ENSG00000090661	CERS4	2.17427964	24.82879152	-3.509870507	5.44E-06	5.58E-05
ENSG00000271254	AC240274.1	453.6577287	782.049921	-0.78475248	5.44E-06	5.58E-05
ENSG00000005700	IBTK	3088.228707	4507.995799	-0.545964943	5.46E-06	5.60E-05
ENSG00000160963	COL26A1	5.812825147	34.65426709	-2.571725736	5.47E-06	5.61E-05
ENSG00000175662	TOM1L2	2379.999763	3310.005498	-0.475685862	5.48E-06	5.62E-05
ENSG00000112078	KCTD20	3120.244036	4524.684053	-0.536407293	5.58E-06	5.72E-05
ENSG00000052841	TTC17	3383.353162	5021.195214	-0.569369692	5.60E-06	5.74E-05
ENSG00000125691	RPL23	14418.23274	19223.42595	-0.414959447	5.61E-06	5.74E-05
ENSG00000123395	ATG101	859.6698622	553.4880337	0.633863935	5.61E-06	5.74E-05
ENSG00000182010	RTKN2	499.5113921	817.7846918	-0.711793358	5.64E-06	5.76E-05
ENSG00000182253	SYNM	42.57723553	102.601717	-1.271363165	5.65E-06	5.77E-05
ENSG00000109680	TBC1D19	341.6400808	208.0328237	0.716658722	5.65E-06	5.77E-05
ENSG00000166819	PLIN1	53.3536559	16.44411608	1.704879498	5.67E-06	5.79E-05
ENSG00000079459	FDFT1	14666.45718	9907.866724	0.565799662	5.71E-06	5.83E-05
ENSG00000073417	PDE8A	1380.583645	2002.610444	-0.536861875	5.78E-06	5.90E-05
ENSG00000183597	TANGO2	570.7455673	378.6580973	0.59157143	5.79E-06	5.91E-05
ENSG00000272047	GTF2H5	275.7122917	454.8970081	-0.723346113	5.81E-06	5.92E-05
ENSG00000144895	EIF2A	3457.751358	4776.004226	-0.465879742	5.83E-06	5.94E-05
ENSG00000134716	CYP2J2	233.3682941	103.3251269	1.181164579	5.92E-06	6.03E-05
ENSG00000010319	SEMA3G	39.81946985	100.790495	-1.340010598	5.93E-06	6.04E-05
ENSG00000113583	C5orf15	2098.509241	2863.138966	-0.447969642	6.02E-06	6.13E-05
ENSG00000103257	SLC7A5	21077.26184	29911.66729	-0.505043167	6.04E-06	6.14E-05

ENSG00000103546	SLC6A2	21.84427541	1.272338916	4.161450716	6.05E-06	6.15E-05
ENSG00000105538	RASIP1	4586.875648	2955.168143	0.634071445	6.05E-06	6.15E-05
ENSG00000266714	MYO15B	400.5103696	2005.631536	-2.323913125	6.07E-06	6.17E-05
ENSG00000225675	LINC01771	49.44921513	14.18094537	1.802302005	6.11E-06	6.20E-05
ENSG00000143952	VPS54	1235.882793	1720.488716	-0.477201984	6.11E-06	6.21E-05
ENSG00000182648	LINC01006	224.7677261	127.4962303	0.820856385	6.12E-06	6.21E-05
ENSG00000185760	KCNQ5	24.22309699	1.710541329	3.768134868	6.17E-06	6.26E-05
ENSG00000227674	LINC00355	0	15.09909747	-6.234317634	6.18E-06	6.26E-05
ENSG00000089248	ERP29	4510.35969	6268.417755	-0.474943929	6.19E-06	6.28E-05
ENSG00000242963	AC026336.1	22.4136517	0.994779868	4.605737915	6.20E-06	6.28E-05
ENSG00000139998	RAB15	592.0193479	889.939567	-0.588080999	6.21E-06	6.29E-05
ENSG00000171811	CFAP46	17.0736411	60.13001015	-1.812403811	6.31E-06	6.39E-05
ENSG00000008735	MAPK8IP2	128.7740488	233.0079912	-0.854779404	6.34E-06	6.42E-05
ENSG00000234424	AL353743.4	49.16000643	12.15650515	2.020596337	6.37E-06	6.44E-05
ENSG00000224940	PRRT4	132.8234135	63.07853095	1.078041407	6.41E-06	6.48E-05
ENSG00000231650	RFESDP1	37.5890569	6.035000416	2.674443528	6.42E-06	6.49E-05
ENSG00000164070	HSPA4L	680.2503164	1193.087949	-0.811371889	6.46E-06	6.53E-05
ENSG00000062598	ELMO2	1091.718652	754.2451555	0.533034631	6.48E-06	6.55E-05
ENSG00000107295	SH3GL2	491.4339099	767.4253145	-0.642072316	6.48E-06	6.55E-05
ENSG00000171604	CXXC5	4378.164419	3057.537006	0.517929724	6.52E-06	6.58E-05
ENSG00000102243	VGLL1	0.373875456	23.24401307	-5.892134902	6.62E-06	6.67E-05
ENSG00000274080	AC005089.1	91.3387831	191.1007835	-1.065077815	6.65E-06	6.71E-05
ENSG00000248538	AC022784.1	107.8092832	47.80409274	1.178569862	6.66E-06	6.71E-05
ENSG00000188056	TREML4	13.05995017	0	6.29103641	6.70E-06	6.75E-05
ENSG00000040487	PQLC2	989.7175932	659.7775648	0.583794843	6.71E-06	6.76E-05
ENSG00000165138	ANKS6	2950.385265	2211.609733	0.415858064	6.72E-06	6.76E-05
ENSG00000108239	TBC1D12	448.9687784	662.1476581	-0.560252706	6.75E-06	6.79E-05
ENSG00000158985	CDC42SE2	2029.446621	2715.751412	-0.420205068	6.85E-06	6.89E-05
ENSG00000253552	HOXA-AS2	280.3475085	161.7550716	0.796054967	6.88E-06	6.91E-05
ENSG00000197728	RPS26	7270.850165	10061.13613	-0.468614408	6.88E-06	6.91E-05
ENSG00000150347	ARID5B	1821.11815	2762.414822	-0.600784936	6.89E-06	6.91E-05
ENSG00000283547	AC099654.7	13.95610971	0	6.383951023	6.89E-06	6.91E-05
ENSG00000132436	FIGNL1	814.0057257	1203.946629	-0.564951465	6.89E-06	6.92E-05
ENSG00000267519	AC020916.1	1617.712707	1142.191123	0.502219313	6.95E-06	6.97E-05
ENSG00000128283	CDC42EP1	1458.610365	2071.891952	-0.506630575	6.96E-06	6.98E-05
ENSG00000083845	RPS5	15443.43523	20314.53731	-0.395510043	6.98E-06	6.99E-05
ENSG00000189431	RASSF10	0	15.06026891	-6.22693687	7.00E-06	7.01E-05
ENSG00000278917	AC006213.6	13.0352154	0	6.288799109	7.02E-06	7.03E-05
ENSG00000088179	PTPN4	863.1994715	1211.963357	-0.489646168	7.08E-06	7.08E-05
ENSG00000230356	NCAPD2P1	0	15.89397541	-6.31308774	7.11E-06	7.12E-05
ENSG00000198961	PJA2	3297.707186	4343.90102	-0.397547167	7.13E-06	7.13E-05
ENSG00000070731	ST6GALNAC2	75.83771324	164.1523441	-1.110031862	7.15E-06	7.15E-05
ENSG00000159433	STARD9	1232.522941	845.9885575	0.542808747	7.19E-06	7.18E-05

ENSG00000166987	MBD6	1914.365152	2904.641727	-0.60125866	7.19E-06	7.19E-05
ENSG00000009950	MLXIPL	3135.303564	5255.007366	-0.74494795	7.27E-06	7.27E-05
ENSG00000185101	ANO9	1504.555353	2763.829867	-0.876999921	7.30E-06	7.29E-05
ENSG00000128016	ZFP36	2270.290876	1446.691128	0.649806802	7.30E-06	7.29E-05
ENSG00000181894	ZNF329	351.6156334	197.9360566	0.832253046	7.36E-06	7.34E-05
ENSG00000163902	RPN1	11126.39989	15063.13826	-0.4370647	7.40E-06	7.37E-05
ENSG00000172428	COPS9	737.4644633	488.3909528	0.593890006	7.40E-06	7.38E-05
ENSG00000025434	NR1H3	941.8377674	652.4455605	0.528953936	7.42E-06	7.39E-05
ENSG00000228794	LINC01128	325.6347754	202.827139	0.682635707	7.43E-06	7.40E-05
ENSG00000184669	OR7E14P	94.10575247	173.7195054	-0.88406808	7.43E-06	7.40E-05
ENSG00000168159	RNF187	5005.689852	8502.935803	-0.764320614	7.54E-06	7.51E-05
ENSG00000122741	DCAF10	1381.225454	1896.93577	-0.457671963	7.56E-06	7.52E-05
ENSG00000225295	MTND4P4	22.89550735	0.931898428	4.652124335	7.60E-06	7.55E-05
ENSG00000159885	ZNF222	0.373875456	23.56771021	-5.912511591	7.63E-06	7.59E-05
ENSG00000133019	CHRM3	303.0841336	177.6358345	0.768096082	7.65E-06	7.60E-05
ENSG00000147041	SYTL5	1.464265867	30.74335819	-4.395251586	7.72E-06	7.67E-05
ENSG00000172667	ZMAT3	1059.210189	719.8030245	0.556263217	7.73E-06	7.68E-05
ENSG00000166002	SMCO4	413.066903	262.2052172	0.654252552	7.81E-06	7.75E-05
ENSG00000143164	DCAF6	2465.747398	3259.729094	-0.402673725	7.88E-06	7.82E-05
ENSG00000131069	ACSS2	3976.420144	2653.278438	0.583635751	7.88E-06	7.82E-05
ENSG00000158716	DUSP23	387.2580923	237.1593376	0.705349749	7.93E-06	7.86E-05
ENSG00000173726	TOMM20	6875.799583	8982.818247	-0.385651566	8.00E-06	7.92E-05
ENSG00000141337	ARSG	533.4932991	283.5279323	0.913360072	8.01E-06	7.94E-05
ENSG00000001461	NIPAL3	1452.814144	1997.378907	-0.459229134	8.04E-06	7.96E-05
ENSG00000144063	MALL	149.3811203	67.49396347	1.14339212	8.05E-06	7.96E-05
ENSG00000220848	RPS18P9	76.78968092	151.7281565	-0.98237367	8.12E-06	8.03E-05
ENSG00000140950	TLDC1	1689.039477	1219.922026	0.469495988	8.18E-06	8.09E-05
ENSG00000144713	RPL32	13403.45442	17953.85994	-0.421684546	8.25E-06	8.15E-05
ENSG00000175573	C11orf68	950.2902183	632.0695707	0.587252242	8.25E-06	8.15E-05
ENSG00000134910	STT3A	6139.40795	8720.769246	-0.506311081	8.25E-06	8.15E-05
ENSG00000151150	ANK3	1493.575013	2117.782829	-0.503405823	8.33E-06	8.22E-05
ENSG00000104980	TIMM44	1781.452141	2527.709561	-0.504882472	8.37E-06	8.26E-05
ENSG00000155974	GRIP1	319.6640352	197.3000109	0.697091916	8.38E-06	8.27E-05
ENSG00000172007	RAB33B	350.8930968	546.0294195	-0.637772987	8.45E-06	8.33E-05
ENSG00000117614	SYF2	1293.125174	1834.997756	-0.50460533	8.47E-06	8.35E-05
ENSG00000186994	KANK3	39.32175795	93.92367744	-1.255299211	8.48E-06	8.35E-05
ENSG00000101695	RNF125	403.8636847	651.900931	-0.69210972	8.51E-06	8.39E-05
ENSG00000259330	INAFM2	143.6799114	68.9721328	1.05395223	8.65E-06	8.52E-05
ENSG00000274895	AC011700.1	21.51590824	1.464423001	3.844877537	8.71E-06	8.57E-05
ENSG00000167100	SAMD14	47.47170629	10.46950839	2.193709339	8.74E-06	8.59E-05
ENSG00000155008	APOOL	838.2229382	1195.357008	-0.512335537	8.76E-06	8.61E-05
ENSG00000110171	TRIM3	219.2780369	371.4329752	-0.761986748	8.84E-06	8.69E-05
ENSG00000255045	AP000866.5	12.28873445	48.33902773	-1.968739212	8.89E-06	8.74E-05

ENSG00000124224	PPP4R1L	13.74649913	53.45750473	-1.961411898	8.91E-06	8.75E-05
ENSG00000226435	ANKRD18DP	25.9543655	3.11354225	3.086587469	8.95E-06	8.79E-05
ENSG00000095787	WAC	6264.083663	8315.191245	-0.408680121	9.01E-06	8.84E-05
ENSG00000075073	TACR2	36.66293137	8.30065984	2.158106247	9.04E-06	8.87E-05
ENSG00000126432	PRDX5	7492.258636	5300.987145	0.499138331	9.11E-06	8.93E-05
ENSG00000151164	RAD9B	73.37262976	25.56224738	1.518482272	9.13E-06	8.95E-05
ENSG00000188343	FAM92A	1507.908971	1096.887705	0.459224233	9.22E-06	9.04E-05
ENSG00000241570	PAQR9-AS1	46.16741887	10.72696263	2.095873359	9.24E-06	9.05E-05
ENSG00000134308	YWHAQ	16201.116	10178.78593	0.670435405	9.28E-06	9.08E-05
ENSG00000277459	AP001527.2	102.9541335	46.74351271	1.136417348	9.34E-06	9.14E-05
ENSG00000119772	DNMT3A	1102.657893	1639.831037	-0.571985028	9.34E-06	9.14E-05
ENSG00000031698	SARS	7812.339551	11307.84216	-0.533476683	9.39E-06	9.18E-05
ENSG00000143369	ECM1	133.8614804	56.71245829	1.233084712	9.43E-06	9.21E-05
ENSG00000186767	SPIN4	622.8055945	966.0653934	-0.633630997	9.43E-06	9.21E-05
ENSG00000180015	AC093909.1	0.716514956	24.57382599	-5.080919015	9.48E-06	9.25E-05
ENSG00000258125	AC025254.1	13.11719086	0	6.296249954	9.54E-06	9.31E-05
ENSG00000120784	ZFP30	472.352166	290.5005391	0.702737683	9.61E-06	9.37E-05
ENSG00000116016	EPAS1	843.6800737	1266.782325	-0.586403725	9.63E-06	9.40E-05
ENSG00000225968	ELFN1	142.1549089	71.90140881	0.984768857	9.68E-06	9.43E-05
ENSG00000013364	MVP	4622.6606	3194.77436	0.533259273	9.73E-06	9.48E-05
ENSG00000177675	CD163L1	22.45915999	0.609152337	5.218093351	9.77E-06	9.51E-05
ENSG00000104142	VPS18	4126.708661	2944.546322	0.486629123	9.82E-06	9.56E-05
ENSG00000150593	PDCD4	1870.45592	2563.662905	-0.454837984	9.84E-06	9.58E-05
ENSG00000152518	ZFP36L2	1028.046955	1428.962464	-0.475394333	9.86E-06	9.59E-05
ENSG00000107140	TESK1	1171.115256	764.9225785	0.61345134	9.90E-06	9.63E-05
ENSG00000196323	ZBTB44	1735.090856	2436.365718	-0.489341572	9.92E-06	9.65E-05
ENSG00000143882	ATP6V1C2	366.3635586	548.4018447	-0.582129832	9.93E-06	9.65E-05
ENSG00000189337	KAZN	747.0588683	473.9726707	0.655991384	9.94E-06	9.66E-05
ENSG00000239713	APOBEC3G	0	14.46194418	-6.177153051	9.99E-06	9.70E-05
ENSG00000204387	C6orf48	626.9838052	1072.976192	-0.774610298	1.00E-05	9.72E-05
ENSG00000214193	SH3D21	224.899485	422.1189674	-0.90685008	1.00E-05	9.73E-05
ENSG00000274173	AL035661.1	223.6682945	115.9487992	0.951691999	1.01E-05	9.77E-05
ENSG00000214293	APTR	200.3167849	373.2238832	-0.896539169	1.01E-05	9.78E-05
ENSG00000231728	TMSB15B-AS1	69.23796749	26.42241717	1.387847572	1.01E-05	9.82E-05
ENSG00000086475	SEPHS1	3086.618763	4538.420752	-0.556333629	1.02E-05	9.83E-05
ENSG00000126012	KDM5C	6501.733848	4865.927614	0.418032424	1.03E-05	9.93E-05
ENSG00000224259	LINC01133	1.470767051	30.34347602	-4.376198091	1.03E-05	9.96E-05
ENSG00000157593	SLC35B2	3067.842875	2218.902479	0.467103251	1.04E-05	0.000100236
ENSG00000179841	AKAP5	167.1061019	322.2447364	-0.948324946	1.04E-05	0.000100901
ENSG00000137135	ARHGEF39	786.1551624	1303.392549	-0.729862746	1.04E-05	0.000100991
ENSG00000198682	PAPSS2	352.7093572	696.4551395	-0.980446284	1.05E-05	0.000101313
ENSG00000178460	MCMDC2	2.958497727	26.95494174	-3.206676712	1.05E-05	0.000101525
ENSG00000131408	NR1H2	2042.709969	1471.74063	0.473085896	1.05E-05	0.000101562

ENSG00000153561	RMND5A	2346.716303	3151.636863	-0.42558261	1.06E-05	0.000102005
ENSG00000151414	NEK7	1715.806807	2430.841458	-0.502653299	1.06E-05	0.000102078
ENSG00000197363	ZNF517	614.3407242	388.7815757	0.661771324	1.06E-05	0.000102126
ENSG00000164828	SUN1	7608.721512	9844.139135	-0.371607428	1.07E-05	0.000103335
ENSG00000114853	ZBTB47	753.579159	507.3252427	0.571726427	1.08E-05	0.00010379
ENSG00000186231	KLHL32	53.31433484	11.75559363	2.193511655	1.08E-05	0.000103834
ENSG00000108861	DUSP3	3094.193126	2348.221632	0.397979323	1.08E-05	0.000103942
ENSG00000132330	SCLY	144.0255562	247.0597467	-0.777675138	1.10E-05	0.000106133
ENSG00000100934	SEC23A	2968.973303	4047.194244	-0.447057478	1.12E-05	0.000108236
ENSG00000179820	MYADM	1876.008235	2718.158147	-0.535134723	1.13E-05	0.000108522
ENSG00000008283	CYB561	1885.743536	2740.560511	-0.539240294	1.13E-05	0.000109116
ENSG00000197603	CPLANE1	1527.400746	2251.919942	-0.559747747	1.14E-05	0.000109131
ENSG00000151623	NR3C2	65.67758756	24.0275551	1.456342975	1.15E-05	0.000110238
ENSG00000176102	CSTF3	939.3785684	1397.805308	-0.574037328	1.15E-05	0.000110238
ENSG00000178878	APOLD1	264.7018175	448.6899631	-0.76020837	1.15E-05	0.000110371
ENSG00000179869	ABCA13	12.93627631	0	6.279488188	1.15E-05	0.000110627
ENSG00000080845	DLGAP4	2209.998905	1586.924532	0.47806345	1.15E-05	0.000110744
ENSG00000182580	EPHB3	404.0506098	620.5201595	-0.617789964	1.16E-05	0.000110855
ENSG00000171148	TADA3	3323.576119	2461.683332	0.433033754	1.16E-05	0.000110971
ENSG00000261663	AC009065.8	64.05572058	131.7617688	-1.038723281	1.16E-05	0.000111103
ENSG00000167968	DNASE1L2	132.9783121	279.3287679	-1.068810517	1.16E-05	0.000111103
ENSG00000016864	GLT8D1	920.2482744	1262.681664	-0.45660566	1.17E-05	0.000111574
ENSG00000076321	KLHL20	953.2024172	681.9357208	0.483087769	1.17E-05	0.000112436
ENSG00000280152	AC009078.3	148.5340939	73.54708829	1.016829898	1.18E-05	0.000112724
ENSG00000041982	TNC	150.1675419	62.60019722	1.2580952	1.18E-05	0.000113137
ENSG00000175854	SWI5	503.0546429	329.6571985	0.609394635	1.20E-05	0.000114323
ENSG00000187257	RSBN1L	1123.873385	1670.267377	-0.571925445	1.20E-05	0.000114422
ENSG00000105695	MAG	26.11990034	4.000253635	2.725691144	1.20E-05	0.000114515
ENSG00000169710	FASN	55838.76426	31524.49282	0.824765335	1.20E-05	0.00011475
ENSG00000137216	TMEM63B	2169.606264	1579.337784	0.457927135	1.21E-05	0.000115321
ENSG00000221986	MYBPHL	38.06995654	9.415795155	2.021410727	1.22E-05	0.000116193
ENSG00000111801	BTN3A3	159.3406541	270.3711103	-0.761769391	1.22E-05	0.000116193
ENSG00000107438	PDLIM1	2611.037133	3671.738334	-0.49177315	1.22E-05	0.000116731
ENSG00000108654	DDX5	16930.0269	22136.02752	-0.386825578	1.23E-05	0.000116882
ENSG00000120256	LRP11	77.36824969	147.414525	-0.930897674	1.23E-05	0.000117085
ENSG00000105835	NAMPT	4325.601922	2842.332397	0.605477214	1.23E-05	0.000117488
ENSG00000157353	FUK	1222.127382	1794.652472	-0.553940441	1.23E-05	0.000117548
ENSG00000160299	PCNT	4701.989096	6388.147834	-0.442282294	1.24E-05	0.000117771
ENSG00000155016	CYP2U1	555.5365376	821.172678	-0.563453263	1.25E-05	0.000119
ENSG00000130720	FIBCD1	557.1354275	343.7789628	0.69805117	1.25E-05	0.000119
ENSG00000107731	UNC5B	411.8379244	246.9075988	0.737640103	1.26E-05	0.00012
ENSG00000226287	TMEM191A	243.2677333	142.5644864	0.768794326	1.26E-05	0.000120039
ENSG00000245275	SAP30L-AS1	104.7542238	48.20690636	1.122049608	1.27E-05	0.000120413

ENSG00000214796	AC098934.1	120.98672	220.5034627	-0.865978125	1.27E-05	0.000120607
ENSG00000279443	AL513497.1	37.16444194	94.11225619	-1.341475445	1.28E-05	0.000121601
ENSG00000135521	LTV1	248.7039917	458.0826176	-0.882985917	1.28E-05	0.00012185
ENSG00000175029	CTBP2	5188.070155	6920.689278	-0.415766257	1.28E-05	0.000121851
ENSG00000271347	AC124312.5	12.4306419	0	6.217838584	1.29E-05	0.000122065
ENSG00000144579	CTDSP1	3876.719086	5510.195737	-0.507086168	1.30E-05	0.000122814
ENSG00000071282	LMCD1	297.8092974	171.3061545	0.796649267	1.30E-05	0.000123128
ENSG00000134955	SLC37A2	109.8367382	48.87748078	1.169954604	1.30E-05	0.000123147
ENSG00000171889	MIR31HG	50.50599225	14.01884264	1.844490177	1.30E-05	0.000123341
ENSG00000108091	CCDC6	2789.732723	3742.450607	-0.423908647	1.33E-05	0.000125548
ENSG00000114738	MAPKAPK3	2972.037008	1991.646574	0.577077253	1.33E-05	0.00012564
ENSG00000033100	CHPF2	4653.471834	2733.251803	0.767940407	1.33E-05	0.000126162
ENSG00000033170	FUT8	1071.171708	1567.256175	-0.548511086	1.34E-05	0.000126325
ENSG00000204569	PPP1R10	3605.502495	4767.203177	-0.403072788	1.38E-05	0.000130589
ENSG00000131759	RARA	844.238884	558.3382817	0.595537759	1.39E-05	0.000131007
ENSG00000124587	PEX6	708.0859259	1181.634759	-0.738098261	1.39E-05	0.000131181
ENSG00000133321	RARRES3	0.367374272	21.15669141	-5.75590781	1.39E-05	0.00013162
ENSG00000188522	FAM83G	2855.351043	2082.413058	0.455158354	1.40E-05	0.000131888
ENSG00000197063	MAFG	3990.190756	2783.703434	0.519145238	1.42E-05	0.000133735
ENSG00000083307	GRHL2	1897.530157	2511.318985	-0.404488037	1.42E-05	0.000133735
ENSG00000106049	HIBADH	1462.266056	2098.11628	-0.520686618	1.43E-05	0.000134538
ENSG00000167601	AXL	107.2909604	45.64204542	1.237542583	1.43E-05	0.000134781
ENSG00000168275	COA6	574.0732083	365.579153	0.649451806	1.44E-05	0.000135592
ENSG00000054611	TBC1D22A	1631.98498	1201.526592	0.441727422	1.44E-05	0.000135929
ENSG00000051128	HOMER3	760.754314	489.754994	0.63482744	1.45E-05	0.000135936
ENSG00000136279	DBNL	4513.913577	3366.938745	0.423119779	1.45E-05	0.000135936
ENSG00000099812	MISP	5701.160588	7414.264927	-0.378979999	1.45E-05	0.00013662
ENSG00000108175	ZMIZ1	1703.34681	2453.965637	-0.52646181	1.46E-05	0.000137425
ENSG00000243224	AC006252.1	29.79237309	91.5645686	-1.617549627	1.51E-05	0.000141471
ENSG00000214870	AC004540.1	107.589199	50.49026018	1.092643434	1.51E-05	0.000141471
ENSG00000005020	SKAP2	518.0862398	318.8455824	0.699035861	1.51E-05	0.000141785
ENSG00000138760	SCARB2	7659.13005	10274.56882	-0.423737906	1.52E-05	0.000142355
ENSG00000137090	DMRT1	20.12980795	0.855270664	4.489206388	1.52E-05	0.000142623
ENSG00000007372	PAX6	260.0781817	144.8906169	0.841729524	1.53E-05	0.000143006
ENSG00000266265	KLF14	28.35015071	4.614305097	2.634944894	1.54E-05	0.00014443
ENSG00000186188	FFAR4	0	13.06186198	-6.023975867	1.54E-05	0.00014451
ENSG00000168140	VASN	799.5097038	1198.385894	-0.583654605	1.54E-05	0.000144517
ENSG00000250412	KLHL2P1	39.11991852	9.580386594	2.025849608	1.55E-05	0.000145092
ENSG00000249306	LINC01411	57.6005848	18.7652691	1.630870853	1.55E-05	0.00014539
ENSG00000237438	CECR7	0	14.3808473	-6.157855871	1.58E-05	0.000147388
ENSG00000165996	HACD1	973.1843267	614.1309191	0.663170272	1.58E-05	0.000147388
ENSG00000087495	PHACTR3	31.93272549	6.482050027	2.319374372	1.58E-05	0.000147832
ENSG00000206190	ATP10A	19.93716099	0.886711385	4.468756422	1.59E-05	0.000148756

ENSG00000164346	NSA2	2515.7092	3358.089376	-0.416769236	1.60E-05	0.00014921
ENSG00000125652	ALKBH7	872.7680006	1300.792466	-0.575362099	1.61E-05	0.000149781
ENSG00000180113	TDRD6	20.79812332	1.595085007	3.761985752	1.61E-05	0.000149781
ENSG00000142534	RPS11	17665.21876	23770.13258	-0.428222101	1.61E-05	0.000150182
ENSG00000115592	PRKAG3	69.28220582	27.08706311	1.36099184	1.61E-05	0.000150308
ENSG00000181773	GPR3	231.3948979	117.2120303	0.976748858	1.62E-05	0.000150965
ENSG00000157873	TNFRSF14	474.451131	890.8255867	-0.907785568	1.62E-05	0.000151278
ENSG00000136636	KCTD3	2751.449049	3995.897616	-0.538561747	1.63E-05	0.000151554
ENSG00000168765	GSTM4	2003.584727	1189.688665	0.751331401	1.63E-05	0.000152121
ENSG00000131931	THAP1	267.8282498	434.1961352	-0.697763461	1.64E-05	0.00015279
ENSG00000196150	ZNF250	418.0317794	273.9558334	0.609604088	1.65E-05	0.000153284
ENSG00000198198	SZT2	2932.858139	2189.841634	0.421760498	1.66E-05	0.00015399
ENSG00000187239	FNBP1	14.81088481	63.78140611	-2.099176866	1.68E-05	0.000155966
ENSG00000174844	DNAH12	263.9990983	149.9660696	0.815869306	1.69E-05	0.000156891
ENSG00000204618	RNF39	549.4575228	342.2689228	0.684188337	1.69E-05	0.000157234
ENSG00000134508	CABLES1	780.1485829	1093.504004	-0.487039835	1.70E-05	0.000157878
ENSG00000160097	FNDC5	25.67673783	79.97664174	-1.643195378	1.70E-05	0.000157939
ENSG00000169045	HNRNPH1	15825.85996	22801.59334	-0.526819334	1.70E-05	0.000157961
ENSG00000145555	MYO10	5083.729304	6784.77332	-0.416423135	1.71E-05	0.000158508
ENSG00000171722	SPATA46	83.25623516	30.62755016	1.439527409	1.73E-05	0.000160245
ENSG00000143036	SLC44A3	302.7226296	533.439338	-0.815655431	1.74E-05	0.000161254
ENSG00000162817	C1orf115	910.2733722	1264.863047	-0.474924629	1.74E-05	0.000161604
ENSG00000094975	SUCO	3438.193985	2605.41296	0.400007911	1.76E-05	0.000163113
ENSG00000184900	SUMO3	3515.831693	2391.602763	0.55558664	1.76E-05	0.000163373
ENSG00000115548	KDM3A	2742.375821	1882.232361	0.543455565	1.77E-05	0.000163872
ENSG00000162522	KIAA1522	5268.549738	6892.329696	-0.387591398	1.77E-05	0.000163915
ENSG00000147872	PLIN2	2.192513228	23.91209877	-3.449490107	1.79E-05	0.000165331
ENSG00000107201	DDX58	424.9435595	616.5348636	-0.536985204	1.79E-05	0.000165775
ENSG00000169213	RAB3B	369.0713864	214.7376824	0.780574385	1.80E-05	0.000166008
ENSG00000100243	CYB5R3	6145.188816	4140.990987	0.569264988	1.80E-05	0.000166255
ENSG00000005893	LAMP2	4782.215993	3634.273541	0.395910162	1.80E-05	0.000166438
ENSG00000184307	ZDHHC23	1543.123806	1102.763716	0.484369335	1.81E-05	0.00016706
ENSG00000268812	AC004264.1	107.8441665	49.42375167	1.124359392	1.82E-05	0.000167675
ENSG00000030066	NUP160	4186.774197	2757.588247	0.602106858	1.84E-05	0.000169615
ENSG00000181904	C5orf24	2411.575625	3282.55854	-0.445045457	1.85E-05	0.000170206
ENSG00000173334	TRIB1	554.5183218	369.4662569	0.586741409	1.85E-05	0.000170867
ENSG00000106327	TFR2	530.4122012	319.6365122	0.728768178	1.86E-05	0.000171086
ENSG00000119596	YLPM1	3952.407763	2921.497039	0.435815287	1.86E-05	0.000171279
ENSG00000258769	RAP1AP	34.18914317	5.365455351	2.666302158	1.87E-05	0.000172558
ENSG00000275945	EIF3FP1	59.79801529	19.8578109	1.596787049	1.90E-05	0.000174446
ENSG00000133962	CATSPERB	31.30468717	5.406694321	2.529289544	1.92E-05	0.000176753
ENSG00000049769	PPP1R3F	343.2335545	190.4267566	0.85308323	1.95E-05	0.000179238
ENSG00000151779	NBAS	3145.262212	2288.799917	0.458386179	1.96E-05	0.000179954

ENSG00000136807	CDK9	2452.702219	1771.611283	0.468967661	1.96E-05	0.000180265
ENSG00000213462	ERV3-1	764.1775441	497.1047459	0.621990903	1.97E-05	0.000180792
ENSG00000188227	ZNF793	22.1517177	2.387474231	3.203322426	1.98E-05	0.000181568
ENSG00000100813	ACIN1	14209.98507	11085.95536	0.358138575	2.00E-05	0.000183129
ENSG00000204310	AGPAT1	1574.11088	1158.98265	0.441696958	2.01E-05	0.0001841
ENSG00000237489	C10orf143	104.3310301	48.15777124	1.117206415	2.01E-05	0.000184333
ENSG00000141736	ERBB2	7104.569991	9452.868626	-0.411905887	2.01E-05	0.000184479
ENSG00000154319	FAM167A	22.10747938	68.28129697	-1.624520114	2.02E-05	0.000185
ENSG00000154447	SH3RF1	651.5976265	990.3357964	-0.604424897	2.05E-05	0.000187689
ENSG00000163328	GPR155	292.8642385	180.2595378	0.700813518	2.05E-05	0.000187786
ENSG00000142233	NTN5	56.8463327	138.7093445	-1.283026678	2.06E-05	0.000188985
ENSG00000189164	ZNF527	223.7174614	126.5938833	0.82046779	2.08E-05	0.000189913
ENSG00000186591	UBE2H	7325.247097	5540.179359	0.402896719	2.08E-05	0.000190349
ENSG00000260852	FBXL19-AS1	446.2369838	267.6155336	0.73928751	2.08E-05	0.000190463
ENSG00000186854	TRABD2A	478.3324239	706.6841841	-0.563544391	2.09E-05	0.000190695
ENSG00000267510	AC011451.1	73.78710746	27.96492729	1.405335302	2.09E-05	0.000190772
ENSG00000170043	TRAPPC1	1335.326984	908.2352837	0.555393181	2.10E-05	0.000191486
ENSG00000233303	XXYLT1-AS1	59.54781369	18.85418383	1.662139058	2.10E-05	0.000191655
ENSG00000222375	RN7SKP127	0	12.80199733	-5.994189326	2.10E-05	0.000191968
ENSG00000107819	SFXN3	1885.055097	1356.580219	0.474497724	2.11E-05	0.000192186
ENSG00000084207	GSTP1	30446.16631	23395.60078	0.379993667	2.11E-05	0.000192189
ENSG00000177989	ODF3B	36.7177835	115.9615162	-1.657484895	2.13E-05	0.000194051
ENSG00000176658	MYO1D	6799.131849	5238.335684	0.376266279	2.13E-05	0.00019428
ENSG00000266733	TBC1D29	104.0537276	43.87995856	1.248529668	2.13E-05	0.000194296
ENSG00000003393	ALS2	2521.618158	1876.78431	0.425990676	2.14E-05	0.000194913
ENSG00000167513	CDT1	1849.401902	2580.983648	-0.481158619	2.14E-05	0.000194913
ENSG00000171360	KRT38	19.97489813	0.600305139	5.04986605	2.14E-05	0.000195119
ENSG00000222009	BTBD19	165.5099145	313.308538	-0.919748943	2.15E-05	0.000195749
ENSG00000112531	QKI	573.4245478	865.1123912	-0.593907019	2.18E-05	0.00019791
ENSG00000165730	STOX1	351.2194005	528.39156	-0.588166757	2.18E-05	0.000198265
ENSG00000003147	ICA1	1292.487323	1874.407777	-0.536034104	2.20E-05	0.000199473
ENSG00000135709	KIAA0513	545.563208	361.2504071	0.595894113	2.20E-05	0.000199927
ENSG00000121671	CRY2	1180.789728	798.2498479	0.565792986	2.20E-05	0.000200054
ENSG00000134909	ARHGAP32	3307.389386	2437.369918	0.440207682	2.21E-05	0.000200375
ENSG00000163577	EIF5A2	773.2672399	480.280136	0.685458663	2.22E-05	0.000201061
ENSG00000158315	RHBDL2	17.38504464	52.61246233	-1.596605762	2.22E-05	0.000201061
ENSG00000163219	ARHGAP25	73.08597221	27.6510284	1.40617416	2.22E-05	0.000201061
ENSG00000171747	LGALS4	7.339562925	46.21674728	-2.656593955	2.23E-05	0.000201851
ENSG00000204946	ZNF783	1220.031105	788.2848743	0.630785189	2.23E-05	0.000202097
ENSG00000253540	FAM86HP	499.3609088	324.3230928	0.623414176	2.25E-05	0.000203953
ENSG00000104231	ZFAND1	1526.985318	2099.15988	-0.45891827	2.28E-05	0.000206178
ENSG00000170949	ZNF160	1220.180621	809.2659789	0.593493057	2.28E-05	0.000206284
ENSG00000185483	ROR1	225.9286735	107.6558404	1.066284542	2.28E-05	0.000206284

ENSG00000226742	HSBP1L1	1688.849849	2389.975973	-0.500594369	2.28E-05	0.000206559
ENSG00000065057	NTHL1	304.1280709	486.8897071	-0.68018255	2.29E-05	0.000206713
ENSG00000141510	TP53	2322.328327	3163.664451	-0.446323891	2.30E-05	0.000207733
ENSG00000186723	OR10H1	1.090390412	22.66923291	-4.381207386	2.31E-05	0.000208554
ENSG00000278311	GGNBP2	2504.836571	3280.021303	-0.388958201	2.32E-05	0.000209261
ENSG00000105559	PLEKHA4	1719.367299	1178.369735	0.544484495	2.35E-05	0.000211835
ENSG00000269335	IKBKG	395.1713179	259.5267893	0.607357042	2.35E-05	0.000211876
ENSG00000198125	MB	99.12912831	244.8379063	-1.302389704	2.35E-05	0.000211876
ENSG00000127603	MACF1	11256.452	8611.133333	0.386415143	2.35E-05	0.000212242
ENSG00000267123	LINC02081	280.2839292	161.897434	0.79466218	2.36E-05	0.000212242
ENSG00000158286	RNF207	963.715014	1657.389036	-0.78171166	2.36E-05	0.000212242
ENSG00000175866	BAIAP2	1600.46163	2212.804532	-0.466954299	2.36E-05	0.000212811
ENSG00000125207	PIWIL1	20.38254947	1.563644287	3.740621978	2.37E-05	0.000213363
ENSG00000226380	LINC-PINT	105.3914658	44.74751619	1.234608366	2.37E-05	0.000213364
ENSG00000088808	PPP1R13B	855.2819482	1215.082777	-0.50660477	2.37E-05	0.000213438
ENSG00000140992	PDPK1	1615.848241	2141.412872	-0.406160006	2.38E-05	0.000213891
ENSG00000225969	ABHD11-AS1	24.76376603	72.35232819	-1.546542768	2.39E-05	0.00021469
ENSG00000129128	SPCS3	3752.723017	5310.863786	-0.501039076	2.39E-05	0.00021471
ENSG00000198918	RPL39	6094.332233	8217.771455	-0.431211292	2.39E-05	0.000214949
ENSG00000178149	DALRD3	1151.112184	1574.989858	-0.451966698	2.40E-05	0.00021547
ENSG00000130706	ADRM1	2584.526864	1726.186482	0.581801113	2.42E-05	0.000217274
ENSG00000007968	E2F2	1332.086866	839.7705293	0.664895669	2.47E-05	0.000221259
ENSG00000104695	PPP2CB	2874.773823	2054.879117	0.484029324	2.47E-05	0.000221933
ENSG00000013392	RWDD2A	321.4725245	492.2118906	-0.614075464	2.49E-05	0.000223324
ENSG00000196381	ZNF781	43.19877265	11.21327081	1.966718933	2.49E-05	0.000223324
ENSG00000148158	SNX30	1606.59899	2184.568182	-0.443146047	2.49E-05	0.000223455
ENSG00000005448	WDR54	1117.779411	776.5509244	0.524982456	2.50E-05	0.000223555
ENSG00000184995	IFNE	40.59448424	9.464930272	2.10725607	2.50E-05	0.000223618
ENSG00000204650	LINC02210	1626.008535	1144.273935	0.507485199	2.50E-05	0.000223836
ENSG00000137648	TMPRSS4	942.0145805	489.7064716	0.945326783	2.51E-05	0.000224344
ENSG00000156049	GNA14	3.203468104	27.46040367	-3.076685692	2.51E-05	0.00022448
ENSG00000180532	ZSCAN4	18.87150535	0.877864186	4.390971097	2.54E-05	0.000226717
ENSG00000108389	MTMR4	3085.130999	4219.197784	-0.451536045	2.54E-05	0.000227334
ENSG00000174527	MYO1H	22.23765442	82.43662632	-1.887501888	2.56E-05	0.000228332
ENSG00000101577	LPIN2	1678.356288	2235.365306	-0.413424485	2.56E-05	0.000228686
ENSG00000142449	FBN3	63.65694777	142.5096962	-1.160736701	2.56E-05	0.000228969
ENSG00000232445	AC006329.1	174.13806	302.3317181	-0.798233138	2.58E-05	0.000230305
ENSG00000067177	PHKA1	1083.514457	1484.084436	-0.454085062	2.59E-05	0.000230968
ENSG00000197136	PCNX3	4827.212016	3442.276494	0.487584709	2.59E-05	0.000231268
ENSG00000197111	PCBP2	5870.844455	7642.442176	-0.380393834	2.60E-05	0.000232176
ENSG00000126709	IFI6	694.6566699	1086.977824	-0.645271461	2.61E-05	0.000232228
ENSG00000067082	KLF6	2538.309023	1727.197351	0.55486831	2.62E-05	0.000233753
ENSG00000277149	TYW1B	176.7098425	282.3944648	-0.675817189	2.62E-05	0.000233753

ENSG00000186889	TMEM17	194.1284892	105.1055986	0.881804183	2.63E-05	0.000234221
ENSG00000126337	KRT36	19.61925626	0.663186579	5.003620034	2.64E-05	0.00023475
ENSG00000113384	GOLPH3	2752.164757	3572.785521	-0.376445156	2.64E-05	0.000234887
ENSG00000178229	ZNF543	463.1674699	312.9361099	0.564833259	2.64E-05	0.000234971
ENSG00000082269	FAM135A	536.155459	790.6788649	-0.561224308	2.65E-05	0.00023589
ENSG00000165886	UBTD1	672.8362069	457.339741	0.557242541	2.67E-05	0.000237566
ENSG00000081138	CDH7	87.31431578	591.8467955	-2.760956588	2.68E-05	0.000238084
ENSG00000164877	MICALL2	2650.051849	1766.683819	0.585337476	2.68E-05	0.000238084
ENSG00000163565	IFI16	21.58107145	2.181643822	3.336398893	2.69E-05	0.000239179
ENSG00000188549	CCDC9B	447.6498793	243.7529217	0.879413738	2.70E-05	0.000239354
ENSG00000130758	MAP3K10	1676.686709	1189.655294	0.494687193	2.70E-05	0.000239457
ENSG00000198727	MT-CYB	101722.0465	76385.56694	0.413252072	2.70E-05	0.000239717
ENSG00000169925	BRD3	1815.159313	2447.077058	-0.430987919	2.71E-05	0.000240242
ENSG00000176170	SPHK1	212.3098095	113.2133016	0.904318134	2.72E-05	0.000240599
ENSG00000214944	ARHGEF28	284.2327709	445.4904774	-0.647929188	2.72E-05	0.000240859
ENSG00000070778	PTPN21	829.8056621	576.5857236	0.524140399	2.75E-05	0.00024344
ENSG00000083750	RRAGB	370.4844363	584.0639349	-0.655263542	2.76E-05	0.000243939
ENSG00000274317	LINC02334	24.81054428	3.682406669	2.765627577	2.76E-05	0.000244175
ENSG00000135766	EGLN1	2861.51729	2168.84422	0.399990654	2.76E-05	0.000244411
ENSG00000188917	TRMT2B	773.7931684	549.6793176	0.492673084	2.79E-05	0.00024662
ENSG00000134138	MEIS2	376.9689214	237.0974072	0.666859844	2.80E-05	0.000247105
ENSG00000135378	PRRG4	615.1068713	900.3138146	-0.55047825	2.81E-05	0.000247831
ENSG00000135723	FHOD1	1476.188872	1008.095418	0.549561506	2.81E-05	0.000247923
ENSG00000155085	AK9	269.2950444	164.3985408	0.71337645	2.81E-05	0.0002481
ENSG00000075234	TTC38	783.3583784	1089.364112	-0.475156648	2.81E-05	0.00024814
ENSG00000139974	SLC38A6	255.6620706	154.9312001	0.723340096	2.83E-05	0.000249675
ENSG00000134287	ARF3	6918.026125	5195.203409	0.413026695	2.83E-05	0.000249888
ENSG00000196214	ZNF766	912.4252944	634.3121811	0.523694153	2.84E-05	0.00025036
ENSG00000135472	FAIM2	2.915529367	28.36327179	-3.279670201	2.86E-05	0.00025203
ENSG00000116133	DHCR24	17384.45797	12998.08022	0.419462395	2.89E-05	0.000254498
ENSG00000120756	PLS1	1386.438497	2072.66149	-0.580465808	2.89E-05	0.000254563
ENSG00000148090	AUH	679.7757158	978.3082447	-0.525207131	2.90E-05	0.000254895
ENSG00000145687	SSBP2	212.0120362	106.1292394	1.003144065	2.92E-05	0.000256919
ENSG00000124570	SERPINB6	2142.059487	2936.690259	-0.455099884	2.94E-05	0.000258939
ENSG00000198034	RPS4X	15991.13446	20703.3606	-0.372600394	2.96E-05	0.0002605
ENSG00000235257	ITGA9-AS1	262.8527484	124.1104454	1.087345622	2.98E-05	0.000262091
ENSG00000089053	ANAPC5	3965.231313	5312.390642	-0.422022037	3.00E-05	0.000263394
ENSG00000230061	TRPM2-AS	557.1861558	359.2038034	0.634820672	3.00E-05	0.000263424
ENSG00000115109	EPB41L5	1280.209054	1873.766485	-0.549838221	3.03E-05	0.00026612
ENSG00000181481	RNF135	583.2559558	899.5313803	-0.625468256	3.03E-05	0.000266273
ENSG00000214063	TSPAN4	1369.670218	1956.165909	-0.513708294	3.04E-05	0.000266696
ENSG00000083290	ULK2	970.1187682	1401.138923	-0.52974544	3.06E-05	0.000268253
ENSG00000122224	LY9	18.81157337	0.900457708	4.379435297	3.12E-05	0.000273251

ENSG00000081377	CDC14B	1436.451983	2071.726527	-0.527862843	3.14E-05	0.0002751
ENSG00000133059	DSTYK	1575.449858	1150.404353	0.453457838	3.14E-05	0.000275162
ENSG00000145016	RUBCN	1491.793145	998.93322	0.577715141	3.14E-05	0.000275454
ENSG00000158715	SLC45A3	1399.219698	941.3929582	0.571191677	3.15E-05	0.000275471
ENSG00000101680	LAMA1	93.28521857	39.26316475	1.252865343	3.15E-05	0.000275861
ENSG00000106351	AGFG2	291.9593518	499.7558374	-0.776620446	3.17E-05	0.000277498
ENSG00000272398	CD24	203.3104686	375.5403338	-0.883989372	3.18E-05	0.000278153
ENSG00000064201	TSPAN32	44.98094325	12.66987596	1.813628401	3.19E-05	0.000278709
ENSG00000174010	KLHL15	602.9042362	867.2972274	-0.523899383	3.20E-05	0.000279476
ENSG00000062370	ZNF112	200.5198943	104.8269191	0.941057521	3.20E-05	0.000279955
ENSG00000143198	MGST3	2891.201355	2200.745879	0.393466879	3.21E-05	0.000279955
ENSG00000111674	ENO2	2998.759366	2176.91587	0.461958246	3.23E-05	0.000281842
ENSG00000141314	RHBDL3	123.2767398	51.90760677	1.23944786	3.24E-05	0.000282565
ENSG00000168672	FAM84B	1779.134232	2432.716859	-0.451558699	3.26E-05	0.000284425
ENSG00000144130	NT5DC4	37.21264152	4.896763269	2.933120834	3.29E-05	0.000286602
ENSG00000154359	LONRF1	1295.743073	1853.198279	-0.516721746	3.31E-05	0.000288384
ENSG00000256802	AC022613.1	1.396562736	22.75280577	-3.985207963	3.32E-05	0.000289233
ENSG00000229660	AC004975.2	8.790826425	37.2426726	-2.086150379	3.33E-05	0.000290171
ENSG00000172671	ZFAND4	83.38830807	154.9302618	-0.894572949	3.35E-05	0.000291765
ENSG00000140743	CDR2	1079.540578	1675.214628	-0.634496001	3.37E-05	0.000293086
ENSG00000163481	RNF25	779.3857691	536.9834339	0.536192147	3.37E-05	0.000293086
ENSG00000105738	SIPA1L3	5129.023992	3872.688176	0.405305522	3.38E-05	0.000293911
ENSG00000152894	PTPRK	1491.741601	1974.98249	-0.404713699	3.40E-05	0.000296119
ENSG00000133112	TPT1	18628.06075	25042.92007	-0.426888315	3.41E-05	0.000296244
ENSG00000167186	COQ7	678.0890025	975.7093502	-0.525800993	3.42E-05	0.00029738
ENSG00000168065	SLC22A11	79.07367604	166.2754318	-1.071487978	3.43E-05	0.000297802
ENSG00000070018	LRP6	3451.58272	4516.452674	-0.387990991	3.44E-05	0.00029854
ENSG00000049860	HEXB	3028.611315	3997.046223	-0.400138099	3.44E-05	0.000298811
ENSG00000064490	RFXANK	1134.170921	1561.172415	-0.460998608	3.44E-05	0.000298889
ENSG00000147065	MSN	21.27379175	2.364880709	3.152211145	3.47E-05	0.000301102
ENSG00000171865	RNASEH1	1565.832038	1015.783172	0.623452031	3.48E-05	0.000301426
ENSG00000139718	SETD1B	3612.900549	4947.946166	-0.453588713	3.48E-05	0.000301426
ENSG00000205622	AP001043.1	12.570172	50.57565684	-1.998551009	3.48E-05	0.000301426
ENSG00000187017	ESPN	227.6390171	419.5791759	-0.880682686	3.48E-05	0.00030171
ENSG00000249379	AL033397.2	43.52032469	9.006114741	2.249419549	3.48E-05	0.000301798
ENSG00000224032	EPB41L4A-AS1	398.1922763	742.0743674	-0.897442831	3.49E-05	0.000302165
ENSG00000196954	CASP4	763.7814738	1174.521357	-0.620075025	3.50E-05	0.000303019
ENSG00000071967	CYBRD1	2040.972353	3211.760608	-0.653776125	3.52E-05	0.000304866
ENSG00000283897	AC011416.3	32.90975311	7.019473725	2.238925706	3.54E-05	0.000306362
ENSG00000156482	RPL30	6763.857616	9217.580842	-0.446518509	3.54E-05	0.00030642
ENSG00000069702	TGFBR3	1403.63956	1905.590214	-0.440840177	3.59E-05	0.000310264
ENSG00000103005	USB1	2154.747533	1409.796819	0.611418374	3.59E-05	0.000310264
ENSG00000050426	LETMD1	1553.017969	2218.198299	-0.514082031	3.60E-05	0.000310591

ENSG00000198719	DLL1	92.43996113	177.5468287	-0.938152397	3.60E-05	0.000310791
ENSG00000101126	ADNP	2953.064374	3889.455428	-0.397417498	3.61E-05	0.00031149
ENSG00000074855	ANO8	1336.184568	1850.298318	-0.469453453	3.63E-05	0.000313077
ENSG00000139531	SUOX	439.3366333	661.9912359	-0.59246976	3.65E-05	0.000314855
ENSG00000143502	SUSD4	905.8529635	629.7636892	0.525491264	3.67E-05	0.000316471
ENSG00000135144	DTX1	25.72652133	4.438456048	2.520047793	3.71E-05	0.000319839
ENSG00000161970	RPL26	855.0391896	1269.314923	-0.570521357	3.71E-05	0.000319839
ENSG00000278845	MRPL45	1454.812912	2000.141416	-0.459063517	3.72E-05	0.000320609
ENSG00000143674	MAP3K21	1611.846599	2347.293892	-0.542603876	3.75E-05	0.000322749
ENSG00000180855	ZNF443	458.3126483	303.2766724	0.595329693	3.77E-05	0.000324539
ENSG00000175348	TMEM9B	570.3008292	797.2667681	-0.48343421	3.82E-05	0.000328276
ENSG00000104267	CA2	132.0518726	302.2447452	-1.196782988	3.82E-05	0.000328872
ENSG00000141497	ZMYND15	77.64683336	154.1583418	-0.991493497	3.84E-05	0.000330341
ENSG00000184281	TSSC4	707.2508086	1018.096455	-0.526379348	3.85E-05	0.000330949
ENSG00000166997	CNPY4	86.8118492	164.8842623	-0.923427296	3.86E-05	0.000331796
ENSG00000143028	SYPL2	78.87944517	34.56097428	1.188327289	3.88E-05	0.000332828
ENSG00000135845	PIGC	901.5421525	661.8425408	0.44577502	3.88E-05	0.000332853
ENSG00000136848	DAB2IP	432.4491339	655.342015	-0.598310915	3.88E-05	0.000333313
ENSG00000105429	MEGF8	7038.095884	5378.718341	0.387990844	3.91E-05	0.000335758
ENSG00000160318	CLDND2	67.99916845	24.57975447	1.459622989	3.92E-05	0.000335908
ENSG00000263535	AK4P1	86.26420243	32.77475624	1.393174721	3.93E-05	0.000337162
ENSG00000055130	CUL1	4354.185715	3108.107744	0.486132269	3.95E-05	0.000338518
ENSG00000142484	TM4SF5	0.367374272	17.53665787	-5.488358334	3.96E-05	0.000338869
ENSG00000131094	C1QL1	38.72257826	103.5517271	-1.414842306	3.96E-05	0.000338965
ENSG00000129514	FOXA1	1151.349545	1588.796003	-0.464714843	3.97E-05	0.000339588
ENSG00000227036	LINC00511	388.9213549	630.6268544	-0.696737048	3.97E-05	0.000339776
ENSG00000149187	CELF1	9884.196026	7406.744746	0.416160649	3.99E-05	0.000341575
ENSG00000181004	BBS12	173.2262068	100.2215523	0.78861293	4.00E-05	0.00034248
ENSG00000213672	NCKIPSD	1774.923622	1286.055805	0.464195617	4.03E-05	0.000344189
ENSG00000176438	SYNE3	462.7277892	284.7571572	0.700303177	4.07E-05	0.000347649
ENSG00000233223	AC016876.1	265.8383553	160.288681	0.732295994	4.09E-05	0.000349484
ENSG00000168300	PCMTD1	687.8866712	1120.515001	-0.703166662	4.10E-05	0.000350015
ENSG00000213930	GALT	454.3049791	653.8850053	-0.524616289	4.10E-05	0.000350183
ENSG00000167112	TRUB2	1461.098591	1944.544952	-0.412632065	4.11E-05	0.00035033
ENSG00000134755	DSC2	2605.971776	4003.669894	-0.619264158	4.13E-05	0.000352054
ENSG00000164615	CAMLG	1080.93044	1527.817172	-0.499149281	4.13E-05	0.000352126
ENSG00000148516	ZEB1	44.30724529	11.17152353	1.988264969	4.15E-05	0.000353789
ENSG00000187840	EIF4EBP1	2347.89637	3482.1053	-0.568809468	4.15E-05	0.000353957
ENSG00000171132	PRKCE	450.5207162	301.2822263	0.579352566	4.16E-05	0.0003543
ENSG00000117054	ACADM	1616.198024	2290.636508	-0.503499149	4.18E-05	0.000355713
ENSG00000068323	TFE3	3109.422786	2182.780569	0.510089479	4.18E-05	0.000356193
ENSG00000196466	ZNF799	321.8016963	183.8812783	0.804757058	4.21E-05	0.000357959
ENSG00000133067	LGR6	492.0887322	303.4045981	0.700176525	4.22E-05	0.000358639

ENSG00000132275	RRP8	377.8772674	563.0802391	-0.576215891	4.22E-05	0.00035864
ENSG00000176595	KBTBD11	1124.714145	807.677917	0.477512739	4.23E-05	0.000359578
ENSG00000096433	ITPR3	12303.62337	9293.317139	0.404745861	4.27E-05	0.000362574
ENSG00000182325	FBXL6	1432.082034	1044.060395	0.4553324	4.29E-05	0.000364031
ENSG00000134318	ROCK2	7567.072362	5599.363584	0.434467737	4.31E-05	0.000366036
ENSG00000136352	NKX2-1	55.37398173	11.6308601	2.233427516	4.33E-05	0.000367345
ENSG00000130529	TRPM4	1351.974403	1829.079934	-0.436019057	4.38E-05	0.000371527
ENSG00000125731	SH2D3A	2407.507735	1748.551808	0.461207675	4.39E-05	0.000372091
ENSG00000105976	MET	2010.816452	2602.906929	-0.37233123	4.40E-05	0.000372912
ENSG00000235863	B3GALT4	113.407104	196.3297922	-0.790399952	4.40E-05	0.000372912
ENSG00000152669	CCNO	579.9572071	915.4912965	-0.658803984	4.41E-05	0.000373653
ENSG00000145536	ADAMTS16	28.81012547	4.661980854	2.603876424	4.43E-05	0.000375697
ENSG00000177732	SOX12	3028.020803	2247.215663	0.430296873	4.44E-05	0.000376414
ENSG00000180592	SKIDA1	536.8927363	362.9185633	0.563904021	4.45E-05	0.000376973
ENSG00000177984	LCN15	0.72301614	23.29211884	-4.997312355	4.49E-05	0.000379648
ENSG00000179344	HLA-DQB1	44.70346695	12.69736861	1.802331159	4.50E-05	0.000381066
ENSG00000173818	ENDOV	811.5070036	531.2760007	0.612540053	4.55E-05	0.000385102
ENSG00000179965	ZNF771	307.1813839	450.6454238	-0.553336548	4.56E-05	0.000385423
ENSG00000160199	PKNOX1	1441.529595	2014.399716	-0.48240428	4.61E-05	0.000389406
ENSG00000203993	ARRDC1-AS1	1139.421662	821.7016588	0.471598085	4.63E-05	0.00039146
ENSG00000165879	FRAT1	177.6009221	282.3256548	-0.668122186	4.67E-05	0.000394538
ENSG00000070540	WIPI1	1021.042866	699.0273734	0.546807751	4.69E-05	0.000396035
ENSG00000162913	OBSCN-AS1	247.0346932	117.9191925	1.071589556	4.69E-05	0.000396153
ENSG00000250312	ZNF718	475.3333127	323.8103996	0.553132021	4.71E-05	0.000397182
ENSG00000242516	LINC00960	205.5324937	323.7096279	-0.655189061	4.73E-05	0.000398881
ENSG00000167785	ZNF558	1374.999421	1010.715356	0.443879703	4.74E-05	0.00039961
ENSG00000260757	AC093520.1	1.813406552	21.07809598	-3.541438469	4.78E-05	0.000402855
ENSG00000171813	PWWP2B	1342.966007	1833.577439	-0.448939429	4.79E-05	0.000403359
ENSG00000175416	CLTB	2393.593444	1827.91935	0.388798878	4.80E-05	0.000404364
ENSG00000251002	AC244502.1	22.50354968	3.08210153	2.887541645	4.83E-05	0.000406496
ENSG00000184292	TACSTD2	3709.628103	5704.034061	-0.620847213	4.85E-05	0.000407931
ENSG00000113300	CNOT6	3201.601632	4238.582269	-0.404867125	4.89E-05	0.000411443
ENSG00000100448	CTSG	10.21862512	0	5.935533193	4.89E-05	0.000411443
ENSG00000131188	PRR7	1797.106279	1199.662966	0.582414087	4.94E-05	0.000414978
ENSG00000255587	RAB44	27.47872594	5.08394823	2.428656057	4.99E-05	0.000419175
ENSG00000174939	ASPHD1	41.11962221	94.65473562	-1.206355538	5.00E-05	0.000420319
ENSG00000123179	EBPL	1192.425114	1699.295865	-0.51102913	5.01E-05	0.000420384
ENSG00000143552	NUP210L	38.17412685	86.57261042	-1.179716443	5.01E-05	0.000420847
ENSG00000136436	CALCOCO2	2065.43776	2835.141618	-0.45678245	5.05E-05	0.000424194
ENSG00000151491	EPS8	1337.523284	1846.389549	-0.465358506	5.06E-05	0.000424194
ENSG00000106038	EVX1	43.94002238	112.0344506	-1.34699232	5.07E-05	0.000424847
ENSG00000027869	SH2D2A	156.9488301	84.06969261	0.899331163	5.08E-05	0.000426165
ENSG00000177954	RPS27	12631.65389	17868.73512	-0.500351731	5.09E-05	0.000426319

ENSG00000185519	FAM131C	244.4597541	119.2346467	1.030715777	5.14E-05	0.000430814
ENSG00000071909	MYO3B	87.96185764	34.84687222	1.328889702	5.15E-05	0.000431573
ENSG00000067167	TRAM1	5182.576486	7466.840053	-0.526752272	5.16E-05	0.000431869
ENSG00000170006	TMEM154	117.0010972	210.0670622	-0.845297488	5.16E-05	0.000431869
ENSG00000119661	DNAL1	428.9626331	622.1580994	-0.53548858	5.16E-05	0.000431869
ENSG00000196878	LAMB3	4671.632454	2869.140439	0.703637736	5.17E-05	0.000432339
ENSG00000143614	GATAD2B	2176.40931	1516.435286	0.520848148	5.25E-05	0.00043911
ENSG00000104892	KLC3	683.861887	441.1799467	0.630813198	5.30E-05	0.000443298
ENSG00000186866	POFUT2	1684.246984	1220.573303	0.464616565	5.32E-05	0.000444311
ENSG00000185404	SP140L	493.0375545	744.3200929	-0.593400759	5.32E-05	0.000444633
ENSG00000144668	ITGA9	40.3301729	112.0324702	-1.4706375	5.35E-05	0.000446387
ENSG00000140006	WDR89	320.4547657	484.8966166	-0.598680356	5.36E-05	0.000447457
ENSG00000152439	ZNF773	70.47153531	30.45411152	1.210368785	5.37E-05	0.000447792
ENSG00000186470	BTN3A2	255.0776174	405.5286844	-0.669753016	5.38E-05	0.000448736
ENSG00000136295	TTYH3	5293.951418	4061.049578	0.38256395	5.38E-05	0.000448884
ENSG00000165861	ZFYVE1	847.3483161	596.5916433	0.506684882	5.41E-05	0.000450914
ENSG00000135622	SEMA4F	1330.436311	934.5284982	0.508892298	5.41E-05	0.000450914
ENSG00000104218	CSPP1	707.8859902	972.3878934	-0.458589774	5.47E-05	0.000455991
ENSG00000181830	SLC35C1	1709.219385	1289.292295	0.406610591	5.48E-05	0.000456462
ENSG00000145604	SKP2	1353.2554	2010.940695	-0.571665437	5.48E-05	0.000456462
ENSG00000182040	USH1G	14.07090505	53.28784483	-1.912273591	5.49E-05	0.000456756
ENSG00000214135	AC132008.2	1116.69061	795.0965515	0.490572726	5.52E-05	0.000459015
ENSG00000130449	ZSWIM6	988.8620276	701.1854997	0.495169725	5.54E-05	0.000460855
ENSG00000223125	RNU2-32P	10.43886072	0	5.969633044	5.55E-05	0.000461792
ENSG00000110536	PTPMT1	253.3236775	159.2871126	0.667804773	5.56E-05	0.000462216
ENSG00000261888	AC144831.1	6.97091869	46.48691849	-2.738783055	5.60E-05	0.000465219
ENSG00000135451	TROAP	1714.115953	2575.207774	-0.587376577	5.62E-05	0.000466673
ENSG00000072422	RHOBTB1	121.4371771	58.92364073	1.037347056	5.62E-05	0.000466673
ENSG00000164930	FZD6	1542.771941	2066.432567	-0.421247411	5.62E-05	0.000466673
ENSG00000257727	CNPY2	130.2942854	222.4456328	-0.772395212	5.64E-05	0.000467791
ENSG00000170265	ZNF282	1692.073138	2207.749078	-0.383834356	5.67E-05	0.000470014
ENSG00000135052	GOLM1	4637.953195	6089.282909	-0.392855623	5.67E-05	0.000470413
ENSG00000163032	VSNL1	1952.74432	1344.437904	0.537897233	5.69E-05	0.000471933
ENSG00000161677	JOSD2	823.9969383	586.7797702	0.489620222	5.71E-05	0.000473504
ENSG00000136842	TMOD1	93.20563167	44.58592177	1.063862209	5.72E-05	0.000473827
ENSG00000118260	CREB1	1271.509875	1739.160654	-0.452218568	5.73E-05	0.000474264
ENSG00000102547	CAB39L	1981.496226	3137.312007	-0.662580917	5.73E-05	0.000474264
ENSG00000130584	ZBTB46	727.1232942	521.5452515	0.479681015	5.75E-05	0.0004754
ENSG00000225328	LINC01594	16.42879053	75.59936013	-2.200200704	5.77E-05	0.000477637
ENSG00000158828	PINK1	1131.113005	809.9605658	0.48138389	5.80E-05	0.000479817
ENSG00000205414	AC007608.1	95.23165405	42.53245124	1.167186711	5.85E-05	0.000483131
ENSG00000232043	AL133230.1	74.19220765	31.38111082	1.241452513	5.85E-05	0.000483131
ENSG00000149054	ZNF215	130.0537528	223.3237319	-0.780675408	5.89E-05	0.000486312

ENSG00000123136	DDX39A	1553.582159	2210.250694	-0.508953339	5.90E-05	0.0004869
ENSG00000210140	MT-TC	3617.476734	5010.926783	-0.470204363	5.93E-05	0.000489642
ENSG00000105519	CAPS	201.816248	367.8195866	-0.864421276	5.95E-05	0.000491022
ENSG00000130675	MNX1	351.1933958	505.7815864	-0.526363305	5.97E-05	0.000492123
ENSG00000166037	CEP57	2597.257332	3324.838762	-0.356333244	5.98E-05	0.000492827
ENSG00000116717	GADD45A	1107.293735	652.4727523	0.762082139	5.98E-05	0.000492827
ENSG00000167333	TRIM68	256.0660634	404.4878448	-0.661090584	5.99E-05	0.000493438
ENSG00000122861	PLAU	1383.516435	422.4260788	1.710930546	5.99E-05	0.000493505
ENSG00000272009	AL121944.1	115.9409868	199.6173852	-0.786153745	6.00E-05	0.000493769
ENSG00000135299	ANKRD6	89.54900394	38.98414634	1.201147176	6.00E-05	0.000494166
ENSG00000284691	AC073111.5	189.8311459	302.8855464	-0.672761009	6.02E-05	0.000495706
ENSG00000107796	ACTA2	164.361176	86.52261529	0.920655709	6.02E-05	0.000495706
ENSG00000175215	CTDSP2	7007.434015	9167.048004	-0.387482751	6.03E-05	0.000496084
ENSG00000169359	SLC33A1	548.8191651	909.978072	-0.729038781	6.04E-05	0.000496543
ENSG00000146830	GIGYF1	4378.589031	6254.183466	-0.514183682	6.06E-05	0.000498025
ENSG00000182095	TNRC18	10047.34114	13286.72161	-0.40311677	6.14E-05	0.000504593
ENSG00000158882	TOMM40L	523.8449951	338.3448284	0.628662729	6.14E-05	0.000504593
ENSG00000158865	SLC5A11	26.25926786	4.770049337	2.450546704	6.16E-05	0.000505886
ENSG00000116977	LGALS8	6558.158044	4790.183833	0.453406344	6.17E-05	0.000506699
ENSG00000111879	FAM184A	227.439869	138.9233187	0.709630968	6.21E-05	0.000509247
ENSG00000165269	AQP7	57.73868235	117.0708924	-1.019616691	6.23E-05	0.000510656
ENSG00000067715	SYT1	26.44430625	5.241151831	2.353914776	6.25E-05	0.000512734
ENSG00000124098	FAM210B	1126.869715	1519.196009	-0.431084426	6.26E-05	0.000513184
ENSG00000183747	ACSM2A	9.78227775	0	5.874471573	6.26E-05	0.000513272
ENSG00000228624	HDAC2-AS2	345.6165141	168.8667961	1.036779684	6.33E-05	0.000518406
ENSG00000165175	MID1IP1	2662.860849	3646.160599	-0.453456913	6.33E-05	0.000518773
ENSG00000261488	TBILA	122.6070143	60.15122261	1.028920233	6.34E-05	0.000518917
ENSG00000115525	ST3GAL5	263.7544531	156.1289044	0.752815248	6.34E-05	0.000518917
ENSG00000171169	NAIF1	265.1935159	415.8404554	-0.648076999	6.35E-05	0.000519529
ENSG00000166340	TPP1	1401.745297	1926.210003	-0.45807085	6.36E-05	0.000520051
ENSG00000143590	EFNA3	264.2018908	157.5786173	0.745011237	6.36E-05	0.000520069
ENSG00000127220	ABHD8	178.2340403	305.8033299	-0.776682136	6.51E-05	0.000532214
ENSG00000112679	DUSP22	789.9883928	562.3636433	0.490945626	6.53E-05	0.00053335
ENSG00000276900	AC023157.3	111.8415217	54.0984495	1.052308718	6.54E-05	0.000534103
ENSG00000186832	KRT16	23.97431673	3.166117132	2.881842756	6.56E-05	0.000535309
ENSG00000174780	SRP72	4593.140316	6235.783497	-0.441218238	6.59E-05	0.000537493
ENSG00000264364	DYNLL2	6368.609622	4954.774902	0.362069685	6.59E-05	0.00053772
ENSG00000269890	AL353593.1	3.699747455	25.76953715	-2.814994929	6.63E-05	0.000540479
ENSG00000109618	SEPSECS	745.6144117	1032.71391	-0.469567528	6.63E-05	0.000540532
ENSG00000268516	AC020915.3	317.1460087	186.4520935	0.769769779	6.63E-05	0.000540532
ENSG00000165458	INPPL1	6098.504952	8299.821305	-0.444511118	6.64E-05	0.000541107
ENSG00000131788	PIAS3	2506.439602	1845.168898	0.441722363	6.65E-05	0.000542084
ENSG00000116678	LEPR	152.4569421	83.93850956	0.864085388	6.66E-05	0.000542606

ENSG00000088833	NSFL1C	2165.268941	1473.254774	0.554860142	6.70E-05	0.000545186
ENSG00000072135	PTPN18	1460.361768	1896.822842	-0.377195353	6.74E-05	0.00054874
ENSG00000274021	AC024909.2	20.6433761	0.663186579	5.070759275	6.79E-05	0.000552456
ENSG00000126733	DACH2	17.46575014	1.272338916	3.835697434	6.83E-05	0.000555251
ENSG00000088305	DNMT3B	320.8186328	510.1570442	-0.670410264	6.85E-05	0.00055714
ENSG00000059122	FLYWCH1	2548.664487	1861.610434	0.452716756	6.87E-05	0.000558631
ENSG00000103227	LMF1	873.0780082	574.612607	0.604904122	6.88E-05	0.000559152
ENSG00000237654	AP003025.1	9.88771802	0	5.887230589	6.89E-05	0.000559235
ENSG00000249353	NPM1P27	588.8315958	833.3752554	-0.502084005	6.91E-05	0.000561066
ENSG00000182899	RPL35A	11808.74629	15450.25247	-0.387765197	6.92E-05	0.000561622
ENSG00000284883	AC021066.2	2.553386316	23.96957278	-3.233487696	6.94E-05	0.000562789
ENSG00000204348	DXO	467.3663506	663.0935107	-0.50393986	6.95E-05	0.000563375
ENSG00000075275	CELSR1	4977.521944	6576.619898	-0.401811111	7.00E-05	0.000567429
ENSG00000076685	NT5C2	1268.142619	1721.635343	-0.440681604	7.12E-05	0.000576974
ENSG00000109794	FAM149A	188.5438446	298.6922704	-0.662988097	7.14E-05	0.00057868
ENSG00000137440	FGFBP1	484.1212711	726.7027039	-0.586211231	7.17E-05	0.00058043
ENSG00000198856	OSTC	1899.110186	2665.865661	-0.489216705	7.17E-05	0.000580911
ENSG00000231107	LINC01508	48.04091999	98.91038479	-1.043087944	7.18E-05	0.00058154
ENSG00000169239	CA5B	129.0570703	68.62335342	0.908974471	7.23E-05	0.000585326
ENSG00000250564	AC109454.3	17.10883831	0.900457708	4.241008453	7.26E-05	0.000587052
ENSG00000086015	MAST2	4711.367414	3592.885892	0.390901527	7.26E-05	0.00058734
ENSG00000120616	EPC1	735.2315329	1000.40163	-0.443758142	7.29E-05	0.000589063
ENSG00000010810	FYN	2264.17954	1623.726304	0.479469137	7.30E-05	0.000589632
ENSG00000163714	U2SURP	4597.564313	6580.000528	-0.517356293	7.31E-05	0.000590732
ENSG00000055044	NOP58	2869.93049	4060.00346	-0.500674965	7.32E-05	0.000590827
ENSG00000149573	MPZL2	1390.976987	900.4129446	0.626432109	7.36E-05	0.000594463
ENSG00000197013	ZNF429	269.3657528	173.7347894	0.632998186	7.46E-05	0.000601952
ENSG00000272701	MESTIT1	74.963272	30.84566753	1.271891136	7.47E-05	0.000602916
ENSG00000140416	TPM1	1548.243253	2220.497398	-0.520147034	7.48E-05	0.000603431
ENSG00000141959	PFKL	8723.545623	6745.330051	0.371001475	7.52E-05	0.000606066
ENSG00000270207	AC068313.1	40.37964245	10.71562672	1.922453383	7.52E-05	0.000606097
ENSG00000133422	MORC2	3020.209584	2162.58456	0.481507055	7.53E-05	0.000607061
ENSG00000146426	TIAM2	82.58046259	148.9865737	-0.852045697	7.54E-05	0.000607061
ENSG00000158806	NPM2	1.482499455	18.90331895	-3.692006299	7.59E-05	0.000611172
ENSG00000196693	ZNF33B	1125.259137	1688.0292	-0.584620236	7.59E-05	0.000611292
ENSG00000066294	CD84	9.892949241	0	5.887952075	7.64E-05	0.000614716
ENSG00000203697	CAPN8	64.22632406	15.86047598	2.037731157	7.68E-05	0.000617625
ENSG00000147180	ZNF711	415.0169858	677.5324189	-0.705882728	7.68E-05	0.000617666
ENSG00000182747	SLC35D3	47.54987186	14.22072498	1.730954106	7.70E-05	0.000618944
ENSG00000131153	GINS2	1383.589869	892.626725	0.631359313	7.74E-05	0.000622386
ENSG00000103222	ABCC1	6321.173048	4614.163996	0.453970739	7.76E-05	0.000623225
ENSG00000116661	FBXO2	963.0489105	1346.805651	-0.483395397	7.76E-05	0.000623256
ENSG00000056736	IL17RB	793.7691627	563.4180998	0.494297506	7.78E-05	0.000624981

ENSG00000147251	DOCK11	186.564892	338.7312106	-0.859969485	7.84E-05	0.000629497
ENSG00000104872	PIH1D1	2594.739162	3333.539044	-0.361460096	7.89E-05	0.000633201
ENSG00000198576	ARC	1.825138956	20.27977827	-3.474928207	7.92E-05	0.000635395
ENSG00000175147	TMEM51-AS1	171.5512117	307.944112	-0.842486119	7.97E-05	0.00063892
ENSG00000124107	SLPI	103.6968046	188.224578	-0.857908728	8.00E-05	0.000641498
ENSG00000163297	ANTXR2	236.3779967	145.1562407	0.705981115	8.01E-05	0.000642152
ENSG00000171310	CHST11	62.95470514	24.13167551	1.389690946	8.03E-05	0.000643565
ENSG00000233559	LINC00513	72.31507045	140.0861298	-0.953719916	8.04E-05	0.000644164
ENSG00000172752	COL6A5	1.396562736	22.2776769	-3.954120048	8.06E-05	0.000644921
ENSG00000131149	GSE1	5703.142243	7526.948986	-0.400180113	8.06E-05	0.00064515
ENSG00000118181	RPS25	10999.84501	14457.10097	-0.394283913	8.09E-05	0.000647263
ENSG00000137944	KYAT3	1825.235403	2364.412828	-0.373361717	8.09E-05	0.000647337
ENSG00000160255	ITGB2	155.4335108	67.64514756	1.208202273	8.15E-05	0.000651576
ENSG00000104320	NBN	1668.649801	2280.303773	-0.45092391	8.18E-05	0.000653944
ENSG00000177054	ZDHHC13	464.3677525	698.7856204	-0.589327425	8.19E-05	0.000653944
ENSG00000032219	ARID4A	964.7914714	1316.907024	-0.448663624	8.21E-05	0.000656051
ENSG00000140299	BNIP2	2164.570357	1669.147667	0.375124759	8.25E-05	0.000658908
ENSG00000140391	TSPAN3	5300.676983	4165.126179	0.347905336	8.27E-05	0.000660144
ENSG00000074657	ZNF532	1472.30657	1030.542807	0.515436809	8.29E-05	0.0006614
ENSG00000267260	AC020928.1	13.81943348	0.300152569	5.410122652	8.30E-05	0.000661721
ENSG00000107159	CA9	390.2356141	671.1911041	-0.780918245	8.39E-05	0.000668738
ENSG00000261116	AL049555.1	113.5271306	189.9843327	-0.741206313	8.41E-05	0.000670025
ENSG00000101972	STAG2	4638.271616	6250.493066	-0.430492663	8.42E-05	0.000670803
ENSG00000168310	IRF2	463.3264923	646.0183137	-0.479249817	8.44E-05	0.000672321
ENSG00000151689	INPP1	718.6868284	490.5322559	0.549765691	8.57E-05	0.000682352
ENSG00000099992	TBC1D10A	426.209936	288.2203643	0.562658465	8.61E-05	0.000685535
ENSG00000139192	TAPBPL	259.2590691	420.6261917	-0.697008924	8.62E-05	0.000685679
ENSG00000111843	TMEM14C	3042.612448	2377.784262	0.355756957	8.66E-05	0.000688697
ENSG00000074356	NCBP3	3096.915907	4094.146312	-0.402861404	8.71E-05	0.000692362
ENSG00000188211	NCR3LG1	1375.720517	2059.603263	-0.582632065	8.75E-05	0.000695848
ENSG00000171408	PDE7B	0	10.3791343	-5.691313013	8.76E-05	0.000696558
ENSG00000238279	BX470102.1	47.71651408	15.484725	1.627237185	8.78E-05	0.000697806
ENSG00000171621	SPSB1	675.024131	455.0085291	0.567622985	8.79E-05	0.000698188
ENSG00000091039	OSBPL8	4127.233423	2847.528688	0.535170101	8.83E-05	0.000701444
ENSG00000011275	RNF216	2613.102656	1918.622147	0.44532777	8.87E-05	0.000703889
ENSG00000149273	RPS3	28090.65739	37071.43009	-0.400222066	8.91E-05	0.00070666
ENSG00000017260	ATP2C1	4314.832704	5594.075147	-0.374487549	8.93E-05	0.000708369
ENSG00000278535	DHRS11	866.6658296	1306.307857	-0.592453487	8.94E-05	0.000708995
ENSG00000169621	APLF	162.2626846	91.97397561	0.818606195	8.94E-05	0.000708997
ENSG00000165288	BRWD3	1462.73249	1937.913828	-0.405992589	8.96E-05	0.000709978
ENSG00000134049	IER3IP1	744.7659864	1005.247108	-0.43296919	8.99E-05	0.000712015
ENSG00000231956	HNRNPA1P9	1.096891595	18.52163949	-4.083725483	9.00E-05	0.000712716
ENSG00000260565	ERVK13-1	1406.957047	1002.568602	0.489625141	9.00E-05	0.000712885

ENSG00000211445	GPX3	382.301172	244.5300011	0.643300158	9.02E-05	0.000713819
ENSG00000119408	NEK6	2202.488251	1637.969508	0.427219342	9.03E-05	0.000714505
ENSG00000213853	EMP2	3804.122301	4911.094522	-0.368382492	9.04E-05	0.000714688
ENSG00000258472	AC005726.1	357.3423593	233.6638027	0.613857803	9.17E-05	0.000724683
ENSG00000131469	RPL27	11307.12465	14969.80838	-0.404862064	9.19E-05	0.000726471
ENSG00000132300	PTCD3	3121.910195	4104.184159	-0.394807882	9.23E-05	0.000729456
ENSG00000092820	EZR	10758.47887	15662.80813	-0.541930816	9.34E-05	0.000737734
ENSG00000118418	HMGN3	1169.194114	1672.112477	-0.516308998	9.39E-05	0.000741595
ENSG00000055732	MCOLN3	1551.261537	926.9206667	0.743709649	9.42E-05	0.00074358
ENSG00000173210	ABLIM3	41.93777877	11.81701571	1.831145343	9.45E-05	0.000745868
ENSG00000232298	AL138902.1	0.72301614	19.22262527	-4.721207929	9.50E-05	0.000748921
ENSG00000138613	APH1B	253.0073567	400.7674953	-0.661932618	9.50E-05	0.000748921
ENSG00000163867	ZMYM6	254.9103472	376.0971844	-0.56152884	9.66E-05	0.000761623
ENSG00000112739	PRPF4B	3507.542484	4502.859237	-0.360365516	9.66E-05	0.000761623
ENSG00000008513	ST3GAL1	122.6656763	64.33669354	0.92769968	9.73E-05	0.000766731
ENSG00000167306	MYO5B	5627.496319	7778.465116	-0.467066004	9.75E-05	0.000767818
ENSG00000107372	ZFAND5	7601.096261	5772.335012	0.396913189	9.77E-05	0.000769627
ENSG00000032389	EIPR1	1588.520755	1152.848636	0.461837868	9.80E-05	0.000771258
ENSG00000160584	SIK3	1559.639604	2064.455202	-0.404624243	9.84E-05	0.000774382
ENSG00000130962	PRRG1	386.497339	590.3326495	-0.612151849	9.85E-05	0.000774954
ENSG00000164038	SLC9B2	351.2579311	534.6840799	-0.607744786	9.86E-05	0.00077544
ENSG00000280123	AC023632.6	93.18881941	184.9056095	-0.990581211	9.93E-05	0.0007802
ENSG00000147789	ZNF7	1103.741634	811.0710499	0.444599028	9.93E-05	0.0007802
ENSG00000164136	IL15	45.50195737	15.74948878	1.53259695	9.93E-05	0.0007802
ENSG00000139146	SINHCAF	3578.380726	4731.595604	-0.403181288	9.94E-05	0.000780828
ENSG00000105894	PTN	0.367374272	15.11774292	-5.274190336	9.96E-05	0.000782111
ENSG00000101846	STS	214.0981673	350.3809128	-0.70904658	9.98E-05	0.000783439
ENSG00000091490	SEL1L3	7512.339171	5877.817767	0.353917042	9.98E-05	0.000783439
ENSG00000267905	AC008750.3	21.5552293	3.059508009	2.832289912	0.000100039	0.00078468
ENSG00000152952	PLOD2	1694.28772	2671.525887	-0.656755942	0.000100409	0.000787333
ENSG00000066697	MSANTD3	847.5997906	524.5199364	0.6909267	0.000101292	0.000793981
ENSG00000137825	ITPKA	345.9521729	202.0801844	0.772501864	0.000101321	0.000793981
ENSG00000140749	IGSF6	50.36106833	17.91738628	1.493777789	0.000101797	0.000797455
ENSG00000226057	PHF2P2	9.072263978	0	5.765334071	0.000102085	0.00079945
ENSG00000117410	ATP6V0B	3481.330914	2581.872832	0.430935144	0.000102139	0.000799621
ENSG00000062716	VMP1	7206.70148	5172.421672	0.478368958	0.000102337	0.00080092
ENSG00000065457	ADAT1	1338.539647	888.667632	0.590049721	0.00010258	0.000802559
ENSG00000168071	CCDC88B	314.4343904	194.6988101	0.693516282	0.000102891	0.000804737
ENSG00000121892	PDS5A	6874.269683	8963.15569	-0.3828343	0.000103068	0.000805867
ENSG00000115652	UXS1	1543.898039	1100.369911	0.48801599	0.000103183	0.000806513
ENSG00000225720	AL031846.1	9.115232337	0	5.770917581	0.000103391	0.00080788
ENSG00000150551	LYPD1	9.913722755	0	5.890666165	0.000103618	0.000809397
ENSG00000138378	STAT4	16.70499686	48.44571515	-1.536544609	0.000104336	0.000814745

ENSG00000103202	NME4	987.9203315	1365.612225	-0.467201951	0.000104475	0.00081547
ENSG00000272031	ANKRD34A	126.1156875	64.96499963	0.956670759	0.000104495	0.00081547
ENSG00000145916	RMND5B	1096.542868	1433.447671	-0.386388465	0.000105044	0.000819487
ENSG00000148832	PAOX	38.49457151	82.87551912	-1.10873957	0.000105264	0.000820945
ENSG00000133302	SLF1	876.7723648	494.1535024	0.825833612	0.000105525	0.000822717
ENSG00000067646	ZFY	359.9645561	518.5268131	-0.525715356	0.000106257	0.000828167
ENSG00000179314	WSCD1	93.17581705	164.6108079	-0.819672839	0.000106301	0.000828246
ENSG00000126351	THRA	2072.09963	1528.441362	0.438798529	0.000106592	0.000830249
ENSG00000114391	RPL24	13298.75568	16953.95248	-0.350342039	0.000106755	0.000831251
ENSG00000160752	FDPS	5557.367185	3966.45602	0.486425419	0.00010703	0.000833134
ENSG00000071243	ING3	342.4885061	487.8350788	-0.509565891	0.000107839	0.000839158
ENSG00000134419	RPS15A	2068.11858	2686.294921	-0.377353158	0.000107877	0.000839189
ENSG00000232354	VIPR1-AS1	37.48488659	86.15005644	-1.199330199	0.000108595	0.000844511
ENSG00000171862	PTEN	3506.637541	4858.993495	-0.470377109	0.000108702	0.000845073
ENSG00000084636	COL16A1	512.2979869	257.3923259	0.995538502	0.000108761	0.000845265
ENSG00000152767	FARP1	3417.815845	4448.157277	-0.380219426	0.000109006	0.000846855
ENSG00000116815	CD58	390.9824231	265.7105888	0.557519293	0.000109059	0.000846855
ENSG00000132517	SLC52A1	11.70239454	46.37892831	-1.986120258	0.000109069	0.000846855
ENSG00000105374	NKG7	42.00690323	12.82459085	1.720705515	0.000109126	0.00084703
ENSG00000278909	AC007608.4	98.08154367	44.35252041	1.150050455	0.000109636	0.000850718
ENSG00000167987	VPS37C	865.8088173	1173.807614	-0.438883144	0.000109911	0.000852582
ENSG00000174437	ATP2A2	15237.35054	20405.59447	-0.421393627	0.000110225	0.000854752
ENSG00000279227	AC009303.4	44.68127211	14.60540146	1.606175121	0.000110277	0.000854886
ENSG00000136153	LMO7	1427.382562	2044.675547	-0.51857663	0.000110379	0.000855401
ENSG00000087157	PGS1	1214.858704	918.0326275	0.404243103	0.000110475	0.000855877
ENSG00000283959	AP002851.1	105.4721713	51.67566477	1.028259556	0.000110593	0.000856521
ENSG00000230953	AC099677.1	0	10.13301598	-5.655433883	0.000110809	0.000857924
ENSG00000179873	NLRP11	61.41623496	24.47855278	1.328463647	0.000110859	0.000858038
ENSG00000143799	PARP1	5828.63058	9287.429158	-0.672228151	0.000110912	0.000858182
ENSG00000204583	LRCOL1	16.41578816	0.600305139	4.765703735	0.000111024	0.000858771
ENSG00000160785	SLC25A44	1201.754028	854.8509797	0.490415075	0.000111208	0.000859923
ENSG00000106178	CCL24	32.50860296	6.327335139	2.331854817	0.000111326	0.000860565
ENSG00000117625	RCOR3	1184.114997	1618.760824	-0.450925004	0.000111523	0.000861821
ENSG00000160345	C9orf116	404.0063714	272.6313623	0.566890612	0.000111765	0.000863421
ENSG00000198730	CTR9	1899.518302	1420.063128	0.419185056	0.000111807	0.000863471
ENSG00000089692	LAG3	55.9787066	18.00381229	1.642013472	0.000112077	0.000865282
ENSG00000273760	AC245041.1	13.50025879	47.7392309	-1.82034746	0.000112543	0.000868451
ENSG00000147689	FAM83A	8.673653751	33.77253313	-1.957224469	0.000112558	0.000868451
ENSG00000140398	NEIL1	494.3927103	852.4057956	-0.785180936	0.000112739	0.000869577
ENSG00000164631	ZNF12	1792.56743	1378.6904	0.378915995	0.000112807	0.000869823
ENSG00000003436	TFPI	271.5466962	515.7721477	-0.924192482	0.000113013	0.000871143
ENSG00000248727	LINC01948	7.944136427	32.14841781	-2.0105883	0.000113049	0.000871143
ENSG00000188554	NBR1	4679.931383	6196.456661	-0.404798845	0.000113361	0.000873274

ENSG00000064012	CASP8	1412.549039	1869.511511	-0.404488614	0.000114507	0.000881823
ENSG00000152520	PAN3	2356.536997	3303.264288	-0.486941863	0.000114917	0.000884709
ENSG00000229453	SPINK8	9.077495198	0	5.766033574	0.000115237	0.000886771
ENSG00000232712	KIZ-AS1	70.85428929	28.12213089	1.338447521	0.000115257	0.000886771
ENSG00000136810	TXN	11214.67943	7255.324172	0.628141913	0.000115479	0.000888193
ENSG00000139200	PIANP	81.83667294	37.57280653	1.12510801	0.000117754	0.000905414
ENSG00000143333	RGS16	151.1851493	278.9266829	-0.885279758	0.000117989	0.000906933
ENSG00000155438	NIFK	1050.056603	1407.453463	-0.422229841	0.000118288	0.000908947
ENSG00000255031	AP002807.1	30.88134218	77.95892444	-1.333175293	0.000118453	0.000909931
ENSG00000118197	DDX59	484.871119	690.8934293	-0.51052662	0.000118633	0.00091103
ENSG00000164181	ELOVL7	829.9003401	1196.827868	-0.528852268	0.000118828	0.000912244
ENSG00000236552	RPL13AP5	192.0369755	318.2949719	-0.728072925	0.000118903	0.000912534
ENSG00000267288	AC138150.2	53.25186293	131.4550101	-1.302406211	0.000119003	0.000913012
ENSG00000124126	PREX1	31.95095907	73.75592846	-1.209677212	0.000119062	0.000913178
ENSG00000156603	MED19	283.7165114	409.8951642	-0.53085679	0.000119303	0.000914745
ENSG00000112425	EPM2A	100.3913922	170.3284824	-0.764016619	0.000120404	0.00092268
ENSG00000171314	PGAM1	12028.61634	7881.700496	0.609792292	0.000120425	0.00092268
ENSG00000188295	ZNF669	307.2346633	202.5589355	0.601146176	0.000120451	0.00092268
ENSG00000175505	CLCF1	388.7361622	230.9450611	0.74907782	0.000120489	0.000922684
ENSG00000074370	ATP2A3	0.741249728	17.75038443	-4.606960949	0.000121002	0.000926327
ENSG00000175354	PTPN2	1654.668676	2219.150071	-0.423551196	0.000121665	0.000931111
ENSG00000162006	MSLNL	12.35643758	57.1539184	-2.205370699	0.000121962	0.000933088
ENSG00000123146	ADGRE5	5521.549337	4356.475713	0.341855761	0.000122109	0.000933921
ENSG00000196458	ZNF605	429.1980858	256.1152572	0.747106483	0.000122262	0.000934803
ENSG00000048140	TSPAN17	2252.046557	1591.332576	0.500422216	0.000122387	0.000935465
ENSG00000164073	MFSD8	723.2778211	975.7576129	-0.43206587	0.000122541	0.000936355
ENSG00000123384	LRP1	2609.948898	1798.287989	0.537946012	0.000122801	0.000938045
ENSG00000196715	VKORC1L1	1965.818209	2627.606337	-0.418925104	0.000123139	0.000940182
ENSG00000281344	HELLPAR	125.2999195	212.2966039	-0.760928433	0.000123157	0.000940182
ENSG00000185246	PRPF39	1418.625688	1851.237021	-0.383947631	0.000124221	0.000948014
ENSG00000048471	SNX29	911.2781398	664.1859484	0.455682776	0.000124755	0.000951791
ENSG00000125354	6-Sep	585.9878822	816.5529397	-0.479239839	0.000125126	0.000954326
ENSG00000170017	ALCAM	1211.30452	1655.753065	-0.451084488	0.000125468	0.000956636
ENSG00000149485	FADS1	1074.279049	1454.06991	-0.437219383	0.000125566	0.000957082
ENSG00000137857	DUOX1	325.1592696	193.2529415	0.75391852	0.000125835	0.000958825
ENSG00000123643	SLC36A1	1000.748978	679.8654283	0.55663635	0.000125872	0.000958825
ENSG00000072071	ADGRL1	4490.811221	3371.993549	0.413260147	0.00012659	0.000963811
ENSG00000134323	MYCN	5.831058735	30.44680198	-2.388987926	0.000126605	0.000963811
ENSG00000273230	AC102953.2	95.75647806	219.2118392	-1.192650625	0.000126811	0.000965077
ENSG00000132819	RBM38	1499.508644	1052.097725	0.510452456	0.000127109	0.000967044
ENSG00000186395	KRT10	598.7359523	377.4637271	0.664332553	0.000127326	0.000968393
ENSG00000136231	IGF2BP3	2107.838547	2744.339497	-0.380696111	0.000127399	0.000968653
ENSG00000011258	MBTD1	617.3896331	866.5978048	-0.489230705	0.000127491	0.000969047

ENSG00000128254	C22orf24	12.69780712	44.39426769	-1.799933863	0.000127871	0.000971416
ENSG00000105997	HOXA3	365.3708373	244.4900649	0.579226209	0.000127881	0.000971416
ENSG00000223842	AL360093.1	34.00648212	8.583118012	1.994937269	0.000129138	0.00098066
ENSG00000114779	ABHD14B	2646.060364	2001.265097	0.402720171	0.000129187	0.000980728
ENSG00000135898	GPR55	20.67968068	3.104695052	2.760777483	0.000129592	0.000983498
ENSG00000186212	SOWAHB	252.4541506	403.6961335	-0.67571586	0.000129738	0.000984304
ENSG00000167967	E4F1	1348.0473	1898.295849	-0.493712126	0.000129891	0.000985111
ENSG00000171786	NHLH1	38.96738605	12.13486268	1.685998685	0.000129925	0.000985111
ENSG00000122406	RPL5	23588.30377	30304.73772	-0.361460631	0.000130637	0.000990207
ENSG00000128928	IVD	1989.162587	2584.550206	-0.377627525	0.000131248	0.000994529
ENSG00000166619	BLCAP	1817.000149	1335.017027	0.445248755	0.000131312	0.000994708
ENSG00000063177	RPL18	18651.40677	24120.98457	-0.370994869	0.000132029	0.000999705
ENSG00000136098	NEK3	585.8174384	389.3080804	0.589173864	0.000132053	0.000999705
ENSG00000137936	BCAR3	620.273761	445.36646	0.478559955	0.000132318	0.001001398
ENSG00000169871	TRIM56	4108.802403	3227.949912	0.348178868	0.000132366	0.001001456
ENSG00000114631	PODXL2	2410.57398	1794.803983	0.42523944	0.000132938	0.001005471
ENSG00000221995	TIAF1	200.0351848	121.8764088	0.712556447	0.0001342	0.001014706
ENSG00000152104	PTPN14	5706.109146	4448.237997	0.359238378	0.000134991	0.001020372
ENSG00000130255	RPL36	8600.274689	11356.08279	-0.401059444	0.000135509	0.001023702
ENSG00000205918	PDPK2P	86.30367485	169.4709965	-0.972283582	0.000135515	0.001023702
ENSG00000068137	PLEKHH3	1605.926744	2200.588791	-0.45435216	0.000135898	0.001026279
ENSG00000122873	CISD1	470.9199154	665.9835807	-0.500849676	0.000136059	0.001027176
ENSG00000182240	BACE2	681.7167799	970.5239014	-0.509761943	0.000136484	0.001030068
ENSG00000188313	PLSCR1	601.7380688	834.8913678	-0.473176	0.000136589	0.001030546
ENSG00000198420	TCAF1	1475.172464	2054.948382	-0.478556342	0.000136863	0.001032293
ENSG00000215807	KRT18P65	160.9569759	87.67049408	0.877192283	0.000137279	0.001035118
ENSG00000119487	MAPKAP1	3057.774514	2278.872483	0.423761926	0.000137627	0.001037418
ENSG00000198633	ZNF534	13.10164857	0.277559048	5.333025361	0.00013774	0.001037955
ENSG00000142765	SYTL1	2222.106913	3491.927253	-0.651941335	0.000137794	0.001038034
ENSG00000198843	SELENOT	1189.227962	1563.356536	-0.394371933	0.000137843	0.001038034
ENSG00000119608	PROX2	34.65798524	7.933677756	2.132828326	0.000137877	0.001038034
ENSG00000113716	HMGXB3	3185.374633	2255.625371	0.497552107	0.000138455	0.001042059
ENSG00000139971	ARMH4	55.1058605	21.64497999	1.355290925	0.000138562	0.001042551
ENSG00000129295	LRRC6	147.8724423	69.37382603	1.098499003	0.000139279	0.001047622
ENSG00000182584	ACTL10	97.26260491	177.4496789	-0.86774966	0.00013968	0.001050318
ENSG00000235652	AL356599.1	32.43677598	75.59163332	-1.219671669	0.000140193	0.001053848
ENSG00000109854	HTATIP2	3822.676221	2653.017163	0.526611696	0.000140404	0.001054996
ENSG00000186468	RPS23	6894.502638	9030.949784	-0.389495171	0.000140431	0.001054996
ENSG00000181026	AEN	2082.553804	1177.442163	0.821991577	0.000140708	0.00105675
ENSG00000138002	IFT172	665.8074196	917.8418334	-0.462381174	0.000141233	0.001060371
ENSG00000213859	KCTD11	1065.247676	715.2998237	0.575653078	0.000141399	0.001061295
ENSG00000003402	CFLAR	2590.750335	3501.244358	-0.434207702	0.000141757	0.001063654
ENSG00000141391	PRELID3A	425.1205016	591.4556412	-0.476965601	0.000141816	0.001063771

ENSG00000070759	TESK2	177.0001585	272.6029969	-0.624532498	0.000141907	0.00106413
ENSG00000033627	ATP6V0A1	4440.370397	3205.970118	0.470176544	0.000142847	0.001070849
ENSG00000229312	AL353693.1	15.97547954	1.272338916	3.706889591	0.000143211	0.001073252
ENSG00000154803	FLCN	1297.580912	986.7497693	0.395379375	0.000143267	0.001073255
ENSG00000104324	CPQ	263.8605325	140.1108861	0.917041695	0.000143299	0.001073255
ENSG00000075618	FSCN1	5542.197471	3707.568951	0.579787401	0.000143432	0.001073923
ENSG00000135365	PHF21A	2727.354638	1948.641495	0.485478748	0.000143703	0.001075627
ENSG00000168028	RPSA	35627.03716	46710.90794	-0.390806637	0.000144028	0.001077731
ENSG00000105220	GPI	14824.72129	9311.453207	0.670825007	0.000144887	0.00108383
ENSG00000186198	SLC51B	7.953177538	40.98401264	-2.360875541	0.00014502	0.00108449
ENSG00000156463	SH3RF2	1553.529031	1136.417315	0.450412579	0.000145328	0.001086461
ENSG00000198231	DDX42	6362.605	7969.946437	-0.325008236	0.00014548	0.001087271
ENSG00000105705	SUGP1	1101.493147	1509.117457	-0.453637768	0.000145535	0.001087347
ENSG00000265554	AP005271.1	23.11716429	3.341966181	2.79639282	0.000147433	0.001101192
ENSG00000185697	MYBL1	653.3794832	427.8935961	0.609399482	0.00014755	0.001101448
ENSG00000274414	AL121772.1	14.63488753	50.03772483	-1.775639201	0.000147557	0.001101448
ENSG00000176087	SLC35A4	2746.970006	1948.467015	0.495089813	0.000147823	0.001103102
ENSG00000166333	ILK	29.15133242	68.80307224	-1.239414029	0.000148248	0.001105939
ENSG00000142669	SH3BGRL3	2025.252853	1284.406086	0.656310293	0.000148724	0.001109148
ENSG00000117000	RLF	1883.160688	1298.271649	0.536374126	0.000148792	0.001109194
ENSG00000142530	FAM71E1	254.9956531	152.8437218	0.741955959	0.00014882	0.001109194
ENSG00000015133	CCDC88C	1012.133479	1421.311374	-0.490198916	0.000149633	0.001114912
ENSG00000170166	HOXD4	6.560576058	27.77138385	-2.084373134	0.000151346	0.001127334
ENSG00000146966	DENND2A	51.52740957	109.1682126	-1.078756967	0.000151946	0.001131464
ENSG00000214756	CSKMT	112.3203609	191.6821571	-0.77294886	0.000151999	0.001131514
ENSG00000251322	SHANK3	1036.677478	782.45736	0.405612547	0.0001525	0.001134898
ENSG00000112782	CLIC5	22.76152242	69.40482401	-1.601311791	0.000154273	0.001147741
ENSG00000056972	TRAF3IP2	755.4225316	1023.036882	-0.437509341	0.000154359	0.001148038
ENSG00000035141	FAM136A	2631.151253	3470.555488	-0.399698803	0.000154561	0.001149189
ENSG00000137210	TMEM14B	1425.660032	1058.048515	0.429857999	0.000154892	0.001151304
ENSG00000088881	EBF4	214.67959	372.593584	-0.793653058	0.000155071	0.001152282
ENSG00000005075	POLR2J	958.710654	696.1659833	0.460761725	0.000155321	0.001153796
ENSG00000164463	CREBRF	604.2338863	934.715305	-0.628361779	0.000156035	0.001158748
ENSG00000141569	TRIM65	1255.566248	1689.457765	-0.428717004	0.000156221	0.001159774
ENSG00000225572	DOCK4-AS1	8.784325241	0	5.716670047	0.000156335	0.00115993
ENSG00000136048	DRAM1	469.6883997	657.0497414	-0.484536972	0.000156336	0.00115993
ENSG00000119514	GALNT12	1262.748532	1700.037636	-0.428969029	0.000156394	0.00116001
ENSG00000129946	SHC2	5.739890795	30.83476163	-2.412268778	0.000157978	0.00117128
ENSG00000115561	CHMP3	1728.883384	1306.330527	0.403811352	0.000158009	0.00117128
ENSG00000157045	NTAN1	709.9422925	471.8472765	0.587731109	0.00015806	0.001171303
ENSG00000147234	FRMPD3	75.95614466	153.2947068	-1.010240409	0.00015889	0.001176794
ENSG00000119906	SLF2	1425.567109	1081.399085	0.398767813	0.000158897	0.001176794
ENSG00000145425	RPS3A	18621.26592	23454.64489	-0.332909076	0.000159535	0.001181167

ENSG00000158457	TSPAN33	256.0359461	384.1406376	-0.586572703	0.000161179	0.001192975
ENSG00000145817	YIPF5	1428.430965	1091.792069	0.38755092	0.000161773	0.001197011
ENSG00000111667	USP5	4560.972328	3092.34877	0.560332856	0.000161891	0.001197528
ENSG00000256087	ZNF432	393.0902472	237.2301665	0.729121519	0.000161975	0.001197784
ENSG00000066923	STAG3	273.5836717	420.9672844	-0.620381595	0.0001623	0.001199722
ENSG00000072609	CHFR	22.20641846	4.223778441	2.394424638	0.000162335	0.001199722
ENSG00000268403	AC132192.2	40.65838871	88.86240103	-1.127044442	0.000162437	0.001200121
ENSG00000153989	NUS1	2614.888662	3480.385212	-0.41270674	0.000162518	0.001200352
ENSG00000185499	MUC1	180.001776	107.6227054	0.741493198	0.000162891	0.00120275
ENSG00000198464	ZNF480	901.6106715	637.184231	0.501432159	0.000163114	0.001203782
ENSG00000213965	NUDT19	1197.393282	1652.121812	-0.465033171	0.000163129	0.001203782
ENSG00000114805	PLCH1	268.8697874	431.1555075	-0.682061943	0.000163512	0.001206235
ENSG00000162614	NEXN	16.01971786	48.17348524	-1.585329698	0.00016356	0.001206235
ENSG00000116266	STXBP3	1181.267633	1531.123008	-0.374209056	0.000165056	0.0012169
ENSG00000138100	TRIM54	201.4602809	36.49883188	2.467212012	0.000165509	0.001219876
ENSG00000100003	SEC14L2	284.6795695	185.2624802	0.618914658	0.000166068	0.001223627
ENSG00000198258	UBL5	1800.521244	1346.388869	0.419046529	0.000166682	0.001227785
ENSG00000114770	ABCC5	1766.627536	1341.411608	0.397366797	0.000167386	0.001232602
ENSG00000106012	IQCE	1757.254092	2518.780596	-0.51900901	0.000167537	0.001233042
ENSG00000273899	NOL12	250.1189506	160.6674417	0.639231994	0.000167546	0.001233042
ENSG00000154059	IMPACT	1389.316555	1811.325965	-0.382629494	0.000169282	0.001245444
ENSG00000116754	SRSF11	8103.122641	10133.01499	-0.322475067	0.000169516	0.001246707
ENSG00000148303	RPL7A	36382.35086	46179.15939	-0.344001699	0.000169555	0.001246707
ENSG00000163818	LZTFL1	847.5116167	1238.68835	-0.546771459	0.000172167	0.001265533
ENSG00000143520	FLG2	14.57368558	0.577711617	4.603514758	0.000172808	0.001269859
ENSG00000263956	NBPF11	466.215703	655.1460083	-0.490376755	0.000173212	0.001272453
ENSG00000179388	EGR3	148.0381285	65.05297604	1.179778362	0.000174064	0.001278325
ENSG00000111605	CPSF6	3824.133492	4923.902301	-0.364832514	0.000174218	0.001279001
ENSG00000143457	GOLPH3L	1104.365077	1471.53545	-0.413813701	0.00017426	0.001279001
ENSG00000151929	BAG3	2593.971726	1821.541695	0.509467515	0.000174457	0.001280067
ENSG00000125970	RALY	5428.418877	4160.971106	0.38343036	0.000174774	0.001282004
ENSG00000255248	MIR100HG	12.39813598	0.331593289	5.252821295	0.000175106	0.001284057
ENSG00000123570	RAB9B	75.27594551	32.01182733	1.243104341	0.000175484	0.0012862
ENSG00000116299	KIAA1324	57.96922903	21.24311742	1.459467071	0.000175503	0.0012862
ENSG00000132912	DCTN4	3039.133555	3844.593769	-0.339282231	0.000175838	0.001288268
ENSG00000258441	LINC00641	489.0176651	798.860183	-0.707575405	0.000176611	0.001293547
ENSG00000009413	REV3L	1756.251915	2336.7556	-0.412120714	0.000177353	0.001298596
ENSG00000247596	TWF2	993.6323309	1345.989861	-0.438131809	0.000177822	0.001301639
ENSG00000266302	AC098850.3	30.12455015	70.62219036	-1.22602696	0.000177885	0.001301715
ENSG00000272888	LINC01578	794.8248632	1147.86585	-0.529488681	0.000178165	0.001303374
ENSG00000274214	AP005212.2	16.81693831	1.563644287	3.460074546	0.000178252	0.001303624
ENSG00000100345	MYH9	44661.58398	34155.66305	0.386893481	0.000178305	0.001303624
ENSG00000198517	MAFK	1415.350079	1048.996209	0.431417865	0.00017858	0.001305245

ENSG00000185825	BCAP31	5742.581328	4214.934998	0.445960484	0.000179729	0.001313253
ENSG00000006282	SPATA20	4919.764818	3367.66756	0.547117042	0.000180032	0.001315077
ENSG00000259570	AC243562.1	0.698281368	18.43513518	-4.669563254	0.000180092	0.001315121
ENSG00000250722	SELENOP	136.4656063	231.9571184	-0.762921725	0.000180758	0.001319589
ENSG00000100575	TIMM9	512.3138431	723.4205951	-0.497084047	0.000181329	0.001323371
ENSG00000248112	AC108174.1	23.36324204	4.537677333	2.364884035	0.000182259	0.001329762
ENSG00000086712	TXLNG	933.0908765	1320.166042	-0.501114512	0.00018328	0.001336813
ENSG00000138336	TET1	44.78940367	99.84088942	-1.15679412	0.000183383	0.001337165
ENSG00000251669	FAM86EP	300.0855213	193.8432661	0.62838181	0.000183744	0.001339398
ENSG00000165795	NDRG2	333.5985892	481.0093051	-0.527527445	0.000184464	0.001344247
ENSG00000058063	ATP11B	2820.864104	3710.976715	-0.395732934	0.00018503	0.001347904
ENSG00000074964	ARHGEF10L	991.9419562	1568.230754	-0.660415046	0.000185075	0.001347904
ENSG00000183542	KLRC4	24.42889768	4.891864145	2.32921262	0.000185269	0.001348917
ENSG00000115594	IL1R1	243.1624444	133.9387484	0.857071547	0.000186232	0.001355521
ENSG00000005206	SPPL2B	2966.953981	4471.951804	-0.591721947	0.000186843	0.001359566
ENSG00000130598	TNNI2	6.04606312	33.68258905	-2.459977382	0.0001871	0.001361037
ENSG00000251148	AL158068.2	15.65503488	0.963339148	4.089298658	0.000187267	0.001361846
ENSG00000169629	RGPD8	72.1768103	132.3252913	-0.873246185	0.000187825	0.001365502
ENSG00000136709	WDR33	3485.386581	4567.984868	-0.390382976	0.000188447	0.001369617
ENSG00000124097	HMGB1P1	25.87619992	62.05384803	-1.264304371	0.000189705	0.001378347
ENSG00000253641	LINCR-0001	66.55947477	28.78239875	1.20590705	0.000190342	0.001382566
ENSG00000132613	MTSS1L	1040.077854	752.5766731	0.466216893	0.000193637	0.001406085
ENSG00000133935	ERG28	3035.106873	2356.312436	0.365194045	0.000193767	0.001406619
ENSG00000142733	MAP3K6	1176.608952	805.5778526	0.545772305	0.000194114	0.001408719
ENSG00000038274	MAT2B	2189.770002	2995.719471	-0.45239312	0.000194319	0.001409792
ENSG00000242193	CRYZL2P	0	9.093049064	-5.501964244	0.00019472	0.001412281
ENSG00000175224	ATG13	3772.703435	2886.880554	0.386073517	0.000194809	0.001412506
ENSG00000196642	RABL6	5434.859959	6990.338415	-0.363137209	0.000195154	0.001414297
ENSG00000166886	NAB2	3456.940871	2316.117565	0.577441497	0.000195171	0.001414297
ENSG00000179277	MEIS3P1	0	9.147083305	-5.509166578	0.000195363	0.001415275
ENSG00000247556	OIP5-AS1	4916.330983	6174.659738	-0.328755291	0.000195625	0.00141675
ENSG00000121361	KCNJ8	2562.859509	1807.369078	0.504265732	0.000196347	0.001421563
ENSG00000204060	FOXO6	249.0187257	154.0542086	0.695566521	0.00019769	0.001430865
ENSG00000088992	TESC	1431.944217	1017.477231	0.492090985	0.000197878	0.001431802
ENSG00000177963	RIC8A	2161.899001	2842.414861	-0.394982879	0.000197992	0.001432206
ENSG00000105372	RPS19	17710.85033	22419.30126	-0.3401284	0.000198532	0.001435684
ENSG00000091536	MYO15A	26.59667614	79.20909984	-1.572037803	0.000200103	0.001446625
ENSG00000186184	POLR1D	3469.516353	4556.153529	-0.392908897	0.000200587	0.001449697
ENSG00000209082	MT-TL1	107.3120479	223.4057273	-1.055121353	0.000200775	0.001450628
ENSG00000272779	AC245060.4	916.9173338	679.7992112	0.431112514	0.000201072	0.001452343
ENSG00000255966	AC006064.3	30.40202645	7.990200711	1.915590395	0.000201566	0.001455485
ENSG00000128284	APOL3	0	9.101896262	-5.503065368	0.000203326	0.001467761
ENSG00000189430	NCR1	0	9.155930504	-5.510285437	0.000203451	0.001468232

ENSG00000180061	TMEM150B	0.349140684	13.86455776	-5.144970087	0.000203864	0.001470618
ENSG00000236347	AL513123.1	37.42368465	11.93787946	1.658371824	0.000203901	0.001470618
ENSG00000197594	ENPP1	45.23891599	91.04302824	-1.008117876	0.000204311	0.001473141
ENSG00000114790	ARHGEF26	347.6055898	530.3439342	-0.609602149	0.000204542	0.001474374
ENSG00000210135	MT-TN	5643.877706	7517.057218	-0.413558419	0.000204649	0.001474711
ENSG00000011451	WIZ	3398.555068	2658.402309	0.354129307	0.000204994	0.00147676
ENSG00000196998	WDR45	997.8529075	1494.813338	-0.582837345	0.000205685	0.001481305
ENSG00000082996	RNF13	904.6460395	1217.091241	-0.427991213	0.000206232	0.001484807
ENSG00000105640	RPL18A	19446.32338	25175.8409	-0.372551892	0.000206956	0.001489585
ENSG00000142784	WDTC1	3637.328833	2843.272138	0.355192242	0.000207053	0.001489847
ENSG00000157216	SSBP3	784.0236885	1085.371429	-0.468463128	0.000207479	0.001492477
ENSG00000081052	COL4A4	31.94572785	69.35183185	-1.118051456	0.000208073	0.001496308
ENSG00000085415	SEH1L	2963.137336	4240.739015	-0.517416261	0.000208159	0.001496493
ENSG00000253931	AC105118.1	0	9.986118933	-5.63207917	0.000208936	0.001501635
ENSG00000101608	MYL12A	7157.690527	5373.756051	0.413425038	0.000210303	0.001511015
ENSG00000111684	LPCAT3	434.2360479	291.9996602	0.5704042	0.000210377	0.001511107
ENSG00000115963	RND3	650.1003753	1052.828537	-0.696145888	0.000210551	0.001511912
ENSG00000197818	SLC9A8	1217.656444	920.3615599	0.403335101	0.000214066	0.001536707
ENSG00000228079	AC012368.2	3.613810736	21.92743816	-2.594205564	0.000214598	0.001540075
ENSG00000162174	ASRGL1	132.6797595	224.7341989	-0.762659239	0.000214991	0.001542444
ENSG00000155229	MMS19	2843.87443	3662.527765	-0.364790494	0.000215197	0.001543468
ENSG00000005812	FBXL3	1032.563275	1353.202982	-0.390239946	0.000215739	0.001546903
ENSG00000103066	PLA2G15	646.4767329	475.2064545	0.444017212	0.000215895	0.001547571
ENSG00000257446	ZNF878	75.65251226	34.43571971	1.136180481	0.000216238	0.001549582
ENSG00000170145	SIK2	1788.072195	1372.829137	0.381490441	0.00021705	0.001554944
ENSG00000172845	SP3	3474.758919	4489.12778	-0.369595257	0.000217559	0.001558135
ENSG00000185359	HGS	3716.788184	2894.2544	0.360815078	0.000217698	0.001558677
ENSG00000088543	C3orf18	363.9292652	541.3841165	-0.571975607	0.000218887	0.001566732
ENSG00000143479	DYRK3	224.5019822	138.5106413	0.693740173	0.000219301	0.001569241
ENSG00000152778	IFIT5	649.5605109	919.1660709	-0.501021705	0.000220251	0.001575574
ENSG00000090776	EFNB1	409.3010363	600.5450026	-0.553793713	0.000221368	0.001583109
ENSG00000111077	TNS2	2593.783407	1845.871069	0.491274463	0.000221494	0.00158355
ENSG00000114120	SLC25A36	5822.080573	7909.715756	-0.441991903	0.000221667	0.00158432
ENSG00000122042	UBL3	1289.574637	929.5167602	0.473188556	0.000221799	0.00158442
ENSG00000170049	KCNAB3	174.0927143	103.3282277	0.750204658	0.00022181	0.00158442
ENSG00000146242	TPBG	1780.199291	1349.033719	0.400487931	0.000221959	0.001585023
ENSG00000114520	SNX4	945.199316	1289.258861	-0.448478746	0.000223131	0.001592738
ENSG00000166855	CLPX	2216.180731	2923.608583	-0.399914042	0.000223169	0.001592738
ENSG00000123191	ATP7B	676.9366168	489.479911	0.467088438	0.000225122	0.001606212
ENSG00000108773	KAT2A	2160.732539	2881.205841	-0.415233275	0.000225779	0.00161043
ENSG00000172731	LRRC20	1382.788017	896.4325096	0.624434702	0.000226352	0.001613582
ENSG00000128655	PDE11A	52.78444219	20.69933524	1.357708254	0.000226352	0.001613582
ENSG00000259439	LINC01833	5.109312558	25.2242956	-2.311849553	0.000226653	0.001615254

ENSG00000168447	SCNN1B	0.349140684	14.80625444	-5.24084969	0.000227069	0.001617752
ENSG00000059804	SLC2A3	19710.60616	13822.34848	0.511978616	0.000227137	0.001617765
ENSG00000173193	PARP14	4895.906099	6166.127532	-0.332688247	0.000228059	0.001623861
ENSG00000074935	TUBE1	1290.967521	1789.530242	-0.470956787	0.000229011	0.001630165
ENSG00000213380	COG8	926.7064408	1260.805945	-0.443780242	0.000229511	0.001633257
ENSG00000238120	LINC01589	16.41055694	1.741982049	3.198484079	0.000229842	0.001635137
ENSG00000198792	TMEM184B	3479.407065	2672.597403	0.380363558	0.000230237	0.001637468
ENSG00000106034	CPED1	61.28051474	17.93354301	1.7815787	0.0002305	0.001638441
ENSG00000125037	EMC3	1210.116777	930.4713047	0.379098298	0.000230507	0.001638441
ENSG00000147383	NSDHL	1293.203819	906.8372074	0.511018911	0.000230965	0.001641225
ENSG00000225032	AL162586.1	96.55909232	46.60933264	1.056496383	0.00023245	0.001651299
ENSG00000188488	SERPINA5	15.49220255	1.178016755	3.694091881	0.000232851	0.001653668
ENSG00000105193	RPS16	19205.33061	24548.05651	-0.354107541	0.000233542	0.001658095
ENSG00000185522	LMNTD2	219.4191621	686.5500249	-1.64514386	0.00023367	0.00165852
ENSG00000089157	RPLP0	56359.84601	70694.71965	-0.326933934	0.00023448	0.001663793
ENSG00000121931	LRIF1	612.0077586	432.995629	0.500502356	0.000235063	0.001667445
ENSG00000108671	PSMD11	4161.628149	2728.90877	0.608465066	0.000235832	0.001672421
ENSG00000242136	AC093904.2	72.03505422	31.26763491	1.198688582	0.000236683	0.001677971
ENSG00000186907	RTN4RL2	387.8653571	267.8423031	0.533259417	0.000236812	0.001678399
ENSG00000112992	NNT	3277.630553	4244.75532	-0.372961378	0.000236919	0.001678672
ENSG00000123405	NFE2	111.4687536	56.32973675	0.991283157	0.000237323	0.001681051
ENSG00000023318	ERP44	1945.112849	2573.533825	-0.403938546	0.000237464	0.00168156
ENSG00000166484	MAPK7	617.0452329	434.801405	0.50415902	0.000237582	0.001681913
ENSG00000169851	PCDH7	1248.92798	889.4978717	0.489011541	0.000239046	0.001691792
ENSG00000274561	AC005332.3	84.27669647	39.33789041	1.104760786	0.000239617	0.00169509
ENSG00000275560	AC008115.3	40.31051798	84.92298287	-1.074380573	0.00023965	0.00169509
ENSG00000196415	PRTN3	46.28252816	15.63403246	1.57623849	0.000240063	0.001697522
ENSG00000185627	PSMD13	2191.655409	3079.664351	-0.49102475	0.000240515	0.001700223
ENSG00000167562	ZNF701	159.7088218	85.33653305	0.908511782	0.00024154	0.001706979
ENSG00000125089	SH3TC1	728.5433329	505.7237081	0.528093931	0.000241672	0.001707423
ENSG00000272692	AC010997.3	104.849353	54.25075397	0.954863694	0.000242238	0.001710931
ENSG00000104312	RIPK2	462.7379377	659.8321073	-0.512555214	0.000243441	0.001718927
ENSG00000285869	AC130456.8	0	8.922099139	-5.479003488	0.000245255	0.001731244
ENSG00000269934	AL353593.2	0	8.944692661	-5.482111715	0.000246398	0.001738807
ENSG00000100593	ISM2	10.54430099	37.42349908	-1.828025	0.000246817	0.001741264
ENSG00000250920	AC105460.1	13.83116589	0.909304906	3.932276272	0.000246998	0.001742045
ENSG00000262585	LINC01979	11.61914911	42.96618454	-1.881662649	0.000247131	0.001742478
ENSG00000197948	FCHSD1	1296.67559	954.8134493	0.442299144	0.000248289	0.001750139
ENSG00000172432	GTPBP2	3396.792418	5050.518466	-0.572166545	0.000249865	0.001760742
ENSG00000127191	TRAF2	1010.03258	1321.875931	-0.388644579	0.000251083	0.001768818
ENSG00000134717	BTF3L4	1943.55393	2453.947405	-0.33635559	0.0002512	0.001769137
ENSG00000162909	CAPN2	9041.444617	7210.769262	0.32645791	0.000251338	0.0017696
ENSG00000214842	RAD51AP2	55.97966261	20.48955676	1.455145594	0.00025171	0.001771714

ENSG00000151881	TMEM267	446.9126162	636.1092124	-0.510022496	0.00025367	0.001784997
ENSG00000255277	ABCC6P2	5.450682095	27.23147143	-2.317752704	0.000256073	0.001801391
ENSG00000244286	ITGB5-AS1	21.63466483	3.838150909	2.480440341	0.000256422	0.001803329
ENSG00000240476	LINC00973	7.957138795	0	5.576990298	0.000257244	0.001808075
ENSG00000164400	CSF2	7.957138795	0	5.576990298	0.000257244	0.001808075
ENSG00000175155	YPEL2	236.1891484	363.6874465	-0.621236255	0.00025846	0.0018161
ENSG00000157617	C2CD2	1413.421221	1819.594546	-0.364442931	0.000258911	0.001818746
ENSG00000213071	LPAL2	55.44945601	19.12435504	1.54547492	0.000258994	0.001818812
ENSG00000127528	KLF2	137.4963674	233.7516881	-0.764479265	0.000259192	0.001819681
ENSG00000187325	TAF9B	802.6383621	1146.008308	-0.514255665	0.000260137	0.001825791
ENSG00000120451	SNX19	2226.574678	1712.630687	0.378206214	0.00026037	0.001826904
ENSG00000118007	STAG1	1405.990681	1838.860105	-0.387506198	0.000261119	0.00183164
ENSG00000230661	YY1P1	58.91358815	22.57293034	1.389168944	0.000261235	0.001831928
ENSG00000229153	EPHA1-AS1	29.68693282	83.77529918	-1.49295586	0.000262759	0.001842088
ENSG00000125657	TNFSF9	227.9453408	122.8875803	0.887740682	0.000262839	0.001842127
ENSG00000185658	BRWD1	3507.321225	4588.207462	-0.387452089	0.0002635	0.001846227
ENSG00000170579	DLGAP1	2.941534102	22.51055721	-2.94214134	0.000264143	0.001850207
ENSG00000099769	IGFALS	64.5650023	27.83177657	1.221913186	0.000265132	0.001856607
ENSG00000101246	ARFRP1	1277.834191	961.9161824	0.409781827	0.000265379	0.001857801
ENSG00000250565	ATP6V1E2	485.3892793	345.0079	0.491891033	0.000266039	0.001861892
ENSG00000253477	AC012213.1	16.68676327	2.141355904	2.975389396	0.000266433	0.001864122
ENSG00000196639	HRH1	264.1261026	172.0999375	0.61582704	0.000266545	0.001864374
ENSG00000146090	RASGEF1C	78.92749339	29.70552828	1.400160858	0.000266905	0.001866359
ENSG00000006606	CCL26	171.0300462	102.4651265	0.73973531	0.000267328	0.001868784
ENSG00000100105	PATZ1	2266.338337	2914.215457	-0.36259739	0.000268822	0.001878695
ENSG00000175105	ZNF654	907.5452473	648.5688818	0.485710887	0.000269795	0.001884954
ENSG00000115761	NOL10	1632.046196	1209.23096	0.431891173	0.000271926	0.001899306
ENSG00000163508	EOMES	10.33342045	44.45043894	-2.097202186	0.000273032	0.001906485
ENSG00000034152	MAP2K3	1498.545143	1017.692796	0.557566183	0.000277393	0.001935954
ENSG00000203999	LINC01270	5.421986067	26.12767203	-2.262921911	0.00027741	0.001935954
ENSG00000069275	NUCKS1	14869.06895	19994.23706	-0.427302619	0.00027916	0.001947616
ENSG00000182628	SKA2	1528.068105	2141.30534	-0.487060706	0.000279252	0.001947704
ENSG00000147804	SLC39A4	1102.612533	783.3913557	0.493600761	0.00027954	0.001949158
ENSG00000102393	GLA	1201.691405	689.4526174	0.800347249	0.00028018	0.001953065
ENSG00000123096	SSPN	52.2957714	20.7936574	1.338920102	0.000280387	0.001953956
ENSG00000254986	DPP3	2183.552844	1518.489538	0.523525311	0.000280858	0.001956681
ENSG00000105173	CCNE1	670.7507432	476.5429391	0.492407974	0.000281263	0.001958945
ENSG00000180998	GPR137C	213.5563797	136.9091851	0.640598256	0.000282012	0.001963606
ENSG00000176092	CRYBG2	3425.239374	2084.206575	0.716314153	0.00028345	0.001973059
ENSG00000171103	TRMT61B	536.798235	382.35683	0.48819124	0.000285187	0.00198459
ENSG00000133247	KMT5C	1007.552302	1318.328603	-0.387743501	0.000286051	0.001990038
ENSG00000173442	EHBP1L1	865.9827961	1255.709567	-0.536208683	0.000286968	0.001995849
ENSG00000197122	SRC	1516.527876	2014.975345	-0.41027207	0.000287581	0.001999164

ENSG00000272602	ZNF595	11.61914911	0.277559048	5.160386493	0.000287607	0.001999164
ENSG00000136840	ST6GALNAC4	760.1387791	1103.244147	-0.537851445	0.000288531	0.002005017
ENSG00000109089	CDR2L	866.4210106	1273.786033	-0.556576733	0.000289729	0.002012634
ENSG00000170291	ELP5	938.4487843	1275.218921	-0.442629514	0.000289868	0.002012634
ENSG00000149922	TBX6	27.39389659	66.68833623	-1.287514395	0.000289873	0.002012634
ENSG00000130159	ECSIT	672.6635542	940.9027787	-0.484672317	0.000290518	0.002016539
ENSG00000276386	CNTNAP3P2	45.07211119	16.74280929	1.422985247	0.000291439	0.002022365
ENSG00000146592	CREB5	31.16150977	9.285133149	1.742830596	0.000292356	0.002028152
ENSG00000005243	COPZ2	190.4603028	119.4196163	0.672756934	0.000295511	0.002049459
ENSG00000133612	AGAP3	2634.707029	3338.641284	-0.341646874	0.000296942	0.002058801
ENSG00000182366	FAM87A	6.990422241	29.18572068	-2.072000553	0.000299929	0.002078926
ENSG00000147050	KDM6A	1059.707893	1404.38658	-0.406786563	0.000300425	0.002081511
ENSG00000148483	TMEM236	47.18122762	18.66458846	1.337254658	0.000300471	0.002081511
ENSG00000108039	XPNPEP1	3123.812218	2485.835854	0.329427393	0.000301354	0.002087039
ENSG00000166816	LDHD	20.9351135	61.24609652	-1.542777058	0.00030154	0.002087736
ENSG00000132357	CARD6	70.55747203	32.81504416	1.102910732	0.000303677	0.00210194
ENSG00000069424	KCNAB2	1726.186767	1264.829899	0.449204635	0.000304126	0.002104455
ENSG00000184898	RBM43	367.2292614	557.426215	-0.601447933	0.000304521	0.002106592
ENSG00000159228	CBR1	1429.369914	1016.606377	0.49097861	0.000308122	0.002130902
ENSG00000128272	ATF4	21400.35896	28773.38292	-0.427079417	0.000309033	0.002136602
ENSG00000186222	BLOC1S4	777.2806252	550.1766883	0.497560065	0.000309718	0.002140525
ENSG00000145741	BTF3	12739.2945	16097.64936	-0.337577605	0.000309801	0.002140525
ENSG00000120075	HOXB5	1303.48685	2295.520842	-0.816092694	0.000309862	0.002140525
ENSG00000187837	HIST1H1C	499.1920294	317.1594985	0.654973865	0.000311475	0.002151058
ENSG00000168286	THAP11	395.6999294	554.9173286	-0.488512347	0.000311842	0.002152993
ENSG00000143458	GABPB2	818.4547968	1151.450247	-0.491885684	0.000313867	0.002166365
ENSG00000170498	KISS1	14.40181214	1.209457476	3.578874879	0.000314502	0.002170136
ENSG00000255774	AP000439.2	33.03738823	71.5099311	-1.113540404	0.000315766	0.002178244
ENSG00000172197	MBOAT1	224.6058498	341.6599143	-0.604501369	0.000318541	0.002196769
ENSG00000154767	XPC	1030.199505	1331.100776	-0.369536388	0.000318719	0.002196918
ENSG00000032742	IFT88	456.3368747	617.9458262	-0.437250143	0.000318742	0.002196918
ENSG00000159713	TPPP3	5.806323963	25.53080666	-2.136945625	0.00032185	0.002217722
ENSG00000177548	RABEP2	1216.412753	916.2130539	0.409054314	0.000322923	0.00222412
ENSG00000166582	CENPV	1034.715663	1594.428517	-0.624392195	0.00032296	0.00222412
ENSG00000224051	CPTP	1259.552816	962.14733	0.388023904	0.000323877	0.00222981
ENSG00000100462	PRMT5	3117.087994	2398.871897	0.377528223	0.000324447	0.002233105
ENSG00000261040	WFDC21P	13.86890303	41.19826024	-1.577057959	0.000325598	0.0022404
ENSG00000162512	SDC3	2177.150043	1670.745971	0.38172654	0.000327442	0.002252458
ENSG00000015413	DPEP1	1938.42764	2768.369697	-0.51378731	0.000327797	0.002254272
ENSG00000060339	CCAR1	3350.870825	4506.831974	-0.427733828	0.000328044	0.002255337
ENSG00000213801	ZNF321P	116.0166236	185.7389901	-0.678103833	0.000328881	0.00226046
ENSG00000181467	RAP2B	1169.7877	854.2144003	0.452752607	0.000329572	0.002264571
ENSG00000167526	RPL13	36871.1564	45738.46639	-0.310907993	0.000330479	0.002270172

ENSG00000109084	TMEM97	1701.676556	2241.342195	-0.39767281	0.000331361	0.00227559
ENSG00000131187	F12	343.5124521	222.0710678	0.626717843	0.00033173	0.002277493
ENSG00000138496	PARP9	943.5149884	1250.363812	-0.406553186	0.000331883	0.002277902
ENSG00000003056	M6PR	2888.87297	2254.440435	0.357506537	0.00033275	0.002283214
ENSG00000143994	ABHD1	84.18171864	152.390822	-0.853300009	0.000335656	0.002302509
ENSG00000162437	RAVER2	1634.443577	2136.900419	-0.386412391	0.00033622	0.002305736
ENSG00000244560	AC004890.2	556.6142621	339.5240826	0.715247019	0.000336541	0.00230729
ENSG00000170619	COMMD5	743.7017633	520.8439395	0.512575429	0.000337091	0.002310419
ENSG00000151834	GABRA2	0	10.2287975	-5.664106724	0.000337849	0.002314971
ENSG00000173156	RHOD	1219.777411	881.8624444	0.467038245	0.000338	0.002315356
ENSG00000210144	MT-TY	241.5293328	379.8456774	-0.651412021	0.000338679	0.002319358
ENSG00000232079	LINC01697	14.44224057	0.940745627	3.98409166	0.000340329	0.00233001
ENSG00000187650	VMAC	147.7367221	250.0105086	-0.757914889	0.000340571	0.00233102
ENSG00000112701	SENP6	3064.443701	3817.570265	-0.317100423	0.000342098	0.002340818
ENSG00000172809	RPL38	5924.612151	7712.764522	-0.380558953	0.000342425	0.002342401
ENSG00000187951	AC091057.1	212.7184168	345.0981573	-0.700049865	0.000344617	0.002356742
ENSG00000197479	PCDHB11	48.85145677	19.19462432	1.355069166	0.000350681	0.002397428
ENSG00000136051	WASHC4	2068.904497	2737.49973	-0.404163538	0.000350762	0.002397428
ENSG00000260532	AL031598.1	21.41569919	3.50801698	2.653105795	0.000351588	0.002402404
ENSG00000268460	AC006262.1	168.9843465	75.011863	1.177242394	0.000352419	0.00240614
ENSG00000151348	EXT2	2001.228391	2634.032372	-0.396546827	0.000352425	0.00240614
ENSG00000144550	CPNE9	44.61990757	14.35928313	1.629920391	0.000352429	0.00240614
ENSG00000178096	BOLA1	245.7862364	159.6826167	0.619956792	0.000355328	0.002424118
ENSG00000170035	UBE2E3	1477.575093	1112.538976	0.408639604	0.000355332	0.002424118
ENSG00000171121	KCNMB3	185.7380195	109.1460491	0.771208984	0.00035538	0.002424118
ENSG00000152284	TCF7L1	178.8191103	273.9748961	-0.613531767	0.000355457	0.002424118
ENSG00000175376	EIF1AD	867.5382104	592.3483209	0.549319371	0.000356234	0.002428745
ENSG00000131051	RBM39	8530.215333	11301.32674	-0.405748451	0.000357903	0.002439447
ENSG00000110693	SOX6	31.56535121	69.47656538	-1.134395745	0.000358418	0.002442277
ENSG00000166200	COPS2	2798.522297	3545.77416	-0.341629864	0.000361354	0.002460944
ENSG00000185798	WDR53	373.0984078	254.8993372	0.547945446	0.0003614	0.002460944
ENSG00000280734	LINC01232	258.6145325	379.7210094	-0.552817261	0.000361458	0.002460944
ENSG00000173898	SPTBN2	2652.372073	3435.014548	-0.372890941	0.000364573	0.002481459
ENSG00000173218	VANGL1	2849.104414	2128.298618	0.420495569	0.000365983	0.002490371
ENSG00000105856	HBP1	958.3436049	1271.5398	-0.407400372	0.000368728	0.00250835
ENSG00000163960	UBXN7	3498.014148	4398.172429	-0.330312564	0.000369386	0.002512132
ENSG00000198496	NBR2	272.7109657	527.465667	-0.950982515	0.000370416	0.002518439
ENSG00000181381	DDX60L	493.2734725	680.4017975	-0.463110492	0.000370548	0.002518641
ENSG00000111726	CMAS	1715.265311	2418.37258	-0.495784934	0.000371879	0.002526988
ENSG00000131019	ULBP3	88.43768865	152.0735871	-0.783141749	0.000374014	0.002540788
ENSG00000234996	AC098934.2	52.27499789	101.2207885	-0.952382268	0.000374413	0.0025428
ENSG00000165621	OXGR1	50.97991417	20.27831891	1.331015424	0.000375174	0.002547265
ENSG00000157107	FCHO2	839.8004155	1128.920668	-0.426707608	0.000375534	0.002549003

ENSG00000269289	AC011503.1	7.670470021	0	5.521526811	0.000377434	0.002561188
ENSG00000166716	ZNF592	2265.491964	2899.457832	-0.356070454	0.000378592	0.002568335
ENSG00000155265	GOLGA7B	259.0033223	153.0362359	0.762549973	0.000378715	0.002568363
ENSG00000111490	TBC1D30	1127.495701	667.1364637	0.755849285	0.000378805	0.002568363
ENSG00000162600	OMA1	505.6484688	687.0587837	-0.441869309	0.000378995	0.002568943
ENSG00000120471	TP53AIP1	17.87355284	2.705321197	2.720314222	0.000379476	0.002571495
ENSG00000130165	ELOF1	1245.830586	945.4338499	0.397378578	0.000381171	0.002582267
ENSG00000172215	CXCR6	72.6716571	31.10604049	1.219207574	0.000381481	0.002583654
ENSG00000159445	THEM4	345.3994463	487.8194558	-0.499067449	0.000383544	0.002596908
ENSG00000143624	INTS3	4217.706293	6034.187885	-0.516547024	0.000385359	0.002608477
ENSG00000183463	URAD	29.30513485	66.57375266	-1.179295136	0.00038728	0.002620757
ENSG00000153064	BANK1	68.87709441	31.79569036	1.123170315	0.000387697	0.002622857
ENSG00000136695	IL36RN	4.25739134	27.38523527	-2.663712786	0.000388611	0.002628314
ENSG00000166707	ZCCHC18	59.39432521	25.45417889	1.217628069	0.0003893	0.002632249
ENSG00000167770	OTUB1	2764.197593	2204.518999	0.326314852	0.000389633	0.002633777
ENSG00000147852	VLDLR	1005.615199	694.3430597	0.534991452	0.000390553	0.002639271
ENSG00000101276	SLC52A3	364.6261141	519.5420893	-0.510329831	0.000390963	0.002641312
ENSG00000204175	GPRIN2	634.7798836	857.9215107	-0.433819572	0.000393194	0.002655655
ENSG00000141404	GNAL	972.9943599	653.97723	0.57199807	0.000393579	0.002657525
ENSG00000010270	STARD3NL	1245.668393	932.9902879	0.416312476	0.000394667	0.002664141
ENSG00000151838	CCDC175	5.782859155	29.62135608	-2.349450199	0.000395269	0.002667468
ENSG00000213523	SRA1	1170.734459	801.034285	0.546377846	0.000396178	0.00267287
ENSG00000091140	DLD	2853.15405	3852.791601	-0.433598048	0.000396612	0.002675062
ENSG00000075239	ACAT1	2003.702681	2887.800768	-0.527610499	0.000397184	0.002678181
ENSG00000204136	GGTA1P	28.05571078	6.71296267	2.040564351	0.000397315	0.002678329
ENSG00000156795	WDYHV1	517.9901576	374.0468019	0.468681619	0.000398183	0.002682902
ENSG00000074047	GLI2	523.1446503	711.5238123	-0.443251617	0.000398212	0.002682902
ENSG00000164111	ANXA5	4951.702161	3875.808047	0.353214808	0.000398693	0.002685408
ENSG00000186814	ZSCAN30	1160.670859	821.6895398	0.499077891	0.000401123	0.002701035
ENSG00000100206	DMC1	26.07693198	60.55506558	-1.217028486	0.000404781	0.00272442
ENSG00000106443	PHF14	761.5816435	1001.827968	-0.395473994	0.000404818	0.00272442
ENSG00000198780	FAM169A	709.0575402	968.2311011	-0.45005688	0.000404989	0.002724826
ENSG00000230882	AC005077.4	37.75601307	12.61335301	1.579276221	0.000406232	0.002732438
ENSG00000253598	SLC10A5	48.44095155	105.2581764	-1.118065736	0.000406448	0.002733144
ENSG00000147140	NONO	13818.57531	17144.98917	-0.311191382	0.00040681	0.002734826
ENSG00000181355	OFCC1	0.741249728	15.33877901	-4.391370655	0.000407031	0.002735563
ENSG00000143365	RORC	162.2886781	76.72584409	1.085968098	0.000409168	0.002749177
ENSG00000253308	AC004080.1	12.72508182	47.9538302	-1.909657092	0.000411142	0.002761579
ENSG00000269893	SNHG8	491.1689452	697.2086595	-0.505829391	0.000411239	0.002761579
ENSG00000102316	MAGED2	3837.739045	3071.59859	0.321280217	0.000411575	0.002763082
ENSG00000135469	COQ10A	187.2438324	276.141347	-0.55990178	0.000411864	0.002764265
ENSG00000106638	TBL2	2198.451589	2835.920351	-0.367467229	0.000412608	0.002768503
ENSG00000233016	SNHG7	748.0036732	1183.41226	-0.661413543	0.000413144	0.002770504

ENSG00000103241	FOXF1	60.59681966	26.04460748	1.226975772	0.0004132	0.002770504
ENSG00000179335	CLK3	2637.286855	2040.478934	0.370367594	0.000413245	0.002770504
ENSG00000213390	ARHGAP19	504.3597237	802.988392	-0.671742187	0.000414179	0.002776007
ENSG00000119242	CCDC92	826.839382	558.7318696	0.566617521	0.000414789	0.002779338
ENSG00000167771	RCOR2	86.22979571	162.1037727	-0.906428662	0.000414912	0.0027794
ENSG00000106004	HOXA5	290.3647595	477.1085191	-0.715331968	0.000415078	0.002779752
ENSG00000134440	NARS	8186.263775	10539.689	-0.364612764	0.000418947	0.002804901
ENSG00000115271	GCA	488.3355681	697.0032607	-0.514136181	0.000419818	0.002809968
ENSG00000172497	ACOT12	7.558528567	0	5.504014688	0.000419972	0.002810227
ENSG00000164088	PPM1M	570.3805675	860.9683021	-0.59296273	0.000420411	0.002812398
ENSG00000102287	GABRE	496.9697465	306.6909796	0.698971106	0.000421234	0.002817141
ENSG00000131183	SLC34A1	20.3422724	4.000253635	2.362926729	0.000422582	0.002825383
ENSG00000152454	ZNF256	169.0561847	92.27543094	0.877669776	0.000423713	0.002832173
ENSG00000088367	EPB41L1	2104.654529	2687.908742	-0.353198555	0.000424448	0.002836314
ENSG00000006576	PHTF2	1235.775144	1726.138899	-0.482327655	0.000425025	0.002839398
ENSG00000113407	TARS	7383.277497	9412.02565	-0.350270926	0.000429244	0.002866804
ENSG00000205890	AC108134.1	138.0865285	249.7319202	-0.852050689	0.000430809	0.002876473
ENSG00000165617	DACT1	29.51870669	7.221864369	2.056055373	0.000431616	0.002881077
ENSG00000100599	RIN3	1057.811097	805.3051656	0.3928664	0.000435345	0.002905178
ENSG00000156886	ITGAD	0.349140684	11.92903227	-4.929545342	0.000435657	0.002906105
ENSG00000211451	GNRHR2	49.12607918	19.4884184	1.329901024	0.00043572	0.002906105
ENSG00000108424	KPNB1	11802.67432	15582.62525	-0.400897199	0.0004363	0.002909138
ENSG00000100744	GSKIP	828.3586738	1078.412595	-0.380864306	0.000436412	0.002909138
ENSG00000127481	UBR4	16989.17277	13105.26684	0.374398096	0.00044187	0.002944212
ENSG00000145247	OCIAD2	1802.118357	1369.365877	0.395615854	0.000441914	0.002944212
ENSG00000253729	PRKDC	15432.85595	24600.59171	-0.672725768	0.000442051	0.002944328
ENSG00000185100	ADSSL1	324.7362498	223.5903067	0.539240675	0.000444385	0.002959074
ENSG00000090686	USP48	2951.667372	3677.201209	-0.317163444	0.000444799	0.002961024
ENSG00000274370	AC130371.2	2.204245632	22.63727115	-3.36569338	0.000446123	0.002968433
ENSG00000100253	MIOX	9.133465925	38.22166018	-2.066437004	0.000446154	0.002968433
ENSG00000213906	LTB4R2	158.4468719	97.18214906	0.706191445	0.000446881	0.002972467
ENSG00000090447	TFAP4	1043.935077	1402.939292	-0.426787598	0.000448252	0.002980476
ENSG00000223685	LINC00571	26.89111606	5.434695277	2.344179697	0.000448328	0.002980476
ENSG00000182621	PLCB1	216.6352291	133.6428173	0.698959299	0.000449051	0.002984477
ENSG00000154781	CCDC174	703.4716368	923.6126694	-0.3923557	0.000449864	0.002989068
ENSG00000167747	C19orf48	5164.093568	6629.233021	-0.360273455	0.000450164	0.002990254
ENSG00000118849	RARRES1	48.78486101	92.424895	-0.922670472	0.000451826	0.003000483
ENSG00000227712	AL359915.1	16.6502961	1.988100376	3.013582517	0.000452306	0.003002857
ENSG00000135678	CPM	57.81938785	23.01121106	1.317846489	0.000454697	0.003017912
ENSG00000131941	RHPN2	2171.510701	2920.164476	-0.427637132	0.000454926	0.003018358
ENSG00000179627	ZBTB42	315.3614496	440.8778522	-0.483503158	0.00045501	0.003018358
ENSG00000129158	SERGEF	179.7622106	272.1342265	-0.597878139	0.000455184	0.003018697
ENSG00000232692	AP001596.1	7.718669601	0	5.529239646	0.000455474	0.003019803

ENSG00000231625	SLC47A1P2	37.08373644	12.9449463	1.516905983	0.000456612	0.003026534
ENSG00000269834	ZNF528-AS1	10.60423298	0.331593289	5.026565714	0.000457781	0.00303346
ENSG00000100417	PMM1	594.1467174	828.3075024	-0.478956888	0.000458762	0.003039145
ENSG00000177700	POLR2L	1923.430635	2517.080987	-0.388343093	0.000459051	0.003040237
ENSG00000079215	SLC1A3	361.409481	583.5972506	-0.689864819	0.000459555	0.003042753
ENSG00000275131	AC241952.1	473.9435763	334.4683957	0.50379753	0.000462139	0.003059036
ENSG00000152503	TRIM36	447.4108046	322.3160736	0.472784079	0.000462818	0.003062707
ENSG00000142676	RPL11	16870.57349	21253.81606	-0.333200322	0.000463126	0.003063918
ENSG00000250073	AP000866.2	28.80869292	64.74886266	-1.172398194	0.000463338	0.003064494
ENSG00000107099	DOCK8	1030.694038	1633.936186	-0.664184561	0.000464536	0.00307159
ENSG00000179431	FJX1	907.6337323	573.7870838	0.660374892	0.000464712	0.003071932
ENSG00000279495	AL928654.4	240.291002	155.2083291	0.628851353	0.000466534	0.003083142
ENSG00000184939	ZFP90	1226.26921	1583.497926	-0.368390747	0.000467757	0.003090392
ENSG00000179930	ZNF648	32.17484198	8.977592742	1.855974496	0.000468672	0.003095604
ENSG00000237223	SULT1C2P1	13.07168257	41.79160755	-1.672314738	0.000469525	0.003100403
ENSG00000148358	GPR107	4460.726603	5527.319497	-0.309223842	0.000470959	0.003109041
ENSG00000164307	ERAP1	592.6642209	877.8354149	-0.565857361	0.000474249	0.003129915
ENSG00000234678	ELF3-AS1	146.2737262	236.4162003	-0.69175295	0.000476437	0.003143509
ENSG00000267091	CTBP2P7	13.29841936	0.600305139	4.465622918	0.000478029	0.00315317
ENSG00000006611	USH1C	0.367374272	12.65020116	-5.012882968	0.00047825	0.003153778
ENSG00000179862	CITED4	128.5552345	209.4697413	-0.703485693	0.000479746	0.003162262
ENSG00000104983	CCDC61	512.7500279	364.6462908	0.490955667	0.000479794	0.003162262
ENSG00000259605	AC074212.1	94.39511254	180.7058314	-0.934202274	0.000480288	0.003164659
ENSG00000198855	FICD	339.1442379	503.1441097	-0.568957411	0.000480416	0.003164659
ENSG00000140396	NCOA2	1889.038382	2399.924125	-0.345037521	0.00048101	0.003167723
ENSG00000131951	LRRC9	51.62999595	21.58948639	1.254058848	0.000482127	0.00317423
ENSG00000113790	EHHADH	836.5016332	606.1576457	0.463998009	0.000482917	0.003178095
ENSG00000269743	SLC25A53	191.1000623	112.679591	0.764367436	0.000482973	0.003178095
ENSG00000154727	GABPA	1637.922427	2087.144046	-0.349768103	0.000484962	0.003190324
ENSG00000143891	GALM	305.7210905	436.7761362	-0.515925241	0.000485302	0.003191706
ENSG00000285852	AL353147.1	15.57036813	2.150203102	2.874498381	0.000491629	0.003232045
ENSG00000182858	ALG12	443.3552639	619.8983156	-0.483059213	0.000491698	0.003232045
ENSG00000135540	NHSL1	461.9150406	659.0536866	-0.513867379	0.000494216	0.003247555
ENSG00000156958	GALK2	603.8190946	834.9079017	-0.468189579	0.000494322	0.003247555
ENSG00000125351	UPF3B	925.7888799	1219.27428	-0.397764196	0.000495012	0.003251214
ENSG00000101825	MXRA5	0	7.928778631	-5.303014477	0.000497572	0.003267154
ENSG00000149809	TM7SF2	4111.278168	5126.655678	-0.318332173	0.000499489	0.003278724
ENSG00000118689	FOXO3	3490.668992	2729.991158	0.354739717	0.000499601	0.003278724
ENSG00000146005	PSD2	90.44785478	45.4534794	0.988715022	0.000500614	0.003284491
ENSG00000147036	LANCL3	0.349140684	11.92413314	-4.929074228	0.000500985	0.003286048
ENSG00000182796	TMEM198B	1450.586922	937.6053992	0.630276702	0.000502206	0.003293181
ENSG00000149313	AASDHPPT	1217.932964	1594.359341	-0.388967041	0.000504221	0.003305505
ENSG00000170175	CHRNB1	892.8837271	1264.383659	-0.501250857	0.000505111	0.003310457

ENSG00000090924	PLEKHG2	1080.568692	1462.952725	-0.436619176	0.000506347	0.003317674
ENSG00000109736	MFSD10	3709.242181	2878.793415	0.365955093	0.000506772	0.003319574
ENSG00000102901	CENPT	799.6999734	1037.935764	-0.375821644	0.000509071	0.003333742
ENSG00000256073	URB1-AS1	226.7231914	136.526059	0.727575487	0.000509279	0.003334214
ENSG00000182158	CREB3L2	3004.364243	3852.353942	-0.35853396	0.000513752	0.003362604
ENSG00000102882	MAPK3	2361.237519	3047.200198	-0.367728235	0.000513958	0.003363052
ENSG00000198455	ZXDB	616.8108763	811.048704	-0.395370457	0.0005146	0.003366361
ENSG00000224786	CETN4P	18.07032364	2.610999037	2.759522213	0.000514899	0.003367414
ENSG00000256417	AC006206.2	48.28081051	18.22148692	1.410246979	0.000517415	0.003382973
ENSG00000109610	SOD3	0	8.014253593	-5.31617656	0.000519717	0.00339712
ENSG00000110900	TSPAN11	60.50326316	24.12187726	1.333379028	0.000521947	0.003410782
ENSG00000054282	SDCCAG8	609.1215336	826.203778	-0.439890354	0.000522468	0.003413283
ENSG00000167658	EEF2	105338.9174	133927.0549	-0.346406486	0.00052346	0.003418852
ENSG00000131435	PDLIM4	15.2524634	2.150203102	2.843966208	0.000524318	0.003423543
ENSG00000135828	RNASEL	413.4642545	294.4109397	0.49135552	0.000525453	0.003430045
ENSG00000237945	LINC00649	849.9898568	589.7804361	0.52839749	0.000525922	0.003432194
ENSG00000100650	SRSF5	6480.566077	8812.95411	-0.443400692	0.000528226	0.003446315
ENSG00000101850	GPR143	269.066233	147.4231246	0.871471526	0.000529027	0.003450288
ENSG00000103540	CCP110	1070.804455	1414.435294	-0.402011118	0.000529116	0.003450288
ENSG00000277196	AC007325.2	52.43244763	110.8456845	-1.077122212	0.000530714	0.003459137
ENSG00000120159	CAAP1	963.3246656	1282.339547	-0.413119641	0.000530755	0.003459137
ENSG00000259803	SLC22A31	67.13281232	29.54875469	1.184396598	0.000531592	0.00346367
ENSG00000092330	TINF2	1310.792124	976.2386572	0.424366263	0.000533353	0.003474223
ENSG00000280347	AC000123.3	195.2082658	122.375434	0.674591243	0.00053417	0.003478625
ENSG00000163083	INHBB	105.430613	180.6330477	-0.778883711	0.00053469	0.003481085
ENSG00000125848	FLRT3	17.34857746	55.11210972	-1.668945753	0.000535737	0.003486975
ENSG00000124151	NCOA3	2593.645416	3266.755487	-0.332976162	0.000537162	0.003495325
ENSG00000186897	C1QL4	16.62825262	50.55957841	-1.602098805	0.000537711	0.00349759
ENSG00000087008	ACOX3	815.2987124	610.8616472	0.416527522	0.000537795	0.00349759
ENSG00000119950	MXI1	1195.005882	1570.428532	-0.393654664	0.000538595	0.0035018
ENSG00000108924	HLF	142.1805997	80.94633937	0.81606934	0.000538727	0.0035018
ENSG00000171503	ETFDH	390.2078741	533.3253407	-0.450245256	0.000539331	0.003504797
ENSG00000198369	SPRED2	3536.727399	2748.427715	0.363596214	0.000539657	0.003505985
ENSG00000262877	AC110285.2	439.5711412	786.2613217	-0.837916406	0.000541079	0.003514293
ENSG00000117899	MESD	1280.141026	1697.146391	-0.406721041	0.000544499	0.003535574
ENSG00000169598	DFFB	396.3955307	561.7461263	-0.503852364	0.000548577	0.00356111
ENSG00000230438	SERPINB9P1	235.4467913	149.7901423	0.65497629	0.000548934	0.003562487
ENSG00000243477	NAA80	553.5858352	400.7997703	0.466585788	0.000550727	0.003573178
ENSG00000021300	PLEKHB1	795.8977797	527.3174033	0.595456938	0.000551312	0.003576024
ENSG00000135686	KLHL36	3312.549261	2570.951769	0.365872294	0.000554007	0.003592561
ENSG00000112715	VEGFA	4575.984621	6705.597922	-0.551190096	0.000554618	0.003595574
ENSG00000139985	ADAM21	128.647521	70.50922275	0.869789148	0.000557343	0.003612283
ENSG00000152926	ZNF117	964.5560328	670.2607816	0.526279173	0.000563171	0.003649092

ENSG00000169507	SLC38A11	0	8.054541512	-5.322390087	0.000565387	0.003662483
ENSG00000048707	VPS13D	2787.981747	2182.467793	0.353048864	0.000565536	0.003662486
ENSG00000157657	ZNF618	2198.11593	2734.69124	-0.31520928	0.000569859	0.003689508
ENSG00000012223	LTF	17.18177266	2.504389913	2.80805705	0.000572031	0.003701824
ENSG00000103742	IGDCC4	4.373294051	21.46423182	-2.297163805	0.000572063	0.003701824
ENSG00000232573	RPL3P4	76.13595183	132.4269358	-0.7989841	0.000575724	0.003724532
ENSG00000102178	UBL4A	1457.548353	1056.212016	0.464227837	0.000576244	0.003726917
ENSG00000235721	AC013268.3	6.530610066	26.07363779	-1.994837322	0.000577832	0.003736203
ENSG00000217648	AL136116.3	33.11809373	73.40559868	-1.145644999	0.000578489	0.003739465
ENSG00000149930	TAOK2	3619.793182	2900.084182	0.319614838	0.000580507	0.003751518
ENSG00000180992	MRPL14	1378.295804	958.7573233	0.522793219	0.000581283	0.00375555
ENSG00000140563	MCTP2	489.4773371	682.514604	-0.479426956	0.000583537	0.003769116
ENSG00000197779	ZNF81	327.8209613	216.2513701	0.601644538	0.000585615	0.003781545
ENSG00000166035	LIPC	2.180780823	17.04098145	-2.960008079	0.000586694	0.003787519
ENSG00000274605	AL355338.1	109.9952953	175.7734187	-0.676680117	0.000587489	0.003791653
ENSG00000135390	ATP5MC2	3190.53704	4027.43874	-0.335972334	0.000588668	0.003798265
ENSG00000068912	ERLEC1	1556.991406	2130.679103	-0.45249965	0.000589135	0.003800274
ENSG00000133313	CNDP2	4254.807103	3278.937751	0.375566024	0.000589992	0.003804802
ENSG00000121039	RDH10	289.0187849	401.6644092	-0.475070449	0.000590825	0.003809174
ENSG00000101849	TBL1X	1523.560151	1971.652823	-0.372358236	0.000591046	0.003809602
ENSG00000117407	ARTN	330.5893408	211.0144922	0.644704771	0.000594122	0.003828422
ENSG00000183723	CMTM4	7173.489089	8889.173447	-0.309360407	0.000594306	0.003828604
ENSG00000178913	TAF7	5114.055055	4158.3101	0.298527778	0.000594679	0.003830001
ENSG00000267249	AP005482.3	27.74684717	64.46288643	-1.216807825	0.000595071	0.003831521
ENSG00000249131	PSD2-AS1	10.43886072	0.277559048	5.008005223	0.00059656	0.003840102
ENSG00000115165	CYTIP	43.20131258	13.19398334	1.731290935	0.000597788	0.003846999
ENSG00000149927	DOC2A	664.6606044	982.2957546	-0.562756565	0.000598839	0.003852751
ENSG00000103495	MAZ	1590.163937	2168.677919	-0.447578787	0.000599852	0.003858258
ENSG00000182568	SATB1	540.0749402	821.7608404	-0.604457152	0.000601648	0.003868795
ENSG00000196535	MYO18A	7461.466112	5539.845201	0.42947038	0.000602325	0.003872137
ENSG00000089009	RPL6	28201.97974	34757.92969	-0.301560163	0.000605375	0.003890721
ENSG00000101199	ARFGAP1	4558.512849	3548.284176	0.361477827	0.000605809	0.003892492
ENSG00000100138	SNU13	2828.598431	3738.311039	-0.402450516	0.000606176	0.003893834
ENSG00000197608	ZNF841	282.536223	179.6448214	0.655399627	0.000612475	0.003933264
ENSG00000159915	ZNF233	73.12132079	31.03028548	1.239574444	0.000614203	0.00394333
ENSG00000255302	EID1	5542.120592	4348.704023	0.349670386	0.000623906	0.004004579
ENSG00000239305	RNF103	844.8616574	1093.04076	-0.371295288	0.00062598	0.00401684
ENSG00000198925	ATG9A	1644.542604	1270.41028	0.372013568	0.000627306	0.004023488
ENSG00000128335	APOL2	930.267914	716.2422378	0.377300285	0.000627344	0.004023488
ENSG00000172379	ARNT2	1625.617483	1258.897208	0.368472914	0.000629196	0.004034318
ENSG00000072210	ALDH3A2	5618.301239	4528.16821	0.311182572	0.000629874	0.004037611
ENSG00000184110	EIF3C	771.0709139	1036.504462	-0.427388876	0.00063175	0.004048579
ENSG00000225127	LINC00237	39.61746784	14.78614963	1.421959346	0.000632178	0.00405026

ENSG00000231948	HS1BP3-IT1	21.52637068	4.408474688	2.318420648	0.000632912	0.004053908
ENSG00000111328	CDK2AP1	1450.813885	964.2035878	0.588844179	0.000634021	0.004059951
ENSG00000166888	STAT6	4006.947375	5441.246495	-0.441257194	0.000635498	0.004068346
ENSG00000117298	ECE1	4376.446105	3376.546898	0.374033442	0.000636597	0.004074322
ENSG00000103091	WDR59	1722.506582	2199.656191	-0.352507712	0.000637325	0.004077914
ENSG00000257923	CUX1	5659.828137	7067.371316	-0.320315309	0.000638794	0.004085905
ENSG00000177842	ZNF620	319.6905165	455.194255	-0.508215782	0.000638906	0.004085905
ENSG00000162517	PEF1	1945.004891	1528.431407	0.347416012	0.000641597	0.004101222
ENSG00000170540	ARL6IP1	6378.70721	8670.007379	-0.442824491	0.000641636	0.004101222
ENSG00000264456	AC138207.4	25.44889367	60.8629577	-1.256288774	0.000641861	0.004101598
ENSG00000163499	CRYBA2	1.849873727	16.73198168	-3.188625635	0.00064416	0.004113417
ENSG00000144736	SHQ1	757.4566615	520.3499573	0.540470654	0.000644195	0.004113417
ENSG00000183386	FHL3	733.4205037	553.9934702	0.40436066	0.000644214	0.004113417
ENSG00000172020	GAP43	6.940952698	0	5.377279642	0.000644954	0.004117074
ENSG00000164520	RAET1E	19.60879382	48.51736549	-1.303192635	0.000649601	0.004145657
ENSG00000221983	UBA52	9185.191323	11427.74363	-0.315194985	0.000649967	0.004146917
ENSG00000110917	MLEC	8153.140935	10249.75257	-0.330120916	0.000650725	0.004150677
ENSG00000198130	HIBCH	620.7245208	842.0902742	-0.440577595	0.000652098	0.004158351
ENSG00000157734	SNX22	149.9503228	230.6877893	-0.620015584	0.000654672	0.004172677
ENSG00000135677	GNS	6338.40551	5155.11042	0.298166328	0.000654684	0.004172677
ENSG00000219438	FAM19A5	12.42287075	0.600305139	4.363185675	0.000655975	0.004179817
ENSG00000251022	THAP9-AS1	848.403501	1164.146784	-0.45689206	0.000656858	0.0041835
ENSG00000047410	TPR	5863.787917	7520.695864	-0.3590572	0.000656894	0.0041835
ENSG00000174165	ZDHHC24	1369.207067	1078.094513	0.344766586	0.000657576	0.004186149
ENSG00000170004	CHD3	6517.831653	8218.031742	-0.334279279	0.000657728	0.004186149
ENSG00000143401	ANP32E	3679.379238	5614.983613	-0.609988477	0.000657821	0.004186149
ENSG00000187554	TLR5	157.669155	94.49159081	0.742348679	0.00065871	0.004190719
ENSG00000161270	NPHS1	42.06541388	103.1634014	-1.292283305	0.000659167	0.004192539
ENSG00000100124	ANKRD54	696.086406	938.9157207	-0.432285912	0.000659684	0.004194741
ENSG00000161849	KRT84	7.686163682	0	5.524358296	0.00065996	0.004195412
ENSG00000167395	ZNF646	1847.572126	1473.557181	0.326458649	0.0006617	0.004205384
ENSG00000254290	AC124067.4	6.54234247	29.24998318	-2.154800085	0.000663168	0.00421362
ENSG00000101084	RAB5IF	1025.294561	717.0107944	0.514783177	0.000667967	0.004243018
ENSG00000108559	NUP88	1783.302718	2622.834476	-0.556953306	0.000669207	0.004249791
ENSG00000161509	GRIN2C	144.2963687	78.63095822	0.880428209	0.000670951	0.004259769
ENSG00000054179	ENTPD2	187.1058862	286.1401701	-0.610552926	0.000671512	0.004261492
ENSG00000139725	RHOF	864.2405661	645.4785833	0.420645175	0.00067157	0.004261492
ENSG00000103489	XYLT1	45.50608122	108.8157585	-1.252669662	0.000672106	0.004263792
ENSG00000115112	TFCP2L1	734.7732963	456.9676262	0.683664939	0.00067666	0.004290742
ENSG00000123201	GUCY1B2	333.2243886	192.01349	0.798942675	0.000676704	0.004290742
ENSG00000265194	AL359922.2	110.5082243	58.83567706	0.904233939	0.000679856	0.004309615
ENSG00000137225	CAPN11	26.8064493	7.538251975	1.831574251	0.000681017	0.004315859
ENSG00000279528	AC115618.3	94.38575747	162.7738388	-0.786033863	0.000681236	0.004316138

ENSG00000136628	EPRS	10426.50775	13593.27581	-0.38269378	0.000682211	0.004321201
ENSG00000117620	SLC35A3	1176.237142	1526.537316	-0.376490502	0.00068403	0.004331604
ENSG00000075945	KIFAP3	589.40414	440.5916026	0.419290583	0.000686103	0.00434361
ENSG00000102710	SUPT20H	1581.313467	2109.936487	-0.416546514	0.00068769	0.004352534
ENSG00000172375	C2CD2L	596.4839724	819.8470337	-0.458567781	0.000690416	0.004368663
ENSG00000175305	CCNE2	319.222294	447.3649453	-0.487473027	0.000691412	0.004373837
ENSG00000143162	CREG1	2970.952693	3693.799778	-0.314087645	0.000692273	0.004378156
ENSG00000189144	ZNF573	197.0040666	123.9857246	0.671180921	0.00069736	0.004409188
ENSG00000181626	ANKRD62	20.2354108	3.399948497	2.59341807	0.000703665	0.004447908
ENSG00000163825	RTP3	6.878480787	0	5.36658189	0.000705163	0.00445623
ENSG00000100554	ATP6V1D	1588.587875	1240.936499	0.356393284	0.000705859	0.004459479
ENSG00000251141	MRPS30-DT	40.48508272	83.92872404	-1.056388865	0.000707018	0.004465656
ENSG00000109381	ELF2	907.3552804	1180.159993	-0.378812451	0.000707667	0.004468079
ENSG00000179088	C12orf42	48.6037951	18.60711445	1.392034951	0.000707766	0.004468079
ENSG00000127980	PEX1	1060.105721	1402.407731	-0.404279985	0.000708053	0.00446874
ENSG00000285212	AL009178.3	1.047422052	14.23987873	-3.721205602	0.000708262	0.004468914
ENSG00000122735	DNAI1	45.93307352	15.89045734	1.546702471	0.000710821	0.004483907
ENSG00000071537	SEL1L	2406.973794	3353.58544	-0.478387373	0.000713426	0.004499184
ENSG00000244300	GATA2-AS1	747.0060571	1078.112378	-0.528376165	0.000713962	0.004501405
ENSG00000166710	B2M	2173.871569	2702.481699	-0.314094579	0.00071573	0.004511395
ENSG00000158246	TENT5B	480.986322	278.1727848	0.78773587	0.000716479	0.004514956
ENSG00000109189	USP46	933.4303258	1197.318906	-0.359522067	0.000719687	0.004534011
ENSG00000114796	KLHL24	1042.996846	1481.706956	-0.506015421	0.000720201	0.004536083
ENSG00000108107	RPL28	11392.05239	14365.19119	-0.334561595	0.000729103	0.004590975
ENSG00000109065	NAT9	1511.560331	1138.098134	0.40920169	0.000731108	0.004602418
ENSG00000118058	KMT2A	4808.402097	5926.563012	-0.301652558	0.000731791	0.004605535
ENSG00000078403	MLLT10	2316.0771	2876.092746	-0.312371746	0.000734216	0.004619616
ENSG00000140848	CPNE2	1153.603891	906.433873	0.347919188	0.00073633	0.00463173
ENSG00000226210	WASH8P	2262.716788	1742.194165	0.377533408	0.000739488	0.004650405
ENSG00000130477	UNC13A	45.78989611	18.91216615	1.282565223	0.000740884	0.004657991
ENSG00000105993	DNAJB6	4540.797592	3641.046882	0.318444836	0.000743597	0.004673845
ENSG00000131747	TOP2A	13112.07325	19076.9966	-0.540968358	0.000746161	0.004688763
ENSG00000177570	SAMD12	1067.16035	831.3985823	0.36030605	0.000749304	0.004707311
ENSG00000165521	EML5	104.0885997	166.8513979	-0.683141513	0.000753296	0.004731179
ENSG00000075292	ZNF638	4221.881207	5230.643824	-0.30898746	0.000753896	0.004733737
ENSG00000163788	SNRK	767.2159314	551.8420426	0.47431688	0.000754547	0.004736611
ENSG00000141367	CLTC	23297.8435	18024.02169	0.370222753	0.000756408	0.00474708
ENSG00000268655	AC008687.4	60.6607129	124.9622108	-1.039337268	0.00076029	0.00477022
ENSG00000002016	RAD52	444.1142595	606.0601572	-0.448901804	0.000762389	0.004782172
ENSG00000149357	LAMTOR1	1836.598946	1436.562215	0.35406877	0.000763493	0.004787029
ENSG00000258976	AC013451.2	9.905951608	0.331593289	4.927512174	0.000763567	0.004787029
ENSG00000105088	OLFM2	97.3379166	176.5175857	-0.855632455	0.000763749	0.004787029
ENSG00000260196	AC124798.1	214.641365	342.039522	-0.674454614	0.000764386	0.004789802

ENSG00000188389	PDCD1	100.639379	50.91815603	0.982060652	0.000765116	0.004793151
ENSG00000105939	ZC3HAV1	2205.511338	2825.022687	-0.357127471	0.000775381	0.004856217
ENSG00000161791	FMNL3	1844.469503	1387.431998	0.411354335	0.000775614	0.004856438
ENSG00000177427	MIEF2	917.4139159	705.1890238	0.379544496	0.00077758	0.00486751
ENSG00000105963	ADAP1	1400.718068	1064.735091	0.395170969	0.000777866	0.004868054
ENSG00000241661	PPP1R2P6	38.66517498	12.44042268	1.65185869	0.000778466	0.00487057
ENSG00000283526	PRRT1B	2.199014411	16.26577832	-2.891052494	0.00078022	0.004880303
ENSG00000132840	BHMT2	1.083889228	15.79218711	-3.85520798	0.000784693	0.004907031
ENSG00000131584	ACAP3	3068.854665	2006.094627	0.613738948	0.000785671	0.004911897
ENSG00000168802	CHTF8	3405.106779	4335.935562	-0.34869239	0.00078623	0.00491414
ENSG00000105186	ANKRD27	2244.454415	2985.985829	-0.412085113	0.000787271	0.004919394
ENSG00000105516	DBP	1048.105384	1459.01749	-0.476753549	0.000794131	0.004960996
ENSG00000268521	VN1R83P	58.68954266	26.44501069	1.146956581	0.000795278	0.004966898
ENSG00000197056	ZMYM1	752.0878064	1021.875426	-0.442786002	0.000796323	0.004972155
ENSG00000225030	AL355483.1	21.05355614	4.693421574	2.144790218	0.000796942	0.004974756
ENSG00000166908	PIP4K2C	1701.055958	1285.776369	0.403142259	0.000799457	0.004989187
ENSG00000116670	MAD2L2	1798.357603	1249.200519	0.525015136	0.000799958	0.004991046
ENSG00000234350	AC007405.1	93.39986254	50.80415907	0.881036677	0.00080164	0.004999789
ENSG00000155918	RAET1L	29.95267672	68.6204347	-1.196329391	0.000801767	0.004999789
ENSG00000074211	PPP2R2C	12.0072969	0.877864186	3.74018202	0.000802981	0.005006088
ENSG00000109790	KLHL5	1789.84281	2246.24903	-0.327668482	0.00080519	0.005018588
ENSG00000104549	SQLE	4602.465179	3636.216456	0.339859547	0.000805761	0.005019761
ENSG00000111802	TDP2	1148.874947	1470.606055	-0.356495203	0.000805787	0.005019761
ENSG00000158427	TMSB15B	43.49194261	17.84565764	1.287819359	0.000806304	0.005021265
ENSG00000227671	AL390728.4	1011.148196	772.2801081	0.388291337	0.000806438	0.005021265
ENSG00000256943	AC148477.3	25.19488218	61.06190858	-1.275793098	0.000806928	0.005023047
ENSG00000123130	ACOT9	1621.371067	1225.439864	0.403199479	0.000807351	0.005024407
ENSG00000117971	CHRNB4	9.751041795	33.18099689	-1.758287298	0.000809706	0.005037782
ENSG00000205730	ITPRIPL2	2036.831053	2631.833617	-0.369625084	0.000811297	0.005046405
ENSG00000260029	AC007608.2	54.25663058	22.342096	1.282125757	0.000812168	0.005050237
ENSG00000225920	RIMKLBP2	96.31649928	51.80683509	0.896800339	0.000812325	0.005050237
ENSG00000283230	AC099654.5	6.910986706	0	5.372152756	0.000814129	0.005060171
ENSG00000145692	BHMT	5.789360339	24.81745561	-2.093169287	0.00081521	0.005065608
ENSG00000204860	FAM201A	529.1407673	731.5305137	-0.46607263	0.000819372	0.005090184
ENSG00000196428	TSC22D2	1419.877487	1057.645818	0.424221091	0.000829185	0.005149841
ENSG00000279488	AC004623.1	43.32133914	16.90001289	1.360705295	0.000829844	0.005152628
ENSG00000138769	CDKL2	121.3371307	191.3904217	-0.657679421	0.000830337	0.005153293
ENSG00000256967	AC018653.3	276.9688336	181.3946996	0.613213708	0.000830446	0.005153293
ENSG00000250326	AC104596.1	5.836289955	26.00190915	-2.157831369	0.00083058	0.005153293
ENSG00000137575	SDCBP	3388.808831	2559.758148	0.404526453	0.000834883	0.005178682
ENSG00000224309	ANKRD30BP2	9.451370654	0.331593289	4.862149297	0.000835954	0.005184012
ENSG00000146007	ZMAT2	1206.956277	941.0790323	0.358539452	0.000837861	0.005194524
ENSG00000165526	RPUSD4	1351.750672	1759.207575	-0.380397717	0.000838944	0.00519993

ENSG00000126602	TRAP1	3310.3351	4668.228621	-0.496060006	0.000843253	0.005225315
ENSG00000065518	NDUFB4	2989.236314	2224.68572	0.425863576	0.000843907	0.005228049
ENSG00000275552	AC243965.2	56.89184099	21.23873934	1.41202935	0.000845211	0.005234809
ENSG00000127325	BEST3	32.74690956	10.40026846	1.644697164	0.000846533	0.005241674
ENSG00000166822	TMEM170A	1212.594027	1573.40104	-0.375626495	0.000847829	0.005248371
ENSG00000188613	NANOS1	636.2411554	475.1862714	0.421330189	0.000850984	0.005266079
ENSG00000118507	AKAP7	148.1288423	238.5196402	-0.688557785	0.000851311	0.005266079
ENSG00000272455	AL391244.3	221.5981852	318.8197315	-0.523612844	0.000851333	0.005266079
ENSG00000075702	WDR62	1443.046851	920.489421	0.647724136	0.000852314	0.005270819
ENSG00000143727	ACP1	4471.736455	3604.965898	0.310689899	0.000855033	0.005286304
ENSG00000177479	ARIH2	2992.967269	3700.704498	-0.306326188	0.000856919	0.00529663
ENSG00000136783	NIPSNAP3A	712.985328	927.4727232	-0.379759718	0.000858128	0.005302771
ENSG00000165806	CASP7	694.3915988	961.9804192	-0.470315211	0.000859511	0.005309978
ENSG00000108641	B9D1	1177.091598	877.7028629	0.422664588	0.000859767	0.005310227
ENSG00000152102	FAM168B	6741.612637	8245.187169	-0.2905067	0.000860578	0.005313333
ENSG00000124120	TTPAL	1486.759018	1154.699572	0.364545255	0.000860703	0.005313333
ENSG00000114573	ATP6V1A	4128.854149	3199.160569	0.367778493	0.00086145	0.005316603
ENSG00000006451	RALA	1414.670191	1824.57376	-0.367291832	0.000862768	0.005323404
ENSG00000017797	RALBP1	2559.754094	3481.081028	-0.443765815	0.000863333	0.005324938
ENSG00000163464	CXCR1	9.434407029	0.300152569	4.860023041	0.000863451	0.005324938
ENSG00000147536	GINS4	1122.850992	659.8808265	0.765678141	0.000864545	0.005330348
ENSG00000127616	SMARCA4	11141.87747	14516.97283	-0.381805678	0.000866432	0.005340638
ENSG00000171729	TMEM51	1102.948837	1479.994133	-0.424538548	0.000868452	0.005351549
ENSG00000203791	EEF1AKMT2	214.0693199	324.858432	-0.603393653	0.000868638	0.005351549
ENSG00000177465	ACOT4	64.78000669	120.0955072	-0.885857543	0.000869197	0.00535365
ENSG00000072518	MARK2	2097.61039	2779.132401	-0.406107907	0.000869802	0.00535497
ENSG00000169410	PTPN9	1935.365126	2402.220495	-0.311765927	0.000869847	0.00535497
ENSG00000259321	AL136295.2	11.42095698	40.2839779	-1.825659096	0.000870251	0.005356112
ENSG00000266076	AC004805.1	41.18367804	12.51018365	1.713769939	0.000874463	0.00538069
ENSG00000186417	GLDN	54.85042768	111.7421941	-1.022800255	0.000876561	0.005389946
ENSG00000169116	PARM1	1189.445938	431.3187169	1.464003132	0.000876566	0.005389946
ENSG00000172006	ZNF554	407.1376986	290.4666224	0.487868133	0.000876626	0.005389946
ENSG00000105063	PPP6R1	4352.765581	3358.864112	0.373710666	0.00087794	0.005396671
ENSG00000104213	PDGFRL	146.1516362	218.9194787	-0.58188873	0.000879062	0.005402217
ENSG00000162976	PQLC3	1027.986555	788.3716406	0.382979593	0.000890225	0.005469446
ENSG00000142453	CARM1	4257.731706	5381.863638	-0.338093999	0.000891984	0.005478886
ENSG00000203709	MIR29B2CHG	117.3662455	209.8409449	-0.83570358	0.000892453	0.005480393
ENSG00000070087	PFN2	4116.775471	3308.457098	0.315534349	0.000893545	0.005485724
ENSG00000112796	ENPP5	682.7142447	511.4106068	0.417739745	0.000893967	0.005486948
ENSG00000163702	IL17RC	932.9077248	1204.802068	-0.368591696	0.000896913	0.005503655
ENSG00000015532	XYLT2	2135.629094	1642.600164	0.378432816	0.000898982	0.005514972
ENSG00000278500	AC009336.2	14.47474649	38.55384008	-1.408590551	0.000899295	0.005515511
ENSG00000160410	SHKBP1	3106.017164	2240.9534	0.470558657	0.000901015	0.005524682

ENSG00000196421	C20orf204	70.40287617	31.61589324	1.160148715	0.000902155	0.00553029
ENSG00000080822	CLDND1	2068.455402	1564.331094	0.402515835	0.000902397	0.00553039
ENSG00000111737	RAB35	3216.847597	2467.823209	0.382096022	0.000908454	0.005566124
ENSG00000126970	ZC4H2	470.8553801	342.1169315	0.460454104	0.000909376	0.005570379
ENSG00000158042	MRPL17	1136.895564	1737.703958	-0.612599326	0.000909764	0.005571131
ENSG00000165449	SLC16A9	447.3124964	603.3253884	-0.432577732	0.000909952	0.005571131
ENSG00000164663	USP49	697.9757633	524.365599	0.411804888	0.00091131	0.005578054
ENSG00000139372	TDG	1346.445744	1869.284471	-0.473653441	0.000912141	0.00558175
ENSG00000167130	DOLPP1	708.5969712	944.4127506	-0.415257268	0.000920658	0.005632461
ENSG00000204791	SMPD5	51.70039038	106.9613299	-1.046840353	0.000921686	0.00563616
ENSG00000155792	DEPTOR	66.30562587	121.6942013	-0.876992768	0.000921889	0.00563616
ENSG00000167107	ACSF2	1003.254933	741.5344579	0.436310071	0.000921951	0.00563616
ENSG00000247157	LINC01252	32.95795269	7.012085887	2.26013655	0.000922879	0.005640428
ENSG00000241832	CECR3	0.367374272	11.16413569	-4.831829186	0.000924495	0.0056489
ENSG00000102531	FNDC3A	3443.352717	4303.999006	-0.321721122	0.000925315	0.0056525
ENSG00000138594	TMOD3	4209.354637	3257.536729	0.369581981	0.000927938	0.005667119
ENSG00000185480	PARPBP	326.3226056	492.8297348	-0.595991037	0.000929139	0.00567269
ENSG00000081041	CXCL2	14.46174413	1.710541329	3.029966254	0.000929313	0.00567269
ENSG00000120370	GORAB	471.5880682	340.8264427	0.468789296	0.000930006	0.005675512
ENSG00000205476	CCDC85C	1426.607876	1810.542176	-0.344084194	0.000931326	0.005682034
ENSG00000143951	WDPCP	341.3870141	238.6844268	0.516738426	0.000931538	0.005682034
ENSG00000176428	VPS37D	187.9487663	285.8281861	-0.603702771	0.000932396	0.005685853
ENSG00000170234	PWWP2A	854.1043847	1097.604769	-0.361667844	0.000932822	0.005687041
ENSG00000141837	CACNA1A	128.3416627	62.04140446	1.054873731	0.000934051	0.005693118
ENSG00000258512	LINC00239	106.8309969	56.45104324	0.92566184	0.000934367	0.005693633
ENSG00000116337	AMPD2	1869.68912	1416.672338	0.39988984	0.00093575	0.005700646
ENSG00000165807	PPP1R36	50.45763008	19.23147247	1.405630288	0.000937555	0.005710225
ENSG00000127947	PTPN12	1557.926617	2029.365223	-0.381519129	0.000938888	0.005716925
ENSG00000180871	CXCR2	11.33898152	0.577711617	4.242707992	0.000939708	0.005720497
ENSG00000163932	PRKCD	609.9939339	817.7053516	-0.422526616	0.000940939	0.005726575
ENSG00000169375	SIN3A	3198.651311	4084.561914	-0.352796388	0.000941633	0.005729378
ENSG00000137494	ANKRD42	499.4777676	361.2486744	0.466288362	0.000943583	0.00573982
ENSG00000169100	SLC25A6	19787.48458	25128.98928	-0.344772086	0.000944567	0.005744381
ENSG00000138768	USO1	4069.501528	5153.013648	-0.340632877	0.000944988	0.005745516
ENSG00000127948	POR	6758.265795	8508.79527	-0.332202297	0.000946509	0.005753343
ENSG00000213516	RBMXL1	644.4451624	862.6939702	-0.420964972	0.000948971	0.005766347
ENSG00000230202	AL450405.1	192.4792074	290.0436256	-0.590538649	0.000949119	0.005766347
ENSG00000109756	RAPGEF2	2487.163596	3077.995621	-0.307366848	0.000949403	0.005766646
ENSG00000167371	PRRT2	74.87368798	152.6082617	-1.023253693	0.000950975	0.005773427
ENSG00000278709	NKILA	6.555344838	0	5.295608368	0.000950989	0.005773427
ENSG00000141452	RMC1	764.991902	540.4155007	0.500022409	0.000951413	0.005774573
ENSG00000147202	DIAPH2	871.9925463	1138.618816	-0.385091795	0.000953788	0.005787553
ENSG00000184986	TMEM121	45.09684596	84.7624178	-0.91212189	0.000955325	0.00579545

ENSG00000250509	AC034213.1	1.102122816	14.85882932	-3.763735135	0.000956382	0.005800426
ENSG00000279878	AP003108.5	72.85177823	33.98919114	1.091304757	0.000957902	0.005808211
ENSG00000039123	MTREX	2480.456498	3292.385528	-0.408780334	0.000959655	0.005817408
ENSG00000183530	PRR14L	3126.029648	4051.904332	-0.374422947	0.000960084	0.005818571
ENSG00000210176	MT-TH	34.74012329	70.99356326	-1.027196612	0.000960504	0.005819678
ENSG00000099821	POLRMT	3031.520668	2316.439439	0.38787923	0.000961644	0.005824825
ENSG00000188878	FBF1	247.4341193	351.1789826	-0.505712536	0.000961828	0.005824825
ENSG00000181035	SLC25A42	365.2679369	502.6890344	-0.461525712	0.000962798	0.005829201
ENSG00000151470	C4orf33	320.4958221	439.8678539	-0.455741411	0.000963025	0.005829201
ENSG00000167900	TK1	5273.014539	3663.88445	0.52500308	0.000963507	0.005830677
ENSG00000134248	LAMTOR5	1905.734324	1463.54994	0.380509872	0.00096544	0.005840903
ENSG00000154914	USP43	856.3111591	1142.199006	-0.416067762	0.000965672	0.005840903
ENSG00000121988	ZRANB3	363.0985746	503.8653057	-0.474144667	0.00096638	0.00584374
ENSG00000230074	AL162231.2	6.886251934	26.76826509	-1.954887918	0.000966807	0.005844887
ENSG00000139880	CDH24	2597.291144	2063.497544	0.332072998	0.000967295	0.005846395
ENSG00000236991	EDRF1-AS1	35.41239992	73.82019096	-1.056299597	0.000967803	0.005848029
ENSG00000250251	PKD1P6	812.2140121	1167.97366	-0.523768046	0.000969545	0.005857109
ENSG00000204498	NFKBIL1	631.5910017	830.1547781	-0.394313961	0.000970554	0.005861766
ENSG00000073756	PTGS2	0.349140684	10.2396251	-4.71313901	0.000972678	0.005873146
ENSG00000111816	FRK	317.078143	437.8163511	-0.46496512	0.000973985	0.005879593
ENSG00000172878	METAP1D	324.7173771	458.7631329	-0.498549	0.000975537	0.005887512
ENSG00000112081	SRSF3	9682.803196	12610.55979	-0.381208148	0.000977765	0.005899508
ENSG00000273706	LHX1	84.11259418	43.17166324	0.956974197	0.000980739	0.005916002
ENSG00000104901	DKKL1	59.197717	27.76502536	1.086860599	0.000981602	0.005919752
ENSG00000235949	AC061961.1	16.7310016	2.526983435	2.763527602	0.000982888	0.005926053
ENSG00000232650	LINC01780	15.93251118	2.297100144	2.754017441	0.000983147	0.005926157
ENSG00000135486	HNRNPA1	39384.09395	47950.95637	-0.283955787	0.000986002	0.00594191
ENSG00000161533	ACOX1	1857.167498	2326.191075	-0.324696452	0.000990028	0.005963708
ENSG00000135272	MDFIC	690.882138	939.6786512	-0.444179387	0.000990173	0.005963708
ENSG00000211584	SLC48A1	1572.789652	1089.001339	0.53123231	0.000990348	0.005963708
ENSG00000275832	ARHGAP23	1322.890275	1669.199773	-0.335674352	0.000990688	0.005964294
ENSG00000109063	MYH3	186.9424147	117.1617748	0.674533078	0.000997116	0.006001518
ENSG00000110442	COMMD9	638.6881454	831.4699465	-0.380053661	0.000998837	0.006010403
ENSG00000095066	HOOK2	3131.167808	4104.659126	-0.390370307	0.001000835	0.006020954
ENSG00000224184	MIR3681HG	6.58007961	0	5.300125176	0.00100137	0.006022695
ENSG00000168491	CCDC110	138.4131491	82.17941972	0.753034722	0.001002201	0.006026214
ENSG00000236202	GRM7-AS1	9.115232337	0.277559048	4.809032485	0.001002592	0.00602709
ENSG00000182150	ERCC6L2	1141.872021	864.9879183	0.40011446	0.00101006	0.006069711
ENSG00000102359	SRPX2	24.70748135	4.869270623	2.345407299	0.001010176	0.006069711
ENSG00000283755	CPHXL	0	7.028320923	-5.128501163	0.001014565	0.006094589
ENSG00000197245	FAM110D	2.910298147	16.99923417	-2.545931709	0.001015259	0.006097265
ENSG00000256443	AP003559.1	159.3820273	97.80607707	0.701886906	0.001016881	0.006105512
ENSG00000117569	PTBP2	668.2564872	879.1691498	-0.396387099	0.0010187	0.006114191

ENSG00000114993	RTKN	3913.660635	3084.024302	0.343467493	0.001018824	0.006114191
ENSG00000145332	KLHL8	1436.616745	993.2764847	0.531437587	0.001020051	0.006120056
ENSG00000230989	HSBP1	3407.037	2470.861777	0.463144959	0.001022221	0.006131578
ENSG00000091622	PITPNM3	778.9676412	1033.392497	-0.407773872	0.001025731	0.006151127
ENSG00000263001	GTF2I	455.1245487	637.3706855	-0.486007976	0.001026052	0.006151549
ENSG00000260949	AP006545.1	3.91998306	22.38625369	-2.493625182	0.001026704	0.006153958
ENSG00000117906	RCN2	3261.596878	4197.691158	-0.364247455	0.001029273	0.006167849
ENSG00000101049	SGK2	45.07211119	88.92425312	-0.978045397	0.001034657	0.006198599
ENSG00000068366	ACSL4	3784.875473	2734.649417	0.468585249	0.001035197	0.00620032
ENSG00000268362	AC092279.1	287.9436228	198.0113816	0.541319433	0.001036027	0.006203779
ENSG00000119048	UBE2B	1308.339286	1674.688554	-0.35664207	0.001037276	0.006209744
ENSG00000129993	CBFA2T3	0	7.454236374	-5.210340006	0.001038991	0.006218493
ENSG00000276831	AC020922.4	0.747750911	13.32662575	-4.187946978	0.001041381	0.006231278
ENSG00000134627	PIWIL4	57.35957568	104.8780346	-0.867348134	0.001043795	0.006244202
ENSG00000113312	TTC1	1631.28685	1302.801637	0.324360389	0.001045551	0.006253187
ENSG00000048342	CC2D2A	530.4442643	365.4544197	0.539407707	0.001047217	0.00626162
ENSG00000169955	ZNF747	114.8181017	185.7133212	-0.692008069	0.001049073	0.006271192
ENSG00000087087	SRRT	6045.434904	7577.019365	-0.325886401	0.001051774	0.006285807
ENSG00000248746	ACTN3	0	7.059761644	-5.133955506	0.00105433	0.00629955
ENSG00000188859	FAM78B	74.128477	34.22440356	1.121636414	0.001057527	0.006317118
ENSG00000230650	AC140479.2	40.44069303	81.22931859	-1.008414695	0.001062838	0.006347296
ENSG00000187634	SAMD11	45.24683851	206.6377193	-2.190085815	0.001063244	0.006348175
ENSG00000117748	RPA2	1606.964337	1171.959219	0.454633471	0.001074604	0.006414443
ENSG00000156042	CFAP70	914.6117747	513.9435703	0.832712615	0.001076126	0.006421965
ENSG00000077458	FAM76B	786.0178582	1010.952809	-0.362791871	0.001077235	0.006427021
ENSG00000153898	MCOLN2	880.1870274	677.937812	0.376298205	0.001080545	0.006445201
ENSG00000101190	TCFL5	1052.54528	1339.961387	-0.348309764	0.001080919	0.006445869
ENSG00000135698	MPHOSPH6	859.1093644	590.6737021	0.539042945	0.001085079	0.006469108
ENSG00000163449	TMEM169	60.92487288	27.79646608	1.126274586	0.001085834	0.006472032
ENSG00000196388	INCA1	135.5653117	234.2673783	-0.788480605	0.001094923	0.006524627
ENSG00000110921	MVK	1396.218795	1060.205924	0.397026676	0.00109859	0.006544891
ENSG00000107863	ARHGAP21	2808.887301	3546.290463	-0.336460697	0.001099211	0.006546975
ENSG00000228451	SDAD1P1	183.2975306	116.4877606	0.656816331	0.001099474	0.006546975
ENSG00000163946	FAM208A	5472.173298	4362.496331	0.326913054	0.00110168	0.006558522
ENSG00000167635	ZNF146	4255.485794	3328.786963	0.354096206	0.001102446	0.006561491
ENSG00000204389	HSPA1A	243.0690505	149.2999771	0.700756087	0.001104242	0.006570589
ENSG00000065534	MYLK	109.195221	63.54327496	0.781984337	0.001104867	0.006571406
ENSG00000136643	RPS6KC1	771.7428766	590.2108077	0.386248035	0.001104915	0.006571406
ENSG00000162989	KCNJ3	28.28371754	5.404283909	2.356580294	0.001108512	0.006591202
ENSG00000243566	UPK3B	66.77954779	127.4441638	-0.931954375	0.001111588	0.006607891
ENSG00000056558	TRAF1	243.132641	425.4587838	-0.805803824	0.001114135	0.006621428
ENSG00000177030	DEAF1	1027.455673	1342.444474	-0.385368259	0.001119762	0.00665326
ENSG00000135631	RAB11FIP5	1654.948945	1278.253336	0.37262445	0.001126048	0.006688995

ENSG00000085063	CD59	2285.002959	2894.60709	-0.34084282	0.001126634	0.006690854
ENSG00000138286	FAM149B1	1006.894286	1278.538166	-0.344473822	0.001127629	0.006695143
ENSG00000230454	U73166.1	54.15229768	115.7424478	-1.092930191	0.001128892	0.006701023
ENSG00000156110	ADK	1208.786485	1662.827251	-0.460633796	0.001139815	0.006764224
ENSG00000101096	NFATC2	40.49539379	80.78621705	-0.998693719	0.00114134	0.006771636
ENSG00000269821	KCNQ1OT1	1153.949974	779.1505353	0.567376322	0.001141622	0.006771676
ENSG00000235899	LINC01564	18.25519945	2.933745128	2.614473929	0.001143346	0.006780262
ENSG00000178187	ZNF454	20.24856454	4.013999958	2.352434358	0.001148048	0.006806503
ENSG00000110955	ATP5F1B	14536.53599	19221.21427	-0.403060439	0.001149468	0.006813278
ENSG00000213626	LBH	18.78160738	3.452523378	2.409817921	0.001153158	0.0068335
ENSG00000070010	UFD1	2106.358557	1533.557805	0.45720476	0.001159818	0.006871306
ENSG00000263465	SRSF8	1636.032915	2026.834146	-0.308982363	0.001160197	0.006871896
ENSG00000173065	FAM222B	847.2447767	638.3236923	0.40796998	0.00116166	0.006878901
ENSG00000145246	ATP10D	270.7334569	186.1437058	0.539829789	0.001165061	0.006897377
ENSG00000138623	SEMA7A	150.6761928	80.90768015	0.892804316	0.00117551	0.006957556
ENSG00000101928	MOSPD1	870.9861835	670.7487694	0.376882356	0.001176821	0.006963639
ENSG00000273066	AL355987.4	119.1260699	253.0374173	-1.084594437	0.001178084	0.006969433
ENSG00000106991	ENG	98.35806396	53.2466186	0.88682318	0.00118048	0.006981926
ENSG00000170160	CCDC144A	140.5799716	225.2810822	-0.67736744	0.001182172	0.006990248
ENSG00000261596	AC005632.2	21.81303946	5.625320002	1.941363061	0.001184961	0.007005052
ENSG00000138161	CUZD1	53.16719617	24.10418286	1.146841307	0.001186805	0.007014262
ENSG00000105996	HOXA2	39.64743383	16.06733575	1.305931785	0.00118808	0.007017993
ENSG00000148110	MFSD14B	3397.748697	4173.199353	-0.296606937	0.001188226	0.007017993
ENSG00000250230	AP002754.1	106.8273383	57.01450023	0.908732551	0.001188294	0.007017993
ENSG00000119203	CPSF3	2553.659839	1807.14787	0.49837305	0.001188904	0.007019909
ENSG00000262691	AC040160.1	120.1227525	71.06134383	0.760757274	0.001193769	0.007045358
ENSG00000102312	PORCN	462.9010839	323.3570826	0.516357711	0.001193788	0.007045358
ENSG00000259768	AC004943.2	297.5372149	211.9816101	0.488528817	0.001197225	0.007063942
ENSG00000100292	HMOX1	649.5527061	975.6833182	-0.587800054	0.001200401	0.007080983
ENSG00000158352	SHROOM4	1.121626367	14.29537234	-3.695562527	0.001201144	0.007083665
ENSG00000127328	RAB3IP	967.2570202	1247.091437	-0.36651934	0.001202933	0.007091881
ENSG00000196116	TDRD7	1058.990267	818.7998754	0.370857003	0.001203115	0.007091881
ENSG00000197576	HOXA4	60.2673339	28.25623266	1.096840151	0.001207806	0.007117819
ENSG00000106524	ANKMY2	837.0981299	1115.582471	-0.414570552	0.001209436	0.007125718
ENSG00000181938	GINS3	522.2916246	375.1178579	0.475794595	0.001211511	0.007134602
ENSG00000092036	HAUS4	662.2519569	477.6041841	0.470845955	0.001211728	0.007134602
ENSG00000269553	U62631.1	15.88558156	1.988100376	2.950498485	0.001211816	0.007134602
ENSG00000203688	LINC02487	0	8.273167193	-5.364082243	0.00121599	0.007157462
ENSG00000162521	RBBP4	5087.714442	6216.617604	-0.289094933	0.001219363	0.007175597
ENSG00000197323	TRIM33	3060.387404	3822.95657	-0.321148091	0.00122036	0.00717974
ENSG00000130751	NPAS1	156.1018262	92.98973304	0.750059167	0.001222687	0.007191707
ENSG00000167895	TMC8	458.8636255	335.1726134	0.453588279	0.001224558	0.007199492
ENSG00000254162	AC009812.3	18.10552085	45.08846498	-1.313393471	0.001224597	0.007199492

ENSG00000122778	KIAA1549	1697.616115	1296.850639	0.38800182	0.001226377	0.007208233
ENSG00000158483	FAM86C1	273.583823	181.5674348	0.588572029	0.001227587	0.007213615
ENSG00000186660	ZFP91	2959.83256	3846.509504	-0.378256524	0.001229255	0.007221688
ENSG00000171067	C11orf24	1356.038531	862.9393336	0.651084329	0.0012317	0.007234324
ENSG00000172292	CERS6	1455.315255	1826.076244	-0.327735401	0.001232334	0.007236313
ENSG00000103978	TMEM87A	1307.032131	1687.098812	-0.368005075	0.001233323	0.00724039
ENSG00000136274	NACAD	5.097580154	23.40121667	-2.195680647	0.001240127	0.007278595
ENSG00000269696	AC005498.3	12.64183639	1.263491718	3.368316989	0.001244345	0.007301602
ENSG00000169727	GPS1	3535.522216	2521.772011	0.487102312	0.001246277	0.007311191
ENSG00000085788	DDHD2	2173.095946	2727.043056	-0.327268848	0.001247662	0.00731757
ENSG00000136381	IREB2	3648.785506	4701.627361	-0.365913493	0.0012505	0.007331951
ENSG00000146411	SLC2A12	21.72187152	48.98065014	-1.170051862	0.001250711	0.007331951
ENSG00000186474	KLK12	4.368062831	23.0454922	-2.394930269	0.001253472	0.007346383
ENSG00000099308	MAST3	717.1505954	926.519964	-0.369311342	0.001256946	0.007364983
ENSG00000145476	CYP4V2	635.3088343	948.594338	-0.577811033	0.00125931	0.007377074
ENSG00000229068	TMPOP1	43.92305876	18.72352183	1.231118336	0.001263692	0.007400977
ENSG00000084731	KIF3C	686.4555504	468.6016912	0.549240425	0.001265189	0.007407978
ENSG00000167106	FAM102A	2355.23459	1774.363377	0.408763188	0.001265634	0.007408818
ENSG00000260750	AC092720.1	175.5222595	105.7260085	0.731390467	0.001267766	0.007419528
ENSG00000261114	AC012181.1	9.146468293	36.76067694	-2.00987561	0.001281255	0.007496687
ENSG00000140403	DNAJA4	18.99517921	4.214931242	2.168969476	0.001282803	0.007503953
ENSG00000196663	TECPR2	1157.855875	899.0536191	0.365388766	0.001284028	0.007509333
ENSG00000145592	RPL37	11913.39256	15369.785	-0.367499419	0.001286152	0.007519963
ENSG00000188001	TPRG1	26.52247182	6.29881314	2.100083433	0.001290591	0.007544118
ENSG00000113645	WWC1	6461.254066	5294.79861	0.287254192	0.001294336	0.007562511
ENSG00000284968	AC093827.4	112.2802464	62.96943312	0.839714593	0.001294353	0.007562511
ENSG00000174099	MSRB3	6.156734611	0	5.206536672	0.001297839	0.007581069
ENSG00000134684	YARS	7998.707078	10148.05293	-0.34340528	0.001299806	0.007590753
ENSG00000133678	TMEM254	206.7197486	311.5549339	-0.590287201	0.00130177	0.007600418
ENSG00000198585	NUDT16	1411.525993	1759.274003	-0.317625865	0.001307673	0.007633066
ENSG00000230753	ZNF341-AS1	24.29349142	6.780743236	1.825886634	0.001308227	0.007633179
ENSG00000090006	LTBP4	4224.214781	2709.807825	0.640783247	0.001308314	0.007633179
ENSG00000166920	C15orf48	12.36293876	37.83164183	-1.609809507	0.001308986	0.007635287
ENSG00000116691	MIIP	928.7139127	1241.339831	-0.419098173	0.001310989	0.007644048
ENSG00000158234	FAIM	426.3826141	572.7541136	-0.425903551	0.001311124	0.007644048
ENSG00000049283	EPN3	694.6256302	945.8529896	-0.445695335	0.001311422	0.007644048
ENSG00000172663	TMEM134	1551.563891	1144.128508	0.439618112	0.001329416	0.007747091
ENSG00000137959	IFI44L	0	6.728168354	-5.0653303	0.001330917	0.007753997
ENSG00000196074	SYCP2	33.13124746	10.78245623	1.631433311	0.001331815	0.007757386
ENSG00000170500	LONRF2	72.35153762	31.66795981	1.188895367	0.00133416	0.007769205
ENSG00000152942	RAD17	1218.905237	1541.355649	-0.338692037	0.001334751	0.007770803
ENSG00000263466	AC006441.1	24.40543287	5.866539204	2.038675783	0.001341127	0.00780607
ENSG00000285593	AC055872.2	6.180199419	0	5.211087935	0.001342228	0.007808778

ENSG00000266278	LINC01910	6.180199419	0	5.211087935	0.001342228	0.007808778
ENSG00000068489	PRR11	1227.258789	1735.464905	-0.500418	0.001343998	0.007815502
ENSG00000100605	ITPK1	2469.496441	3077.701802	-0.317863298	0.001344021	0.007815502
ENSG00000137393	RNF144B	77.81156356	146.0605809	-0.907265975	0.001345824	0.007824137
ENSG00000117682	DHDDS	1501.221354	1163.72239	0.36704649	0.001349222	0.007842032
ENSG00000109180	OCIAD1	3710.129216	4584.006323	-0.305107503	0.001349905	0.007844148
ENSG00000228705	LINC00659	179.9244121	104.7980582	0.780099567	0.001352898	0.00785968
ENSG00000232119	MCTS1	1065.196656	792.9715214	0.424943058	0.001354301	0.007865966
ENSG00000123472	ATPAF1	2603.766035	3184.900207	-0.290654804	0.001355993	0.00787393
ENSG00000158125	XDH	351.5331814	234.87756	0.583760988	0.001360191	0.00789644
ENSG00000224418	STK24-AS1	5.513154006	23.33395715	-2.095306934	0.001360699	0.007897524
ENSG00000198018	ENTPD7	379.627434	523.7674566	-0.46398247	0.001361861	0.0079024
ENSG00000108344	PSMD3	6310.192176	4481.587298	0.493461806	0.001364173	0.007913942
ENSG00000262188	LINC01978	12.33947396	50.07551131	-2.016115933	0.001365974	0.007922518
ENSG00000003249	DBNDD1	1588.242359	2180.370842	-0.457571059	0.001368737	0.007936668
ENSG00000174500	GCSAM	117.708885	67.36820059	0.800185885	0.001373861	0.007964503
ENSG00000204439	C6orf47	478.3872536	639.3098074	-0.41820683	0.001376365	0.007977132
ENSG00000167799	NUDT8	584.3913892	397.2572771	0.555642341	0.001376917	0.007978446
ENSG00000101974	ATP11C	2750.158392	2033.114355	0.435385513	0.001377656	0.007980846
ENSG00000253125	AC055854.1	16.18398274	2.736761918	2.574291314	0.001379453	0.007989373
ENSG00000263154	AC110285.3	72.34233392	34.13548884	1.091703218	0.001382974	0.008007876
ENSG00000110756	HPS5	609.9455605	862.2054225	-0.500066285	0.001386182	0.008024558
ENSG00000223855	HRAT92	62.98197984	22.79937387	1.477618828	0.001386804	0.008026267
ENSG00000215241	LINC02449	85.03363986	45.68482205	0.897664993	0.001388398	0.008033599
ENSG00000153714	LURAP1L	9.868214469	30.36468849	-1.626942424	0.001402805	0.008115046
ENSG00000275052	PPP4R3B	4095.102281	5011.287656	-0.291251466	0.001405464	0.008128517
ENSG00000184076	UQCR10	1633.854018	1157.517339	0.49650691	0.001414623	0.008179557
ENSG00000213160	KLHL23	1465.734284	1997.958873	-0.447376194	0.001417425	0.00819383
ENSG00000169635	HIC2	596.0563605	448.0708302	0.41245021	0.0014182	0.008196379
ENSG00000285219	AL591485.1	30.55693626	10.45825078	1.540623338	0.00141935	0.008201096
ENSG00000188827	SLX4	1104.474482	739.1138882	0.578409135	0.001420121	0.008203624
ENSG00000176058	TPRN	1195.90951	1754.158261	-0.552127732	0.001422276	0.008214139
ENSG00000178947	SMIM10L2A	0.734748544	12.62614828	-4.116241152	0.001423443	0.008218944
ENSG00000214485	RPL7P1	96.78281264	156.0761601	-0.688372826	0.001428	0.008243317
ENSG00000165724	ZMYND19	1550.149558	1146.318727	0.434529515	0.001428453	0.008243997
ENSG00000091262	ABCC6	524.2784744	715.3754619	-0.447337481	0.001429304	0.008246964
ENSG00000185818	NAT8L	860.3150042	1127.181525	-0.390335339	0.001437722	0.008293586
ENSG00000118482	PHF3	3805.234595	4864.634093	-0.354487176	0.00143816	0.008294167
ENSG00000131778	CHD1L	3099.122788	2193.369288	0.49824897	0.001442509	0.008317295
ENSG00000132388	UBE2G1	1777.541538	2268.877557	-0.352358057	0.001443812	0.008322013
ENSG00000261211	AL031123.2	72.38403232	34.35806259	1.082223746	0.001444478	0.008322013
ENSG00000225855	RUSC1-AS1	623.4971896	458.2468306	0.444841434	0.001444644	0.008322013
ENSG00000196155	PLEKHG4	2693.806121	1723.836087	0.644473889	0.001444683	0.008322013

ENSG00000129757	CDKN1C	332.9215355	232.6107544	0.517085655	0.001447611	0.008336921
ENSG00000233483	AC008105.2	103.298043	58.85965164	0.814816574	0.001449051	0.008343256
ENSG00000174028	FAM3C2	263.2339043	165.4386114	0.66676507	0.001451053	0.008352109
ENSG00000249898	MCPH1-AS1	50.19188619	22.6471477	1.148417322	0.001451269	0.008352109
ENSG00000171428	NAT1	36.17172065	73.65859654	-1.027667727	0.001454404	0.008368194
ENSG00000258689	LINC01269	15.99894434	2.804542483	2.538054713	0.001455273	0.008371007
ENSG00000158792	SPATA2L	610.7770334	813.8066386	-0.4134483	0.001455575	0.008371007
ENSG00000163808	KIF15	728.3026519	1040.768909	-0.515538058	0.001457048	0.008377517
ENSG00000212694	LINC01089	278.7757502	454.3910894	-0.703146795	0.001459724	0.008390935
ENSG00000135862	LAMC1	8730.040001	7014.63339	0.315626707	0.001465642	0.008421451
ENSG00000172037	LAMB2	13159.41804	9472.369744	0.474408555	0.001465719	0.008421451
ENSG00000185900	POMK	686.6315447	953.4005843	-0.473430188	0.001468819	0.00843729
ENSG00000086570	FAT2	91.30770973	48.50800998	0.915365846	0.001474367	0.008467178
ENSG00000135750	KCNK1	712.0555328	928.0870751	-0.382756981	0.001476996	0.008480295
ENSG00000112306	RPS12	13074.57617	16309.57354	-0.318988607	0.001479852	0.008494704
ENSG00000051825	MPHOSPH9	1182.483946	1562.097449	-0.402076652	0.001482009	0.008505096
ENSG00000146205	ANO7	104.8848754	57.98229576	0.861316773	0.001482952	0.008508522
ENSG00000166188	ZNF319	861.9608238	651.7711548	0.403604521	0.001483299	0.008508523
ENSG00000113013	HSPA9	15819.54429	20609.48036	-0.381653141	0.001483976	0.008510415
ENSG00000161013	MGAT4B	8714.176564	6900.937853	0.336572056	0.001486465	0.008522701
ENSG00000278611	ZNF426-DT	71.01093439	34.7319242	1.031797717	0.001486888	0.008522921
ENSG00000167378	IRGQ	1410.840737	1102.045114	0.355785779	0.001487198	0.008522921
ENSG00000241622	RARRES2P1	0	6.82738964	-5.083728329	0.001487723	0.008523939
ENSG00000096654	ZNF184	470.9092791	350.7771073	0.424657283	0.001488109	0.008524164
ENSG00000162804	SNED1	122.389784	191.328322	-0.641571181	0.001489108	0.008527011
ENSG00000258334	AC125611.4	9.558080888	31.43609612	-1.724975027	0.001489301	0.008527011
ENSG00000001167	NFYA	1300.916952	1642.130782	-0.335591285	0.001490108	0.008529641
ENSG00000228889	UBAC2-AS1	49.57415895	95.33810531	-0.947984245	0.001493132	0.008543251
ENSG00000175772	LINC01106	153.8271749	231.8595131	-0.59082623	0.001493513	0.008543251
ENSG00000197813	AC011450.1	47.92262872	19.18431776	1.31774124	0.001493529	0.008543251
ENSG00000070495	JMJD6	788.1485162	568.218768	0.470632307	0.001495596	0.008551279
ENSG00000260916	CCPG1	277.8278981	400.4427816	-0.526475993	0.001495629	0.008551279
ENSG00000112659	CUL9	1962.170631	1547.839236	0.342559943	0.001497288	0.008558771
ENSG00000164292	RHOBTB3	12082.31872	14784.92003	-0.291214682	0.001498913	0.008566065
ENSG00000267080	ASB16-AS1	695.9078658	499.3078883	0.480474072	0.001501582	0.008579321
ENSG00000073605	GSDMB	863.6786246	626.5156791	0.46412471	0.001506745	0.008606818
ENSG00000131236	CAP1	5380.661898	4057.126734	0.407106943	0.001513495	0.008643362
ENSG00000256771	ZNF253	321.375018	229.652357	0.486370602	0.001514929	0.008649538
ENSG00000279672	AP006621.5	137.1413676	212.4766872	-0.632382732	0.001517272	0.008660903
ENSG00000166337	TAF10	513.9631586	748.3207632	-0.542429583	0.001518105	0.008663646
ENSG00000156256	USP16	1946.20218	2444.522007	-0.329039275	0.001520761	0.008676785
ENSG00000159917	ZNF235	185.9355837	100.1550617	0.897764312	0.001526861	0.008709563
ENSG00000159674	SPON2	96.47045309	152.5790491	-0.661337613	0.001531511	0.00873406

ENSG00000117450	PRDX1	15836.30569	10516.99967	0.590425027	0.001536765	0.008761987
ENSG00000261534	AL596244.1	39.3258818	15.44933621	1.352809318	0.001537439	0.008763796
ENSG00000086062	B4GALT1	3886.697081	4871.914584	-0.325901728	0.001544148	0.008799997
ENSG00000183023	SLC8A1	66.38221874	29.66069294	1.172741976	0.001545173	0.008803794
ENSG00000197150	ABCB8	1763.744835	1270.523338	0.472560447	0.001550124	0.008829953
ENSG00000205810	KLRC3	24.0914894	6.49579635	1.906145181	0.001551111	0.008833523
ENSG00000164587	RPS14	18672.78256	23331.3859	-0.321328706	0.001555041	0.008853851
ENSG00000139549	DHH	16.6620285	3.350813379	2.322470155	0.001565834	0.008913238
ENSG00000107036	RIC1	1925.099911	1547.821477	0.314363296	0.00157401	0.008957703
ENSG00000165105	RASEF	1748.130032	1418.02283	0.302001831	0.001576151	0.008967806
ENSG00000109929	SC5D	1906.328909	1532.452821	0.31483203	0.001577173	0.008971545
ENSG00000099337	KCNK6	677.1611193	923.4352426	-0.447948321	0.001578191	0.008975252
ENSG00000198707	CEP290	752.6763611	994.2370366	-0.402192185	0.00157964	0.008979481
ENSG00000090615	GOLGA3	6531.619514	4953.844834	0.399015664	0.001579666	0.008979481
ENSG00000088836	SLC4A11	334.4961701	234.799356	0.508876434	0.001585129	0.00900845
ENSG00000080986	NDC80	819.1114051	1199.763438	-0.551245284	0.001586335	0.009013214
ENSG00000176244	ACBD7	115.070832	209.0065092	-0.862914503	0.001591866	0.009042553
ENSG00000257103	LSM14A	4592.407855	5782.508981	-0.332582376	0.001593253	0.009048334
ENSG00000086619	ERO1B	2001.831384	2883.161585	-0.526093905	0.001594532	0.009053504
ENSG00000204792	LINC01291	217.098526	136.7655585	0.670942102	0.00159526	0.009055543
ENSG00000105323	HNRNPUL1	11603.11517	14785.44822	-0.349715934	0.001598709	0.009073026
ENSG00000239893	ZNF736P9Y	17.06983121	2.897405283	2.528164919	0.001604821	0.009105609
ENSG00000126107	HECTD3	1381.728423	1065.291539	0.374554583	0.00160832	0.009123349
ENSG00000256955	AC131009.2	1.090390412	11.75808234	-3.431753142	0.001609539	0.009128158
ENSG00000258077	AC078923.1	170.4213489	112.0090949	0.607049588	0.001613629	0.009149241
ENSG00000119899	SLC17A5	1105.129632	831.8078979	0.40882187	0.00161544	0.009157395
ENSG00000052795	FNIP2	1045.145785	788.7905597	0.405456457	0.0016235	0.009200956
ENSG00000166454	ATMIN	2522.255554	2044.965312	0.302416559	0.001627595	0.009222038
ENSG00000147912	FBXO10	190.1525466	270.2193142	-0.507415356	0.00162979	0.009232345
ENSG00000132746	ALDH3B2	9.464373022	29.37282734	-1.631281535	0.001632464	0.009245356
ENSG00000084710	EFR3B	221.6203801	340.8517598	-0.621765119	0.001635184	0.009258625
ENSG00000031003	FAM13B	566.3031488	838.7763863	-0.565959319	0.001635758	0.009259079
ENSG00000172340	SUCLG2	1934.595528	2413.820839	-0.319361575	0.001636018	0.009259079
ENSG00000106105	GARS	7758.731063	10210.47835	-0.396234632	0.00163825	0.009269575
ENSG00000263006	ROCK1P1	106.4127205	185.8507062	-0.803474798	0.001641538	0.009284371
ENSG00000253094	RF00049	6.255673697	0	5.225770327	0.001641622	0.009284371
ENSG00000067365	METTL22	1214.191171	862.2285498	0.492853207	0.001647306	0.009314373
ENSG00000279255	Z97653.2	38.28606831	14.13816874	1.446274962	0.00165207	0.009339157
ENSG00000124006	OBSL1	21.37669209	4.443355173	2.254286126	0.001657229	0.00936617
ENSG00000249857	AC027338.2	8.423452153	0.331593289	4.693972033	0.001663116	0.009397277
ENSG00000118855	MFSD1	886.1477817	699.6890366	0.340605292	0.001665329	0.009407006
ENSG00000251537	AC005324.3	42.62020389	14.01343521	1.588864287	0.001665605	0.009407006
ENSG00000064225	ST3GAL6	19.9879005	5.11538895	1.964100834	0.001666042	0.009407313

ENSG00000235865	GSN-AS1	44.05861639	17.66096139	1.31414326	0.001668031	0.00941638
ENSG00000234345	AC234782.3	30.68330142	10.34279446	1.572620027	0.001669812	0.009424267
ENSG00000198625	MDM4	1672.319442	2066.458795	-0.305149115	0.001673597	0.009443455
ENSG00000213213	CCDC183	107.9708456	199.5644589	-0.883503109	0.001679951	0.00947713
ENSG00000185633	NDUFA4L2	121.4450997	205.8279087	-0.759496105	0.001680672	0.009479024
ENSG00000248334	WHAMMP2	0.349140684	9.302827546	-4.575295294	0.001688812	0.009522742
ENSG00000239552	HOXB-AS2	791.7839051	482.523423	0.716048508	0.001691657	0.009536598
ENSG00000112851	ERBIN	5323.994173	6682.120274	-0.327869037	0.001697697	0.009568445
ENSG00000185942	NKAIN3	5.819326331	0	5.12413857	0.001706731	0.009617155
ENSG00000131848	ZSCAN5A	157.7615929	103.1292641	0.613875666	0.001708459	0.009624683
ENSG00000233532	LINC00460	8.299778295	0.331593289	4.676746864	0.001711687	0.009640658
ENSG00000111142	METAP2	3845.032461	4844.184704	-0.333409733	0.001715924	0.009660251
ENSG00000075711	DLG1	5261.023648	6504.878629	-0.306134913	0.001716134	0.009660251
ENSG00000153187	HNRNPU	24328.80073	29221.94458	-0.264395526	0.001716346	0.009660251
ENSG00000147119	CHST7	99.36886746	155.4656267	-0.644721433	0.001726774	0.009716714
ENSG00000109814	UGDH	3063.401561	4210.814463	-0.459150405	0.001727835	0.00972046
ENSG00000119820	YIPF4	2483.580385	3138.68351	-0.337684638	0.001738879	0.009780349
ENSG00000175063	UBE2C	1808.137507	2632.614625	-0.542258242	0.001739351	0.00978076
ENSG00000203711	C6orf99	15.37899114	47.59973443	-1.624175656	0.001741964	0.00979321
ENSG00000251034	AC037459.2	10.55999465	30.43641712	-1.529285208	0.001748674	0.009828684
ENSG00000112494	UNC93A	0	6.441762108	-5.002092268	0.001749418	0.009830617
ENSG00000162702	ZNF281	1837.274729	1429.75957	0.361254372	0.001754523	0.009857047
ENSG00000086061	DNAJA1	3083.854965	2389.809545	0.367445273	0.001756203	0.009864227
ENSG00000275111	ZNF2	237.0109522	161.7198522	0.549247987	0.001757908	0.009871546
ENSG00000148204	CRB2	352.3595744	557.3179246	-0.659846418	0.001778606	0.009985054
ENSG00000232098	AC012313.1	721.7831097	551.0088702	0.390545013	0.001779306	0.009985054
ENSG00000170946	DNAJC24	367.2414845	496.5088185	-0.436167768	0.001779341	0.009985054
ENSG00000116741	RGS2	10.97160725	31.57465427	-1.527587933	0.001784631	0.010012448
ENSG00000265750	AC090772.3	33.64433907	11.23294561	1.56911107	0.001786197	0.010018945
ENSG00000256546	AC156455.1	10.78673144	36.28664299	-1.741017369	0.001787316	0.010022933
ENSG00000148343	MIGA2	1441.019357	1803.061319	-0.32348881	0.001788118	0.010025138
ENSG00000123329	ARHGAP9	7.686163682	34.07845757	-2.150144073	0.001788628	0.010025709
ENSG00000169519	METTL15	695.5956801	927.9829165	-0.415586382	0.001791793	0.01004116
ENSG00000138433	CIR1	779.6645042	1006.308144	-0.367693144	0.001793252	0.010045837
ENSG00000280193	AC132219.2	63.70007872	115.0203406	-0.849033118	0.001793446	0.010045837
ENSG00000285980	AP003025.2	5.832328698	0	5.126768188	0.001795678	0.010055722
ENSG00000250644	AC068580.4	21.80003709	49.74752712	-1.194148674	0.001796408	0.010055722
ENSG00000173812	EIF1	11309.88837	15208.19824	-0.42722756	0.00179644	0.010055722
ENSG00000125531	FNDC11	231.490341	138.1881559	0.748280994	0.001798408	0.01006316
ENSG00000277831	AL138960.1	1.439531096	12.35348836	-3.092002743	0.001798589	0.01006316
ENSG00000272980	Z94721.2	13.8129323	36.16585754	-1.389493621	0.001802101	0.010080517
ENSG00000180340	FZD2	884.9549451	1133.706807	-0.357338117	0.001804525	0.01009135
ENSG00000138614	INTS14	1870.184631	2347.336379	-0.327979578	0.00180486	0.01009135

ENSG00000163623	NKX6-1	66.49447415	32.66470735	1.032942056	0.001808496	0.010109377
ENSG00000117139	KDM5B	1936.375579	1429.922924	0.438088388	0.00181113	0.010121798
ENSG00000267679	EIF5AP2	0	6.969387557	-5.118211169	0.001815389	0.010141278
ENSG00000087589	CASS4	71.13731076	36.2336381	0.973924655	0.001815442	0.010141278
ENSG00000012232	EXTL3	2523.657309	1947.110412	0.373774184	0.001816631	0.010145609
ENSG00000168010	ATG16L2	721.7425382	1038.023337	-0.523242395	0.001818411	0.010153244
ENSG00000146085	MUT	1314.997865	1662.291637	-0.337896154	0.001819251	0.010155623
ENSG00000145107	TM4SF19	37.7975601	14.12545176	1.40609182	0.001820774	0.010161814
ENSG00000088899	LZTS3	559.0487292	748.399634	-0.420552823	0.001831929	0.010221747
ENSG00000259275	AC087477.2	148.1952643	93.22854183	0.669339486	0.001833119	0.010224794
ENSG00000117036	ETV3	1238.254929	978.0010047	0.340119157	0.001833308	0.010224794
ENSG00000101639	CEP192	1658.028952	2150.047681	-0.375042369	0.001834141	0.010227115
ENSG00000267882	AL031666.2	5.177015689	22.72721522	-2.149037591	0.00183477	0.010228299
ENSG00000006534	ALDH3B1	2671.355999	3485.079366	-0.383278511	0.001840877	0.010260015
ENSG00000273812	BX640514.2	3.277672419	16.88377785	-2.36228405	0.001844302	0.010276773
ENSG00000064652	SNX24	446.3273919	595.1316894	-0.41466378	0.0018483	0.010296711
ENSG00000163512	AZI2	702.3619055	907.9299188	-0.370769312	0.001855097	0.010332234
ENSG00000229214	LINC00242	16.26341827	39.61742986	-1.282765049	0.001855769	0.010333632
ENSG00000133835	HSD17B4	3410.391464	4177.37273	-0.29263898	0.001856278	0.010334121
ENSG00000232187	FTH1P7	60.60601214	28.23861657	1.095354665	0.001857968	0.010341187
ENSG00000023608	SNAPC1	378.2354716	264.3746523	0.515600394	0.001870558	0.010408901
ENSG00000240499	AC004594.1	0.72301614	11.22555777	-3.949954259	0.001872387	0.010415398
ENSG00000054118	THRAP3	7686.99346	10325.6423	-0.425824703	0.001872816	0.010415398
ENSG00000226053	LINC01776	17.32511266	3.296779137	2.390361589	0.001872998	0.010415398
ENSG00000066117	SMARCD1	3200.973091	2572.41584	0.31515574	0.001873577	0.010416258
ENSG00000167962	ZNF598	2966.222961	3784.755035	-0.351739206	0.001881585	0.010458411
ENSG00000140598	EFL1	1341.750547	1054.648944	0.346800387	0.001886963	0.010485927
ENSG00000213676	ATF6B	2497.537246	3046.437958	-0.286542116	0.001888136	0.010490074
ENSG00000156510	HKDC1	9.506071418	29.53595942	-1.640188678	0.001892821	0.01051372
ENSG00000197324	LRP10	10516.42323	8467.845964	0.312649902	0.001894962	0.010523233
ENSG00000139263	LRIG3	365.6912931	504.9865133	-0.466678544	0.001897932	0.01053734
ENSG00000169692	AGPAT2	1316.967429	1025.170171	0.36172936	0.001898595	0.010538642
ENSG00000119321	FKBP15	2010.786638	1615.219777	0.315866158	0.001901353	0.010551565
ENSG00000115616	SLC9A2	536.1164295	386.1041768	0.471648079	0.001903696	0.010562176
ENSG00000125676	THOC2	4145.489539	5155.302454	-0.314588734	0.001907481	0.010580786
ENSG00000114378	HYAL1	62.05062309	106.1764851	-0.77350038	0.001912311	0.010603396
ENSG00000159200	RCAN1	443.9138526	303.842853	0.545405917	0.001912421	0.010603396
ENSG00000160767	FAM189B	1466.14036	1974.828245	-0.430060288	0.001919952	0.010642749
ENSG00000255145	STX17-AS1	66.09570134	34.61646788	0.936979692	0.001929122	0.010691171
ENSG00000185917	SETD4	602.8948923	780.7888136	-0.373774191	0.001938726	0.010741971
ENSG00000253438	PCAT1	56.89453228	98.4038935	-0.789983566	0.00194021	0.010747763
ENSG00000168301	KCTD6	379.7712281	280.7650859	0.435672133	0.001941788	0.010754082
ENSG00000100811	YY1	5214.252342	6381.897519	-0.291574878	0.001943025	0.010758506

ENSG00000164576	SAP30L	747.0039967	1031.608572	-0.464954928	0.001944626	0.010762676
ENSG00000124702	KLHDC3	2123.216549	2713.005101	-0.353806835	0.001944655	0.010762676
ENSG00000271664	AC004890.3	40.57118202	16.27806528	1.317861591	0.001949565	0.010787418
ENSG00000175334	BANF1	3651.639057	2615.864099	0.480921595	0.001951111	0.010793537
ENSG00000169499	PLEKHA2	2930.351079	2324.522751	0.334218079	0.001958875	0.01083405
ENSG00000014257	ACPP	204.3122422	122.3792254	0.743134973	0.001960812	0.010842317
ENSG00000272269	AL138724.1	24.41843524	7.421336293	1.706437425	0.001964414	0.01085979
ENSG00000160282	FTCD	8.71662211	29.33399878	-1.74501794	0.001970923	0.010893318
ENSG00000067113	PLPP1	241.8518296	343.5256005	-0.504538159	0.001973602	0.01090567
ENSG00000144711	IQSEC1	2382.128378	1942.418451	0.29453521	0.00198231	0.010951323
ENSG00000270469	AC006557.5	11.2947432	0.832677143	3.677865036	0.001983713	0.010956613
ENSG00000203799	CCDC162P	682.3777924	490.5508233	0.477574273	0.001991256	0.010994533
ENSG00000157181	ODR4	556.2394447	736.2314141	-0.403967302	0.001991474	0.010994533
ENSG00000112186	CAP2	945.3965858	690.8857824	0.451989154	0.001993902	0.011005461
ENSG00000182168	UNC5C	8.018340742	0.277559048	4.624174333	0.00199577	0.011013293
ENSG00000185670	ZBTB3	342.2297512	492.1740659	-0.522365131	0.002002884	0.01105007
ENSG00000167460	TPM4	16369.31526	21229.83782	-0.375136723	0.002003687	0.011052014
ENSG00000218226	TATDN2P2	153.1120813	232.3160093	-0.602080509	0.002005872	0.01106158
ENSG00000266896	AL354892.3	16.08488106	40.97129567	-1.353084308	0.00201082	0.011086072
ENSG00000196739	COL27A1	43.18577029	82.07858247	-0.922814837	0.002011216	0.011086072
ENSG00000162735	PEX19	1487.749813	1147.350735	0.374488905	0.002013215	0.011094599
ENSG00000099219	ERMP1	773.8354947	594.6833243	0.379776583	0.002025534	0.011159983
ENSG00000176624	MEX3C	2386.3831	2942.216399	-0.30223735	0.002028718	0.011171179
ENSG00000205609	EIF3CL	130.2164338	196.9428927	-0.594584237	0.002028854	0.011171179
ENSG00000158710	TAGLN2	5742.077129	4306.837027	0.414785993	0.002029075	0.011171179
ENSG00000269940	AL049840.3	31.42836103	65.19634228	-1.054409228	0.00202954	0.011171179
ENSG00000242960	FTH1P23	36.51166886	14.00750673	1.385887843	0.002029841	0.011171179
ENSG00000165275	TRMT10B	355.3188768	479.5491036	-0.431538743	0.002031835	0.011179334
ENSG00000145861	C1QTNF2	129.1495082	78.17813673	0.728254593	0.002032234	0.011179334
ENSG00000271447	MMP28	14.78742	38.5012652	-1.372281314	0.00203612	0.011195273
ENSG00000285570	AL590666.4	25.02950993	7.176677326	1.824518386	0.002036147	0.011195273
ENSG00000272077	AC124045.1	96.32569176	53.26233259	0.860837505	0.002036499	0.011195273
ENSG00000210082	MT-RNR2	77525.6041	103050.3873	-0.410599684	0.002038503	0.01120378
ENSG00000255468	AP001107.9	158.544541	235.2583667	-0.567363366	0.002047031	0.011248133
ENSG00000158966	CACHD1	579.8378114	437.4403394	0.405317068	0.002049286	0.011258004
ENSG00000119689	DLST	6212.068079	4814.412451	0.367508339	0.002052117	0.011271034
ENSG00000107625	DDX50	712.0143363	927.1927665	-0.381586462	0.00206054	0.011314765
ENSG00000072201	LNX1	371.8572202	266.5776127	0.482466544	0.002068024	0.011353325
ENSG00000004799	PDK4	32.00423851	66.81899824	-1.063087528	0.00207027	0.011363111
ENSG00000012171	SEMA3B	5294.877474	8173.372646	-0.62622065	0.002084401	0.011438113
ENSG00000198914	POU3F3	197.5664652	131.7843623	0.583574806	0.002086942	0.011449502
ENSG00000077235	GTF3C1	3937.399536	3125.31875	0.333068835	0.002088431	0.011455013
ENSG00000168264	IRF2BP2	2894.549884	4109.983617	-0.505558547	0.00208888	0.011455013

ENSG00000249421	ADAMTS19-AS1	16.39486328	2.574659192	2.630857456	0.002093868	0.011479804
ENSG00000143079	CTTNBP2NL	1131.980014	875.5330506	0.369948289	0.00209498	0.011483332
ENSG00000197191	CYSRT1	152.435692	252.8252793	-0.728572522	0.002099808	0.011507228
ENSG00000186615	KTN1-AS1	220.0246804	151.7168989	0.537841424	0.002108412	0.011551804
ENSG00000103067	ESRP2	3418.403298	4127.467571	-0.27202136	0.002110148	0.011558736
ENSG00000259583	AC015712.2	62.12497877	115.9694123	-0.897193203	0.002113706	0.011575642
ENSG00000250616	AC012645.1	8.044345477	28.04791354	-1.801132436	0.002114307	0.011576349
ENSG00000255561	FDXACB1	20.76815733	49.70482879	-1.265547612	0.002118831	0.011598532
ENSG00000135083	CCNJL	365.3825809	267.4846109	0.448521534	0.002119652	0.011600442
ENSG00000099984	GSTT2	12.42160079	34.79334628	-1.491035967	0.002126838	0.011637173
ENSG00000120215	MLANA	81.99839789	41.58826585	0.985359983	0.002127967	0.011640755
ENSG00000122378	PRXL2A	1371.245453	1091.171972	0.329109873	0.002131897	0.011659658
ENSG00000127580	WDR24	711.0101936	537.3993817	0.404043114	0.002134576	0.011671712
ENSG00000269190	FBXO17	2.915529367	15.41299636	-2.404641053	0.002135552	0.011674445
ENSG00000196724	ZNF418	27.65440926	7.504322542	1.901063582	0.00214146	0.011704136
ENSG00000257771	LINC02395	2.223749183	15.12804948	-2.778414372	0.00214332	0.011711698
ENSG00000267034	AC010980.2	12.19629655	1.294932438	3.303159987	0.002145713	0.011722163
ENSG00000141349	G6PC3	2342.589261	1643.3995	0.510893597	0.002148593	0.011735284
ENSG00000175262	C1orf127	59.05945686	18.45327231	1.683300732	0.002159449	0.011791957
ENSG00000114331	ACAP2	2014.007117	2509.734937	-0.317573669	0.002166052	0.011825383
ENSG00000261270	AC012181.2	16.17621159	45.72357231	-1.492020032	0.002169442	0.011841255
ENSG00000175061	LRRC75A-AS1	5525.580311	7342.979035	-0.410152116	0.002172666	0.011856216
ENSG00000075151	EIF4G3	6334.953307	7786.868689	-0.297771041	0.002174226	0.011862096
ENSG00000129355	CDKN2D	204.1181627	295.457591	-0.535075587	0.002176394	0.011871282
ENSG00000214279	SCART1	411.5520602	294.2703228	0.48598321	0.002181928	0.011898823
ENSG00000092295	TGM1	169.2307494	251.1175272	-0.567442921	0.002187306	0.011925503
ENSG00000231769	AL035701.1	23.2447994	6.503184188	1.824366384	0.002188564	0.011929716
ENSG00000144381	HSPD1	19829.59317	28437.33363	-0.520166803	0.002190644	0.011938403
ENSG00000254531	FLJ20021	80.63561103	42.97168301	0.903130148	0.00219282	0.011946384
ENSG00000008394	MGST1	1671.29821	1176.420738	0.505863753	0.002193082	0.011946384
ENSG00000196935	SRGAP1	1506.451823	1182.026996	0.349734523	0.002203488	0.012000408
ENSG00000244687	UBE2V1	374.8927649	272.4217277	0.46013534	0.00221769	0.012075075
ENSG00000164620	RELL2	461.4166978	338.7047088	0.444774398	0.002218397	0.012076246
ENSG00000117479	SLC19A2	810.1087056	1046.203003	-0.369537222	0.002226653	0.012117879
ENSG00000270372	AL162413.1	38.05299292	14.83674411	1.373389602	0.002227032	0.012117879
ENSG00000113161	HMGCR	4756.85695	3781.994813	0.330686999	0.002235631	0.012161973
ENSG00000085377	PREP	1500.780274	1112.59649	0.431137473	0.00224049	0.012185704
ENSG00000172757	CFL1	14318.79128	9437.017587	0.601411458	0.00225114	0.012240915
ENSG00000122641	INHBA	10.17961801	0.555118095	4.105267979	0.002253266	0.012249761
ENSG00000164896	FASTK	1949.656127	2456.530377	-0.33308376	0.002256402	0.012264096
ENSG00000271646	AC099343.3	53.93841189	93.22467206	-0.792160437	0.002258381	0.012272134
ENSG00000125611	CHCHD5	610.4009207	463.1188248	0.39866178	0.002265515	0.012308177
ENSG00000134709	HOOK1	4009.183586	4842.469576	-0.272465258	0.002272696	0.01234446

ENSG00000267009	AC007780.1	32.19069823	11.21575952	1.525926552	0.002277589	0.012368302
ENSG00000072163	LIMS2	5.794591559	22.84609858	-1.970978495	0.002282005	0.012388552
ENSG00000114124	GRK7	20.64844473	5.787422728	1.843389715	0.002282327	0.012388552
ENSG00000074800	ENO1	60115.34761	46090.03429	0.383260186	0.002287124	0.012411843
ENSG00000230736	AL021937.1	32.55411124	11.6377269	1.494537013	0.002294478	0.012449001
ENSG00000008294	SPAG9	4651.929117	3750.328206	0.31057751	0.002298493	0.012468031
ENSG00000144847	IGSF11	13.17585288	1.809762614	2.861351841	0.002300202	0.012474546
ENSG00000100478	AP4S1	344.4606099	502.5447047	-0.543964136	0.002301946	0.012481245
ENSG00000258839	MC1R	329.3960276	214.3359494	0.620794523	0.002303967	0.012489444
ENSG00000118705	RPN2	8671.023855	10541.2483	-0.281820015	0.002305158	0.01249314
ENSG00000016082	ISL1	42.35826989	16.99777481	1.309701022	0.002306912	0.012499886
ENSG00000279196	AC135048.4	21.13949286	48.3164342	-1.19055784	0.002307821	0.012502056
ENSG00000164051	CCDC51	780.8585854	1010.152523	-0.371931886	0.002312393	0.01252406
ENSG00000079156	OSBPL6	294.3346916	465.732755	-0.664011153	0.002316796	0.012545137
ENSG00000168056	LTBP3	1455.554994	2185.645308	-0.586166336	0.002327005	0.012597642
ENSG00000159023	EPB41	3871.110736	4755.045966	-0.296783502	0.002328316	0.01260196
ENSG00000174749	FAM241A	342.2521115	220.8380784	0.628988233	0.002329322	0.012604627
ENSG00000008130	NADK	2862.077925	2316.346575	0.305069424	0.00233898	0.012654098
ENSG00000167311	ART5	2.149544868	14.10921673	-2.703167286	0.002339983	0.012656733
ENSG00000170468	RIOX1	423.6086753	602.014494	-0.508143256	0.002341204	0.012660553
ENSG00000231793	DOC2GP	122.0157459	62.17601454	0.979555347	0.002344193	0.012673921
ENSG00000123453	SARDH	1.782170596	13.97511496	-2.951954436	0.002347576	0.012684715
ENSG00000178700	DHFR2	249.0180978	344.3967545	-0.466965871	0.002347612	0.012684715
ENSG00000186272	ZNF17	201.2822315	136.0405981	0.567526604	0.002348222	0.012684715
ENSG00000170873	MTSS1	707.1339528	505.4231399	0.484413201	0.002348303	0.012684715
ENSG00000108551	RASD1	46.56238179	20.8310266	1.152716282	0.002348772	0.012684715
ENSG00000276700	RNA5-8SN2	30.33955454	9.890337412	1.614696654	0.002350329	0.01269033
ENSG00000147400	CETN2	862.3597733	674.3770123	0.354227913	0.002352536	0.012699453
ENSG00000173621	LRFN4	1666.23232	1242.406524	0.42273512	0.002356234	0.012716621
ENSG00000149926	FAM57B	197.8204879	122.7177638	0.693102257	0.002365512	0.012763889
ENSG00000113318	MSH3	810.5084456	1026.0539	-0.339956115	0.002368828	0.012777157
ENSG00000188191	PRKAR1B	1541.561667	1196.025614	0.366247957	0.002369012	0.012777157
ENSG00000134352	IL6ST	3194.945273	2584.923079	0.305489418	0.002371525	0.012787898
ENSG00000271614	ATP2B1-AS1	44.85045425	18.53633686	1.277201528	0.002372331	0.012789438
ENSG00000117308	GALE	2625.033088	1838.887166	0.513050405	0.002373481	0.012792829
ENSG00000171224	FAM241B	324.8683368	209.0313693	0.632992982	0.002376667	0.012807188
ENSG00000284828	AC012020.2	1.090390412	11.84501666	-3.434179253	0.002377639	0.012809615
ENSG00000149418	ST14	9951.335112	12435.91546	-0.321586298	0.002381095	0.012825421
ENSG00000133216	EPHB2	1259.676652	1005.919703	0.324498825	0.002383583	0.012836003
ENSG00000090612	ZNF268	428.9104722	296.7859576	0.533724541	0.002385146	0.012841603
ENSG00000231638	LUARIS	13.07168257	2.172796624	2.6138762	0.002395374	0.012893847
ENSG00000136936	XPA	470.6460864	609.0434802	-0.372204973	0.002398475	0.012902425
ENSG00000100307	CBX7	900.9272763	1189.603685	-0.400120715	0.002398516	0.012902425

ENSG00000115234	SNX17	3914.3007	3044.64086	0.362159938	0.002398545	0.012902425
ENSG00000237512	UNC5B-AS1	35.83320499	14.44081002	1.311076919	0.002401207	0.012913919
ENSG00000154556	SORBS2	269.5951808	434.9229725	-0.688095222	0.002405976	0.012935986
ENSG00000227097	RPS28P7	131.9792522	201.0774174	-0.608261252	0.002406364	0.012935986
ENSG00000136044	APPL2	1196.637292	1479.44535	-0.305764936	0.00240929	0.012948878
ENSG00000197815	AC122129.1	176.4872183	259.3277474	-0.553848346	0.002412966	0.0129658
ENSG00000171612	SLC25A33	1066.588305	1389.808592	-0.382491342	0.002413979	0.012968401
ENSG00000079999	KEAP1	4190.001453	3275.169179	0.355131196	0.002422568	0.013011695
ENSG00000138685	FGF2	281.7307661	425.1382142	-0.595343261	0.002425834	0.013026391
ENSG00000154839	SKA1	738.9742628	1018.529342	-0.46374651	0.002430256	0.013046784
ENSG00000267419	AC011477.1	48.2338809	22.75375682	1.080324709	0.002430695	0.013046784
ENSG00000186280	KDM4D	72.96117976	35.73512126	1.021276256	0.002434515	0.013064432
ENSG00000147155	EBP	3239.775725	2366.531905	0.452739167	0.002455314	0.013173168
ENSG00000204138	PHACTR4	2124.053093	2742.962763	-0.369266643	0.002455974	0.01317383
ENSG00000160326	SLC2A6	432.5551937	301.6739389	0.517491075	0.002457001	0.013176457
ENSG00000146122	DAAM2	15.33189893	2.996626568	2.348160236	0.002473056	0.013259663
ENSG00000130768	SMPDL3B	900.5809587	1143.910327	-0.345352392	0.002479374	0.013290637
ENSG00000079805	DNM2	8775.176064	10739.03138	-0.291294085	0.00248058	0.013294199
ENSG00000163155	LYSMD1	271.2719224	374.1843307	-0.463456519	0.002481995	0.013298877
ENSG00000227906	SNAP25-AS1	5.458453242	0	5.03162518	0.0024834	0.013303505
ENSG00000183091	NEB	418.192086	301.861228	0.470828987	0.002484546	0.01330674
ENSG00000260470	AC023794.5	35.56476981	13.59584591	1.401519356	0.002486341	0.013313445
ENSG00000241186	TDGF1	67.74215172	27.26532257	1.321176081	0.002498221	0.013373399
ENSG00000196502	SULT1A1	691.1508618	949.7215652	-0.457630074	0.002499258	0.013373399
ENSG00000265415	AC099850.3	146.0939302	225.7166263	-0.630063933	0.002499306	0.013373399
ENSG00000226445	BX322234.1	6.260904917	23.22734803	-1.904445295	0.002499716	0.013373399
ENSG00000154529	CNTNAP3B	33.29932223	12.05084707	1.481011479	0.002500314	0.013373686
ENSG00000112812	PRSS16	918.3104006	1375.713433	-0.582593641	0.002511888	0.013432662
ENSG00000115268	RPS15	18074.8967	22263.06318	-0.300690624	0.002513932	0.013439018
ENSG00000228343	AC115618.2	155.5452897	232.8002587	-0.581298576	0.002514171	0.013439018
ENSG00000133424	LARGE1	612.6990368	797.1562109	-0.379593613	0.002518521	0.013459342
ENSG00000142611	PRDM16	265.1527623	188.6265971	0.491504866	0.002522505	0.013477695
ENSG00000104915	STX10	1469.889877	1877.459469	-0.352809517	0.002526379	0.01349546
ENSG00000174106	LEMD3	2247.304821	1826.021246	0.299304076	0.002537639	0.013552659
ENSG00000235151	AC131097.4	46.79307984	21.72014839	1.107842975	0.002539284	0.013558493
ENSG00000188747	NOXA1	2544.535885	1706.083684	0.577223248	0.002545027	0.013584768
ENSG00000070756	PABPC1	41652.29008	50104.25333	-0.26653212	0.002545311	0.013584768
ENSG00000226608	FTLP3	30.76940072	11.78952306	1.386856753	0.002546688	0.013589162
ENSG00000180071	ANKRD18A	507.4353829	651.7835856	-0.361275946	0.002552234	0.013615796
ENSG00000284308	C2orf81	81.19040104	41.84418243	0.960643688	0.002565593	0.013684089
ENSG00000162676	GFI1	378.7014598	261.6718326	0.530588098	0.002571328	0.013710699
ENSG00000186230	ZNF749	398.2333468	291.3300241	0.449871802	0.002571698	0.013710699
ENSG00000022277	RTF2	2074.668422	1673.512204	0.309756244	0.002572482	0.013711897

ENSG00000135916	ITM2C	4546.090005	5973.572365	-0.393805842	0.002578541	0.013737122
ENSG00000184216	IRAK1	4459.738802	5621.373585	-0.334128615	0.002578827	0.013737122
ENSG00000263089	AC007114.2	53.16354887	25.45023082	1.060421389	0.002578893	0.013737122
ENSG00000211460	TSN	5179.416404	6493.266039	-0.326165179	0.002582781	0.013754852
ENSG00000126088	UROD	2014.322109	1562.386004	0.365937217	0.002597291	0.013829125
ENSG00000266074	BAHCC1	1760.549567	2321.568837	-0.398574634	0.002605107	0.01386773
ENSG00000085733	CTTN	12336.84507	9865.216784	0.322489996	0.002608519	0.013882884
ENSG00000153896	ZNF599	221.7874988	143.2996751	0.633707038	0.002609175	0.01388337
ENSG00000268864	AC011487.2	3.638545507	16.88377785	-2.210531732	0.002611231	0.013891296
ENSG00000203875	SNHG5	573.106772	780.0835923	-0.444449232	0.002618649	0.013924741
ENSG00000108443	RPS6KB1	1505.968361	1927.467749	-0.356352844	0.002618652	0.013924741
ENSG00000120963	ZNF706	1925.483792	1529.240265	0.332093548	0.002622947	0.013944557
ENSG00000166925	TSC22D4	1865.8895	2387.008652	-0.355381203	0.002629768	0.013977797
ENSG00000167117	ANKRD40CL	4.043656918	19.5900501	-2.282264578	0.00265002	0.014082389
ENSG00000164845	FAM86FP	165.6546758	107.7120502	0.623215448	0.00265102	0.014084657
ENSG00000134363	FST	0.367374272	8.421015286	-4.431421013	0.002660004	0.014129331
ENSG00000076770	MBNL3	974.8277748	1251.079103	-0.360158375	0.002665592	0.014153049
ENSG00000151240	DIP2C	1167.227647	1518.600968	-0.379836449	0.002665622	0.014153049
ENSG00000100906	NFKBIA	1154.24237	1452.535544	-0.331629625	0.00266938	0.014169934
ENSG00000105576	TNPO2	3225.67452	3980.113347	-0.303448466	0.002670958	0.014175246
ENSG00000257647	AC124312.2	5.818056367	0	5.123972463	0.002694626	0.014295126
ENSG00000169189	NSMCE1	921.0974902	1195.854013	-0.376958395	0.002694711	0.014295126
ENSG00000205238	SPDYE2	150.9029296	96.66490847	0.643244866	0.002703368	0.014337952
ENSG00000198088	NUP62CL	212.283502	319.0376792	-0.5894202	0.002704237	0.014339467
ENSG00000079785	DDX1	5828.707212	4434.648769	0.394145405	0.002715021	0.014393538
ENSG00000053371	AKR7A2	2260.716889	1794.420336	0.332899805	0.002726782	0.014452771
ENSG00000215009	ACSM4	5.464954426	0	5.033049457	0.002727511	0.014453514
ENSG00000284719	AL033527.5	75.3620336	38.89179185	0.960338013	0.002741548	0.01452476
ENSG00000101955	SRPX	305.609463	206.3206279	0.564455862	0.002746342	0.014547022
ENSG00000182173	TSEN54	1600.818516	2024.992748	-0.338737743	0.002748968	0.014557791
ENSG00000215481	BCRP3	0.373875456	9.699712687	-4.628259609	0.00274996	0.014559904
ENSG00000168875	SOX14	0.373875456	8.708880893	-4.47196107	0.002750836	0.014561402
ENSG00000181315	ZNF322	727.6387519	572.252248	0.346121041	0.002753178	0.014570658
ENSG00000179023	KLHDC7A	340.4799014	239.4228473	0.509524021	0.002754017	0.014571955
ENSG00000247809	NR2F2-AS1	289.9070219	199.9767318	0.538265971	0.002760948	0.014605482
ENSG00000241149	AC115220.1	5.438949691	0	5.027420841	0.002762393	0.014609979
ENSG00000246339	EXTL3-AS1	140.6825692	83.84119037	0.74822941	0.002763264	0.014611437
ENSG00000163528	CHCHD4	408.9436704	574.5143508	-0.491008974	0.002768503	0.014635983
ENSG00000251271	ALG1L7P	31.66175037	11.21671057	1.499192319	0.002769543	0.01463833
ENSG00000188451	SRP72P2	4.050158102	18.29416661	-2.183231741	0.002773421	0.014655674
ENSG00000187231	SESTD1	313.3094337	198.3727738	0.656321928	0.002781956	0.01469761
ENSG00000187109	NAP1L1	13367.33143	15891.641	-0.24957261	0.002788899	0.014731123
ENSG00000272848	AL135910.1	6.917487889	24.27367342	-1.811225844	0.002803851	0.014806915

ENSG00000042832	TG	21.04578499	5.308932396	2.009742793	0.002811922	0.014843392
ENSG00000255624	AC073585.1	191.4122705	129.3606265	0.562887537	0.002811968	0.014843392
ENSG00000144026	ZNF514	1188.848822	915.3903574	0.377888821	0.002814539	0.014853775
ENSG00000151552	QDPR	958.324906	679.2241974	0.495372889	0.002820009	0.014879444
ENSG00000219545	UMAD1	389.5289449	520.1661341	-0.415988713	0.002832675	0.014943059
ENSG00000279833	AL031846.2	84.70512132	135.6436346	-0.677932905	0.002836651	0.014960822
ENSG00000119559	C19orf25	1707.433741	1378.122968	0.309082483	0.002837845	0.014963901
ENSG00000166801	FAM111A	4467.144535	5727.491952	-0.358389396	0.002846671	0.01500722
ENSG00000137171	KLC4	557.2671865	766.2425409	-0.458333946	0.002858147	0.015064484
ENSG00000090432	MUL1	1100.387226	875.6195151	0.329546829	0.002870835	0.015128114
ENSG00000188681	TEKT4P2	62.79852537	32.27666941	0.960847767	0.00287475	0.015145493
ENSG00000099365	STX1B	200.4326876	285.1930005	-0.507602685	0.002875657	0.01514702
ENSG00000142798	HSPG2	6776.986337	9006.55437	-0.41019983	0.002877161	0.015151691
ENSG00000259005	AC005479.1	30.55043508	11.77577674	1.375878864	0.002883649	0.0151826
ENSG00000225697	SLC26A6	1203.863795	972.1503091	0.308586048	0.002888245	0.015203538
ENSG00000205611	LINC01597	2.971500094	16.16019855	-2.463330037	0.002890951	0.015214522
ENSG00000112699	GMDS	882.1788056	1206.790754	-0.452715393	0.002892152	0.01521758
ENSG00000065150	IPO5	10942.34437	14016.9353	-0.357320022	0.002896096	0.015235068
ENSG00000205090	TMEM240	47.87300781	83.74840588	-0.806478572	0.002897775	0.015240633
ENSG00000163995	ABLIM2	304.4813466	212.4157734	0.521960686	0.002903701	0.015268528
ENSG00000168884	TNIP2	1424.382366	1122.273205	0.343389517	0.002905216	0.015273224
ENSG00000261654	AL360270.2	68.9224401	36.61341546	0.913012352	0.002908141	0.015285327
ENSG00000154305	MIA3	3008.321996	3691.760613	-0.295327653	0.002918943	0.015338821
ENSG00000163597	SNHG16	1311.092303	1729.368401	-0.39954903	0.002926034	0.015372791
ENSG00000162894	FCMR	50.92267348	23.71958468	1.092978288	0.00293532	0.015418281
ENSG00000135968	GCC2	3351.735056	4851.30814	-0.533306641	0.00294044	0.015441872
ENSG00000140332	TLE3	1825.288206	1469.091035	0.313163403	0.002943403	0.015454125
ENSG00000139197	PEX5	1562.651547	1199.38693	0.380929453	0.002944082	0.015454384
ENSG00000185885	IFITM1	886.0023642	649.642986	0.448915506	0.002945252	0.015457223
ENSG00000125449	ARMC7	757.8530487	950.9766792	-0.327933472	0.002952471	0.015491796
ENSG00000258890	CEP95	1825.421378	2361.412149	-0.371112544	0.002974998	0.015605653
ENSG00000089486	CDIP1	42.72595811	77.25552825	-0.85136772	0.002975441	0.015605653
ENSG00000250596	AC096751.1	0.367374272	8.291812641	-4.40377259	0.002991815	0.015688179
ENSG00000157483	MYO1E	1763.692467	2254.347781	-0.354446148	0.002995138	0.015699589
ENSG00000101442	ACTR5	713.8689505	519.3999218	0.457448932	0.00299527	0.015699589
ENSG00000235314	LINC00957	7.283592198	23.90719965	-1.711711364	0.003000329	0.015720567
ENSG00000105865	DUS4L	312.0718934	415.7913331	-0.415125837	0.003000553	0.015720567
ENSG00000182389	CACNB4	323.2385249	482.9747333	-0.581257092	0.003008186	0.015757196
ENSG00000260686	AC008669.1	7.60799811	24.53499744	-1.682447615	0.00300963	0.015758175
ENSG00000185352	HS6ST3	7.291363345	27.17004935	-1.895378814	0.003009657	0.015758175
ENSG00000181619	GPR135	262.9271153	183.949775	0.514947701	0.003012566	0.015770044
ENSG00000161618	ALDH16A1	662.234528	877.6288022	-0.407130104	0.00301374	0.015770265
ENSG00000134294	SLC38A2	8644.392234	10806.16397	-0.322046678	0.003013893	0.015770265

ENSG00000197457	STMN3	600.4279305	457.9596426	0.390911439	0.003028752	0.015844637
ENSG00000102962	CCL22	5.384248928	0	5.015317204	0.00303026	0.015849152
ENSG00000007866	TEAD3	1831.618852	1480.196057	0.306982486	0.003032316	0.015856527
ENSG00000137970	RPL7P9	85.89999599	137.6303667	-0.681916592	0.003044841	0.015917131
ENSG00000168916	ZNF608	1046.214471	1340.075463	-0.356751166	0.003045202	0.015917131
ENSG00000111012	CYP27B1	199.9362569	280.8627822	-0.490737552	0.003050189	0.015939803
ENSG00000163354	DCST2	123.5272666	67.01633316	0.888949797	0.003056759	0.015970738
ENSG00000137507	LRRC32	10.55349347	1.240898196	3.115235051	0.003057609	0.015971778
ENSG00000273002	AL355388.2	77.34097499	43.40936438	0.836738391	0.003060916	0.015985651
ENSG00000095596	CYP26A1	3.656779095	16.88377785	-2.207578629	0.003061809	0.015986914
ENSG00000175265	GOLGA8A	228.6043123	312.4812298	-0.450737579	0.003062966	0.015989556
ENSG00000149084	HSD17B12	2051.143948	2531.954892	-0.304097855	0.00306587	0.016001312
ENSG00000178458	H3F3AP6	74.52423334	40.84699215	0.868759098	0.00307378	0.016039185
ENSG00000146247	PHIP	2992.337661	3878.809382	-0.374550327	0.003077997	0.016057778
ENSG00000052126	PLEKHA5	896.1778837	1142.87629	-0.351019484	0.003080045	0.016065048
ENSG00000198205	ZXDA	219.8707154	301.1818846	-0.453443689	0.00308392	0.016081841
ENSG00000136895	GARNL3	569.5798652	440.5580249	0.370724893	0.00308749	0.016097041
ENSG00000188566	NDOR1	1224.235276	956.821667	0.354973325	0.003088944	0.0161012
ENSG00000102119	EMD	1854.093695	2322.724936	-0.325453414	0.003089688	0.016101662
ENSG00000113558	SKP1	4776.194822	3888.581486	0.29647799	0.003091475	0.016107554
ENSG00000147526	TACC1	4493.094145	5924.598703	-0.399187658	0.003095799	0.016122015
ENSG00000118503	TNFAIP3	57.82969892	102.3427906	-0.822245351	0.003096129	0.016122015
ENSG00000143924	EML4	3693.309334	4707.901194	-0.350334792	0.003096278	0.016122015
ENSG00000225173	AL662890.1	37.77551662	14.80135531	1.363296416	0.003097703	0.016122015
ENSG00000004777	ARHGAP33	667.0291149	871.0189837	-0.38412788	0.003098105	0.016122015
ENSG00000220842	RPL21P16	50.20869845	87.42599172	-0.803291879	0.00309819	0.016122015
ENSG00000175087	PDIK1L	488.4318159	676.2047825	-0.47021601	0.003102767	0.016142409
ENSG00000090554	FLT3LG	0.367374272	8.120862717	-4.379016067	0.003107944	0.016164495
ENSG00000169894	MUC3A	24.73633997	52.75642791	-1.091845686	0.003108329	0.016164495
ENSG00000267073	AC005256.1	289.819162	201.0178974	0.526716331	0.003114449	0.016190337
ENSG00000130701	RBBP8NL	322.3220796	535.5419293	-0.731390165	0.003114617	0.016190337
ENSG00000234292	AC123595.1	15.28639065	2.055880942	2.873636494	0.003119273	0.016211107
ENSG00000227533	SLC2A1-AS1	101.1914665	61.58120787	0.717501975	0.003122837	0.016226197
ENSG00000171236	LRG1	255.3857017	346.0620432	-0.438842499	0.003124548	0.016231648
ENSG00000284636	AC073648.6	25.97149171	8.675980812	1.579069388	0.003135582	0.016285523
ENSG00000167964	RAB26	696.3989364	1034.91975	-0.570658582	0.003145613	0.016334168
ENSG00000108582	CPD	6178.225898	7429.178599	-0.265963422	0.003160957	0.016410372
ENSG00000132846	ZBED3	1108.505136	1380.70676	-0.316979669	0.003164638	0.016426011
ENSG00000143228	NUF2	861.9906638	1253.386382	-0.540744647	0.00316784	0.016439156
ENSG00000107960	STN1	559.6022492	416.4069349	0.424623024	0.003174399	0.016469712
ENSG00000284770	TBCE	481.8453864	633.9637919	-0.395495132	0.003182536	0.016508439
ENSG00000179918	SEPHS2	2758.077631	3481.972087	-0.33652945	0.003183349	0.016509169
ENSG00000077080	ACTL6B	30.05288577	11.52965841	1.387452088	0.003186068	0.016519779

ENSG00000266973	AC092296.1	9.340699163	0.609152337	3.953398853	0.00319013	0.016537349
ENSG00000279041	AC102945.2	24.17108752	51.65598997	-1.089444237	0.003197124	0.016570106
ENSG00000104897	SF3A2	2878.655951	3507.384128	-0.285045758	0.003199235	0.016577545
ENSG00000137710	RDX	2795.918224	3685.83392	-0.398849442	0.003206139	0.016609814
ENSG00000231991	ANXA2P2	161.6633423	99.14730421	0.70091779	0.003214272	0.016648435
ENSG00000243660	ZNF487	91.95652155	156.3831012	-0.762307002	0.003217555	0.016661927
ENSG00000171488	LRRC8C	335.7920472	463.1982673	-0.465557811	0.003220505	0.016673687
ENSG00000243449	C4orf48	904.2725034	688.4351517	0.392332015	0.003223043	0.016683308
ENSG00000121749	TBC1D15	1563.458776	2016.039991	-0.367205139	0.003224978	0.016689806
ENSG00000105298	CACTIN	1129.683482	885.9445766	0.350003237	0.003226321	0.016693235
ENSG00000170779	CDCA4	1176.797988	1516.244594	-0.366097923	0.003228009	0.016698259
ENSG00000160679	CHTOP	3084.062206	3758.005459	-0.285217189	0.003228652	0.016698259
ENSG00000204099	NEU4	153.9562425	462.233885	-1.585685954	0.003230487	0.016704228
ENSG00000198945	L3MBTL3	182.4717655	257.3825019	-0.495715148	0.003237001	0.016731034
ENSG00000086300	SNX10	1530.211629	1115.741708	0.454952672	0.003237034	0.016731034
ENSG00000160613	PCSK7	1343.659086	1079.49483	0.315264241	0.003248384	0.016786164
ENSG00000117016	RIMS3	940.636041	582.7690945	0.689585138	0.003249844	0.016790176
ENSG00000223838	AC007091.1	5.475416867	0	5.035484596	0.00325094	0.016792305
ENSG00000255874	LINC00346	19.89927249	50.38907123	-1.341035061	0.003253182	0.016800354
ENSG00000184634	MED12	2486.922444	1892.053771	0.393985266	0.003255491	0.016808739
ENSG00000257093	KIAA1147	2556.89447	3193.079144	-0.320265365	0.003286636	0.01696598
ENSG00000223459	TCAF1P1	189.2321288	283.7599797	-0.58486252	0.003293609	0.016998402
ENSG00000089597	GANAB	26099.08999	31179.70674	-0.256619583	0.003294878	0.017001374
ENSG00000213997	PGAM1P7	23.71111276	7.18011709	1.71677505	0.003299235	0.01701808
ENSG00000023171	GRAMD1B	35.94910771	13.77418367	1.392439926	0.003299502	0.01701808
ENSG00000159788	RGS12	1175.197452	915.2298194	0.361007747	0.003307569	0.017056109
ENSG00000160392	C19orf47	749.3603207	553.7117807	0.435152595	0.003314882	0.017086647
ENSG00000115685	PPP1R7	1725.495197	1386.151203	0.315810798	0.003314883	0.017086647
ENSG00000152763	WDR78	123.8291525	191.2698186	-0.624596869	0.003324545	0.017132849
ENSG00000105245	NUMBL	942.5469877	679.253645	0.471689438	0.003325462	0.017133981
ENSG00000234171	RNASEH1-AS1	278.0230849	180.5284973	0.619598747	0.00332754	0.017141088
ENSG00000163638	ADAMTS9	0	6.190744656	-4.94326349	0.00332925	0.017146299
ENSG00000253764	AC019257.1	48.12828926	20.4792502	1.232977037	0.003333965	0.017166985
ENSG00000232530	LIF-AS1	12.67957353	1.719388527	2.841222372	0.003335695	0.01717229
ENSG00000166535	A2ML1	12.34866643	2.150203102	2.538122569	0.003336824	0.017174501
ENSG00000162231	NXF1	1908.171311	2533.076996	-0.40840857	0.003337591	0.017174845
ENSG00000117480	FAAH	760.990235	1077.256794	-0.500618677	0.003340775	0.017187629
ENSG00000181458	TMEM45A	126.6482827	69.61943604	0.86817938	0.003342603	0.017193434
ENSG00000115504	EHBP1	1683.486222	1302.838707	0.369256114	0.003343402	0.01719394
ENSG00000126243	LRFN3	669.1872019	513.3946551	0.381158936	0.003350665	0.017227679
ENSG00000132478	UNK	1462.62437	1806.643411	-0.304868395	0.003355387	0.017248345
ENSG00000160932	LY6E	2834.930249	3793.658287	-0.420377744	0.003368864	0.017313999
ENSG00000249673	NOP14-AS1	742.4179466	579.5922525	0.357712379	0.00337688	0.017351567

ENSG00000273314	AC005229.4	149.7966829	95.76007268	0.641803373	0.003388322	0.017406716
ENSG00000280287	AC131212.3	276.9604345	176.8942349	0.648919322	0.003395338	0.017439108
ENSG00000188933	USP32P1	56.89580224	104.1917335	-0.867801182	0.003419209	0.017558044
ENSG00000237289	CKMT1B	1.083889228	11.11404952	-3.346794806	0.003433862	0.017627332
ENSG00000263624	AC055811.1	52.38043816	26.32955437	0.993722751	0.003434138	0.017627332
ENSG00000153823	PID1	8.698388522	26.06230188	-1.576545779	0.003438527	0.017646171
ENSG00000241954	AL021937.4	20.54300446	5.392947998	1.927328018	0.003453029	0.017716887
ENSG00000125850	OVOL2	274.8058098	368.8021193	-0.424284979	0.003455235	0.017724505
ENSG00000171757	LRRC34	212.7003346	290.3537331	-0.449184892	0.003456461	0.017727089
ENSG00000267395	DM1-AS	48.04123394	93.62439762	-0.957704525	0.003467033	0.017777596
ENSG00000123415	SMUG1	1298.326123	1053.090212	0.302194301	0.003472041	0.017796948
ENSG00000143149	ALDH9A1	2878.806244	3545.477669	-0.300581978	0.003472257	0.017796948
ENSG00000130812	ANGPTL6	38.84767345	17.23160618	1.174572831	0.00348516	0.017859353
ENSG00000181392	SYNE4	124.6122632	191.1646816	-0.614651208	0.003486726	0.01786365
ENSG00000279393	AL139005.1	0	6.761068435	-5.06531544	0.003490311	0.017878286
ENSG00000114353	GNAI2	4191.120374	3360.949701	0.318309093	0.003494149	0.017894214
ENSG00000107736	CDH23	34.5133865	14.53513218	1.250301987	0.003494984	0.017894754
ENSG00000008300	CELSR3	1127.961709	895.2788834	0.33334004	0.003509093	0.017963247
ENSG00000087266	SH3BP2	3594.420971	4595.829433	-0.354418779	0.003516096	0.017995348
ENSG00000004700	RECQL	1427.288437	953.4289781	0.581323193	0.003530458	0.018065086
ENSG00000158321	AUTS2	213.0393268	149.9396063	0.506459251	0.003532135	0.018066746
ENSG00000132518	GUCY2D	0	5.773676404	-4.845998149	0.003532255	0.018066746
ENSG00000143839	REN	16.21917995	3.668660346	2.157331355	0.003535835	0.018077894
ENSG00000197653	DNAH10	62.1038913	31.15612666	0.990271452	0.003535907	0.018077894
ENSG00000124209	RAB22A	1514.27337	1238.878063	0.289535213	0.003547023	0.018130951
ENSG00000134278	SPIRE1	1638.863461	2056.064286	-0.327471349	0.003555295	0.018169453
ENSG00000111110	PPM1H	1498.426641	1848.41331	-0.302553639	0.003559533	0.018187324
ENSG00000140090	SLC24A4	12.12970079	1.549897964	2.98967007	0.003564432	0.018208566
ENSG00000213366	GSTM2	303.5425356	193.0710985	0.654950113	0.00357121	0.018239395
ENSG00000143575	HAX1	2739.801689	3398.902567	-0.311011902	0.00357888	0.018274766
ENSG00000147650	LRP12	1191.281425	926.6336363	0.361758695	0.003580107	0.018277233
ENSG00000096746	HNRNPH3	4736.764991	5906.735274	-0.318562841	0.003583677	0.018291651
ENSG00000219607	PPP1R3G	257.8469753	162.8799142	0.666954878	0.003587723	0.018308499
ENSG00000125743	SNRPD2	3574.624677	4567.966873	-0.353913137	0.003588818	0.018310281
ENSG00000158019	BABAM2	808.3703192	636.0636482	0.345693909	0.003593558	0.018330654
ENSG00000063046	EIF4B	20947.1187	26469.32737	-0.337535052	0.003595894	0.018338761
ENSG00000264370	MIR3125	5.096310191	0	4.932529844	0.003602142	0.018366808
ENSG00000226314	ZNF192P1	55.98251649	27.85145137	1.003174428	0.00361632	0.018431585
ENSG00000205268	PDE7A	1181.304754	1495.802897	-0.340917216	0.003616347	0.018431585
ENSG00000210174	MT-TR	11.22831003	29.64797597	-1.397704183	0.003629124	0.018492865
ENSG00000176018	LYSMD3	1002.763422	1231.408895	-0.296305169	0.003636456	0.018526383
ENSG00000100739	BDKRB1	15.96770839	39.41109114	-1.305753782	0.00364075	0.018543952
ENSG00000204923	FBXO48	169.6418855	97.93147552	0.790366047	0.003641415	0.018543952

ENSG00000274721	RF02039	18.88704765	5.169423192	1.874930335	0.003644931	0.018554383
ENSG00000285230	RALY-AS1	267.7305695	194.2268349	0.463092877	0.003644975	0.018554383
ENSG00000248846	LINC02065	12.23149376	1.841203334	2.749104851	0.003649062	0.018571333
ENSG00000158887	MPZ	9.05403039	0.600305139	3.90927237	0.003651826	0.018581549
ENSG00000120217	CD274	30.10108535	57.66401879	-0.938367613	0.0036541	0.018589269
ENSG00000114383	TUSC2	1100.487911	813.6743219	0.434664941	0.003663481	0.018633129
ENSG00000165476	REEP3	2137.417178	2589.114635	-0.276702897	0.003668977	0.018657221
ENSG00000183691	NOG	5.059843015	0	4.924017733	0.003671631	0.018666849
ENSG00000181896	ZNF101	885.1460417	663.0908011	0.415669139	0.003676203	0.018686225
ENSG00000135454	B4GALNT1	29.21634425	10.40421654	1.479511309	0.003683494	0.018719408
ENSG00000003756	RBM5	2911.523636	3789.634357	-0.380014149	0.003684824	0.018722294
ENSG00000143499	SMYD2	1527.496349	1237.721128	0.30329926	0.00368677	0.018728305
ENSG00000099785	2-Mar	517.7125157	388.3215612	0.414498581	0.003694685	0.018764628
ENSG00000144674	GOLGA4	4089.853447	4942.073689	-0.273104203	0.003714376	0.018858383
ENSG00000280069	AC127024.8	74.80963215	42.66809067	0.808322351	0.003714681	0.018858383
ENSG00000197746	PSAP	23551.17509	18311.78315	0.363079261	0.003719293	0.018877893
ENSG00000173327	MAP3K11	2943.997793	2329.333048	0.337609065	0.003722014	0.018885798
ENSG00000101166	PRELID3B	2918.430193	3816.676804	-0.387374084	0.003722389	0.018885798
ENSG00000197558	SSPO	1443.826087	544.6175192	1.406976269	0.003725318	0.018896755
ENSG00000257052	AP003721.4	20.0151752	5.733388486	1.803537007	0.00373821	0.018958232
ENSG00000263893	AC080037.1	0.72301614	9.724794922	-3.742317282	0.00374581	0.018990016
ENSG00000214530	STARD10	1548.365764	1933.20077	-0.320228021	0.003746907	0.018990016
ENSG00000143156	NME7	415.4926542	312.8300874	0.408723355	0.003747328	0.018990016
ENSG00000160007	ARHGAP35	8021.343323	9626.854351	-0.263235552	0.003747571	0.018990016
ENSG00000226416	MRPL23-AS1	4.704201147	18.81930335	-1.990920074	0.003751764	0.019007336
ENSG00000167880	EVPL	2503.840794	3076.726149	-0.297477646	0.003757321	0.01903029
ENSG00000175221	MED16	2705.25151	2235.724913	0.274976117	0.003757845	0.01903029
ENSG00000224877	NDUFAF8	981.1067366	709.4832278	0.466407809	0.003761267	0.019043693
ENSG00000185630	PBX1	586.7879566	822.7859005	-0.486678232	0.003769178	0.01907981
ENSG00000167103	PIP5KL1	1376.612126	1952.260846	-0.503758913	0.003772028	0.019090302
ENSG00000170222	ADPRM	136.4278579	200.4809037	-0.55606959	0.003777177	0.019112422
ENSG00000075218	GTSE1	1660.897555	2377.85911	-0.518002497	0.003781715	0.019131443
ENSG00000224273	AC005077.2	9.083996382	0.663186579	3.877802003	0.003785174	0.019144993
ENSG00000186076	AC012085.1	50.03697638	23.61787468	1.079294641	0.003788343	0.019157076
ENSG00000163872	YEATS2	3111.215988	2524.175044	0.301407751	0.00379106	0.019166867
ENSG00000148926	ADM	158.7579615	107.2778741	0.566373261	0.003805691	0.01923688
ENSG00000130244	FAM98C	666.7449524	834.0337125	-0.322818786	0.003813936	0.019274586
ENSG00000104205	SGK3	361.8101546	475.41837	-0.395206268	0.003816949	0.019283734
ENSG00000078114	NEBL	4295.223608	3162.026406	0.442145131	0.003817317	0.019283734
ENSG00000072415	MPP5	2274.8267	2925.14939	-0.362806934	0.003821684	0.019301824
ENSG00000236675	MTX1P1	94.36641651	143.746803	-0.605055134	0.003825788	0.019318577
ENSG00000092199	HNRNPC	19186.55809	24923.40817	-0.37743909	0.003827843	0.019322849
ENSG00000261471	AC092145.1	11.86411949	1.818609813	2.713358352	0.003828208	0.019322849

ENSG00000185340	GAS2L1	1413.811292	1115.98922	0.340847881	0.003835069	0.0193535
ENSG00000134759	ELP2	2222.621429	2678.985445	-0.269280187	0.0038377	0.019359596
ENSG00000043591	ADRB1	23.45980379	51.84712301	-1.139184718	0.003837854	0.019359596
ENSG00000065621	GSTO2	1523.533212	1895.062995	-0.314616056	0.003839864	0.019365757
ENSG00000100129	EIF3L	5410.286289	6783.85354	-0.326285413	0.003840903	0.019367019
ENSG00000134146	DPH6	230.9569666	320.394451	-0.473504958	0.003844563	0.019381491
ENSG00000160886	LY6K	15.74874275	3.073254332	2.368173975	0.00384618	0.019385663
ENSG00000029534	ANK1	21.32199132	6.410321388	1.743540934	0.003847943	0.019390568
ENSG00000280233	AC015813.7	22.27158167	6.687880436	1.739016478	0.003859254	0.019443577
ENSG00000234650	PCCA-AS1	5.831058735	20.25082627	-1.798038763	0.003861383	0.019443586
ENSG00000235343	LINC01665	5.13404733	0	4.941330167	0.003861612	0.019443586
ENSG00000105204	DYRK1B	243.9301896	164.8687049	0.563588872	0.003861632	0.019443586
ENSG00000063245	EPN1	4273.001066	3387.172633	0.334938717	0.00388112	0.019537701
ENSG00000188486	H2AFX	4157.804817	5457.203025	-0.392467639	0.003896083	0.019609008
ENSG00000081386	ZNF510	670.905782	524.251941	0.356710752	0.003899454	0.019621953
ENSG00000113580	NR3C1	417.0568152	313.4155259	0.413347176	0.003902997	0.019635756
ENSG00000155111	CDK19	1556.551596	1969.120382	-0.338688586	0.003904414	0.019638859
ENSG00000102606	ARHGEF7	2603.906381	2073.18639	0.32842352	0.003915699	0.019691589
ENSG00000154222	CC2D1B	1918.772864	1565.249492	0.293392259	0.003917139	0.019694792
ENSG00000114439	BBX	4574.221407	3804.250809	0.265979852	0.003918786	0.019699038
ENSG00000234664	HMGN2P5	209.6715938	286.3378437	-0.450805476	0.00392257	0.019714028
ENSG00000160062	ZBTB8A	270.7789652	364.8288373	-0.429366234	0.00393234	0.019759085
ENSG00000248429	FAM198B-AS1	11.40018347	1.487016523	2.922807617	0.003935666	0.019771746
ENSG00000221883	ARIH2OS	51.39961186	87.87098263	-0.770255069	0.003940154	0.019790248
ENSG00000198482	ZNF808	307.6675147	215.7975065	0.514519815	0.003945556	0.019813327
ENSG00000023839	ABCC2	48.06327742	22.36812929	1.099409101	0.00395891	0.019876321
ENSG00000115687	PASK	1828.856974	1425.064859	0.359317554	0.003972018	0.019938053
ENSG00000102974	CTCF	2370.117445	2909.80176	-0.296132932	0.003974833	0.019948105
ENSG00000132024	CC2D1A	3298.202061	2729.791285	0.272785493	0.003976435	0.01995165
ENSG00000107077	KDM4C	1616.156934	2001.505588	-0.308250181	0.003977165	0.01995165
ENSG00000166181	API5	3721.05728	4958.304747	-0.414314694	0.003978149	0.019952511
ENSG00000156876	SASS6	468.7210777	687.7548188	-0.554397739	0.00399181	0.020016939
ENSG00000115556	PLCD4	223.2095897	155.9543453	0.519152022	0.003998422	0.020045997
ENSG00000176761	ZNF285B	24.29349142	8.036847115	1.611976087	0.004019017	0.02014514
ENSG00000173786	CNP	2708.88224	3384.123197	-0.321328984	0.004022199	0.020156973
ENSG00000096092	TMEM14A	945.6615168	755.3374497	0.324246429	0.004023303	0.020158389
ENSG00000120742	SERP1	4150.942178	5309.627486	-0.35516826	0.00402683	0.020171945
ENSG00000073169	SELENOO	1152.519363	907.4105014	0.34521778	0.004035536	0.020207552
ENSG00000135049	AGTPBP1	2301.262268	1795.900956	0.357221402	0.004035584	0.020207552
ENSG00000135164	DMTF1	2192.127282	2779.224677	-0.34202709	0.00404231	0.020237104
ENSG00000131269	ABCB7	1029.748572	1259.794565	-0.290948496	0.004050531	0.020274125
ENSG00000128311	TST	1148.721316	1431.422695	-0.317651191	0.004055751	0.020295692
ENSG00000101856	PGRMC1	3905.794331	3220.522218	0.278112957	0.004056493	0.020295692

ENSG00000102098	SCML2	285.1309938	388.6262484	-0.44810535	0.004058215	0.020300169
ENSG00000204516	MICB	719.2365303	472.7343666	0.604113552	0.004059651	0.020303217
ENSG00000105991	HOXA1	57.81288667	29.18426132	0.979699806	0.004061193	0.020306791
ENSG00000184988	TMEM106A	56.1493213	94.36883768	-0.749753275	0.004069368	0.020343524
ENSG00000127334	DYRK2	1380.393841	1692.269397	-0.293870849	0.004076772	0.020376388
ENSG00000158480	SPATA2	466.3821939	336.3788788	0.469634457	0.004079723	0.020384113
ENSG00000275216	AL161431.1	3647.817637	1772.499833	1.041078417	0.004080562	0.020384113
ENSG00000145740	SLC30A5	1794.061881	2243.130665	-0.322451888	0.004080808	0.020384113
ENSG00000112394	SLC16A10	324.1353236	428.4440229	-0.40328719	0.0040837	0.02039441
ENSG00000183340	JRKL	833.0587399	659.9122534	0.336782858	0.004093508	0.020439236
ENSG00000142599	RERE	3604.854141	2994.985519	0.267211306	0.004118457	0.020559627
ENSG00000119632	IFI27L2	365.4721508	259.2085123	0.495556369	0.004121137	0.02056882
ENSG00000104760	FGL1	0.367374272	7.735235185	-4.311557014	0.004130045	0.020609094
ENSG00000203985	LDLRAD1	49.94216113	111.6714293	-1.157347591	0.004142588	0.020665172
ENSG00000109689	STIM2	1003.551159	1264.168653	-0.332937816	0.004142967	0.020665172
ENSG00000188690	UROS	1750.540718	1438.491943	0.283120388	0.004154963	0.0207208
ENSG00000145860	RNF145	4078.576576	3390.482675	0.266746885	0.004170084	0.020789705
ENSG00000167377	ZNF23	94.72174442	55.77169993	0.765506566	0.004170473	0.020789705
ENSG00000250215	CIR1P2	12.46837903	1.809762614	2.781227684	0.004175262	0.020809034
ENSG00000177425	PAWR	3484.880445	2739.050853	0.347196823	0.004176046	0.020809034
ENSG00000043039	BARX2	151.1383823	242.3956941	-0.684179043	0.004180873	0.020828856
ENSG00000181085	MAPK15	426.9995479	289.2672365	0.563537256	0.004182029	0.020830389
ENSG00000204843	DCTN1	5114.469538	4139.224053	0.305025353	0.004187535	0.020853586
ENSG00000071205	ARHGAP10	568.7709236	739.6634552	-0.379709198	0.004192551	0.02087433
ENSG00000278996	FP671120.1	5.382978964	247.8817252	-5.52392978	0.0042048	0.020931073
ENSG00000198863	RUNDC1	934.110979	719.593246	0.375403196	0.004209187	0.020948663
ENSG00000197744	PTMAP2	80.55856406	136.4298347	-0.762656289	0.004215003	0.020971716
ENSG00000076864	RAP1GAP	353.9086697	259.1805113	0.44892565	0.004215528	0.020971716
ENSG00000198805	PNP	1097.409073	1446.951605	-0.399486916	0.004222816	0.021003721
ENSG00000235174	RPL39P3	59.08831547	96.28563943	-0.703064426	0.004228331	0.021026889
ENSG00000162419	GMEB1	677.0983529	885.486426	-0.387647658	0.004230852	0.021035166
ENSG00000146963	LUC7L2	1997.088016	2425.349455	-0.280312735	0.004254846	0.021150176
ENSG00000167550	RHEBL1	138.0197701	205.9860376	-0.580321143	0.004256306	0.021153152
ENSG00000133115	STOML3	12.79024502	2.42776215	2.400910635	0.004262715	0.021180717
ENSG00000186908	ZDHHC17	1142.279976	1428.416325	-0.322182064	0.004263775	0.021181696
ENSG00000241593	AC099542.1	13.52626353	2.759355439	2.304951451	0.004274128	0.021228834
ENSG00000125149	C16orf70	957.3007637	1265.498154	-0.403422569	0.004289702	0.021301876
ENSG00000160563	MED27	773.0646295	540.3617797	0.515210918	0.004298424	0.021340871
ENSG00000140307	GTF2A2	1163.028769	901.5109979	0.366478155	0.00430848	0.021386472
ENSG00000237940	LINC01238	169.9418818	113.7869085	0.579068272	0.004333216	0.021504911
ENSG00000197343	ZNF655	970.3261587	1296.782101	-0.418015577	0.004342337	0.021545819
ENSG00000177951	BET1L	1078.10912	1359.317201	-0.334598825	0.004344955	0.021554453
ENSG00000101752	MIB1	2465.508918	3066.729816	-0.315105415	0.004358293	0.021616251

ENSG00000261420	AL022069.1	10.6094642	28.57262027	-1.435019734	0.004365476	0.021647506
ENSG00000185624	P4HB	20268.25266	24993.31175	-0.302317393	0.004374692	0.021688824
ENSG00000103061	SLC7A6OS	375.2848964	278.3128408	0.430494313	0.004377785	0.021699776
ENSG00000198105	ZNF248	851.3296554	670.7442748	0.344989901	0.004394209	0.021776791
ENSG00000184786	TCTE3	79.62846605	123.6428737	-0.633747263	0.00439888	0.021791271
ENSG00000154451	GBP5	0.72301614	9.815169009	-3.753953443	0.004398906	0.021791271
ENSG00000086730	LAT2	433.29819	576.2800455	-0.411046874	0.004402489	0.02180462
ENSG00000175283	DOLK	597.1718026	461.7233028	0.369774487	0.004404299	0.021809188
ENSG00000198324	PHETA1	387.9527263	293.9134522	0.400349991	0.004407666	0.021821458
ENSG00000180448	ARHGAP45	2052.72235	2621.857467	-0.35265232	0.004422167	0.021888836
ENSG00000121350	PYROXD1	534.478238	676.8476679	-0.34013172	0.004428032	0.021913388
ENSG00000072818	ACAP1	276.7807787	192.4861556	0.525937492	0.004428913	0.021913388
ENSG00000197959	DNM3	50.76507237	23.69347309	1.109237078	0.004437274	0.021949596
ENSG00000136450	SRSF1	2377.732617	2949.727549	-0.311204137	0.004438019	0.021949596
ENSG00000278129	ZNF8	925.8289997	692.7309197	0.419707234	0.004439019	0.021950122
ENSG00000205060	SLC35B4	916.9233584	676.0157865	0.438708534	0.00444123	0.021956631
ENSG00000154493	C10orf90	6.896714375	0.277559048	4.407954497	0.004445131	0.021971496
ENSG00000081189	MEF2C	169.5029833	115.3221091	0.555657376	0.004453344	0.02200766
ENSG00000151849	CENPJ	1270.606772	924.4101411	0.458091792	0.004456613	0.022019379
ENSG00000119227	PIGZ	150.8791396	241.3349578	-0.675951948	0.004459705	0.022030223
ENSG00000155330	C16orf87	480.4491489	635.902888	-0.405292526	0.004460691	0.022030661
ENSG00000260966	AP001486.2	43.42011564	81.23705813	-0.902804531	0.004471635	0.022080272
ENSG00000036549	AC118549.1	1853.263979	2226.0455	-0.264361925	0.004478587	0.022110152
ENSG00000008438	PGLYRP1	64.94141768	34.99814734	0.8967124	0.004482453	0.022124787
ENSG00000248610	HSPA8P4	5.425947324	0	5.024430631	0.004484455	0.022130224
ENSG00000131148	EMC8	1333.442846	1020.963684	0.384392452	0.004494724	0.022173722
ENSG00000105202	FBL	7067.4256	5317.797507	0.410153602	0.004495076	0.022173722
ENSG00000188599	NPIPP1	77.24965568	130.3247601	-0.75303276	0.004497973	0.022183557
ENSG00000161542	PRPSAP1	1412.480683	1741.535072	-0.302103453	0.004502374	0.022199983
ENSG00000178425	NT5DC1	969.1370225	1242.245437	-0.357617369	0.004503113	0.022199983
ENSG00000054690	PLEKHH1	1182.031243	953.5972817	0.309991368	0.004504074	0.022200267
ENSG00000148335	NTMT1	1226.868541	931.8458743	0.395849772	0.004511273	0.022231285
ENSG00000053900	ANAPC4	877.5182374	1109.381524	-0.338311817	0.004514867	0.022244533
ENSG00000108179	PPIF	3451.39665	2586.486906	0.415806503	0.004517136	0.022251243
ENSG00000006704	GTF2IRD1	2482.223578	3034.438786	-0.289633864	0.004523711	0.022279161
ENSG00000119927	GPAM	1207.445904	959.3017051	0.331294409	0.004530135	0.022306325
ENSG00000258701	LINC00638	78.24632701	151.2312814	-0.948050977	0.004534137	0.022321556
ENSG00000282842	FRG2EP	5.132777367	0	4.941227503	0.004546142	0.022376169
ENSG00000119630	PGF	3.68278383	18.17338116	-2.318524713	0.004555046	0.022415501
ENSG00000167995	BEST1	56.02278234	116.2651085	-1.053778082	0.004558706	0.022429017
ENSG00000165671	NSD1	6441.758619	7624.019602	-0.243037688	0.004563463	0.022447922
ENSG00000101474	APMAP	3952.390314	3200.641603	0.304265755	0.004573717	0.022493854
ENSG00000187492	CDHR4	23.80498322	7.735235185	1.615335564	0.004578896	0.022514816

ENSG00000197756	RPL37A	15460.00666	18842.66319	-0.285473477	0.004587816	0.02255416
ENSG00000136943	CTSV	421.6351277	598.9606685	-0.507061487	0.00459496	0.02258476
ENSG00000197780	TAF13	713.3890069	515.5647806	0.466939276	0.004597542	0.022592926
ENSG00000013288	MAN2B2	1868.010037	1529.715512	0.288622986	0.004608974	0.022644572
ENSG00000151882	CCL28	429.7049902	321.784839	0.418768186	0.004614233	0.022665874
ENSG00000198840	MT-ND3	39236.01437	30887.0892	0.345149047	0.00461691	0.022674485
ENSG00000279838	AL356273.3	7.938905207	23.44838412	-1.558821167	0.004621036	0.022686391
ENSG00000260105	AOC4P	13.34011776	2.055880942	2.678432191	0.004621812	0.022686391
ENSG00000260853	AC109460.2	86.36741673	142.9602512	-0.725088393	0.004622106	0.022686391
ENSG00000114446	IFT57	730.2282914	955.9077889	-0.389474855	0.00462411	0.022691688
ENSG00000119125	GDA	1416.21327	1073.750459	0.399463004	0.004631249	0.022722182
ENSG00000177469	CAVIN1	5725.948184	4416.172424	0.374522242	0.004632966	0.022726063
ENSG00000108433	GOSR2	1248.662228	999.0459915	0.322305017	0.004634628	0.022729677
ENSG00000181222	POLR2A	15689.00956	12214.81764	0.361046551	0.004642159	0.022758643
ENSG00000271046	AL512631.1	41.42595712	17.75133548	1.223818404	0.004642389	0.022758643
ENSG00000189403	HMGB1	13792.18657	18924.0227	-0.45641689	0.004643555	0.022759818
ENSG00000237222	LINC01968	17.74068651	4.837829903	1.877250582	0.004649847	0.022786108
ENSG00000163053	SLC16A14	909.8433746	1299.446603	-0.514785466	0.004660297	0.022832757
ENSG00000143061	IGSF3	2418.410778	1970.149116	0.295563167	0.00466175	0.022835322
ENSG00000186376	ZNF75D	463.1676325	618.6480893	-0.416141813	0.004662858	0.022836192
ENSG00000274956	NKAIN3-IT1	5.083307823	0	4.92952765	0.004665607	0.022845095
ENSG00000020633	RUNX3	0.373875456	7.443929815	-4.255902485	0.004667605	0.022850323
ENSG00000066027	PPP2R5A	1985.606527	2433.33055	-0.293056444	0.004671778	0.022866195
ENSG00000178764	ZHX2	786.598779	1000.269377	-0.346518409	0.004679891	0.022898407
ENSG00000198728	LDB1	2890.479418	2357.655484	0.294026222	0.004680225	0.022898407
ENSG00000005483	KMT2E	2435.668496	2941.047467	-0.271976385	0.004698505	0.022983262
ENSG00000015479	MATR3	485.5169032	377.2894558	0.364002986	0.004705304	0.023011936
ENSG00000010310	GIPR	400.5424019	576.9222676	-0.525148943	0.004709403	0.023027396
ENSG00000156521	TYSND1	1735.233052	2115.132412	-0.285630366	0.004712117	0.023036078
ENSG00000255819	KLRC4-KLRK1	26.267039	8.968745543	1.563577528	0.00471548	0.023047928
ENSG00000183696	UPP1	496.5474864	360.9732651	0.457961944	0.004721308	0.023068507
ENSG00000279141	LINC01451	11.22307881	1.478169325	2.907118645	0.004721569	0.023068507
ENSG00000142556	ZNF614	23.31758239	7.127542209	1.72524791	0.004726885	0.023089882
ENSG00000123091	RNF11	2847.81566	2339.026925	0.283686092	0.004729687	0.023098976
ENSG00000264281	AC016596.1	85.74096234	141.4869154	-0.720452413	0.004731521	0.023100437
ENSG00000253320	AZIN1-AS1	108.7381001	63.91413955	0.764215306	0.004731868	0.023100437
ENSG00000221887	HMSD	45.07719104	80.31855432	-0.835601616	0.004734218	0.023102546
ENSG00000254760	AC008750.1	16.26341827	3.797862991	2.076646153	0.004734877	0.023102546
ENSG00000018189	RUFY3	721.5237269	905.072867	-0.327365797	0.004735123	0.023102546
ENSG00000213693	SEC14L1P1	30.57786115	61.11448346	-0.996600454	0.004736569	0.023105008
ENSG00000165801	ARHGEF40	1346.939118	1024.517474	0.394027098	0.004746621	0.023149442
ENSG00000100296	THOC5	1166.667312	1519.35949	-0.381488547	0.004747786	0.023150524
ENSG00000261026	AC105046.1	10.15092199	1.232050997	3.061592009	0.004749382	0.023153707

ENSG00000198890	PRMT6	756.8351356	994.8610556	-0.394750705	0.004755987	0.023181305
ENSG00000041880	PARP3	769.1301862	591.182147	0.380594819	0.004770538	0.023241395
ENSG00000117115	PADI2	35.83320499	66.26629056	-0.883936582	0.004771437	0.023241395
ENSG00000170471	RALGAPB	4294.735666	3596.73211	0.255774114	0.004771806	0.023241395
ENSG00000132196	HSD17B7	794.595253	623.8425944	0.34815816	0.004772514	0.023241395
ENSG00000047365	ARAP2	890.34828	692.7684325	0.361756725	0.004773049	0.023241395
ENSG00000117791	2-Mar	499.559101	640.6980326	-0.358668551	0.004777002	0.023256033
ENSG00000187514	PTMA	45539.60848	60173.62375	-0.402027584	0.004783097	0.023281087
ENSG00000214717	ZBED1	1204.446967	1472.557984	-0.290216504	0.004789252	0.023306429
ENSG00000235381	AL596202.1	15.68230958	36.62862114	-1.222542211	0.004797516	0.023342018
ENSG00000066735	KIF26A	1077.778864	1328.0276	-0.300967025	0.004818716	0.023440517
ENSG00000248360	LINC00504	9.758812942	0.900457708	3.433194871	0.004832924	0.023504978
ENSG00000166793	YPEL4	3.643776728	16.52813168	-2.184784483	0.004845453	0.023561242
ENSG00000186998	EMID1	296.0093697	403.0425886	-0.443425449	0.004847381	0.023565948
ENSG00000276728	AC142472.1	52.42086659	90.24719925	-0.78453073	0.004860104	0.023623129
ENSG00000183145	RIPPLY3	1.464265867	11.14257152	-2.942794671	0.004862777	0.023631442
ENSG00000033050	ABCF2	1021.387894	771.0377375	0.404523678	0.004871223	0.023667801
ENSG00000149260	CAPN5	355.8533586	464.4708796	-0.384717146	0.004873418	0.023673692
ENSG00000207561	MIR635	62.33348197	32.57382495	0.936895342	0.004874364	0.023673692
ENSG00000143001	TMEM61	17.39550708	41.50425025	-1.248301943	0.004885073	0.023719953
ENSG00000222112	RN7SKP16	22.95543934	6.68152195	1.75731512	0.004885822	0.023719953
ENSG00000173960	UBXN2A	1102.92731	1405.331612	-0.350184731	0.004891779	0.023740712
ENSG00000154122	ANKH	1816.227475	1493.732735	0.281831511	0.004892031	0.023740712
ENSG00000227403	LINC01806	320.083419	450.6003408	-0.49243874	0.004893256	0.023741962
ENSG00000227017	AC007036.1	0.367374272	7.727847347	-4.301166444	0.004901231	0.023775959
ENSG00000185721	DRG1	1657.944728	2052.647008	-0.308396958	0.004916516	0.023845393
ENSG00000102931	ARL2BP	199.681138	136.7952008	0.543718661	0.004923036	0.0238723
ENSG00000136100	VPS36	1937.591766	2333.23967	-0.268100157	0.004930225	0.02390244
ENSG00000164938	TP53INP1	150.5548963	98.46720495	0.61580446	0.004937423	0.023932612
ENSG00000151229	SLC2A13	419.1830466	312.8645379	0.420188372	0.004943631	0.023953466
ENSG00000146729	NIPSNAP2	1779.897688	2151.965771	-0.273964788	0.004943677	0.023953466
ENSG00000234771	SLC25A25-AS1	400.8303435	615.8914816	-0.619174684	0.004956168	0.024009252
ENSG00000172071	EIF2AK3	737.1958655	937.9953533	-0.347297208	0.004979819	0.024119065
ENSG00000164741	DLC1	616.4332022	907.1004221	-0.558169178	0.004995808	0.024191733
ENSG00000180385	EMC3-AS1	509.8985573	394.6010512	0.370011247	0.005010747	0.02425929
ENSG00000138385	SSB	3387.609536	4646.533713	-0.456100065	0.005012911	0.024264981
ENSG00000112667	DNPH1	2041.715498	2670.302164	-0.387415946	0.005020593	0.024297374
ENSG00000103479	RBL2	2783.887078	3378.624956	-0.279081051	0.005045157	0.024411439
ENSG00000171517	LPAR3	14.22977612	3.01922009	2.235521835	0.00505685	0.024463196
ENSG00000042445	RETSAT	1986.304438	1637.66301	0.278691464	0.005059038	0.02446896
ENSG00000128908	INO80	1689.944064	2200.643141	-0.381378875	0.005064195	0.024489078
ENSG00000157111	TMEM171	13.61109287	37.30417298	-1.449453407	0.005073181	0.0245277
ENSG00000087250	MT3	0	5.384100799	-4.749145078	0.005076597	0.024539382

ENSG00000113924	HGD	16.23218232	37.66172126	-1.210414205	0.005083862	0.024569664
ENSG00000139329	LUM	4.728935919	0	4.824763165	0.005094368	0.024615595
ENSG00000136603	SKIL	509.4645985	716.7110725	-0.491607102	0.005097266	0.024624748
ENSG00000124275	MTRR	699.0580545	892.8865499	-0.353825071	0.00510059	0.02463596
ENSG00000271811	Z97200.1	6.567077242	0.331593289	4.3357846	0.005102919	0.024642362
ENSG00000186480	INSIG1	11466.94841	8711.314382	0.396424115	0.005113977	0.024690906
ENSG00000165732	DDX21	8487.681692	11677.47715	-0.460376331	0.005116471	0.02469809
ENSG00000156531	PHF6	1659.895162	2141.458958	-0.367893146	0.005118209	0.024701622
ENSG00000197381	ADARB1	729.4601034	913.3780873	-0.323800285	0.005138094	0.024792716
ENSG00000220749	RPL21P28	45.82255339	84.57970196	-0.887732743	0.00514589	0.024825458
ENSG00000138107	ACTR1A	3619.439612	2776.507071	0.382162464	0.005149285	0.024836953
ENSG00000064666	CNN2	10259.33803	8454.497988	0.27906641	0.005153017	0.024849461
ENSG00000175550	DRAP1	2353.613805	1583.828346	0.570940515	0.005153902	0.024849461
ENSG00000242078	AC084864.1	33.77086681	14.83528475	1.189474691	0.005158985	0.024869086
ENSG00000261253	AC137932.2	24.37942814	8.33210056	1.562898098	0.005161161	0.024874686
ENSG00000143630	HCN3	1456.926029	1170.948842	0.315842153	0.005166238	0.024894271
ENSG00000114841	DNAH1	1057.168274	2295.655031	-1.118585494	0.005183395	0.024969011
ENSG00000105568	PPP2R1A	9779.54771	7990.650035	0.291373736	0.005184082	0.024969011
ENSG00000114030	KPNA1	2033.783613	1668.277855	0.285433059	0.005185025	0.024969011
ENSG00000047249	ATP6V1H	1657.495367	1317.0067	0.33120833	0.005185817	0.024969011
ENSG00000242797	GLYCTK-AS1	41.87926811	75.3564209	-0.845732807	0.005187842	0.024973864
ENSG00000131100	ATP6V1E1	3178.036295	2532.514115	0.327228137	0.005190119	0.02497993
ENSG00000182308	DCAF4L1	51.76286229	26.14880619	0.982311977	0.005192485	0.024986416
ENSG00000171223	JUNB	1554.436143	2102.953716	-0.435860052	0.005196982	0.025000657
ENSG00000204147	ASAH2B	182.3090958	251.7955022	-0.464672462	0.005197481	0.025000657
ENSG00000132915	PDE6A	20.50241344	5.719642162	1.838956384	0.005202454	0.025019679
ENSG00000279924	AL356585.4	0.349140684	7.242998531	-4.213048901	0.005204645	0.025020567
ENSG00000123297	TSFM	581.6334497	770.4817212	-0.406459595	0.005206181	0.025020567
ENSG00000198162	MAN1A2	3139.941475	3772.014295	-0.264403938	0.005206645	0.025020567
ENSG00000122877	EGR2	58.55667632	29.92751543	0.959369999	0.005207234	0.025020567
ENSG00000089022	MAPKAPK5	1263.161875	1538.000223	-0.284373426	0.005208166	0.025020567
ENSG00000176463	SLCO3A1	45.56205194	78.78042228	-0.787279972	0.005208754	0.025020567
ENSG00000145354	CISD2	1009.482732	1276.636386	-0.339151063	0.005215653	0.025046071
ENSG00000085978	ATG16L1	1397.408646	1138.042707	0.295816704	0.005216104	0.025046071
ENSG00000159231	CBR3	94.62962047	58.07618791	0.704142524	0.005221732	0.025063384
ENSG00000165502	RPL36AL	2760.525266	3519.359152	-0.350476412	0.005221751	0.025063384
ENSG00000186976	EFCAB6	68.37796117	37.03289412	0.890995012	0.00523817	0.025137279
ENSG00000020129	NCDN	1701.382951	1383.879562	0.298201851	0.005239492	0.025138707
ENSG00000116857	TMEM9	2986.340162	3679.83734	-0.30117593	0.005253097	0.02519906
ENSG00000185379	RAD51D	551.6644626	697.654055	-0.33869368	0.005256946	0.025212597
ENSG00000234630	AC245060.2	115.9262379	65.51564861	0.825148471	0.005259164	0.025218307
ENSG00000228065	LINC01515	119.2015442	71.33657076	0.738581347	0.005268474	0.025258016
ENSG00000271551	AL355297.4	3.676282646	16.79435482	-2.198054004	0.005270975	0.025265074

ENSG00000173875	ZNF791	1139.943321	916.2218228	0.315304622	0.005281369	0.025305604
ENSG00000236753	MKLN1-AS	89.65302288	51.12046838	0.814085632	0.005281492	0.025305604
ENSG00000215021	PHB2	6472.463983	8010.448922	-0.307671956	0.005295779	0.025369105
ENSG00000239605	STPG4	99.3285904	62.21054332	0.674216403	0.005297013	0.02537007
ENSG00000266028	SRGAP2	1877.889833	1468.487137	0.354437168	0.005302775	0.025392712
ENSG00000084070	SMAP2	792.3645231	595.174362	0.41172069	0.005308322	0.025414318
ENSG00000132967	HMGB1P5	188.1889961	302.5820194	-0.687306049	0.00531611	0.025446642
ENSG00000174282	ZBTB4	3188.969066	4145.682816	-0.378268841	0.005322296	0.025471285
ENSG00000089177	KIF16B	738.2041516	940.4218381	-0.349398765	0.005324964	0.025479085
ENSG00000253313	C1orf210	551.3562128	405.755128	0.441923033	0.005333024	0.025506991
ENSG00000251144	AC113346.1	23.5612828	8.206337679	1.527351303	0.005333662	0.025506991
ENSG00000184232	OAF	807.7048353	1010.594138	-0.323673512	0.005333913	0.025506991
ENSG00000256525	POLG2	676.6502873	841.292946	-0.314281424	0.005339176	0.025527187
ENSG00000230426	ERVMER61-1	4.760171874	0	4.832583774	0.005341864	0.025530096
ENSG00000283311	MTND6P29	4.760171874	0	4.832583774	0.005341864	0.025530096
ENSG00000237167	AC128709.3	16.92000125	40.6249267	-1.255533121	0.005354827	0.025587069
ENSG00000161010	MRNIP	1847.464319	1500.022856	0.300918723	0.005358051	0.025594101
ENSG00000158473	CD1D	2.137812464	12.78920206	-2.561602599	0.005358384	0.025594101
ENSG00000006377	DLX6	157.8874927	224.9167582	-0.511504347	0.005364343	0.025617582
ENSG00000089916	GPATCH2L	2398.327415	1938.093171	0.30782647	0.005365614	0.025618666
ENSG00000132432	SEC61G	2606.749778	2054.830669	0.342888813	0.00536666	0.025618682
ENSG00000269439	AC010618.3	138.5016258	209.6219147	-0.597675242	0.005369351	0.025626542
ENSG00000168356	SCN11A	4.704201147	0	4.818566067	0.0053707	0.025627781
ENSG00000151176	PLBD2	3365.080878	2680.934885	0.327712002	0.005371698	0.025627781
ENSG00000158604	TMED4	3755.957171	4462.287991	-0.248680321	0.005373382	0.025630833
ENSG00000223431	MTND6P21	12.12192964	1.841203334	2.729614323	0.005380812	0.025661289
ENSG00000284474	AC099654.12	4.741938286	0	4.828005301	0.005396355	0.025727051
ENSG00000135372	NAT10	2326.157922	2993.235029	-0.364085985	0.005396697	0.025727051
ENSG00000101596	SMCHD1	3400.364396	4180.37856	-0.298117932	0.005398436	0.025730346
ENSG00000244879	GABPB1-AS1	2444.467983	3441.720344	-0.493421717	0.005402193	0.025743254
ENSG00000185515	BRCC3	927.3122843	1159.053101	-0.322432124	0.005410508	0.025777873
ENSG00000134758	RNF138	1327.470371	1687.380099	-0.346513712	0.005418454	0.025810723
ENSG00000151461	UPF2	1493.091108	1856.140246	-0.31440041	0.005419792	0.025812086
ENSG00000250305	TRMT9B	76.82106824	144.690298	-0.910661602	0.005430191	0.025856597
ENSG00000100393	EP300	2148.478335	2589.415466	-0.26933728	0.005448159	0.025937124
ENSG00000115677	HDLBP	27803.49795	33401.43405	-0.264632187	0.005451701	0.025947483
ENSG00000099810	MTAP	2185.743045	1604.835093	0.445139398	0.005452448	0.025947483
ENSG00000273373	AL355488.1	297.6413964	212.238895	0.490313333	0.005454889	0.025950733
ENSG00000222317	RNA5SP118	4.722434735	0	4.823156162	0.005455246	0.025950733
ENSG00000112787	FBRSL1	3359.805571	2764.045435	0.281377268	0.00546093	0.02597274
ENSG00000183036	PCP4	6.572308462	21.20926629	-1.692479644	0.005464787	0.025986053
ENSG00000160194	NDUFV3	909.4433094	1123.429031	-0.30514882	0.005470415	0.026007778
ENSG00000135253	KCP	64.66790265	109.5615797	-0.757162219	0.005479712	0.026046931

ENSG00000184274	LINC00315	30.37189787	11.55715106	1.396188645	0.005500379	0.026140109
ENSG00000204248	COL11A2	408.6458971	258.3449285	0.66409418	0.005504201	0.026153213
ENSG00000121741	ZMYM2	3363.529989	4187.559472	-0.315930677	0.005511935	0.026184893
ENSG00000159423	ALDH4A1	502.6168995	700.4071045	-0.479590785	0.005514933	0.02619304
ENSG00000134256	CD101	51.73670619	25.04002936	1.052107226	0.005516638	0.02619304
ENSG00000167912	AC090152.1	0	5.092795428	-4.66848562	0.005516851	0.02619304
ENSG00000167720	SRR	208.0652579	141.1280771	0.556274717	0.005532518	0.026254326
ENSG00000249626	AC024560.2	85.75634204	143.005881	-0.736846271	0.005532955	0.026254326
ENSG00000135437	RDH5	99.840726	55.6738597	0.850221952	0.005533622	0.026254326
ENSG00000141447	OSBPL1A	2152.835254	1745.287513	0.302787014	0.005536289	0.026254326
ENSG00000146477	SLC22A3	27.65964048	8.36303297	1.719534315	0.005537703	0.026254326
ENSG00000141385	AFG3L2	2343.886377	2894.123284	-0.30450731	0.005537954	0.026254326
ENSG00000050405	LIMA1	2262.610734	2845.365012	-0.330828254	0.005538168	0.026254326
ENSG00000007202	KIAA0100	5995.440316	4904.916245	0.289474507	0.005538314	0.026254326
ENSG00000168993	CPLX1	144.9258396	211.698487	-0.545201669	0.005541369	0.026259011
ENSG00000170448	NFXL1	1050.597423	1301.269758	-0.309130901	0.005541715	0.026259011
ENSG00000259230	LINC02323	78.45103154	44.69743002	0.810713374	0.005542511	0.026259011
ENSG00000170903	MSANTD4	728.6822127	577.2399592	0.336157747	0.005551002	0.026294163
ENSG00000137101	CD72	152.9104156	93.03688775	0.721944055	0.005565302	0.026356815
ENSG00000285730	Z94721.3	13.17316159	34.16795891	-1.378712012	0.005570862	0.026378057
ENSG00000124772	CPNE5	12.39163479	2.342287188	2.378045769	0.005574626	0.02639079
ENSG00000222071	MIR1915	48.1804501	23.48721268	1.030136887	0.005583904	0.026429005
ENSG00000126461	SCAF1	4811.505232	3829.956029	0.328914442	0.005584851	0.026429005
ENSG00000123983	ACSL3	5847.688124	4653.679084	0.32937962	0.005589803	0.026447343
ENSG00000146281	PM20D2	2643.987511	3255.399618	-0.300384468	0.005592496	0.026454984
ENSG00000059588	TARBP1	2015.794777	2493.384609	-0.306479539	0.00560303	0.026495143
ENSG00000173267	SNCG	387.960974	280.3549882	0.467157452	0.005603144	0.026495143
ENSG00000261655	AC100803.2	1.831640139	11.18921792	-2.618210211	0.005606068	0.026503865
ENSG00000265828	MIR3939	2.217247999	13.14097846	-2.583980752	0.00562718	0.026598556
ENSG00000089159	PXN	7243.889137	6022.297051	0.266378499	0.005639356	0.026650978
ENSG00000123870	ZNF137P	58.04216338	26.16247421	1.157262918	0.005641852	0.026657643
ENSG00000163110	PDLIM5	998.9958205	1323.268951	-0.406298286	0.005644304	0.026664097
ENSG00000083817	ZNF416	248.4458676	176.1128954	0.497718601	0.005658258	0.026724871
ENSG00000059378	PARP12	980.0650028	785.8259334	0.318342657	0.005662242	0.026738547
ENSG00000279431	AC011840.5	19.50335355	5.850304168	1.753297768	0.005675262	0.026794878
ENSG00000167733	HSD11B1L	63.9214217	100.8532981	-0.658468704	0.005680881	0.026816247
ENSG00000243364	EFNA4	593.0669183	438.8237951	0.434967381	0.005688151	0.026845406
ENSG00000234509	AP000253.1	37.24514744	14.7610674	1.342902063	0.005692733	0.026861866
ENSG00000204580	DDR1	8759.747732	7199.37834	0.283017557	0.005696965	0.026876668
ENSG00000149243	KLHL35	175.2076769	267.57019	-0.609236644	0.005700115	0.026886365
ENSG00000156735	BAG4	963.6259348	1268.033177	-0.396746337	0.005706047	0.026909175
ENSG00000135956	TMEM127	2736.532556	2265.540489	0.272736574	0.005707796	0.026912251
ENSG00000205464	ATP6AP1L	684.2248123	504.7621801	0.440228102	0.005719315	0.026961386

ENSG00000162585	FAAP20	2621.664054	2167.698007	0.274540296	0.005721013	0.026964212
ENSG00000151718	WWC2	2844.956774	3567.657966	-0.326627738	0.005740057	0.027048781
ENSG00000188856	RPSAP47	628.4874317	827.388009	-0.39785921	0.00575309	0.027104992
ENSG00000178741	COX5A	2549.925046	3278.635148	-0.362881933	0.005755461	0.027110961
ENSG00000130876	SLC7A10	105.1991328	58.60124635	0.849747841	0.00575868	0.027120918
ENSG00000107758	PPP3CB	1517.672841	1919.612066	-0.339437669	0.005762709	0.027134689
ENSG00000273428	AC004832.6	18.80380222	5.47842296	1.786859774	0.005781134	0.027216227
ENSG00000134283	PPHLN1	1894.383324	2282.037963	-0.268680867	0.005783012	0.027219848
ENSG00000125534	PPDPF	8886.34799	7375.666262	0.268732477	0.005784587	0.027221962
ENSG00000274225	AP001065.1	27.36647053	8.465251278	1.693710959	0.005785679	0.027221962
ENSG00000204421	LY6G6C	10.96510606	30.94677818	-1.494333408	0.005789216	0.027233387
ENSG00000270959	LPP-AS2	85.12100913	46.64713185	0.874871615	0.005795111	0.027255892
ENSG00000108349	CASC3	3044.268906	3763.205058	-0.306091088	0.005813513	0.027337207
ENSG00000185436	IFNLR1	545.3840541	713.8192835	-0.389401431	0.005829192	0.027405688
ENSG00000116984	MTR	3330.41603	2614.655188	0.348719736	0.005835447	0.027429843
ENSG00000205592	MUC19	4.778405462	0	4.837269287	0.005843602	0.027462915
ENSG00000170759	KIF5B	10377.61416	12947.97843	-0.319301932	0.005847756	0.027477177
ENSG00000180346	TIGD2	502.9580811	636.9882498	-0.341505901	0.005852303	0.027493281
ENSG00000174945	AMZ1	41.23441755	17.49782931	1.246073007	0.005857955	0.027514571
ENSG00000089693	MLF2	8004.138184	6012.219985	0.41268728	0.005863184	0.027533863
ENSG00000168259	DNAJC7	2424.324132	2890.313145	-0.2538143	0.005871504	0.02756766
ENSG00000124713	GNMT	27.00305751	52.27107079	-0.956874089	0.005883197	0.027614002
ENSG00000259862	AC026336.2	8.03657433	0.577711617	3.749388717	0.005883624	0.027614002
ENSG00000185022	MAFF	1072.296491	801.7678048	0.418565691	0.005888808	0.027633052
ENSG00000205981	DNAJC19	909.3418416	1111.903035	-0.290471398	0.005903909	0.02769862
ENSG00000167110	GOLGA2	4137.059339	3397.684223	0.283958651	0.005905481	0.027700701
ENSG00000227726	AP001271.1	1.433029912	10.71957479	-2.885710952	0.005912586	0.027728728
ENSG00000137818	RPLP1	24571.49665	30172.72071	-0.296273909	0.005913851	0.027729365
ENSG00000121068	TBX2	19.61529501	41.13734647	-1.067417521	0.005919847	0.027752179
ENSG00000134986	NREP	260.3543768	182.8640873	0.512496377	0.005944771	0.027863704
ENSG00000100031	GGT1	539.5033604	837.9535987	-0.634219104	0.005955244	0.027907464
ENSG00000139697	SBNO1	3905.24369	4851.728104	-0.313274245	0.005965383	0.027949643
ENSG00000118922	KLF12	1713.775691	1346.388507	0.348711888	0.005970504	0.027968298
ENSG00000131127	ZNF141	396.3406673	305.006732	0.378277113	0.005985504	0.028033215
ENSG00000234745	HLA-B	1813.051537	2372.743221	-0.387816673	0.005998289	0.028087735
ENSG00000231711	LINC00899	308.1206856	222.792419	0.47017076	0.006002994	0.028104407
ENSG00000117222	RBBP5	1156.045036	895.2716264	0.367828022	0.006005702	0.028111723
ENSG00000180185	FAHD1	856.2106473	666.9460471	0.359373359	0.006009055	0.028122055
ENSG00000110721	CHKA	936.4023024	1290.799767	-0.462445301	0.006017353	0.028155525
ENSG00000257178	AC103702.1	619.2610147	238.9040821	1.375055092	0.006037106	0.028242566
ENSG00000204524	ZNF805	267.8897545	192.1196946	0.480715841	0.006044457	0.028271567
ENSG00000121957	GPSM2	2312.051357	2951.685073	-0.352398392	0.006047815	0.028281887
ENSG00000205758	CRYZL1	520.8252842	658.6319143	-0.339330765	0.006052333	0.028297627

ENSG00000166436	TRIM66	1896.527604	2464.63378	-0.377686322	0.006065198	0.028352376
ENSG00000125798	FOXA2	33.04642934	12.78284357	1.362769108	0.006083352	0.028426724
ENSG00000027075	PRKCH	521.5026406	714.5848704	-0.455618401	0.006083419	0.028426724
ENSG00000232237	ASCL5	164.8680804	108.9500169	0.602403861	0.00609669	0.02847837
ENSG00000283041	AC008038.1	138.5475994	198.7596258	-0.521683461	0.006096791	0.02847837
ENSG00000170965	PLAC1	0.741249728	9.331779553	-3.668659973	0.006109564	0.02853146
ENSG00000101665	SMAD7	2709.204725	2133.136837	0.345247593	0.006110946	0.02853146
ENSG00000253671	AC027117.1	17.97265452	42.66852068	-1.237695935	0.006111643	0.02853146
ENSG00000150244	TRIM48	4.765403095	0	4.834061213	0.006130878	0.028615816
ENSG00000276345	AC004556.1	1.470767051	12.83584846	-3.129806316	0.006140077	0.028653306
ENSG00000183617	MRPL54	554.3464259	709.1640128	-0.35588972	0.006144398	0.028668018
ENSG00000121905	HPCA	12.3499364	33.01640545	-1.41321923	0.006149402	0.028685914
ENSG00000263731	AC145207.5	63.56863371	105.8763326	-0.732675703	0.006150943	0.028687654
ENSG00000174498	IGDCC3	2.572889867	13.67247368	-2.411995695	0.006154657	0.028699521
ENSG00000137804	NUSAP1	399.400958	560.1651009	-0.489075322	0.006162214	0.028729306
ENSG00000163982	OTOP1	65.20589161	31.66057197	1.042551747	0.006167425	0.02874814
ENSG00000090565	RAB11FIP3	2492.034126	3059.185737	-0.295600299	0.00617369	0.028771881
ENSG00000140943	MBTPS1	5033.438087	5996.074144	-0.252552425	0.006184313	0.028815818
ENSG00000272894	AC004982.2	89.6582541	138.3523173	-0.623362918	0.006185465	0.028815818
ENSG00000164975	SNAPC3	2155.752917	1742.660878	0.306481166	0.006190427	0.028831947
ENSG00000229124	VIM-AS1	24.34296096	8.9235585	1.460516429	0.006192109	0.028831947
ENSG00000233936	AL390778.2	12.90741769	1.827457011	2.825461269	0.006192451	0.028831947
ENSG00000189180	ZNF33A	1709.602806	2069.395551	-0.275574242	0.006198077	0.028852671
ENSG00000251532	AC091849.2	6.59054205	0.331593289	4.340165013	0.006200216	0.028857155
ENSG00000197181	PIWIL2	59.31488967	110.5793831	-0.895300332	0.006212934	0.028910867
ENSG00000257702	LBX2-AS1	192.1473442	261.0889742	-0.44165972	0.00622824	0.028976602
ENSG00000147854	UHRF2	1866.358373	2337.420075	-0.324378936	0.006241962	0.029034938
ENSG00000253651	SOD1P3	14.65312112	3.3822541	2.126462238	0.006257278	0.029099886
ENSG00000131238	PPT1	4131.39243	5006.0753	-0.27711584	0.006258295	0.029099886
ENSG00000237952	RPL7AP73	15.66930721	3.377354975	2.226307859	0.006260158	0.029103036
ENSG00000268858	AL118506.1	365.0115481	274.2785012	0.412461609	0.006279071	0.029185435
ENSG00000120738	EGR1	3996.0767	1944.125672	1.039377404	0.00628629	0.029213461
ENSG00000196547	MAN2A2	2672.601799	3336.205141	-0.319843537	0.006288833	0.029219746
ENSG00000222020	AC062017.1	66.18417798	36.24746273	0.86108757	0.006299527	0.029263897
ENSG00000270704	AC124312.4	4.715933551	0	4.82151978	0.006303882	0.029278588
ENSG00000249464	LINC01091	1.433029912	10.73332111	-2.887656303	0.006321245	0.029352008
ENSG00000111452	ADGRD1	13.78819753	2.965185848	2.204132614	0.0063221	0.029352008
ENSG00000116833	NR5A2	34.856026	61.99191764	-0.831275781	0.006323277	0.029352008
ENSG00000115641	FHL2	1607.498037	1228.076127	0.387725991	0.00633159	0.029380527
ENSG00000214425	LRRC37A4P	722.4691905	951.0687863	-0.395574706	0.006331814	0.029380527
ENSG00000223508	RPL23AP53	107.2958777	66.28785472	0.699589555	0.006348665	0.02945315
ENSG00000225339	AL354740.1	24.71033523	8.868064897	1.465662796	0.006351988	0.029462998
ENSG00000272141	AL390719.2	228.4405128	389.1665768	-0.76711393	0.006357503	0.029483008

ENSG00000272595	OR10AH1P	0	5.694559928	-4.819798696	0.006359345	0.029485983
ENSG00000198839	ZNF277	1220.907293	964.6417252	0.340358698	0.006365479	0.029508851
ENSG00000243147	MRPL33	775.2799543	610.4786122	0.343849278	0.00637223	0.029534572
ENSG00000077238	IL4R	836.9895106	1150.608657	-0.459863833	0.006377431	0.029553098
ENSG00000175611	LINC00476	93.95321999	142.41699	-0.599257037	0.006383398	0.029575165
ENSG00000198888	MT-ND1	75651.43908	89598.10595	-0.244107256	0.006406165	0.02967505
ENSG00000135932	CAB39	4209.800673	3385.138838	0.314278247	0.006409279	0.029678994
ENSG00000060069	CTDP1	1504.688993	1152.245373	0.384257908	0.006409434	0.029678994
ENSG00000171368	TPPP	442.1877929	332.2701397	0.413786823	0.006433754	0.029785988
ENSG00000182957	SPATA13	642.4397286	458.5973695	0.484545268	0.006458457	0.029894718
ENSG00000173473	SMARCC1	7347.762166	9297.204372	-0.339588438	0.006470805	0.029946226
ENSG00000269656	NOP53-AS1	8.355749022	24.10177245	-1.52856622	0.00648249	0.02999465
ENSG00000188493	C19orf54	1072.411416	1310.676564	-0.289007846	0.006487026	0.030004926
ENSG00000180176	TH	5.794591559	21.05704012	-1.866721921	0.006487155	0.030004926
ENSG00000261379	AC010735.1	18.12629437	4.883016946	1.899872043	0.006492313	0.030023125
ENSG00000179981	TSHZ1	847.1041055	1056.812529	-0.318761229	0.006499882	0.030052468
ENSG00000188725	SMIM15	1571.749362	1891.094183	-0.267059451	0.006501669	0.03005507
ENSG00000153885	KCTD15	1586.86036	2088.675529	-0.396114055	0.00650515	0.030065501
ENSG00000204525	HLA-C	8749.137938	7141.377387	0.292857355	0.006527325	0.030162309
ENSG00000131095	GFAP	11.23354125	2.09616886	2.417096416	0.00653828	0.030207248
ENSG00000105887	MTPN	7459.194873	6032.990854	0.306003747	0.006553731	0.030272935
ENSG00000197506	SLC28A3	0	5.217098949	-4.709697814	0.006556252	0.030278881
ENSG00000065060	UHRF1BP1	3348.495574	4095.78499	-0.290747499	0.006562594	0.03030247
ENSG00000189343	RPS2P46	2388.526281	1963.176211	0.28279133	0.006575113	0.030341582
ENSG00000169490	TM2D2	975.9661881	770.7350453	0.340146705	0.006575175	0.030341582
ENSG00000206053	JPT2	4577.152683	5574.423335	-0.284477115	0.006575698	0.030341582
ENSG00000065615	CYB5R4	384.5260174	285.0302726	0.429759475	0.006576007	0.030341582
ENSG00000171858	RPS21	6763.848174	8519.839358	-0.333051085	0.006578973	0.030349562
ENSG00000254122	PCDHGB7	16.9122301	38.13531246	-1.168815259	0.006605915	0.030468124
ENSG00000181722	ZBTB20	179.0962614	101.6838255	0.821653721	0.006625105	0.030550895
ENSG00000148950	IMMP1L	288.3921679	394.318489	-0.452941458	0.006638259	0.030605802
ENSG00000254198	AC113191.1	211.9703378	300.109617	-0.501153699	0.006641795	0.030616354
ENSG00000253311	LINC01847	6.174968198	0.277559048	4.248245742	0.006652998	0.030662239
ENSG00000248092	NNT-AS1	635.5168581	507.4595666	0.325269512	0.006658338	0.030681092
ENSG00000014123	UFL1	2231.582556	2677.684727	-0.262987671	0.006662111	0.030692716
ENSG00000168101	NUDT16L1	992.523578	1270.653817	-0.355821648	0.006683362	0.030784842
ENSG00000140990	NDUFB10	2039.952228	2619.047179	-0.360828473	0.006706337	0.030884877
ENSG00000198561	CTNND1	12408.11488	14745.31718	-0.248964059	0.006718783	0.03093639
ENSG00000272273	IER3-AS1	12.65483876	2.297100144	2.425771095	0.006722442	0.030947438
ENSG00000181704	YIPF6	2403.859139	2876.39592	-0.258765056	0.006725588	0.030956113
ENSG00000249352	LINC02198	17.33430513	4.932152063	1.830525076	0.006726866	0.030956194
ENSG00000165233	CARD19	1056.412937	840.4589916	0.329504463	0.006728158	0.030956335
ENSG00000261305	AC005586.2	18.8792765	6.024693857	1.647004418	0.006731688	0.030966776

ENSG00000258572	AL133467.1	10.24732115	1.294932438	3.043377003	0.006733038	0.03096718
ENSG00000066427	ATXN3	903.7513296	1164.839103	-0.365918306	0.006739152	0.030989495
ENSG00000115718	PROC	10.11841607	26.76285765	-1.400719929	0.006752852	0.031046681
ENSG00000075303	SLC25A40	1010.636396	1326.160599	-0.392533719	0.006761074	0.03107866
ENSG00000138738	PRDM5	329.6322962	438.3083143	-0.412022785	0.006767902	0.031104224
ENSG00000251364	AC107884.1	79.25713052	42.09226842	0.922079466	0.006778076	0.031139484
ENSG00000105246	EBI3	30.1205889	12.84572501	1.22403102	0.006778652	0.031139484
ENSG00000162664	ZNF326	1624.297065	1998.888256	-0.299673758	0.006779379	0.031139484
ENSG00000109320	NFKB1	1371.34156	1752.360798	-0.354256866	0.006796892	0.031214083
ENSG00000175356	SCUBE2	12.03726289	29.43279006	-1.294271058	0.006801178	0.031227927
ENSG00000123178	SPRYD7	556.7029042	381.6773043	0.542417619	0.006804958	0.031239438
ENSG00000101361	NOP56	5371.905314	6764.749319	-0.332748211	0.006830191	0.031346858
ENSG00000173531	MST1	390.3394592	647.6681096	-0.729630246	0.006830911	0.031346858
ENSG00000265681	RPL17	500.4230686	658.2572837	-0.396706445	0.006842695	0.031395067
ENSG00000263412	AC004477.1	46.02694397	21.21665413	1.113828157	0.006844985	0.031399707
ENSG00000136986	DERL1	1831.395132	1507.708462	0.280238753	0.006858736	0.03145691
ENSG00000099290	WASHC2A	2034.555793	2528.788774	-0.313340188	0.006867628	0.031491807
ENSG00000160233	LRRC3	315.3433786	226.582178	0.477347196	0.00687576	0.031523208
ENSG00000122512	PMS2	961.0812279	1177.482528	-0.293415685	0.006889043	0.031578211
ENSG00000151376	ME3	596.4741603	474.5133521	0.330712187	0.006919579	0.031708273
ENSG00000204519	ZNF551	599.3790788	452.5325446	0.405029635	0.00692	0.031708273
ENSG00000166349	RAG1	121.3540943	79.15866197	0.619841206	0.006923536	0.031718552
ENSG00000230105	AL354793.1	6.224437742	0.331593289	4.257492384	0.006941132	0.031793234
ENSG00000164331	ANKRA2	475.077249	603.2822858	-0.343845515	0.006953827	0.031845442
ENSG00000273729	AC007686.3	46.5051411	78.35691724	-0.75181076	0.006955128	0.031845459
ENSG00000085831	TTC39A	848.1748635	687.8243452	0.302384868	0.006975088	0.031930894
ENSG00000233178	AL161457.2	14.35884378	2.965185848	2.266333926	0.006976646	0.031932072
ENSG00000005302	MSL3	926.2185718	751.0891883	0.302204591	0.006982999	0.031951779
ENSG00000226239	AL031658.1	6.756076892	0.300152569	4.383020731	0.006983555	0.031951779
ENSG00000143368	SF3B4	3020.488319	2473.056827	0.288182223	0.00698918	0.031971559
ENSG00000057935	MTA3	1451.796859	1784.405865	-0.297276471	0.00699781	0.032005073
ENSG00000266283	AC091588.3	6.491602964	22.16865737	-1.765624597	0.007005272	0.032033234
ENSG00000018625	ATP1A2	21.40000553	6.239371464	1.769277484	0.007012594	0.032060745
ENSG00000198056	PRIM1	467.846157	620.5798357	-0.408767138	0.007019065	0.032084353
ENSG00000274928	KRT89P	15.64457244	4.277812683	1.880167902	0.007044733	0.03219569
ENSG00000106546	AHR	880.3692261	609.2831365	0.532073927	0.007051538	0.032220789
ENSG00000267040	AC027097.1	22.89296743	45.90980622	-1.001531877	0.007077915	0.032335298
ENSG00000180263	FGD6	566.79515	744.4950833	-0.394054361	0.007099623	0.032428435
ENSG00000164221	CCDC112	339.8498251	468.9666145	-0.466081598	0.007103401	0.032439658
ENSG00000214894	LINC00243	4.30559092	19.12040697	-2.138789123	0.007120748	0.032512829
ENSG00000146872	TLK2	1831.021739	2251.2908	-0.298493885	0.007128992	0.032544417
ENSG00000173511	VEGFB	1027.867005	804.8285248	0.353575868	0.007131688	0.032550675
ENSG00000284602	AL031432.4	58.60614586	94.29505034	-0.685524689	0.007135208	0.032560687

ENSG00000183255	PTTG1IP	9800.499296	7722.242721	0.34397204	0.007137419	0.032564723
ENSG00000118495	PLAGL1	1246.953987	1566.848318	-0.329666512	0.007143325	0.032585616
ENSG00000236756	DNAJC9-AS1	31.32054342	11.78213522	1.426776011	0.007145442	0.032589217
ENSG00000232533	AC093673.1	134.5077523	84.38520746	0.668176015	0.007174747	0.032716793
ENSG00000212135	SNORD67	26.47554221	10.01858901	1.38874855	0.007181529	0.032741638
ENSG00000145779	TNFAIP8	587.0384833	423.9066715	0.468046438	0.00718534	0.032752932
ENSG00000159905	ZNF221	20.31230641	6.436862983	1.669361659	0.007189397	0.032760157
ENSG00000166086	JAM3	62.9586664	103.6785448	-0.723743075	0.007189594	0.032760157
ENSG00000135535	CD164	8945.50947	10512.10009	-0.232783235	0.007194673	0.032777215
ENSG00000265692	LINC01970	21.34149488	43.75960311	-1.031000147	0.007200815	0.032799112
ENSG00000197043	ANXA6	1731.565961	2112.30538	-0.286865551	0.007209934	0.032834557
ENSG00000166147	FBN1	47.76233632	18.82136205	1.329447642	0.00723173	0.032927706
ENSG00000183397	C19orf71	365.7239617	508.0534862	-0.473106448	0.007237101	0.032946055
ENSG00000113328	CCNG1	3562.614145	4301.536224	-0.272015492	0.007256324	0.033027442
ENSG00000165118	C9orf64	993.5283037	1245.907322	-0.327254427	0.007258319	0.033030396
ENSG00000104412	EMC2	928.5047816	1160.529459	-0.322437998	0.007270619	0.033080237
ENSG00000183520	UTP11	1333.425874	1002.944966	0.409985826	0.007280766	0.033120266
ENSG00000104427	ZC2HC1A	269.8055961	356.7988697	-0.403124758	0.007291169	0.033161445
ENSG00000268895	A1BG-AS1	13.79469871	3.3822541	2.042802353	0.007306237	0.033223822
ENSG00000138207	RBP4	0.734748544	8.899505618	-3.614605942	0.007308869	0.033229637
ENSG00000105677	TMEM147	2128.547364	1596.138796	0.414772321	0.007331238	0.033325165
ENSG00000105696	TMEM59L	18.10679082	5.563897922	1.72033575	0.007333163	0.033327748
ENSG00000145808	ADAMTS19	46.64831851	22.8328733	1.021039726	0.007355659	0.033423797
ENSG00000273056	AL354694.1	24.90821341	8.049134078	1.620590521	0.007357656	0.033426684
ENSG00000112578	BYSL	636.2818978	885.6162023	-0.477843401	0.007369253	0.033473179
ENSG00000123600	METTL8	517.1788049	700.359168	-0.438699202	0.007381307	0.033521727
ENSG00000147439	BIN3	441.96264	342.2602977	0.369434058	0.007383636	0.033526105
ENSG00000166856	GPR182	20.81508695	7.028320923	1.573281684	0.007386492	0.033532869
ENSG00000151366	NDUFC2	183.7300681	249.8795732	-0.444139578	0.007393663	0.033559221
ENSG00000163357	DCST1	84.72477624	49.98479825	0.754961907	0.007399763	0.0335807
ENSG00000153790	C7orf31	176.3878139	124.4397321	0.501506701	0.007414037	0.033639257
ENSG00000196656	AC004057.1	73.60476036	112.5976341	-0.612985861	0.007433072	0.033718621
ENSG00000058056	USP13	1572.45905	1924.987112	-0.292228768	0.007434275	0.033718621
ENSG00000148331	ASB6	1315.351261	1062.069628	0.307877949	0.007440479	0.033740526
ENSG00000224598	RPS5P2	6.163235794	0.300152569	4.246014408	0.007445069	0.033755103
ENSG00000156508	EEF1A1	301475.3584	362215.8541	-0.264807516	0.007450465	0.033773333
ENSG00000171155	C1GALT1C1	604.4132281	766.2788292	-0.342592181	0.007457313	0.033798132
ENSG00000250398	AC097467.2	4.790137866	0	4.840580306	0.007466915	0.033829962
ENSG00000267681	AC135721.1	13.17712284	35.14891415	-1.414809617	0.007467092	0.033829962
ENSG00000168143	FAM83B	313.4897062	408.8802668	-0.382057161	0.00746922	0.033833359
ENSG00000156170	NDUFAF6	411.3186709	539.2520378	-0.38975723	0.007472776	0.033843226
ENSG00000243156	MICAL3	1534.613733	1906.58605	-0.313519393	0.007475219	0.033848045
ENSG00000274373	AC148476.1	22.48785601	8.269219119	1.454155844	0.007487808	0.033898798

ENSG00000168569	TMEM223	531.6606917	671.1041422	-0.336103264	0.007493295	0.033916498
ENSG00000203668	CHML	1561.47718	2007.867127	-0.363123362	0.007494481	0.033916498
ENSG00000227473	TSSK5P	101.0024668	63.3650155	0.670876144	0.007504621	0.033956126
ENSG00000139182	CLSTN3	3236.093538	2614.619902	0.307926042	0.007510878	0.033978178
ENSG00000110723	EXPH5	2216.376215	1665.649227	0.411625625	0.007546781	0.034134306
ENSG00000234136	AC055764.1	4.386296419	0	4.714996603	0.007565588	0.03421307
ENSG00000261824	LINC00662	331.4097121	459.3289347	-0.470991349	0.007567264	0.034214344
ENSG00000169062	UPF3A	2683.945846	3191.132816	-0.249872473	0.007590254	0.034311973
ENSG00000240429	LRRFIP1P1	148.7348372	101.9138782	0.54828464	0.00761378	0.034411988
ENSG00000068650	ATP11A	5664.532027	4750.11818	0.254099031	0.00762107	0.034432745
ENSG00000178718	RPP25	6.524108883	21.06631732	-1.686038279	0.007621178	0.034432745
ENSG00000238273	AC108058.1	62.71385861	34.796265	0.856548642	0.007623964	0.034438997
ENSG00000108465	CDK5RAP3	911.4057974	1287.524904	-0.497759009	0.007634302	0.034473282
ENSG00000265808	SEC22B	3685.491414	3071.651671	0.262678788	0.007634362	0.034473282
ENSG00000137767	SQOR	191.5714668	259.1230246	-0.434871257	0.00763677	0.034477812
ENSG00000149582	TMEM25	11.30251434	30.75864217	-1.439255084	0.007638684	0.034480112
ENSG00000148429	USP6NL	2833.116708	3388.971489	-0.258428075	0.007646971	0.034511174
ENSG00000196411	EPHB4	2410.568892	2925.536056	-0.279536504	0.007656436	0.03454754
ENSG00000183291	SELENOF	2952.443317	3509.132312	-0.249134534	0.007659585	0.034555397
ENSG00000155876	RRAGA	1797.973439	1475.33446	0.285131182	0.007670745	0.034599386
ENSG00000100351	GRAP2	6.107265067	0.331593289	4.235437911	0.007677116	0.034621764
ENSG00000166866	MYO1A	43.15057307	75.1902918	-0.800099714	0.007697987	0.034709511
ENSG00000169684	CHRNA5	321.1127588	433.9226152	-0.436239283	0.007702038	0.034718465
ENSG00000204282	TNRC6C-AS1	391.7969212	580.8987417	-0.567102036	0.007702801	0.034718465
ENSG00000119421	NDUFA8	1689.947697	1314.630822	0.361663636	0.007711677	0.034752089
ENSG00000188825	LINC00910	288.5044345	204.918826	0.49653526	0.007718572	0.034771606
ENSG00000082898	XPO1	12880.9955	16098.75631	-0.321754296	0.00771884	0.034771606
ENSG00000100445	SDR39U1	516.0546498	405.4399648	0.348132234	0.007726143	0.034798118
ENSG00000142973	CYP4B1	31.11854141	13.49121719	1.203582793	0.007729659	0.034807567
ENSG00000141429	GALNT1	1659.527628	2060.130836	-0.312309657	0.007738189	0.034839589
ENSG00000100918	REC8	132.8467157	205.8642486	-0.6297007	0.007749602	0.034884578
ENSG00000269584	TDGF1P7	4.752400727	0	4.830934964	0.007763006	0.034938506
ENSG00000232811	AL360270.1	30.08412172	12.79169077	1.226434614	0.007769768	0.034962531
ENSG00000260496	AC009041.1	7.877703259	0.631745859	3.692050811	0.007793083	0.035061019
ENSG00000104903	LYL1	26.14986633	49.90764944	-0.931302706	0.007796972	0.035070181
ENSG00000198752	CDC42BPB	9054.420702	10819.96505	-0.257028579	0.007797976	0.035070181
ENSG00000106384	MOGAT3	15.33713015	37.27016525	-1.280745911	0.007819296	0.03515962
ENSG00000162378	ZYG11B	1852.662411	2251.305902	-0.281327502	0.007824039	0.035174508
ENSG00000281189	GHET1	53.39155562	24.23492317	1.138492916	0.007826445	0.035178883
ENSG00000115966	ATF2	1619.511045	2013.520941	-0.313717906	0.007829257	0.035185079
ENSG00000214558	AL365217.1	15.19126145	4.160897	1.859055914	0.007851703	0.035279496
ENSG00000173757	STAT5B	1973.283832	2345.65694	-0.249478728	0.007854288	0.035284654
ENSG00000132950	ZMYM5	430.2074538	574.818073	-0.416523791	0.007872092	0.035358164

ENSG00000108946	PRKAR1A	9402.843011	7993.238826	0.234340201	0.007881253	0.035392839
ENSG00000129925	TMEM8A	3647.551896	2994.808731	0.284242983	0.007888237	0.035417722
ENSG00000229950	TFAP2A-AS1	36.05581794	64.18481907	-0.832291007	0.007892963	0.03543246
ENSG00000095209	TMEM38B	727.1209197	540.7571144	0.425752453	0.007894888	0.035434624
ENSG00000278725	AC020765.4	6.561846022	23.56032238	-1.841136168	0.007897923	0.035437842
ENSG00000141562	NARF	1778.267559	1465.872593	0.279097524	0.007898492	0.035437842
ENSG00000167656	LY6D	35.17916195	16.38123464	1.106655719	0.007905559	0.035456778
ENSG00000167522	ANKRD11	9713.598133	11488.25668	-0.242104289	0.007905601	0.035456778
ENSG00000129292	PHF20L1	1763.612555	1476.367235	0.256321227	0.007910138	0.035470649
ENSG00000162595	DIRAS3	23.2720741	7.882132227	1.542002184	0.007918611	0.035502157
ENSG00000279071	AC073592.3	32.6505104	13.30454054	1.309184213	0.007920341	0.035503432
ENSG00000249738	AC008691.1	49.48710364	25.10240249	0.979924869	0.007929471	0.035537867
ENSG00000232324	AC008440.3	24.97084791	52.50937127	-1.074564752	0.007937834	0.035568855
ENSG00000079691	CARMIL1	1264.438756	1599.837232	-0.339559859	0.007946707	0.035600614
ENSG00000254944	ATP5PBP5	10.02074694	1.272338916	3.027103389	0.007949698	0.035600614
ENSG00000245571	FAM111A-DT	269.2409603	363.3368433	-0.430967398	0.007950488	0.035600614
ENSG00000143252	SDHC	4263.272253	3604.35843	0.242239324	0.007950722	0.035600614
ENSG00000182093	WRB	432.4216966	570.0109419	-0.398766536	0.00795231	0.035601231
ENSG00000061938	TNK2	2033.965952	1621.750167	0.327222144	0.007959336	0.035624861
ENSG00000179152	TCAIM	745.8950699	913.6177033	-0.292327911	0.00796049	0.035624861
ENSG00000165752	STK32C	452.8996892	346.396138	0.38618029	0.007968713	0.035655161
ENSG00000162971	TYW5	393.8319846	501.2056015	-0.348412218	0.007982846	0.035705616
ENSG00000146243	IRAK1BP1	267.4989154	358.3190724	-0.420433302	0.007982898	0.035705616
ENSG00000234521	AC005041.2	4.392797602	0	4.716785609	0.007986528	0.035715347
ENSG00000181856	SLC2A4	58.83684391	98.13335785	-0.739936299	0.008000455	0.035764749
ENSG00000173221	GLRX	898.8245097	654.623528	0.457524846	0.008000489	0.035764749
ENSG00000156298	TSPAN7	53.65443441	23.53772885	1.19704657	0.008033438	0.035905502
ENSG00000100227	POLDIP3	3368.953915	3972.860864	-0.237885647	0.008050946	0.035977206
ENSG00000071655	MBD3	3186.243823	3787.341619	-0.24919649	0.008060016	0.036011185
ENSG00000198804	MT-CO1	503766.7912	412840.0807	0.287175192	0.00807572	0.036072817
ENSG00000154511	FAM69A	275.9304527	176.1103026	0.644916973	0.00807675	0.036072817
ENSG00000208005	MIR503	4.373294051	0	4.711533027	0.008079274	0.036077528
ENSG00000181649	PHLDA2	1930.065737	1344.247122	0.521244561	0.008094345	0.036136905
ENSG00000109079	TNFAIP1	2389.784118	1874.453829	0.350062988	0.008095514	0.036136905
ENSG00000151651	ADAM8	436.143964	321.5921683	0.441632536	0.008101453	0.036156841
ENSG00000134444	RELCH	1851.86615	2298.324017	-0.311940927	0.008136626	0.036307215
ENSG00000107560	RAB11FIP2	747.2784789	914.5749189	-0.291509193	0.008144377	0.036335198
ENSG00000172361	CFAP53	88.89750082	50.3555718	0.824325986	0.008154277	0.036372756
ENSG00000122068	FYTTD1	3675.959666	3070.292114	0.25952758	0.008176021	0.036463122
ENSG00000241839	PLEKHO2	302.2737481	214.368915	0.497527555	0.008190009	0.036515815
ENSG00000214176	PLEKHM1P1	980.36263	1360.001798	-0.471458166	0.008192227	0.036515815
ENSG00000221821	C6orf226	112.983759	175.0377218	-0.634800346	0.00819339	0.036515815
ENSG00000223773	CD99P1	140.8073504	199.3323473	-0.50132765	0.008193785	0.036515815

ENSG00000224081	SLC44A3-AS1	33.2433515	62.37307605	-0.901365991	0.008205095	0.03655577
ENSG00000130203	APOE	37.48234667	69.87345052	-0.8954109	0.00820621	0.03655577
ENSG00000111247	RAD51AP1	869.0413573	1186.105637	-0.449319031	0.00820868	0.03655577
ENSG00000136854	STXBP1	2324.292363	1904.451391	0.287212756	0.008208707	0.03655577
ENSG00000135336	ORC3	570.1284792	752.5723093	-0.401452234	0.008215596	0.036579813
ENSG00000161381	PLXDC1	51.6249161	89.41897849	-0.791493486	0.008219045	0.036588536
ENSG00000284526	AC015802.6	9.808282485	25.50572442	-1.376722315	0.008224766	0.036607366
ENSG00000184599	FAM19A3	2.156046052	11.55963977	-2.418166618	0.008249698	0.03670547
ENSG00000214309	MBLAC1	75.93918103	116.2077911	-0.611989555	0.008250069	0.03670547
ENSG00000174738	NR1D2	3316.022659	3987.951599	-0.266245763	0.008251889	0.03670547
ENSG00000130818	ZNF426	1232.843405	993.3156529	0.312496503	0.008252788	0.03670547
ENSG00000100258	LMF2	2882.036521	2399.005802	0.264559993	0.008258106	0.036722471
ENSG00000261713	SSTR5-AS1	24.39370047	7.762727832	1.645103787	0.008268916	0.036763883
ENSG00000110429	FBXO3	1327.466373	1594.278662	-0.263935458	0.008276227	0.036789725
ENSG00000129255	MPDU1	1426.690353	1103.953396	0.369343792	0.008286201	0.036827392
ENSG00000150527	MIA2	1662.143915	2075.511345	-0.320364661	0.008299082	0.036877965
ENSG00000260702	AL031713.1	17.88005403	5.254898154	1.781190049	0.008300853	0.036879159
ENSG00000174748	RPL15	33826.65149	40143.82117	-0.247003044	0.008303269	0.036883218
ENSG00000091542	ALKBH5	2933.131357	2390.020874	0.295224208	0.008305509	0.036886496
ENSG00000219891	ZSCAN12P1	205.2447064	283.206751	-0.46595981	0.008309942	0.03689951
ENSG00000183458	AC138932.1	555.8741309	738.1257646	-0.40826541	0.008337658	0.037015886
ENSG00000127324	TSPAN8	297.8598855	788.4941998	-1.4042087	0.008343937	0.037037062
ENSG00000170310	STX8	601.401451	476.8492808	0.335445507	0.008346826	0.037043188
ENSG00000264448	AC084346.2	25.71209763	9.508657955	1.42751588	0.00835166	0.037057943
ENSG00000165416	SUGT1	1181.346615	1459.526807	-0.305617245	0.008355308	0.037067433
ENSG00000170430	MGMT	878.8088467	715.1987529	0.297652066	0.008370153	0.03712658
ENSG00000091947	TMEM101	1068.052534	1311.998832	-0.2963305	0.008385773	0.037189148
ENSG00000136040	PLXNC1	74.77569368	118.3265535	-0.6623124	0.008398482	0.037238781
ENSG00000240038	AMY2B	49.61204746	25.30479314	0.97514624	0.00840668	0.037268401
ENSG00000228629	AC092295.1	8.019610705	0.909304906	3.144691408	0.008420987	0.037325086
ENSG00000175387	SMAD2	4145.313634	3483.783557	0.250673937	0.008423015	0.037327336
ENSG00000034713	GABARAPL2	1535.092581	1279.269237	0.262943399	0.008440169	0.037390705
ENSG00000071626	DAZAP1	7419.728374	9416.449039	-0.343929422	0.00844036	0.037390705
ENSG00000160991	ORAI2	1239.159501	1015.75349	0.287235104	0.00844683	0.037412614
ENSG00000254233	LINC02365	0.747750911	9.582797005	-3.704665268	0.008457177	0.037451687
ENSG00000170776	AKAP13	3107.2776	2595.57245	0.259636667	0.008459959	0.03745725
ENSG00000104375	STK3	829.2947935	1090.292767	-0.395138949	0.008482838	0.037551777
ENSG00000143622	RIT1	666.3989795	841.2391069	-0.335434411	0.008496552	0.037605708
ENSG00000233392	AC104809.2	0	4.972439982	-4.626060485	0.008507106	0.03764563
ENSG00000206262	FOXL2NB	37.89253793	15.53627053	1.298842664	0.008530177	0.037740923
ENSG00000116128	BCL9	2121.582798	1740.325875	0.285516147	0.008544046	0.037789732
ENSG00000112539	C6orf118	1.083889228	9.983200212	-3.204314046	0.008544287	0.037789732
ENSG00000143514	TP53BP2	1882.634591	2258.219483	-0.262537016	0.008556406	0.037836512

ENSG00000237149	ZNF503-AS2	186.1195147	130.221148	0.517875253	0.008561162	0.037850729
ENSG00000136280	CCM2	1075.782959	846.77735	0.344626649	0.008588987	0.037966909
ENSG00000125863	MKKS	572.2997395	441.4612189	0.373634145	0.008610667	0.038055892
ENSG00000254859	AC067930.4	3.638545507	15.58145758	-2.091573985	0.008619342	0.038087375
ENSG00000276851	AC002401.4	8.680154934	1.240898196	2.834553285	0.008627627	0.038117126
ENSG00000165525	NEMF	1213.039937	1485.946176	-0.292882341	0.008636014	0.03814131
ENSG00000149781	FERMT3	149.7102809	95.88713832	0.644444232	0.008638395	0.03814131
ENSG00000182134	TDRKH	500.0687079	664.3259274	-0.410810975	0.00863853	0.03814131
ENSG00000140022	STON2	185.8155684	121.8121336	0.608410826	0.008639315	0.03814131
ENSG00000120832	MTERF2	297.9800634	395.0061842	-0.405451784	0.008644109	0.038155613
ENSG00000228526	MIR34AHG	149.2847099	207.9115172	-0.475909516	0.008649566	0.038172835
ENSG00000269729	AC006262.2	29.37933917	12.60450581	1.218274505	0.008666931	0.038242598
ENSG00000137802	MAPKBP1	722.4268642	888.4392596	-0.297905582	0.008677474	0.038282238
ENSG00000133106	EPSTI1	8.010569595	26.39484622	-1.718346125	0.008682542	0.038297715
ENSG00000178297	TMPRSS9	283.6124925	429.2734029	-0.596652605	0.008689109	0.038319796
ENSG00000213995	NAXD	2082.733075	1747.965732	0.252651114	0.008700668	0.038363882
ENSG00000107175	CREB3	1674.639392	1386.597809	0.27202763	0.008702623	0.038365614
ENSG00000250072	SH3TC2-DT	11.01061435	1.478169325	2.877752332	0.008706226	0.038374606
ENSG00000168385	2-Sep	13658.05707	15889.60799	-0.218340725	0.008714943	0.038406134
ENSG00000144504	ANKMY1	1051.646569	826.7087173	0.347778129	0.008717818	0.038411908
ENSG00000156136	DCK	1108.701442	1459.706957	-0.397424325	0.008720001	0.038414633
ENSG00000111696	NT5DC3	1655.419769	1282.49766	0.367815321	0.008732487	0.038462734
ENSG00000198874	TYW1	765.1745406	932.091237	-0.28438732	0.008747174	0.038518133
ENSG00000246273	SBF2-AS1	93.83128142	148.808666	-0.662964593	0.008748212	0.038518133
ENSG00000218891	ZNF579	966.4656506	1177.975129	-0.2857943	0.008749771	0.038518133
ENSG00000224577	LINC01117	5.807593926	0.331593289	4.159955786	0.008754921	0.038526988
ENSG00000233355	CHRM3-AS2	5.807593926	0.331593289	4.159955786	0.008754921	0.038526988
ENSG00000133138	TBC1D8B	795.7706577	639.3259768	0.316158797	0.008762298	0.038552543
ENSG00000177692	DNAJC28	83.60981364	51.37551221	0.702191437	0.008766108	0.038562392
ENSG00000184432	COPB2	10579.74269	8828.458125	0.260990033	0.008778665	0.038610713
ENSG00000131018	SYNE1	1.78867178	10.57121839	-2.548902952	0.008780912	0.038613681
ENSG00000278058	AC009159.3	13.13161456	2.665033279	2.285888444	0.008795678	0.038671684
ENSG00000234883	MIR155HG	4.056659286	18.02158499	-2.165006989	0.008806828	0.038713776
ENSG00000232724	TRIM80P	4.728935919	16.96040562	-1.83729994	0.008814815	0.038741947
ENSG00000233246	AL513327.2	20.41647672	6.825930279	1.56883708	0.008869929	0.0389772
ENSG00000130479	MAP1S	2312.565895	1799.699222	0.361304767	0.008875068	0.038992805
ENSG00000182809	CRIP2	86.64251569	54.19920845	0.679605584	0.008883742	0.039023931
ENSG00000159459	UBR1	1267.655846	1532.399765	-0.273785353	0.008886249	0.039025721
ENSG00000165475	CRYL1	740.3178572	938.3013961	-0.340938006	0.008887329	0.039025721
ENSG00000263326	AC133552.4	22.64799705	8.045694314	1.506929343	0.008892912	0.039035891
ENSG00000235029	MNX1-AS2	17.0164004	36.13836489	-1.085110589	0.008893842	0.039035891
ENSG00000140465	CYP1A1	92.2208329	136.5083519	-0.567180853	0.008894415	0.039035891
ENSG00000214655	ZSWIM8	2302.710804	1891.19291	0.284485106	0.00890852	0.03909081

ENSG00000148341	SH3GLB2	6738.233463	8853.028166	-0.393697938	0.008924762	0.039146684
ENSG00000233806	LINC01237	107.8094346	69.33164874	0.638726751	0.008925276	0.039146684
ENSG00000114670	NEK11	139.5802838	89.32304036	0.648376329	0.008927583	0.039146684
ENSG00000101421	CHMP4B	3162.2121	2566.74304	0.300701424	0.008927632	0.039146684
ENSG00000272622	AC010735.2	19.47861878	6.204490979	1.670775957	0.008944495	0.039213626
ENSG00000106100	NOD1	359.2677185	466.052661	-0.375755184	0.008956549	0.039254623
ENSG00000143862	ARL8A	1099.166007	1355.919977	-0.302534523	0.008957044	0.039254623
ENSG00000100483	VCPKMT	371.3127413	284.2289579	0.384067321	0.008964963	0.039282316
ENSG00000065413	ANKRD44	80.75136238	125.307707	-0.631540806	0.008974004	0.039314911
ENSG00000150867	PIP4K2A	1044.465272	815.3235722	0.356229502	0.008982607	0.039345582
ENSG00000136938	ANP32B	8205.373248	9816.809716	-0.258774237	0.008989398	0.039368306
ENSG00000181513	ACBD4	811.1415272	1055.587227	-0.379132535	0.009003965	0.039425067
ENSG00000279095	AC243964.3	615.9672057	468.8378276	0.392861453	0.009024149	0.039506399
ENSG00000181097	BREA2	46.95972206	23.0544177	1.028870753	0.009038405	0.039561754
ENSG00000006831	ADIPOR2	3745.402405	2967.402454	0.33561498	0.009061852	0.039657313
ENSG00000260805	AC092803.2	88.83629888	56.25363003	0.658729835	0.00906558	0.039666556
ENSG00000136156	ITM2B	5140.440382	6158.90645	-0.260618132	0.009071382	0.03968487
ENSG00000160695	VPS11	1652.464394	1369.917452	0.270267633	0.00909183	0.03976724
ENSG00000137726	FXYD6	41.98343842	18.21512844	1.201790685	0.009093773	0.039768504
ENSG00000066135	KDM4A	959.7705877	1199.986336	-0.322127484	0.009095359	0.039768504
ENSG00000168216	LMBRD1	1072.182607	1359.863917	-0.342328429	0.009099182	0.039778135
ENSG00000166558	SLC38A8	71.85382572	39.84233573	0.857420845	0.009107674	0.039808173
ENSG00000233903	Z83851.1	75.4091258	44.09712488	0.772272091	0.009111766	0.039818968
ENSG00000235403	AC114808.1	11.9655985	2.204237344	2.473315961	0.009120194	0.039848706
ENSG00000177000	MTHFR	1122.700937	1526.211573	-0.442426701	0.009142269	0.039938049
ENSG00000100983	GSS	3429.654226	2606.25605	0.39576181	0.009154301	0.039983497
ENSG00000158109	TPRG1L	1081.96507	877.7822658	0.302165445	0.009169649	0.040039955
ENSG00000182944	EWSR1	10792.72753	12729.17637	-0.238118199	0.009170489	0.040039955
ENSG00000119547	ONECUT2	449.534813	335.239104	0.421150099	0.009184334	0.040093278
ENSG00000174903	RAB1B	2529.603511	2051.700113	0.301865702	0.009191817	0.040118807
ENSG00000228544	CCDC183-AS1	171.4844646	268.4372794	-0.644059026	0.009204861	0.040168598
ENSG00000263528	IKBKE	321.6014296	244.8791322	0.392635507	0.009211298	0.040186031
ENSG00000007516	BAIAP3	135.1462531	236.6487547	-0.805875353	0.009212129	0.040186031
ENSG00000186710	CFAP73	27.11658288	10.6292007	1.357089239	0.009244546	0.040317632
ENSG00000012174	MBTPS2	1262.620261	1600.799762	-0.342945136	0.009245581	0.040317632
ENSG00000133687	TMTC1	5.86229469	0.300152569	4.170876735	0.009260773	0.040371889
ENSG00000179950	PUF60	4527.375572	3472.285316	0.382511967	0.009261312	0.040371889
ENSG00000247345	AC092343.1	11.5449448	2.073575339	2.465858587	0.009280208	0.040447077
ENSG00000126091	ST3GAL3	135.3633209	92.16751906	0.553431438	0.009282573	0.040450205
ENSG00000226153	Z93242.1	5.86229469	0.277559048	4.170882912	0.009305465	0.040540284
ENSG00000204564	C6orf136	483.1789866	623.636855	-0.369099814	0.009307444	0.040540284
ENSG00000182986	ZNF320	426.8798382	295.104811	0.534016637	0.009308199	0.040540284
ENSG00000272275	AC092687.3	35.42127844	62.7720199	-0.828199219	0.009312863	0.040553407

ENSG00000058804	NDC1	2629.748377	3530.104977	-0.425037488	0.009317194	0.040565071
ENSG00000131398	KCNC3	525.6742466	331.7365587	0.665719027	0.009333459	0.040628679
ENSG00000075624	ACTB	90506.99457	64808.12732	0.481840391	0.009335167	0.040628911
ENSG00000089775	ZBTB25	928.7906458	1123.494297	-0.274363576	0.009338394	0.04063575
ENSG00000100852	ARHGAP5	2811.569906	3390.325227	-0.270116161	0.009356039	0.040705319
ENSG00000250740	AC109361.2	3.712749822	16.66420112	-2.176177382	0.009370212	0.040757371
ENSG00000253372	AC016405.1	4.705471111	17.33572659	-1.876895882	0.009371323	0.040757371
ENSG00000169231	THBS3	452.1113473	663.467125	-0.552154182	0.009376511	0.040772708
ENSG00000213600	U73169.1	11.68162102	2.351134386	2.291617232	0.009384333	0.040799498
ENSG00000104067	TJP1	8053.072545	9969.172254	-0.307996174	0.009388067	0.040808502
ENSG00000005810	MYCBP2	3162.726162	2523.227405	0.325524673	0.009399602	0.040851411
ENSG00000228409	CCT6P1	239.8858906	318.0548349	-0.405814779	0.009408496	0.04088283
ENSG00000157020	SEC13	3416.29814	2694.20195	0.34220716	0.009411496	0.040888629
ENSG00000197774	EME2	1813.082767	2568.582217	-0.502262951	0.009413681	0.040890885
ENSG00000223343	AC137630.1	22.29362514	45.00444938	-1.014377075	0.009421543	0.040913471
ENSG00000115414	FN1	200.7423447	288.0751746	-0.519917383	0.009422214	0.040913471
ENSG00000078070	MCCC1	768.9941632	938.0179738	-0.286735048	0.009428086	0.04093032
ENSG00000006607	FARP2	1969.528758	2461.385134	-0.322017055	0.009429428	0.04093032
ENSG00000231535	LINC00278	12.42937193	30.25069152	-1.283410488	0.009448608	0.041006324
ENSG00000278291	AL161772.1	124.68917	79.53837376	0.649584909	0.009452526	0.041016077
ENSG00000279145	AC011912.1	25.26670916	50.22395874	-0.98523054	0.009454588	0.041017776
ENSG00000138587	MNS1	11.84715586	30.125958	-1.342750288	0.009475838	0.041102703
ENSG00000072274	TFRC	8516.10707	6490.364526	0.391734223	0.009483666	0.041129393
ENSG00000076201	PTPN23	3789.86287	3100.782577	0.289246528	0.009492802	0.04116174
ENSG00000180879	SSR4	2912.099499	3515.854824	-0.27180002	0.009498201	0.041177882
ENSG00000164414	SLC35A1	185.6467002	133.5455764	0.474842861	0.009502101	0.041187513
ENSG00000103260	METRN	1632.630148	1358.49564	0.264835637	0.009529684	0.041299783
ENSG00000173011	TADA2B	1176.910989	1410.263269	-0.261178246	0.009532262	0.041303665
ENSG00000147394	ZNF185	1128.22461	1424.126278	-0.33549044	0.009539191	0.041326396
ENSG00000177051	FBXO46	1018.312246	1268.792613	-0.316989257	0.009550686	0.041359605
ENSG00000150459	SAP18	2689.519215	3230.439005	-0.264420406	0.00955077	0.041359605
ENSG00000137642	SORL1	4181.592385	5376.409401	-0.362367254	0.009551911	0.041359605
ENSG00000164626	KCNK5	613.3148662	765.9961107	-0.320299745	0.009573021	0.041443703
ENSG00000118804	STBD1	117.6594155	167.3765346	-0.509482549	0.009594038	0.041527368
ENSG00000167476	JSRP1	1338.900624	1096.341838	0.287925591	0.009607668	0.041575434
ENSG00000199023	MIR339	1.795172964	11.12779584	-2.615924535	0.00960853	0.041575434
ENSG00000115282	TTC31	1149.00704	1387.417365	-0.271788394	0.009615247	0.04159717
ENSG00000279982	AL162274.3	86.40927771	54.32936214	0.669960971	0.009637278	0.041685133
ENSG00000173480	ZNF417	478.7001009	354.2921862	0.436110396	0.009641312	0.041695234
ENSG00000054148	PHPT1	1644.092192	2138.457507	-0.378906186	0.009649726	0.041724272
ENSG00000133121	STARD13	139.7994008	83.35407501	0.745799204	0.009659534	0.041759328
ENSG00000185716	MOSMO	598.656842	473.5336213	0.339138032	0.00971694	0.042000102
ENSG00000166033	HTRA1	81.85237782	49.59178288	0.719627463	0.009736615	0.04207774

ENSG00000236266	Z98884.1	15.92854992	4.515083812	1.818503459	0.009742374	0.042095216
ENSG00000133985	TTC9	1052.009385	1414.279956	-0.426295076	0.009783683	0.042266266
ENSG00000281420	AP001052.1	48.87206769	25.97536755	0.9111084	0.009788696	0.042280486
ENSG00000168306	ACOX2	12.73554426	36.25322187	-1.504164655	0.009798618	0.042315895
ENSG00000154620	TMSB4Y	188.137138	263.9890375	-0.491451748	0.009859972	0.042573368
ENSG00000134070	IRAK2	53.67251663	90.11456957	-0.751844063	0.009863485	0.042580479
ENSG00000254343	AC091563.1	5.875297058	18.90821807	-1.690901574	0.009865087	0.042580479
ENSG00000172775	FAM192A	2812.667712	2368.942726	0.247438336	0.009872462	0.042604817
ENSG00000068001	HYAL2	1793.909819	1387.059245	0.370841992	0.009878921	0.042625201
ENSG00000178467	P4HTM	2599.416052	3331.971917	-0.357929597	0.009883736	0.042631129
ENSG00000136875	PRPF4	1883.714889	1431.397576	0.395638818	0.009885468	0.042631129
ENSG00000153814	JAZF1	185.6378216	130.2836778	0.510389615	0.00988593	0.042631129
ENSG00000162910	MRPL55	964.7053243	746.2712936	0.369560226	0.009888812	0.042631129
ENSG00000144320	LNPK	1356.91598	1061.91799	0.353050523	0.009888977	0.042631129
ENSG00000169403	PTAFR	80.07797837	45.59401795	0.806011321	0.009892729	0.042639814
ENSG00000156076	WIF1	7.938905207	0.886711385	3.145875081	0.009902236	0.042673303
ENSG00000167468	GPX4	4863.879393	6062.328392	-0.317878203	0.009906961	0.042681956
ENSG00000225285	LINC01770	14.78615004	34.27748675	-1.204609344	0.009907721	0.042681956
ENSG00000166949	SMAD3	2273.780517	1874.808014	0.278619927	0.009916439	0.042710402
ENSG00000145934	TENM2	38.29129953	16.7629141	1.195205775	0.009917804	0.042710402
ENSG00000205863	C1QTNF9B	17.18304262	5.164524067	1.737533788	0.009919938	0.0427121
ENSG00000164442	CITED2	1620.745114	2014.403955	-0.313897908	0.009938921	0.04278633
ENSG00000233757	AC092835.1	52.50173468	27.99344929	0.90210019	0.009951325	0.042832218
ENSG00000180573	HIST1H2AC	127.2566548	233.0660649	-0.870964373	0.00997136	0.042910931
ENSG00000143633	C1orf131	477.2965716	598.9576444	-0.328361153	0.009981346	0.042946376
ENSG00000069812	HES2	96.25417874	145.3516862	-0.593076703	0.009990527	0.042973734
ENSG00000207340	RNVU1-1	0.698281368	8.206337679	-3.510683233	0.009991205	0.042973734
ENSG00000130829	DUSP9	13.10814975	2.589864876	2.387483901	0.009999209	0.043000629
ENSG00000253616	AC107959.3	45.16581906	21.54670976	1.069454227	0.010016595	0.043067852
ENSG00000005889	ZFX	1259.813362	1522.716533	-0.273203497	0.010021989	0.043083496
ENSG00000135269	TES	2596.674984	3137.088281	-0.272710358	0.010025643	0.043086407
ENSG00000204706	MAMDC2-AS1	21.31025892	7.619778864	1.492154426	0.010027356	0.043086407
ENSG00000197647	ZNF433	76.32701487	42.44361482	0.844682307	0.010027931	0.043086407
ENSG00000147548	NSD3	3893.010587	4644.583711	-0.254828063	0.010040884	0.043134512
ENSG00000119718	EIF2B2	1147.089614	858.6810649	0.416831387	0.010055962	0.043191728
ENSG00000141519	CCDC40	350.007865	254.5855551	0.460883692	0.01006163	0.043198847
ENSG00000236137	AL445231.1	33.08685777	15.25089364	1.112205207	0.010062794	0.043198847
ENSG00000003509	NDUFAF7	881.228565	716.7542403	0.297309592	0.010062898	0.043198847
ENSG00000127914	AKAP9	3524.232872	4206.473907	-0.255358973	0.010075569	0.043240273
ENSG00000148175	STOM	140.7553409	92.95259875	0.596358075	0.01007607	0.043240273
ENSG00000125971	DYNLRB1	2287.854873	1830.775242	0.321149338	0.010079143	0.0432459
ENSG00000129749	CHRNA10	34.63706036	66.76831273	-0.943882032	0.010100582	0.043330313
ENSG00000227354	RBM26-AS1	77.96331676	122.0743303	-0.646986824	0.010103948	0.043336808

ENSG00000184557	SOCS3	109.9156972	62.02326732	0.832794448	0.010105626	0.043336808
ENSG00000236255	AC009404.1	293.8304785	211.2387077	0.476913109	0.010131027	0.043438149
ENSG00000156500	FAM122C	262.9589791	198.087501	0.410199784	0.010151177	0.043516946
ENSG00000176387	HSD11B2	174.584881	120.7709934	0.532190245	0.010154901	0.04352531
ENSG00000129515	SNX6	2542.011352	3045.910111	-0.260925868	0.010158405	0.043532728
ENSG00000277290	AC136475.10	57.60819336	91.91101587	-0.67512933	0.010171247	0.043573431
ENSG00000276293	PIP4K2B	3214.244494	3786.467587	-0.236264705	0.010171453	0.043573431
ENSG00000185495	AC138393.1	334.9056998	501.7142158	-0.582784871	0.010211099	0.043735641
ENSG00000170315	UBB	4946.44514	7065.171939	-0.514393366	0.010217329	0.043754689
ENSG00000237765	FAM200B	514.4557711	637.8165234	-0.310642989	0.010238889	0.043839374
ENSG00000217733	CCT7P1	1.06565564	8.730015054	-3.012506262	0.010254679	0.043899324
ENSG00000068745	IP6K2	3096.543705	3798.976625	-0.294676892	0.010262875	0.043926751
ENSG00000050130	JKAMP	1537.006382	1283.612329	0.260120544	0.010279001	0.043988103
ENSG00000169129	AFAP1L2	80.20307356	120.9567063	-0.589927476	0.01028219	0.043990643
ENSG00000138964	PARVG	0	4.501337488	-4.489647894	0.010284933	0.043990643
ENSG00000085760	MTIF2	1846.597206	2247.980586	-0.284072117	0.01028497	0.043990643
ENSG00000114251	WNT5A	7.290093382	0.555118095	3.627540235	0.010286857	0.04399105
ENSG00000134042	MRO	39.12514974	18.7741163	1.07097422	0.010298974	0.044035199
ENSG00000107185	RGP1	2177.486809	1822.142937	0.257117585	0.010312124	0.044083745
ENSG00000227619	AL391056.1	75.62635616	116.5522707	-0.627056581	0.010321302	0.044115299
ENSG00000136908	DPM2	1505.748007	1149.576926	0.388602869	0.010339125	0.044183787
ENSG00000258245	RPL10P13	47.6137651	25.07345049	0.919544716	0.010341212	0.044185014
ENSG00000165949	IFI27	25.13764149	57.71445666	-1.196620749	0.010388703	0.044380208
ENSG00000227627	AL080276.2	5.85706347	18.36048782	-1.654828445	0.010398368	0.044413769
ENSG00000167657	DAPK3	1914.720805	1594.646815	0.263611026	0.0104169	0.044485182
ENSG00000025772	TOMM34	2284.203502	1709.924872	0.4172178	0.010421671	0.044497816
ENSG00000197620	CXorf40A	320.4935961	229.0431519	0.484150934	0.010433164	0.044539141
ENSG00000167216	KATNAL2	221.6527346	164.3464869	0.431445851	0.010438146	0.044552664
ENSG00000188483	IER5L	2615.631949	2046.821796	0.353848894	0.010443123	0.044566159
ENSG00000270049	AC009061.2	111.2264745	70.20658147	0.668218006	0.01044964	0.044586221
ENSG00000171150	SOCS5	1077.396415	887.917302	0.278536582	0.010455041	0.044599046
ENSG00000143183	TMCO1	2732.121996	2299.015227	0.249092891	0.010456279	0.044599046
ENSG00000272320	AL445309.1	55.44833741	29.8852471	0.890853937	0.010460651	0.044609945
ENSG00000198814	GK	313.6493819	415.5389346	-0.406873058	0.010463482	0.044613907
ENSG00000166415	WDR72	207.1400884	145.26913	0.514291378	0.010465215	0.044613907
ENSG00000181800	CELF2-AS1	15.68627084	4.115709957	1.912421501	0.01047684	0.044655711
ENSG00000226578	AL132657.1	31.27741248	11.4362873	1.4495538	0.010486603	0.044686383
ENSG00000122711	SPINK4	0.741249728	8.014253593	-3.450467767	0.010490064	0.044686383
ENSG00000196712	NF1	6644.514984	7816.734057	-0.234356342	0.010490527	0.044686383
ENSG00000116586	LAMTOR2	1046.334004	822.038346	0.347480307	0.010491316	0.044686383
ENSG00000064115	TM7SF3	2591.025751	2196.633961	0.238040457	0.010509132	0.044749775
ENSG00000240350	AC017002.3	5.739890795	0.300152569	4.146250977	0.010509845	0.044749775
ENSG00000178980	SELENOW	1463.946894	1201.694663	0.284225935	0.010535852	0.044852731

ENSG00000275234	AC010503.4	371.4494288	476.4650344	-0.358330437	0.010579798	0.045032008
ENSG00000170959	DCDC1	23.33200609	8.855777935	1.404269707	0.010582802	0.045035467
ENSG00000163888	CAMK2N2	210.4069703	138.1315289	0.604226839	0.010584279	0.045035467
ENSG00000242841	KRT8P35	7.357796513	0.631745859	3.585422355	0.010589407	0.045044332
ENSG00000258458	AL160314.2	37.47838541	18.37328309	1.027311472	0.010591237	0.045044332
ENSG00000161664	ASB16	224.4380777	150.850644	0.57628529	0.010591867	0.045044332
ENSG00000173611	SCAI	810.1879897	983.2953438	-0.278897381	0.01059593	0.045053807
ENSG00000165197	VEGFD	9.861713285	1.41038876	2.748920163	0.010608344	0.045098778
ENSG00000227597	WASF1P1	4.417532374	19.40827257	-2.137216019	0.010621013	0.045144821
ENSG00000243753	HLA-L	13.83639711	32.6460619	-1.236479372	0.010629883	0.045174702
ENSG00000008277	ADAM22	554.2066043	716.426231	-0.369291377	0.0106431	0.045221781
ENSG00000188732	FAM221A	581.8116983	740.5289271	-0.348286724	0.010644645	0.045221781
ENSG00000186918	ZNF395	1803.337338	1498.057937	0.267252744	0.010648591	0.045222949
ENSG00000129474	AJUBA	11695.041	9765.154885	0.260265394	0.010648604	0.045222949
ENSG00000162728	KCNJ9	12.17156178	2.472949193	2.320914418	0.010664856	0.045277993
ENSG00000133398	MED10	1254.709399	1027.973463	0.286906826	0.010667139	0.045277993
ENSG00000254057	AC084346.1	22.88519628	8.211236804	1.483122014	0.010667741	0.045277993
ENSG00000115520	COQ10B	772.2659653	559.6051091	0.463162528	0.010668942	0.045277993
ENSG00000255153	TOLLIP-AS1	43.92971131	77.21582694	-0.813319981	0.010673509	0.045289547
ENSG00000142207	URB1	3208.58333	2500.683336	0.359235677	0.010698212	0.045386524
ENSG00000072786	STK10	641.9525015	498.2061473	0.364363511	0.010705756	0.045410682
ENSG00000187642	PERM1	92.99189725	136.4404147	-0.552228702	0.010708408	0.045414081
ENSG00000115297	TLX2	22.81226193	8.654846651	1.414564137	0.01072133	0.045461031
ENSG00000250673	REELD1	17.44878651	5.209711111	1.754015287	0.01072524	0.045469757
ENSG00000166813	KIF7	879.0406267	1147.864652	-0.385261904	0.010730251	0.045483147
ENSG00000204540	PSORS1C1	117.6841503	72.16316283	0.711276415	0.010737554	0.045506247
ENSG00000259299	AC061965.1	4.323824508	0	4.697816516	0.010748663	0.045545466
ENSG00000107223	EDF1	4833.169955	5913.020005	-0.290910292	0.010780822	0.045673848
ENSG00000276216	AC245014.3	11.3220179	2.42776215	2.224866848	0.010792538	0.045715598
ENSG00000198400	NTRK1	26.49250583	10.01464093	1.39050347	0.010823117	0.045837215
ENSG00000187240	DYNC2H1	328.0784575	444.378221	-0.436296064	0.010833717	0.045874195
ENSG00000235568	NFAM1	5.377747744	18.82420247	-1.793222175	0.010863312	0.045991579
ENSG00000273188	AL022328.3	62.94709658	112.4515188	-0.832509931	0.010879892	0.046053832
ENSG00000163006	CCDC138	428.782697	580.8406038	-0.438814474	0.010884587	0.046059553
ENSG00000090263	MRPS33	681.9089504	832.5777194	-0.288147247	0.010886201	0.046059553
ENSG00000084733	RAB10	6984.295519	5948.882054	0.231563514	0.010886872	0.046059553
ENSG00000180776	ZDHHC20	4728.212127	5545.592257	-0.230033927	0.010921542	0.046198274
ENSG00000146232	NFKBIE	421.2168401	311.2734137	0.434153208	0.010924731	0.046203803
ENSG00000116017	ARID3A	1331.182142	904.1975946	0.558772604	0.010939516	0.046258364
ENSG00000122965	RBM19	1752.228219	1433.59647	0.288929327	0.010950395	0.046293561
ENSG00000235010	AL512622.1	5.745122016	0.300152569	4.147279298	0.010951611	0.046293561
ENSG00000107959	PITRM1	2573.174729	2061.208549	0.319624857	0.010956554	0.046306483
ENSG00000265817	FSBP	4.624765612	16.92260641	-1.856289364	0.010975955	0.046380496

ENSG00000136874	STX17	1339.86246	1105.13927	0.277373899	0.010981718	0.046396862
ENSG00000183474	GTF2H2C	282.2996517	385.1708973	-0.44940864	0.010987113	0.046408546
ENSG00000137812	KNL1	1753.595503	2407.686767	-0.457594138	0.010988264	0.046408546
ENSG00000139926	FRMD6	462.9047425	588.2321939	-0.345182496	0.010995797	0.046432373
ENSG00000233718	MYCNOS	0	4.614305097	-4.520667863	0.011044174	0.046628634
ENSG00000104936	DMPK	1190.005048	1638.333622	-0.460664746	0.01104613	0.046628875
ENSG00000227060	LINC00629	14.6596223	3.838150909	1.916099476	0.011052433	0.04664746
ENSG00000257167	TMPO-AS1	263.4706494	196.4659527	0.423395137	0.011061727	0.046673346
ENSG00000248713	C4orf54	7.314828153	0.600305139	3.598940445	0.011062368	0.046673346
ENSG00000187091	PLCD1	321.7360424	415.918816	-0.369033107	0.011073803	0.046713564
ENSG00000162729	IGSF8	1871.522661	1399.93232	0.418288849	0.011082449	0.046742002
ENSG00000145041	DCAF1	2120.614663	2640.48848	-0.316670427	0.011092987	0.046778414
ENSG00000081059	TCF7	940.4253484	779.0117034	0.27175399	0.011095013	0.046778924
ENSG00000134538	SLCO1B1	24.68814039	9.572998756	1.380963972	0.011113136	0.046847286
ENSG00000276855	AC015922.3	0	4.447303246	-4.474905212	0.011116603	0.046851505
ENSG00000123612	ACVR1C	23.00236895	8.869524258	1.381701066	0.011119947	0.046851505
ENSG00000168395	ING5	2097.383169	2462.741306	-0.231611616	0.011121612	0.046851505
ENSG00000198899	MT-ATP6	116376.4621	100090.0634	0.217499045	0.01112177	0.046851505
ENSG00000166275	BORCS7	254.9190744	356.4767614	-0.483164538	0.011125244	0.0468581
ENSG00000206712	RNU6-26P	17.34984743	4.694880934	1.90971864	0.011134391	0.046888582
ENSG00000119431	HDHD3	935.9583352	1138.215572	-0.282725088	0.011188729	0.047109328
ENSG00000243479	MNX1-AS1	77.36570976	117.6948204	-0.606682354	0.011208764	0.047185589
ENSG00000079950	STX7	2048.814428	1713.539433	0.258223988	0.011224656	0.047244392
ENSG00000048828	FAM120A	7930.456901	9444.703104	-0.252177583	0.011255691	0.047366894
ENSG00000070601	FRMPD1	15.29543176	32.76495799	-1.101911812	0.011262547	0.047387626
ENSG00000214078	CPNE1	3141.392777	2569.51966	0.289832093	0.011267672	0.047396822
ENSG00000160813	PPP1R35	507.9015532	676.5304728	-0.414025826	0.011269634	0.047396822
ENSG00000178403	NEUROG2	21.97191053	7.506811255	1.544769227	0.011270525	0.047396822
ENSG00000132661	NXT1	570.2813144	419.8224023	0.440173913	0.011274299	0.047404575
ENSG00000267319	SELENOKP1	11.28951198	2.472949193	2.208800553	0.01127851	0.047414163
ENSG00000178950	GAK	4727.592117	3991.23326	0.244197936	0.011293608	0.047469505
ENSG00000139428	MMAB	1337.588433	1096.878559	0.286547295	0.011298645	0.047482547
ENSG00000100865	CINP	685.8652492	550.0011254	0.317626229	0.011310719	0.047525152
ENSG00000175066	GK5	1102.50838	1426.427078	-0.372242054	0.011323261	0.047569709
ENSG00000121577	POPDC2	47.87680648	23.3063862	1.038286824	0.011353993	0.047690656
ENSG00000127824	TUBA4A	3226.974653	1630.703905	0.984510193	0.011362325	0.04771749
ENSG00000228253	MT-ATP8	2910.796664	2434.797225	0.257519139	0.011368415	0.047734899
ENSG00000280332	AC020917.4	89.05415714	53.39256459	0.740328857	0.011417669	0.047933515
ENSG00000137776	SLTM	3460.377245	4190.08471	-0.276255135	0.011432645	0.047988181
ENSG00000265055	AC145343.1	48.93470219	23.40078666	1.05768437	0.011439545	0.048008936
ENSG00000214212	C19orf38	117.0121988	68.99163826	0.767891755	0.011443053	0.048015451
ENSG00000106006	HOXA6	116.8598177	184.306724	-0.6548151	0.011447175	0.048024536
ENSG00000173535	TNFRSF10C	22.08924579	43.27131453	-0.96474842	0.01145139	0.048033082

ENSG00000160471	COX6B2	9.425365919	26.07458884	-1.46558032	0.011453125	0.048033082
ENSG00000134253	TRIM45	235.9389468	314.897565	-0.414887133	0.011461693	0.048060807
ENSG00000230002	ALMS1-IT1	49.49503737	87.95713524	-0.831217054	0.011483978	0.048146028
ENSG00000130202	NECTIN2	3560.668895	4398.353412	-0.304944587	0.011529352	0.048328006
ENSG00000137500	CCDC90B	875.1035428	704.7464687	0.311576189	0.011536797	0.048350957
ENSG00000183971	NPW	15.63934122	33.86531763	-1.111751612	0.011542166	0.048365202
ENSG00000176049	JAKMIP2	11.31424675	2.226830866	2.383450561	0.01154778	0.048380468
ENSG00000163558	PRKCI	1909.765306	2381.691082	-0.318866345	0.011558823	0.04841847
ENSG00000213275	IFITM9P	5.427217287	0.277559048	4.063198323	0.01158027	0.048500036
ENSG00000172137	CALB2	12.18329418	2.705321197	2.17326917	0.011585266	0.048512685
ENSG00000113721	PDGFRB	31.27868244	58.97948604	-0.910858601	0.011595802	0.048546992
ENSG00000132429	POPDC3	37.33647796	18.38308134	1.022412425	0.011597414	0.048546992
ENSG00000219507	FTH1P8	19.65176218	7.188964289	1.445471969	0.01164788	0.04874335
ENSG00000211452	DIO1	33.27458746	15.52845269	1.095709965	0.011648293	0.04874335
ENSG00000159596	TMEM69	1068.799043	1332.722624	-0.319107155	0.011673097	0.04883882
ENSG00000174137	FAM53A	85.30604752	136.9816954	-0.678552521	0.011679832	0.048853272
ENSG00000125457	MIF4GD	949.3605856	1195.46634	-0.332864887	0.011681526	0.048853272
ENSG00000038532	CLEC16A	1647.820136	2065.574391	-0.326328674	0.011682521	0.048853272
ENSG00000151725	CENPU	1248.276834	1656.961651	-0.409096793	0.011686759	0.048862676
ENSG00000142279	WTIP	140.6797041	91.2302915	0.620379016	0.011691836	0.048875576
ENSG00000126870	WDR60	1009.234566	786.6044983	0.360682062	0.011693854	0.048875692
ENSG00000089682	RBM41	569.4711028	708.7866987	-0.315788302	0.011700224	0.04889399
ENSG00000025708	TYMP	53.72880132	89.13439604	-0.726895421	0.011734464	0.049028732
ENSG00000166734	CASC4	4832.264867	5673.843545	-0.231515527	0.01174208	0.049052205
ENSG00000143341	HMCN1	6.842013611	23.8884759	-1.791852385	0.011751307	0.049082398
ENSG00000159082	SYNJ1	504.876928	400.0620147	0.335107022	0.011762934	0.049122604
ENSG00000267904	AC024075.1	97.50455882	146.9452336	-0.590338702	0.011802438	0.049279194
ENSG00000204619	PPP1R11	2142.915739	1794.545748	0.25591202	0.01180807	0.049294327
ENSG00000142875	PRKACB	1042.81345	853.9877222	0.28888341	0.011838209	0.049396782
ENSG00000112183	RBM24	59.44031004	115.0919651	-0.954726873	0.011838497	0.049396782
ENSG00000230124	ACBD6	2029.293777	2398.599992	-0.241472035	0.011838649	0.049396782
ENSG00000110395	CBL	3626.109054	2928.342436	0.308050851	0.011851949	0.049443872
ENSG00000284630	AP000553.5	28.43481746	53.73885525	-0.920035656	0.011862178	0.04947422
ENSG00000109016	DHRS7B	623.2290796	477.3157573	0.383063579	0.011863253	0.04947422
ENSG00000175893	ZDHHC21	1112.638576	1388.221847	-0.319125917	0.011911068	0.049665188
ENSG00000124217	MOCS3	453.2797519	563.186965	-0.312688388	0.011937238	0.049765857
ENSG00000103264	FBXO31	1133.595198	1378.747586	-0.28258467	0.011942453	0.049776069
ENSG00000081277	PKP1	385.2783574	289.6840441	0.411496677	0.011943742	0.049776069
ENSG00000232218	AL021937.2	14.92678752	4.237524764	1.818239456	0.011948286	0.049781755
ENSG00000065883	CDK13	2852.069387	3374.414208	-0.242660372	0.011949162	0.049781755
ENSG00000241975	ELOCP19	22.76929357	45.0908754	-0.980006929	0.01197255	0.049870729
ENSG00000112031	MTRF1L	331.2100874	423.7922703	-0.356680818	0.011976817	0.049880041
ENSG00000165209	STRBP	2393.276821	2859.104496	-0.256746169	0.011983153	0.049897964

ENSG00000068308	OTUD5	2031.11902	2486.663265	-0.291870975	0.011999736	0.049958542
